# Dermal Blimp1 Acts Downstream of Epidermal TGFβ and Wnt/β-Catenin to Regulate Hair Follicle Formation and Growth

**DOI:** 10.1016/j.jid.2017.06.015

**Published:** 2017-11

**Authors:** Stephanie B. Telerman, Emanuel Rognoni, Inês Sequeira, Angela Oliveira Pisco, Beate M. Lichtenberger, Oliver J. Culley, Priyalakshmi Viswanathan, Ryan R. Driskell, Fiona M. Watt

**Affiliations:** 1 King’s College London Centre for Stem Cells and Regenerative Medicine, Great Maze Pond, London, UK; 2 Medical University of Vienna, Skin and Endothelium Research Division (SERD), Department of Dermatology, Vienna, Austria

**Keywords:** Blimp1, B-lymphocyte-induced maturation protein 1, BMP, bone morphogenic protein, DP, dermal papilla, EdU, 5-ethynyl-2’-deoxyuridine, FGF, fibroblast growth factor, GF, growth factor, HC, hair cycle, HF, hair follicle, qPCR, quantitative PCR, TGFβ, transforming growth factor-β

## Abstract

B-lymphocyte-induced maturation protein 1 (Blimp1) is a transcriptional repressor that regulates cell growth and differentiation in multiple tissues, including skin. Although in the epidermis Blimp1 is important for keratinocyte and sebocyte differentiation, its role in dermal fibroblasts is unclear. Here we show that Blimp1 is dynamically regulated in dermal papilla cells during hair follicle (HF) morphogenesis and the postnatal hair cycle, preceding dermal Wnt/β-catenin activation. Blimp1 ablation in E12.5 mouse dermal fibroblasts delayed HF morphogenesis and growth and prevented new HF formation after wounding. By combining targeted quantitative PCR screens with bioinformatic analysis and experimental validation we demonstrated that Blimp1 is both a target and a mediator of key dermal papilla inductive signaling pathways including transforming growth factor-β and Wnt/β-catenin. Epidermal overexpression of stabilized β-catenin was able to override the HF defects in Blimp1 mutant mice, underlining the close reciprocal relationship between the dermal papilla and adjacent HF epithelial cells. Overall, our study reveals the functional role of Blimp1 in promoting the dermal papilla inductive signaling cascade that initiates HF growth.

## Introduction

B-lymphocyte-induced maturation protein 1 (Blimp1; Prdm1) is a zinc finger transcription factor that is first expressed at mouse embryonic (E) day 6.25 of development, and mice lacking Blimp1 die at E10.5 due to placental insufficiency (Vincent et al., 2005). Within mouse skin, Blimp1 is highly expressed from E16.5 in developing hair follicles (HFs), sebaceous glands, and interfollicular epidermis (Chang and Calame, 2002). Epidermal deletion of Blimp1 causes multiple differentiation defects (Kretzschmar et al., 2014), including interfollicular epidermal hyperplasia (Chiang et al., 2013), sebaceous gland enlargement (Horsley et al., 2006), and impaired epidermal barrier formation (Magnúsdóttir et al., 2007). One of the underlying molecular mechanisms is Blimp1-mediated repression of c-Myc (Horsley et al., 2006, Kretzschmar et al., 2014, Lin et al., 1997).

In the dermis, Blimp1 is first expressed at E14.5 in the condensates of fibroblasts that will form the HF dermal papilla (DP) (Lesko et al., 2013, Robertson et al., 2007). Blimp1+ cells give rise to the DP, dermal sheath, arrector pili muscles, and the papillary fibroblasts that are required for HF neogenesis during wound healing (Driskell et al., 2013, Lesko et al., 2013, Robertson et al., 2007). Furthermore, when Blimp1 is deleted in Sox2+ cells, whiskers do not develop (Robertson et al., 2007).

Given the importance of Blimp1 in regulating differentiation in a wide range of cell types, we have investigated the effects of dermal-specific Blimp1 deletion on skin homeostasis and wound healing. Our findings demonstrate that Blimp1 is induced by transforming growth factor-β (TGFβ) and controls dermal Wnt/β-catenin signaling, identifying Blimp1 as a core transcriptional regulator of DP activity during HF growth initiation.

## Results

### Dynamic expression of Blimp1 in the DP

**Figure 1:**
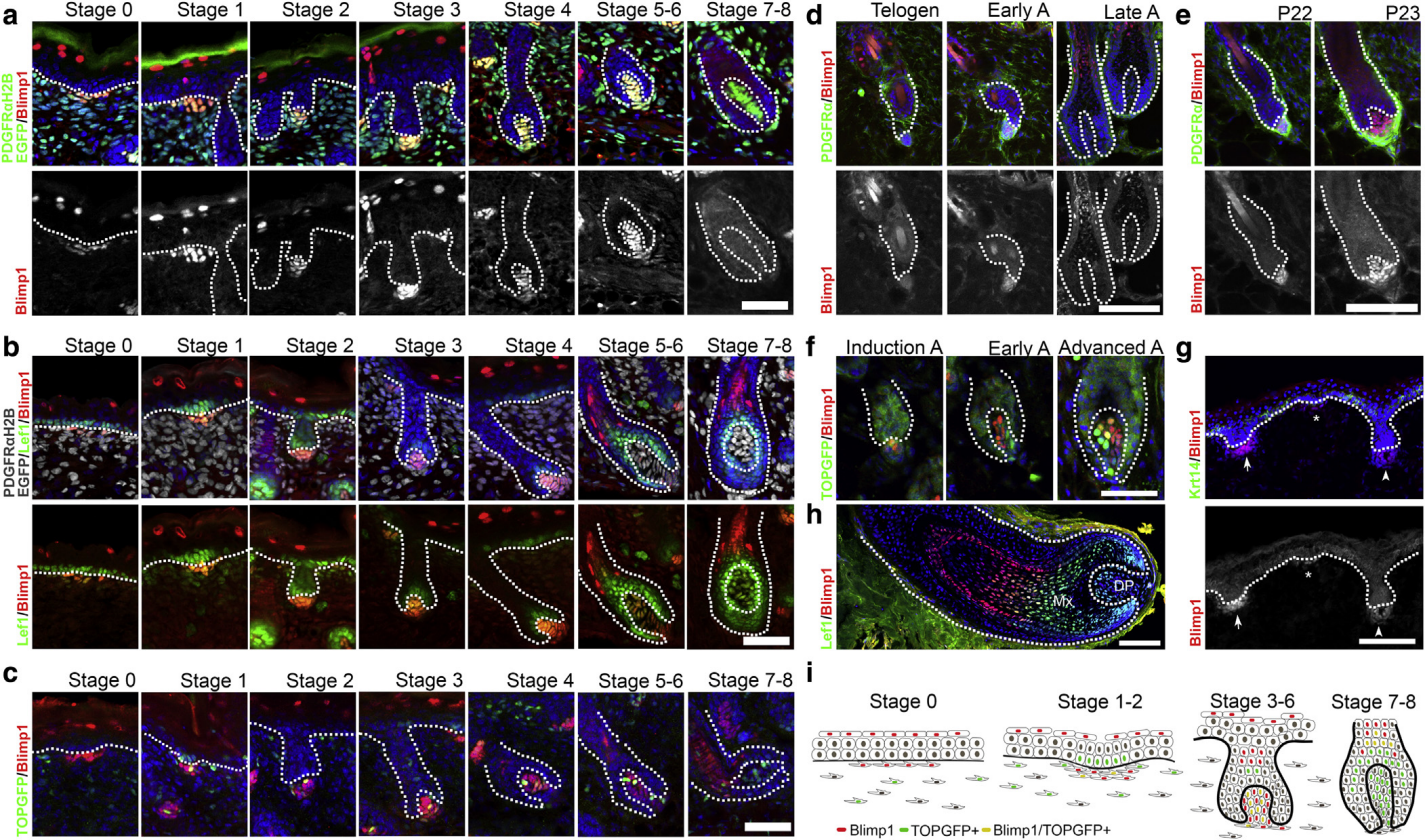
**Blimp1 is dynamically expressed in DP during HF morphogenesis and postnatal anagen induction.** (**a**–**c**) Blimp1 expression during HF morphogenesis. Skin sections from PDGFRαH2BeGFP mice immunostained for (**a**) GFP and Blimp1 or (**b**) Lef1 and Blimp1. (**c**) TOPGFP back skin sections immunolabeled for GFP and Blimp1. (**d**–**f**) Blimp1 expression during postnatal HC. (**d, e**) Immunostaining of adult back skin for PDGFRα and Blimp1 at the indicated age or HC stage. (**f**) TOPGFP skin sections immunolabeled for GFP and Blimp1 at the indicated HC stage. (**g**) Immunostaining of human embryonic skin (week 13) for Krt14 and Blimp1. Early (asterisk), middle (arrow), and late (arrowhead) placode formation stage. (**h**) Adult human HF was immunostained for Lef1 and Blimp1. (**i**) Schematic summary of Blimp1 expression and TOPGFP activity in dermal fibroblasts and keratinocytes during HF development. Scale bars = 50 μm (**a**–**f**), 100 μm (**g, h**). A, anagen; Blimp1, B-lymphocyte-induced maturation protein 1; DP, dermal papilla; HC, hair cycle; HF, hair follicle; Mx, HF matrix.

We first performed a characterization of Blimp1 expression during HF morphogenesis and the hair cycle (HC). Blimp1 was expressed at the earliest stage (stage 0) of HF development (Paus et al., 1999) in PDGFRα+ fibroblasts underlying the epidermal thickening that precedes dermal condensate formation (Figure 1a). Blimp1 was expressed in all dermal condensates (stage 1), the dermal placodes (stages 2 and 3), and developing (stage 4) and mature DPs (stages 5 and 6), regardless of HF type. Blimp1 expression was progressively lost from the DP at stages 7 and 8. Blimp1 began to be expressed in differentiating HF matrix epithelial cells at stages 5 and 6 (Figure 1a). In dermal condensate fibroblasts, Blimp1 expression correlated with the early placode marker Lef1 (Figure 1b; Kratochwil et al., 1996). However, Lef1 expression persisted in the DP at later HF stages, whereas Blimp1 expression did not (Figure 1b).

To correlate Blimp1 expression with active Wnt/β-catenin signaling, we examined transgenic Wnt reporter HFs (TOPGFP; Ferrer-Vaquer et al., 2010). Blimp1 expression preceded β-catenin signaling activation in DP fibroblasts, because TOPGFP was only observed after stage 1, in agreement with earlier studies (Tsai et al., 2014). At stages 7 and 8, TOPGFP activity remained high in the DP, consistent with Lef1 expression, whereas Blimp1 expression was lost (Figure 1b, 1c).

In adults, Blimp1 was not expressed during HC catagen or telogen stages. However, Blimp1 was expressed in DP cells during anagen induction (P22–P23) (Figure 1d, 1e), remained high during early and advanced anagen, and was lost in late anagen (Figure 1d). Similarly, Blimp1 expression preceded TOPGFP activation in the DP, which remained high at advanced anagen, when Blimp1 expression started to decrease (Figure 1f). In human skin, Blimp1 expression was restricted to placodes and absent in full anagen DP cells (Figure 1g, 1h).

We conclude that Blimp1 is an early placode marker in human and mouse skin, where it precedes Wnt signaling activation. In the DP, Blimp1 is downregulated in late HF anagen and re-expressed during anagen induction (Figure 1i).

### Dermis-specific Blimp1 deletion delays HF morphogenesis and anagen onset

**Figure 2:**
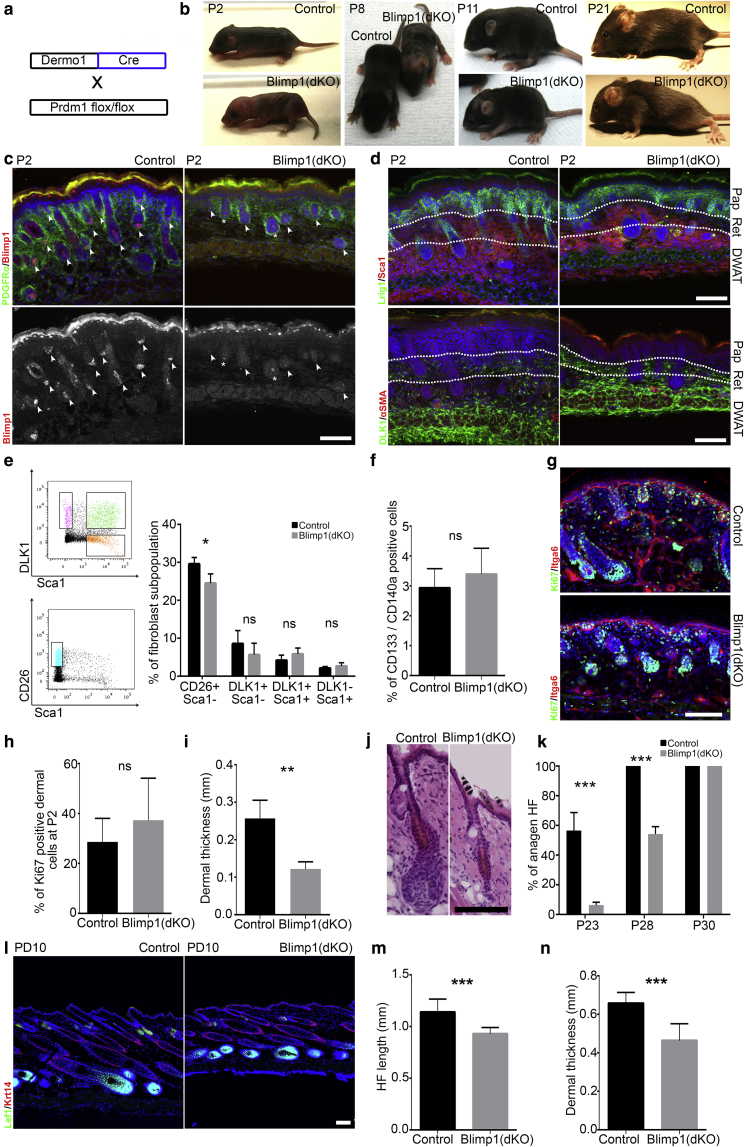
**Specific deletion of Blimp1 in the dermis leads to HF growth retardation and decrease in the papillary fibroblast subpopulation.** (**a**) Breeding strategy. (**b**) Mice at the indicated postnatal ages. (**c**) Immunofluorescence staining for PDGFRα and Blimp1 of P2 skin. DP (arrowhead); single Blimp1+ DP cells (asterisk). (**d**) P2 skin immunostaining for Lrig1 and Sca1 (upper panel), DLK1 and αSMA (lower panel). The white dashed line indicates boundaries of papillary (Pap), reticular (Ret) dermis, and dermal white adipose tissue (DWAT). (**e**) Fibroblast subpopulation FACS analysis and quantification. (**f**) FACS quantification of DP cells as percent of total fibroblasts. (**g**) Immunostaining for Ki67 and Itga6 of P2 skin and (**h**) quantification. (**i**) Dermal thickness quantification at P1. (**j**) H&E-stained skin sections at P23. (**k**) Percentage of HFs in anagen at P23, P28, and P30. (**l–n**) Analysis of depilation induced anagen induction. (**l**) Immunostaining of skin 10 days post depilation (PD10) for Lef1 and Krt-14. Quantification of (**m**) HF length and (**n**) dermis thickness at PD10. Data shown are means ± standard deviation. ∗*P* < 0.05, ∗∗*P* < 0.005, ∗∗∗*P* < 0.0005. Scale bars = 100 μm. Blimp1, B-lymphocyte-induced maturation protein 1; DP, dermal papilla; H&E, hematoxylin and eosin; HF, hair follicle; ns, not significant.

Next, we generated a dermis-specific conditional Blimp1 knockout (Blimp1(dKO)), by crossing Prdm1flox/flox mice with Dermo1Cre transgenics (Supplementary Table S1 online, Figure 2a, 2b). Successful dermis-specific Blimp1 deletion was confirmed by antibody labeling, with only scattered Blimp1+ dermal cells detected in Blimp1(dKO) skin (Figure 2c). At P2, Blimp1(dKO) mice were distinguished from littermate controls by the pale coloration of the back skin and forehead. By P21, the hair coat had developed but was sparser than in controls and whisker development was delayed (Figure 2b, Supplementary Figure S1a online).

All fibroblast subpopulations were present in the P2 dermis and the arrector pili muscle differentiated normally (Figure 2d). There was a small, but statistically significant, reduction in papillary fibroblasts (CD26+,Sca1−). However, there was no change in other fibroblast populations, including DP cells (Figure 2d–f, Supplementary Figure S1b).

Total dermal cell density and proliferation were not significantly affected by Blimp1 deletion (Figure 2g, 2h, Supplementary Figure S1c). However, the P2 dermis was thinner in Blimp1(dKO) mice (Figure 2i), which probably reflects the delay in hair coat development (Figure 2b).

Dermal Blimp1 deletion did not alter HF density and spacing after HF morphogenesis or postnatal HCs (Supplementary Figure S2a, S2b online). Blimp1(dKO) HFs were shorter at P8, but catagen induction was not delayed at P16, indicating that the hair growth phase was shortened on Blimp1 deletion (Supplementary Figure S2c, S2d). The telogen to anagen transition was delayed as Blimp1(dKO) HFs failed to enter anagen at P23 (Figure 2j, 2k). By P30, Blimp1(dKO) HFs were all in anagen; they also transitioned through catagen and telogen normally, pointing to a shortened HF growth phase. Both control and Blimp1(dKO) HFs entered the asynchronous HC phase after P80 (Supplementary Figure S2c). The anagen induction defect was also observed in adult Blimp1(dKO) after depilation as HFs were shorter and the dermis was thinner than in controls (Figure 2l–n, Supplementary Figure S2e).

In summary, although Blimp1 is not required for the development of the papillary lineages, including the DP, it does influence the number of papillary fibroblasts. Besides, in the absence of Blimp1, both HF morphogenesis and anagen are delayed, resulting in a shorter HF growth phase.

### Dermal Blimp1 ablation alters HF maturation and type

**Figure 3:**
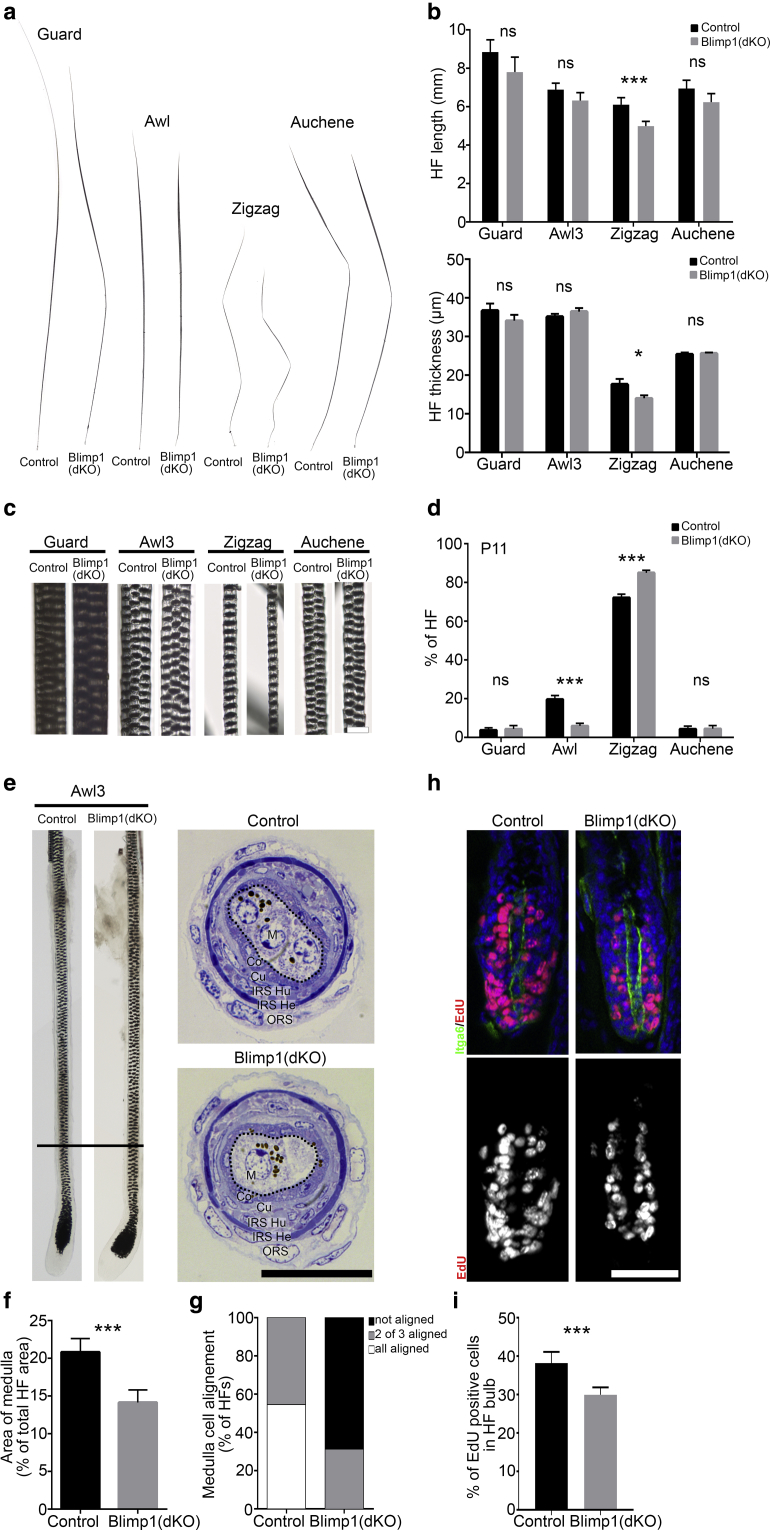
**Dermal Blimp1 ablation impairs the HF maturation process leading to changes in HF size, thickness, type, and medulla cell organization.** (**a**) Bright field images of HF types at P60 and (**b**) quantification of HF length (upper panel) and thickness (lower panel). (**c**) HF type bright field close-up at P60. (**d**) HF type quantification at P11. (**e**) Bright field image of microdissected awl3 HF (left panel) and HF transversal semithin section. The black bar indicates the region of the HF cross section shown in the right panel. (**f, g**) Quantification of medulla cell (**f**) area and (**g**) alignment in awl3 HF cross sections. (**h**) EdU labeling of P11 back skin sections immunostained for Itga6 and (**i**) quantification. Data shown are means ± standard deviation. ∗*P* < 0.05, ∗∗∗*P* < 0.0005. Scale bars = 20 μm (**c**), 25 μm (**e**), 50 μm (**h**)**.** Blimp1, B-lymphocyte-induced maturation protein 1; Co, cortex; Cu, cuticle; EdU, 5-ethynyl-2’-deoxyuridine; He, Henle layer; HF, hair follicle; Hu, Huxley layer; IRS, inner root sheet; M, medulla; ns, not significant; ORS, outer root sheath.

The mouse coat consists of four different HF types that arise in three consecutive waves during development (Chi et al., 2013a, Schlake, 2007, Tsai et al., 2014). All HF types were present in Blimp1(dKO) skin, but zigzag HFs were thinner and smaller (Figure 3a–c, Supplementary Figure S3a online). Furthermore, the number of awl and auchene HFs was decreased, whereas zigzag HFs were significantly increased (Figure 3d). Although awl3 HF size was not altered, Blimp1 deletion resulted in aberrant medulla cell organization: the medulla area was significantly reduced and cells failed to align in triplicates (Figure 3e–g). In contrast, medulla cell organization in guard HFs was not affected (Supplementary Figure S3b, S3c).

To examine the effect of dermal Blimp1 deletion on epidermal cells that are juxtaposed to the DP (Legué and Nicolas, 2005), we labeled hair bulb cells for P-cadherin and measured 5-ethynyl-2’-deoxyuridine (EdU) incorporation in anagen (Figure 3h, 3i, Supplementary Figure S3d). Although HF matrix cell organization showed no obvious defects, the number of EdU+ cells was significantly reduced in Blimp1(dKO) HFs.

We conclude that dermal Blimp1 ablation impairs HF matrix cell proliferation in all HF types. We suggest that the decreased HF matrix cell proliferation could account for the awl3 medulla cell disorganization and the changes in zigzag number and size on dermal Blimp1 deletion, two defects associated with perturbations in DP cell number or signaling (Chi et al., 2013a, Enshell-Seijffers et al., 2010).

### Dermal Blimp1 deletion impairs skin regeneration

**Figure 4:**
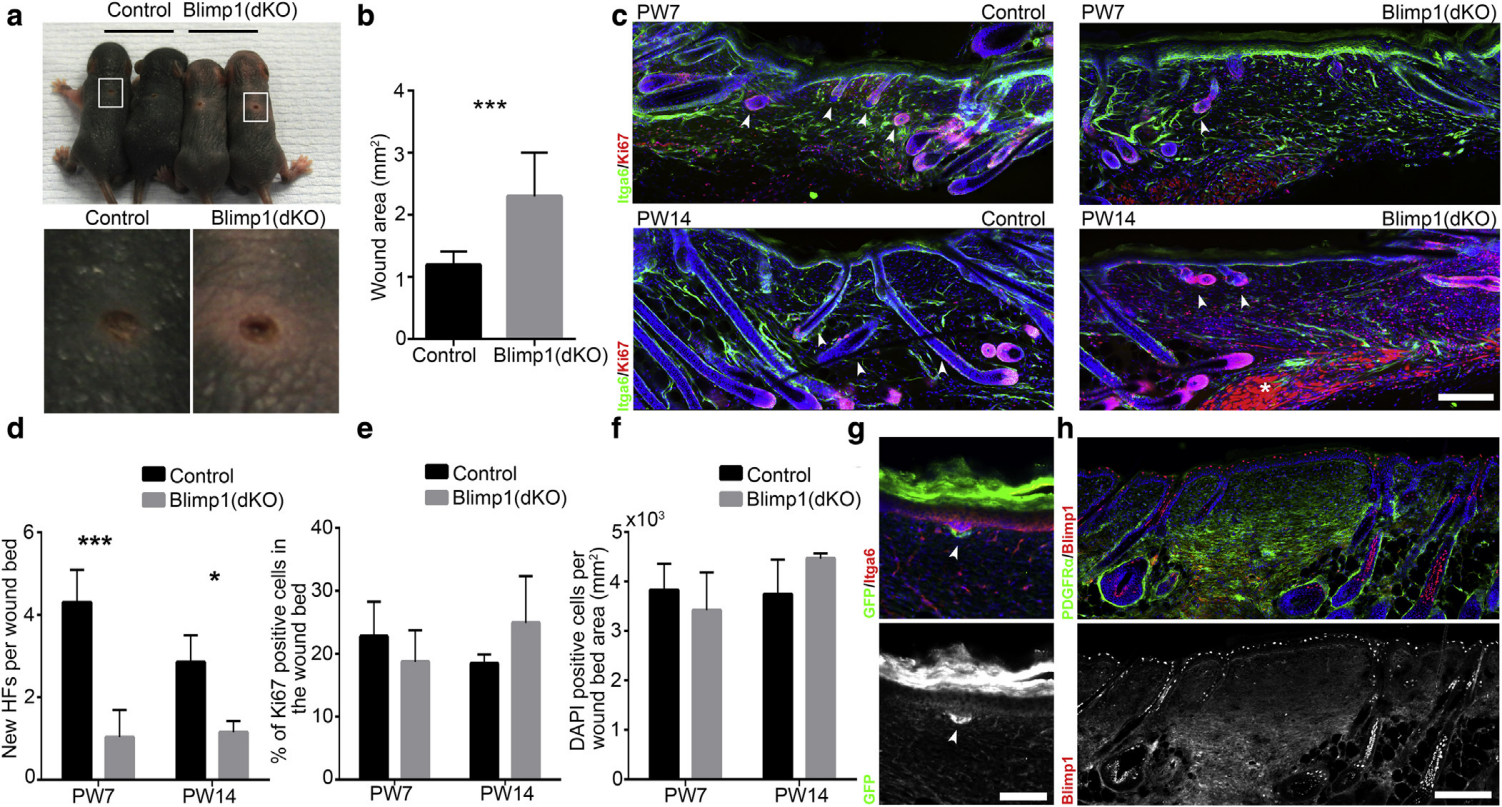
**Effect of dermal Blimp1 deletion on skin regeneration in neonatal wounds.** (**a**) Picture of P2 wounded mice at PW7 (upper panel). The lower panel shows a close-up of boxed wound areas in the upper panel. (**b**) PW7 wound area quantification. (**c**) Immunostaining of PW7 and PW14 wound beds for Itga6 and Ki67. Arrowheads indicate new HFs and the asterisk indicates unspecific Ki67 staining in the lower wound bed. (**d–f**) Quantification of (**d**) new HFs per wound bed, (**e**) Ki67+ dermal cells in the wound bed area, and (**f**) dermal cells in the wound bed area at PW7 and PW14. (**g**) PW7 neonatal wound immunostained for Itga6 and GFP in Blimp1GFP reporter mice. The arrowhead indicates high GFP expression around new forming HFs. (**h**) Immunostaining for PDGFRα and Blimp1 10 days after wounding P21 old mice. Data shown are means ± standard deviation. ∗*P* < 0.05, ∗∗∗*P* < 0.0005. Scale bars = 50 μm (**g**), 100 μm (**h**)**.** Blimp1, B-lymphocyte-induced maturation protein 1; HF, hair follicle; PW, postwounding.

We next analyzed the ability of Blimp1-depleted dermis to repair full thickness wounds and to regenerate HFs. At postwounding day 7, Blimp1(dKO) wounds were larger than controls (Figure 4a, 4b). Although the control wound beds showed new HFs at postwounding day 7, HF formation was significantly reduced in Blimp1-depleted wounds and at postwounding day 14 only a few immature HFs were observed (Figure 4c, 4d). The HF formation defect did not correlate with changes in wound bed cell density or proliferation (Figure 4e, 4f).

To confirm that Blimp1 is normally expressed in newly forming HFs, we examined wounds in Blimp1GFP reporter mice. At postwounding day 7, there was strong GFP expression in the developing HF placodes (Figure 4g). In adult wounds, which do not regenerate HFs (Rognoni et al., 2016), Blimp1 was not expressed in the wound bed dermis (Figure 4h). We conclude that although Blimp1 is not required for HF formation during development, Blimp1 is important for HF regeneration in neonatal wounds.

### Blimp1 is associated with core DP signaling pathways, including TGFβ and Wnt/β-catenin

**Figure 5:**
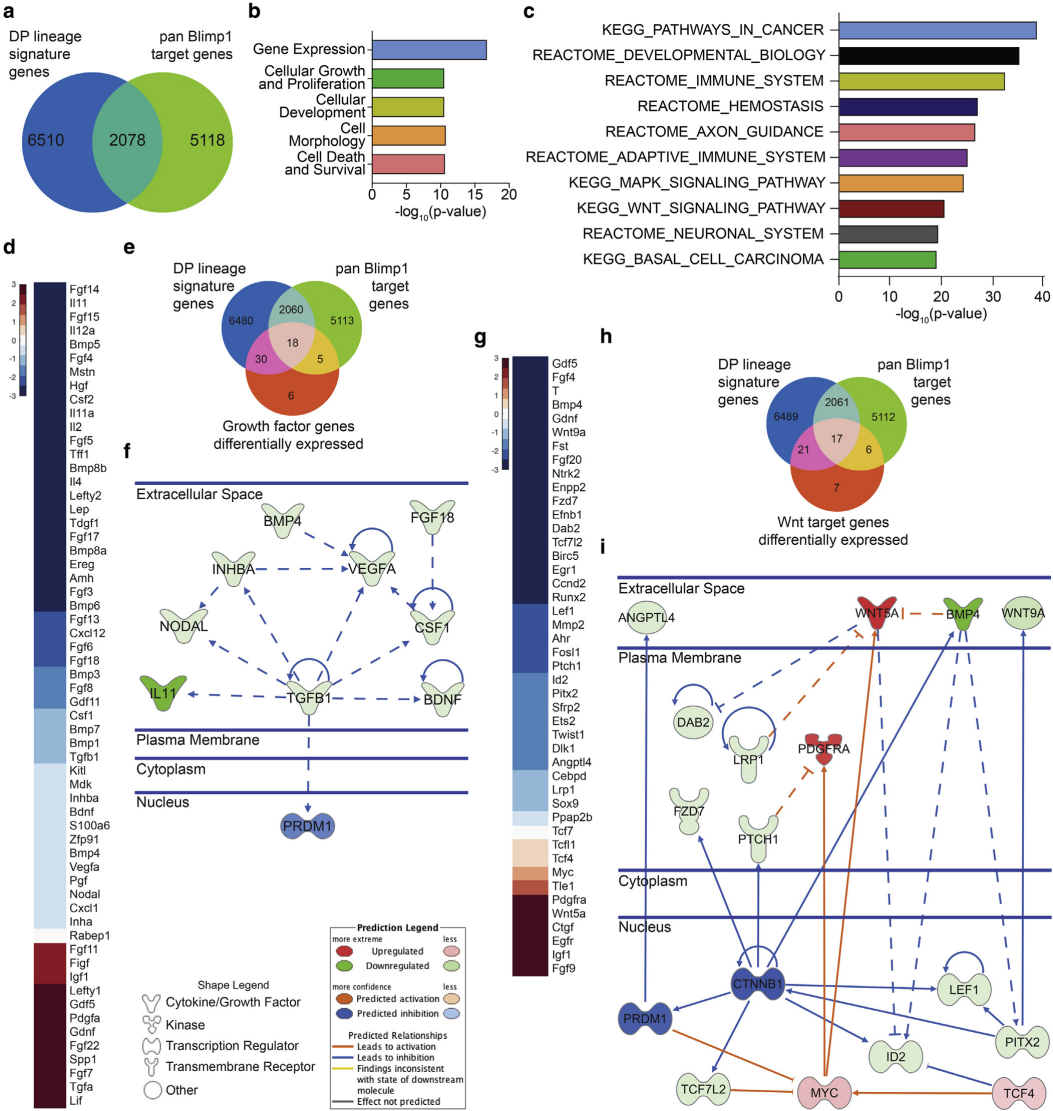
**Dissecting the Blimp1 signaling network in the DP.** (**a**) DP signature and pan-Blimp1 target genes Venn diagram. (**b**) GO term and (**c**) GSEA pathway analysis of intersected Blimp1-regulated DP genes in (**a**). (**d**–**f**) Identifying Blimp1-regulated GFs. (**d**) Gene expression heatmap of the DP cell GF qPCR screen. (**e**) Venn diagram intersecting differentially expressed GFs with DP signature and pan-Blimp1 target genes. (**f**) Ingenuity Pathway Analysis of differentially expressed GFs in the DP. (**g–i**) Identifying Blimp1-regulated Wnt/β-catenin signaling networks. (**g**) Gene expression heatmap of DP cell Wnt signaling qPCR. (**h**) Venn diagram intersecting differentially expressed Wnt signaling genes with DP signature and pan-Blimp1 target genes. (**i**) Ingenuity Pathway Analysis of differentially expressed Blimp1-regulated Wnt signaling genes in the DP. Note that in both identified networks (**f, i**) Blimp1 is predicted to be downregulated. Solid lines indicate direct and dashed lines indirect interactions. Color intensities reflect average gene expression log_2_-fold change. Blimp1, B-lymphocyte-induced maturation protein 1; DP, dermal papilla; GF, growth factor; qPCR, quantitative PCR.

Employing a bioinformatic pipeline, we generated a broad DP lineage signature consisting of 8,588 genes and intersected that with a list of pan-Blimp1 target genes comprising 7,196 entities, compiled from different published screens (Figure 5a). Of 8,588 DP entities, 2,078 are known to be regulated by Blimp1 and are associated with GO terms such as “Cellular Growth and Proliferation” and “Cellular Development” (Figure 5b). Using the GSEA MGSig Database to mine canonical signaling pathways we found that the DP/Blimp1-regulated entities are strongly associated with growth factor (GF) pathways as well as mitogen-activated protein kinase and Wnt signaling (Figure 5c).

To validate the bioinformatics predictions we flow sorted CD140a+,CD133+ DP cells from P2 control and Blimp1(dKO) dorsal skin and performed quantitative PCR (qPCR) analysis using a reverse transcriptase-PCR array to detect expression of a panel of GFs and Wnt/β-catenin target genes (Figure 5d, 5g). Of 60 GFs analyzed, 80% were downregulated in Blimp1(dKO) DP cells, 24 significantly (fold change > 2) (Figure 5d). These included several well-known DP signaling factors of the bone morphogenic protein (BMP)/TGFβ and fibroblast growth factor (FGF) families, including BMP4/6/7/8 and FGF8 and FGF18 (Lee and Tumbar, 2012, Sennett and Rendl, 2012). In the Wnt signaling screen, 34 of 45 genes (76%) were reduced in expression, 22 significantly (fold change > 2) (Figure 5g). These genes were strongly associated with canonical Wnt signaling, such as Lef1, Wnt9a, PTCH1, and FZD7, whereas expression of noncanonical Wnt signaling genes such as Wnt5a was increased (Figure 5g).

We next intersected the differentially expressed genes of both qPCR screens with the pan-Blimp1 and DP signatures and represented each intersection using Ingenuity Pathway Analysis annotated interactions, allowing us to highlight the connections between different nodes and Blimp1 (Figure 5e, 5f, 5h, 5i). We identified 18 DP genes potentially regulated by Blimp1 in the GF screen (Figure 5e) and confidently connected 9 of 14 hits, with TGFβ1 in the center of the network (Figure 5f). In the case of Wnt signaling, we identified 17 DP genes potentially regulated by Blimp1 (Figure 5h) with β-catenin as a connecting entity, which was added to the network and allowed connection of 15 of 16 hits (Figure 5i). As expected, Myc was significantly upregulated in Blimp1 deleted DP cells and was identified as being directly repressed by Blimp1 (Figure 5g, 5i). Intriguingly, the predictions for molecular activity in both resulting Ingenuity Pathway Analysis networks anticipated strong Blimp1 inhibition, supporting the qPCR screen results (Figure 5f, 5i).

**Figure 6:**
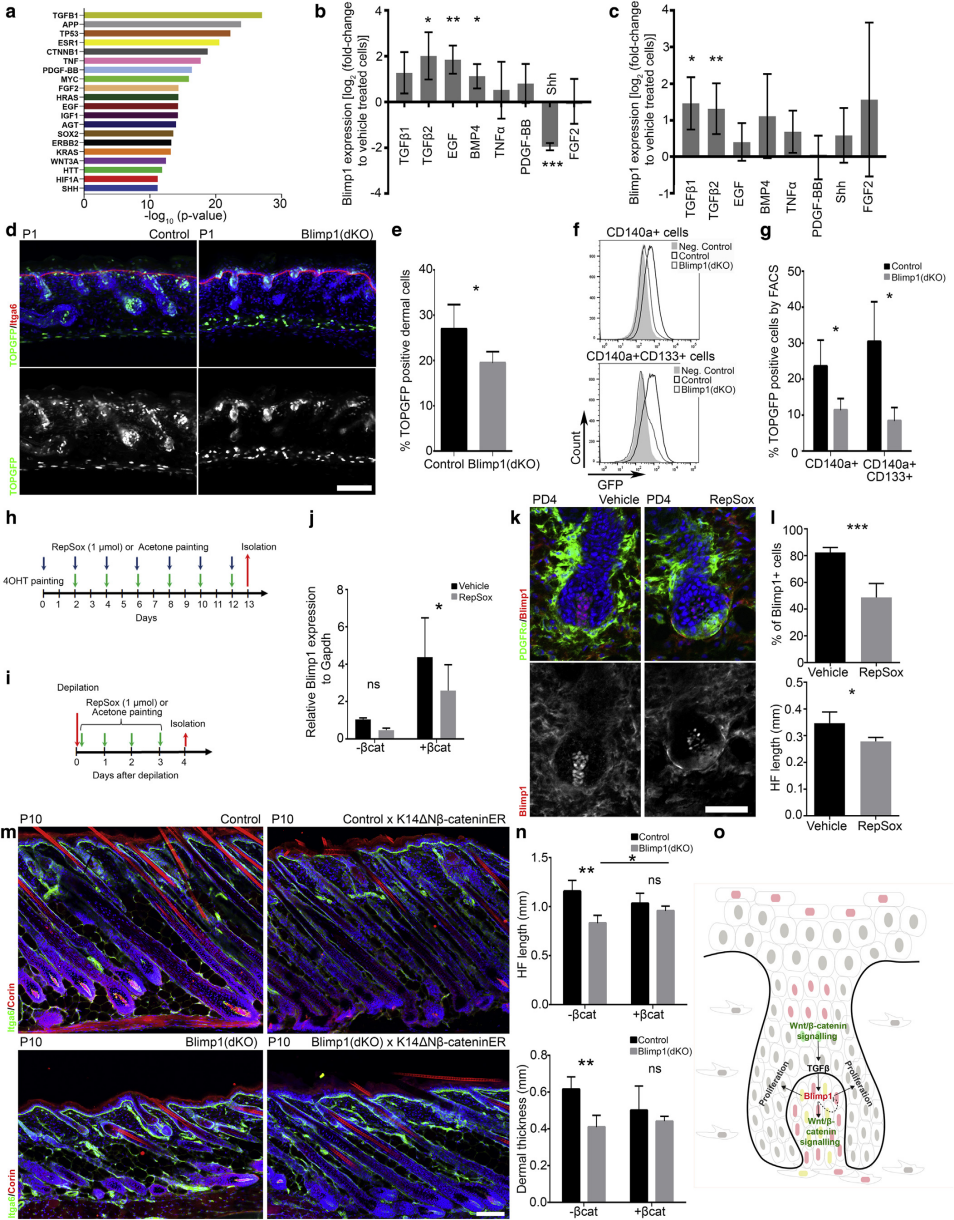
**Blimp1 is induced by TGFβ promoting, Wnt/β-catenin signaling in fibroblasts, and epidermal Wnt/β-catenin activation overcomes HF growth defect after Blimp1 ablation.** (**a**) Top 20 predicted upstream DP Blimp1 regulators by Ingenuity Pathway Analysis. (**b, c**) qPCR for Blimp1 after GF treatment of (**b**) murine DP fibroblasts and (**c**) primary human fibroblasts. (**d**–**g**) Blimp1 deletion impairs dermal Wnt/β-catenin signaling. (**d**) TOPGFP Control and Blimp1(dKO) skin section immunostained for GFP and Itga6; (**e**) TOPGFP+ dermal cells were quantified. (**f**) TOPGFP FACS plots of total (upper panel) and DP fibroblasts (lower panel) and (**g**) quantification. (**h, i**) TGFβ signaling inhibition experimental protocols. (**j**) qPCR for Blimp1 in fibroblasts with and without epidermal β-catenin activation. (**k, l**) TGFβ signaling inhibition during depilation induced HF anagen. (**k**) Skin section immunostained for PDGFRα and Blimp1. (**l**) Quantification of Blimp1+ DP cells (upper panel) and HF length (lower panel) 4 days after depilation. (**m, n**) Epidermal β-catenin activation during dermal maturation. (**m**) Skin sections immunostained for Itga6 and Corin. (**n**) HF length (upper panel) and dermal thickness quantification (lower panel). (**o**) Model of Blimp1 signaling during HF growth induction. We propose that during anagen induction epithelial Wnt/β-catenin signaling induces TGFβ signaling, promoting Blimp1 expression in the DP. Blimp1 activates Wnt/β-catenin signaling in the DP and stimulates proliferation of surrounding hair bulb cells, essential for HF downgrowth. At a later anagen stage, Blimp1 expression in the DP is rapidly downregulated, presumably through self-inhibition (dotted line). Nuclei color code: red, Blimp1+; green, TOPGFP+; yellow, Blimp1+/TOPGFP+; gray, unspecified. Data shown are means ± standard deviation. ∗*P* < 0.05, ∗∗*P* < 0.005, ∗∗∗*P* < 0.0005. Scale bars = 100 μm. βcat, 4OHT-induced epidermal β-catenin; Blimp1, B-lymphocyte-induced maturation protein 1; BW, body weight; DP, dermal papilla; HF, hair follicle; IP, intraperitoneal injection; ns, not significant; PD, postdepilation; qPCR, quantitative PCR; TGFβ, transforming growth factor-β.

Next, by performing a core analysis in Ingenuity Pathway Analysis we identified potential upstream Blimp1 regulators. The most prominent putative regulator was TGFβ1, with TNFα, FGF2, IGF, Wnt effector CTNNB1 (β-catenin), SHH, and EGF also represented (Figure 6a). We tested the effects of candidate GFs on primary CD140a+,CD133+ DP cells (Figure 6b). TGFβ2, EGF, and BMP4 significantly increased Blimp1 expression, whereas TNFα, PDGF-BB, and FGF2 had no effect and SHH significantly reduced Blimp1 expression. TGFβ2 also induced Blimp1 expression in unfractionated cultured mouse fibroblasts (Supplementary Figure S4 online), whereas TGFβ1 and TGFβ2 induced Blimp1 in primary human dermal fibroblasts (Figure 6c). The response to SHH was, however, restricted to DP cells (Figure 6b, 6c, Supplementary Figure S4).

To determine whether Blimp1 loss would reduce dermal Wnt/β-catenin signaling activity, we crossed Blimp1(dKO) with TOPGFP mice and quantified TOPGFP+ cells (Figure 6d–g). As previously shown (Rognoni et al., 2016), Wnt signaling was highly active in papillary, dermal sheath, and DP cells of control mice (Figure 6d). In Blimp1(dKO) skin, the number of TOPGFP+ fibroblasts was significantly decreased, both in unfractionated fibroblasts and DP cells (Figure 6e–g). Consistently, nuclear accumulation of Lef1, TCF1, TCF4, and active β-catenin in Blimp1(dKO) anagen DP cells was reduced and there was a decrease in Sox2 expression in awl, auchene, and guard HFs (Supplementary Figure S5a, S5b online). The reduced Sox2 expression could be an indirect effect of attenuated core DP signaling activity or a direct consequence of loss of Blimp1 transcriptional activity as shown in other tissues (Kurimoto et al. 2008).

Thus, by combining targeted qPCR screens, bioinformatic analysis, and in vivo validation, we identified Blimp1 as both a target and central mediator of DP inductive signaling pathways, including FGF, Wnt/β-catenin, and TGFβ/BMP. The HF defects resulting from Blimp1 deletion are likely to be due, at least in part, to downregulation of β-catenin in the DP because β-catenin signaling regulates HF growth and cycling (Enshell-Seijffers et al., 2010, Kaushal et al., 2015, Tsai et al., 2014).

### Epidermal β-catenin activation induces Blimp1 expression and rescues the effect of dermal Blimp1 deletion

Because epidermal Wnt/β-catenin signaling is essential for HF morphogenesis and anagen induction (Alonso and Fuchs, 2003, Andl et al., 2002, Huelsken et al., 2001, Kobielak et al., 2003, Myung et al., 2013), we examined whether epidermal β-catenin activation would induce dermal Blimp1 expression in K14ΔNβ-cateninER mice (Supplementary Table S1, Supplementary Figure S6a, S6b online). Repeated tamoxifen applications stimulate epidermis-derived GF expression, including TGFβ2, Shh, and FGF2, which induce anagen, ectopic HF formation, and fibroblast proliferation (Lichtenberger et al., 2016, Lo Celso et al., 2004). We found that Blimp1 expression was induced in DPs of anagen and ectopic HFs but not in other fibroblast subpopulations (Supplementary Figure S6c).

To examine whether TGFβ was required for Blimp1 induction, we treated K14ΔNβ-cateninER transgenic mice with the TGFβ inhibitor RepSox, which has previously been shown to inhibit anagen and ectopic HF formation (Lichtenberger et al. 2016). RepSox significantly inhibited Blimp1 expression in ectopic DPs (Figure 6h, 6j). These observations were confirmed by inducing anagen in adult mice by depilation. Treatment with RepSox led to a significant reduction in Blimp1+ DP cells and reduction in HF growth (Figure 6i, 6k, 6l).

To determine whether epidermal β-catenin activation could rescue the HF growth defects induced by Blimp1 loss, we crossed K14ΔNβ-cateninER transgenics with Blimp1(dKO) mice and neonates were treated with tamoxifen (Supplementary Figure S6d, S6e). Epidermal β-catenin overexpression overcame the reduction in HF length and dermal thickness and increased fibroblast proliferation (Figure 6m, 6n, Supplementary Figure S6f, S6g).

To evaluate whether the delay in depilation-induced anagen could also be overridden, mice were depilated and then treated with 4OHT (Figure S6e, S6h). Epidermal β-catenin activation accelerated anagen induction in both control and Blimp1(dKO) mice without affecting HF density (Supplementary Figure S6i–k).

We conclude that epidermal β-catenin activation induces Blimp1 expression in the DP via a TGFβ signaling axis and is able to overcome the dermal Blimp1 deletion defects in HF morphogenesis and the postnatal HC.

## Discussion

We have explored the function of Blimp1 in dermal fibroblasts during postnatal development. Blimp1 is one of the earliest dermal condensate markers and is rapidly downregulated at later stages of HF morphogenesis. Similarly, during the postnatal HC, Blimp1 is highly expressed at anagen initiation and downregulated subsequently. We speculate that dynamic Blimp1 expression in the DP is a consequence of an autoregulatory feedback loop, whereby Blimp1 represses its own expression, as shown in other tissues (Mora-López et al., 2007, Shaffer et al., 2002, Yan et al., 2007; Figure 6o). Blimp1 expression in the hair matrix seems to coincide with hair matrix cell differentiation; however, it is unclear whether this is directly related to Blimp1 loss in the DP. Blimp1 thus differs from other dermal condensate markers such as Lef1 or Sox2, which are expressed throughout morphogenesis (Figure 1b; Driskell et al., 2009).

Our data suggest that transient Blimp1 expression is essential to promote core DP signals such as Wnt/β-catenin, TGFβ/BMP, and FGF during anagen entry and HF formation. Indeed dermal Blimp1 deletion led to a delay in HF morphogenesis and anagen onset as well as a failure of HF neogenesis during wound healing. Zigzag HFs are the last to form at the end of anagen, in the third HF wave, which might explain why their size is most severely affected by the loss of Blimp1. It has previously been reported that Sox2Cre-mediated Blimp1 deletion leads to loss of the sensory vibrissae with no obvious defect in pelage HFs (Robertson et al., 2007), whereas in our studies, vibrissae formation was only delayed (Supplementary Figure S1a). This is most likely because Sox2Cre is active earlier in development (E6.5) than Dermo1Cre (E12.5).

Lineage tracing experiments have shown that Blimp1 expressing cells give rise to cells of the papillary fibroblast lineages, which undergo expansion in response to epidermal β-catenin activation (Driskell et al., 2013, Lichtenberger et al., 2016). However, prolonged epidermal β-catenin activation fails to induce Blimp1 expression outside of the DP (Supplementary Figure S6c). It seems likely that the increase in papillary dermis is due to Blimp1 lineage expansion rather than expansion and migration of Blimp1+ DP cells, because DP cells rarely proliferate and do not exit the DP after wounding (Driskell et al., 2013, Kaushal et al., 2015).

TGFβ1 and TGFβ2 are potent upstream regulators of Blimp1 in dermal fibroblasts (Figure 6a–c) and may potentially act via the c-RAF to AP-1 pathway identified in breast cancer cells (Romagnoli et al., 2012). TGFβ/BMP family members are strongly expressed in the DP and surrounding epithelial cells during placode formation and HF anagen induction (Jamora et al., 2005, Lyons et al., 1990, Oshimori and Fuchs, 2012). Consistent with these observations, treatment with the TGFβ inhibitor RepSox inhibited Blimp1 expression in the DP (Figure 6h–l). Because cultured fibroblasts expressed Blimp1 in response to TGFβ, we suggest that the lack of Blimp1 expression outside the DP is due to an insufficient concentration of TGFβ/BMP in the overlying epidermis. We therefore propose a model where Blimp1 expression in the DP is induced by the TGFβ/BMP signaling axis through epithelial Wnt/β-catenin activation during the telogen to anagen transition (Figure 6o).

Ingenuity Pathway Analysis indicated that β-catenin is downregulated on Blimp1 ablation. In line with this, dermal Blimp1 deletion led to a reduction in Wnt/β-catenin signaling in the DP and papillary dermis (Figures 5g–i and 6d–g). This could reflect the lower number of papillary fibroblasts in Blimp1 mutants because papillary fibroblasts at P2 are characterized by an active Wnt signaling signature (Driskell et al., 2013, Rognoni et al., 2016). The failure of wounds to regenerate HFs in the absence of Blimp1 might be the combination of two defects: first, the reduction in papillary fibroblasts and secondly inefficient epidermal Wnt/β-catenin signaling induction in regenerating placodes and HFs surrounding the wounds.

Blimp1 deletion not only affected HF growth but also HF type and size, as observed after DP β-catenin ablation (Enshell-Seijffers et al., 2010, Tsai et al., 2014). These findings are consistent with our data indicating that Blimp1 positively regulates Wnt/β-catenin signaling. Because epidermal β-catenin stabilization rescues the hair growth defects resulting from Blimp1 deletion, we propose that Blimp1 is a mediator of reciprocal Wnt signaling involving the epidermis and dermis (Millar, 2002).

In summary, our results shed light on the previously unrecognized role of Blimp1 in DP signaling initiation and define its critical involvement in epidermal-mesenchymal communication during HF initiation and regeneration.

## Materials and Methods

### Mice

All experimental procedures were carried out under the terms of a UK Home Office project license. All mice (Supplementary Table S1) were maintained on a C57BL6/CBA background and male and female mice were used in experiments. K14ΔNβ-cateninER mice were injected with 10 μl tamoxifen (50 μg/g body weight, dissolved in corn oil) (Sigma-Aldrich/Merck, Darmstadt, Germany) intraperitoneally at P0, followed by three topical applications of 4OHT (100 μg in acetone; Sigma-Aldrich). For depilation experiments, four topical applications of 4OHT (100 μg in acetone) were made. To assess proliferation P11 mice were injected intraperitoneally with 500 μg EdU in phosphate buffered saline (Invitrogen/Thermo Fisher Scientific, Waltham, MA) 2 hours before isolation.

Neonatal wound healing was performed as described before (Rognoni et al., 2016). Skin depilation was performed as described (Sequeira et al., 2014) with adult (P56) telogen mice. For TGFβ inhibition, skin was treated with RepSox (1 μmol in acetone; Tocris, Minneapolis, MN) daily before isolation.

### Histology, microdissection, and microscopy

Human tissues were obtained with appropriate ethical approval from the UK Human Developmental Biology Resource. Adult surgical waste skin was obtained from the King’s Health Partners Cancer Biobank (HTA Licence No: 12121, REC-No: 12-EE-0493). Tissue samples were embedded in OCT and 12-μm cryosections were processed following a standard protocol (Supplementary Table S2 online). EdU incorporation was detected with the Click-iT EdU-Alexa-Fluor555 kit (Thermo Fisher Scientific, Waltham, MA). Wholemounts were processed as described previously (Rognoni et al., 2016). HF microdissection and sectioning were performed as previously (Sequeira et al., 2014, Sequeira and Nicolas, 2012). Imaging and processing were performed as previously (Rognoni et al., 2016). Three skin sections from at least three biological replicates per genotype were quantified.

### FACS

Neonatal fibroblasts were isolated, labeled, and sorted or analyzed as described previously (Driskell et al., 2013, Jensen et al., 2010).

### Fibroblast culture

Mouse DP cells were sorted (EGFP+, CD133+, CD45−, CD31−, CD324−) and cultured in AmnioMaxC-100 with supplements (Gibco) (37 °C, 5%CO_2_). Human fibroblasts were cultured in DMEM+10% fetal bovine serum (Gibco/Thermo Fisher Scientific, Waltham, MA). For GF screening, DP and human fibroblasts were treated with the indicated GFs for 24 hours before mRNA isolation (Supplementary Table S3 online).

### mRNA isolation and qPCR

RNA was purified with the Purelink RNA microkit (Invitrogen) and reverse transcribed with SuperScriptIII (Qiagen, Germantown, MD). qPCRs were performed with RT^2^ Profiler PCR Arrays (Growth-Factor-Array: PAMM-041ZE-1; Wnt-Signaling-Array: PAMM-243ZE-1) using RT^2^-SYBR-Green-qPCR-Mastermix (Qiagen). Analysis was performed by the delta-Ct method, using one-way analysis of variance with Geisser-Greenhouse correction and Holm-Sidak’s multiple comparisons test.

### Bioinformatics

We compiled the pan-Blimp1 target gene signature by pooling published RNA-seq and CHIP-seq data (Doody et al., 2010, Magnúsdóttir et al., 2007, Mould et al., 2015, Shaffer et al., 2002; Supplementary Table S4 online) and generated the DP-specific signature by analyzing the data from Greco et al. (2009), Hair-GEL (Sennett et al., 2015) and our lab (Driskell et al., 2009; Supplementary Table S4). The intersection between pan-Blimp1 target and DP lineage genes was analyzed using the GSEA MGSig Database (Liberzon et al., 2011, Subramanian et al., 2005) and the Ingenuity Pathway Analysis (Supplementary Table S5 online, Figure 6a).

### Quantification and statistical analysis

Statistical analyses were performed with GraphPad-Prism7. Unless stated otherwise, statistical significance was determined by the unpaired *t*-test for biological effects assuming a normal distribution (ns, not significant, *P < 0.05, **P < 0.005, ***P < 0.0005). Icy software spot-detector plugin was used for identification and quantification of nuclei labeled with DAPI, Ki67, EdU, Blimp1, or TOPGFP.

For HF size, type, and thickness quantification, follicles were randomly plucked from female adult telogen and P11 mice. For whisker quantification, rows were microdissected and HF length was measured from DP to skin surface. To quantitate new HFs in wounds, at least eight 60-μm sections per wound were analyzed. Dermal, Ki67+, Blimp1+ DP, and TOPGFP+ cell densities were quantified from at least eight 12-μm skin section or 60-μm horizontal wholemounts per genotype.

## Conflict of Interest

The authors state no conflict of interest.

## Acknowledgments

We thank Nikon Imaging Centre and Biological Services Unit staff for assistance and gratefully acknowledge access to core facilities supported by the National Institute for Health Research comprehensive Biomedical Research Centre award to Guy’s & St Thomas’ National Health Service (NHS) Foundation Trust in partnership with King's College London (KCL) and KCL Hospital NHS Foundation Trust. FMW acknowledges financial support from the Medical Research Council and Wellcome Trust. ER has an European Molecular Biology Organization long-term-fellowship [ALTF594-2014]. We thank Ana Korosec for experimental support and Kai Kretzschmar and Giacomo Donati for their advice.

## Author Contributions

ER and SBT designed, performed, and analyzed experiments and wrote the paper; IS, BML, PV, OC, and AOP performed experiments; and AOP did bioinformatics analysis. RRD contributed to study initiation. FMW contributed to experimental design and interpretation and co-wrote the paper.
