# Supplementary material for: Dermal Blimp1 Acts Downstream of Epidermal TGFβ and Wnt/β-Catenin to Regulate Hair Follicle Formation and Growth
Source: J Invest Dermatol. 2017 Nov;137(11):2270–81. doi: 10.1016/j.jid.2017.06.015 (PMC5646946; doi:10.1016/j.jid.2017.06.015)
Supplement: Supplementary Figures S1–S6 and Supplementary Tables S1–S5 [file mmc1.pdf]

Dermal Blimp1 acts downstream of epidermal TGFβ and Wnt/β-catenin to regulate hair follicle formation and growth

Stephanie B. Telerman[1,3], Emanuel Rognoni[1,3], Inês Sequeira[1], Angela Oliveira Pisco[1], Beate M. Lichtenberger[2], Oliver Culley[1], Priyalakshmi Viswanathan[1], Ryan R. Driskell[1] and Fiona M. Watt[1]*

[1]King's College London Centre for Stem Cells and Regenerative Medicine, 28th Floor, Tower Wing, Guy's Hospital Campus, Great Maze Pond, London SE1 9RT

[2]Medical University of Vienna, Skin & Endothelium Research Division (SERD), Department of Dermatology, Vienna, Austria

[3]Equal contribution

*Corresponding author: fiona.watt@kcl.ac.uk

SUPPLEMENTAL MATERIAL INVENTORY

Figure S1: Whisker growth upon Blimp1 deletion and DP FACS gating strategy

Figure S2: Loss of dermal Blimp1 induces a delay in hair growth during development and postnatal anagen induction

Figure S3: HF type quantification, guard hair analysis and P-Cadherin analysis of the HF bulb

Figure S4: TGFβ signalling induces Blimp1 expression in neonatal and adult dermal fibroblasts

Figure S5: Dermal Blimp1 loss attenuates Wnt/β-catenin signalling in the DP

Figure S6: Epidermal β-catenin overexpression induces Blimp1 expression during anagen and ectopic HF formation and overcomes hair growth defect in Blimp1(dKO) mice after depilation

Table S1: List of mice lines used in this study

Table S2: List of antibodies used in immunohistochemistry (IHC) and Flow cytometry (FC)

Table S3: List of growth factors used in this study

Table S4: Generation of pan-DP signature and panBlimp1 target genes

Table S5: Bioinformatic analysis of the intersection between the pan-DP signature and the pan-Blimp1 target genes

Figure S1

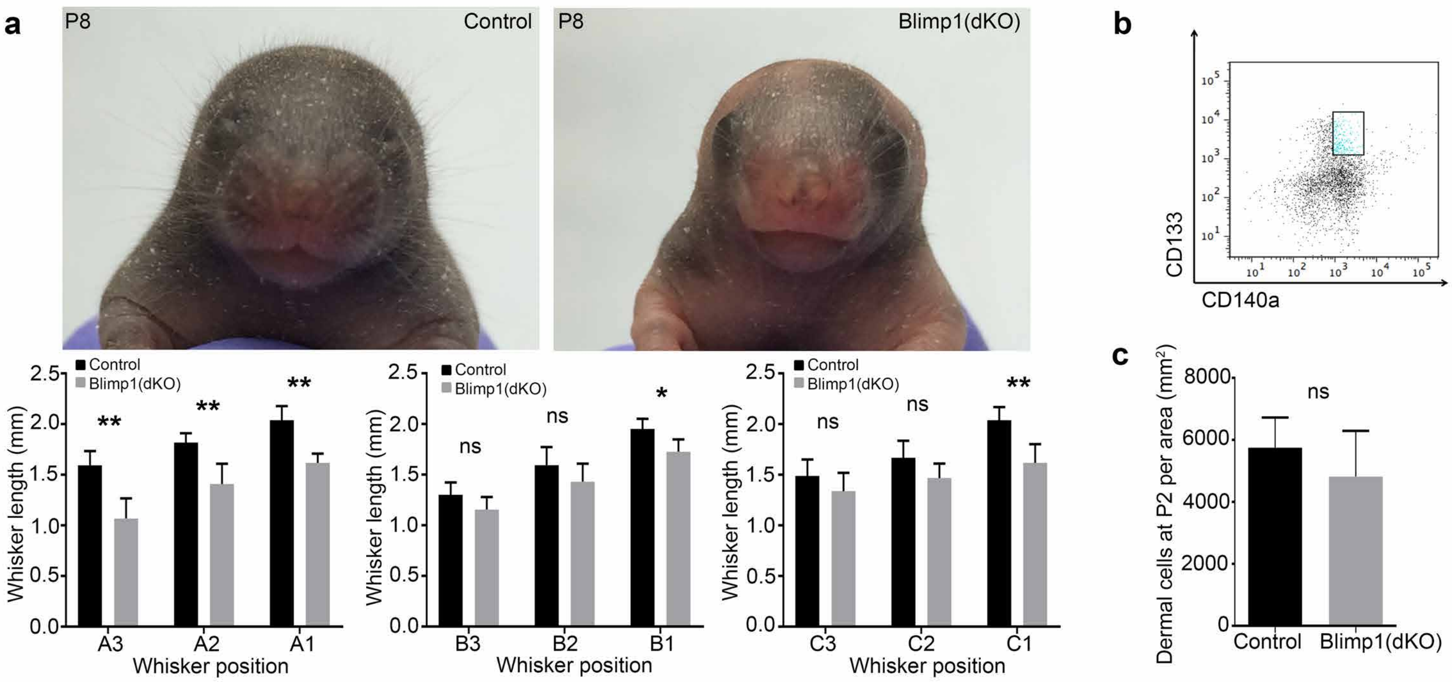

Figure S1: Whisker growth upon Blimp1 deletion and DP FACS gating strategy
(a) Whisker growth analysis. Mouse pictures at indicated postnatal age, showing delayed whisker growth (upper panels) and quantification of whisker length of row A, B and C at P8 (n=3 biological replicates per genotype) (lower panels). **(b)** DP FACS gating strategy isolating CD133+ and CD140a+ cells. **(c)** Quantification of dermal cell density in P2 mice (n=4 sections per mouse; n=5 Control, 4 Blimp1(dKO) biological replicates). Data shown are means ±s.d. ns, not significant, *P<0.05, **P<0.005.

Figure S2

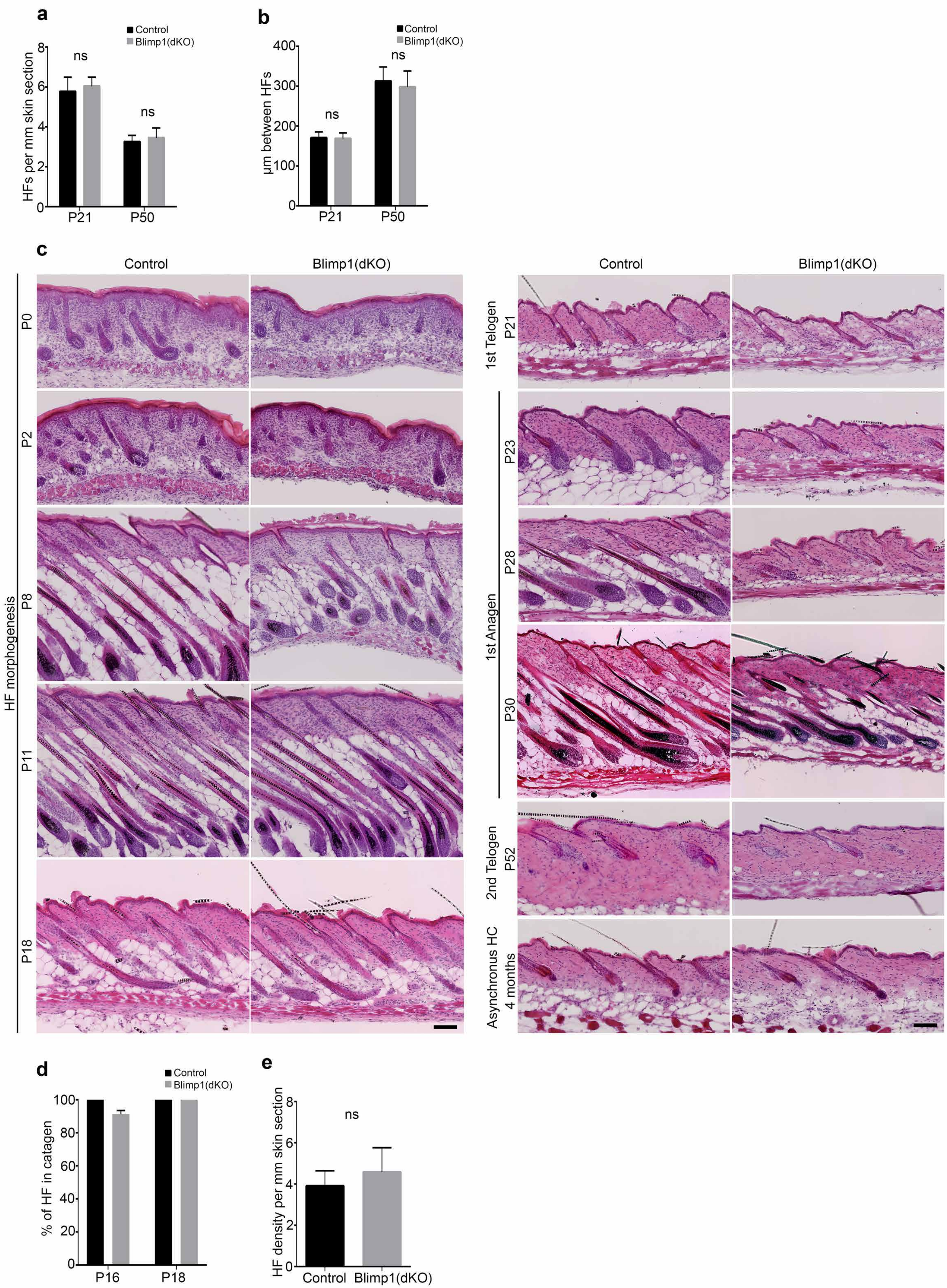
a
Control
Blimp1(dKO)
HFs per mm skin section
ns
ns
P21
P50
b
Control
Blimp1(dKO)
µm between HFs
ns
ns
P21
P50
c
Control
Blimp1(dKO)
HF morphogenesis
P0
P2
P8
P11
P18
1st Telogen
P21
1st Anagen
P23
P28
P30
2nd Telogen
P52
Asynchronus HC
4 months
d
Control
Blimp1(dKO)
% of HF in catagen
P16
P18
e
HF density per mm skin section
ns
Control
Blimp1(dKO)

Figure S2: Loss of dermal Blimp1 induces a delay in hair growth during development and postnatal anagen induction

(a,b) Quantification of HF density **(a)** and HF spacing **(b)** at indicated time points (n=6 skin sections per mouse, n=3 biological replicates per genotype). **(c)** H&E-stained back skin section of indicated time points during hair morphogenesis (left panels) and hair cycle stage (right panels). **(d)** Quantification of HF in catagen (n= 3 Control, 2 Blimp1(dKO) at P16; 2 per genotype at P18). **(e)** Quantification of HF density at PD10 (n=4 whole mount sections per mouse; n=8 Control, 10 Blimp1(dKO) biological replicates). Data shown are means ±s.d. ns, not significant, *P<0.05, **P<0.005. HC, hair cycle. Scale bars, 100µm.

Figure S3

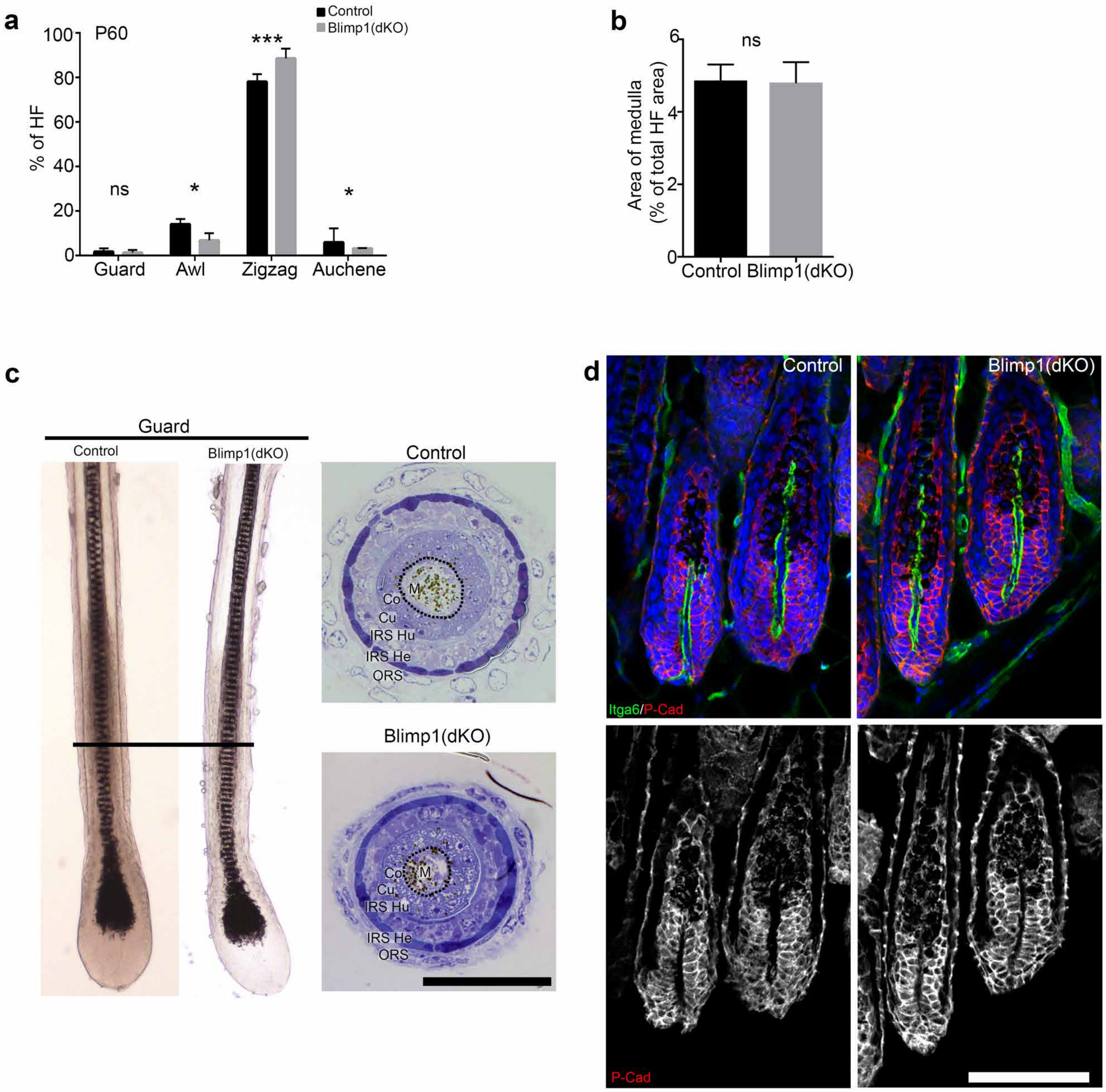

Figure S3: HF type quantification, guard hair analysis and P-Cadherin analysis of the HF bulb
(a) Quantification of HF types in control and Blimp1(dKO) mice at P60 (n=200 HFs per mouse, n=3 biological replicates per genotype). **(b,c)** Guard hair analysis. Quantification of medulla cell area (n= 8 sections per genotype) **(b)**. Bright field image of microdissected guard HF (left panel) and HF transversal semi-thin section **(c)**. Black bar indicates region of HF cross section shown in right panel. **(d)** Immunostaining of control and Blimp1(dKO) back skin at P11 for Itga6 (green) and P-Cadherin (red). Nuclei were labelled with DAPI (blue). Data shown are means ±s.d. ns, not significant, *P<0.05, ***P<0.0005, Scale bars, 50 µm **(c)** ,100µm **(d)**.

Figure S4

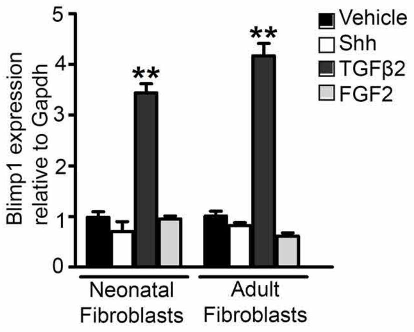

Figure S4: TGFβ signalling induces Blimp1 expression in neonatal and adult dermal fibroblasts
qPCR analysis for Blimp1 expression in neonatal (P3) and adult dermal fibroblasts after 24 hours of treatment with indicated growth factor normalised to Gapdh (n=3 biological replicates). Data shown are means ±s.e.m. **P<0.005.

Figure S5

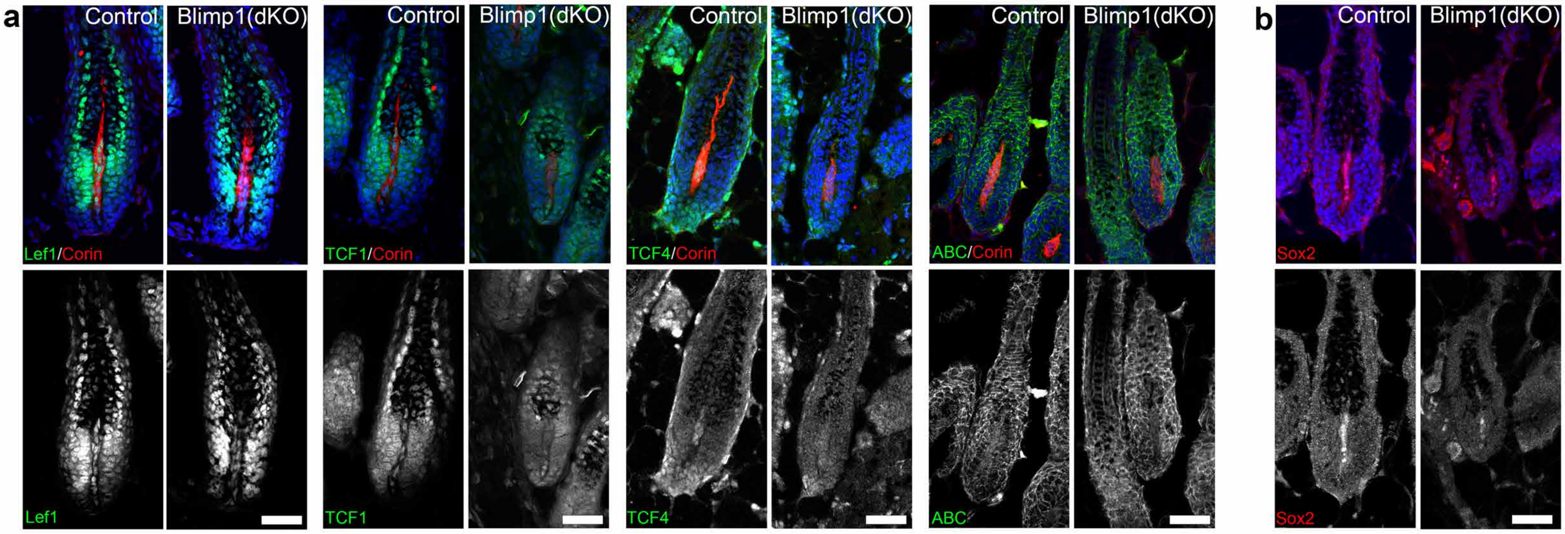

Figure S5: Dermal Blimp1 loss attenuates Wnt/β-catenin signalling in the DP
(a) Immunostaining of Control and Blimp1(dKO) back skin at P11 for Lef1, TCF1, TCF4, active β-catenin (ABC) (green) and Corin (red). **(b)** Immunostaining of Control and Blimp1(dKO) back skin at P11 for Sox2 (red). Nuclei were labelled with DAPI (blue). Scale bars, 100µm.

Figure S6

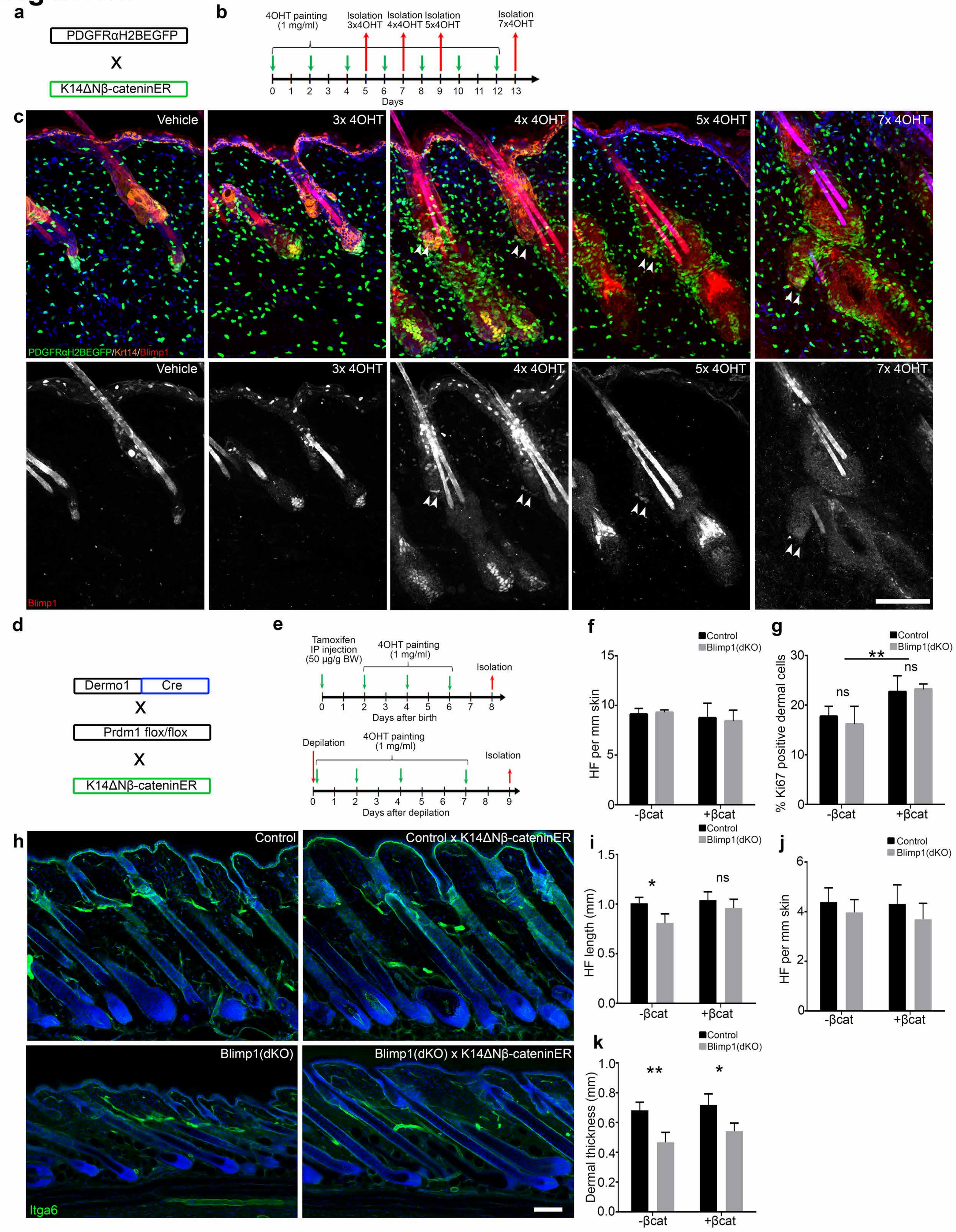

a
PDGFRαH2BEGFP
X
K14ΔNβ-cateninER
b
4OHT painting (1 mg/ml)
Isolation 3x4OHT
Isolation 4x4OHT
Isolation 5x4OHT
Isolation 7x4OHT
0 1 2 3 4 5 6 7 8 9 10 11 12 13
Days
c
Vehicle
3x 4OHT
4x 4OHT
5x 4OHT
7x 4OHT
PDGFRαH2BEGFP/Krt14/Blimp1
Blimp1
d
Dermo1
Cre
X
Prdm1 flox/flox
X
K14ΔNβ-cateninER
e
Tamoxifen IP injection (50 µg/g BW)
4OHT painting (1 mg/ml)
Isolation
0 1 2 3 4 5 6 7 8
Days after birth
Depilation
4OHT painting (1 mg/ml)
Isolation
0 1 2 3 4 5 6 7 8 9
Days after depilation
f
Control
Blimp1(dKO)
HF per mm skin
-βcat
+βcat
g
Control
Blimp1(dKO)
% Ki67 positive dermal cells
ns
**
ns
-βcat
+βcat
h
Control
Control x K14ΔNβ-cateninER
Blimp1(dKO)
Blimp1(dKO) x K14ΔNβ-cateninER
Itga6
i
Control
Blimp1(dKO)
HF length (mm)
*
ns
-βcat
+βcat
j
Control
Blimp1(dKO)
HF per mm skin
-βcat
+βcat
k
Control
Blimp1(dKO)
Dermal thickness (mm)
**
*
-βcat
+βcat

Figure S6: Epidermal β-catenin overexpression induces Blimp1 expression during anagen and ectopic HF formation and overcomes hair growth defect in Blimp1(dKO) mice after depilation

(a,b) Breeding strategy **(a)** and experimental design **(b)** for epidermal β-catenin activation in adult telogen mice. Note that only after four 4OHT treatments ectopic HFs are formed. **(c)** Immunofluorescence staining for Blimp1 (red) and Krt14 (orange) of K14ΔNβ-cateninER x PDGFRαH2BEGFP back skin section (green) after indicated treatment. **(d,e)** Breeding strategy **(d)** and experimental design **(e)** for epidermal β-catenin activation during dermal maturation (upper panel) and after HF depilation (lower panel). **(f,g)** Quantification of HF density **(f)** and Ki67+ dermal cells **(g)** at P10 after epidermal β-catenin activation during dermal maturation (n=3 whole mounts per mouse; n=4 Control, 4 Control x K14ΔNβ-cateninER, 3 Blimp1(dKO), 6 Blimp1(dKO) x K14ΔNβ-cateninER biological replicates). **(h-k)** Epidermal β-catenin overexpression in adult mice after HF depilation during anagen induction. Back skin sections from Control, Control with epidermal β-catenin, Blimp1(dKO) and Blimp1(dKO) with epidermal β-catenin were immunostained for Itga6 (green) 9 days after depilation **(h)**. Quantification of HF length **(i)**, HF density **(j)** and dermal thickness **(k)** 9 days after depilation (n=4 whole mounts per mouse; n=3 Control, 4 Control x K14ΔNβ-cateninER, 3 Blimp1(dKO), 3 Blimp1(dKO) x K14ΔNβ-cateninER biological replicates). Nuclei were labelled with DAPI (blue). Data shown are means ±s.d. ns, not significant, *P<0.05; **P<0.005. βcat, 4OHT induced epidermal β-catenin. Scale bars, 100µm.

Table S1: List of mice lines used in this study

Mice strain	Description	References
TopH2BeGFP	Wnt reporter: H2B-EGFP is expressed under control of multiple TCF sites	Ferrer-Vaquer et al., 2010
Blimp1meGFP	GFP is expressed under the control of Blimp1 transcriptional regulatory region	Ohinata et al., 2005
Prdm1flox/flox	Prdm1 Exons 6-8 are flanked by LoxP sites	Shapiro-Shelef et al., 2003
Dermo1Cre	Cre is expressed under control of Twist2 promoter. Dermo1Cre targets all dermal fibroblasts from E12.5 and is not expressed in the epidermis	Šošić et al., 2003<br>Lichtenberger et al., 2016
PDGFRαH2BeGFP	Dermal fibroblasts reporter: H2B-EGFP is expressed under control of PDGFRα	Hamilton et al., 2003<br>Collins et al., 2011<br>Driskell et al., 2013
K14ΔNβ-cateninER	Truncated β-catenin is expressed under the Keratin 14 promoter and translocate to the nucleus upon tamoxifen injection/ 4OHT topical application	Lo Celso et al., 2004<br>Lichtenberger et al., 2016

Table S2: List of antibodies used in immunohistochemistry (IHC) and Flow cytometry (FC)

**Antigen**	**Conjugate**	**Supplier**	**Reference**	**Applicat-ion**
CD26	PercP cy5.5	eBioscience	45-0261	FC
CD133	APC	eBioscience	17-1331	FC
CD133	PE	eBioscience	12-1331	FC
Ly-6A/E	AF700	eBioscience	56-5981	FC
CD140a	PE	eBioscience	17-1401	FC
Dlk1	PE	MBL Int.	D187-5	FC
CD45	APC	eBioscience	17-0451	FC
CD31	APC	eBioscience	17-0311	FC
CD324	APC	BioLegend	324107	FC
Blimp1	n/a	eBioscience	14-5963-82	IHC
GFP	n/a	abcam	ab13970	IHC
K14	n/a	Cambridge Bioscience	906001	IHC
Ki67	n/a	abcam	ab16667	IHC
Lef1	n/a	Cell Signaling	2230s	IHC
P-Cadherin	n/a	R&D	AF761	IHC
CD49f	n/a	BioLegend	313602	IHC

**Antigen**	**Host species**	**Species reactivity**	**Supplier**	**Reference**	**Applicat-ion**
AF 488	Donkey	Rabbit	ThermoFisher	R37118	IHC
AF 555	Donkey	Rabbit	ThermoFisher	A-31572	IHC
AF 647	Chicken	Rat	ThermoFisher	A-21472	IHC

Table S3: List of growth factors used in this study

Growth Factor	Concentra-tion (ng/ml)	Supplier	Species of recombinant protein	Reference	Used on murine cells	Used on human cells
TGFβ1	10	PeproTech	Human	100-21 B	•	•
TGFβ2	10	PeproTech	Human	100-35B	•	•
BMP4	100	PeproTech	Human	120-05ET	•	•
SHH	1	R&D	Murine	461-SH	•	•
FGF2	20	R&D	Murine	3139-FB	•	•
EGF	100	PeproTech	Murine	315-09	•	•
PDGF-BB	10	PeproTech	Murine	315-18	•	•
TNFα	10	PeproTech	Murine	315-01A	•	
TNFα	10	PeproTech	Human	300-01A		•

Table S4: Generation of pan-DP signature and panBlimp1 target genes

This table consists of 3 tabs

Sennet et al., 2015	Greco et al., 2009									Driskel et al., 2009
	P43	P56	P69	P43 DP Bulge	P56 DP Bulge	P69 DP Bulge	P43 DP HG	P56 DP HG	P69 DP HG	
Pemt	[]	[]	[]	[]	[]	[]	[]	[]	[]	B230215L15Rik
CSN1S1	Psph	Psph	Psph	Psph	Psph	[]	Pcx	Actb	[]	Slc1a1
Sst	Rfk	Rfk	Rfk	Rfk	Rfk	Tfrc	Golm1	Actb	[]	Corin
Igsf1	Ddr1	Ddr1	Ddr1	Ddr1	Ddr1	Pcx	Snrnp27	Gapdh	[]	Prlr
Usp51	Ldhc	Ldhc	Ldhc	Ldhc	Ldhc	Derl1	Pfdn5	Pcx	[]	Clstn2
Lrrc6	Tgfbi	Tgfbi	Tgfbi	Tgfbi	Tgfbi	Golm1	BC031181	Copg1	[]	Gfra1
Cdh12	Sgk1	Sgk1	Sgk1	Sgk1	Sgk1	Tmem129	Sec22b	Atg5	[]	Prlr
Cdh23	Nnt	Nnt	Nnt	Nnt	Nnt	Snapin	Ythdf1	Mrpl27	[]	Prlr
Pcsk2	Perp	Perp	Perp	Perp	Perp	Lamtor5	Itch	Golm1	[]	Slc1a1
Grid1	Emilin1	Emilin1	Emilin1	Emilin1	Emilin1	Pnliprp1	Armcx2	Gps1	[]	Frmd3
Cdh18	Stxbp2	Stxbp2	Stxbp2	Stxbp2	Stxbp2	Armcx2	Vps35	Txndc12	[]	Prlr
Cacng5	Clcn3	Clcn3	Clcn3	Clcn3	Clcn3	Ublcp1	Ublcp1	Psmc5	[]	Gfra1
Gm1653	Spint1	Spint1	Spint1	Spint1	Spint1	Tpbpa	Cx3cl1	Tbl3	[]	Kcnj3
Rragb	Stxbp3a	Stxbp3a	Stxbp3a	Stxbp3a	Stxbp3a	Gsn	Cx3cl1	Ube2v1	[]	Kcnj3
Crtac1	Rnd3	Rnd3	Rnd3	Rnd3	Rnd3	Anxa6	Anxa6	Snapin	[]	Clic5
Gpr165	Fkbp7	Fkbp7	Fkbp7	Fkbp7	Fkbp7	Agtr2	Scd2	Gbf1	[]	D3Bwg0562e
Oprk1	Msh6	Msh6	Msh6	Msh6	Msh6	Mlst8	Scd2	Lamtor5	[]	Tnnt1
Spock3	Ckmt1	Ckmt1	Ckmt1	Ckmt1	Ckmt1	Ldhc	Scd2	Sec22b	[]	Hey2
Sod3	Palmd	Palmd	Palmd	Palmd	Palmd	Rasa3	Agtr2	Itch	[]	Nhedc2
Nlgn1	Plekha2	Plekha2	Plekha2	Plekha2	Plekha2	Dpysl3	Serinc1	Wdr6	[]	Inhba
Kcnd2	Fzd6	Fzd6	Fzd6	Fzd6	Fzd6	Enpp2	Syt4	Armcx2	[]	Lhfpl3
Snap91	Cav2	Cav2	Cav2	Cav2	Cav2	Snrpn///Snurf	Dpysl3	Ganab	[]	Col19a1
Trpm3	Rasa4	Rasa4	Rasa4	Rasa4	Rasa4	Snrpn///Snurf	Cela3b	Ublcp1	[]	Adamts20
Fbxo2	Tuba4a	Tuba4a	Tuba4a	Tuba4a	Tuba4a	Plod3	Sh2d3c	Ublcp1	[]	Dio2
Ppp3r2	Npepps	Npepps	Npepps	Npepps	Npepps	Ccnd3	Enpp2	Ublcp1	[]	Kcnj3
Mc2r	Cd248	Cd248	Cd248	Cd248	Cd248	Ndn	Snrpn///Snurf	Anxa6	[]	Col11a1
Nrxn1	Ibsp	Ibsp	Ibsp	Ibsp	Ibsp	Igf2	Kit	Ppp2r1a	Tfrc	Rps6ka5
Rmst	Apoc1	Apoc1	Apoc1	Apoc1	Apoc1	Fkbp10	Ccnd3	Scd2	Pcx	Col11a1
Gm11437	Arhgap6	Arhgap6	Arhgap6	Arhgap6	Arhgap6	Slc2a4	Impact	Scd2	Pcx	D7Ertd715e
Luzp2	Anxa8	Anxa8	Anxa8	Anxa8	Anxa8	Marcks	Ndn	Scd2	[]	Clstn2
RSPO4	Kdf1	Kdf1	Kdf1	Kdf1	Kdf1	Marcks	Actc1	Agtr2	Psap	Nox4
Hand2	Lamb3	Lamb3	Lamb3	Lamb3	Lamb3	Marcks	Igf2	Serinc1	Golm1	Col19a1
Sostdc1	Galr3///Gc	Galr3///Gc	Galr3///Gc	Galr3///Gcat	Galr3///Gcat	Tubb3	Fmod	Bpgm	Naa60	Plod2
Spp1	Lypd3	Lypd3	Lypd3	Lypd3	Lypd3	Hdlbp	Fkbp10	Bpgm	Pabpn1	Epha7
Gpx3	Plvap	Plvap	Plvap	Plvap	Plvap	Cyp2e1	Prm1	Calu	Aup1	D3Bwg0562e
Cntn1	Rtn3	Rtn3	Rtn3	Rtn3	Rtn3	Cldn11	Slc2a4	Dpysl3	Elp2	Enpp2
Galnt13	Kcnj8	Kcnj8	Kcnj8	Kcnj8	Kcnj8	Mdk	Kap	Cela3b	Armcx2	Runx2
F5	Trp63	Trp63	Trp63	Trp63	Trp63	Tspan3	Marcks	Hsp90b1	Ganab	Stac2
Pde1c	Hspa4l	Hspa4l	Hspa4l	Hspa4l	Hspa4l	Fabp3	Marcks	Sgpl1	Spin1	Pipox
Pappa2	Syk	Syk	Syk	Syk	Syk	Gldc	Marcks	Sgpl1	Gja1	Kif26b
Dtx1	Irf6	Irf6	Irf6	Irf6	Irf6	Scarb1	Hdlbp	Enpp2	Gja1	Ncald
Slc25a31	Fxyd3	Fxyd3	Fxyd3	Fxyd3	Fxyd3	C2	Vcam1	Snrpn///Snurf	Cx3cl1	Eya2
Nrg2	Gpr124	Gpr124	Gpr124	Gpr124	Gpr124	Lrrn1	Sqle	Mgst1	Scd2	1110064A23Rik
Dcc	Lad1	Lad1	Lad1	Lad1	Lad1	Sav1	Hey1	Junb	Scd2	Rps6ka5
Lingo2	Ripk4	Ripk4	Ripk4	Ripk4	Ripk4	Adam15	Cldn11	Plod3	Scd2	Rgnef
Crispld1	Ripk4	Ripk4	Ripk4	Ripk4	Ripk4	Zfand5	Mdk	Lpl	Agtr2	Runx2
Unc5c	Ptpre	Ptpre	Ptpre	Ptpre	Ptpre	Nnt	Copg1	Ccnd3	Mlst8	Runx2
Kcnq3	Dgka	Dgka	Dgka	Dgka	Dgka	Sparcl1	Gldc	Stip1	Syt4	Clic5
Ephx2	Astn1	Astn1	Astn1	Astn1	Astn1	Lox	Scarb1	Tpi1	Syt4	Prdm1
Vstm2a	Etv3	Etv3	Etv3	Etv3	Etv3	Fkbp5	Lrrn1	Ndn	Ldhc	Sp7
Pax1	S100a13	S100a13	S100a13	S100a13	S100a13	Aplp1	Tbc1d15	Fkbp10	Rasa3	Osbpl6
Greb1l	Tnnt2	Tnnt2	Tnnt2	Tnnt2	Tnnt2	Mmp2	Cd34	Slc2a4	Tyrp1	A730054J21Rik
A	Twist1	Twist1	Twist1	Twist1	Twist1	Reg2	Adm	Itm2c	Cct4	Gdf10
Il12a	Fads3	Fads3	Fads3	Fads3	Fads3	Nr2f2	Adam15	Scd1	Spry1	Dio2
Mc5r	Sh3gl2	Sh3gl2	Sh3gl2	Sh3gl2	Sh3gl2	Nr2f2	Rps15	Marcks	Dpysl3	A730082K24Rik
Khdrbs2	Sh3gl2	Sh3gl2	Sh3gl2	Sh3gl2	Sh3gl2	Nr2f2	Rps15	Marcks	Chgb	Acan
Mc3r	Col17a1	Col17a1	Col17a1	Col17a1	Col17a1	Fbln5	Sparcl1	Marcks	Hsp90b1	Kif5a
Fgf14	Pkp3	Pkp3	Pkp3	Pkp3	Pkp3	Rab31	Lox	Isyna1	Enpp2	D9Ertd356e
Snhg11	Foxd1	Foxd1	Foxd1	Foxd1	Foxd1	Prdx4	Fkbp5	Tubb3	Junb	Slc1a1
SCUBE2	Fam110c	Fam110c	Fam110c	Fam110c	Fam110c	Serpinf1	Efr3a	Clcn4-2	Plod3	Cblb
SLC26A6	Rab3d	Rab3d	Rab3d	Rab3d	Rab3d	Plekhb1	Aplp1	Hdlbp	Lpl	Rspo3
Gm5607	Skap2	Skap2	Skap2	Skap2	Skap2	Apba3	Mmp2	Vcam1	Ccnd3	6030442H21Rik
Clmn	Rassf7	Rassf7	Rassf7	Rassf7	Rassf7	Aqp1	Srp54a///Srp54b	Hey1	Gm15483///LOC102	1700021K14Rik
Zcchc18	2610018G	2610018G	2610018G	2610018G03Rik	2610018G03Rik	Gpd1	Nr2f2	Mdk	Tnfrsf19	EG545758 /// Slc10a4
Dok6	Rhbg	Rhbg	Rhbg	Rhbg	Rhbg	Surf4	Nr2f2	Tspan3	Ndn	6430550H21Rik
Shisa9	Akr1c18	Akr1c18	Akr1c18	Akr1c18	Akr1c18	Fstl1	Nr2f2	Abce1	Igf2	Plscr4
Grin3a	Dut	Dut	Dut	Dut	Dut	Rfk	Fbln5	Copg1	Smoc2	Appl2
Gm9839	Fgd6	Fgd6	Fgd6	Fgd6	Fgd6	Olig2	Prdx4	Mcm7	Pebp1	Tcfap2c
Psd2	Hook2	Hook2	Hook2	Hook2	Hook2	Tie1	Serpinf1	Tmem109	Fkbp10	Clic5
GPR98	Bcl11a	Bcl11a	Bcl11a	Bcl11a	Bcl11a	Klhl13	Mesdc2	Cd24a	Slc2a4	Abhd3
Fgf10	Slc2a10	Slc2a10	Slc2a10	Slc2a10	Slc2a10	Pdyn	Car1	Fkbp1a	Slc2a4	Bmp7
Cux2	Matn2	Matn2	Matn2	Matn2	Matn2	Scoc	Kifc3	Snd1	Itm2c	Dio2
Nav3	Arhgef28	Arhgef28	Arhgef28	Arhgef28	Arhgef28	Tnfaip2	Aqp1	Lipf	Scd1	Olfm2
Fam227b	Tpd52	Tpd52	Tpd52	Tpd52	Tpd52	Plod1	Sipa1	Scarb1	Scd1	Cntn3
Lrrtm3	Tuba8	Tuba8	Tuba8	Tuba8	Tuba8	Prdx3	Surf4	C2	Txnip	Prss12
Rasd1	Unc5c	Unc5c	Unc5c	Unc5c	Unc5c	Ebf1	Fstl1	Lrrn1	Cotl1	Ebf1
Scube3	Hoxa2	Hoxa2	Hoxa2	Hoxa2	Hoxa2	Ebf1	Arpc1b///Gm56	Amy2a1///Amy2	Cldn11	9630027E11 /// Grik3
Trim30d	H2-DMb2	H2-DMb2	H2-DMb2	H2-DMb2	H2-DMb2	Mllt11	Olig2	Ndufb11	Mdk	Casq2
Zfp804b	Mfi2	Mfi2	Mfi2	Mfi2	Mfi2	Prelp	Ass1///Gm5424	Tbc1d15	Satb1	Osbpl6
Gca	Chil3	Chil3	Chil3	Chil3	Chil3	Rbmx	Sec13	Cd34	Satb1	Nox4
Sox2	[]	[]	[]	[]	[]	Rbmx	Klhl13	Adam15	Tspan3	Ajap1
Crabp2	Xbp1	Xbp1	Xbp1	Xbp1	Xbp1	Gsta4	Scoc	Lrrc4	Ehd1	D7Ertd715e
Grid2	[]	[]	[]	[]	[]	Apod	Reg3b	Nnt	Fabp3	Rgnef
BC051142	AA517841	AA517841	AA517841	AA517841	AA517841	Rcbtb2	Mmp9	Kti12	Gldc	Tcfap2c
Sfrp1	Gpr87	Gpr87	Gpr87	Gpr87	Gpr87	Rcbtb2	Ebf1	Sparcl1	Scarb1	Gcnt2
Gm5415	Ltb4r1	Ltb4r1	Ltb4r1	Ltb4r1	Ltb4r1	Hmox2	Ebf1	Lox	C2	Nid1
Myo16	Dhx32	Dhx32	Dhx32	Dhx32	Dhx32	Bgn	Mllt11	Fkbp5	Lrrn1	Lrrn1
Rspo2	Shox2	Shox2	Shox2	Shox2	Shox2	Nsmaf	Prelp	Aplp1	Amy2a1///Amy2a2//	Depdc2
Acot11	AK009121	AK009121	AK009121	AK009121	AK009121	Emilin1	Cd81	Mmp2	Hspa5	Rragd
S100b	Bnipl	Bnipl	Bnipl	Bnipl	Bnipl	Gabarapl1	Wwox	Reg2	Sav1	Sorbs1
6430573F11Rik	Sgk3	Sgk3	Sgk3	Sgk3	Sgk3	Tubb6	Rbmx	Hspa4	Adm	6430550H21Rik
Col9a1	Vsnl1	Vsnl1	Vsnl1	Vsnl1	Vsnl1	Pfkfb3	Gsta4	Srsf3	Adam15	AW121567
Mycbpap	Dst	Dst	Dst	Dst	Dst	Cpq	Zscan21	Nr2f2	Zfand5	Cblb
Zfhx4	Ccr2	Ccr2	Ccr2	Ccr2	Ccr2	Tmed6	Apod	Fbln5	Zfand5	Ltbp1
Kcnc4	Ajuba	Ajuba	Ajuba	Ajuba	Ajuba	Pdia4	Rcbtb2	Rab31	Zfand5	Edn3
Mir1188	Cend1	Cend1	Cend1	Cend1	Cend1	Lamb2	Mesdc1	Prdx4	Tpst2	Cbln1 /// LOC100046314
Cd83	Mta3	Mta3	Mta3	Mta3	Mta3	Fscn1	Mesdc1	Serpinf1	Nnt	Lamc3
Tmem255a	Zfhx4	Zfhx4	Zfhx4	Zfhx4	Zfhx4	H1foo	Hmox2	Mesdc2	Kti12	Gfra1
P4ha3	Cyp3a16	Cyp3a16	Cyp3a16	Cyp3a16	Cyp3a16	Sepw1	Bgn	Sec61a1	Cd83	Tnni3
FGF7	Serpinb5	Serpinb5	Serpinb5	Serpinb5	Serpinb5	Slc35b4	Pea15a	Sec61a1	Sparcl1	Col19a1

Mir1897,Sox2ot	Map7	Map7	Map7	Map7	Map7	Stra13	Nsmaf	Nelfcd	Lox	Rspo1
Gm20172	Map7	Map7	Map7	Map7	Map7	Tspan31	Emilin1	Crk	Fkbp5	Bmp7
Spef2	Nxn	Nxn	Nxn	Nxn	Nxn	Slc13a3	Gabarapl1	Aqp1	Errfi1	Kbtbd11
Gareml	Mt1	Mt1	Mt1	Mt1	Mt1	Traf4	Gabarapl1	Sipa1	Aplp1	C030003D03Rik
Islr	Atxn10	Atxn10	Atxn10	Atxn10	Atxn10	Mmp14	Ssb	Surf4	Mmp2	Fgl2
Rspo3	Ecel1	Ecel1	Ecel1	Ecel1	Ecel1	Sparc	Ssb	Fstl1	Olig1	Ncald
GLIS3	Tmem45a	Tmem45a	Tmem45a	Tmem45a	Tmem45a	Glrx	Tubb6	Nsdhl	Nr2f2	Rgs6
Edn3	Fam111a	Fam111a	Fam111a	Fam111a	Fam111a	Glrx	Cpq	Arpc1b///Gm563	Nr2f2	Ss18
Acss3	Moxd1	Moxd1	Moxd1	Moxd1	Moxd1	Sfrp1	Elovl2	Arpc1b///Gm563	Nr2f2	Chsy3
Npas4	Cblc	Cblc	Cblc	Cblc	Cblc	Glis2	Tmem30a	Rfk	Fbln5	2900054C01Rik
Erc2	Krt15	Krt15	Krt15	Krt15	Krt15	Serping1	Arf2	Olig2	Rab31	Clcf1
RAB39B	Serpinb8	Serpinb8	Serpinb8	Serpinb8	Serpinb8	Fam96a	Miox	Psmb7	Prdx4	Fgfr1
Sned1	Scel	Scel	Scel	Scel	Scel	Sall2	Aldh1a1	Sec13	Serpinf1	Tnmd
Hs3st1	Hspb1	Hspb1	Hspb1	Hspb1	Hspb1	Kcne1l	Syap1	Klhl13	Vdac3	Fgl2
Ncam2	Krt17	Krt17	Krt17	Krt17	Krt17	Sema3b	Igdcc4	Coro1a	Plekhb1	D1Ertd622e
Chst8	Coch	Coch	Coch	Coch	Coch	Afp	Igdcc4	Abcb9	Gm2a	Lamc3
Hhip	Rpl39l	Rpl39l	Rpl39l	Rpl39l	Rpl39l	Ambp	Tmem14c	Scoc	Aqp1	Daam2
Steap2	Zic1	Zic1	Zic1	Zic1	Zic1	Aspn	Ddost	Slc26a6	Surf4	Ndph
Plagl1	Atp8a1	Atp8a1	Atp8a1	Atp8a1	Atp8a1	Frzb	Ndufs5	Psmc3	Rpl37a	Cacna1b
Crabp1	Plet1	Plet1	Plet1	Plet1	Plet1	Serpine2	Dctn6	Plod1	Fstl1	Ephb1
Scube1	Mfap4	Mfap4	Mfap4	Mfap4	Mfap4	Plod2	Ulk1	Prdx3	Arpc1b///Gm5637	Ebf1
Ikzf4	Grhl1	Grhl1	Grhl1	Grhl1	Grhl1	Plod2	Cnpy2	Scamp3	Arpc1b///Gm5637	Slco2a1
Wasf3	Cd151	Cd151	Cd151	Cd151	Cd151	Snap91	Lamb2	Mmp9	Olig2	LOC552901
Nap1l3	Krt5	Krt5	Krt5	Krt5	Krt5	Foxc2	Fscn1	Ebf1	Lrrc59	Tpd52l1
Itga9	Col6a3	Col6a3	Col6a3	Col6a3	Col6a3	Gk2	H1foo	Ebf1	Ass1///Gm5424	Pde8b
Ltbp1	Stap2	Stap2	Stap2	Stap2	Stap2	Dpp4	Spop	Ap1m1	Klhl13	Epha7
Gstt1	Atg101	Atg101	Atg101	Atg101	Atg101	Mapk14	Slc35b4	Prelp	Abcb9	D7Ertd715e
Mpped1	Mrps6	Mrps6	Mrps6	Mrps6	Mrps6	Tppp3	Slc35b4	Cd81	Pdyn	Crabp2
D3Bwg0562e	Mfsd6	Mfsd6	Mfsd6	Mfsd6	Mfsd6	Irf8	Zfp239	Cirbp	Scoc	Cacna2d1
Kif1a	Angptl3	Angptl3	Angptl3	Angptl3	Angptl3	Tcf4	Sparc	Gm14277///Snrp	Tnfaip2	9030409G11Rik
Zfp354b	Ranbp10	Ranbp10	Ranbp10	Ranbp10	Ranbp10	Tcf4	Rab34	Men1	Rgs4	A330076H08Rik
Socs3	Serpinb5	Serpinb5	Serpinb5	Serpinb5	Serpinb5	Tcf4	Glrx	Men1	Plod1	Prkg2
Plscr4	Sec14l4	Sec14l4	Sec14l4	Sec14l4	Sec14l4	Col5a1	Slc44a4	Mrpl34	Prdx3	Pde3a
Ano5	Pitx2	Pitx2	Pitx2	Pitx2	Pitx2	Ldb3	Glis2	Mfsd10	Il2rg	Frem1
Cdk20	Bnc1	Bnc1	Bnc1	Bnc1	Bnc1	Prkar1b	Glis2	Dync1i1	Ebf1	Nr1d2
Dram1	Fam83f	Fam83f	Fam83f	Fam83f	Fam83f	Prkar1b	Rcan1	Gsta4	Ebf1	Phex
Sulf1	BC016579	BC016579	BC016579	BC016579	BC016579	Fis1	Thbs3	Zscan21	Nusap1	Clcf1
Tle2	Myc	Myc	Myc	Myc	Myc	Crym	Serping1	Apod	Mllt11	Runx1
Pcdh11x	Baiap2l1	Baiap2l1	Baiap2l1	Baiap2l1	Baiap2l1	Ceacam12	Sall2	Ap3m1	Prelp	Ebf1
Tmem151b	Tmem40	Tmem40	Tmem40	Tmem40	Tmem40	Sdpr	Sema3b	Tmem97	Prelp	Crispld1
Mterfd2	Mbp	Mbp	Mbp	Mbp	Mbp	Sdpr	Aspn	Rcbtb2	Crisp1	Rc3h2
Pcdhb10	Hip1r	Hip1r	Hip1r	Hip1r	Hip1r	Praf2	Frzb	Pycrl	Men1	Notum
Slc26a7	Klc3	Klc3	Klc3	Klc3	Klc3	Pgk2	Eif3a	Abcb10	Rbmx	Runx1
Arsj	Epha3	Epha3	Epha3	Epha3	Epha3	Cryl1	Serpine2	Bgn	Rbmx	Slc16a2
Lrrn3	Tmem176a	Tmem176a	Tmem176a	Tmem176a	Tmem176a	Fkbp7	Apoh	Pafah1b3	Mcam	Slco2a1
Tbc1d4	Lepr	Lepr	Lepr	Lepr	Lepr	Ehbp1l1	Ube3a	Emilin1	Dync1i1	LOC100044139 /// Snrpn /
Bai3	Hoxc13	Hoxc13	Hoxc13	Hoxc13	Hoxc13	Nid1	Plod2	Gabarapl1	Fkbp4///LOC102642	Cacna2d1
P2ry13	Trim25	Trim25	Trim25	Trim25	Trim25	Cyp3a11	Plod2	Gabarapl1	Gsta4	Ddx26b
Phactr3	Odf2	Odf2	Odf2	Odf2	Odf2	Parva	Snap91	Thap11	Apod	LOC677224
Slc9b2	Parp3	Parp3	Parp3	Parp3	Parp3	Cdc37	Coil	Tubb6	Tmem97	2310005E10Rik
Pkdcc	Vwa5a	Vwa5a	Vwa5a	Vwa5a	Vwa5a	Osbpl1a	Foxc2	Pfkfb3	Pcx	LOC633966
Nrk	Mapre2	Mapre2	Mapre2	Mapre2	Mapre2	B230118H07Rik	Dpp4	Mapk8ip3	Rcbtb2	Cacna2d1
9230009I02Rik	Cwh43	Cwh43	Cwh43	Cwh43	Cwh43	Snta1	Tppp3	Cpq	Rcbtb2	Syde2
Cbln3	Hapln1	Hapln1	Hapln1	Hapln1	Hapln1	Tbxas1	Irf8	Tmem30a	Mesdc1	Cacna1h
Ogfrl1	Alcam	Alcam	Alcam	Alcam	Alcam	1110059E24Rik	Crisp2	Cryab	Bgn	Adamts15
FAM198A	Bcl11a	Bcl11a	Bcl11a	Bcl11a	Bcl11a	Pde6d	Tcf4	Ddah2	Pea15a	Plag1
Nwd1	Il17re	Il17re	Il17re	Il17re	Il17re	Pdzrn3	Tcf4	Arf2	Nsmaf	AW049604
Igfbp3	Iffo2	Iffo2	Iffo2	Iffo2	Iffo2	Abhd8	Mkln1	Arf2	Ctsj	Cd200
Chrna7	D930015E(	D930015E(	D930015E06Rik	D930015E06Rik	D930015E06Rik	Parp9	Col5a1	Caprin1	Emilin1	Rasa4
Rab3b	Aftph	Aftph	Aftph	Aftph	Aftph	Cers1///Gdf1	Col5a1	Caprin1	Gabarapl1	Slco2a1
Slitrk5	Rbmx	Rbmx	Rbmx	Rbmx	Rbmx	Aatk	Ldb3	Caprin1	Gabarapl1	2700046A07Rik
Draxin	Aim1	Aim1	Aim1	Aim1	Aim1	Ptov1	Prkar1b	Ddx3x	Tubb6	Rc3h2
Ndp	Cdcp1	Cdcp1	Cdcp1	Cdcp1	Cdcp1	Slc25a10	Prkar1b	Rpn1	Pfkfb3	Thsd1
Cacna1d	Armcx4	Armcx4	Armcx4	Armcx4	Armcx4	Pou2af1	Galnt16	Igdcc4	Bcl2l10	Aof1
Csdc2	Wnk4	Wnk4	Wnk4	Wnk4	Wnk4	Sox2	Fis1	Ddost	Mapk8ip3	Rspo3
Nog	Clip4	Clip4	Clip4	Clip4	Clip4	Stam2	Trappc3	Pdia4	Cpq	Flt1
Nell1	Arhgef16	Arhgef16	Arhgef16	Arhgef16	Arhgef16	Fcgrt	Crym	Lxn	Oat	Runx1
Vegfa	Itgb4	Itgb4	Itgb4	Itgb4	Itgb4	Sirpa	Sdpr	Ulk1	Acta2	Ankrd28
Ncoa3	Pip4k2b	Pip4k2b	Pip4k2b	Pip4k2b	Pip4k2b	Klc1	Sdpr	Cdc42ep4	Arf2	Rragd
Slco4c1	Pdlim5	Pdlim5	Pdlim5	Pdlim5	Pdlim5	C1ra	Praf2	Fscn1	Arf2	Ednrb
DDX26B	Pof1b	Pof1b	Pof1b	Pof1b	Pof1b	Sdc2	Nck2	Fscn1	Aldh1a1	Asph
Prrt1	Lama3	Lama3	Lama3	Lama3	Lama3	Sdc2	Fkbp7	Sepw1	Rpn1	Zfp423
Eya2	Vmn2r88	Vmn2r88	Vmn2r88	Vmn2r88	Vmn2r88	Efemp2	Ehbp1l1	Grcc10	Ccng2	Nr1d2
St6galnac5	[]	[]	[]	[]	[]	Spo11	Nid1	Spop	Ccne1	Igfbp3
Corin	Hbb-b2	Hbb-b2	Hbb-b2	Hbb-b2	Hbb-b2	Fabp4	Tia1	Creld1	Ddost	Rora
Pbx1	Gimap6	Gimap6	Gimap6	Gimap6	Gimap6	Tmem206	Parva	Siae	Pdia4	Stmn2
Slc16a2	Srpx2	Srpx2	Srpx2	Srpx2	Srpx2	Cul7	Cdc37	Slc35b4	Cdc42ep4	Frzb
Frmpd4	Ccdc120	Ccdc120	Ccdc120	Ccdc120	Ccdc120	Krtcap2	B230118H07Rik	Slc35b4	Lamb2	Tanc1
Hoga1	Lphn1	Lphn1	Lphn1	Lphn1	Lphn1	Krtcap2	Snta1	Stra13	Fscn1	Jam2
Ak1	Dmkn	Dmkn	Dmkn	Dmkn	Dmkn	Ptpn1	Med20	Rrp8	Fscn1	Pcdhb16
Dnali1	Otud7b	Otud7b	Otud7b	Otud7b	Otud7b	Gmfb	Tbxas1	Traf4	Spop	Csdc2
Lrrtm1	Pcdh18	Pcdh18	Pcdh18	Pcdh18	Pcdh18	Ceacam10	Mut	Mmp14	Rab32	B3gnt2
ASAP3	[]	[]	[]	[]	[]	Lgals2	1110059E24Rik	Pofut2	Creld1	Enpp2
Vcan	Clip4	Clip4	Clip4	Clip4	Clip4	Pth1r	Pde6d	Socs3	Nfe2l2	Ncald
Gldn	Jam2	Jam2	Jam2	Jam2	Jam2	Emp3	Prmt2	Bad	Slc35b4	1110003F05Rik
Bmp6	Clcn3	Clcn3	Clcn3	Clcn3	Clcn3	Tinagl1	Pdzrn3	Sparc	Slc35b4	Afap1l2
OSR1	Tmem30b	Tmem30b	Tmem30b	Tmem30b	Tmem30b	Man1a	Ncdn	Rab34	Stra13	1110003F05Rik
Alx4	Acvr1b	Acvr1b	Acvr1b	Acvr1b	Acvr1b	Arl2bp	Fgd1	Rab34	Traf4	Pcdh17
Stx19	Ldb3	Ldb3	Ldb3	Ldb3	Ldb3	Ahcy	Tspan6	Glrx	Socs3	Erc2
Bai1	Hs1bp3	Hs1bp3	Hs1bp3	Hs1bp3	Hs1bp3	Meis2	Rgs10	Sfrp1	Man2b2	Tktl1
Il1rapl1	Zxdc	Zxdc	Zxdc	Zxdc	Zxdc	Angptl4	Cers1///Gdf1	Glis2	Zfp239	Stmn2
Trim60,Trim61	Zfp277	Zfp277	Zfp277	Zfp277	Zfp277	Pmp22	Tsn	Rcan1	Sparc	Fgfr1
Bdh2	Sh3bgrl2	Sh3bgrl2	Sh3bgrl2	Sh3bgrl2	Sh3bgrl2	Igtp	Dlg3	Thbs3	Rab34	Pgm2l1
Alpl	Cnot6l	Cnot6l	Cnot6l	Cnot6l	Cnot6l	Fam69b	Trpv2	Serping1	Rab34	C030044C12Rik
Zc3h12b	Otud4	Otud4	Otud4	Otud4	Otud4	Pdgfrb	Pcsk1n	Tmem223	Glrx	Nkd2
Prr16	Elmo3	Elmo3	Elmo3	Elmo3	Elmo3	Ntsr2	Sox2	Slc1a5	Glrx	1500015O10Rik
Col11a1	Plekha7	Plekha7	Plekha7	Plekha7	Plekha7	Slc25a14	Fcgrt	Fam96a	Hdgfrp2	Nkd2
1810011O10Rik	Vwf	Vwf	Vwf	Vwf	Vwf	Krt19	Ttc1	Sall2	Glis2	Per3
4931406H21Rik	Dsp	Dsp	Dsp	Dsp	Dsp	Zxdc	Pacsin3	Rfpl4	Acss1	Ankrd28
Ldb2	Dsp	Dsp	Dsp	Dsp	Dsp	Cdk2ap2	Klc1	Aspn	4632428N05Rik	Ednrb
Zic1	Nfix	Nfix	Nfix	Nfix	Nfix	Psip1	C1ra	Akt1	Thbs3	Pgm2l1
Ar	Arl4c	Arl4c	Arl4c	Arl4c	Arl4c	Lztfl1	Sdc2	Frzb	Serping1	Hey1

Ppyr1	Tgfbi	Tgfbi	Tgfbi	Tgfbi	Tgfbi	Tmem218	Efemp2	Ndufa9	Slc1a5	Kctd1
Oxnad1	Frmd4b	Frmd4b	Frmd4b	Frmd4b	Frmd4b	Galk1	Spo11	Serpine2	Fam96a	Slc6a6
Dlk1	[]	[]	[]	[]	[]	Hbb-b1///Hbb-b2/	Fabp4	Emc2	Sall2	Tex15
Fam196b	Serpinb5	Serpinb5	Serpinb5	Serpinb5	Serpinb5	Dnajb9	Pfdn1	Bace2	Slc2a5	Rora
Col15a1	Rasgrp2	Rasgrp2	Rasgrp2	Rasgrp2	Rasgrp2	Tmsb10	Trim2	Kng1	Kcne1l	Pdlim3
Rgl1	Sema4a	Sema4a	Sema4a	Sema4a	Sema4a	Ppm1a	Tmem206	Plod2	Ambp	Rnf144b
Col23a1	Spint2	Spint2	Spint2	Spint2	Spint2	Ehd3	Ube2g2	Plod2	Rfpl4	Mycbpap
SLC5A7	Sema4a	Sema4a	Sema4a	Sema4a	Sema4a	Ccl6	Sac3d1	Snap91	Aspn	Chst11
Arsi	Tgfbi	Tgfbi	Tgfbi	Tgfbi	Tgfbi	Cd14	Cul7	Foxc2	Frzb	Wnt5a
MGST1	Ywhaz	Ywhaz	Ywhaz	Ywhaz	Ywhaz	Eng	C1qtnf4	D17Wsu104e	Serpine2	9330159F19Rik
Thbd	Grb7	Grb7	Grb7	Grb7	Grb7	Fam114a1	Krtcap2	Dpp4	Bace2	Wnt5a
Kcne1l	Cdh1	Cdh1	Cdh1	Cdh1	Cdh1	Nkd1	Krtcap2	Tppp3	Ptpru	Ypel2
SCARA5	Polr1d	Polr1d	Polr1d	Polr1d	Polr1d	Itpr1	C1qb	Irf8	Plod2	Nr1d1
Aard	Tax1bp1	Tax1bp1	Tax1bp1	Tax1bp1	Tax1bp1	Mmp23	Adck3	Tcf4	Plod2	Mpped2
Tbxa2r	Irf8	Irf8	Irf8	Irf8	Irf8	Mmp23	Fabp9	Tcf4	Snap91	Neto2
Tmem2	Olfml3	Olfml3	Olfml3	Olfml3	Olfml3	Lrg1	Lgals2	Tcf4	Coil	Cd1d2
Ankrd6	Crtap	Crtap	Crtap	Crtap	Crtap	Speg	Sec61b	Csnk2b	Gk2	Lama2
Arhgef28	Aldh1a3	Aldh1a3	Aldh1a3	Aldh1a3	Aldh1a3	Brinp1	Zfp346	Gys1	Dpp4	2900078E11Rik /// Cadm2
Zkscan16	Dusp19	Dusp19	Dusp19	Dusp19	Dusp19	Pvrl3	Pth1r	Col5a1	Serpini1	Zfp618
Ptprz1	F2rl1	F2rl1	F2rl1	F2rl1	F2rl1	Btrc	Emp3	Col5a1	Pmf1	Pcdh17
Grb14	Stard10	Stard10	Stard10	Stard10	Stard10	Ercc1	Man1a	Cfdp1	Tppp3	Ednrb
En1	Myo1b	Myo1b	Myo1b	Myo1b	Myo1b	Arl6	Arl2bp	LOC102642017//	Irf8	Phactr3
Cebpd	Prim1	Prim1	Prim1	Prim1	Prim1	Rasa4	Map3k12	LOC102642017//	Tcf4	Daam2
Snai2	Lrba	Lrba	Lrba	Lrba	Lrba	Epb4.2	Gabra6	Prkar1b	Tcf4	Mpped2
Sdk2	Col11a1	Col11a1	Col11a1	Col11a1	Col11a1	Bloc1s6	Plekho1	Fis1	Ube2s	Ube2m
4921511H03Rik	Epb4.1l4a	Epb4.1l4a	Epb4.1l4a	Epb4.1l4a	Epb4.1l4a	Sorbs1	Meis2	Stk25	Rcl1	Ypel2
Myrf	Comt	Comt	Comt	Comt	Comt	Mfap2	Angptl4	Crym	Col5a1	Neto2
Ddit4l	Cxcl16	Cxcl16	Cxcl16	Cxcl16	Cxcl16	Zfp61	Pmp22	Sdpr	LOC102642017///Ua	Slc5a7
RTL1	Gjb5	Gjb5	Gjb5	Gjb5	Gjb5	Slc7a7	Lpar1	Sdpr	Htra1	Pcdhb3
Cyr61	Cdkl2	Cdkl2	Cdkl2	Cdkl2	Cdkl2	Gas6	Nfxl1	Praf2	Ldb3	Epha4
B230217C12Rik	Spp1	Spp1	Spp1	Spp1	Spp1	Nap1l5	Fam69b	Praf2	Prkar1b	Zfp354a
Fbxo27	Nudt1	Nudt1	Nudt1	Nudt1	Nudt1	Srgn	Pdgfrb	Acvr1	Prkar1b	Frzb
Anks6	C1qc	C1qc	C1qc	C1qc	C1qc	Gng3	Ntsr2	Idh3g	Mical1	LOC100044094 /// LOC100
Cer1	Dscam	Dscam	Dscam	Dscam	Dscam	Fmo1	Krt19	Ppm1g	Galnt16	Tgfbr1
Fam65c	Plek2	Plek2	Plek2	Plek2	Plek2	Cd248	Zxdc	Cryl1	Crym	Arrdc3
Prtg	Lpin3	Lpin3	Lpin3	Lpin3	Lpin3	Dnajc12	Wfdc18	Nck2	Sdpr	Cdh2 /// LOC100044363
Sfmbt2	Slc2a10	Slc2a10	Slc2a10	Slc2a10	Slc2a10	Ifitm2	Cdk2ap2	Trpm7	Sdpr	LOC677224 /// Ubash3b
Glis1	Sh3yl1	Sh3yl1	Sh3yl1	Sh3yl1	Sh3yl1	Apobec3	Dusp10	Fkbp7	Praf2	OTTMUSG00000006457
Arhgap20	AA522020	AA522020	AA522020	AA522020	AA522020	Shd	Psip1	Ehbp1l1	Praf2	Shox2
WIF1	Chmp4c	Chmp4c	Chmp4c	Chmp4c	Chmp4c	Cp	Galk1	Fam198b	Pgk2	Dnali1
Csrnp3	Ubqln2	Ubqln2	Ubqln2	Ubqln2	Ubqln2	Cp	Hbb-b1///Hbb-b	Nid1	Acvr1	Actg2
Paqr8	Pak1	Pak1	Pak1	Pak1	Pak1	Cp	Dnajb9	Parva	Nck2	Pde8b
Tspan4	Dst	Dst	Dst	Dst	Dst	Serpinf2	Wwc2	Cdc37	Fkbp7	Pgm2l1
Uba7	Sytl1	Sytl1	Sytl1	Sytl1	Sytl1	Tgm2	Eif2s3y	Cdc37	Nid1	Gprasp2
Zic4	Ptger3	Ptger3	Ptger3	Ptger3	Ptger3	Fbxo6	Rbm6	Dgcr14	Tia1	Alx4
Sh2b2	Ak4	Ak4	Ak4	Ak4	Ak4	Ddit3	Magel2	B230118H07Rik	Tia1	Gpc2
Serpinf1	Pdgfb	Pdgfb	Pdgfb	Pdgfb	Pdgfb	Fbxo32	Tmsb10	Snta1	Tia1	Kifc2
Ebf1	Klra15	Klra15	Klra15	Klra15	Klra15	Plek	Ppm1a	Gstm5	Parva	Col23a1
9230115E21Rik	Etv1///Gm	Etv1///Gm	Etv1///Gm	Etv1///Gm5454	Etv1///Gm5454	Pcbp3	Cldn2	Pde6d	Dgcr14	Sorbs1
Serpine2	Cdpf1	Cdpf1	Cdpf1	Cdpf1	Cdpf1	Itgb5	Mmp11	Tmem132a	B230118H07Rik	Gcnt2
Zmiz1	Prr5	Prr5	Prr5	Prr5	Prr5	Itgb5	Ehd3	Pdzrn3	Snta1	A830059I20Rik
Gdf10	Baiap2l1	Baiap2l1	Baiap2l1	Baiap2l1	Baiap2l1	Slc35a1	Ehd3	Cisd1	Tbxas1	Jag1
Hgf	Tmem40	Tmem40	Tmem40	Tmem40	Tmem40	Slc35a1	Gpr180	Ncdn	Mid1ip1	Col23a1
Rnf144a	Fam20b	Fam20b	Fam20b	Fam20b	Fam20b	Rps6ka2	Cel	Ncdn	1110059E24Rik	6720418B01Rik
F630111L10Rik	[]	[]	[]	[]	[]	Sart3	Cd14	Abhd8	Tmem132a	Hs3st3b1
Bdnf	Npnt	Npnt	Npnt	Npnt	Npnt	Fap	Eng	Fgd1	Ncdn	Col8a2
JAM2	Il17re	Il17re	Il17re	Il17re	Il17re	Plac1	Fam114a1	Cers1///Gdf1	Abhd8	Dmxl2
Cldn9	Nbea	Nbea	Nbea	Nbea	Nbea	Fabp1	Itpr1	Dnajc15	Adam8	Foxd1
Sp7	Sptbn2	Sptbn2	Sptbn2	Sptbn2	Sptbn2	Fyn	Slc17a1	Fam89b///Sssca	Rgs10	A630005I04Rik
Chodl	Fam83h	Fam83h	Fam83h	Fam83h	Fam83h	Eif4ebp1	Mmp23	Nphp1	C1d	Ajap1
Eda	Arap2	Arap2	Arap2	Arap2	Arap2	Eif4ebp1	Mmp23	Rbm14-rbm4///R	Tnni2	A330021E22Rik
Tbkbp1	Rai2	Rai2	Rai2	Rai2	Rai2	Mpg	Lynx1	Rbm12	Utf1	Zfp618
Phactr2	Gm21451/	Gm21451/	Gm21451/	Gm21451///Loxl2	Gm21451///Loxl2	Mpg	Lrg1	Trpv2	Cers1///Gdf1	D030011O10Rik
Lrfn5	Hoxb7///H	Hoxb7///H	Hoxb7///H	Hoxb7///Hoxb8	Hoxb7///Hoxb8	Cd28	Akr7a5	Aatk	Aatk	1700019D03Rik
Plag1	Fam83g	Fam83g	Fam83g	Fam83g	Fam83g	Per2	Chrd	Gabarap	Ppa1	6720401G13Rik
Dpysl4	Serpinf1	Serpinf1	Serpinf1	Serpinf1	Serpinf1	Camk1	Speg	Acaa1a///Acaa1b	Ptov1	Krt8
Gm10664	Esrp1	Esrp1	Esrp1	Esrp1	Esrp1	Camk1	Cfb	Mrpl23	Slc39a7	Zcchc18
Crhr2	Mical2	Mical2	Mical2	Mical2	Mical2	Ier5	Brinp1	Slc39a7	Slc25a10	Dab1
Gabre	Pomc	Pomc	Pomc	Pomc	Pomc	Ier5	Pvrl3	Slc25a10	Pou2af1	Tgfbr1
Lamc3	Rnf114	Rnf114	Rnf114	Rnf114	Rnf114	Slc12a2	Btrc	Kcnab2	Pcsk1n	Col23a1
Ptprd	Tgfbi	Tgfbi	Tgfbi	Tgfbi	Tgfbi	Rsph1	Slc23a2	Rcc1	Sox2	Akap10
Prss12	Limd2	Limd2	Limd2	Limd2	Limd2	Sspn	Slc23a2	Eefsec	Gtse1	4930413G21Rik
Smarcd3	[]	[]	[]	[]	[]	Cdkn1c	Arl6	Pcsk1n	Fcgrt	Krt8
Rasa4	Serpinh1	Serpinh1	Serpinh1	Serpinh1	Serpinh1	Pvalb	Rasa4	Sox2	Sirpa	A630033E08Rik
4930447C04Rik	[]	[]	[]	[]	[]	Elk3	Sytl4	Stam2	Mfng	Kctd1
Spock2	C79468	C79468	C79468	C79468	C79468	Grb14	Epb4.2	Fcgrt	Klc1	Scn3a
Nr3c2	Ptpn6	Ptpn6	Ptpn6	Ptpn6	Ptpn6	Opn3	Peg3	Pacsin3	Crat	Dtx1
Pcolce	Wdr81	Wdr81	Wdr81	Wdr81	Wdr81	Kcna5	Sorbs1	Rrs1	C1ra	6720401G13Rik
PAPPA	Hspa12b	Hspa12b	Hspa12b	Hspa12b	Hspa12b	Prss2	Mfap2	Klc1	Sdc2	Nkd2
LEPR	Eif3e	Eif3e	Eif3e	Eif3e	Eif3e	Soat1	Rhcg	Crat	Sdc2	Jag1
Gprasp2	Sep-11	Sep-11	Sep-11	Sep-11	Sep-11	Arhgap6	Ndufa2	C1ra	Efemp2	LOC631639 /// Lonrf1
Dpyd	Mc5r	Mc5r	Mc5r	Mc5r	Mc5r	Naglu	Il16	Sdc2	Spo11	Jak2
Tox3						Hba-a1///Hba-a2	Slc7a7	Sdc2	Fabp4	Ntrk2
Maob						Srsf9	Gas6	Efemp2	Cul7	Gnl2
Gem						Pygl	Nap1l5	Fabp4	Lcat	Enpp2
Sep-03						Pkd2	Slc6a3	Ube2g2	Bid	Skil
PCBP3						Lsp1	Srgn	Ube2g2	Rhd	3021401C12Rik
Spock1						Pla1a	Fmo1	Cul7	Pcdh8	Klhdc2
Mum1l1						Sncg	Cdr2	Slc37a4	1700071K01Rik///Ph	Tcfap2c
Bai2						Ccl11	Cd248	Bid	Krtcap2	Wnt5a
4933424G05Rik						Rasgrp2	Dnajc12	Pcdh8	Ptpn1	Fgfr1
Mdk						Mug1	E2f5	1700071K01Rik/	Qk	Ptprk
Igfbp4						Gpx7	Ifitm2	Krtcap2	Lgals2	Rora
Itgbl1						Spon2	Tnnc2	Slc22a6	Rcn1	Spsb1
Golm1						Fam181b	Tex19.1	Sec61b	Pth1r	4930430E16Rik
Naga						Ctsz	Shd	Pth1r	Gtf2h4	Jam2
Mab21l1						Ctsz	Bmi1	Gtf2h4	Cd320	Iqcb1
Efnb3						Ctsz	Cp	Ddt	Emp3	Mum1l1
Bcl3						Nenf	Cp	Emp3	Tinagl1	Rora
Hey2						Gstt2	Serpinf2	Tinagl1	Man1a	Arntl
DIO2						Ints5	Tgm2	Man1a	Man1a	Fbxo32
Dgkh						Zp3r	Calb1	Man1a	Arl2bp	Slc26a7

Gfra1	Dfna5	Plagl2	Arl2bp	Vav3	Phf3
Lrrk2	Prl7a2	Efna2	Map3k12	Ahcy	Ccrl1
Heph	Amn	Plek	Cstf1	Plekho1	Cacna2d1
ENPP2	Pak3	Pcbp3	Naa10	Meis2	Ebf3
Hif3a	Ccl22	Spag6	Ahcy	Angptl4	Elavl1
Dnahc2	Igfbp6	Cyp2j5	Plekho1	Dsn1	0910001A06Rik
Fosb	Gng4	Itgb5	Meis2	Nfxl1	Spsb1
Nol3	Tnmd	Itgb5	Pmp22	Fam69b	Pde3a
Ptplad2	Btd	Slc35a1	Fam69b	Pdgfrb	Hsd17b7
Kif13b	Svs3a///Svs3b	Rps6ka2	Pdgfrb	Zxdc	Slc7a3
Gm11149	Tmem176b	Rps6ka2	Zxdc	Cdk2ap2	Amot
Thsd7a	Sncb	Sart3	Zxdc	Lztfl1	Zscan18
FIGNL2	Iqcf3	Zfp68	Wfdc18	Ube2l6	Runx3
Trim9	Eltd1	Fap	Cdk2ap2	Tmem218	Camk2n1
Thrb	Kera	Plac1	Dusp10	Galk1	D7Bwg0826e
A330021E22Rik	Tfpt	Fyn	Csnk1e	Gipc2	Zic1
Amot	St6galnac4	Ncald	Galk1	Kifap3	Hoxd1
Olfm2	Nmt1	Mpg	Dnaja2	Hbb-b1///Hbb-b2///	8430419K02Rik
Samd14	Plvap	Mpg	Hbb-b1///Hbb-b	Dnajb9	Syn2
Adam11	Crlf2	Gmppa	Ube2k	Kdelr2	Agtr1b
Adrbk2	Adcy4	Selenbp1	Ube2k	Sertad2	Tenc1
Cacna1h	Hes1	Gkap1	Sod2	Cldn2	Rnf144a
Hmgcll1	Hey2	B9d1	Wwc2	Ehd3	Rora
Plscr1	Tcea2	Per2	Nt5c2	Gpr180	Skil
Emilin1	Rtkn2	Camk1	Sertad2	Spata5	Gprc5b
Stat5b	Gspt2	Camk1	Tmsb10	Mmp13	Hip1
Olfml3	Cyp2d10	Apoa5	Capn1	Ccl6	B4galt6 /// LOC675709
Tsc22d3	Myh11	Ier5	Mmp11	Fkbp11	Zfp57
Mir143hg	Ccl5	St6galnac2	Ehd3	Cd14	5830468K08Rik
Dand5	Tgfb1i1	Itih2	Ehd3	Eng	Tmem166
Grik5	Dcx	Rac2	Pzp	Pdk4	Gdnf
Magi2	Kcnj8	Slc12a2	Spata5	Mal	Tbx18
Armcx6	Tfap2c	Limk1	Capzb	Tulp2	Omd
Pcdhb13	Mtmr4	Spa17	Coq5	Cyp4f16	Igfbp3
PIEZO2	Myot	Lefty1	Coq5	Nkd1	LOC671878 /// Sms
Kcnn3	Nr2f1	Cd79b	Fkbp11	Itpr1	Abca1
Gmpr	Mkrn3	Galntl5	Cd14	Mmp23	Cdc14b
Pcyt1b	Tlr4	Aldh1a3	Cdk9	Mmp23	Prkar1b
Fam149a	Tlr4	Sspn	Eng	Lynx1	BC043118
Ssc5d	Zcchc14	Cdkn1c	Fam114a1	H13	Ets1
Cela1	Zcchc14	Ndrg3	Mal	Lrg1	4930420K17Rik
Lrrn2	Hebp1	Ndrg3	Nkd1	Ifi47	Tmod3
Gng8	Atg3	Slc4a10	Itpr1	Mta1	Ypel2
Ksr1	Gstt1	Grb14	Mmp23	Mvd	5830468K08Rik
Usp11	Ramp2	Ptpro	Mmp23	Chrd	Bcar1
Camk2n1	Plekhg2	Opn3	Mapkap1	Speg	LOC100040358 /// Zfp521
Per3	Aif1	Arhgap6	H13	Brinp1	6330403K07Rik
Vcam1	Pfn2	Syt3	Plekha2	Pvrl3	Ntrk2
Appl2	Pfn2	Cyp46a1	Lrg1	Btrc	LOC545261
1810041L15Rik	Omg	Gm9905	Mta1	Slc23a2	Cdc2l6
Myd88	Rbfox2	Hba-a1///Hba-a	Chrd	Arl6	Ccrl1
Pcdhb3	Loxl3	Laptm5	Speg	Rasa4	4832441B07Rik
Soga3	Adamts8	Pqbp1	Cfb	Epb4.2	Flrt2
Frem1	Serpina1b	Akap8l	Brinp1	Emc7	Amy1
Izumo4	Ifit2	Pygl	Pvrl3	Bloc1s6	Tnn
Rgs6	Fxyd5	Pkd2	Btrc	Sorbs1	Spred1
Tceal3	Dpysl4	Topors	Anapc11	Mfap2	AI844685
4833422C13Rik	Ppt2	Lsp1	Slc23a2	Zfp61	Spon1
Cpxm1	Cdhr1	Nr0b1	Slc23a2	Gpc1	Rgs6
Kcnq4	Tnfrsf11b	Azgp1	Arl6	Slc7a7	Hsf2
Igtp	Rlbp1	Als2	Rasa4	Gas6	Amot
Ncoa7	Rnf128	Als2	Ppp1r2	F3	Ppm1a
Hdx	Agtpbp1	Pla1a	Peg3	Nap1l5	Pdgfra
Mir369,Mir410,Mir412,Mirg	Rab4a	Sncg	Mfap2	Slc6a3	Ubash3b
Cblb	Tnfsf12///Tnfsf12T	Zfml	Gpc1	Srgn	Slc15a2
Fgfr1	Insl6	Xpnpep2	Slc7a7	Fmo1	Slc5a3
Mmp17	Cog1	Kcnb1///Pacsin2	Gas6	Cdr2	Zfp462
Park2	Nucb2	Serinc3	Smim11	Gpd2	5330433J24Rik
Gria4	Wbscr27	Tor1b	Nap1l5	Cd248	Nid2
Kctd12b	Rnf112	Aqp8	Slc6a3	Dnajc12	Afap1l2
Shox2	Ftl1///Gm20746///	Smc1a	Srgn	E2f5	Rerg
Cdh11	Gpr124	Gpx7	Rnaseh2c	Tacc3	Maoa
Ssbp2	Apcdd1	Gm20738///Gm2	Cd248	Tgfb3	Letm2
Nphp3	Apcdd1	Ulk2	Arid1a	Ifitm2	Prrx1
Rspo1	Mphosph8	Ulk2	Tgfb3	Rgs5	Jag1
Zfp641	Cd97	Spon2	Ifitm2	Apobec3	Ccdc138
Igdcc4	Tspan32	Ccnc	Cap1	Shd	Sema3g
Slc43a3	Larp6	Fam181b	Cap1	Cp	LOC100044234 /// Nptx2
Arhgef9	Adam19	Ctsz	Rgs5	Cp	C030033M12Rik /// Rsf1
Gria3	Tnfaip6	Ctsz	Apobec3	Cp	Il17rd
Ncam1	Slc16a2	Ctsz	Ibsp	Cp	Snrpn /// Snurf
5430421F17Rik	Slc16a2	Nenf	Shd	Serpinf2	Crispld1
Mill2	Islr	Gstt2	Cp	Tgm2	Grasp
Cacng7	Gng2	1810009A15Rik/	Serpinf2	Calb1	Pitpnm3
Tmem100	Gng2	Apobec2	Tspan7	Fbxo32	Igfbp4
Meis1	Mfap5	Gzma	Plagl2	Plek	Per2
Brsk1	Copz2	Dfna5	Plagl2	Rab33a	1700021K14Rik
Gm15713	Pgf	Serpinc1	Pcbp3	Cyp2j5	Nhedc1
Inhba	Matn1	Timm10b	Ap3m2	Itgb5	6720401G13Rik
Meg3,Mir1906-2,Mir770	Vnn1	Amn	Slc35a1	Itgb5	Jak2
Mdga2	Grem2	Pak3	Rps6ka2	Hells	Prrx2
Aplp1	Fgf13	Nab2	Flot2	Rps6ka2	Galnt7
Snrk	Fgf13	Igfbp6	Sart3	Flot2	Ntrk3
Sh3bp5	Nap1l3	Gng4	Fap	Sart3	Tmtc1
Hs6st3	Socs2	Gng4	Fyn	Fap	Sox11
Lancl3	Grb2	Abhd3	Eif4ebp1	Fyn	C77405
Bcl6	Dpt	Ilf2	Med7	Eif4ebp1	Acta2
Il16	Rgl1	Sst	Ncald	Eif4ebp1	Trim24
Lrp3	Kdelr3	Cpeb1	Anapc1	Mpg	Crim2
Pyroxd2	Wasf1	Fbxo31	Mpg	Mpg	1700065O13Rik
Ccdc39	Stambpl1	Kpna4	Mpg	Nup210	AU022245
Hoxc8	Svs6	Tnmd	Selenbp1	Timeless	Hip1

Zfp575	Tssk3	Tssk1	Ptges2	Timeless	A630033E08Rik
2010001M06Rik	Rtp4	Ppp1r14d	Tusc2	Meox1	Hck
Lysmd2	Cbfa2t3	Ngb	Gkap1	Cd28	4933439C10Rik
MTHFR	Fgfr4	Ngb	B9d1	Cd276	BC031353
NDNF	Col11a1	Tmem176b	Camk1	Per2	Fzd3
Tril	Avpr1a	Cml1	Camk1	Per2	C1qtnf7
Svep1	Avpr1a	Exo1	Calr	Calr	A330068G13Rik
Heatr8	Slfn1	C4bp	Ier5	Tmem37	Nhedc1
Spry2	Kcnj1	Abcc5	Ier5	Ier5	2410025L10Rik
Pabpc5	Astn1	Inpp1	Gadd45gip1	Ier5	Bnip2
4933407K13RIK	En1	Nap1l2	Limk1	St6galnac2	Mina
6030419C18Rik	Spata6	Neurod6	Atp6v0a1	Slc12a2	Prelid2
Tpst1	Spata6	Ltbp3	Sod3	Slc12a2	Epha3
Iffo1	Cyp2c50	Neurod4	Spa17	Slc22a4	Bmp4
Cpq	Abhd2	Eltd1	Lefty1	Galntl5	Ctbp2
Maged2	Hspg2	Ltbp2	Aldh1a3	Aldh1a3	Slc6a6
Scn4b	Actn3	Apoc2	Sspn	Sspn	Ctbp2
Gtdc2	Adad1	St6galnac4	Cdkn1c	Sspn	Rora
Pde3b	Calcr	St6galnac4	Pvalb	Cdkn1c	Tspan7
Nsg1	Ptprz1	B4galt2	Tbrg4	Lao1	LOC545261
Taf4b	Fech	Nrp1	Ndrg3	Pvalb	Prkaa2
A330076H08Rik,Mir344g	S100a13	Ppp1r14a	Abhd12	Elk3	Riok2
Efr3b	Slc38a3	Plvap	Grb14	Ndrg3	Ephb6
Letm2	Pcbd1	Crlf2	Mmp24	Rtn4ip1	Ntrk3
Nova1	Twist1	Adcy4	Kcna5	Grb14	Arhgef9
Il17d	B3galnt1	Hey2	Drg2	Ptpro	Trpc1
Shank2	Scn1b	Tcea2	Soat1	Opn3	Slc6a6
Hey1	Tesc	Gspt2	Soat1	Mmp24	Nap1l3
Hoxc4	Omd	Rbpj	Gtf2f1	Kcna5	Gnaq
Zkscan2	Psd3	Tgfb1i1	Arhgap6	Fam20c	Gprasp1
Phf20l1	Gfpt2	Dcx	Otub1	Soat1	Pcdhb9
Pcsk5	Tbx15	Kcnj8	Naglu	Soat1	Nfxl1
Tmem132e	Trh	Tfap2c	Syt3	Soat1	Eif2c3
Ache	Timd2	Kcne4	Gm9905	Arhgap6	Eif2s2
B020004J07Rik	Timd2	Nr2f1	Hba-a1///Hba-a	Hba-a1///Hba-a2	Sobp
Lrp1	Myoz2	Tfcp2	Got2	Myh6	Pctk2
Hspb6	Fads3	Tlr4	Laptm5	Akap8l	C030026M15Rik
Lypd1	Gbp8	Tlr4	Srsf9	Pygl	Kbtbd11
Epha7	Ccdc109b	Stx2	Mbd3	Coro1c	Il17rd
Nfkbie	Rxrg	Tfap4	Pygl	Pkd2	Dcun1d4
Rps6ka5	Trpm5	Zcchc14	Pkd2	Itga2b	Etnk1
Hoxc5	Grik5	Hebp1	Topors	Amy1	Npy1r
Hck	Mapk8ip2	Atg3	Topors	Pla1a	Endod1
Slit2	Mbl2	Sp1	Nr0b1	Ccl11	Agrn
Csf1	Tek	Gstt1	Cyb5b	Serinc3	Zbtb11
Penk	Fezf2	Ramp2	Pla1a	Wwtr1	9630023C09Rik
Stat5a	Ms4a8a	Malat1	Sncg	Tor1b	Utx
C430049B03Rik,Mir322,Mir351,Mir503	Cdh2	Galnt10	Rasgrp2	Gpx7	6430550H21Rik
Pde3a	Aqp5	Galnt10	Wwtr1	Cldn5	Tbx18
Vwc2	Cyp2a12	Plekhg2	Wwtr1	Cldn6	4921505C17Rik
Hapln3	Eno2	Aif1	Mug1	Spon2	A830059I20Rik
Ccnd3	Qprt	Pfn2	Gpx7	Fam181b	4930589M24Rik
Plcl2	Chrna1	Pfn2	Gm20738///Gm2	Ctsz	Vegfa
Shc2	Echdc3	Ggt5	Zfp704	Ctsz	Ctso
Pde8b	En2	Ceacam9	Spon2	Ctsz	Zfp618
PAGR1A	Ppp1r32	Foxf2	Ctsz	Gstt2	Cd1d1
Gnao1	Foxd1	Csn1s2b	Ctsz	Pdxp	Actn1
MRAS	[]	Lims1	Ctsz	Gzma	Jhdm1d
Hdac4	Gfra3	Apof	Nenf	Zp3r	Tmed8
Prrx2	Rhoj	Rbfox2	Gstt2	Prl7a2	Tef
Prlr	Aqp2	Rbfox2	1810009A15Rik/	1700001F09Rik///Gr	Slc5a3
1700104A03Rik	F5	Ddx25	Brms1	Amn	Slc25a23
Birc3	Bmp7	Htr3a	Dfna5	Pdlim4	Eif2c1
Tbl2	Acsl4	Loxl3	Gm10015///Gm1	Nab2	Snrpn /// Snurf
Nlgn2	Zeb1	Adamts8	Serpinc1	Igfbp6	Clock
Dcaf12l1	Habp4	Bhlhe22	Rap2b	Ccl9	Flrt2
Usp13	Ift57	Ezh1	Ccl22	Dact1	Plekhg2
Slc2a10	Nfil3	Fxyd5	Pdlim4	Rad51ap1	D030029J20Rik
Srsf12	Hlx	Dpysl4	Igfbp6	Fam3c	Arid4b
Idua	St3gal1	Dpysl4	Dact1	Pdlim7	Mpp5
Hoxc6	Gdf15	Dpysl4	Abhd3	Cpeb1	Ppm1a
Magee1	Drd2	Ppt2	Ilf2	Ghr	Hdac6
Matn2	Stac	Zfp276	Tspan1	Fbxo31	Trim47
Thbs3	Cideb	Nr5a1	Tnmd	Ada	Arhgef9
Hmcn1	Akr1c14	Rnf128	Btd	Eif4e3	Clock
Scara3	Cnp	Crem	Asic2	Tnmd	Lrrc15
Bmp3	Cebpa	Chkb///ChkbCpt1	Ngb	Btd	Neo1
Tmem47	Ms4a4d	Pgpep1	Tmem176b	Svs3a///Svs3b	2810439F02Rik
Trib2	Npy5r	Agtpbp1	Cml1	Svs3a	Tagln
Fam171b	Zfpm2	Gtf2ird2	Bhlhe40	Asic2	Cmklr1
BICC1	Corin	Tnfsf12///Tnfsf1	Inpp1	Ngb	Flrt2
Gm9159	Fads2	Nucb2	Ltbp3	Tmem176b	Zfp612
Mrc2	Rtn2	Foxg1	Eef1a2	Paxbp1	Psd2
Cdh2	Nepn	Wbscr27	Kera	Cml1	4930589M24Rik
Gdf11	Cys1	Rnf112	Tfpt	Cpd	Manba
Daam2	Pcp2	Ftl1///Gm20746,	Ndufa10	C4a///C4b	Sox11
Gpsm1	Stom	Hist2h2aa2	St6galnac4	Bhlhe40	Sox11
Rian	Vstm2b	Hist2h2aa2	B4galt2	Abcc5	Zmym5
Abl1	Tpmt	Tnnc1	Dnajc30	Abcc5	Krt27
Dlg4	Hoxd9	Gpr124	Ppp1r14a	Iqcf3	Krt25
Sat2	Slc22a13	Apcdd1	Ufd1l	Eltd1	Mcpt4
Jdp2	Tlr2	Cd97	Plvap	Eltd1	Cma1
Asph	Shank3	Gpsm3	Car4	Apoc2	Mrgprb1
Smarca1	Cypt1///Cypt7///Cy	Zfp275	Smpx	St6galnac4	Pscdbp
Arrb1	Gck	Larp6	Adcy4	B4galt2	Sla
Trps1	Sox4	Adam19	Chd3os	Nrp1	H2-Ea
Fbln2	Nox4	Rad9a	Hes1	Ppp1r14a	Aqp3
Tex15	Svep1	Tpd52l1	Hey2	Plvap	Rgs1
Tns3	St8sia4	Psg23	Tcea2	Smpx	Ap1s3
Efemp2	1700012L04Rik///G	Gm11397///Serp	Rtkn2	Crlf2	Serpinb1a
Lrrtm4	Hipk3	Tnfaip6	Gspt2	Adcy4	Ckmt1
Chrm2	Il4i1///Nup62-il4i1	Slc16a2	Mrpl10	Hey2	A030004J04Rik

CRTC1	Fxyd7	Slc16a2	Cyp2d10	Tcea2	Tacstd2
Zfp945	Cd37	Islr	Ubfd1	Gspt2	Cpa3
Pcdhb16	Zfp37	Gng2	Tgfb1i1	Cyp2d10	S100a9
Slc8a3	Hrh1	Gng2	Kcnj8	Adcy6	Hdc
Lynx1	Icosl	Mfap5	Kcne4	Samhd1	H2-Aa
Ampd2	Tbxa2r	Copz2	Nr2f1	Bcl3	Gpr65
Map2k6	Dtna	Matn4	Tlr4	Tgfb1i1	Krt14
Lama2	Cacna2d3	Smarcd3	Zcchc14	Dcx	Ms4a2
Kdm1b	Elac1	Nrip1	Zcchc14	Dcx	Il1rl1
Rbms3	Tex14	Yes1	Tceal8	Kcnj8	Serpinb1a
Hoxa9	Cdk14	Pgf	Hebp1	Tfap2c	Pscdbp
Igfbp5	Cdk14	Matn1	Dbp	Myot	Krt15
Stk40	Sptbn1	Ppbp	Ndufaf3	Kcne4	Tpsb2
Adam22	Rsu1	Tekt2	Gstt1	Nr2f1	Rgs5
Gsto2	Poc5	Slc4a3	Ramp2	Tfcp2	Dsg1a
Slc4a3	Cyhr1	Grem2	Mnt	Tlr4	Ripk4
Slc22a21	Ift81	Foxa1	Ipo4///Tm9sf1	Tlr4	Rgs5
Pcdhb17	Htra3	Fgf13	Plekhg2	Zcchc14	Krt28
Aff2	Flt1	Nap1l3	Pfn2	Zcchc14	Sla
Hoxd3	Invs	Nudt4	Pfn2	Zcchc14	Gfi1
Lpar4	Rfxank	Socs2	Omg	Tceal8	Krt1
Zfp36	Tinag	Dpt	Foxf2	Hebp1	H2-Aa
Dchs1	1700028P14Rik	Stk3	Nfu1	Ramp2	Krt35
Srgap3	Klf7	Pcm1	Lims1	Plekhg2	Shh
Zfp334	Ss18	Fzd2	Rbfox2	Sdf2l1	Stfa1 /// Stfa3
Ptch1	Gosr2	Rgl1	Gla	Krt23	1110020A10Rik
Cpne2	Fibin	Isoc2b	Apip	Foxf2	Krt14
Prdm5	Zfp423	Kdelr3	Slc12a7	Csn1s2b	BB144871
Stxbp1	Dhrs4	Wasf1	Loxl3	Lims1	Serpinb5
Abcd1	S100b	Stambpl1	Bhlhe22	Gbp2	Il7r
Klhl14	Pde10a	Defa-rs1	Gm10349///Gm9	Rbfox2	Cyp11a1
Shd	Abcg5	Cbfa2t3	Vps72	Rbfox2	Tpsab1
Apc2	Six6	Ccnh	Zfp87	Gla	Krt71
H6pd	Batf	Kpna6	Ifit2	Padi2	Dmkn
Spon1	Gng13	Fgfr4	Fxyd5	Loxl3	Pkp1
Frmd3	Vegfc	Col11a1	Dpysl4	Adamts8	Bnc1
Ston1	St6galnac5	Klf1	Dpysl4	Apoc3	Ripk4
Trim34a	Slc2a10	Cdh15	Fn3k	Rad51	Adh1
Dagla	Sim2	Avpr1a	Rbfox1	Serpina1b	S100a14
Ppox	Matn2	Hoxd10	Rnf128	Vps72	Cpz
Sfrp2	Wdr86	Gpr162	Eci1	Ifit2	Krt73
Sobp	Nkd2	Kcnj1	Pgpep1	Fxyd5	Sprr1a
St6gal2	Nkd2	Kcnj1	Agtpbp1	Dpysl4	Cd74
Cep170	Clec14a	Astn1	Rab4a	Ppt2	Gjb2
Pcdha1,Pcdha10,Pcdha11,Pcdha12,Pcdha2,Pcdha3,Pcdha4,Pcdha4-g,Pcdha5,Pcdha6,Pcdha7,Pcdha8,Pcdha9,P	Clec14a	En1	Tnfsf12///Tnfsf1	Cdhr1	Serpinb5
Clec14a	Gnb4	Phox2a	Insl6	Rlbp1	Krt5
Hs3st3a1	Gnb4	Khdrbs1	Cog1	Agtpbp1	2010205A11Rik /// EG628
Lsamp	Sell	Spata6	Foxg1	Mtf1	1600029D21Rik
Cadm2	C3ar1	Cyp2c50	Rnf112	Recql	Epgn
Ranbp17	Fam19a5	Mpdz	Gas8	Rab4a	Irf6
Mov10	Fam19a5	Ptx3	Ftl1///Gm20746,	Gtf2ird2	Dsc3
Plekhg2	Pabpc2	Hspg2	Siva1	Tnfsf12///Tnfsf12Tnf	Adipoq
Runx1t1	Ghdc	Akr1c13	Gpr124	Dnmt3b	Gata2
Hip1	Stc2	Actn3	Terf1	Cog1	Rbm35a
Zic3	Pitx1	Adad1	Apcdd1	Nucb2	Serpinb5
Tenc1	Igf1	Cts6	Apcdd1	Wbscr27	Hdc
Masp1	Tph1	Ptprz1	Gbp3	Rnf112	Mpzl2
Npr2	Fthl17///Gm14458,	2810428I15Rik	Cd97	Zfp42	Rgs5
Gm10400	Ppp1r2-ps9	S100a13	Zfp275	Retnlb	1600029D21Rik
Fam161a	Arg1	Ngp	Tspan32	Pgam2	Serpinb2
Inpp1	Zfp467	Twist1	Larp6	Gpr124	Actc1 /// LOC100048431
Dfna5	Slc28a3	B3galnt1	Adam19	Apcdd1	Ptprc
Bcl2	Slc28a3	Tesc	Rad9a	Apcdd1	Fyb
Pdgfra	Lgals1	Omd	Pde8a	Mphosph8	AW551984
Gnl2	Ttc28	Sit1	Prl8a6	Phf21a	BC100530 /// Stfa1 /// Stfa
Agphd1	Rp2h	Gfpt2	Tnfaip6	Gbp3	Dpp4
Ptchd4	Cd93	Tbx15	Mkrn1	Itga7	Figf
Man1a	Unc5c	Trh	Mlx	Cd97	Trp63
Tram1l1	Defb4	Trim69	Fabp2	Tspan32	5033414K04Rik
STAT2	Ifi204	Cd2	Slc16a2	Larp6	Cdh1
	Zbp1	Fads3	Slc16a2	Adam19	Col17a1
	1700010I14Rik	Ccl25	Rras	Adam19	Ccl11
	Hspa1l	Ccdc109b	Islr	Cav3	Serpinb5
	Ghrh	Rxrg	Gng2	Msc	Calm4
	2310057J18Rik///2	Grik5	Gng2	Tnfaip6	Mt4
	Slc25a36	Mapk8ip2	Mfap5	Sp7	Dsp
	C920025E04Rik///H	Tek	Copz2	Fabp2	Figf
	Ogn	Fezf2	Anapc7	Slc16a2	Dct
	Ogn	Cds2	Ccdc91	Slc16a2	Ptgds
	Nupr1	Ms4a8a	Rnf141	Islr	Krt17
	Nupr1	Srpk3	Smarcd3	Gng2	EG628498 /// ENSMUSG0
	Sgcb	Bhlha15	Yes1	Gng2	Tpsb2
	Prtn3	R74862	Pgf	Mfap5	Ror1
	Spock1	Sct	Aspa	Smarcd3	Scel
	Spock1	3110070M22Rik	Matn1	Anxa11///Gm2260//	Myb
	Masp1	Cdh2	Ppbp	Pgf	Perp
	Lats2	Aqp5	Grem2	Aspa	EG628498 /// ENSMUSG0
	Gpc6	Eno2	Foxa1	Matn1	Ifi202b
	Gpc6	Slc30a4	Fgf13	Ppbp	Spint2
	Camp	Ap4m1	Nap1l3	Slc4a3	Asprv1
	Colec12	Epc1	Hspa9	Calcrl	Krt79
	Prom1	Aox3	Socs2	Grem2	Krt85 /// LOC100044179
	Col5a3	Echdc3	Dpt	Ebf2	Chrdl1
	Akap12	En2	Stk3	Foxa1	Sytl2
	Wnt6	Sfxn4	Stk3	Fgf13	Csta
	1700029F12Rik	Foxd1	Pcm1	Fgf13	Pof1b
	Gabrb1	[]	Dad1	Kcne3	Krtdap
	Gp9	Acrv1	Fzd2	Nap1l3	Myog
	Tpm2	Gfra3	Fzd2	Socs2	Prkcb1
	Pde6b	Nlrp5	Rgl1	Dpt	S100a3
	Abcd2	Rhoj	Kdelr3	Rgl1	Clca2
	Abcb1a	Ufm1	Cenpo	Kdelr3	Cd53

Abcb1a	F5	Wasf1	Wasf1	Rab27b
Cul2	Bmp7	Stambpl1	Stambpl1	Abcc9
[]	Acsl4	Cetn2	Cga	Trp63
[]	Bhmt2	Cbfa2t3	Opn1sw	Dpp4
AA672641	Spp2	Traf3	Rtp4	Coro1a
Rpl27a	Cldn15	Top3a	Cbfa2t3	Myb
[]	Cadm3	Col11a1	Ccnh	Plau
Ccdc12	Cadm3	Avpr1a	Nrsn1	LOC666466 /// Obox2
Hdlbp	Slc17a3	Astn1	Fgfr4	Lum
Adcy9	Zeb1	En1	Avpr1a	Kit
Pkhd1	Habp4	Icam5	Avpr1a	Abca9
Eif3e	Ift57	Rab2a	Slfn1	Lpl
Arap3	Nfil3	Cyp2c50	Astn1	H2-Eb1
[]	Dio2	Ino80b	En1	8430420F16Rik
Prpf19	Hlx	Rmdn1	Clu	Cd200r3
Krt72	Ift74	Abhd2	Khdrbs1	Adrb1
Gm2682	St3gal1	Mpdz	Ube2h	Astn2
[]	Nek3	Hspg2	Tspan13	Fxyd3
[]	Gdf15	Actn3	Spata6	H2-Ab1 /// LOC641240 ///
[]	Txlnb	Alg13	Cyp2c50	Nat8l
2810449G22Rik	Zfp93	Lin7b	Rmdn1	Krt26
[]	Stac	Hnrnpc	Abhd2	Rgs18
D930048N14Rik	Nosip	Inmt	Mpdz	Zcchc5
Tet1	Cideb	2810428I15Rik	Hspg2	Lpl
Hpgd	Akr1c14	Apoc4	Actn3	Sp6
Hpgd	Cebpa	Pcbd1	Serpind1	Rgs5
Fcrla	Ms4a4d	Twist1	Adad1	Ikzf1
[]	Lyrm5	B3galnt1	Calcr	Slco4a1
Srxn1	Bves	Tbx15	Ttc36	Clec12b
C77144	Klk1b5	Trim69	Rbms1	Lgals7
Cphx3	Rhag	Nit2	S100a13	Ifi202b
Deptor	Wisp2	Bpnt1	Slc38a3	Rgs4
Slc35a5	Corin	Timd2	Mrps25	Dapp1
Arhgef25	Akap4	Cpb2	Twist1	Stfa2l1
Creb3	Gif	Fads3	H2-Q2	Aoc2 /// Aoc3
Gba2	Fads2	Gbp8	B3galnt1	Marveld3
[]	Csnk2a1	Ccdc109b	Scn1b	Agtr1a
Zfp644	Cyp2d22	Grik5	Tesc	Clec4d
Rab3il1	Cyp2d22	Srpk3	Omd	Gucy1a3
Rab3il1	Pcnx	R74862	Psd3	1190003J15Rik
Xbp1	Rtn2	Fasl	Gfpt2	Car3
Aak1	Slc5a1	3110070M22Rik	Adcy8	Dsp
C80171	Ugt8a	Cdh2	Tbx15	2200001I15Rik
Slu7	Cys1	Aqp5	Cyth3	C130090K23Rik
Maf1	Cys1	Arl8b	Cd55	Osr2
[]	Tmeff2	Qprt	Timd2	Clca2
Mtfr1l	Mpp3	Foxd1	Fads3	Lypd3
[]	Pcp2	[]	Gbp8	Il1r2
Tktl1	Pcp2	Gfra3	Ccl25	Ucp2
Fzd3	Sf3a1	Nlrp5	Ccdc109b	Sox21
[]	Slk	Csnk1d	Rxrg	Krt16
Zfp334	Nr1h4	Rhoj	Trpm5	Rgs18
Zfp334	Ophn1	Rpn2	Grik5	Odz2
AA517858	Nlk	Chid1	Mapk8ip2	Dsc1
Pttg1ip	Alg14	F5	Tek	Penk1
AA517023	Vstm2b	Bmp7	Fezf2	Vav3
Spsb1	Pdgfc	Cadm3	Srpk3	Kit
Igll1	Ptpn18	Cadm3	R74862	Ctsg
[]	Hoxd9	Zeb1	Serpina5	Tnxb
C76628	Npy	Habp4	Cdh2	Adh7
Tmem191c	Ltb	Ift57	Cyp2a12	Grhl2
[]	Shank3	Nfil3	Irgm1	Gpr87
[]	Acvr2b	Dio2	Eno2	Abcc9
[]	Ctsr	Dio2	Agxt	Rassf9
[]	2810417H13Rik	Pcdhb22	Arg2	Cd200r2 /// Cd200r4 /// L(
[]	Sox4	Cysltr1	Ppp1r32	Nfib
[]	Nox4	St3gal1	Abcb1b	Eppk1
[]	Zfp260	Gdf15	Sfxn4	Rgs7bp
[]	Fam174a	Fbxo16	Foxd1	Tcrg-V3
[]	Svep1	Tssk6	[]	Gata2
Prss58	Mlxipl	Stac	Gfra3	Hopx
[]	1700012L04Rik/,	Slc35g3	Rhoj	Krt10
Prss29	Il4i1///Nup62-il4	Skp2	Rpn2	Dlk1
[]	Fxyd7	Bcl10	F5	Acpp
[]	Zfp37	Akr1c14	Bmp7	Gstm2
[]	Hrh1	Cebpa	Spp2	Slc39a8
AA414903	Cacna2d3	Ms4a4d	Cldn15	4632413I24Rik
[]	Cct6b	F10	Zeb1	Nfib
[]	Elac1	Baat	Habp4	Dlx2
Clec4e	Aire	Bves	Maff	E130203B14Rik
Lce1d	9530002B09Rik	Crybb3	Dio2	Slc4a4
Slc12a8	Rgs2	Rhag	Dio2	Fgfbp1
Gbx2	Cdk14	Corin	Hlx	Grhl3
Asb12	Cdk14	Mcf2	Pcdhb22	Tacstd1
Lhx5	Sptbn1	Eno1///Eno1b	St3gal1	Kcne3
Ptgfr	Acad8	Eno1	Gdf15	Tmcc3
Krtap13	Tns1	Fads2	Stac	Figf
Spesp1	Ift81	Cntnap4	Skp2	Pkp3
Csn2	Jam2	Rtn2	Bcl10	Lhx2
St8sia2	Mettl6	Ier5l	Cideb	Plek
Apobr	Htra3	Cys1	Akr1c14	Myb
Pard3	Creb3l1	Saa1	Cnp	Arhgap24
Ubl4b	Flt1	Apom	Cebpa	Adamts16
Sval1	Heyl	Ophn1	Ms4a4d	Cntfr
Ramp3	Heyl	Hoxd9	Bak1	Dsg2
Atp2b2	T	Itgax	Gtsf1l	Mmp2
Slco1a4	Invs	F13b	Cryba2	Apoc1
Peg12	Pdpn	Tlr2	Zfpm2	Tmprss13
Slc7a11	Atp2a1	Shank3	Corin	ORF63
Sema3a	Saa4	2810417H13Rik	Eno1///Eno1b	Gzma
Sema3a	Chst5	Sox4	Eno1	Cldn1
Pcdhb21	Pdxdc1	Sox4	Fads2	Esrrg

Tcl1b4	Sorbs3	Sox4	Csnk2a1	Itgb2
Prdm1	Cdh17	Acy1	Iigp1	Nfia
Pcdhb3	Ctla4	Svep1	Rtn2	C1qtnf2
Cldn16	Neu3	Il4i1///Nup62-il4	Ugt8a	2210023G05Rik
1700011A15Rik	Epha8	Fxyd7	Cys1	Grem1
Orm2	Pth	Zfp37	Saa1	Elmod1
Ppy	Bpifa2	Icosl	Mpp3	S100a8
Sult1e1	Cyp2d9	Tnfrsf11a	Tnfsf11	Mcoln3
Krtap5-2	Fibin	Cep131	Timp3	Gp49a /// Lilrb4
Dvl3	Fxyd2	Vangl2	Timp3	Plch2
Il1rapl2	Zfp423	Xirp1	Tdo2	Dcn
Gm2083///Mup1//	S100b	Tbxa2r	Stom	Ndst3
4933411G06Rik	Pde10a	Cacna2d3	Vstm2b	Ctnnd2 /// LOC100045979
Vtn	Pde10a	Cct6b	Hcst	Sprr2f
Klk1b16	Myog	Elac1	Mettl1	Prss35
Stxbp1	Abcg5	9530002B09Rik	Pdgfc	Krt77
Dkk2	Hc	Cdk14	Hoxd9	A430001F24Rik
Tmem47	Tac2	Cdk14	Npy	Adam33
a	Vegfc	Rsu1	Shank3	Mmp2
Gpatch2	St6galnac5	Snrpb	Cypt1///Cypt7///Cyp	Kif2c
Crybb2	Ank1	Adrm1	Cd163	1190003J15Rik
Chn1	Stab2	Poc5	Smtnl1	Coro1a
Alx3	Ccl21a///Ccl21b/	Usp48	Gck	C2
Selp	Csf3	Ift81	Serpine1	9330182L06Rik
Shox2	Cyp26a1	Htra3	Sox4	Ovol1
Trpc7	Slc2a10	Heyl	Sox4	Ucp2
Insrr	Matn2	Rfxank	Sox4	Cldn1
AK009121	Tbc1d1	Padi1	Nox4	Sorcs2
Lpar2	Pias2	Sorbs3	Zfp260	Mt2
Ptp4a2	Wdr86	Ctla4	Zfp260	Nckap1l
Dnaja3	Nkd2	BC051019	Svep1	Rgs4
Bscl2	Nkd2	Klf7	Fhl2	Si
Sqrdl	Clec14a	Sorcs2	St8sia4	Vwa2
Zfhx3	Clec14a	Mrpl35	1700012L04Rik///Gn	Bpil2
Ate1	Gnb4	Zmat5	Il4i1///Nup62-il4i1	Dlx3
Ucp3	Gnb4	Fibin	Wfdc15b	Dapp1
Lrrc6	Clec2d	Fxyd2	Fxyd7	H19
Doc2b	Fam19a5	Zfp423	Zfp37	Tyr
Doc2b	Fam19a5	Dhrs4	Cxcl1	St14
Sgce	Pabpc2	Ubqln1	Hrh1	Cd52
4933415F23Rik	Ghdc	Pde10a	Icosl	F2rl1
Fgf10	Ggps1	Abcg5	Tbxa2r	Ly6a
Csf2ra	Ces1e	Mns1	Cacna2d3	Tyr
Srcin1	Msh4	Batf	Krt12	Nfib
Hpn	Igf1	Gng13	Helb	Opcml
Cd247	Nat8	Rarg	Tex14	Rgs7bp
Tex15	Zfp94	Vegfc	Cdk14	Dsg2
4921536K21Rik	Fgr	St6galnac5	Cdk14	Bcl2a1a /// Bcl2a1b /// Bcl
Cab39l	Comp	Ptf1a	Eps15	2610301F02Rik
Mzf1	Sult2a2	Gaa	Mthfd2	Mcoln3
Cntn3	Il12b	Slc2a10	Mthfd2	EG328264
Il25	1700102P08Rik	Matn2	Sptbn1	Lrmp
Ppp3cc	Olr1	Gnai2	Dazap1	Ap2b1
Adat1	Fthl17///Gm144!	Pias2	Pip4k2a	Cxcl12
Fut7	Ppp1r2-ps9	Magt1	Ccl12	Zim1
1700019G17Rik	Dazl	Gtl3	Cyhr1	Ikzf3
Dhx58	Arg1	Wdr86	Ift81	P4ha2
4933400A11Rik	Elf5	Nkd2	Jam2	Twist2
Tas1r3	Birc6	Nkd2	Htra3	Tusc5
Tnfrsf17	Zfp467	Clec14a	Flt1	Nfia
Gm10256///Gm10:	Fank1	Clec14a	Fzd4	Zfp179
Art2b	Isg20	Gnb4	Heyl	Ly6d
Ahrr	Lgals1	Gnb4	Tnfrsf13c	Fn1
Pcdha1///Pcdha10,	Mbl1	Nudc	Invs	Tacstd1
Myl10	Dlg4	Fam19a5	Pdpn	Ptgs2
Hdac7	Ttc28	Fam19a5	Atp2a1	Grhl1 /// LOC100043996
Vamp2	Spag1	Slco1a6	Ccnt1	Aspn
Vamp2	Cd93	Pabpc2	Tinag	C77370
Ntrk2	Unc5c	Krtap15	Saa4	Clec7a
Ntrk2	Ifi204	Ces1e	F7	Nfib
Plekha3	Clec10a	Cnih3	Lhx9	Ncapg
Gm21540///Tmed2	Tnf	Igf1	1700028P14Rik	Gm266
Gucy1b3	4632415L05Rik	Zmynd19	1700080E11Rik	Gm885
Gm4887///Twf1	Kcnn1	Irx4	Klf7	1600029D21Rik
Sep-06	1700010I14Rik	Fthl17///Gm144!	Klf7	Gjb3
Nsd1	Ghrh	Atp6v1c1	Klf7	Gda
Sln	2310057J18Rik//	Cd47	Ss18	Zfp750
Xbp1	Zfr	Lipc	Gosr2	Gas1
Tgfbr1	Slc25a36	Lgals1	Atp6v1b1	4833423E24Rik
Tgfbr1	C920025E04Rik/,	Mbl1	Fibin	Mmp23
St6galnac3	Ogn	Ttc28	Zfp423	Krtap3-2 /// Krtap3-3
Il17ra	Srr	Rp2h	Dhrs4	Ereg
Vegfa	Nupr1	Unc5c	S100b	Hmga2-ps1
Ppap2c	Nupr1	Ifi204	Pde10a	1100001H23Rik /// LOC1(
Mfge8	Sgcb	Ccr1	Abcg5	Htra3
Hs6st2	Prtn3	4632415L05Rik	S100a8	Rab27b
Rgs5	Spock1	4632415L05Rik	Batf	Ror1
Atrx	Spock1	Kcnn1	Gng13	Cobll1
Enc1	Masp1	Prss21	Vegfc	A630038E17Rik
Arid5b	2810408M09Rik,	Hspa1l	St6galnac5	Ifit2
Kif3b	Gpc6	Ghrh	Ccl21a///Ccl21b///C	Tmem54
4933411K20Rik	Gpc6	2310057J18Rik//	Cntfr	Muc15
Hsd17b11	Camp	Nkain1	Spam1	Ccrl2
Aaed1	Ltc4s	Yae1d1	Slc2a10	Trim29
Ranbp17	Colec12	Ogn	Tsks	Gpr109a
Acyp1	St8sia1	Ogn	Matn2	Loxl1
Gna12	Prom1	Nupr1	Tbc1d1	Plek
Mef2c	Anks3	Nupr1	Gnai2	Ppp1r14a
Mef2c	Col5a3	Sgcb	Wdr86	Gfra2
Npas2	Akap12	Prtn3	Nkd2	Pla2g2e /// Pla2g2f
Kcnn4	Wnt6	Ftcd	Nkd2	Efemp1
Mrc2	Gabrb1	Spock1	Clec14a	Itih5

Alg2	Cxcl5	Spock1	Clec14a	Abca9
Mllt1	1700037H04Rik	Dpep1	Gnb4	Ptgfr
Guca1a	H2-DMb2	2810408M09Rik,	Gnb4	Irx4
Jak2	Abcd2	Gpc6	Ehf	Trpm1
Jak2	Nfx1	2610002M06Rik,	Ehf	Samsn1
Phf2	Abcb1a	Camp	Sell	Lgr5
Gpc4	Abcb1a	Colec12	Fam19a5	S100a16
Trpc1	Nkap	St8sia1	Fam19a5	LOC100047619 /// Slc7a5
Cml2	Padi3	Prom1	Pabpc2	Arhgap30
Nkx2-2	Gm16343///Gm1	Col5a3	Ghdc	C920025E04Rik /// H2-T23
Cdk5r1	Dnajc1	Car5b	Stc2	6330406I15Rik
Zfp111	[]	Akap12	Ripk1	Esr1
Edn3	AA517864	Wnt6	Ces1e	Rnf43
Gnao1	AA516738	Gabrb1	Igf1	Nfia
Hcn2	[]	Tpm2	Comp	Aspn
Abcg3	Rpl27a	Abcd2	Il23a	Aqp3
Plcb1	[]	Abcb1a	Nhlh1	5033414K04Rik
Thap2	Hdlbp	Abcb1a	Ppp1r2-ps9	Plek
Mgat4c	Cog4	Nkap	Fahd1	Sox21
Ttyh2	S100a1	Aplf	Arg1	Lcp1
Mettl7a1	Kif17	[]	Stk39	Plac8
Ccr2	Kif17	[]	Stk39	Nrn1
Ptpn11	Mark1	AA516738	Fank1	Chst11 /// Phactr1
Itgav	[]	Agap2	Isg20	Mmp12
Tro	Prpf19	LOC102638854	Slc28a3	Il18r1
Chrna4	[]	2610019E17Rik/,	Slc28a3	Plxnc1
Plxna1	[]	Hdlbp	Lgals1	Egfr
Pknox1	Tlr7	Adcy9	Slc7a12	Thbs2
Ptprt	[]	S100a1	Dlg4	Lama5
Pax9	[]	Errfi1	Ttc28	Arg2
Slc39a9	[]	Arap3	Cd93	Ptgs2
Zfp40	2810449G22Rik	Dlx1as	Gsdmc///Gsdmc2	Mpzl2
Pigb	2810449G22Rik	[]	Unc5c	AW551984
Lipg	[]	2810449G22Rik	Ifi204	AI661453
Ugcg	[]	C77545	Zbp1	Il1b
Pik3ap1	[]	Tet1	Ccr1	Sbsn
Hspb7	C77545	Hpgd	Ccr1	Oxtr
Cacna1c	AA536748	D15Ertd55e	4632415L05Rik	Myh11
Lef1	Tet1	D15Ertd55e	Col7a1	LOC666466 /// Obox2
Pcdh10	Tet1	C030018P15Rik	Pttg1	9830134C10Rik
Cttn	Hpgd	Srxn1	1700010I14Rik	Zbtb16
Ptgfrn	Hpgd	D4Ertd117e	Hspa1l	Lox
Qtrtd1	Rnf20	C80678	Ghrh	Spn
Axin2	Tmed7	Deptor	2310057J18Rik///23	Laptm5
[]	[]	Slc35a5	Mbp	C230078M08Rik
Lrat	Lrig1	Arhgef25	Nkain1	Ptgfr
Tex9	D15Ertd55e	Rab3il1	Nkain1	E2f1
Frk	[]	Anapc16	C920025E04Rik///H2	Ccl5
Mab21l1	Pdrg1	Nsun5	Ogn	4930570C03Rik
Fxyd1	C030018P15Rik	Xbp1	Srr	2610018G03Rik
Traf6	D4Ertd117e	Slu7	Nupr1	Ccnd2
Prlr	Zfand3	C78859	Nupr1	4732474O15Rik
Slc12a1	C77144	Mtfr1l	Sgcb	Tns4
Vipr2	Deptor	AA675344	Prtn3	Ccnd2
Xiap	C77370	Cd160	Spock1	D3Ertd258e
Kcnmb1	Slc35a5	[]	Spock1	LOC100044106 /// Plxnc1
Pi15	Slc35a5	[]	Dpep1	Cldn4
Msi1	Arhgef25	Fzd3	Masp1	Mlana
Pstpip2	Map3k7	D13Ertd205e	Lats2	Dmrt2
Elac1	Rab3il1	[]	Lats2	Anpep
Ccr10	Rab3il1	Coprs	Cep41	Cpe /// LOC100046434
Angptl1	[]	Coprs	Camp	Sorl1
Hhip	Nsun5	Pttg1ip	Colec12	Loxl1
Arl10	Xbp1	AA517023	St8sia1	Nfia
Zfhx4	Xbp1	Spsb1	Prom1	Ehf
Grid2	Aak1	Tmem191c	Col5a3	Centb1 /// Tnk1
Pcdhb14	Aak1	[]	Car5b	Lrig1
Pcdhb14	C80171	Slc8a1	Wnt6	Mmp23
Wnt8b	Slu7	[]	Il3ra	9630041G16Rik
Angpt1	Chek1	[]	Cd274	Thbs1
Onecut1	Fam73b	Smarcd1	Gp9	Ahrr
Crb1	[]	[]	Slc26a4	2310014G06Rik
Lepre1	Maf1	Ccl6	Daf2	Ptgfr
Runx3	Jmjd6	[]	Tpm2	Odz2
Zfp318	Jmjd6	2610201A13Rik	Pde6b	Marveld2
Ugt2a1///Ugt2a2	Mtfr1l	2610201A13Rik	Abcd2	Chmp4c /// LOC10004485
Egr3	[]	Gpank1	Slc22a27	Dlx4
Rabgap1l	Tktl1	[]	Abcb1a	Hist1h1e
Krtap4-2	[]	Pitpnb	Abcb1a	Krt6a
Cep250	[]	Clec4e	Dnajc1	Dnajc15
Kcns1	[]	Slc12a8	[]	Slc35a2
Epha6	[]	Adamtsl5	AA516738	Galntl1
Gabrq	Fgf4	Gbx2	Agap2	Slc6a2
Hoxd3///Hoxd4	Fzd3	Cspp1	AA672641	Dock2
Zic4	Zscan21	Gdap10	2610019E17Rik///Ra	Rarres2
Fbxo4	[]	Lhx5	[]	Centb1 /// LOC100045877
Cdx4	[]	Ptgfr	Hdlbp	Pde1b
Adrb3	D13Ertd205e	Lta	Cog4	Gata3
Grid1	[]	Grk4	Errfi1	Gpc6 /// LOC100045283
Hif3a	Zfp334	Sva	Ksr1	Areg
Evx2	Zfp334	Spesp1	[]	Fbln1
Cd46	Coprs	Sntb2	Krt72	Hlf
Ncam2	Coprs	Kif3a	Gm2682	Pde7b
Ncam2	[]	St8sia2	[]	LOC640441 /// Thbs1
Cx3cl1	Tcta	Slco1a1	[]	6430704M03Rik
H2afy2///H2afy3	Pttg1ip	Prss12	[]	Eln
Il5ra	AA517023	Zfp354a	2810449G22Rik	Pycr1
Enah	Slc6a6	Pard3	2810449G22Rik	Dlx4
Tmc1	Spsb1	Ramp3	[]	Tmtc2
Hapln1	AA408954	Peg12	DXErtd223e	Casp12 /// LOC100044205
Sox17	AA408954	Tnfsf10	Tet1	Krtap17-1
Msmb	AA409749	Sema3a	Zc3h7a	C2

Vcan	AA511261	Pcdhb21	Hpgd	Ptpn6
Enam	Dntt	Hypm	Hpgd	1200016E24Rik
Zfp59	D11Ertd326e	Prdm1	Fcrla	Pcsk6
Scn3a	Igll1	Ager	Mphosph9	E2f2
Tmc2	[]	Pcdhb3	D15Ertd55e	Sfrp2
Vmn2r26	[]	Rptn	Gm12264	Tmeff2
Nr5a1	[]	Ppy	C030018P15Rik	Gpc6 /// LOC100045283
Matk	Slc8a1	Rhox2a///Rhox2	Tmem38b	Igsf10
Fbxo17	[]	Gm2083///Mup1	C80678	Klf14
Gpr132	[]	4933411G06Rik	Tubb2a-ps2	Cst7
Htr6	AA414993	Mtpn	Slc35a5	2900076A13Rik
Bmp10	[]	Nap1l1	Arhgef25	Gyltl1b
Vmn1r172	[]	Stxbp1	Creb3	Ctsh
Pax5	Smarcd1	Dkk2	Gba2	Hist1h2bb /// Hist1h2bc //
Uchl4	Glb1l	Tmem47	Rab3il1	Cited2
Lmx1b	Tubg2	a	Wwc1	Spint1
Olfr74	Ccl6	Eral1	Xbp1	LOC665506
Ang3	D2Ertd63e	Ccdc50	Xbp1	Ung
Efna5	Prph	Masp2	C80171	Tyrp1
Uts2	[]	Magi2	Slu7	Il10ra
Defb3	[]	Gucy1a3	[]	Plxnc1
Defb5	2610201A13Rik	Chrdl2	[]	Hs6st2
Epdr1	[]	Rit1	Jmjd6	Ccrl1
Bcl6	Tap1	Rdh16	Jmjd6	S100a16
Bace1	Prss58	Chn1	Mtfr1l	Ddc
Map1b	Col9a3	Knop1	AA675344	Pde7b
Fgl2	Prss29	Alx3	[]	Pilrb1
Fgl2	[]	Selp	Tktl1	Ctsh
S100a3	[]	Shox2	[]	Esrrg
Nr3c1	[]	Chrne	[]	Rgs4
Trim44	Rep15	Gria3	[]	Cdca5
Sh3bgrl	Clec4e	AK009121	Vmn2r103///Vmn2r	Rab25
Mapk9	Slc12a8	Chad	Fgf4	Tmcc3
Sos1	Dmc1	Hoxd1	[]	Phactr1
St3gal2	Cspp1	Cd209e	Zfp334	Klhl13
Elk1	Cldn14	Zfp493	Zfp334	Lhfpl2
Mr1	Bpifa1	Tpo	[]	Pvrl4
Med29	Lhx5	Rbm18	AA517858	Opcml
Pdgfra	Ptgfr	Prkacb	AA517023	Mt1
Pdgfra	Lce1a2	Aes	Spsb1	Gucy1a3
Serpina3m	Grk4	Dnaja3	AA409749	Tnfrsf18
Sh3bp5	Ninj2	Bscl2	D7Ertd183e	Mgl1
Sh3bp5	Dkk1	Sqrdl	Igll1	Klhl13
Slc2a3	Bik	Zfhx3	Igll1	Mcm5
Mapk11	Csn2	Zfhx3	[]	Elovl7
Tbl2	Sntb2	Doc2b	Tmem191c	Ercc6l
Adcy3	Sntb2	4932438H23Rik	Gadd45b	Ucp2
Dnaaf1	Kif3a	Sgce	[]	Fxyd6
Add2	St8sia2	Fgf10	[]	Sbsn
Mmp19	Zfp354a	Il2ra	[]	Casp14
Papss2	Gfi1b	Dach1	[]	Mmp19
Papss2	Sval1	Srcin1	[]	Dsg2
Papss2	Ramp3	Pparg	Ankzf1	Lpxn
Igfbp4	Atp2b2	Tex15	Glb1l	Pam
Eif2ak2	Peg12	Tcp11	Tubg2	Cntfr
Foxp2	Nr5a2	Pmfbp1	[]	Mapre2
Zfp319	Sema3a	Mzf1	2610201A13Rik	Chrdl1
Fli1	Pcdhb21	Lce1i	2610201A13Rik	Hoxc13
Atp6v0a4	Hypm	Ppp3cc	Samhd1	Cdcp1
Zfand6	Prdm1	Adat1	Prss58	Sptlc3
Gjc3	Pcdhb3	1110006O24Rik	Prss29	Fn1
Inhba	Cldn16	Gm10228	Gpank1	Slc4a4
Cbx2	1700011A15Rik	5830411J07Rik	[]	Ptprc
Akr1c20	Orm2	Tsc22d3	[]	Stxbp6
Zbtb20	Ppy	Hdhd1a	[]	Pstpip2
Gstm6	Slc16a8	Ahrr	[]	Efna5
Ky	Sult1e1	Hdac7	Clec4e	Ppl
Nfya	Krtap5-2	Gdi2	Txndc8	A130010J15Rik
Bmx	Crygs	Ywhag	Slc12a8	LOC100045677 /// Mcm3
Stat5b	Gcm2	Ywhaq	Adamtsl5	F3
Spn	Il1rapl2	Vamp2	Gbx2	Ror1
LOC102643033	Gm2083///Mup1	Ntrk2	Tmco5	4930562D21Rik
Sema5b	Mucl1	Plekha3	Gdap10	Tmem40
Foxo4	Cnnm3	Plekha3	Asb12	Gm428
Hmx1	Vtn	Sufu	Ptgfr	Bnc2
Hist1h3b///Hist1h3	Dab2	B3gnt2	Prss22	Gpr177
Itgb1bp1	Stxbp1	Lancl2	Lta	Rbm35b
H2-T24	Dkk2	Zbtb14	Grk4	Angptl2
Siglec1	Tmem47	Gm21540///Tme	Krtap13	Ccr5
Pou3f4	a	Gm21540///Tme	Spesp1	Hmga2
Bdnf	Ccdc50	Gucy1b3	Csn2	Klk7
Slc5a3	Ccdc50	Gm4887///Twf1	Sntb2	Lipg
2810408B13Rik	Dcbld2	Sep-06	Gna14	Tslp
Pdx1	Dpf1	Ywhab	Prss12	Dyrk3
Tpsb2	Gucy1a3	Acd	Ubl4b	Lad1
Hnf4g	Crybb2	Xbp1	Gp49a///Lilrb4	Hist1h4a /// Hist1h4b /// H
C5ar1	Chrdl2	Tgfbr1	Ramp3	Stard10
H2-Ob///LOC10263	Chn1	Rab18	Atp2b2	Cybb
Avpr1b	Epha5	Hk1	Peg12	Il2rb
B3galt1	Selp	St6galnac3	Abcc9	Tyrobp
Gnb5	Shox2	Il17ra	Krt35	B930041F14Rik
Foxh1	Chrne	Mfge8	Tnfsf10	Hist1h2ab /// Hist1h2ac //
Hand2	Slurp1	Usp9x	Sema3a	St6galnac3
Usp17lc///Usp17ld	AK009121	Timp2	Sema3a	Sirpa
Fgf7	Prkd3	St6gal1	Syt2	Pak6
[]	Chad	Hs6st2	Otof	LOC100038947 /// LOC668
Ccr5	Prl6a1	Rgs5	Pcdhb21	Vtn
P2ry4	Hoxd1	Rgs5	Tcl1b4	Dock10
Glra1	Aicda	Atrx	Prdm1	Ahr
Drd3	Cftr	Znrf1	Ager	Lamb3
Sstr4	4930429B21Rik	Son	Pcdhb3	Ahr
Ctsq	Zfp493	Add1	Cldn16	Ear2

Nog	Tsga13	Add1	Orm2	Dscam
Ftl1///Gm20746///	Cacng2	Fancg	Pcdhb19	Ret
Lcn4	Defa-rs2	Hapln2	Sult1e1	LOC100047803 /// Ntsr1
Flad1///Lenep	Raet1a///Raet1b	Enc1	Tac4	AA407782
Il1rl1	Mtag2	Enc1	Ripply3	Gpr177
Ntrk3	Rbm18	Nacc1	Il1rapl2	Nbl1
Actg2	Prkacb	Adcy7	Gm2083///Mup1///I	Adamtsl4
Olfr544	Ptp4a2	Man1a2	4933411G06Rik	Klk11
Magea6	Aes	Plxna3	Vtn	Spp2
Olfr672	Csn1s1	Angptl2	Klk1b16	Ppp2r2c
Tlm	Dnaja3	Mobp	Stxbp1	Chst12
Olfr68///Olfr69	Bscl2	Hsd17b11	Dkk2	Grhl2
Olfr71	Jmy	Srprb	Tmem47	Sgpp1
Vmn1r41	Sqrdl	Pitpnb	a	Ehf
Olfr151///Olfr160	Zfhx3	Pole3	Masp2	Spint2
Olfr1509	Zfhx3	Ighmbp2	Gpatch2	Cap2
Vmn1r13	Tgfb1	Aaed1	Crybb2	E2f2
Vmn1r-ps8///Vmn1	Itgb3bp	Mef2c	Chn1	Nup210
Hist1h1e	Doc2b	Mef2c	Alx3	Rpl7
Sprr3	Lce1a1	Arhgef2	Selp	Dsg1b
H2-D4	Cts8	Mrc2	Shox2	Icos
Ferd3l	Acox3	Kif1a	Chrne	Il1f9
Magee1	Sgce	Jak2	Insrr	Chst2
Col5a2	Fgf10	Jak2	AK009121	Tuba4a
Tff2	Il2ra	Cyp7b1	Stra8	Gcet2
Rcn2	Gpx5	Nr4a3	Aicda	4921521F21Rik
Tcl1	Clec7a	Banf1	Gpr143	Csf2
Bnip3	Folr4	Banf1	Nxf2	Kif13a
Cdk20	Csf2ra	Banf1	Zfp493	Stxbp6
Mageh1	Pparg	Gpc4	Tpo	Ptgds2
Syt5	Cd247	Epb4.1l1	Mtag2	Pscdbp
Tnni3	Tex15	Zbtb33	Csn1s1	Txndc5
Extl2	Tmlhe	Trpc1	8430419L09Rik	H2-Ab1 /// Rmcs2 /// Rmc
Fbln1	Tmlhe	Bhlhe41	Bscl2	Lrrc8e
Tbx2	Cab39l	Gm6910///Rybp	Sqrdl	Tcfap2e
Arl2	Cntn3	Nkx2-2	Sec61a2	AU018091
Map6	Lce1i	Rtn4	Tgfb1	Ovol1
Pten	Ppp3cc	Cdk5r1	Procr	Hic1
Adamts5	Gm10228	Strbp	Doc2b	Map3k5
Rrad	Dtx3	Pvrl3	Lce1a1	Sirpa
Cr1l	Chrnd	Edn3	Acox3	Gfra2
Mxd4	Klk4	Atn1	Sgce	Thbs2
Rab3a	Socs7	Gnao1	Fgf10	Cd37
Ap3s1	Dhx58	Loxl4	Il2ra	Copz2
Nkain4	Dhx58	Hcn2	Gpx5	Gpc6 /// LOC100045283
Rasgrf1	Tsc22d3	Atp11a	Clec7a	4933417O08Rik
Wnt5b	Hdhd1a	Irf4	Folr4	Dsg2
Etv1///Gm5454	Tas1r3	Myt1l	Csf2ra	Rab3d
Igf2bp3	Etos1	Pbx3	Csf2ra	Fcer1g
Igf2bp3	Trpd52l3	Itgav	Srcin1	Tac1
Wdr54	Gm10256///Gm1	Nudt16	Dao	Cercam
Gm10058///Gm10(	4933436I01Rik	Abcc6	Hpn	Fermt3
Ppap2a	Speer4f	Ids	Lbx2	Itm2a
Ppap2a	Otog	Bco2	Tex15	Cdca8
Ly6h	Pcdha1///Pcdha1	Slc4a4	Tmlhe	AI662270
Mmp16	Npas1	Rnf144a	Tcp11	Cd207
1700020D05Rik	Il13	Cabp1	Gabrr2	1810019J16Rik
Vsig2	Clec4d	Ugcg	Pmfbp1	C1s /// LOC100044326
Pcdhb9	Fgf16	Dst	Lce1i	Ccnd2
Hfe	Hdac7	Spta1	Ppp3cc	Adh7
Sgca	Vamp2	Gabra1	Gm10228	Rprm
Plcz1	Ntrk2	Gabra1	Dtx3	Shmt1
Naalad2	Plekha3	Bmp5	5830411J07Rik	LOC640441 /// Thbs1
Prkd1	Eln	Pecam1	1700019G17Rik	Arg2
Toporsl	Itga9	Cacna1c	Sprr2d	Dpp4
Zap70	Gucy1b3	Lef1	Tsc22d3	Chrdl1
Pmepa1	Sep-06	Rabep1	Tas1r3	Rarres2
Cacna1h	Nsd1	Mgmt	Gm10256///Gm1035	Cit
Pnck	Sln	Pcdh10	4930433N12Rik	Arhgap9
Ddr2	Xbp1	Cttn	Ahrr	Eln
Phka1	Wnt7b	Pla2g2f	Pcdha1///Pcdha10//	Ccnd2
Bicd2	Tgfbr1	Cend1	Ntsr1	Cd200r3
Zeb2	St6galnac3	Cend1	Clec4d	Tuba4a
Mapre1	Vegfa	Tgm3	Myl10	Creb5
Rad51d	Ppap2c	Tex9	Hdac7	A630033H20Rik
Myt1	Mfge8	Frk	Hdac7	Sh3d19
Smpd3	Cd151	Clec5a	Ntrk2	Lgals12
Slc43a3	St6gal1	Fxyd1	Ntrk2	5830405N20Rik
Pafah1b2	Srrm1	S100a6	Plekha3	Vash1
Cacng1	Rgs5	Prlr	Eln	LOC100041195 /// LOC10(
Cartpt	Atrx	Slc12a1	Itga9	Lbh /// LOC100048380
Igfals	Atrx	Vipr2	Gm21540///Tmed2	1100001G20Rik
Fbn2	Add1	Pi15	Gucy1b3	Capns2
Neurog2	Apc	Zim1	Gucy1b3	Lama5
Agpat6	Enc1	Pstpip2	Sep-06	Svopl
Trip4	Slc25a15	Ccr10	Nsd1	Dsc3
Col13a1	Adcy7	Sugct	Sln	Lbh
Hoxc4	Polh	Hhip	Sez6	Il13
Prg2	4933411K20Rik	Arl10	Xbp1	Rassf5
Ctbp2	Ankrd1	Zfhx4	Bcl2l1	Gfra2
Pcdh18	Cnot4	Angpt1	Wnt7b	Btk
Pcdh18	Cntrl	Pgr	Tgfbr1	Slc6a14
H2-Ea-ps	Hsd17b11	Lrp8	Rab18	Kbtbd10
Slc22a12	Nrg3	Lepre1	St6galnac3	Med12l
Fmo2	Mef2c	Nkx3-2	Il17ra	Slc35c2
Abcg2	Mef2c	Wnt2b	Il17ra	Gja1
Cbln3	Magi3	Stat5a	Vegfa	Mchr1
Bmp4	Npas2	Krtap4-2	Ppap2c	Camk2n2
Fgf3	Mip	Sp4	Usp9x	Ms4a4d
Tnfsf9	Arhgef2	Hoxd3///Hoxd4	St6gal1	Pde7b
Mc2r	Arhgef2	Zic4	Mapk8	Ccr5
Bcl2	Mrc2	Fbxo4	Rgs5	Hmga1 /// Hmga1-rs1

Kif5c	Lats2///Xpo4	Syne1	Rgs5	Ppap2b
Hist1h4a///Hist1h4	Alg2	Pcdhb15	Znrf1	Stxbp2
Pcdhb4	Snrpn///Snurf	Adrb3	Asph	Gfra2
Syt17	Jak2	Cntnap1	Adcy7	B930041F14Rik
D1Pas1	Phf2	Ncam2	Arid5b	Tmed3
Rad23a	Vhl	Ncam2	Arid5b	Ccnd2
Agtrap	Sertad3	H2afy2///H2afy3	Man1a2	Cnnm1
Ar	Tcf23	Rapgef4	B3gnt5	Cobl
Met	Gpc4	Zfy1///Zfy2	Etv5	LOC381765 /// LOC66550
Bmf	Trpc1	Psg17	Car6	Espl1
Dgke	Trpc1	Cd1d2	Rsad2	Acpp
Spred2	Stap1	Tusc3	Mobp	Klc3
Pag1	Ldb2	Samt4	Ranbp17	Loh11cr2a
Sec1	Bmp2k	Itpr2	Gna12	Mcpt-ps1
Pcdhb6	Gbp4///Gbp8	Cdkn1a	Mef2c	Dock8
Gypa	Cml2	Krtap19-5	Mef2c	4930570C03Rik
Gjd2	Strbp	Agrp	Kcnn4	Cdh4
Tpm1	Ryr2	Vcan	Mrc2	Cdkn1c
Nsg1	Zrsr2	Zfp92	Kif1a	Il1rl1
Igfbp3	Pvrl3	Vmn1r171	Dnase1l3	2310038H17Rik
6720475J19Rik	Pvrl3	Eya1	Alg2	Saa3
6720475J19Rik	Edn3	Nr5a1	Mllt1	Nova1
Cmpk1	Atn1	Tpst1	Snrpn///Snurf	Cpne4
Gpm6b	Gnao1	Cyp3a16	Jak2	Lamc2
Incenp	Hcn2	Fbxo17	Jak2	Tgfbi
Col1a2	Dsc2	Lhx3	Ptger4	Crxos1
St6galnac6	Myt1l	Fcer1a	Per3	AA409316
Aip	Npcd///Nptxr	Upk2	Per3	Cited2
Lipa	Clec1b	[]	Gpc4	Pilra
Hes5	Mettl7a1	Egfbp2	Slc7a10	Ifng
Dnajb11	Ccr2	Kcnab3	Zbtb33	Man2b1
Cfh	Ccr2	Efna5	Trpc1	Il7r
Sri	Gabrb3	Defb5	Stap1	Edar
Spred1	Gopc	Epdr1	Ldb2	Fzd5
Spred1	Itga4	Gsr	Vamp3	9530053H05Rik
Taf7	Chrna4	Set	Mtdh	Slc16a12
Cetn1	Chrna4	Ppp2cb	Nkx2-2	Cdh3
Phc1	Nudt16	Ppp2cb	Cdk5r1	Rbm47
Cap2	LOC102638292	Bace1	Ryr2	Vapb
B4galt6	Bche	Ak4	Pvrl3	Gpr177
Nrgn	Fip1l1	Pip5k1b	Pvrl3	Mpl
Src	Hnf1a	Adat3///Scamp4	Areg	Krt28
Tgfb2	Ptprt	Il1rap	Zfp386	Vkorc1
Mpz	Il6st	Map1b	Tial1	Hopx
1500015O10Rik	Rnf144a	Kcnk5	Gnao1	Mcm2
Coch	Zfp40	Fgl2	Dsc2	Tmem16j
Hyou1	Ugcg	Fgl2	Cgnl1	Tgfbi
Prx	Socs4	Clstn1	Rfng	Lif
Bbc3	Gabra1	Nr3c1	Atp11a	Sell
Pnn	Pik3ap1	Sh3bgrl	Abcg3	P2rx1
Rpl39l	Atp4a	Rab24	Myt1l	Gna14
Gdap1	F2rl3	Mrps23	Mgat4c	Plxna4
Pvrl3	Cacna1c	Elavl2	Ttyh2	6720458F09Rik
Sdcbp	Lef1	Sos1	Higd1c///Mettl7a1//	Pcdh10
Ergic1	Zic2	Sos1	Mettl7a1	Col12a1
Sec23a	Pcdh10	Aplp2	Ccr2	Ebf2
Crispld1	Cttn	Aplp2	Itga4	B230120H23Rik
Crispld1	Lce1g	Aplp2	Ptpn11	Ddah1
Cacna1g	Mynn	St3gal2	Itgav	Bace2
Laptm4a	Egfl7	St3gal2	Nudt16	Afap1
Nfatc4	Extl3	Elk1	Ids	2310010M24Rik
Nfatc4	Kcns2	Eif2ak1	Ttll3	Nxph1
Agt	Cend1	Med29	Bco2	Egfr
Ugt2b38	Pde7b	Pdgfra	Slc4a4	Itm2a
Etv6	Tex9	Pdgfra	Pknox1	Fosl1
Timp4	4930549C01Rik	Anp32a	Esr1	Cxadr
Fbln2	Mab21l1	Sh3bp5	Pax9	Egfr
Ptgs1	Fxyd1	Sh3bp5	Slc39a9	Ace2
Pdia3	S100a6	Mapk11	Btnl10	2610301F02Rik
Zic3	Zfp354b	[]	Ugcg	Tnfaip8
Ror2	Insr	Crp	Bmp5	2310067E19Rik
Gsta3	Slc12a1	Crkl	Pik3ap1	Vgll3
Slc46a2	Vipr2	L1cam	F2rl3	Cxcl14
Zic1	Grin2d	Adcy3	Lef1	Car13
Bicc1	Pi15	Nipa2	Adarb2	Fn3k
Sncaip	Sema6a	Mmp19	Pcdh10	Arhgap9
Brd2	Elac1	Hnf4a	Cttn	Trem1
Jam3	Sugct	Stc1	Ptgfrn	Osm
Tagln	Rcan2	Papss2	Smyd1	C030019I05Rik
Nnat	Hhip	Igfbp4	Hs3st3b1	C86090
Iapp	Slc5a7	Igfbp4	Map3k5	Chchd6
Nid2	Ptrf	Hs1bp3	Axin2	Cmah
Aass	Arl10	Spry4	[]	Il1rn
Arid3b	Zfhx4	Fli1	Prkg2	Ccbe1
G6pc2	Dnah5	Ets1	Tex9	Cd3e
Stk32c	Pcdhb14	Dlx3	Grk1	Vgll3
Strn3	Angpt1	Tnfrsf22	Frk	Ptgfr
Rbms2	Flt4	Gjc3	Mab21l1	Car3
Swap70	Gpr110	Tsc1	Il1f5	Minpp1
Zfp207	Slc26a3	Ndst1	Fxyd1	H2-Aa
Ggcx	Onecut1	Inhba	Traf6	Amigo1
Ifi44	Ralgapa1	Pex5	Traf6	Snhg7
Kcnc1	Zfp108	Zbtb20	Col9a1	Ptgfr
Gatm	Samsn1	Klrb1b	Lyst	Csf1
Abcg1	Lrp8	Bmx	Kremen1	Esr1
Ankrd36	Mpl	Stat5b	Slc12a1	Ndufb4
Igfbp7	Lepre1	Slc5a5	Vipr2	Irf4
Igfbp7	Runx3	Htr2b	Grin2d	Adi1
Zfpm1	Stat5a	Dhh	Xiap	Col25a1
Itm2a	Cant1	Mgat3	Kcnmb1	
Alpl	Zfp318	Foxo4	[]	
Lrrc8c	Zfp318	Hist1h3b///Hist1	Msi1	

Rasd1	Ttbk2	Sh3pxd2a	Elac1
Pcdhga1///Pcdhga1	Rabgap1l	Pou3f4	Ccr10
Cygb	2010106E10Rik	Sema5a	Rcan2
Gpr146	Acer2	Bdnf	Zfhx4
Bmp2	Olfr78	Slc5a3	Grid2
Zdhhc3	Sorcs1	Mmp1a	Pcdhb14
Col1a1	Naip5	Ak1	Wnt8b
Tmem246	Pde4a	Htr5a	Angpt1
Fads1	Kcnd1	B3gnt3	Slc26a3
Aars	Zic4	Npffr2	Prkcg
Gpsm1	Mef2b	Hand2	Onecut1
Gpsm1	Fbxo4	Cyp2a4///Cyp2a5	Ralgapa1
Ak3	Syne1	Htr7	Crb1
Zwint	Cd180	Fgf7	Jph2
Bambi	Pcdhb2	Irs4	Samsn1
Igfbp4	Epm2a	Ccr5	Lepre1
Athl1	T2	Klf9	Runx3
Unc93b1	Cd209c	A030005K14Rik	Stat5a
Ddx41	Serpina3c	Sike1	Ugt2a1///Ugt2a2
Cxx1a///Cxx1b	Pet2	Nog	Egr3
Zfp503	Grid1	Ftl1///Gm20746,	Rabgap1l
Rasl11b	4930524B15Rik	Lcn4	Prpmp5
Slc44a1	Evx2	Ifnb1	Hpgds
Gypc	Cntnap1	Neurog3	Acer2
D030056L22Rik	Kcnj6	G6pd2///G6pdx	2010204K13Rik
Lamc1	Ncam2	Gja10	Mixl1
Celf2	Ncam2	Ntrk3	Cep250
Rnf187	Fam184b	Olfr155	Epha6
Tmem176a	Shbg	Fpr-rs3	Txnrd1
Aspscr1	Ceacam2	Magea6	Dfna5
Olfml2b	H2afy2///H2afy3	Olfr672	Pde4a
Pkig	Myo1f	Vmn1r40	Hoxd3///Hoxd4
Kcnab3	Il5ra	[]	Zic4
C3	Zfy1///Zfy2	Vmn1r25	Syne1
Cers2	Pcdhb1	Vmn1r13	Pcdhb2
Ropn1l	Hapln1	Vmn1r15	Pcdhb15
Meis3	Capn8	Rab23	Trim34a///Trim34b
Gdf10	Tbc1d8b	Hes3	Ifi202b
Gm3470///Rbpms2	Cysltr2	Defa4	Adrb3
Mfap4	Cemip	Magee1	Serpina3c
Arl6ip6	Htr3b	Col5a2	Grid1
Ginm1	Psg18	Ins1	Ly6c1///Ly6c2
Ginm1	Adra1a	Rcn2	Hif3a
Tspyl4	Scn9a	Tcl1	Rap2a
Hvcn1	Fpr3	Ppt1	Cntnap1
C1s1	Hsd17b3	Acss2	Mrgprh
Fgfr1	[]	Acss2	Ncam2
Col4a2	Cdkn1a	Bnip2	Ncam2
Manbal	Nrg4	Cdk20	Trim26
Urm1	Gm3417///Gm34	Mageh1	Gsx2
Oaf	Clec4b1	Aebp1	Vgll1
Tcf4	Krtap21-1	Fibp	Kcnh3
Gpx8	Vcan	Znrd1	Tmc1
Cend1	Pkhd1l1	Casq2	Zfy1///Zfy2
Jkamp	Tex16	Cyp51	Hapln1
Igf2r	Vmn2r10	Id2	Tbc1d8b
Lamb1	Zfp92	Extl2	1700082M22Rik
Lamb1	Sele	Fbln1	Ikzf2
Kcnk13	Vmn1r58	Arl2	Cd1d2
Ptrf	Pcdhb18	Map6	Scn9a
Col6a3	Eya1	Pten	Il17a
Tmem98	Tpst1	Gamt	[]
Fabp4	St8sia5	Mcg1038069///U	Cdkn1a
Ccdc80	Matk	Adamts5	Msmb
Ccdc80	Fbxo17	Nfic	Cacna1e
Tmem41a	Kcnj5	Atxn10	Vcan
Parm1	Bmp10	Rab3b	Strc
Asb8	Adk	Skiv2l	Zfp59
Meox2	Homer1	Rab3a	Zfp92
Meox2	Olfr74	Ap3s1	Sele
Fam65a	Upk2	Nkain4	Vmn1r58
Ces2c///Ces2d-ps	Uevld	Rasgrf1	Arnt
Ifitm1	Kcnab3	Wnt5b	Vmn1r171
Slc12a1	Fgf11	Ppp1r1a	Ap4b1
Aif1l	Klrc3	Etv1///Gm5454	Eya1
Dclk1	Uts2	Arpp19	Nr5a1
Dclk1	Epdr1	Ppap2a	Matk
Tmem55a	Bcl6	Ppap2a	Fbxo17
Esco1	Ppp2cb	Nos3	Lhx3
Tspan17	Bace1	Mmp16	Bmp10
Snx15	Abca1	1700020D05Rik	Vmn1r172
Pcdha1///Pcdha10,	Golph3	Pcdhb9	Pax5
Lpgat1	Map1b	Hfe	Lmx1b
Lpgat1	Fgl2	Slc7a2	Etv2
Ube2e2	Fgl2	Adipoq	Upk2
Ppapdc3	S100a3	Ptch2	Ang3
Mycbpap	Nr3c1	Serpinb9b	Fgf11
Psmf1	Nr3c1	Naalad2	Uts2
Psmf1	Sh3bgrl	Prkd1	Defb3
Psmf1	Sos1	Crygb///Crygc	Defb5
Armcx3	Sos1	Ctr9	Psap
Rcn3	St3gal2	Ttyh1	Bcl6
Selm	St3gal2	Ttyh1	Bace1
Asrgl1	St3gal2	Pmepa1	Twsg1
Cbx6	Tgs1	Pmepa1	Il1rap
Spon1	Tcf20	Cacna1h	Il1rap
Csgalnact2	Mrpl19	Pnck	Map1b
Msrb2	Pdgfra	Acp1	Kcnk5
Abcg4	Pdgfra	Ap3s2	Fgl2
Mrps6	Ccr9	Pcdhb10	Fgl2
Tm6sf1	Serpina3m	Bbx	Nr3c1
Gprc5c	Sh3bp5	Phka1	Pnlip

Acaa1b	Sh3bp5	Zeb2	Trim44
Lpcat1	Tbl2	Zmynd10	Sh3bgrl
Phldb1	Camk4	Chst2	Rab24
Phldb1	Lhx1	Gipc1	Rab24
Camkk2	Capn6	Syncrip	St3gal2
Camkk2	Crkl	Smad6	St3gal2
Camkk2	Nedd4	1700088E04Rik	St3gal2
Cst8	L1cam	Tlr3	Mr1
Angptl3	Dnajb5	Slc43a3	Pdgfra
Txnrd1	Gria2	Pafah1b2	Pdgfra
Txnrd1	Mmp19	Pafah1b2	Ccr9
Zfp428	Phex	Prep	Serpina3m
Ugt3a1	Kcnc3	Prrc2a	Sh3bp5
Atl3	Hnf4a	Arf5	Sh3bp5
Oit1	Papss2	Zfp191	Tbl2
Zfp768	Papss2	Fbn2	Camk4
Dram1	Igfbp4	Kcnd2	Pcyt1a
Mmd2	Hs1bp3	Kcnd2	Adcy3
Oas1e	Tfap2a	Agpat6	Adcy3
S100a4	Mxd1	Rbp2	Dnaaf1
Nck1	Cxcr5	Trip4	Add2
Zfyve27	D730048I06Rik	Col13a1	Mmp19
Pycr1	Clec4a2	Hoxc4	Mmp19
Slc25a4	Foxp2	Pcdhb12	Kcnc3
Slc25a4	Spry4	Syt12	Kcnc3
Stac2	Zfp319	Snrpd3	Papss2
Srgap3	Fli1	Ctbp2	Igfbp4
Samd4	Atp6v0a4	Rnf5	Igfbp4
Lmcd1	Strn	Pcdh18	Akr1c12
Fgl1	Dlx3	Pcdh18	D730048I06Rik
Aoc1	Tnfrsf22	Sfmbt1	Crhr2
Sumf1	Sema7a	Slc22a12	Spry4
Gprc5b	Gjc3	Bmp4	Spry4
Tceal1	Cdh8	Tnfsf9	Fli1
Ift27	Inhba	Mc2r	Ets1
Pdia5	Mid1	Bcl2	Cntf///Zfp91///Zfp91Cntf
Acp2	Ntn3	Galr2	Tnfrsf22
Acp2	Nodal	Kif5c	Gjc3
Slit2	Nodal	Pcdhb4	Ndst1
Crtc2	Zbtb20	Sapcd1	Itgam
Plekhf1	Klrb1b	Zfp60	Cdh5
Fam26e	Mc1r	Syt17	Cdh8
Cacfd1	Cdx2	Rnf114	Inhba
Cacfd1	Acot4	Srd5a2	Ntn3
Gca	Acot4	Sprr2i	Akr1c20
Tmem38b	Il7	Rad23a	Pex5
Runx2	Ky	Agtrap	Zbtb20
Calml4	Bmx	Ip6k1	Il7
Tmem150a	Mycl	Kat2a	Ky
Ccr5	Mycl	Mme	Nfya
Slc15a2	Stat5b	Cybb	Pfkfb2
Rasgrf1	Aff2	Ar	Cmpk2
Ap5m1	Eaf2	Alyref///Alyref2	4933434I20Rik
Galnt11	Fcer2a	Art2a-ps	Acvr1b
Ms4a7	Apc2	Mecom	Oprl1
BC018242	Mgat3	Ifna2	Stat5b
Rsph9	Hist1h3b///Hist1	Stt3a	Stat5b
Sez6l	Clstn2	Stx6	Erf
Cdh22	H2-T24	Ncbp2	Aff2
Cald1	Siglec1	Ncbp2	Sync
Cald1	Pou3f4	Tpm1	Eaf2
Cald1	Sema5a	Hnrnpu	Fcer2a
Reep3	Bdnf	Arf4	Htr2b
Reep3	Slc5a3	Igfbp3	LOC102643033
Reep3	2810408B13Rik	Cdk5rap3	Plau
Pitx2	Pdx1	Msmo1	Csprs///Gm15433///Gm2666///Gm7609///LOC
Lama4	Hnf4g	Derl1	Akap7
Lama4	Ak1	B3galt2	Inhbe
Tasp1	B3galt1	Prickle4///Tomm	Pla2g6
Alg12	B3gnt3	Gpm6b	Hmx1
Slain1	Hand2	Paqr4	Itgb1bp1
Ncmap	Grin2b	Col1a2	Clstn2
Mef2c	I54	Ube2d3	Sh3pxd2a
Ptprd	Klf12	St6galnac6	Pou3f4
Rab13	Usp17lc///Usp17	Impa1	Sema5a
Kdm5d	Mc3r	Aip	Slc5a3
Kif12	Hoxd13	Cwc15	Fgf23
Wipi1	Fgf7	Lipa	Tpsb2
Bst2	Mrvi1	Dnajb11	Hnf4g
Gdap1l1	Irs4	Cfh	Ak1
Steap1	Map3k2	Gnpnat1	C5ar1
BC021614	[]	Spred1	B3galt1
Pip5k1c	Kcna4	Spred1	Gnb5
Car8	Chrm3	Gpr137b///Gpr1	Foxd3
Upp2	Msh3	Mta2	Foxh1
Purg	Lhx6	Cd36	Hand2
Cyp3a25	Klf9	Lgi1	Grin2b
Ppp1r42	Mycs	Tiam2	Ttc39d
Odf4	Foxb2	Tmem167	[]
Glyctk	Il6ra	Phc1	Fgf7
Tcte2	Mmp1b	Spcs2	Pkdrej
Tpm2	Drd3	Mrpl49	Kcna4
Dnmt3l	Gzmk	Cap2	Ccr5
Bpifb5	Otp	Tctn2	Klf9
Ppp6r2	Phxr2	B4galt6	Phxr4
Ppp6r2	Xcr1	Cebpd	Mmp1b
Pbld1	Mecom	Tgfb2	Gja8
Wasf3	Krtap28-13	Mpz	Glra1
Ntrk3	Ftl1///Gm20746,	1500015O10Rik	Drd3
Ntrk3	Ftl1///Gm20746,	Minpp1	Sstr4
Skp2	Lcn4	Nedd4l	Nkx2-9
Gatad2b	Igfbp5	Ints6	Ctsq

Tm6sf2	[]	Slc34a1	Pfdn5
Zfp286	Lbx1	Stmn2	Lcn4
Zfp286	Ntrk3	Coch	Flad1///Lenep
Kcnc4	Ifna11	Hyou1	Igfbp5
Wars	Cyp21a1	Mest	Clcn6
Rnf183	Gja3	Tmem39a	Gp1ba
Ncam1	Nkx2-1	Myadm	Il1rl1
Ptpn5	Npy6r	Pnn	Sf1
Klk1b11	Olfr544	Entpd1	Lbx1
Acnat2	Olfr672	Rpl39l	G6pd2///G6pdx
Gbp7	Tlm	Pvrl3	Gja10
Golt1a	Olfr17	Sdcbp	Ntrk3
Rorb	Olfr66	Ergic1	Actg2
C1ql3	Vmn1r50	Ergic1	Nkx2-1
Sgip1	Vmn1r44	Orc4	Pou4f3
Sgip1	Olfr151///Olfr16	Cspg4	Olfr544
Lmbrd1	Olfr1509	Fzd8	Olfr690
Phospho2	Vmn1r29	Socs5	Olfr68///Olfr69
Lbh	Tas2r105	Crispld1	Olfr49
Slc22a29	Vmn1r-ps8///Vm	Crispld1	Olfr15
2210407C18Rik	Sprr3	Lipt2	Vmn1r41
Krtap6-5	H2-D4	Sort1	Olfr151///Olfr160
Theg	Ifna4	Cacna1g	[]
Acot5	Ferd3l	Laptm4a	Tas2r105
Pear1	Ear3	Csnk1g2	Rab23
Pear1	Defa4	Nfatc4	Ifna1///Ifna12///Ifna15///Ifna16///Ifna5///Ifna
Psmc3ip	Magee1	Nfatc4	Ferd3l
Crygn	2210010C04Rik	Akr1cl	Ear1///Ear12///Ear2///Ear3
Zcchc18	Col5a2	Ap1g1	Magee1
Rgs3	Tff2	Tsnax	2210010C04Rik
Ncam2	Rcn2	Agt	2210010C04Rik
Prima1	Tcl1	Gkn1	Col5a2
BC027072	Acss2	Fbln2	Ins1
Trim31	Acss2	Chtop	Rcn2
Col26a1	Mageh1	Asb4	Tcl1
Tmem116	Pate4	Pdia3	Mad2l1
Gm13271///Gm132	Prph	Zic3	Acss2
Cd8a	Syt5	Ror2	Cdk20
Srprb	Cyp51	Gsta3	Mageh1
Myef2	Extl2	Pck1	Ctdspl
Tdpoz1	Ptprm	Dapk3	Aebp1
Hgf	Ropn1	Rab11b	Cyp51
Adam26a	Tbx2	Hs3st1	Extl2
Cpsf4l	Map6	Slc35a5	Fbln1
Dnm3	Zkscan3	Knstrn	Ptprm
2610034M16Rik	Pten	Cnnm2	Tbx2
Nlrp3	Adamts5	Zic1	Rprm
Slc1a1	Rrad	Bicc1	Pten
Glis1	Fam129a	Nfix	Adamts5
Wif1	Mxd4	Igdcc3	Rrad
Mef2a	Myl4	Sncaip	Nfic
Krtap16-3	Lep	Jam3	Thbs2
Msr1	Rab3b	Tagln	Ampd3
[]	Odf1	Nnat	Ap3s1
Slc41a3	Rab3a	Sirt2	Rasgrf1
Oscar	Ap3s1	AW209491	Serpinb9
Klra12	Nkain4	Nid2	Wnt5b
Il12a	Rasgrf1	Csk	Etv1///Gm5454
Churc1	Wnt5b	Aass	Igf2bp3
Grb10	Etv1///Gm5454	Ccer1	Ly6h
Plp1	Igf2bp3	Bcas3	Mmp16
[]	Ppap2a	Strn3	1700020D05Rik
Slu7	Ppap2a	Rbms2	Vsig2
Slu7	Ly6h	Swap70	Calcb
Mylk	Mmp16	Zfp207	Pcdhb9
Mark1	1700020D05Rik	Ergic3	Hfe
Pik3r1	Pcdhb9	Slc1a4	Slc7a2
P2rx4	Dok5	Leprotl1	Cntn6
Prrx1	Sh3bgr	Dnajb3	Sgca
Prrx1	Hfe	Ggcx	Ptch2
Bin1	Cryga	Hsph1	Plcz1
Otc	Plcz1	Gatm	Camk2d
Plekha5	Serpinb9b	Srd5a3	Naalad2
Plekha5	Naalad2	Fhl4	Ctr9
Lst1	Prkd1	Dmrt1	Ctr9
Pcdh10	Ctr9	Igfbp7	Toporsl
Epha3	Irak1bp1	Igfbp7	Sptlc1
Epha3	Pmepa1	Axl	Ttyh1
Galnt7	Cacna1h	Exosc10	Zap70
[]	Pnck	Ednrb	Pmepa1
Mlph	Stra6	Itm2a	Pmepa1
Lamc3	Mak	Alpl	Cacna1h
AI317395	Pcdhb10	Lrrc8c	Isl1
Plcb1	Ddr2	Rasd1	Stra6
Tmem176a	[]	Pcdhga1///Pcdhg	Speer4a
Ncf1	Bbx	Cygb	Inha
Trim35	Phka1	Bmp2	Ddr2
Edil3	Zeb2	Aco1	Tnfrsf21
Epm2aip1	Zmynd10	Zdhhc3	Phka1
Nol6	Tle1	Pdia6	Phka1
Gypa	Chst2	Cdipt	Bicd2
Lepr	Padi4	Col1a1	Zeb2
Nmrk1	Smad6	Ttc30b	Tle1
Trim60	1700088E04Rik	Ldoc1l	Slc5a4b
Slc39a14	Slc43a3	Usp1	Bysl
Rbpms	Nppc	Tmem246	Tlr3
Cdadc1	Prrc2a	Fads1	Slc43a3
Cdadc1	Rims2	Cmtr1	Aldh1a2
Klhl20	Cacng1	Aars	Ing3
Rfxank	Mutyh	Gpsm1	Cartpt
Akr1b10	Lipe	Gpsm1	Igfals
Akr1b10	Igfals	Psmd6	Drd4

B3gat1	Drd4	H1f0	Kcnd2
Tbx5	Fbn2	Pla2g15	Kcnd2
Nxnl1	Kcnd2	Pgd	Pabpn1
Rgs3	Agpat6	Farsb	Col13a1
Enpp5	Hmga2	Dlst	Gda
BC025446	Col13a1	Abcb8	Hoxc4
Ppp2r5c	Pcdhb12	Ak3	Kcnj12
Cldn19	Clk4	Tpm1	Bmpr1b
Suv420h1	Ctbp2	Zwint	Prg2
Stxbp6	Pcdh18	Cnih1	Capn9
Impg1	Pcdh18	Snap47	Sypl
Pitpnm1	Slc22a12	Txndc5	Clk4
A730028G07Rik	Ly86	Bambi	Ctbp2
Tc2n	Cbln3	Igfbp4	Pcdh18
Fcrla	Bmp4	Igfbp4	Pcdh18
Usp12	Gast	Mettl13	H2-Ea-ps
Med21	Tnfsf9	Athl1	Slc22a12
Myocd	Acot3	Prkcdbp	Ly86
Fabp4	Mc2r	Chchd6	Fmo2
Zfp287	Icam4	Nup133	Abcg2
Dtx1	Fzd5	Sf3a3	Cbln3
Eml6	Bcl2	Ddx41	Bmp4
Sorbs1	Kif5c	Cxx1a///Cxx1b	1810009J06Rik///Gm2663
Limk1	Hes7	Noc4l	Hrasls
Slc26a7	Ccr3	Fam49b	Tnfsf9
Nek6	Krtap5-4	Gga1	Mc2r
Prlr	Nat3	Zfp503	Bcl2
Cacna2d1	Rad23a	Coa3	Galr2
Psen2	Ip6k1	Rnf41	Hist1h4a///Hist1h4b///Hist1h4c///Hist1h4d///H
Lepr	Nt5e	Csdc2	Hist1h4a///Hist1h4b///Hist1h4c///Hist1h4d///H
Lepr	Mme	Mphosph6	Nos1
Kcnab2	Ar	Rasl11b	Pcdhb4
Wnt9a	Clip2	Abcf2	Fpr2
Fbn1	Esrrb	Serpina3k	Zfp60
Itsn1	Sgsh	Gypc	D1Pas1
Sema6a	Dmpk	D030056L22Rik	Srd5a2
Dnajc2	Setd7	Lamc1	Agtrap
Fgfr1	Vipr1	Telo2	Mxd3
Cep164	Espn	Celf2	Cybb
Spats2l	Art2a-ps	Hsbp1	Ar
Ifi44l	Sec1	Tmem176a	Ntn1
Atf5	Sacs	Aspscr1	Met
Xkr6	Adra2a	Hid1	Pikfyve
Mlx	Sh2d1b1///Sh2d	Olfml2b	Dgke
Arntl2	Stt3a	Mlf2	Pag1
B4galt4	Nsg1	Slc35b2	Setd7
Gpm6b	Igfbp3	Hpx	Vipr1
Rad51d	Dnmt3a	C3	Pim1
Slc25a30	B3galt2	Cers2	Sec1
Speg	Gpm6b	Wdr26	Syt7
Ros1	Col1a2	Cd99l2	Foxf1
[]	St6galnac6	Meis3	Pcdhb6
Myocd	Rilpl2	Abl1	Gypa
Masp1	Lipa	Pomk	Hspe1-ps2///Hspe1-ps2
Dcun1d1	Lipa	Gdf10	Adra2a
Kcnma1	Gtpbp4	Gm3470///Rbpm	Hes2
Nfatc2	Cfh	Mfap4	Aplnr
Kank2	BC005537	Dennd5a	Prosc
Slc6a5	Sri	Hint1	Arf4
Pign	Gnpnat1	Srsf7	Arf4
Oxtr	Spred1	Rora	Nsg1
Eomes	Spred1	Map7d1	Igfbp3
Ccar2	Spred1	C1s1	Dnmt3a
Tmpo	Taf7	Tmem5	6720475J19Rik
Dbn1	Tiam2	Tmem242	6720475J19Rik
Prpf6	Gabarapl2	Cyb5r1	Snx9
Serinc4	Pspc1	Fgfr1	B3galt2
Rgs16	Cetn1	Col4a2	Gpm6b
Tfap2a	Phc1	Tcf25	Incenp
Bcl2l1	Ms4a1	Btbd2	Incenp
Gem	B4galt6	Usp48	Col1a2
Trib3	Inpp5e	Manbal	St6galnac6
Kcnd3	Inpp5e	Rab1b	Aip
Cmklr1	Itgb1bp2	Tcta	Thy1
Cdc27	Src	Dcps	Lipa
Dio2	Nktr	Tbc1d13	Lipa
Fmr1	Tgfb2	Oaf	Dnajb11
Trim34a///Trim34b	Mpz	Tcf4	Cfh
Hrh4	Syt9	Gpx8	Spred1
Slc19a2	1500015O10Rik	Hnrnpl	Spred1
Rrbp1	Polg	Jkamp	Spred1
Prss44	Ints6	Glo1	Cd36
Park2	Stmn2	Igf2r	Tnfrsf13b
Klra21	Stmn2	Lamb1	Cetn1
Ackr4	Coch	Lamb1	Tmem167
Trdn	Prx	Ppp5c	Phc1
Gm21320///LOC10	Add3	Eya2	B4galt6
Cd209b	Add3	Ptrf	Etv4
[]	Hhex	Col6a3	Galnt1
Mup5	Pnn	Tmem98	Itgb1bp2
Lat2	Rpl39l	Gdpd5	Mpp1
Cd209a	Gdap1	Sall4	Cyp2c68
Cacna2d2	Pvrl3	Tax1bp3	Tgfb2
Dusp15	Cspg4	Ccdc80	1500015O10Rik
Igh-VJ558	Barx1	Ccdc80	Stmn2
Krtap19-4	Sec23a	Pigc	Stmn2
Tmem216	Socs5	Tmem41a	Coch
Ttc39c	Crispld1	Fam216a	Hyou1
Cmtm2a	Crispld1	Slc25a33	Prx
Kras	Sort1	Parm1	Add3
Bmp1	Cacna1g	Susd4	Pde7a
Mapre2	Wnt7a	Asb8	Tmem39a

Mapre2	Igfbpl1	Meox2	Rad18
Pros1	Nfatc4	Meox2	Hhex
Umod	Nfatc4	Rwdd4a	Myadm
Pacrgl	Akr1cl	Arhgap9	Rpl39l
Pank3	Ugt2b38	Ifitm1	Gdap1
Gm8539///Zbtb8os	Fbln2	Lmf1	Pvrl3
Ifih1	Rbm47	Slc12a1	Ergic1
Lama2	Smarcc1	Aif1l	Ergic1
Dimt1	Pdia3	Gm5526///Gm8	Crispld1
Hapln1	Zic3	Dclk1	Crispld1
Hapln1	Adcyap1	Dclk1	Cacna1g
Snx20	Ror2	Haus7	Laptm4a
B4galt7	Pck1	Apex2	Nfatc4
Maged2	Hs3st1	Esco1	Nfatc4
6330416G13Rik	Sema6c	Tspan17	Agt
Pdgfd	Ptbp2	Pcdha1///Pcdha1	Ugt2b38
Bcl2l11	Sep-02	Ube2m	Yaf2
Tead4	Zic1	Lpgat1	Kl
Prepl	Prkcb	Lpgat1	Timp4
Col4a1	Bicc1	Ppapdc3	Fbln2
H2afy2	Cript	Psmf1	Meig1
Arf1	Igdcc3	Psmf1	Ptgs1
Tgfbr2	Sncaip	Car11	Gpr83
Capns1	Jam3	Rcn3	Pdia3
Ttll1	Nnat	Selm	Zic3
Ddx3y	Scn8a	Asrgl1	Plbd2
Dhrs7	Nid2	Dhx36	Adcyap1
Gpm6a	Aass	Cbx6	Ror2
Plcl2	Bcas3	Ogfrl1	Rhox5
Rps6kl1	G6pc2	Spon1	Slc46a2
Tbp	Strn3	Tada1	Zic1
Klhl22	Gap43	Ino80e	Prkcb
Bfar	Ptpn5	Ubtd1	Bicc1
Tifa	Ifngr2	Msrb2	Igdcc3
Sccpdh	Kcnc1	Mrps6	Klhl10
Chst15	Nell2	Gprc5c	Sncaip
Gnaz	Prrt1	Acaa1b	Sox5
P4ha1	S1pr1	Pcyt1a	Jam3
Zfp553	Fhl4	Gprasp1	Tagln
Me2	Col11a2	Lpcat1	Nnat
Me2	Igfbp7	Ccdc58	Iapp
Add3	Igfbp7	Phldb1	Gykl1
Gnl2	Napsa	Camkk2	Nid2
Slc18a1	Ednrb	Camkk2	Npm3///Npm3-ps1
[]	Nek6	Angptl3	Arid3b
Ttf1	Atp8a1	Txnrd1	Bcas3
Tlcd1	Traf1	Zfp428	G6pc2
Qpct	Itm2a	Flywch2	Stk32c
Ano10	Alpl	Atl3	Rbms2
Sepn1	Lrrc8c	Utp6	Armc12
9030624J02Rik	Rasd1	Dram1	Swap70
AU040320	Cygb	S100a4	Slc1a4
Xylb	Nr2e3	Ppp1r8	Ggcx
Antxr2	Gpr146	Pycr1	Ifi44
Calm1///Calm2///C	Hrh2	Slc25a4	Akr1b7
Tdrd7	Tmem68	Polr3d	Prrt1
Cnn3	Col1a1	Polr3d	Abcg1
Des	Dner	Tspan2	Col11a2
Donson	Ldoc1l	Ddx46	Trpc4
Appl2	Fads1	Ift22	Ankrd36
Ckap4	Gpsm1	Stac2	Igfbp7
Ampd2	Gpsm1	Srgap3	Igfbp7
Meg3	Ak3	Samd4	Axl
Commd7	Cnih1	Ddx6	Ednrb
6330403K07Rik	Bambi	Xrcc4	Zfpm1
Msl3l2	Igfbp4	Sumf1	Lum
Pex5	Igfbp4	Gprc5b	Itm2a
Rab8b	Nup133	Cst12	Lrrc8c
Sep-08	Cxx1a///Cxx1b	Brca1	Bin1
Fmnl3	Zfp503	Tceal1	Rasd1
Sirt4	Rasl11b	Ift27	Pcdhga1///Pcdhga10///Pcdhga11///Pcdhga12/,
Nov	Wdr33	Pdia5	Cygb
Nov	Fam160b1	Eva1a	Gpr146
Inhbb	Gypc	Tspan15	Bmp2
Rbmx	Lamc1	Acp2	Galnt4
Ncam1	Apbb1	Slit2	Pdia6
Ncam1	Apbb1	Fam222b	Zdhhc14
Chst14	Celf2	Cux1	Col1a1
Srxn1	Pvr	Zfyve21	Fads1
Slc25a45	Hsbp1	Zfyve21	Gpsm1
Jmjd1c	Tmem176a	Exosc4	Gpsm1
Fndc3a	Aspscr1	Cacfd1	Enoph1
Mndal	Olfml2b	Cacfd1	Pgd
Dapk1	Celf1	L3hypdh	Abcb8
Ccdc93	Hpx	Fam210b	Ak3
Medag	1110008P14Rik	Gca	Gm15455///Rbm10
Col6a2	Cers2	Ccdc136	Bambi
Parp16	Palm	Tmem38b	Ifitm3
Uba7	Meis3	Runx2	Igfbp4
Gpr153	B230219D22Rik	Gm4024///Tmec	Athl1
Cubn	Gdf10	Gorasp2	Unc93b1
Fbxo2	Gm3470///Rbpm	Calml4	Tor2a
Nckap5l	Mfap4	Mis12	Nup133
Satb2	Pagr1a	Mapt	Vopp1
Scara3	Dennd5a	Tmem150a	Cxx1a///Cxx1b
Phyhipl	Ginm1	Rrp36	Hmgcs2
Myh4	Tspyl4	Tmem192	Gypc
Htra3	Rora	Ap5m1	D030056L22Rik
Ace	Fgfr1	Creb3	Celf2
Slc39a14	Col4a2	Creb3	Tmem176a
Penk	Manbal	Sds	Aspscr1
Mdfic	Tcta	Galnt11	Olfml2b

Amph	Oaf	Serpina10	Pkig
Stard8	Tcf4	Fam115c	Pdlim2
Ap2b1	Zfp868	Rsph9	Kcnab3
Mapre3	Gpx8	Sez6l	C3
A630072M18Rik	Igf2r	Cdh22	Cers2
[]	Lamb1	Cald1	Ropn1l
Maz	Lamb1	Cald1	Meis3
Metrn	Rnf8	Cald1	Pomk
Ttc19	Eya2	Reep3	Gdf10
Adamts10	Col6a3	Reep3	Gm3470///Rbpms2
Plekha6	Gprin1	Reep3	Mfap4
Armcx4	Sall4	Pbp2	Aqp9
Col14a1	Tmem38a	1700001O22Rik	Pagr1a
Ldlrad4	Ccdc80	Pitx2	Dennd5a
Ldlrad4	Ccdc80	Lama4	Mcfd2
Npr2	Pigc	Lama4	Hvcn1
Mettl20	Pigc	Slain1	Rora
Cep112	Tmem41a	Glycam1	C1s1
Ifnz	Smarca5	Gas5	Tmem242
Acsm2	Fundc1	Cep68	Fgfr1
Dock6	Meox2	Mef2c	Col4a2
Ddx4	Fam65a	Trim34a	Neil3
Vcan	Ces2c///Ces2d-p	Ptbp1	Scgb1b27
Vcan	Arhgap9	Ptprd	Oaf
Scn2a1	Ifitm1	Rab13	Tcf4
Malat1	Slc12a1	Gpr85	Nek9
Ddhd1	Aif1l	Gpr85	Gpx8
Sycp1	Gm5526///Gm88	Farsa	Cend1
Dnm3os///Mir214	Dclk1	Wipi1	Pttg1
Enpp3	Dclk1	Bst2	Nme1
Vps18	Stat3	Sec63	Igf2r
Ptgir	Tmem55a	Dnah8	Igf2r
Tmed7	Igj	Steap1	Lamb1
Akna	Slc25a19	Pip5k1c	Lamb1
2810474O19Rik	Esco1	Zfp418	Gm13770///Gm2573///Wdr83os
Aldh8a1	Pcdha1///Pcdha1	Cacul1	Kcnk13
Slc30a2	Lpgat1	Uroc1	Ptrf
Sult1a1	Lpgat1	Klrc1	Col6a3
Zfp931	Wfdc2	Stx18	Tmem98
Dapk1	Ube2e2	Ephb2	Rhbdf1
Kdm7a	Ppapdc3	Mcts1	Gale
Hoxc6	Uqcr10	Tpm2	Gpr37l1
Hoxc6	Armcx3	Ppp6r2	Fabp4
Fes	Gimap4	Ntrk3	Ccdc80
Muc3	Gimap4	Ntrk3	Ccdc80
Hsd3b3	Rcn3	Zfp286	Pigc
Dscaml1	Selm	Zfp286	Tmem41a
F9	Asrgl1	Slamf6	Fam216a
Slco1a5	Asrgl1	Ptcd1	Ncln
Cyb5rl	Dhx36	Zfp106	Ptger4
Galnt6	Rufy3	Rbp3	Slc25a33
Pip4k2b	Cbx6	Sorcs3	Parm1
Myo1b	Lims2	Rbbp6	Mtfp1
BC018473///BC018	Ogfrl1	Ifi27l2b	Meox2
Bmp1	Spon1	Klhl36	Meox2
Atp1a2	Ccpg1	Ncam1	Fam65a
Pigu	Csgalnact2	Hsd3b2	Ces2c///Ces2d-ps
Eml5	Msrb2	Ccdc166	Ifitm1
Ptprb	Mrps6	Tspan33	Lmf1
Itga8	Pja2	Trabd	Slc12a1
Scn7a	Tm4sf5	Rorb	Dclk1
Myh1	Frs3	Gzmn	Dclk1
Plekhs1	Gprc5c	Rho	Haus7
Bicd1	Gprasp1	Sgip1	Pomt1
Slc22a18	Lpcat1	Sgip1	Tmem55a
4930535I16Rik	Lpcat1	Ska1	Slc24a3
Obsl1	Phldb1	Lmbrd1	Tspan17
Sobp	Rapgef3	Phospho2	Oasl1
Krtap16-3	Rapgef3	Ptpn2	Pcdha1///Pcdha10///Pcdha11///Pcdha12///Pcc
[]	Camkk2	Ptpn2	Lpgat1
Rian	Camkk2	Ddx19b	Ube2e2
8430419L09Rik	Camkk2	P2ry6	Ppapdc3
Tsix	Cst8	Ffar2	Mycbpap
Plekhh2	Angptl3	Gngt1	Gimap4
Cacna1h	Txnrd1	Dguok	Gimap4
Fut10	Txnrd1	Tmem258	Car11
Cul9	Ugt3a1	abParts///Igh///I	Rcn3
Muc5b	Zfp768	Ptger3	Selm
Trbv13-2	Cd177	Mad1l1	Cbx6
Ceacam1	Ccdc12	Dixdc1	Cldn23
Pappa	Dram1	Eri3	Ogfrl1
Nek4	Mmd2	Rap1gds1	Spon1
Runx1t1	S100a4	Pear1	Mtap
[]	Car10	Psmc3ip	Msrb2
Trav9d-3	Pycr1	Asph	Abcg4
[]	Ece2	Fbrs	Mrps6
BC025933	Slc25a4	Tsc22d3	Tm4sf5
Siglec1	Slc25a4	Crygn	Trim6
Sema3e	Stac2	Dtna	Gprc5c
Pcdha1///Pcdha10,	Farp2	Zcchc18	Acaa1b
Ifnar2	Srgap3	Rgs3	Gprasp1
Gnrhr	Caap1	Dak	Ipo9
Ceacam1	Samd4	Ncam2	Phldb1
Gdf11	Lmcd1	Prima1	Phldb1
Zfp148	Ddx6	Col26a1	Camkk2
Nfia	Fgl1	Zfp605	Camkk2
Dscaml1	Sumf1	Clmn	Camkk2
Lcn2	Gprc5b	Cyb5r3	Arhgef7
Eri3	Hpd	Plcb4	Angptl3
Camk2d	Cst12	Plcb4	Txnrd1
TCRB///Tcrb-J///Trl	Rev3l	Zfp318	Txnrd1
Slc2a3	Tceal1	Myef2	Flywch2

Defb7	Hmgcl	Cyp2d13	Atl3
Defb10	Pdia5	Aqp4	Oit1
Fgfr4	Eva1a	Pbx1	Atp6v0a2
Defb8	Tspan15	Akr1c20	B230354K17Rik
[]	Slit2	Dnm3	Coa7
LOC171588	Plekhf1	Clec4a2	Grp
Mfsd11	Creb3l3	Chrna2	Tgds
[]	Cacfd1	Nlrp3	Cgref1
[]	Gca	Slc1a1	Mmd2
Ercc6l2	Ccdc136	Glis1	Oas1e
Cebpb	Runx2	Wif1	Nck1
Igkv6-15	Calml4	Krtap19-3	Zfyve27
[]	1300017J02Rik	Oprm1	9430015G10Rik
Myh1	Tmem150a	Msr1	Pycr1
Col3a1	Ccr5	Guca1b	Ece2
Col3a1	P2ry14	Tgfbr2	Slc25a4
Pold4	Rasgrf1	Klra12	Slc25a4
Gimap6	Ap5m1	Il12a	Polr3d
Vasn	Creb3	Grb10	Stac2
Tmem173	Sds	Fbxw11	Srgap3
Rhoq	Galnt11	Senp2	Dnase1
Stx17	Ms4a7	Smyd5	Lmcd1
Maml2	Hip1	Tox	Fgl1
Acyp2	Rsph9	Slu7	Sumf1
Dpyd	Sez6l	Slu7	Gm4354
Dpyd	Cdh22	Bmpr1a	Gm4354
Ccdc102a	Cald1	[]	Gprc5b
Tgif2lx1///Tgif2lx2	Cald1	Mark1	Ifi35
Cdc73	Cald1	Mark1	Camk1g
Gm10436///Gm204	Reep3	Map2k7	Pdia5
Efcab14	Reep3	Pik3r1	Tspan15
Cmip	Reep3	Dhdds	Acp2
Spin4	Reep3	Rbm25	Acp2
Col16a1	Dram2	Prrx1	Slit2
Rufy2	Gm13139	Prrx1	Cux1
BC028528	Wdr45	Prrx1	Mab21l1
6030458C11Rik	Pitx2	Znhit1	Cacfd1
Pitpnc1	Adam5	Bin1	Gca
Tshz2	Enah	Otc	Tmem38b
LOC100653389	Lama4	Trnt1	Runx2
Mchr1	Lama4	Pcdh10	Calml4
Gm568	Cecr5	Rest	Tmem150a
Wdr20	Glycam1	Anxa5	Adig
[]	Rbks	Asap1	Rasgrf1
Ankrd46	Kcnma1	Epha3	Slc25a25
Tmem158	Mef2c	Epha3	Creb3
Slitrk1	Olah	Gfra2	Ms4a7
Sh3bgrl	Homer3	[]	Hip1
Manf	Glyat	Tnpo2	BC018242
Lingo1	Ptprd	Lamc3	Rsph9
Lman1	Rab13	Qk	Sez6l
Eef1d	Gpr85	Gm11787///Lyn	Cdh22
Ap5s1	Lrrc46	Gatad1	Cald1
Eif1b	Cipc	Tmem176a	Cald1
2210016F16Rik	Wipi1	Dusp3	Cald1
Ebf3	Sec63	Galnt2	Oas1a
Ppm1m	Sec63	Cux1	Reep3
Hba-a1///Hba-a2	Iglc1///Iglv1	Epm2aip1	Reep3
Atraid	Myo5c	Lepr	Reep3
Rassf2	Plin5	Rbpms	Reep3
Lrrc8a///Phyhd1	Steap1	Krtap16-1	Dram2
Zcchc3	Ahdc1	Zic5	1700001O22Rik
Col14a1	Ahdc1	St3gal4	Ccsap
Spsb1	Car8	Btrc	Pitx2
Lphn1	Rp1	Cflar	Tmem214
Zswim7	Upp2	B3gat1	Lama4
Rarres2	Purg	Tbx5	Lama4
Ppfia4	Zfp418	Crebzf	Slain1
Ppfia4	Emr4	Abhd14a	Ncmap
Lysmd2	Proser2	Grm1	Gas5
Cand1	Nr2c2	Rgs3	Cep68
Ckmt2	Ubxn2a	Enpp5	Mef2c
Nfam1	Tmem106a	Homer1	Ptprd
Cd99	Mboat2	Akt1	Slc29a4
Parm1	Dnmt3l	Rnf146	Kdm5d
Rftn2	Slc22a19	Iigp1b	Mtfmt
Slc9a3r2	Kcnj6	Ppp2r5c	Wipi1
Slc9a3r2	Ppp6r2	Cldn19	Gdap1l1
Abcc3	Zfp472	Mxi1	Dnah8
Ptprd	Prmt6	1500002F19Rik	Steap1
Limd2	Wasf3	Mgat5	BC021614
Toporsos	Fcrl1	Stxbp6	Pip5k1c
Zbtb46	Fcrl1	Bcat2	Ahdc1
Sox17	Arnt	Diablo	Ahdc1
Cdadc1	Ntrk3	A730028G07Rik	Tapbpl
Saal1	Gatad2b	Nmnat1	Rp1
5730408K05Rik///N	Dnajc18	C87436	Ppp1r42
Ikbip	Zfp286	Tc2n	Odf4
2610524H06Rik	Zfp286	Olfm1	Glyctk
Rbpms	Otor	Pola2	Glyctk
Ercc8	Gimap7	Map3k7	Sectm1a
Calcoco2	Ptcd1	Mbd2	Tcte2
Ppap2b	Zfp106	Med21	Ephb2
Plscr1	BC006965	Myocd	E430018J23Rik
Pet100	Klhl36	Fabp4	Tpm2
Wrnip1	Oit3	Zfp287	Slc17a2
Nudcd1	Ncam1	Dtx1	Dnmt3l
DXBay18///Gm146	Rab3il1	Sorbs1	Lhx3
Dtna	Dnm2	Slc26a7	Ppp6r2
Pvr	H2-Q10	Edil3	Ppp6r2
Ahnak	Rorb	Zak	Wasf3
Nupl1	Gngt1	Prlr	Arnt

Cdc42ep5	Adam15	Psen2	Ntrk3
Ppp3cc	Rho	Kcnip2	Ntrk3
Tsnax	Sgip1	Gm10229	Skp2
Bcat1	Sgip1	Lepr	Dnajc18
Tmod2	Klk1b22///Klk1b	Lepr	Zfp286
Grb10	Lmbrd1	Grtp1	Zfp286
Cyp4f16///Cyp4f37	Ptpn2	Fbn1	Otor
Cyp4f16	Ube3a	[]	Rarres2
Aktip	Lbh	Nfkb2	Slc44a3
Dqx1	P2ry6	Gnb1	Sptbn4
Eif2ak3	Scgb3a2	Fgfr1	Spire2
Glb1l	Krtap6-5	Cep164	Rnf183
Sulf2	Slc14a2	Spats2l	Ncam1
St8sia4	Dixdc1	Ifi44l	Ptpn5
Abcb9	Pear1	1810055G02Rik	Rab3il1
Pcdh18	Pear1	Atf5	Gbp7
Bcl2l2	Asph	Xkr6	Golt1a
Sncaip	Slit1	Mlx	Rorb
Auh	Tsc22d3	Nt5c2	Gngt1
Cd99	Crygn	B4galt4	Rho
Vamp5	Zfp189	Hspb11	Sgip1
Armc6	Zcchc18	Ankmy2	Sgip1
Mvk	Rgs3	Fanci	Klk1b22///Klk1b9
Pla2g7	Zfp825	Gpm6b	Klhl25
Tcte2	Naip1	Klra16	Tnfrsf19
4933428G20Rik	Ncam2	Myocd	P2ry6
Habp4	4930432F04Rik	Col5a3	Slc22a29
Prss23os	Carf	Masp1	Birc3
Ube2s	Dock7	Eya3	Tcf7l2
Morn3	abParts///Ighm	Kank2	Wsb1
Pdia6	Rab43	Slc7a2	Slc14a2
Farsb	Plcb4	Epb4.1l3	Mad1l1
Ubap2l	Plcb4	Tro	Acot5
Sqrdl	Ccdc28a	Plaa	Pear1
Scoc	Zfp318	Cdc7	Pear1
Psmd11	Zfp318	Dbn1	Kif1b
Snca	Myef2	Prpf6	Asph
Pitpnc1	Myef2	Pde1c	Tsc22d3
Fbxo3	Srxn1	Tfap2a	Crygn
Hoxd8	Acsf2	Cenpb	Cr2
Rragd	B4galnt1	Npy1r	Dtna
Fndc4	Sox10	Gem	Zcchc18
Clec2h	Erg	Fmr1	Rgs3
Hspa9	Dnajc19///Dnajc	Fert2	Naip1
Tmem263	Herc1	Ccdc106	Ncam2
Twf2	Pbx1	Tk2	BC027072
Tmod2	Ube4a	Ubxn11	Trim31
Wfdc1	Adat1	Asb18	Dock7
Parva	Cpsf4l	Tomm40	Gm13271///Gm13272///Gm13275///Gm13276
Mbtps1	Dnm3	[]	Ms4a4b
Jam2	2610034M16Rik	Park2	Cd8a
Cxxc5	Slc1a1	Ackr4	Plcb4
Synj2bp	Klra21///Klra8	Chpt1	Kcnk3
Cd164	Lats2	Cldn10	Ccdc28a
Psen2	Glis1	Gipc3	Srprb
Gm15270	[]	Stx3	Myef2
Lypd1	Wif1	Gm21320///LOC	Myef2
Dpp3	Gm20767	Lats2	Enpp3
Lhx9	Hif3a	Stk16	Riok1
Uggt2	Krtap16-3	Lat2	Hus1
Ccdc97	[]	Hax1	Itgal
Dtnbp1	Msr1	Dusp15	Numb
Gtf2i	Guca1b	Rev3l	Dnajc19///Dnajc19-ps
Gmfb	Oscar	Igh-VJ558	Tdpoz1
Kazn	Tfap2d	Plagl1	Herc1
Lig3	Tgfbr2	Tmem161a	Hgf
Bnip2	[]	Tmem161a	Akr1c20
Mto1	Ppap2a	Tmem216	Mfsd7b
Sdccag3	Klra12	Ttc39c	Cpsf4l
Irak1bp1	Il12a	Cmtm2a	Dnm3
Cbx1	Grb10	Bmp1	2610034M16Rik
Hmgn3	Mtmr2	Polr2a	Tprkb
Ypel1	Senp2	Mapre2	Rfx3
Stk11ip	Vps39	Mapre2	Slc1a1
Rgl3	Tox	Timm17a	Gm20274
Emilin1	Tox	Pank3	Glis1
C1qtnf6	Mtmr6	Tmem203	[]
LOC102636151	Slu7	Wdpcp	Wif1
Mus81	Slu7	Lama2	Krtap16-3
C4bp-ps1	Wdr13	Atxn7	Msr1
Pde3a	[]	Maged2	[]
Sep-06	[]	Bre	Guca1b
As3mt	Mark1	Bre	Tgfbr2
Artn	Map2k7	6330416G13Rik	Cldn18
Prdm5	Pik3r1	6330416G13Rik	Grb10
Cyp1b1	Dhdds	Bcl2l11	Senp2
Prrx1	P2rx4	Ntng1	[]
Tex13	Prrx1	Prepl	Slu7
Eng	Prrx1	Prepl	Mylk
Sox5	Prrx1	Col4a1	Pik3r1
Tlcd2	Znhit1	H2afy2	Ogt
Sntg1	Bin1	Far1	Ogt
Ost4	Otc	Far1	Paip1
Matk	Plekha5	Oxnad1	Prrx1
Prrx2	Plekha5	Reep5	Prrx1
Sync	Lst1	Tgfbr2	Bin1
Spr	Cdk12	Vwa1	Plekha5
Epha1	Cdk12	Capns1	Plekha5
Bmp7	Trnt1	Neurod1	Lst1
Ak3	Pcdh10	Atoh8	Tsc22d3
Spn-ps	Rest	Thap7	Trnt1
Pde10a	Slamf1	Ddx3y	Pcdh10

Nnmt	Epha3	Dhrs7	Rest
Nup54	Epha3	Gpm6a	Rest
Sec62	Ahcyl1	Rhbdd2	Asap1
Prrg2	Gfra2	Nup35	Epha3
Nkx2-4	Emcn	Pja1	Epha3
[]	Lamc3	Plcl2	Ahcyl1
Tgm2	Gm11787///Lyn	Rps6kl1	Gfra2
Ttc7b	Plcb1	0610037L13Rik	Emcn
Edil3	Tmem176a	0610037L13Rik	Mlph
Epb4.1l2	Slc5a8	Tbp	Tnpo2
Epb4.1l2	Ncf1	Hmbs	Lamc3
Reep1	Edil3	Klhl22	Qk
Fli1	Nol6	Ubxn4	Gm11787///Lyn
Timp2	Ppp1r3c	Bfar	Plcb1
2210016L21Rik	Cfhr2	Tdp1	Tmem176a
Ppp1r3c	Lepr	Kdm5c	Crkl
Ldb3	Rbpms	Rnf121	Ncf1
Sun2	St3gal4	Sccpdh	Cux1
Sdhaf1	Mapk8ip1	Chst15	Cux1
Zik1	Btrc	Lpin1	H2-D1
Tufm	[]	Gnaz	Tgfbr3
Cdh5	Akr1b10	P4ha1	Edil3
Rrnad1	Cflar	Gnpda2	Epm2aip1
Fermt3	Dpys	Nrp2	Gstm1
Fam117b	B3gat1	Trp53	Nol6
Repin1	Dnah8	Usp11	Gypa
Repin1	Tbx5	Endod1	Lepr
Ubfd1	Nxnl1	Sidt1	Trim60
Lamtor1	Abhd14a	Scpep1	Slc39a14
Tcf4	Rgs3	0610010B08Rik/,	Rbpms
Tcf4	Enpp5	Olfm1	Cdadc1
Khdc1b	Dhrs11	Me2	Cdadc1
Fermt2	Ero1lb	Add3	Klhl20
Fermt2	Kcnj6	Gnl2	Zic5
Shmt2	Nfam1	Atf2	St3gal4
[]	Iigp1b	Atf2	Lpp
Fam174b	Ppp2r5c	Gab3	Mapk8ip1
Snhg11	Ppp2r5c	Uty	Tprkb
Prkar1b	Cldn19	Crtac1	[]
Coro2b	Ankrd24	Ttyh1	Akr1b10
S100b	Pld1	Ppp2r2b	Akr1b10
Fez2	Suv420h1	Qpct	Cflar
Sfmbt2	Stxbp6	Tmem184c	Dpys
Map3k14	Pitpnm1	Tmeff1	B3gat1
AW112010	Bcat2	Mcm3	Tbx5
Gbp7	[]	Zc3hc1	Tbx5
Rnf157	Six4	Ano10	Abhd14a
Morc4	Col4a4	Flna	Grm1
Samhd1	Tmem167	Sepn1	Rgs3
Atcay	Rorc	Lrpap1	Rnf17
Papss2	Fmo4	Antxr2	Akt1
Aard	Myocd	Calm1///Calm2/,	Bak1
Rps6kc1	Calcrl	Tmco3	Ppp2r5c
Tbca	Zfp287	Apbb2	Ppp2r5c
Sema4d	Slc8a1	Cnn3	Srgap3
Hsd17b11	Zbtb7c	Des	Stxbp6
Ccm2	Dtx1	Abcf3	Pitpnm1
Ctbp2	Cfhr2	Ckap4	Six4
Ero1lb	Sorbs1	Ampd2	Akr1d1
Coro2b	Bbx	6330403K07Rik	Nmnat1
Sema5a	Slc26a7	Msl3l2	LOC101055731///LOC101055745///Sytl3
Cacng5	Shoc2	Tyw1	Fcrla
Pkig	Nek6	Dhx38	Myocd
Hmgn3	Amigo1	Ssrp1	Fabp4
Atp1a2	Catsperg1	Rusc2	Calcrl
Hspb7	Prlr	Rusc2	Dtx1
Tpcn1	Plekha4	Rab8b	Cfhr2
Dnajc27	Psen2	Sep-08	Eml6
Sacs	Igk-V28	Fmnl3	Sorbs1
Dhh	Gm10229	Eif4g3	Gpam
Eif4ebp1	Lepr	Map2k6	Limk1
Pi4ka	Lepr	Nov	Slc26a7
Zfp787	Zfp352	Nov	Edil3
Tmem27	Zfp352	Arhgef25	Dusp26
Pitpnc1	Gdf2	Inhbb	Nek6
Klhdc2	Kcnab2	Ncam1	Prlr
Tvp23a	Wnt9a	Ncam1	Cacna2d1
Elk3	Ly6i	Chst14	Psen2
A230050P20Rik	Fhit	Nudt10///Nudt1	Lepr
Gjc2	Mrgprf	Ehmt2	Lepr
N6amt2	Fbn1	Zfp191	Wnt9a
Cox6b2	Dnajc2	Jmjd1c	Pnoc
1110004E09Rik	Fgfr1	Dnajc10	Fbn1
Far1	Armcx1	Dnajc10	[]
Psma6	Ifi44l	Lss	Olfm3
Psma6	Otx2	Dapk1	Itsn1
Lpgat1	Atf5	Rc3h2	Nfkb2
Lpgat1	Mlx	Plcg2	Amot
Fam105a	Hspb11	Nova1	Fgfr1
Ndn	Gpm6b	Tsr2	Cep164
Ndn	Ifng	Fbxw8	Slc26a8
Vwf	Slc17a9	Col6a2	Ifi44l
Rhoc	Gcg	Tpr	[]
B3gnt9	Klra16	Parp16	Ndufa12
Fcgr2b	Ubc	Rps9	1810055G02Rik
Bmp7	Mcpt4	Rdh10	Atf5
Adora1	Speg	Sec24d	Xkr6
Ptprd	Mapk8ip3	Gpr153	Rad50
Rasgrf1	Myocd	2310067B10Rik	Gpm6b
Hs3st3a1	Col5a3	Klhl42	Rad51d
Il17d	Masp1	Cubn	Slc25a30
Snrpn///Snurf	Nfatc2	Pcnxl4	Tbata

Rnaseh2c	Kank2	Zfand3	Il1f9
Vimp	Asah2	Fbxo2	Ubc
Fgf14	Oxtr	Dennd4b	Speg
Cebpzos	Eomes	Ptprz1	[]
B4galt6	Dmp1	Scara3	Masp1
Fam169a	Ubxn10	Fth1	Eya3
Aplp1	Pip5k1a	Mtmr7	Nfatc2
Cd300a	Ccar2	Myh4	Kank2
Atp1a1	Tro	Htra3	Wt1
Tfb1m	Tmpo	Ace	Oxtr
Dpep1	Dbn1	Slc39a14	Eomes
Taok3	Laptm5	Penk	Cdc7
Meg3///Meg3	Prpf6	Amph	Kcnq2
Kalrn	Nfat5	Synpo	Slc7a2
Slc6a17	Nfatc2	Acacb	Cdhr5
Gm20559	Capn3	Adamts15	Tro
2310075K07Rik	Tfap2a	Nt5dc3	Tmpo
Tcf7l1	Xpr1	Stard8	Dbn1
Tle2	Pigq	A630072M18Rik	Nfat5
[]	[]	[]	Rgs16
Dpys	[]	Maz	Ptprr
Vps53	Cyp3a44	9530068E07Rik	Tfap2a
Zbtb12	Cdc27	Tmem259	Xpr1
Ran	Cd247	5430421N21Rik	Pigq
1110057K04Rik	Wnt10b	Fbxo4	Gem
4932441J04Rik	Gm10997	Copg2os2	Cmklr1
Ceacam10	Cd72	Adamts10	Moxd2
Rab40b	Kcnj9	Bckdhb	Gpr108
Pag1	Cd244///LOC677	Ccdc85a	Kcnq2
Bola2	Coro6	Armcx4	Dio2
Hamp2	Klra18	Col14a1	BC003331
B130024G19Rik	Trdn	Slc27a3	Fert2
Slc35a2	Park2	Ldlrad4	Ccdc106
Rundc3a	Klra21	Ldlrad4	Cyp2j13
Aldh9a1	Rex2	Npr2	Asb18
Nrep	Mrgpra1	BC022960	Rrbp1
Sorbs1	Trdn	Mettl20	Klra18
Cnn3	Casp3	Afg3l2	Prss44
Fam13c	Mup5	Krtap8-1	Mitd1
Pgd	Klra7	Mapkap1	Park2
Slc7a4	Ighg	Pfkfb1	Klra21
Wnt5a	Il24	Ifnz	Rex2
Ttll1	Pdcd6ip	B930041F14Rik	Ackr4
Cnn3	Fgf5	Ift122	Trdn
Impa1	Dusp15	Ddx4	Cldn10
Pkn1///Ptger1	Bcl2l1	Rell1	Gm21320///LOC100048884///Mup1///Mup10/
Il7	Otos	Dmrtb1	Usf1
Tmsb10	Igkv4-72	Suz12	Mup5
Gpsm1	Krtap19-4	Vcan	Cd8b1
Rab2b	Parp3	Vcan	Cd209a
Klf9	Imp4	Trim24	[]
Wipf1	Tmem216	Rnf214	BC005685
Wipf1	Ttc39c	Six1	Dusp15
Pdgfrb	Cmtm2a	Malat1	Ighm
Cct8	Rbp4	Slc38a10	Oprl1
Rbm14	Vit	Slc38a10	Plagl1
Sntb2	Bmp1	Dnm3os///Mir21	Tmem161a
Pfn2	Mapre2	Ptgir	Glul
Lyz1	Mapre2	Tmed7	Glul
Sh3bgrl	Mau2	Brat1	Bmp1
Pla2g1b	Cpz	A230046K03Rik	Mapre2
Rbm14	Umod	Tmem120b	Mapre2
Slc2a3	Tm2d1	Akna	Pros1
Api5	Nefl	Ighm	Umod
Phyhipl	Pank3	Cars	Pacrgl
Lats2	Vamp7	Slc30a2	Tm2d1
Igf2bp2	Smc5	Gtpbp6	Smc5
Gm15393///Gm15	Smc5	Clasp1	Smc5
Foxm1	Wdpcp	Dapk1	Slc9a8
Rbpms	Ntm	Kdm7a	Uxs1
Pcolce	Lama2	Hoxc6	Catsper1
Gsn	Dimt1	Hoxc6	Krt20
Tmsb10	Zfp790	Fes	Lama2
Gabbr1	Hapln1	Abca8a	Hapln1
Mapk10	Hapln1	Hsd3b3	Hapln1
Ccdc88a	Rad52	Krt75	Snx20
Xbp1	Upk1a	Aldh1a3	Maged2
Prmt2	Maged2	Slco1a5	Bre
Lpp	Asb9	Mar-09	Stard6
D2hgdh	Pdgfd	[]	Kcnmb2
Ctnna1	Tbx4	Pip4k2b	Ntng1
Tgm2	Scn3b	Prmt5	Slc1a3
F2r	Cacng5	Abcf1	Col4a1
Pttg1ip	Frmd4b	Ttn	H2afy2
Bscl2	Bcl2l11	Myo1b	Rin2
Ddx39b	Cacng7	Cpn2	Far1
Gtf2h4	Ntng1	Hspa5	Arf1
Fgd1	Slc1a3	Pigu	Tgfbr2
Scarb1	Prepl	Ppp3cb	Capns1
Pgd	Col4a1	Napb	Neurod1
Slc52a2	Zbed6///Zc3h11a	Usp12	Gm17066///Rbm26
Creb3l2	H2afy2	Atp1a3	Slc4a4
Aldh9a1	Far1	Lrr1	Dhrs7
Igf1	Far1	Mphosph8	Gpm6a
Igfbp4	Far1	Epha7	Rhbdd2
Igfbp4	Zfp281	Obsl1	Rps6kl1
[]	Tgfbr2	Sobp	0610037L13Rik
[]	Rbm26	Uba1y	Tbp
Sgcg	Ttll1	Clns1a	Zfp52
Gnl2	Rhbdd2	Kmt2d	Rasa1
Mmp16	Pja1	Atf2	Rasa1
Atf7	Pja1	Prkca	Klhl22

Rac1	Plcl2	Tpm3	Bfar
Glrx2	Dlgap4	Ift80	Tdp1
Cux1	Rps6kl1	Igk-V28///Igkv6-	Tifa
D1Pas1	Tbp	Rhbdl3	Gfap
Pcbp3	Zfp52	Plekhh2	Sccpdh
Pitpnm1	Rasa1	Gm13583	Olfm3
C1qb	Rasa1	Cdh9	Chst15
Lrrtm1	Klhl22	Pappa	Nrp2
Tmem158	Bfar	Kif5a	Klhl5
Fuca1	Kifc2	Runx1t1	Usp11
Zbed3	Kdm5c	Zyg11a	Ttc14
Aar2	Tifa	Tcrb-J	0610010B08Rik///Gm14295///Gm14305///Gm
Armcx2	Evi2a-evi2b///Ev	Clk4	Igf1r
Ndn	Sccpdh	Nfic	Slc2a9
Bgn	Chst15	Siglec1	Me2
Rarres2	Gnaz	Ifnar2	Add3
Lox	Btg4	Pou2f1	Fbxo22
Ergic1	Gnpda2	Nf2	Slc37a1
Bcl2a1a///Bcl2a1b/	Nrp2	Smyd1	Crtac1
Mrpl42	Zmynd11	Psg19	Ppp2r2b
AU040096	Zmynd11	Zfp148	Qpct
Grin1	Trp53	A230006K03Rik/	Xiap
1110057K04Rik	Usp11	Gm4736///Gm8	Six3
Ap1s2	Endod1	Lcn2	Trib2
Spata6	Endod1	Igk	Trib2
Rmdn1	Endod1	Tcf3	Myh8
Gm9222	Mri1	Cftr	Ano10
Ptov1	Sidt1	Rabac1	Flna
Rbm5	Scpep1	Nespas	Sepn1
Hhip	0610010B08Rik/	LOC171588	Unk
Ankrd54	Olfm1	Ctse	9030624J02Rik
Vim	Zfp553	[]	Antxr2
Pex6	Slc2a9	Slco1b2	Calm1///Calm2///Calm3
Zdhhc14	Me2	Zfp260	Tmco3
Pcsk5	Snapin	Cebpb	Slc6a15
Ikzf4	Atf2	abParts///Igkv6-	Slc46a1
Cdk16	Tspan10	Hbb-b2	Apbb2
Ccdc102a	Gab3	Col3a1	Cnn3
Slc22a6	Fbxo22	Col3a1	Ets1
Cnn3	[]	Gimap6	Ptdss2
Pfdn5	Ttf1	Vasn	Des
Acly	Ndrg4	Pip5kl1	Des
Zbtb20	Tlcd1	Phpt1	Fam43a
Gm13102///Prame	Tmem184c	1110037F02Rik	Ckap4
Nos1	Kctd14	Ssbp3	Galnt2
Masp1	Six3	Rhoq	Ampd2
Masp1	Tcf7l2	Srpx2	Meg3
Pgd	Trib2	Pdxk	Oaz2
Lasp1	Ano10	Lyrm2	6330403K07Rik
Aplnr	Sepn1	Acyp2	Pex5
Mmd2	Map3k3	Dpyd	Trim47
Arf2	Lrpap1	Dpyd	Dhx38
Ptpn1	Antxr2	B9d2	Rusc2
Slc4a7	Slc6a15	Pcgf1	Ptprs
Gbp6	Slc46a1	Ccdc102a	Sep-08
Edn3	Slc46a1	Zfp689	Sep-08
Cbfa2t3	Cnn3	Efcab14	Fmnl3
Pklr	Fam131a	Cmip	Ddx26b
Osbpl6	Donson	Col16a1	Atg2a
Cacul1	Appl2	Rab12	Nov
Dpysl3	Ampd2	Cpa5	Nov
Ergic1	Meg3	BC028528	Arhgef25
Trp53	Oaz2	Thtpa	Inhbb
Eftud2	6330403K07Rik	Timm9	Rbmx
Tnfaip2	Msl3l2	Rasip1	Ncam1
Heyl	Ptprs	Map1lc3b	Ncam1
Maea	Sep-08	Pitpnc1	Chst14
Sccpdh	Tprn	Onecut3	Srxn1
Fdft1	Psme4	Ap3s2	Slc25a45
Fibp	Fmnl3	Nudt16l1	Jmjd1c
Rasgrp2	Ahcyl1	Cacna1d	Fndc3a
Rasgrp2	Ddx26b	Rian///Rian///Ri	Mndal
Ampd2	Nov	Arap1	Galnt7
Iqcf3	Nov	Slc44a2	Dapk1
Acyp1	Arhgef25	Tmem158	Itgb1
Ufm1	Inhbb	1110057K04Rik	Rc3h2
Adcyap1r1	Inhbb	Slitrk1	Ccdc93
Wipi1	Rbmx	Sh3bgrl	BC005512///F630007L15Rik///Gm6958
Nynrin	Ncam1	Tmcc2	Medag
Polr3f	Ncam1	Vps11	Nova1
Ahi1	Chst14	Manf	Col6a2
D17Wsu92e	Slc25a45	Rab2b	Parp16
Cops5	Zfp191	Lingo1	Rps9
[]	Jmjd1c	Atxn7l3b	Pacs2
Enpp3	Mndal	Lman1	Axin1
Lasp1	Lss	Cd47	Rdh10
Mmp2	Dapk1	Strada	Uba7
Slc9a3r2	Scrn3	G6pc3	Uba7
Wnt5b	Rc3h2	Snrnp40	Gpr153
Meg3	Medag	Eif1b	Flywch1
Ppp2r4	Nova1	Polr2l///Usp50	Fnbp1
Slc13a3	Col6a2	Pdlim7	Cubn
Psmc1	Parp16	Arxes1///Arxes2	Arsg
Med1	Rps9	Scfd1	Fbxo2
Lyz1	Axin1	Ebf3	Plk2
Twf2	Rdh10	Ppm1m	Car9
Lats2	Gpr153	Hba-a1///Hba-a	Nckap5l
Slc52a2	Os9	Atraid	Satb2
Rnaseh2c	2310067B10Rik	Ppdpf	Tsnaxip1
Lct	Cubn	Nub1	Ptprz1
Pramel7	Xpr1	Col14a1	Scara3
Fgf11	Fbxo2	Bola1	Lgr6

Abhd11os	Satb2	Spsb1	Htra3
Syt2	Scara3	Lphn1	Ace
D9Ertd26e	Phyhipl	Rarres2	Slc39a14
Slco5a1	Htra3	Basp1	Penk
Cbfa2t3	Ace	Chn2	Mdfic
Zfp771	Slc39a14	Chn2	Amph
D18Ertd232e	Penk	Ppfia4	Acacb
C76132	Mdfic	Ppfia4	Abi3bp
Brwd3	Amph	Atg7	Abi3bp
Dpp4	Synpo	Cox20	Adamts15
D2Ertd127e	Stard8	Elavl4	Snx21
Kalrn	Pcmtd2	Tha1	Stard8
C78878	[]	Pgp	Snx19
C79122	Ssr4	Nfam1	Mapre3
[]	Zfp612	Gata2	A630072M18Rik
[]	Myh3	Cd99	Maz
C79452	Fbxo4	Ptch1	Slfn4
[]	Lrrc58	Nolc1	Cenpn
[]	Adamts10	Rftn2	Zbtb3
C77691	Iqsec1	Hmg20a	Tmem259
DXErtd242e	Plekha6	Tril	Ttc19
Habp2	Ccdc85a	Ankzf1///Glb1l	Mtmr3
[]	Armcx4	Ptprd	Lrp2
Zc2hc1c	Mrps33	Limd2	Adamts10
[]	0610010B08Rik/	Msl2	Plekha6
C78532	Krt33b	Toporsos	Armcx4
[]	Ldlrad4	Zbtb46	Col14a1
Gm6211	Npr2	Mtf1	Psma8
[]	Mustn1	Cdadc1	Ldlrad4
D12Ertd216e	Cep112	5730408K05Rik/	Ldlrad4
D3Wsu167e	Ifnz	Lrrc51	Efemp1
D8Wsu26e	Acsm2	Ikbip	Npr2
C77815	Gabrg1	2610524H06Rik	Brd8
C77137	Robo1	Anxa6	Atr
[]	Dock6	Qrsl1	Myo15b
D7Bwg0826e	Vcan	Qk	Cep112
Nucks1	Vcan	Rhoh	Krtap8-1
Auh	Six1	Rbpms	Pfkfb1
Egfl8	Scn2a1	Eny2	Ifnz
Zfp398	Malat1	Tcf7l2	Klhl11
Mrpl38	Ddhd1	2610301B20Rik	Svs5
[]	Dnm3os///Mir21	Ptgr2	Robo1
Ttl	Cd48	Plscr1	Dock6
[]	Enpp3	Rae1	Ddx4
C78441	Ptgir	Cep112	Vcan
D17892	A230046K03Rik	Trappc4	Vcan
Cdca3	Wipi1	Pmpcb	Scn2a1
Fam46c	Akna	Rtn1	Malat1
Tbc1d24	2810474O19Rik	Dtna	Ddhd1
Gnao1	Slc30a2	Arl2bp	Sycp1
Tfpt	Sult1a1	Cyp2c65	Auts2
LOC102640476///R	Zfp931	Tlk2	Dnm3os///Mir214
Nmrk1	Kdm7a	Grb10	Cd48
[]	4930507D05Rik	Rwdd3	Ptgir
Rln1	Fes	Cyp4f16///Cyp4f	Wipi1
AA408251	Nlrp6	Ppp1r42	Ighm
Colq	Abca8a	Wdr77	Cars
D4Ertd58e	Hsd3b3	Gusb	Slc30a2
Necap1	Actn1	Eif2ak3	Sult1a1
Gdf9	Lrrc2	Glb1l	Gm10439///Gm15080///Gm15085///Gm15093
Gusb	Lbx1	Sulf2	Zc3h12a
Enpp2	Polr2f	Abcb9	Zfp931
Igf2	Slco1a5	Pcdh18	Ighm
Vcam1	Dleu2	Sncaip	Dapk1
Pnliprp2	Suco	Auh	Kdm7a
Sfrp2	[]	Vamp5	4930507D05Rik
Sav1	Pip4k2b	Ctnnb1	Hoxc6
Lasp1	Myo1b	Erh///LOC10105	Hoxc6
Slc22a17	[]	Dbf4	[]
Pdhb	Hoxc6	Mrpl38	Fes
Syngr3	Cpn2	Pfkp	Abca8a
Lox	Pigu	Pibf1	Trmt10a
Bcl7b	Ppp3cb	Msh5	Hsd3b3
Gpd1	Fbxl3	Mbd1	Irx6
Clmp	Leap2	LOC102641923/	Lrrc2
Clmp	Lmod1	Scoc	Igf2os
Fstl1	Birc6	Mrpl3	Muc4
Klhl13	Abcb7	Gm21540///Tme	Lbx1
Tspan4	Zfp81	Htatsf1	Polr2f
Ebf1	Adam4	Plscr3	Slco1a5
Slc1a1	Arsi	Rabl3	Eno1///Eno1b
Cmtm3	Baiap3	Pcmt1	Scml4
Bgn	Adora2a	Tmem33	Nkx6-2
Crabp1	Myh1	Auh	Cyb5rl
Adprh	Plekhs1	[]	Galnt6
Tmem30a	Arhgap20	Cuedc2	Slc25a51
Ccng2	Mphosph8	Hspa8	Six2
Mylpf	Fgfr1op2	Tom1	Pip4k2b
Mmp14	Obsl1	Slc9a3r2	Myo1b
Sparc	Zfp125	Fndc4	BC018473///BC018473
Myl2	Zfp369	Mga	Igk-V28///Igkc///Igkj1///Igkv4-53///Igkv6-23///
Lrmp	Sobp	Twf2	Bmp1
Wfs1	Uba1y	Stx18	Hspa5
Sema3b	Scml4	Tmod2	Atp1a2
Aspn	Slc26a3	Parva	Usp12
Frzb	Prkca	Mbtps1	Ptprb
Gyg	Diap2	Polr2m	Scn7a
Pcolce	Utrn	Lrrk2	Srsf2
Serpini1	Srpr	Iqgap1	Plekhs1
Chkb	Chic1	Jam2	Epha7
Ripk3	Fbxw14	Stk39	Slc22a18
Irf8	Igk-V28///Igkv6-	Cxxc5	Obsl1

Hsd3b1	Rian	[]	Sobp
Kcnip1	Hic2	Sh3kbp1	Slc26a3
Acvr1	Fgf6	Uggt2	Krtap16-3
Ehbp1l1	Rictor	Dtnbp1	Gstz1
Nid1	Zfp28	Rnf219	Kmt2d
Olfml3	Clcn1	Gtf2i	Mylk2
Chst12	Gm9798	Gmfb	Diap2
Ggt1	Zfp697	Il17b	Abcc5
Thbd	Tsix	Lig3	[]
Prl8a9	Plekhh2	Slc7a13	8430419L09Rik
Acy3	Cacna1h	Smap1	Clcn1
Sdc2	Sox14	Sdccag3	Tsix
Lbp	Ghrhr	Ctrl	Plekhh2
Prlr	Defb9	Irak1bp1	Boll
Fam13c	Itga4	Cbx1	[]
Zfp346	Gm13583	Hmgn3	Cacna1h
Rsrc1	Fut10	Ypel1	Sox14
Col6a1	Sh3tc1	Sema3b	Ghrhr
Crtap	[]	Rgl3	Itga4
Wisp1	Tmed8	Exosc10	Cul9
Rhoc	Herc2	Emilin1	[]
Lpar1	Il22///Iltifb	C1qtnf6	Herc2
Nt5c2	BC038268	Phf19	Muc5b
Slc14a1	Ceacam1	Mus81	Ptprj
Fam114a1	Pappa	Mllt3	Ceacam1
Enpep	Kcnh7	As3mt	Cd55///Daf2
Lrp1	Runx1t1	Dnm2	Pappa
Speg	[]	Ap2a2	Runx1t1
Pvrl3	Il1rapl2	Arhgap31	[]
Rnf25	Synj2	Artn	Arhgef2
Camk2b	Tcrb-J	Smu1	Runx1
Podxl	Ctbs	Prdm5	[]
Kcnk1	Tssc4	Ccdc132	BC025933
Cxcr4	Tcrb-J	Sh3gl3	Clk4
Tle6	Siglec1	Pign	Siglec1
Cpa3	Kdm6a	Prrx1	Cit
Cp	Vmn1r45	Eng	Kdm6a
Rbp1	Zim3	Toe1	Pcdha1///Pcdha10///Pcdha11///Pcdha12///Pcd
Col15a1	Tpte	Fbxo9	Ifnar2
S100a9	Ifnar2	Sh3kbp1	Gnrhr
Il2rb	Gnrhr	Ackr1	Pou2f1
Mog	Smyd1	Tlcd2	Fech
Slc35b1	Nt5c1b	Ap5s1	Six4
Gm16340///Ifi203/	Cd80	1700074P13Rik	Ceacam1
Gadd45gip1	Chrng	Prrx2	Smyd1
Slc12a2	Ehd2	Bmp7	Cd80
Runx1t1	Dscaml1	Kansl1	Psg19
Cd200	Gm12606	Ak3	Chrng
Aldh1a3	Lcn2	Rnf19b	Nfia
Sema6b	Dnm1	Sf3a3	Dscaml1
Cyp2f2	abParts///Ighm	Ube4b	Acta1
Elk3	Art5	4933429H19Rik	Lcn2
Cyp11a1	Gm7040///Prl2c	Cd80	Igkv1-117
Gne	Defb15	[]	Art5
Wnt5a	Rabac1	Tgm2	Itgb1
Myh6	Defb7	Cdk5r1	Defb10
Kank3	Fgfr4	Ttc7b	Fgfr4
Cdo1	Fgfr4	Tcf7	Defb13
Ltbp1	Defb8	Edil3	Nespas
Evc	Defb35	Epb4.1l2	[]
Hp	Usmg2	Epb4.1l2	LOC171588
Cpxm1	[]	Glt25d1	[]
Mrps34///Nme3	Krtap19-1	Reep1	[]
Ppap2b	[]	Fli1	[]
C1qtnf1	Tyms	Etnk1	[]
Cd302	Ids	Rpl41	Copg2os2
Twist2	[]	Gns	[]
F13a1	[]	Fam21	[]
Gng11	[]	Inpp5a	Abcd4
Pllp	[]	Ap2s1	Pou2f1
Tnfrsf1b	Slco1b2	Pank3	Tmem189
Cxxc5	[]	Pcbp4	Cebpb
S100g	Zfp260	Timp2	abParts///Igh-VJ558///Ighm///Ighv1-53///Ighv1
Nfat5	Tuba-rs1	Snhg1	abParts///Igkv6-13///Igkv6-14
Tfap2c	Krtap6-3	Snhg1	LOC102633540
Acadl	Igh-VJ558	Pld4	Myh1
Myo1b	Igkv4-73///Igkv4	2210016L21Rik	abParts///Ighm
Myo1b	abParts///Igkv6-	Zfp213	Col3a1
Rom1	LOC101055909//	Srp68	Col3a1
Cyth1	Hbb-b2	Cd38	Gimap6
Slc16a3	LOC102633540	Ubn1	Vasn
Gla	Myh1	Ldb3	Maml2
Fndc4	Ptafr	Calr	Pdxk
Retnla	Defa15	Dusp9	Acyp2
Nes	Col3a1	Abat	Dpyd
Hexa	Col3a1	Peg3	Dpyd
Rhou	Gimap6	AU020206	B9d2
Rhou	Vasn	Spag7	Zkscan14
Hsd11b1	Tmem173	Zik1	Pcgf1
Rab4a	Ssbp3	Pla2g1b	Abhd10
Tdh	Rhoq	Dhps	Ccdc102a
Slc2a2	Vma21	Fam117b	Tgif2lx1///Tgif2lx2
Apcdd1	Acyp2	Pdap1	Cacna1d
Fbp2	Dpyd	Repin1	Gm10436///Gm2042///Oog1
Gjc1	Dpyd	Synpo	Efcab14
Ccdc127	B9d2	Mul1	Cmip
Ebf2	Ltn1	Zg16	Csad
Ebf2	Aipl1	Prune	Col16a1
Upk3a	Abhd10	Tcf4	Rufy2
Gpx3	Ccdc102a	Tcf4	Cpa5
Socs2	Cmtm8	Fermt2	BC028528
Zfp90	Zfp689	Fermt2	Mar-01

Selplg	Gm10436///Gm2	Tmem151b	Scfd1
Cd1d1	Gm10436///Gm2	Tomm70a	Krtap13-1
Cd1d1	Efcab14	Fam174b	Abcc10
Sox18	Csad	Snhg11	Timm9
Gna11	Spin4	Ift27	6030458C11Rik
Chst1	Col16a1	U2af1l4	Map1lc3b
Mmp12	Usp45	Fntb	Pitpnc1
Col11a1	Usp45	Eif3h	Tshz2
Nr2c1	Rufy2	Prkar1b	LOC100653389
Akap2	Cpa5	Coro2b	Mchr1
Gpr65	BC028528	S100b	Gm15498
Ccna1	Snapc5	Fez2	Gm568
Pglyrp1	Saal1	Sfmbt2	BC028777
Cd5l	Mar-01	Kpnb1	Igf1os
St3gal5	Scfd1	Faf1	Gprc6a
Papolb	Pknox2	Mxd4	Slc8a2
Slc22a21	Obp2a	Rnf157	Rian///Rian///Rian
Rrbp1	Pitpnc1	Morc4	Klra1
Ebi3	Tshz2	Stbd1	[]
Mapk8ip2	Adam34	Col5a1	Slitrk1
Hic1	Zfp937	Ldhb	Klhl7
Gata1	Fsip1	Ttyh2	Sh3bgrl
Bhlha15	Otoa	Aard	Manf
Fasl	Gm568	Clta	Rab2b
Psg16	Ap3s2	Tmem56	Lingo1
Cdh2	Zmym1	Hip1	Eef1d
Ndp	Rian///Rian///Ria	Rps6kc1	Prpf39
Spp1	Klra1	Ptdss2	Strada
Ufm1	Tmem163	Hsd17b11	Bex4
Syt11	Slitrk1	Ncan	Eif1b
F5	Klhl7	Extl3	Klf9
Cadm3	2510009E07Rik	Ctbp2	Klf9
Mettl21a	Sh3bgrl	Rusc1	Gm10340///Gm10406///Gm2888///Gm2897///
1700029P11Rik	Rab2b	Ctrc	Pdlim7
Gpx2	Dynlt1f	Coro2b	Ebf3
Ntng1	Lingo1	Cacng5	Ppm1m
Pde1a	Lman1	Mllt1	Hba-a1///Hba-a2
Lrp5	Eef1d	Hexdc	2810025M15Rik
Sesn3	Usp9x	Hmgn3	Col14a1
2310061J03Rik	Prkd3	Evl	Bola1
Dbndd1	Akirin2	Tpcn1	Spsb1
Zfpm2	Dynlrb1	Morn4	Lphn1
Tenm3	Tceb2	Sacs	Zswim7
Rspo1	Klf9	Eif4ebp1	Snhg5
1600015I10Rik	Klf9	Pi4ka	Rarres2
Zfp235	Map4k2	Zfp787	Basp1
Fhl5	Gm10340///Gm1	Spock2	Ppfia4
Xlr4a///Xlr4b///Xlr4	Pcnp	Pitpnc1	Ppfia4
Mpp6	Pdlim7	Klhdc2	Pnpla2
Osr1	Ift80	Zfp362	Atg7
M1ap	Arxes1///Arxes2	Stx18	Atg7
Trex2	Ppm1m	Unc5b	Lysmd2
Dcn	Hba-a1///Hba-a2	Stmn3	Cand1
Tmprss2	Eif4g2	Tvp23a	Vip
Ces2a	Zcchc3	Elk3	Maoa
Nicn1	Spsb1	Atp1b2	Prpf38b
Pacsin1	Lphn1	A230050P20Rik	Ckmt2
Adssl1	Zswim7	B3galt6	Tha1
Zfp37	Rarres2	Tmem141	Nfam1
Hsd17b1	Ddx10	Cox6b2	Acot6
Aoc3	Basp1	Dgcr2	Gata2
Rpl3l	Chn2	Sod1	Hddc2
Chst7	Ppfia4	Far1	Dck
Pde9a	Ppfia4	Gm21596///Gm6	Parm1
Ift81	Mrpl41	Lpgat1	Rftn2
Jam2	Pnpla2	Lpgat1	Ammecr1l
Fbxo36	Atg7	Ndufaf4	Rbm25
Pde1b	Lysmd2	Zfp651	Hmg20a
Anxa10	Rit1	Ces1d	Tril
Fkbp1b	Ckmt2	Fam105a	Parp6
Car3	Elavl4	Ndn	Slc9a3r2
LOC102643023///M	Slc10a6	Ndn	Slc9a3r2
Cst10	Nckap1l	Vwf	Smpd5
Ptges	Pgp	Rhoc	Ankzf1
Ptges	Nfam1	Slc22a8	Ankzf1///Glb1l
Asb13	Gata2	Chpt1	2610005L07Rik
Rbp7	H13	Bcl2l11	Ptprd
Clec3b	Parm1	B3gnt9	Ggct
St6galnac5	Rftn2	Bmp7	Limd2
Pkd2l2	Tril	Ost4	Nsun4
Dlx1	Slc9a3r2	Ptprd	Gpcpd1
Kcnmb4	Slc9a3r2	Fhod3	Itih5
Gpr12	Smpd5	Pcid2	Zbtb46
Slc2a10	Ankzf1///Glb1l	Cela3b	Mtf1
Mmp7	Ankzf1///Glb1l	Rasgrf1	Sox17
Hist3h2ba///Hist3h	Ptprd	Rpl12	Saal1
Stc2	Limd2	Il17d	5730408K05Rik///Mir5136
Dazl	Toporsos	Snrpn///Snurf	Ikbip
Il27ra	Zbtb46	Rnaseh2c	Dph7
Serf1	Sox17	Ift27	Pus3
Ttc28	Cdadc1	Cebpzos	2610524H06Rik
Unc5c	5730408K05Rik/	B4galt6	Qrsl1
Fmo3	Ikbip	Fam169a	Rbpms
Gdpd3	Pus3	Actr10	Nanog
Pcdhb7	2610524H06Rik	Zbtb45	Tcf7l2
Trps1	Anxa6	Fancf	2610301B20Rik
Leprel2	Klf17	Cdc42bpa	Calcoco2
Chrng	Bcl2l14	Aplp1	Ppap2b
Msh5	Rbpms	Son	Plscr1
Fgf18	Nanog	Chpt1	Rae1
Cntn1	Eny2	Itsn1	Cldn25
Klb	2610301B20Rik	Tfb1m	Dppa4

Trhr	Ptdss2	Taok3	Trappc4
Alpk3	Ppap2b	Scrn2	Wrnip1
Tpm2	Plscr1	Tln1	1810009N02Rik
Emid1	Cep112	Kalrn	Patz1
Mras	Dppa4	2310075K07Rik	DXBay18///Gm14685///Gm5639///Gm5640///(
[]	Mgme1	Tcf7l1	1700017B05Rik
Pagr1a	Trappc4	Tle2	Dtna
Mark1	Dppa2	Vps53	Arl2bp
Fkbp10	Eqtn	Chd4	Srgap2
Prpf19	Patz1	Ppp1r15b	Zbp1
LOC101055763	B930003M22Rik	Zbtb12	Rimklb
Gpr137b	Dtna	Apoa4	Cabyr
Tufm	Eral1	4932441J04Rik	Cdc42ep5
Tmem38b	Pvr	Rab40b	Bcat1
Oxsm	Ahnak	Pag1	Reep6
Oxsm	Ppp6r3	B130024G19Rik	Tmod2
Nr5a2	Tbata	Ltbp4	Grb10
D8Ertd738e	Cebpz	1700019G17Rik	Cyp4f16///Cyp4f37
AA522020	Tlk2	Slc35e4	Aktip
Slc19a1	Ppp3cc	Meg3	Dqx1
AA517023	Rbm4b	Nrep	Me1
Spsb1	Tsnax	Sorbs1	Gusb
Dntt	Tmod2	Cnn3	Scyl1
C76628	Grb10	Fam13c	Glb1l
[]	Cyp4f16///Cyp4f	Klf9	Pcdh18
Tmem191c	Cyp4f16	Pgd	Vps9d1
Tbc1d14	Aktip	Slc7a4	Bcl2l2
Gadd45b	Cep70	Sox11	Sncaip
[]	Aqr	Wnt5a	Phka2
AA517650	Rps15a-ps6	Surf4	Vamp5
AA414993	Dock5	Cdc42ep4	Ctnnb1
Frat1	Gusb	Sep-06	Dbf4
Dmc1	Scyl1	Ttll1	Zkscan5
Fgf2	Eif2ak3	Cnn3	Tpp2
Ptgfr	Ccl27a///Ccl27b/	Ccdc71l	Mvk
Prl3c1	Glb1l	Impa1	Msh3
1700091H14Rik///(	Pcdh18	Pkn1///Ptger1	Pla2g7
Sprr2f	Anxa10	Arl14ep	Zfp943
Gna14	Bcl2l2	Gabra1	4931406G06Rik///4931406G06Rik
Scube1	Igbp1b	Tmsb10	Tcte2
Golt1b	Cd99	Laptm5	Golph3
Higd1b	Vamp5	Rasal2	4933428G20Rik
Il4	Ctnnb1	Gpsm1	Ptk2
Hoxc9	Dbf4	Rab2b	Mbd1
Tbx18	Nt5c3b	Klf9	Mbd1
Bmp8a	Zkscan5	Wipf1	Habp4
Prkg1	Crem	Wipf1	Tpmt
Tmem47	Mrpl38	Pdgfrb	Ube2s
Dcbld2	Brox	Sntb2	Aqr
Acr	[]	Pfn2	Farsb
Grin3b	Msantd3	Trim43a	Sqrdl
Map3k6	Zfp943	Pla2g1b	Mrpl3
Clec4f	Cyb5r3	Tacr3	Eci2
Selp	2810040C05Rik	Slc2a3	Psmd11
Cd209g	Tcte2	Igf2bp2	Snca
4933425L06Rik	Ankrd24	Snx30	Dtd2
Cpeb4	Golph3	Wwtr1	Styx
Ilk	Tmc1	Pcolce	Fbxo3
Zfhx3	Ptk2	Tmsb10	Rragd
Lce1h	Mbd1	Gabbr1	Hspa8
Park2	Mbd1	Cbll1	Syt1
Spock3	Tpmt	Brd2	Slc9a3r2
Gabrd	Farsb	Ccdc88a	Agbl3
Il11	Mrpl3	Xbp1	Mga
Gimap1	Rcbtb2	Marcksl1	Nono
Prss29	Arid4b	Zfp110	Clec2h
Cacna2d1	Arid4b	D2hgdh	Tmem263
Ctnnb1	Pitpnc1	Ctnna1	Twf2
Ubqln2	Rabl3	Tgm2	Ppp1r18
Gtpbp1	Hoxd8	Impad1	Tmod2
B3gnt2	Tmem33	F2r	Wfdc1
Sdc3	Stk35	Trmt1	Scara5
Itga9	Tma16	Cnp	Parva
Dctn4	Auh	Scarb1	Mphosph10
Timp2	[]	Pgd	Lrrk2
Arx	Enah	Creb3l2	Iqgap1
Hs6st2	Hspa8	Igf1	Prelid1
Maged1	Sdccag8	Igfbp4	Stk39
Fmn2	Slc9a3r2	Igfbp4	Hoxa10
Adcy7	Agbl3	P4hb	Gm9757
Celf2	Gm3579	Gh	[]
Nrk	Fndc4	Polg	Lypd1
Nrk	[]	Gnl2	Dpp3
Cxx1c	Nono	Mmp16	Uggt2
Ighmbp2	Clec2h	Gpr85	Dtnbp1
Acyp1	2610042L04Rik/,	Dtnb	Gdi2
Gna12	Tmem263	Bach2	Cntfr
Renbp	Med24	Irs3	Capzb
Gnaq	Cldn25	Glrx2	Gtf2i
Tcf7l1	Tmod2	Exosc4	Gtf2i
Scn1a	Wfdc1	Pitpnm1	Gmfb
Scn1a	Scara5	Alkbh5	Il17b
Ryr2	Fam45a	Lrrtm1	Ralgps2
Pofut1	Rpgrip1	Cntnap2	Kazn
Ncs1	Slc9b1	Eml5	Lig3
Wrn	Parva	Zbed3	Herc4
Ascl1	Mbtps1	Rbmx	Proz
Brdt	Mbtps1	Armcx2	P2ry12
Clcnka	Jam2	Ndn	Bnip2
Gnas	Prelid1	Zfp944	Mto1
Gys1	Stk39	Bgn	Slc7a13
Smyd1	LOC100049077	Btbd17	Irak1bp1

Elmo1	Cxxc5	Rarres2	Hmgn3
Bmp15	Hoxa10	Ergic1	Ypel1
4933417A18Rik	Gm10817	Mea1	Ypel1
Col4a3	Gm17748///Ppih	Rapgef3	Sema3b
Insr	Cd164	Grin1	Itih4
Prlr	Gm9757	Tsr3	Slc6a9
Scamp5	[]	Hsp90b1	Tex9
Otud7a	Lypd1	Ptov1	Rgl3
Lnx1	Sh3kbp1	Rbm5	Hmgcs2
Onecut1	Dpp3	Hhip	Emilin1
Stat5a	Uggt2	Vim	C1qtnf6
Chka	Angel2	Fdps	Phf19
Scn10a	Kcnk10	2610002J02Rik	Mus81
Pcdhb11	Rnf219	Flot2	Pdss1
Rrh	Ica1	Pmf1	Foxa3
Pde3a	Tcrb-J	Ankef1	Pde3a
Hormad1	Cntfr	Fam109b	Ldlr
Vax2	Gtf2i	Cdk16	Sep-06
Zp1	[]	Ccdc102a	As3mt
H2afy3	Ipo5	Cnn3	Hif1a
Astn2	Lig3	Ddx47	Arhgap31
Pcdhb13	Srgap1	Lrrc20	LOC102641289///Tbc1d14
Angpt4	Fmn2	Zbtb20	Artn
LOC100043315	Tmem80	Gm13102///Pram	Neurog3
Arr3	Bnip2	Masp1	Smu1
Il10	Mto1	Masp1	Prdm5
Pcdhb8	Pdzd9	Zdhhc14	Entpd7
Ptger3	Ush2a	Sfrp1	Cyp1b1
Jdp2	Irak1bp1	Pgd	Sh3gl3
Ptdss2	Cbx1	Tnp1	Pcgf6
Prop1	Sema3b	Lasp1	Prrx1
Epdr1	Cyp2d13	Fam227b	Tex13
Nf2	Synj2	Slc4a7	Slc9b1
Abca1	Rgl3	Edn3	Eng
Slc22a5	Emilin1	Cbfa2t3	AK010638///Gm14820///Gm14820
Pde6a	Angptl2	Cacul1	Tlcd2
Cbx5	C1qtnf6	Trp53	Ost4
Kdelc1	Phf19	Calm1///Calm2/	Prrx2
Il18bp	Foxa3	Spata5	Sync
Ncam1	Pde3a	Tnfaip2	Epha1
Ncam1	Pigq	Gmcl1	Bmp7
Gfra1	Neu2	Maea	Fbxw2
Dnaaf1	As3mt	Fdft1	Ak3
Nr1h3	Ap2a2	Rasgrp2	Ms4a10
Socs1	Arhgap31	Rasgrp2	Pde10a
Rilpl1	Artn	Ndn	Nnmt
Spock2	Exosc1	Ampd2	Nup54
Pde6g	Prdm5	Tfpt	4930507D05Rik
Crhr2	Dnaja3	Zdhhc14	2900097C17Rik
Cmpk2	Entpd7	Flywch2	Adam1b
Vsx1	Sh3gl3	Nfat5	Prrg2
Kiss1r	Pign	Ddx17	Psmd7
Klrk1	Prrx1	Mark4	[]
Mthfr	Ttc14	Wipi1	Nkx2-4
Cacna1a	Eng	Nynrin	Mcm10
Hnf4g	Sox5	Ahi1	Tgm2
Cacng3	Ercc8	Tsc22d1	Mela
Cldn9	Sh3kbp1	H2-T10///H2-T22	Cdk5r1
B3galt5	Ikbkb	Pfkl	Cdk5r1
B3galt1	Itgav	[]	Ttc7b
Plagl1	1700074P13Rik	Lin7b	Pi4k2a
Krtap12-1	Prrx2	[]	Tcf7
Cma2///Mcpt9	Sync	Enpp3	Edil3
Magea1///Magea2	Spr	Ddit4l	Epb4.1l2
Ammecr1	Epha1	Mmp2	Epb4.1l2
Ifna1	Bmp7	Slc9a3r2	Reep1
Col2a1	Pdcl2	Slc9a3r2	Fli1
Bfsp1	Nnmt	Sf3b5	Etnk1
Bdkrb1	4933429H19Rik	Sumo3	Rpl41
Olfr68///Olfr69	Sec62	Wnt5b	Acsl4
Vmn1r53	2900097C17Rik	Meg3	Mbp
Olfr1508	Adam1b	Ppp2r4	Marveld1
Ifnab	Mdn1	Myadm	Cdc37l1
Ifna5	Nfatc4	Vps9d1	Sox4
Pdia4	Elavl2	Gpd1	Inpp5a
Col5a2	Nacc1	Qsox1	Coq10a
Slc2a8	Rptn	Lats2	Pld4
Vim	Tgm2	Rnaseh2c	2210016L21Rik
Myo10	Pnlip	Atg12	Cry1
Ctsk	Prss2	Uhrf1bp1	Trio
Spz1	Ttc7b	Myrf	S100pbp
Adamts5	Tcf7	Crxos	Nisch
Nfic	Edil3	Slc8a1	Rhbdd3
Dbh	Epb4.1l2	Slco5a1	Ube2o
Tagln3	Epb4.1l2	Cbfa2t3	Ldb3
Etv1///Gm5454	Reep1	Zfp771	Calr
Rgs17	Fli1	D18Ertd232e	Dusp9
Dusp2	Etnk1	Zfp518b	Abat
Hfe	Etnk1	C76132	Atp2b2
Scg2	Acsl4	Dpp4	Spag5
Defa5///Gm10104/	Gns	C76554	AU020206
Kcnj9	Dab2ip	C79242	Tufm
Sh2b2	Prss2	Kalrn	Cdh5
Fam126a	1190002N15Rik	C78878	Rrnad1
Tnfrsf21	Gm6682///Tuba1	C79122	Tle1
Sec16b	Fam21	Dhrs7b	Cerk
Tbl1xr1	Inpp5a	[]	Repin1
Pafah1b2	Coq10a	AA511254	Mafg
Cdh11	Pcbp4	D15Ertd30e	Mthfr
Bmp6	Timp2	[]	Atg9b
Ing3	Pld4	C80060	Tcf4
Zfp191	Ppp1r3c	C77691	Tcf4

3632451O06Rik	Cry1	DXErtd242e	Mettl7a1///Mettl7a2
Fut9	S100pbp	Zc2hc1c	Mettl7a1
Reck	Ldb3	C78532	Khdc1b
Trip4	Mobp	[]	Ece1
Saa1	Sun2	[]	Fermt2
Atoh7	Ppp4r2	[]	Fermt2
Adam21	Abat	C78948	Prss35
Bcl2a1a///Bcl2a1b/	Syt17	D7Bwg0826e	Atpif1
Lyz1	Peg3	Arl5a	Tmem151b
Saa3	Zik1	Maf	Rrp12
Hoxc5	Pla2g1b	Auh	Gba2
Serpinh1	Cdh5	[]	Fam174b
Col1a2	Fermt3	[]	Snhg11
Dclk1	Ccdc50	C78441	Eif3h
Lipa	Pdap1	D1Ertd75e	Eif3h
Cfh	Repin1	Npr3	Prkar1b
Gpr137b	Mafg	Tbc1d24	Coro2b
Tmem167	Arf4	LOC102640476//	Uqcr10
Src	Mthfr	Tm4sf1	S100b
Tgfb2	Rufy1	[]	Fez2
Tgfb2	Tcf4	Rln1	Sfmbt2
Hdgfrp3	Tcf4	AA408251	Kpnb1
Igfbpl1	Khdc1b	Ctr9	Map3k14
Tceal3///Tceal5///T	Khdc1b	D5Ertd102e	AW112010
Gadd45b	Fermt2	Trim27	Gbp7
Cacng4	Fermt2	Necap1	Tgif2
Gpc3	Ppp2r5c	Uba1	Spred2
Meis1	Ndufs8	Gusb	Crybg3
Xdh	[]	Slc19a1	Rnf157
Rnf151	Fam174b	Pald1	Samhd1
Slc29a3	Ibtk	Enpp2	Arhgap39
Ror1	Snhg11	Igf2	BC003331
Hcn3	Ift27	Sdc1	Col5a1
Bcl2l2	U2surp	Rab7	Mef2d
Fhl4	Eif3h	Spt1	Plk3
Trpc4	Prkar1b	Ehd1	Ldhb
Syt13	Coro2b	Cd24a	Ttyh2
Itm2a	Uqcr10	Fkbp1a	Papss2
Zfp474	S100b	Sfrp2	Aard
Pex5l	Fez2	Lasp1	Cpd
Aars	Sfmbt2	Cox8a	Hip1
Ggt7	Kpnb1	Lox	Rps6kc1
Esyt1	AW112010	Ldhb	[]
Vash2	Rnf157	Hmox1	Slc38a10
Aacs	Cox17	Bcl7b	Nrf1
Fbln1	Aqp4	Clmp	Hsd17b11
Pink1	Atcay	Clmp	Ctbp2
Cyhr1	BC003331	Surf4	Hlf
Celf2	Dusp3	Slc29a2	Hlf
Lrrc49	Col5a1	Fstl1	Coro2b
Nrros	Mef2d	Klhl13	Sema5a
Aen	Ldhb	C1qbp	Cacng5
Crabp2	Ttyh2	Tspan4	Pkig
Scara5	Slc4a1	Pold2	Hmgn3
Rerg	Papss2	Plrg1	Kctd12///Mir5130
Jkamp	Wdr77	Hsd17b10	Slc25a4
Pxk	Anp32a	Ebf1	Evl
Fabp4	Hip1	Slc1a1	Hspb7
Frmd6	Sema4d	Pcsk2	Tpcn1
Tram1l1	Hsd17b11	Pycr2	Dnajc27
Ntmt1	Cldn3	Cmtm3	Sacs
Aif1l	Lrrc8a	Plin2	Eif4ebp1
Dclk1	Ddrgk1	Smoc1	Pi4ka
Gm11787///Lyn	Ctbp2	Bgn	Tmem27
Abhd14b	Ero1lb	Rnps1	Pitpnc1
Zfp521	Ids	Crabp1	Tvp23a
Plac8	Sema5a	Adam3	Ugcg
Spon1	Cacng5	Pex19	Elk3
Tmem119	Vamp4	Adprh	Leng8
Polr3gl	Foxo3	Drg1	Eomes
Alg1	Hexdc	Cfl1	Sox3
Commd5	Cox17	Asl	A230050P20Rik
Ugt3a1	Kctd12///Mir513	Rpn1	N6amt2
Aamdc	Mtdh	Ccng2	Irf3
Dnajc24	Slc25a4	Stx1a	Cox6b2
Pcsk5	0610005C13Rik	Dctn6	Dgcr2
Dhx58	Rab6b	Lgals3bp	1110004E09Rik
Slit2	Evl	Mmp14	Kif11
Chodl	Phf2	Pofut2	Tti2
Antxr1	Hspb7	Sparc	Psma6
Gca	Tpcn1	Sfrp1	Psma6
Thnsl2	Neo1	Tln1	Lpgat1
Ifnar2	Morn4	Eid1	Fam105a
Prr5	Dnajc27	Wfs1	Ndn
Plxnd1	Sacs	Sema3b	Ndn
Tmem53	Ccl27a	Aspn	Vwf
Cadps2	Pi4ka	Frzb	Mip
Ushbp1	Cela3b///Gm130	Sardh	B3gnt9
Mef2c	Spock2	Nbl1	Fcgr2b
Mef2c	Pitpnc1	Asb6	Bmp7
Serpina1b	Klhdc2	Pcolce	Adora1
Tmem82	Unc5b	Vps28	Kctd17
Steap1	Dnajc27	Ripk3	Ptprd
Chil1	Tvp23a	Irf8	Phka1
Krt84	Ugcg	Acvr1	Dab1
Gm16340///Ifi203/	Elk3	Ehbp1l1	Rasgrf1
Zbtb20	Crebrf	Nid1	Hs3st3a1
Ppp6r2	Tsr2	Fbp1	Herpud1
Tulp1	Eomes	Vars	2610528A11Rik
Mmgt2	Sox3	Olfml3	Rpl12
Sphk1	A230050P20Rik	Chst12	Abcc5
Tmprss11d	Gjc2	Med20	Il17d

A530016L24Rik	BC023829	Adck4	Rnaseh2c
Amelx	B3galt6	Tspan6	Fgf14
Lmbrd1	Irf3	Slc16a7	B4galt6
Mrpl15	Cox6b2	Ptprcap	Fam169a
Rprd1a	Far1	Nphp1	Csprs///Gm15433///Gm2666///Gm7609///LOC
Ighg	Psma6	Thbd	Eif4e2
Airn	Lpgat1	Ttc1	Cdc42bpa
Armc1	Lpgat1	Rsrp1	Aplp1
Fam161a	Pyhin1	Acy3	Son
Alas2	Pyhin1	Klc1	Elavl3
Srxn1	Ndufaf4	Crat	Cd300a
Amelx	Stau1	Sdc2	Gbp2
Cant1	Zfp651	Rassf3	Atp1a1
Ednra	Fam105a	Lbp	Atp1a1
Fgf12	Ndn	Prlr	Tfb1m
Evc	Ndn	Fam13c	Dpep1
Cmtm7	Vwf	Bid	Zc3h3
Oprm1	Rhoc	Upp1	LOC102643254///Rps6///Rps6-ps4
Oscar	Chpt1	Mlec	Taok3
Prpf4b	B3gnt9	Rsrc1	Gmppa
Pik3r1	Bmp7	Gtf2h4	Nav2
Wbp1l	Kctd17	Col6a1	Sugp2
Znhit1	Ptprd	Crtap	Meg3///Meg3
Apobec1	Phka1	Wisp1	Kalrn
Flt1	Pcid2	Wisp1	Fcho1
Lamc3	Sephs2	Bex1	Slc6a17
Slc20a2	Cela3b	Pygm	Gm20559
Pcdha1///Pcdha10,	Rasgrf1	Rhoc	E2f8
Tpcn1	Il17d	Lpar1	Mbp
Ddx60	Snrpn///Snurf	Nampt	Simc1
Nfatc2ip	Vimp	Nt5c2	Tle2
F11	Ift27	Ccs	Otud5
Tfpi	Fgf14	Cd2bp2	1600014C10Rik
Tfpi	B4galt6	Spata5	Ptrh1
Tom1l2	Ctps2	Wdr12	2700079J08Rik
Adam22	Fancf	Fam114a1	Ppp1r15b
Suv420h1	Cdc42bpa	Enpep	Zbtb12
Scrn2	Smarcb1	Mtch2	Rps10
Btn1a1	Aplp1	Lrp1	Tfap2c
Nox4	Chpt1	Cacnb3	1700097N02Rik
Prlr	Itsn1	Speg	Kdelc1
Nebl	Itsn1	Arfgap2	Ptgs1
Lmnb2	Atp1a1	Pvrl3	Rab2a
Prlr	Dpep1	Emc8	Rps24
Csn3	Taok3	Kcnk1	Rab40b
Notum	Nlk	Heph	Pag1
Trim30a	Nav2	G0s2	Bola2
Nxnl2	Mfsd4	Cxcr4	B130024G19Rik
Arhgap6	Meg3///Meg3	Rngtt	Ltbp4
AF366264	Kalrn	Gcdh	Rundc3a
Eda	AI118078	Lamtor1	Gm10193///Zfp706
Sema6c	Slc6a17	Snapc2	Ceacam11
Mx1	Csn1s2a	Sep-04	Sorbs1
Ubxn10	1110054M08Rik	Ctsb	Cnn3
Sec14l1	Ndrg4	Cp	Fam13c
Ace	2310075K07Rik	Cp	Klf9
Fgfrl1	Tcf7l1	Calb1	Pgd
Hyou1	Tmem71	Rangrf	Ogt
Nfat5	Tle2	Galns	Sox11
Rftn2	D830044I16Rik	Lor	Wnt5a
Mrgpra4	[]	Plek	Gm5451///Rpl9
Vrk2	Rnf207	Plek	Cdc42ep4
H2-D1	Dstyk	Ap3m2	Scyl1
Srpx	Vps53	Rbp1	Sep-06
Fcgr2b	Pdxdc1	Col15a1	Cnn3
Tnfsf11	Tfap2c	Pml	Snca
Cabyr	Ipo4	Fyn	Il7
Igh-VJ558///Ighg///	Kdelc1	Slc35b1	Tacc3
Vegfa	Zfp260	Gm16340///Ifi20	Sfpq
Loxl1	Rps24	Runx1t1	Rasal2
Casd1	H1fx	Cd200	Gpsm1
Irx1	Map3k3	Aldh1a3	Rab2b
Mapre2	Pag1	Cyp2f2	Adcy3
Mapre2	[]	Elk3	Adcy3
Epha7	Spry2	Cyp11a1	Klf9
Ldb3	Inpp4a	Ptgis	Wipf1
Rbm14	Lonp2	Wnt5a	Wipf1
Calca	1700019G17Rik	Ube2j1	AK077428
Gm8539///Zbtb8os	Riok2	Angpt2	Pdgfrb
Vamp7	Gm10193///Zfp7	E2f6	Rbm14
Atp10a	Gm10193///Zfp7	Kank3	Sntb2
Igf1	Hbb-y	Cdo1	Lyz1
Baalc	Clcc1	Cxcl13	Sh3bgrl
Prkar1a	Sorbs1	Icam2	Pla2g1b
Col4a1	Wnk1	Ltbp1	Rbm14
Zbtb8a	Cnn3	Zhx1	Skp2
Thap7	Klf9	Evc	Snd1
Dcun1d1	Luc7l2	Hp	Mapk8
Zbed3	Luc7l2	Gtf2e2	Slc2a3
Chst15	Arhgef40	Cpxm1	Api5
Arl16	Psma1	Sep-15	Phyhipl
Igfbp5	Psma1	Mrps34///Nme3	Timp2
Pth2r	Slc7a4	Thop1	Lats2
Snrpb2	Acp2	Ppap2b	Igf2bp2
Tmem175	Sox11	C1qtnf1	Gm15393///Gm15393
Slc6a1	Wnt5a	Cd302	Wwtr1
H6pd	Ttll1	Twist2	Rbpms
Chpf2	Cnn3	Gng11	Pcolce
Jade1	Impa1	Chd3os	Gabbr1
Meg3	Il7	Cxxc5	Ccdc88a
Pde2a	Doc2a	S100g	Xbp1
Mndal	Sfpq	Coprs	D2hgdh

Medag	Tmsb10	Klk6	Dcun1d5
Prickle1	Pcm1	Myo1b	Clcf1
Col6a2	Cacnb4	Myo1b	Clcf1
Nbea	Gpsm1	Ina	Ctnna1
Mtmr9	Atp7a	Rom1	Tgm2
Cidec	Rab2b	Fndc4	Exosc10
Tenc1	Gm15776///Gng	Gm10349///Gm9	Adrb2
Cubn	Klf9	Hexa	F2r
Zfand3	Wipf1	Ifnar1	Uba7
Farp1	Wipf1	Rnf128	Xpa
Tmem106b	Nsmf	Crem	Aldh18a1
Slit3	Pdgfrb	Hsd11b1	Cela3b///LOC102641402
Lrp2	Pfn2	Naga	Aldh18a1
Pvt1	Lyz1	Eef1e1	Krt13
Iqsec1	Sh3bgrl	Josd2	Coq3
Mxra8	Trim43a	Rab4a	Ddx39b
Ccdc85a	Becn1	Pcbp4	Gtf2h4
Adamts7	Rapgef3	Pcbp4	Scarb1
Ldlrad4	Pla2g1b	Gli1	Pgd
Lmod2	Snd1	Sec22b	Slc52a2
Ifi204///Ifi205///M	Slc2a3	Apcdd1	Creb3l2
Ifi205///Mnda	Prm1	N6amt2	Aldh9a1
2810459M11Rik	Phyhipl	Tex40	Igf1
Rai2	Zbed3	Hdac2	Igfbp4
Zfp444	Lats2	Ces1d	[]
Malat1	Igf2bp2	Ccdc91	Trim12a
Epha7	Mrpl11	Gjc1	Clu
Dnm3os///Mir214	Foxm1	Ccdc127	P4hb
Enpp3	Tmx1	Txnrd2	Unc13a
Wipi1	Rbpms	Pard6a	[]
Spag4	Pcolce	Gpx3	Serinc1
Fes	Pdlim1	Socs2	Gm7040///Prl2c2///Prl2c3///Prl2c4///Prl2c5
Igk-V28///Igkc///Ig	Tmsb10	Dtymk	Sgcg
Heatr1	Gabbr1	Arih2	[]
Pclo	Eif3m	Zfp90	[]
Lpar4	Ccdc88a	Cd1d1	Polg
Tfpi	Xbp1	Cd1d1	Gnl2
Gm21451///Loxl2	Nmd3	Spic	Mmp16
Tnfsf12///Tnfsf12T	Hnrnph1	Sox18	Gcnt2
Atp8b2	Clcf1	Ostc	Gpr85
Atp11c	Clcf1	Chst1	Aldh18a1
Usp9y	Tgm2	Col11a1	B3galt2
Sox5	Cela3b///LOC102	Sigirr	Snhg1
Cd247	Cnp	Akap2	Cux1
Epb4.1l2	Pttg1ip	Pdlim3	Clu
[]	Bscl2	Pdc	D1Pas1
Ofa	Ovca2	St3gal5	Rad23a
[]	Coq3	Slc22a21	Exosc4
[]	Ddx39b	Hic1	Pitpnm1
Rprd1b	Fgd1	Rnf138rt1	C1qb
Gm9780///Plac9a/	Scarb1	Cdh2	Alkbh5
Adamts4	Slc52a2	Actrt2	Omt2a
Ssbp1	Igf1	Ndp	Lrrtm1
Pcbd2	Igfbp4	Spp1	Cpeb3
Lrrtm1	Igfbp4	F5	Igfbp7
3110007F17Rik	Gdap1	Mettl21a	Mtch2
Myl1	Rnpc3	Gpx2	Zbed3
Ap1s2	Unc13a	Ntng1	Bace2
Svop	Lrrfip1	Cnp	Rbmx
Tmcc2	[]	Pde1a	Armcx2
Rab2b	Sp4	Slc7a13	Ndn
Myl9	Xiap	Sesn3	Map4
Prpf39	[]	F10	Bche
Rps4l	[]	Zfpm2	Bgn
Lepre1	Fubp1	Tenm3	Btbd17
Creld2	Phf20l1	Rspo1	Rarres2
Hba-a1///Hba-a2	Gnl2	Gm13363///Ptp4	Lox
Glipr2	Gnl2	Fads2	Ergic1
Il6st	Mmp16	Sostdc1	D530037H12Rik
Pou6f1	Zbtb14	Ccdc34	Mrpl42
Ppfia4	Ywhaq	Osr1	Dnm2
Med12l	Gpr85	Pax1	Rnf114
Prkce	B3galt2	Dcn	Xpr1
Rian	Atf7	Dnajc3	1110057K04Rik
Pgp	AU021092	Nicn1	Gja1
Pitpnc1	Rac1	Adssl1	Ap1s2
Pitpnc1	Cux1	Hsd17b1	Rmdn1
Rftn2	Anxa2	Chst7	Hsp90b1
Slc9a3r2	Fmod	Pde9a	Gm9222
Cntn1	A230046K03Rik	Jam2	Ptov1
Taf1d	Pcbp3	Dscam	Ybx1
Prpf31	Pitpnm1	Pde1b	Gm5481///Gm6109///Gm6570///Gm7429///Rp
Batf3	Omt2a	Med9	Mrpl11
Col22a1	Eif4ebp2	Ptges	Hhip
Polr3e	Lrrtm1	Ptges	Vim
Plscr1	Foxh1	Bst1	Zdhhc14
Oasl2	Igfbp7	Foxi1	Coq10a
Wbscr25	Il10ra	Asb13	2610002J02Rik
Eqtn	Zbed3	Rbp7	Flot2
Pgm1	Pofut2	Clec3b	Ankef1
Ankrd33b	Bace2	Gm20737///Gm2	Hist1h1c
Khdrbs3	Armcx2	St6galnac5	Tram1
Slamf7	Ndn	Gpr12	Pcsk5
Far1	[]	Mok	Ikzf4
Cd99	Bgn	Slc2a10	Ccdc102a
Rad23a	Rarres2	Mmp7	Slc22a6
Mbd1	Lox	Hist3h2ba///Hist	Cnn3
Serpinf1	Ergic1	Stc2	Pfdn5
Ndst3	Bcl2a1a///Bcl2a1	Mbd4	Slc13a3
BC051212	D530037H12Rik	Reg3a	Pttg1
Ergic2	Zfp787	Gzmm	Rbm39
Pi16	Rnf114	Kpna1	Rilpl1

Zbed3	Grin1	Zfp467	Zbtb20
Gadd45g	Sep-07	Qpctl	Inpp5k
1700120B22Rik	Rmdn1	Gadd45a	Gm13102///Pramel4
Ptrf	Gm9222	Ttc28	Nos1
Slc37a3	Rbm5	Cd93	Arhgef40
Ptgfr	Mrpl11	Unc5c	[]
Gm9706	Hhip	Eda	Tor2a
Cdk14	Ankrd54	Gdpd3	Fancf
Ercc6l2	Vim	Trps1	Masp1
Lig3	Fdps	Leprel2	Masp1
Fmo2	Coq10a	Tmem100	Tnni2
Ubxn8	Rhbdd2	Znrf4	Gm9840///Rbx1
Zfp142	Pcsk5	Pbx1	Pgd
Prmt3	Cited4	Kcnh2	Lasp1
Chpf	Tpp2	Fgf18	Lasp1
Lmo2	Ikzf4	Efnb2	Fam227b
Cxxc1	AA545190	Myo1c	Bace2
Slc9b1	Cdk16	Nkain1	Gja1
4921524J17Rik	Ccdc102a	Msx2	Aplnr
Stk31	0610037L13Rik	Krtap1-5	Mmd2
Ccnc	Cnn3	Cntn1	Arf2
C330007P06Rik	Pfdn5	Cyp2g1	Ptpn1
Ercc8	Pfdn5	Alpk3	Slc4a7
Ikbkb	App	Cdc42	Gbp6
Gpa33	App	Tpm2	Trim8
Cyba	Fam120b	H2-DMb1///H2-I	Edn3
Kcnip1	Rilpl1	Emid1	Fnbp4
Tspan12	Zbtb20	Pcdhb20	Cbfa2t3
Dpysl3	Khdrbs1	Nkap	Pklr
Cpa2	5830428H23Rik	Tcf15	Osbpl6
Iffo1	Chd4	[]	Irf3
Iffo1	Masp1	C79490	C80068
Tmem229b	Masp1	[]	Thyn1
Etnk1	Clps	Hdlbp	Dpysl3
Thra	Gm9840///Rbx1	Pagr1a	[]
Timp2	Bace2	Adcy9	[]
Pten	Slc4a7	Mark1	Slc20a1
Dusp9	Gm12942///Zmy	Fkbp10	Calm1///Calm2///Calm3
Ficd	Csn1s2a	C920021L13Rik/,	Eftud2
[]	Edn3	[]	Arg2
Apcdd1	Cbfa2t3	Cct3	Spata5
Rpl35	[]	Tufm	Cldn1
Pkdcc	Trim30d	Ccnb1///Gm559	Tnfaip2
Slc52a2	Osbpl6	Tubb2a-ps2	Irak1
Upk3b	Adam15	Nr5a2	Hdgfrp2
Amot	Dpysl3	4930453N24Rik	Tmem206
Ngfr	Mtch2	[]	Fdft1
Gm561	Spata5	Spsb1	Gm3362///Gm6177///Gm8112///Gm8137///Gr
Rnf157	Ip6k1	Ssx2ip	Rasgrp2
Atcay	Tnfaip2	[]	Rasgrp2
Ntn1	Heyl	Tmem191c	Ampd2
Slc25a4	Map3k12	[]	Tgm2
Ahsg	Sccpdh	Tap1	Gja1
Ckb	Hdgfrp2	Fgf2	Gja1
Atp1a2	Hdgfrp2	Acan	Pitpnm1
Nlgn2	Fdft1	Ptgfr	Flywch2
Ppp2r3d	Fdft1	Sntb2	Gmppb
Pitpnc1	[]	St8sia2	Ddx17
Cml2	Gm3362///Gm6	Slco1a1	Adcyap1r1
Rprd2	Rasgrp2	Ephb4	Wipi1
Rgs16	Rasgrp2	Scube1	Nynrin
Fcgr2b	Ndn	Nr0b2	A930012O16Rik
Masp1	Ampd2	Golt1b	Tsc22d1
Bai1	Pim3	Il4	Gm4120///Gm4983///Gm9292///LOC10105582
Dnd1	Cds2	Hoxc9	[]
Cp	Fkbp1a	Vpreb1	[]
Fam131b	Fmod	Atp6v0a2	Rhox4b
Sep-04	Pitpnm1	Tbx18	[]
Lgals1	Pm20d1	Prkg1	Enpp3
D430019H16Rik	Flywch2	Efcab2	Lasp1
Adcy2	Iqcf3	Tmem47	Osbpl9
Gpr133	Nfat5	Timm9	Mmp2
3110057O12Rik	Prl3d1///Prl3d2	Galc	Slc9a3r2
Syne1	Ddx17	Map3k6	Dmtf1
Col1a1	Recql	Selp	Meg3
Cnn3	Zfp36l2	Pitx3	Slc13a3
Csn1s2a	Wipi1	Csnk1d	Med1
C1qtnf1	Ahi1	Tsen15	Bpifb1
Hoxa9	Cdc73	Ilk	Lyz1
Tspan17	[]	Zfhx3	Slc52a2
Sec62	Cops5	Cacna1f	Lct
Mterfd3	Tnrc6a	Fgf14	AI506816
Mtcp1	[]	Lce1h	[]
Gm1976	Gpr137b///Gpr1	Krtap9-3	Zfp865
Taok3	Enpp3	Mcpt8	Pramel7
Ndn	Lasp1	Spock3	Fgf11
Vasn	Gm5595	Il11	Ttc39b
Scarb1	Ddit4l	Nqo2	Slc8a1
Lnx1	Flii	Gimap1	Abhd11os
Gprin1	Mmp2	4933421I07Rik	Syt2
Tspan31	Grsf1	Cacna2d1	D9Ertd26e
Pomc	Slc9a3r2	Ctnnb1	Slco5a1
Nanos1	Slc9a3r2	Ubqln2	Cbfa2t3
Lrrtm1	Sumo3	Gtpbp1	Cbfa2t3
Rspo2	Prm1	B3gnt2	Eif2s2
Slc2a3	Meg3	Sdc3	D18Ertd232e
Tgm2	Slc13a3	Itga9	C76132
Chpt1	Myadm	Dctn4	Dpp4
Adrb3	Nsmf	Usp9x	Erlin1
Chrna3	Bpifb1	Timp2	C530030P08Rik
Rbpms	Herpud2	Tmem59	Dpf3
Ceacam11	Lyz1	Hs6st2	[]

Adamts4	Twf2	Idh2	D9Ertd306e
Matn2	Lats2	Hira	C79242
Xpa	A230046K03Rik	Apc	[]
Marcks	Slc52a2	Enc1	Kalrn
Dtna	Ceacam11	Maged1	C78878
BC089491	Nsmce4a	Fmn2	C79122
Rnf112	Gm21596///Gm6	Fmn2	[]
Tsc22d1	Lct	Adcy7	[]
Itgb5	Myrf	Baz1b	D8Ertd67e
[]	D13Ertd608e///L	Celf2	C79130
Acsm2	Pramel7	Nrk	C77691
Itgb5	Fgf11	Nrk	DXErtd242e
Fgf17	2310010J17Rik	Cxx1c	C1galt1c1
Ap1s2	Slc9b2	Etv5	[]
Rbm5	Abhd11os	Angptl2	Zc2hc1c
Notch4	H2afj	Nolc1	[]
Slc52a2	Psmd11	Srprb	[]
Vim	Syt2	Ighmbp2	D8Ertd107e
Pex6	Slco5a1	Ranbp17	Eif2d
Adcy7	Cbfa2t3	Gna12	[]
Lasp1	Cbfa2t3	Hoga1	Gm6211
Gsn	D18Ertd232e	Evl	D8Wsu26e
Arhgap6	Dpp4	Renbp	C77815
Shroom1	C76554	Gas2	C77137
Limd2	Arhgap29	Gnaq	[]
Dner	D2Ertd127e	Supt20	Dcpp3
Cnn3	D9Ertd306e	Tcf7l1	Arl5a
Ubxn10	C79242	Scn1a	Fancm
Zic4	C77609	Ptprg	Maf
C1ra///C1rb	C78878	Xpr1	Auh
Mical1	Dhrs7b	Cd38	Egfl8
Ccdc53	[]	Gopc	Mrpl38
Ewsr1	AA511254	Wrn	Rpp30
Atxn10	[]	Brdt	AI596198
Zfp574	C79709	Cux2	C78441
Trim30a///Trim30d	[]	Sh2b3	D1Ertd75e
Osbpl6	C80060	Gnas	D17892
Tcfl5	[]	Zfp106	Sash1
Prokr1	C79130	Hcn1	A430104N18Rik///Mir142
1700097N02Rik	C77691	Mynn	Ubap2l
Gsn	DXErtd242e	Fgf22	Taok3
Nnt	[]	Pde7b	Npr3
Wipi1	Habp2	Ccl28	Tbc1d24
Eya4	[]	Apaf1	Tbx3
Acta2	Sbf2	Col4a3	LOC102640359
Marcks	Zc2hc1c	Prlr	Tfpt
LOC102640192	Irs2	Rsph6a	LOC102640476///Rnf2
Wwp2	C78532	Otud7a	[]
Wrap53	E230006M18Rik	Lnx1	B4galt3
Ezr	Nucks1	Cer1	Fndc7
Serpinh1	Nucks1	Stat5a	Bub3
Armcx2	D8Ertd107e	Smad9	[]
Cog1///LOC102641	D7Ertd187e	Pde3a	Image:619641
Gpm6a	AA407107	Il6	AA408251
Ergic3	[]	Vax2	Ctr9
Rasal3	Gli2	4930591A17Rik	Necap1
She	D3Wsu167e	H2afy3	Klk1
Akap17b	C80425	Zfp354c	Serinc1
[]	D8Wsu26e	5730493B19Rik	Kitl
LOC102641785///T	[]	LOC100043315	Gusb
[]	BC053393	Vmn1r187///Vm	Rit2
[]	Arl5a	Ptger3	Enpp2
Naalad2	Neu1	Jdp2	Tnfrsf19
AI450241	Maf	Ptdss2	Igf2
C80360	Zfyve16	[]	Vcam1
Tmem109	Auh	Msn	Siah2
Iqgap2	Egfl8	Epdr1	Ehd1
Sema7a	C76614	Nf2	Hn1
Cdr2l	Gm11944	Bace1	Herpud1
Nxph1	Zfp398	Pip5k1b	Pnliprp2
Usp42	Ttl	Mrpl19	Sfrp2
AA517562	[]	St3gal3	Sav1
Thyn1	Mcm9	Anp32a	Slc22a17
Aldh7a1	AI596198	Slc48a1	Syngr3
Adprh	1110008P14Rik	Stag1	Lox
Sfrp1	[]	Capn6	Mbtps1
Osbpl1a	D1Ertd75e	Pcyt1a	Clmp
Itpr1	Cdca3	Ncam1	Clmp
Fbn1	2610001J05Rik	Ncam1	Slc29a2
Pkd1	Ankhd1	Gfra1	Fstl1
Prl7d1	Vps13c	Socs1	Uchl1
Guca2b	C76336	Rilpl1	Klhl13
St3gal5	Fam46c	Rcan3	Tspan4
Zfp105	[]	Spock2	Cela2a
Cmtm7	Tbc1d24	Cbl	Crmp1
1700094J05Rik	Tbx3	Sppl2a	Mmp9
Itga9	LOC102640359	Crhr2	Ebf1
Timp2	Gnao1	Ptpn12	Slc1a1
Nrg3	Inca1	Sox3	Ick
Akt3	Rnf2	Itga2	Pcsk2
Fut8	Nmrk1	Dpysl2	Cmtm3
Chst3	Ing4	Foxn1	Akr1b3///Gm6644
B4galt6	Huwe1	Chst11	Bgn
Anxa3	Mipol1	Rab17	Crabp1
Sh3kbp1	Sema6d	H1f0	Actn2
Esam	Stk11	Tlx1	Cpq
Armcx3	AA408251	Pvt1	Cfl1
Dnm1	C76533	Klra15	Rpn1
Hspa12b	[]	Defb2	Stx1a
Ccdc117	C78228	Tex13	Fscn1
Psip1	Necap1	Vmn1r51	Lgals3bp
Arhgef10l	Ctps2	Cd19	Pofut2

Gabrg1	Bpgm	Bfsp1	Sparc
Fbln7	Gusb	Akp3	4632428N05Rik
Zdhhc1	Enpp2	Cyp11b2	Sema3b
Obsl1	Igf2	Olfr68///Olfr69	Aspn
Tmem160	Lcp1	Olfr1508	Frzb
Pde4dip	Vcam1	Vmn1r46	Pcolce
Arrb1	Fkbp1a	Ifnab	Irf1
2700089E24Rik	Pnliprp2	Col5a2	Chkb
Ednra	Sfrp2	Qtrt1	Ripk3
Trim46	Syngr3	Rhoa	Irf8
Ints8	Cox8a	Aebp1	Hsd3b1
Sep-11	Lox	Gm3837///Pdcd!	Acvr1
Fastk	Ldhb	Vim	Kcnab1
Atox1	Gm2a	Gng10	Nid1
Traf4	Clmp	Ctsk	Olfml3
S1pr3	Clmp	Adamts5	Chst12
Cckbr	Fstl1	Rgs7	Slc39a8
U90926	Klhl13	Nfic	Lrrfip1
Klhdc2	Tspan4	Dbh	Tead2
Col9a3	Cela2a	Atrip///Trex1	Sars2
Mc5r	Reg3b	Col9a2	Thbd
Ercc2	Mmp9	Itgb2	Prl8a9
Slc35c2	Ebf1	Rag1	Sirpa
Limd2	Tuba3a///Tuba3l	Zfp143	Acy3
A630038E17Rik	Rab6a	Tagln3	Klc1
	Cmtm3	Etv1///Gm5454	Sdc2
	Smoc1	Prss3	Lbp
	Bgn	Dusp2	Prlr
	Crabp1	Slc7a2	Fam13c
	Adprh	Jarid2	Bid
	Cpq	Sh2b2	Upp1
	Tmem30a	Fam126a	1700071K01Rik///Phb
	Vapa	Hs2st1	Prl4a1
	Dnajc7	Hs2st1	Gtf2h4
	Dctn6	Tnfrsf21	Crtap
	Tm9sf3	Sec16b	Wisp1
	Slpi	Pno1	Bex1
	Wap	Syncrip	Pygm
	Sparc	Keap1	Rhoc
	Eid1	Pafah1b2	Lsm4
	4632428N05Rik	Cdh11	Hipk2
	Hps1	Bmp6	Psmb10
	Wfs1	3632451O06Rik	Fam114a1
	Aspn	Fut9	Enpep
	Tmed4	Mos	Pole
	Frzb	Fabp7	Lrp1
	Sardh	Reck	Mvd
	Asb6	Rhpn1	Speg
	Pcolce	Prss28	Irak1
	Serpini1	Kat2b	Pvrl3
	Irf8	Ptch1	Rnf25
	Top2b	Nrep	Camk2b
	Ehbp1l1	Serpinh1	Podxl
	Nid1	Ncbp2	Kcnk1
	Olfml3	Col1a2	G0s2
	Chst12	Fancc	Cxcr4
	Lrrfip1	Dclk1	BC094435///Ccrn4l///Cog6///Sgip1
	Serpina6	Calm1///Calm2/,	Cish
	Efhd1	Hgsnat	Tle6
	Ptprcap	Cfh	Sep-04
	Tead2	Cd36	Cpa3
	Thbd	Cd36	Cp
	Rsrp1	Ap2m1	Cp
	Acy3	Cox16	Lor
	Sdc2	Brd3	Fbxo32
	Rassf3	Tmem167	Plek
	Lbp	Eif4e	Rbp1
	Prlr	Ppib	Col15a1
	Prl4a1	B4galt6	S100a9
	Zfp346	Src	Fyn
	Col6a1	Aptx	Gjb1
	Crtap	Tgfb2	Mog
	Wisp1	Tgfb2	Slc35b1
	Mmp17	Hdgfrp3	Gm16340///Ifi203///LOC100862473
	Msx1	Prkca	Gadd45gip1
	Pygm	LOC102642893//	Txndc11
	Rhoc	Sqstm1	Runx1t1
	Lpar1	Tceal3///Tceal5/	Aldh1a3
	Nampt	Ptplad2	Sema6b
	Dvl2	Pccb	Cyp2f2
	Fcgr3	Gadd45b	Elk3
	Hipk2	Cacng4	Eps8l3
	Spata5	Gpc3	Rtn4ip1
	Slc14a1	Aco2	Cyp11a1
	Fam114a1	Slc29a3	Usf1
	Enpep	Ergic3	Gne
	Lrp1	Ctsf	Wnt5a
	Speg	Lrp6	Angpt2
	Irak1	Arl1	E2f6
	Pvrl3	Ftsj3	Cdo1
	Rnf25	Baiap2	Serinc3
	Fam118a	Ptpn9	Rassf1
	Prkci	Nop9	Icam2
	G0s2	Bcap31	Ltbp1
	Cxcr4	Scyl1	Evc
	Hba-x	1700018B24Rik	Hp
	Tle6	Stxbp4	Dock7
	Cp	Sgta	Ncor2
	Snai1	Gdi1	Akr1b8
	Plek	Aars	Ctnna2
	Plek	Esyt1	Cpxm1
	Col15a1	Vash2	Sep-15

Fyn	Tut1	Ppap2b
Mog	Fbln1	C1qtnf1
Runx1t1	Polr1d	Csf1
Cd200	Vopp1	Mafg
Aldh1a3	Eif2b2	Cd302
Sema6b	Cyhr1	Twist2
Elk3	Celf2	F13a1
Wnt5a	Hsbp1	Gng11
Pdk2	Ccdc101	Nrp1
Vil1	Arhgdia	Slc50a1
Pfpl	Spcs3	Car4
Cdo1	Mavs	Il1r1
Rpp25	Nt5c3b	Cadps
Serinc3	Sf3b5	S100g
Rgn	Aen	Nfat5
Mug-ps1///Mug1	Zswim1	Gria1
Icam2	Crabp2	Tfap2c
Ltbp1	Scara5	Ghrl
Evc	Prrc1	Acadl
Dlx2	Immp1l	Myo1b
Hp	Scamp5	Myo1b
Dock7	Rerg	Ina
Cpxm1	Jkamp	Rom1
Fam3c	Lamb1	Slc16a3
Mrps34///Nme3	Ppp5c	Gla
Ppap2b	Zfp422	Fndc4
C1qtnf1	Fabp4	Retnla
Mafg	Tram1l1	Nes
Cd302	Dusp18	Rnf128
Twist2	Erc1	Crem
Kcnn2	Arpp21	Hsd11b1
F13a1	Zscan12	Naga
B4galt2	Arap3	Rab4a
Gng11	Fus	Cog1///LOC102641618
Nrp1	Aif1l	Gli1
Cxxc5	Dclk1	Slc2a2
Plscr2	Map1lc3a	Apcdd1
Tfap2c	Nabp2	St3gal6
Ghrl	Rrp9	Fbp2
Acadl	Cby1	Gjc1
Myo1b	Cmbl	Poli
Myo1b	Zfp521	Ebf2
Gla	Spon1	Gpx3
Fndc4	Tmem119	Socs2
Nes	Mtap	Rhob
Hexa	Mtap	Rgl1
Rhou	Ino80e	Zfp90
Rhou	Lpcat1	Cd1d1
Syn2	Cops6	Cd1d1
Klkb1	Cops6	Spic
4930544G11Rik	Ccdc107	Sox18
Crem	Zfyve19	Nudcd2
Hsd11b1	Aamdc	Chst1
Ctcf	Kdm8	Mmp12
Hacl1	Cuta	Col11a1
Tlr1	Pcsk5	Nr2c1
Pcbp4	Nop16	Akap2
Kel	Brca1	Piwil2
Gli1	Spsb4	Ccna1
Apcdd1	Egfl7	Midn
Myl7	Egfl7	Cd5l
Tex40	Slit2	St3gal5
Mfap5	Chodl	Slco1a5
Gjc1	Antxr1	Slc22a21
Ebf2	Pcdh20	Cox8b
Ebf2	Ifnar2	Rrbp1
Sh2d2a	Prr5	Ebi3
Gpx3	Mtg2	Trpm5
Socs2	Ears2	Hic1
Stk3	Cryzl1	Psg16
Pcm1	Plxnd1	Rnf138rt1
Zfp90	Zfp940	Cdh2
Selplg	Syn1	Clcn2
Cd1d1	Csnk1a1	Ndp
Cd1d1	Cadps2	Spp1
Opn1sw	Mef2c	Syt11
Sox18	Mef2c	Gfpt1
Chst1	Serpina1b	F5
Mmp12	Steap1	Gpx2
Col11a1	Mpi	Esm1
Akap2	Ggact	Ntng1
Gpr65	Gm16340///Ifi20	Casp12
Wfdc12	Nr2c2	Pde1a
St3gal5	Fam92a	Sesn3
Igf2bp1	Zbtb20	Zfpm2
Slc22a21	Ppp6r2	Tenm3
Trpm5	Tulp1	Rspo1
Mapk8ip2	Mmgt2	Gm13363///Ptp4a1
Sh3gl2	Cplx4	Fads2
Bhlha15	Snx6	1600015I10Rik
Psg16	Rtbdn	Ly75
Hrg	Acvrl1	Fhl5
Cdh2	Psg-ps1	Timp3
Ndp	Slc1a2	Tdo2
Smc1b	Airn	Mpp6
Spp1	Cebpg	Osr1
Pbx2	Rab4b	Pdgfc
Ufm1	Slc24a1	M1ap
Syt11	Ap4s1	Pax1
Mecp2	Slc12a5	Csf2rb2
Gfpt1	Drap1	Aurkc
F5	Arxes1///Arxes2	Dcn

Cadm3	Ednra	Tmprss2
1700029P11Rik	Fgf12	Sox4
Ccl19///Gm1240	Gpx4	Pipox
Gpx2	Cmtm7	Ces2a
Tescl	Scgb2b20///Scgb	Kdr
Ntng1	Oprm1	Pacsin1
Skp2	Mafb	Slc6a12
Pde1a	Mtmr6	Adssl1
Sesn3	Slu7	Tal1
Hsf2	Pik3r1	Gpatch4
Dbndd1	Znhit1	Zfp37
Tenm3	Atg12	Aoc3
Cyp4f15	Flt1	Chst7
Tubg2	Lamc3	Pde9a
Rspo1	Hmgcr	Jam2
Serpina1b///Serp	Pcdha1///Pcdha	Pde1b
Gm13363///Ptp4	Ddx60	Trpc6
Ly75	Ryk	Klf7
Pdzd3	Tfpi	Cst10
Fhl5	Tfpi	Ptges
Anapc16	Pja1	Ptges
2210409E12Rik	Klhl24	Foxi1
Osr1	Tom1l2	Rbp7
Pdgfc	Ccdc25	Klk1b8
Zrsr1	Gcc2	Kcnq1
Csf2rb2	Vegfb	Clec3b
Tbx21	Rwdd3	Gm20737///Gm20772///Gm20773///Gm20777
Mmp8	Zfp451	St6galnac5
Sox4	Diras1	Pkd2l2
Pipox	Sp100	Dlx1
Ces2a	Btn1a1	Kcnmb4
Pacsin1	Copz1	Cd40
Adssl1	Nox4	Slc2a10
4931417E11Rik	Sptbn1	Mmp7
Tal1	Osbpl6	Stc2
Gpatch4	Cklf	Mbd4
Zfp37	Ncor2	Il12b
Ankrd61	Notum	Dazl
Il1b	Arhgap6	Kpna1
C1qc	Lsm2	Il27ra
Chst7	Speg	Ssbp4
Pde9a	Kif13a	Gadd45a
Ift81	Gtf2ird1	Ttc28
Jam2	Fgfrl1	Unc5c
Sult1c2	Hyou1	Fmo3
Zfp53	Trim15	Gdpd3
Fzd4	Rftn2	Pcdhb7
Kcne2	Mrgpra4	Figf
Fkbp1b	Vrk2	Trps1
Trpc6	Srpx	Leprel2
Mmel1	Ppm1a	Msh5
Klf7	Igkv5-39	1700010I14Rik
Decr1	abParts///Igkv14	Zscan5b
Gp2	Prok2	Zfr
Foxi1	Mrap	Fetub
Rbp7	Glcci1	Krtap1-5
Reln	Uba5	1700001G17Rik
Clec3b	Cmtm1///Cmtm	Cntn1
Gm20737///Gm	Loxl1	Thpo
St6galnac5	Arrb2	Trhr
Pkd2l2	Mapre2	Tpm2
Kcnmb4	Mapre2	Pcdhb20
Slc2a10	Epha7	Il1rap
Mmp7	Taf11	Nkap
Gm10094///Sap	Gm8539///Zbtb	Mras
Stc2	Vamp7	Casp4
Tssk2	Igf1	LOC102638039
Il12b	Ldb1	[]
Gzmm	Col4a1	C79490
Kpna1	Zfp281	Acp1
Il27ra	Ppp1cc	Pagr1a
Grk5	Rnf11	[]
Gadd45a	Vstm2a	Fkbp10
Ttc28	Dcun1d1	C920021L13Rik///C920021L13Rik
Cd93	Zbed3	Tlr7
Unc5c	Copb2	Cct3
Pcdhb7	Arl16	LOC101055763
Trps1	Igfbp5	Nudcd1///Nudcd1
Leprel2	Zfp160	[]
Chrng	H6pd	C77545
Sycp3	Ets1	AA536748
Kcnn1	Grwd1	Mphosph9
Gcnt1	Srebf2	[]
1700010I14Rik	Meg3	Tufm
Kcnh2	Ipo8	Tufm
Fgf18	Prcp	Tmem38b
Tle3	Prcp	C80678
Nkapl	Swsap1	Tubb2a-ps2
Cntn1	Pde2a	Oxsm
Tpsg1	Prdm4	Arhgef25
Tktl1	Ccdc117	Arhgef25
Klrb1b///Klrb1c	Ccser2	Etf1
Trhr	[]	Nr5a2
Gstp1	Dnajc10	[]
Emid1	Col6a2	Fgf4
Pcdhb20	Nbea	AA522020
Nkap	Slc35f4	AA517023
Mras	Tenc1	Spsb1
LOC102638039	Cubn	[]
[]	Zfand3	D7Ertd183e
Gm3716	Farp1	[]
[]	Rassf8	[]

Hdlbp	Ptprz1	Tmem191c
Pagr1a	Msi1	Gadd45b
Sel1l2	Slit3	AA414993
Mark1	Rmdn1	[]
Fkbp10	2810002D19Rik	Prph
Prpf19	Spryd3	[]
Trav12n-1	Pvt1	[]
[]	Iqsec1	Frat1
Gpr137b	Mxra8	Ssbp2
[]	Zfp395	Abcb11
Tmem38b	Itsn1	Dmc1
Arhgef25	Adamts7	Dach2
Arhgef25	Zfp518b	Fgf2
Map3k7	Echs1	Ptgfr
C80171	B3gnt1	Prl3c1
Nr5a2	2810459M11Rik	Tctex1d1
Atp6v1e1	Rd3	Sntb2
[]	Rai2	Gna14
4930453N24Rik	Rabl6	Scube1
[]	Malat1	Golt1b
[]	Epha7	Higd1b
[]	Creb3l2	Il4
Fzd3	Dnm3os///Mir21	Sema3a
AA522020	Wipi1	Hoxc9
AA517023	Cars	Tbx18
Spsb1	Rgs6	Bmp8a
AA511261	Hoxa11os	Prkg1
D11Ertd326e	Fes	Casr
AA589418	Hoxc8	Tmem47
Ssx2ip	Actn1	Mlph
C76628	Lpar4	Grin3b
AA517650	Abcf1	Map3k6
AA517650	Tfpi	Selp
[]	Ccdc167	4933425L06Rik
Zfp30	Bean1	Prg3
Abcb4	Prkab1	Tsen15
Dmc1	Erbb3	8430419L09Rik
Prg4	Gm11767	Ilk
Fgf2	Dlx6	Sec61a2
Ptgfr	Fanca	Prss30
Tctex1d1	Xylt2	Lce1h
Sprr2f	P2rx4	Capn12
Spesp1	Ceacam1	Cd209f
Crisp3	Tcf3	Spock3
Scube1	[]	Il11
Nr0b2	Galm	Samt3
Higd1b	Zfp688	Gimap1
Il4	Ptk7	Vamp5
Sema3a	Gm9780///Plac9	Cacna2d1
Hoxc9	Adamts4	Ccng1
Tbx18	AI413582	Ubqln2
Bmp8a	Taf6l	Gtpbp1
Ly96	1190005I06Rik	Gtpbp1
Prkg1	Ltn1	B3gnt2
1700008F21Rik/,	Ssbp1	Sdc3
Casr	Cdk5rap2	Itga9
Tmem47	Lrrtm1	Tub
Epas1	Senp6	Arx
Dcbld2	Bag5	Tmem59
Mlph	Enpp4	Hs6st2
Map3k6	Trim62	Hira
Selp	Tmem158	Asph
2210418O10Rik	Zdhhc2	Maged1
Lag3	Zdhhc2	Fmn2
Cd209g	Ap1s2	Fmn2
Cd70	Ttc27	Adcy7
Zfhx3	Tmcc2	Celf2
Lce1h	Rab2b	Nrk
Nptx2	Myl9	Cxx1c
Capn12	Luc7l	Etv5
Spock3	Rps4l	Srprb
Gabrd	Ebf3	Acyp1
Il11	Lepre1	Gna12
Samt3	Creld2	Hoga1
2310034C09Rik	Hba-a1///Hba-a2	Adam10
Prss29	Rrbp1	Renbp
Vamp5	Txn2	Gas2
Cacna2d1	Prmt1	Scn1a
Ctnnb1	Pigk	Scn1a
Cx3cr1	Lphn1	Ryr2
Cx3cr1	Cnep1r1	Abcg3
Ubqln2	Cad	Pla2r1
B3gnt2	Lpin2	Mcoln3
Itga9	Il6st	Hmmr
Slco2a1	Med12l	Ikbkg
Usp9x	Prkce	Ids
Timp2	Gtl3	Ptprt
Hs6st2	Pgp	Ern1
Atrx	Tpi1	Brdt
Kif2a	Pitpnc1	Sh2b3
Vsnl1	Cntn1	Tef
Enc1	Sec31a	Gnas
Maged1	Msl2	Zfp106
Fmn2	Rgs8	Sox13
Adcy7	Snapc3	Hcn1
Ubr1	Obox1///Obox2	Gys1
Celf2	Slc25a39	Proz
Kif3b	Wdsub1	Grin1
Kif3b	Gkn2	Smyd1
4933411K20Rik	Ptgr2	Dlc1
Nrk	Plscr1	Elmo1
Cxx1c	Smpd4	Hoxd3///Hoxd4

Etv5	Rai1	Bmp15
Angptl2	2700089E24Rik	Pcdhb16
Nolc1	Dhx30	Spdef
Hoga1	Rpusd1	4933417A18Rik
Tcerg1	Ppp2r2a	Klk1b4
Magi3	Pgm1	Apaf1
Renbp	Anapc5	Prlr
Abcc2	Khdrbs3	Xiap
Gas2	Tex9	Dnase2b
Gnaq	Abhd12	Fut2
Tcf7l1	Ttc39b	Otud7a
Tnnt3	Chst4	Lnx1
Scn1a	Rab28	Stat5a
Scn1a	Timm10b	Pcdhb5
Ryr2	Dynlt1a///Dynlt1	Chka
Atp2a3	Far1	Scn10a
Gata5	Wdr33	Tlr8
Loxl4	Tmem216	Pcdhb11
Fzd3	Pter	Il1rl2
Pla2r1	Hbs1l	Rrh
Ankef1	Thoc7	Atp7b
Gopc	Rnf41	Pde3a
Itga4	Derl3	Npr3
Rem1	Nphp1	Hormad1
Ikbkg	Serpinf1	Il6
Dpf3	Pitpnc1	Vax2
Ascl1	Fam110a	Klrc2
Slc4a4	Ndst3	Astn2
Gzme	BC051212	Grid2ip
Ngfr	Ergic2	Cdh20
Cux2	Nsa2	Dqx1
Clcnka	Pi16	Pcdhb13
Gnas	Zbed3	LOC100043315
Gys1	Hnrnpab	Arr3
Csf2rb///Csf2rb2	Gadd45g	Gata2
Proz	Zbtb46	Il21
Grin1	Slc25a26	Setd1a
Smyd1	Ptrf	Clcnkb
Hoxd3///Hoxd4	Ptgfr	Ptger3
Pde7b	Ssb	Syt10
Rcvrn	Sdccag8	Jdp2
Ccl28	Cdk14	Mtnr1a
Ccl28	Med1	Ptdss2
Spdef	Lig3	Arl4d
4933417A18Rik	Stxbp4	Prop1
Klk1b4	Ubxn8	Figla
Insr	Exosc10	Kcnip4
Prlr	Chpf	Tshb
Rsph6a	Rgs3	Tapbp
Otud7a	Rnf138	Msn
Lnx1	Ddhd1	Bcl6
Onecut1	Slx1b	Bace1
Arhgap31	Lmo2	Abca1
Cer1	Pdss1	Slc22a5
Pcdhb5	Cxxc1	St3gal3
Chka	Kif2a	Serhl
Scn10a	Jmjd6	Kdelc1
Vmn2r-ps54	Mterfd1	Chrna6
Tacr3	Pcgf6	Ncam1
Tbata	4921524J17Rik	Nr1h3
Atp7b	Ccnc	Socs1
Pde3a	Pla2r1	Stc1
Uba1y	Clpb	Rilpl1
Mageb3	Eri2	Rcan3
Chrna7	Cyba	Spock2
Gabrr1	[]	Pde6g
4930470P17Rik	Tspan12	Il21r
Vax2	Ttr	Cbl
4930591A17Rik	Dpysl3	Ncoa2
H2afy3	C86187	Sppl2a
Dqx1	Iffo1	Crhr2
4931432E15Rik	Gtf2a1	Smad3
Setd1a	Tmem229b	Cnr2
Nek8	Etnk1	Grk6
Pcdhb8	Rpl41	Vsx1
Ptger3	Chchd7	Sall1
Jdp2	Zranb2	Cngb3
Mtnr1a	Spata13	Musk
Trhr2	Timp2	Ntn4
Figla	Hdc	Kcnj11
Pspn	Dusp9	Tal2
Epdr1	Aamp	Hnf4g
Abca1	Tsc22d1	Cldn9
Golph3	Rbbp4	Tlx1
Slc22a5	[]	Plagl1
Map1b	Serinc1	Krtap12-1
Anp32a	Pkdcc	Mid2
Pde6a	Rbbp4	Ammecr1
Yipf4	Amot	Elk4
Chrna6	Cds2	Mgst3
Chrna6	Lpp	Ifna1
Capn6	Rarb	Col2a1
Mrc1	Pbx4	Rbm14
Rbpjl	Gm561	Bdkrb1
Ncam1	Psma5	Olfr749
Gfra1	Ublcp1	Vmn1r53
Nr1h3	Ntn1	Olfr1508
Hnf4a	Ambra1	Hist1h1t
Stc1	H2-Ke6	Ifnab
Rilpl1	Nhsl2	Ifna5
Rcan3	Srr	Pdia4
Pde6g	Slc25a4	Col5a2

Akr1c12///Akr1c	Angptl2	Slc2a8
Cbl	Vtn	Qtrt1
Ncoa2	Ckb	Rhoa
Serpinb9c	Atp1a2	Alppl2
Pitx2	Nlgn2	Aebp1
Sox3	Syt11	Vim
Sall1	Ppp2r3d	Cyp51
Kcna7	Maneal	Gng10
Kiss1r	Pitpnc1	Ctsk
Ceacam1	Mrps11	Spz1
Mthfr	Rgs16	Pten
Chst11	Coro1a	Adamts5
Cacna1a	Gm13889	Rgs7
Zscan2	Leprel4	Thbs2
Rab17	Cp	Dbh
Tal2	Sep-04	Tagln3
Tlx1	Lgals1	Etv1///Gm5454
Plagl1	D430019H16Rik	Dusp2
Cma2///Mcpt9	Adcy2	Hfe
Krtap5-1	Ap2a2	Slc7a2
Magea1///Mage	Syne1	Kcnj9
Usp17lc///Usp17	Col1a1	Adamts1
Htr1f	Mfsd4	Sh2b2
Klra15	Mrs2	Fam126a
Hoxd12	Ddx49	Tnfrsf21
Tex13	Cnn3	Sec16b
Mgst3	Eif4ebp2	Polr3k
Ifna1	C1qtnf1	Zpbp
Col2a1	Hoxa9	Cyct
Bfsp1	Tspan17	Cdh11
Cyp11b2	Mterfd3	Rims2
Chrm4	Taok3	Zfp191
Galr3///Gcat	Ndn	3632451O06Rik
Fpr3	Olfm1	Fut9
Bdkrb1	Arhgef40	Kcnd2
Olfr67	Vasn	Agpat6
Olfr749	Scarb1	Reck
Ifna15///Ifna6///	Lnx1	Trip4
Magea4	Gm6277	4930550L24Rik
Vmn1r10///Vmn	Pomc	Adcyap1r1
Vmn1r53	Lrrtm1	Adam21
Vmn1r14	Rspo2	Tlx3
Olfr140	Kcnk1	Fpr1
Pnmt	Tgm2	Bcl2a1a///Bcl2a1b///Bcl2a1c///Bcl2a1d
Orm3	Chpt1	Lyz1
Ifnab	2610507B11Rik	Ptch1
Col5a2	Adrb3	Saa3
Aebp1	Gpaa1	Serpinh1
Vim	Adamts4	F2r
Gng10	Zmiz1	Col1a2
Myo10	Gjc2	Fancc
Ctsk	Vgll4	Rad54l
Spz1	Ddx18	Dclk1
Pten	Xpa	Cfh
Adamts5	Marcks	Cd36
Dbh	Fmod	Cd36
Col9a2	Elmo2	Tmem167
Tagln3	Rnf112	Cap2
Etv1///Gm5454	Zic5	B4galt6
Igf2bp3	AK213652	Src
Rgs17	Calr	Tgfb2
Ahr	Mela	Tgfb2
Dusp2	Rbm5	Dnase1l2
Selenbp1	Slc52a2	Entpd1
Hfe	Neto1	Prkca
Scg2	Vim	Nrl
Cyp1a2	Obox2	Pln
Sh2b2	Adcy7	Tm4sf1
Fam126a	Ptpla	Tceal3///Tceal5///Tceal6
Sec16b	Pofut1	Gadd45b
Nr4a2	Sf3b2	Cacng4
Zpbp	Shroom1	Gpc3
Cdh11	Limd2	Dnajb7
Cntnap2	Cnn3	Meis1
Bmp6	Ubxn10	Xdh
3632451O06Rik	Ppargc1a	Rnf151
Hmga2	Rcbtb1	Ifrd2
Reck	C1ra///C1rb	Hcn3
Trip4	Rab3il1	Akr1c21
Rhpn1	Mela	Trpc4
Tg	Zfp574	Scyl1
Avp	Wdr33	Zfp474
Cbx1	Trim30a///Trim3	Stxbp4
Adam21	Osbpl6	Ggt7
Bcl2a1a///Bcl2a1	Dapk3	Esyt1
Capn15	Eras	Vash2
Lyz1	Tcfl5	Nup133
Hoxc5	Nnt	Aacs
Serpinh1	Ndn	Fbln1
F2r	AU018829///Gm	Vopp1
Arvcf	Chst11///Phactr	Calb2
Col1a2	Eif4ebp2	Celf2
Dclk1	Bsg	Aen
Mrps24	Wipi1	Crabp2
Lipa	Sarnp	Scara5
Cfh	Ubqln2	Scamp5
Gpr137b	4930414L22Rik	Rerg
Cd36	Eya4	Jkamp
Cox16	Serpinh1	Lamb1
Tmem167	Armcx2	Rnpep
Cap2	Gpm6a	Pxk
B4galt6	Ergic3	Fabp4

Tgfb2	Zfp14	Frmd6
Tgfb2	Rasal3	Tram1l1
Hdgfrp3	C80360	Pdzd11
Pnn	C76472	Ntmt1
Gdap1	Iqgap2	Aif1l
Cspg4	Sema7a	Dclk1
Nrl	Cdr2l	Rrp9
Smc3	Ggnbp2	Ctsl
Yme1l1	Bub3	Gm11787///Lyn
Sqstm1	Mir425	Abhd14b
Tm4sf1	AA517562	Tmed1
Tceal3///Tceal5/	Cacna1a	Inpp5b
Cacng4	Thyn1	Zfp521
Adprm	Aldh7a1	Acrbp
Tdgf1	Slbp	Nisch
Gpc3	Lasp1	Spon1
Meis1	Sfrp1	Tmem119
1700019N19Rik	Itpr1	Mtap
Cnnm2	Grasp	Ino80e
Rnf151	Fbn1	Deptor
Nol11	Usp39	Pnpla7
Ror1	Pkd1	Polr3gl
Lrp6	Pcp4	Alg1
Hcn3	Polr1a	Ccdc107
Arl1	Snx2	Mrap
Bcl2l2	Trap1a	Chuk
Rock2	Stag3	Dpp6
Syt13	Guca2b	Pcsk5
Orm1	St3gal5	Nop16
Zfp474	Sostdc1	1810011O10Rik
Pex5l	Zfp105	Brca1
Stxbp4	Cmtm7	Spsb4
Txndc5	Gtf2i	Dhx58
Vash2	Grap2	Egfl7
Tbc1d22a	Itga9	Slit2
Nup133	Timp2	Cux1
Fbln1	Lpin2	Chodl
Pink1	Smarca1	Antxr1
Cyhr1	Il6st	Gca
Celf2	Nphs2	Thnsl2
Vldlr	Nr3c1	Ahctf1
Hsbp1	Akt3	Ifnar2
Lrrc49	Ict1	Prr5
Nrros	Tal2	Ears2
Spcs3	Pik3ca	Plxnd1
Crabp2	B4galt6	Tmem53
Tmx1	Tm9sf2	Ccsap
Scara5	Sh3kbp1	Csnk1a1
Prrc1	Mad2l2	Cadps2
Scamp5	Asrgl1	Ushbp1
Cend1	Rtfdc1	Mef2c
Rerg	Dnm1	Mef2c
Jkamp	Hspa12b	Serpina1b
Lamb1	Gnao1	Glyat
Zfp422	Slc1a1	Steap1
Pxk	Eny2	Chil1
Fabp4	Nudcd3	Mpi
Tram1l1	Ccdc117	Ssbp2
Dusp18	Fbln7	Krt84
Fus	Zdhhc1	Art5
Aif1l	Obsl1	Uroc1
1810043G02Rik	Lincpint	Gm16340///Ifi203///LOC100862473///LOC1026
Dclk1	2700089E24Rik	Zbtb20
Tmod2	Ednra	Mmgt2
Lgalsl	Nabp1	Cplx4
Vcam1	Cdc23	Ces3a///Ces3b
Gm11787///Lyn	Trim46	A530016L24Rik
Cmbl	Sep-11	Klk1b21
Abhd14b	Trim16	Amelx
Zfp521	Prpf19	Psg-ps1
Acrbp	Yars	[]
Nisch	Ap1g1	Slc1a2
Spon1	Ilf3	Ankrd33
Tmem119	Klhdc2	Armc1
Acer2	Ehmt2	Cebpg
Lpcat1	Dnajc21	Rab4b
Polr3gl	Tspyl1	Fam161a
Ccdc107	Mtch1	Slc12a5
Ugt3a1	Soat2	Alas2
Utp6	Mc5r	Srxn1
Chuk	Ap2a1	BC089597
Gemin8	Ercc2	Amelx
Pcsk5	Rock1	Ednra
Slit2	Slc35c2	Fgf12
Clec2h	A630038E17Rik	Evc
Chodl		Cmtm7
Ccdc104		Slc41a3
Gca		Mafb
Pcdh20		Mafb
Ifnar2		Mtmr6
Mfap3		Pik3r1
Mtg2		Ogt
Cntln		Atg12
Plxnd1		Irak4
Tmem53		Apobec1
Zfp940		Flt1
Syn1		Lamc3
Luc7l3		Hmgcr
Cadps2		Ncf1
Ushbp1		Ddx60
Mef2c		Crtc3
Mef2c		Nfatc2ip

Usp43	Tfpi
Glyat	Tfpi
Fbxw2	Klhl24
Sgtb	Tom1l2
Steap1	Adam22
Ssbp2	Diras1
Fbxo21	Scrn2
Upp2	Nox4
Art5	Acsl4
Uroc1	Nf2
Zfp810	Ap3b2
Gm16340///Ifi203///LOC1008624	Pde7a
Fam92a	Ncor2
Bcl9	Lmnb2
Zbtb20	Prlr
Ttr	Csn3
Ppp6r2	Fktn
Cplx4	Notum
Pax4	Trim30a
Dyx1c1	Arhgap6
Tspan33	Kcnj6
Cxcl17	Brd4
Hrnr	Ghr
Amelx	Eda
Ces4a	[]
Psg-ps1	Dysf
Golph3l	Magi1
Lmbrd1	Scn8a
[]	Adam33
Lbh	Mx1
Ighg	Ubxn10
Gngt1	Sec14l1
Airn	Prpf4b
Slc22a29///Slc22a30	Cd6
Ankrd33	Ace
Prol1	Fgfrl1
Armc1	Cct3
Rsrc2	Nfat5
Kif1b	Rftn2
Fam161a	Edn1
Zfp825	Rad18
Clmn	H2-D1
Trim3	Srpx
Slc12a5	Fcgr2b
Alas2	Tnfsf11
Arxes1///Arxes2	Smr2
Cant1	Il1f10
Ednra	Vegfa
Fgf12	Parp3
Cmtm7	Uba5
Gabra6	Loxl1
Slc41a3	Mapre2
Oscar	Mapre2
Wnt9b	Epha7
Prpf4b	Ldb3
Dbn1	Nfe2
Pik3r1	Rbm14
Ddit4l	Calca
Flt1	Uxs1
[]	Atp10a
Lamc3	Igf1
Hmgcr	Cyyr1
Ncf1	Col4a1
Kifap3	Vstm2a
Tfpi	Epsti1
Tfpi	Zbed3
Klhl24	Chst15
Tom1l2	Arl16
Ccdc25	Igf1r
Vegfb	Rab23
Phip	Plk4
Suv420h1	Rrp1b
Nox4	Fbxo22
Nf2	Pth2r
Prlr	Tmem175
Fktn	Slc6a1
Notum	H6pd
Arhgap6	Ets1
Jmy	Chpf2
Speg	Meg3
Dysf	Prcp
Kl	Pde2a
Magi1	Lmna
Scn8a	Ccdc117
Sema6c	Dnajc10
Kynu	Mndal
Prpf4b	Galnt7
Ace	Tmem191c
Fgfrl1	Medag
Cct3	Prickle1
Trim15	Col6a2
Nfat5	Nbea
Lman1l	Cidec
Rftn2	Tenc1
Rad18	Cubn
Glra4	Arsg
Sntb1	Cfp
Srpx	Rassf8
Fcgr2b	Ptprz1
[]	Msi1
Vegfa	Pmepa1
Mia2	Rmdn1

Parp3	Kif11
Loxl1	Mxra8
Casd1	4930427A07Rik
Irx1	Adamts7
Mapre2	Zfp518b
Mapre2	Ldlrad4
Epha7	Lmod2
Ldb3	Ifi204///Ifi205///Mnda///Mndal
Nfe2	Brd8
Dlat	2810459M11Rik
Chrna3	Rai2
Atp10a	Trim16
Igf1	Csgalnact1
Cyyr1	Zfp444
Baalc	Malat1
Col4a1	Epha7
Zfp281	Dnm3os///Mir214
Vstm2a	Enpp3
Pja1	Wipi1
Chst15	Akna
Vimp	Plce1
Arl16	Erdr1
Igf1r	Spag4
Mtrr	Gpr31b
Fyb	Fes
Fbxo22	Il6ra
Slc6a1	Pclo
H6pd	Tfpi
Lrpap1	Gm21451///Loxl2
Chpf2	Tnfsf12///Tnfsf12Tnfsf13
Srebf2	Slc41a2
Meg3	Myof
Pde2a	Myh1
Ccdc117	[]
[]	Gm11767
Mndal	Atp11c
Tmem191c	Smok3a///Smok3b///Smok3c
Medag	Cd247
Nova1	Itgb1
Col6a2	[]
Nbea	Kdm5d
Cidec	LOC102632747
Tenc1	Ercc6l2
Cubn	[]
Arsg	Rprd1b
Cfp	Zfp688
Farp1	Ptk7
Rassf8	Gm9780///Plac9a///Plac9b
Msi1	Adamts4
Snx19	AI413582
Slit3	Agbl3
Atp1a2	Lrrtm1
Dmgdh	Rufy2
2810002D19Rik	Aak1
Hoxb9	Enpp4
Zfp82	Mbd3l2
Iqsec1	Myl1
Mxra8	Ap1s2
Mfsd8	Klhl7
4930427A07Rik	Svop
Itsn1	Ttc27
Adamts7	Rab2b
Zfp518b	Myl9
Apbb2	Snrnp40
Ldlrad4	Klf9
Ifi205///Mnda	Rps4l
Rai2	Gm10340///Gm10409///Gm16525///Gm2897/
Magi1	Trak1
Malat1	Ebf3
Epha7	Lepre1
Dnm3os///Mir214	Hba-a1///Hba-a2
Enpp3	Rrbp1
Wipi1	Pigk
Akna	Lphn1
Hoxa11os	Lpin2
Wtap	Il6st
Gpr31b	Ppfia4
Fes	Pcp4l1
Hoxc8	Pgp
Peak1	2810408M09Rik
Il6ra	Pitpnc1
Tfpi	Pitpnc1
Gm21451///Loxl2	Rftn2
Ccdc167	Ube2c
Tnfsf12///Tnfsf12Tnfsf13	Krtap3-2///Krtap3-3
Napb	Tril
Ercc6l2	Cntn1
Ankrd26	Prpf31
Prr15	Rgs8
Alpk2	Batf3
Copg2os2	Snapc3
Ap2a2	Col22a1
Gm11767	Obox1///Obox2
Cyp2c38	Polr3e
Lamc2	Plscr1
Ptms	Smpd4
Usp9y	Oasl2
Pax7	Rai1
Sox5	Dhx30
Nf1	Pgm1
Pax6	Ankrd33b
P2rx4	Anapc5

Ceacam1	Khdrbs3
Igk-V1///Igkv9-120	Fndc1
Defb11	Angptl4
Gm1549	Gnas
Ofa	Csnk2a1
Slco1b2	Slamf7
[]	Far1
Iscu	LOC102643054
Rprd1b	Fem1a
Ptk7	Stx8
Gm9780///Plac9a///Plac9b	Serpinf1
Tceb1	Ccnl2
Adamts4	Pitpnc1
AI413582	Dtl
1700001C19Rik	Fam110a
Agbl3	Ndst3
Lrrtm1	BC051212
Senp6	Hspa9
Rufy2	Pi16
Dnm1l	Pabpc1
Enpp4	Zbed3
3110007F17Rik	Gadd45g
Ndufa13///Yjefn3	Zbtb46
Klra1///Klra22	Rdh14
Ap1s2	Xk
Klhl7	1700120B22Rik
Tmcc2	Ptrf
Rab2b	Ptgfr
Rab2b	Gm9706
Myl9	Mrps5
Pnpt1	Cdk14
Prpf39	Pfn3
Luc7l	Med1
Rps4l	Fam122a
Lepre1	Ercc6l2
Creld2	Lig3
Hba-a1///Hba-a2	Stxbp4
Rrbp1	Cenpo
Gm6793///Hnrnpa3	Fmo2
Pigk	Ubxn8
Lphn1	Zfp142
Lpin2	Prmt3
Il6st	Ephb2
Pou6f1	Chpf
Ppfia4	Ppp6r3
Med12l	Flt1
Znhit3	Ddhd1
Scaf11	Lmo2
Rian	Pdss1
Galc	Cxxc1
Pitpnc1	Mknk1
Pitpnc1	Pcgf6
Rftn2	Stk31
Cntn1	Ccnc
Fam166a	Pla2g2d
Tspo2	Clpb
Rgs8	Ercc8
Batf3	Ikbkb
Col22a1	Dnajc2
3110079O15Rik	Rpgrip1
Obox1///Obox2	Cyba
Surf2	Nfatc4
Ptdss2	Kcnip1
Plscr1	Dpysl3
2700089E24Rik	Cpa2
Dhx30	Iffo1
Wbscr25	Iffo1
Eqtn	Tmem229b
Ankrd33b	Etnk1
Anapc5	Rpl41
Khdrbs3	Mbp
Fndc1	Zranb2
Tex9	Fez1
Sh2b2	Snhg1
Csnk2a1	Rhbdd3
Timm10b	Pten
Rps6	Map1s
Abhd15	Dusp9
Ppp2r3c	Aamp
Lta4h	Ficd
Far1	[]
Hba-a1	Serinc1
Hbs1l	Apcdd1
Rad23a	Rpl35
Rnf41	Pkdcc
Obp1a	Clu
Derl3	Mettl7a1
Cep55	Phldb2
Serpinf1	Slc9a8
Angel2	Slc52a2
Pitpnc1	Upk3b
Ccdc34///LOC102632005	Zbtb46
Ndst3	Amot
BC051212	Cds2
Orc4	6330509M05Rik///Slc9a7
BC065403	Ngfr
1110035H17Rik	Tbcel
Eed	Psma5
Pi16	Atcay
Zbed3	Slc5a2
Gadd45g	Ckap4
Zbtb46	Nhsl2

1700120B22Rik	Slc25a4
Tiam1	Ckb
Ptrf	Nlgn2
Eif2ak1	Syt11
Gm9817	Myzap
Ptgfr	Rps24
Sdccag8	Pitpnc1
Xrcc2	Mrps11
Igf2bp3	Ldhb
Pfn3	Rprd2
Fam122a	Rgs16
Lig3	BC094435
Ide	Fcgr2b
Cenpo	A4galt
Cts3	Bai1
Fmo2	Cp
Zfp142	Fam131b
Prmt3	Sep-04
Chpf	Lgals1
Flt1	Acadsb
Pibf1	D430019H16Rik
Thumpd3	Cyp2c54
Ddhd1	Adcy2
Lmo2	Gpr133
Cxxc1	Lasp1
Ccnc	Pigr
Grk4	Syne1
Ercc8	Col1a1
Kif2c	Cnn3
Eri2	Csn1s2a
Gpa33	Gm20559
Cyba	C1qtnf1
Cbx3///Gm5792///Gm6901///Gm	Cklf
Adam1b	Tspan17
Nfatc4	Tardbp
Cd80	Tial1
Tspan12	Gm1976
Ttr	Taok3
Dpysl3	Vprbp
Cpa2	Abcc8
Iffo1	Gtpbp8
Iffo1	Ndn
Etnk1	4922502D21Rik
Fez1	Eif3l
Timp2	Vasn
Sec62	Scarb1
Pten	Lnx1
Map1s	Pomc
C230052I12Rik	Txnl1
Ficd	Nanos1
[]	Lrrtm1
Serinc1	Rspo2
Apcdd1	Mvb12a
Pkdcc	Slc2a3
Slc9a8	Socs3
Slc52a2	Tgm2
Rbbp4	Chpt1
Zbtb46	Adrb3
Amot	Chrna3
Cds2	Rbpms
Fam189b	Atp1b1
Gm561	Ceacam11
Psma5	Adamts4
Rprd1a	Klk1
Ntn1	Matn2
Gabbr1	Sec61a1
Nhsl2	Gjc2
Mia3	Ddx18
Slc25a4	Xpa
Akr1d1	Arf3
Ckb	Preb
Nlgn2	Polr1a
Akap9	Elmo2
Syt11	BC089491
Ahi1	Rnf112
Pitpnc1	Kifc5b
Cml2	[]
Mrps11	Calr
Rprd2	Mela
1110004E09Rik	Svs2
Wdr33	Acsm2
Pigx	Gm13889
Fcgr2b	Socs3
A4galt	Mbp
Masp1	Ap1s2
Bai1	Gm5481///Gm6109///Gm6570///Gm7429///Rp
Sep-04	Vim
Lgals1	Adcy7
D430019H16Rik	Lasp1
Adcy2	Acbd3
Gpr133	Gas1
Fam21	Cep68
3110057O12Rik	Arhgap6
Elavl3	Klf9
Hnrnph3	Shroom1
Syne1	Limd2
Col1a1	Dner
Ighv14-2	Cnn3
Mfsd4	Zic4
Mrs2	Zfp64
Cps1	C1ra///C1rb
Serinc3	Mical1

Magnúsdóttir et al	Doody et al	Shaffer et al	Mould et al
Tcea1	CXorf42	FCRL5	U6
Tcea1	NR3C1	BCL11A	Tcea1
Tcea1	TAPBP	HLA-DMB	Vcpip1
Tcea1	NFIX	IL10	Sgk3
Rgs20	C14orf43	HLA-DPA1	Slco5a1
Rgs20	RBCK1	CD53	SNORA17
Rgs20	ADAMTS6	PRKCB1	Il17a
Rgs20	BTN3A3	CD68	Mcm3
Rrs1	FOXP1	PMAIP1	U6
Adhfe1	GLI3	LMO2	Ptpn18
Rrs1	INPPL1	CTSB	4632411B12Rik
Adhfe1	KLHDC9	CD27	Zap70
Vcpip1	LOC644974	CXCL10	Vwa3b
AK006607	MRPS23	SYK	Inpp4a
Vcpip1	PTPN18	CCL5	Wdr75
AK006607	RPL10A	AKT2	Stat1
Sgk3	SP100	IL16	Stat1
Sgk3	SP110	PTPN6	Stat1
Sgk3	ZSCAN2	TNFAIP3	Inpp1
Cops5	TLN1	FCER1G	Rftn2
Cops5	C22orf32	GCET2	Plcl1
Cspp1	APOL3	CR2	Nbeal1
Cspp1	C14orf181	ACTB	Rpl17-ps1
Cspp1	CCDC28B	HLA-DMA	Nrp2
Cspp1	CWF19L1	MYBL1	Nrp2
A830018L16Rik	IFIT2	TXNIP	AC167197.1
A830018L16Rik	SPC25	MYO1E	Gm216
Sulf1	ZHX1	TLR10	Vil1
Slco5a1	ORC4L	HLA-DOA	Ttll4
Slco5a1	TRIM34	AFF3	Stk11ip
Slco5a1	LRRC41	AIM2	SNORA17
Slco5a1	TNRC6B	CD24	Irs1
Slco5a1	SLC25A22	CD37	Pid1
Slco5a1	PSMB8	GSTP1	Sp110
AK015384	STAT6	VNN2	Rpl30-ps6
Rpl7	LMO2	IRF8	Alppl2
Rdh10	PSMB10	hCG_1998957///HLA-	Eif4e2
Tceb1	PVRIG	SREBF2	Gigyf2
Tceb1	RBM25	MGLL	D130058E05Rik
Tmem70	RNASEL	KPNB1	Scly
Tceb1	TRIM44	AICDA	Stk25
Tceb1	BCAT1	ISG20	Gm3608
Tmem70	PXMP4	EIF4EBP1	Gm10193
Tceb1	C2CD2	PAG1///PRDX1	U6
Tceb1	KIF18A	CSK	Zcchc2
Tmem70	NDUFA5	SLC2A5	Slc35f5
Jph1	ELL3	FCRL4	Gpr39
Tcfap2d	NUB1	BCL6	Ccnt2
Tcfap2d	RPS6	LYN	Rassf5
Efhc1	TNFRSF10B	SPIB	Elk4
4921533L14Rik	FAM123B	IFI16	Sox13
4921533L14Rik	ACSS2	SP140	SNORA32
4921533L14Rik	EHD1	BCL2A1	Zc3h11a
Col19a1	FCRL4	CD69	Gm3834
Col19a1	IL28A	BLNK	Cacna1s
Bai3	NEGR1	ARPC2	5730559C18Rik
Bai3	OR6K2	PCSK4///SUB1///IFRD	AC125186.1
B230209C24Rik	C11orf67	RGS18///RGS13	SNORA17
Bpag1	RALY	DNMT1	5S_rRNA
Bpag1	PIK3CA	MYO1B	Cfhr1
Bpag1	TMEM179B	CDC123	Trove2
Bpag1	AMY1A	RB1	Fam5c
Dst	ATF3	CAMK1	U6
Bpag1	PSMA4	PEA15	Rgl1
Bpag1	YWHAH	CIITA	Smg7
Bpag1	KRT85	CHKA	Smg7
Dst	PEX12	PSMB9	Glul
Ptpn18	ASB8	TUBB	Gm9530
Ptpn18	DUSP10	FTL	Tor1aip1
AK082264	MAN1C1	PCDHGC3	Fam20b
Arid5a	DYNC1I2	PARP1	Gas5
Arid5a	HDGF2	POU2F2	Prdx6
4632411B12Rik	BTN3A2	GOT2	AI848100
4632411B12Rik	ITGB7	NAPSB	Prrc2c
1700024G10Rik	UBR2	OAS1	Brp44
1700024G10Rik	RNF219	PA2G4	Gpa33
Tsga10	TPD52L1	YWHAQ	Uck2
Tsga10	FAM129C	FTH1	Uap1
Tsga10	HIRIP3	WIPF1	Uap1
Tsga10	HMBOX1	FCRLA	Uap1
Tsga10	CIITA	IGLC2	U1
AK039030	TMEM79	PTDSS1	1700009P17Rik
Lipt1	EPHA4	RHOH	B4galt3
Npas2	RLIM	MTHFD2	Ncstn
Map4k4	AIM2	ITGB7	Dcaf8
Map4k4	CLEC4A	ATP2A3	Apcs
Il1r1	C10orf4	PTPRC	U2
Il1r1	KIAA1680	PCDHB16///ME1	Sdccag8
BC058417	CHM	CCL4	Gm17407
BC059892	ZFP36L1	FLII///FLI1	4930527J03Rik
AK019154	CUEDC1	SLC25A37///MSC	Efcab2
AK048630	ALG10	TMSB4X	Gm15480
Pou3f3	WDR5	IGJ	Sccpdh
AK053590	MYL6	ID3	Adck3
AK019154	RFX5	CCL3	Acbd3
AK048630	ANGEL2	BNIP3L	Cnih4
AK011913	HEATR1	DAXX	5S_rRNA
AK019154	HNRNPK	ZFP36L2	Srp9
AK048630	KCNH8	BCL7A	Degs1
AK011913	MTHFS	TACC3	Tlr5
AK019154	NME7	DUSP12///DUSP1	Dusp10
AK048630	TSLP	C6orf108	Dusp10
AK011913	MCFD2	ID2	Mosc2
Tpp2	WIBG	MCM7	Mosc2
Tpp2	CCNB1IP1	BIRC4	Mir194-1
1700029F09Rik	SART3	GADD45B	Gpatch2
1700029F09Rik	TBC1D22B	ACTL6A	AC124613.1
Kdelc1	TMEM69	XRCC5	Atf3
Kdelc1	SMPD3	EBF1	Traf5
Bivm	OSBPL3	ST3GAL1	U5
Bivm	BCAP31	HLA-DRA	7SK
Bivm	CYB5B	PLEK	A130010J15Rik
Col5a2	HIST2H2BF	GMPS	7SK
Col5a2	PPFIBP2	ACTG2	AC109145.1
AK133153	TMEM39A	TLR1	Frmd4a
TMEFF2	FBXO16	KLF10	Optn
Tmeff2	HAS3	MARK2	Upf2
Stat1	INTS8	XRCC6	Gm13218
Stat1	TAGLN3	STAT6	Prkcq
Stat1	DCC	CDK2	Il2ra
Stat1	BRIP1	CLPP	Fam23a
Stat1	TMF1	IL8	Mrc1
Stat1	FAM82A2	PAK3///CDKN1A	Cacnb2
Stat1	GJA8	ISG15	Gm13313
Stat1	RFX1	ICAM1	Gm13334
Stat1	SH3BP5L	TRIP10	Gm20539
Stat1	EML2	ST6GAL1	Etl4
Hspd1	MGAT2	ANP32A	Etl4
Hspe1	SMS	FYN	Arhgap21
Plcl1	HIGD1A	NOL5A	Arhgap21
1110034B05Rik	SSR2	CDC37	Gm13380
1110034B05Rik	MORN2	VEGFA	Yme1l1
9430016H08Rik	PIGR	HSPA1B///HSPA1A	AL732528.1
Ppil3	AGK	MAP2K1	E130003G02Rik
Ppil3	MEIS1	GSK3A	Fbxw5
Ppil3	ADCK4	DDIT3	Lcn14
Ppil3	KRT10	RAP1B	Gm13553
Nif3l1	C10orf26	PBEF1	Qsox2
Nif3l1	CCDC19	SIAH2	Vav2
Nif3l1	CCL4	IFITM1	AA645442
Trak2	CCL4L1#CCL4L2	LRMP	Vav2
PAPK-A	CCL4L1#CCL4L2	GPR18	Gfi1b
Als2cr2	SDR39U1	HLA-DQA1	Gm13394
Als2cr2	ELMOD3	PLK1	1700101E01Rik
PAPK-A	NFE2L2	IFITM2	Gm13402
PAPK-A	TECPR1	MMP1	Gm14471
Fzd7	OCIAD2	MMP2	Ier5l
Fzd7	ANKRD17	DUT	Gm14486

Sumo1	SPATA4	S100A4	Fubp3
Nol5	HMGN4	SLC25A21///ODC1	Lamc3
Bmpr2	GGTLC1	RAD23B	Prrc2b
Bmpr2	Sep-07	TOX4///LCP1	Fam102a
Nbeal1	IVNS1ABP	LCK	Stxbp1
Nbeal1	LDB1	PHKG2	Fam129b
Pard3b	CMTM3	BCKDHA	9430024E24Rik
Pard3b	MTMR11	CD74	Mapkap1
Nrp2	ATXN7L2	ADSS	Phf19
Nrp2	INHBC	TUBG1	Gm13446
Nrp2	FANCF	SOX4	Zbtb6
Nrp2	RIOK2	CDC20	Lhx2
Nrp2	BCAN	DLEU2	Wdr38
Nrp2	ELA3A	CD19	Gm16523
A430093A21Rik	PCDH18	CD97	U7
AK017924	POM121L1P	NTHL1	1700019E08Rik
Klf7	FLJ39739	CD22	Rnd3
Klf7	KIAA1712	IL13RA2	Rbm43
Klf7	LYRM7	CD180	Nmi
Creb1	UQCRC2	MS4A2///MS4A1	Gm13540
Ikzf2	ELMOD3	IGKC	Nr4a2
Ikzf2	SNRPF	PSMB10	Acvr1c
AK084284	PTPN6	NFKBIA	Ifih1
AK084284	GAL3ST4	ITGAL	Gca
Mar-04	MVP	IFNGR1	Grb14
Ankar	SLC7A11	TCF7L1///TCF3	Mettl8
Rpl37a	QRSL1	EBI3	Itga6
Rufy4	GLT8D1	IGHG3	8430437L04Rik
Slc11a1	NRAS	MCM7///MCM2	Gm13666
Ctdsp1	MAGI2	CYFIP2	Gm13667
CS444436	TIPRL	CD83	AL772404.1
mmu-mir-26b	CALM2	C5	Gm13699
mKIAA0236	ZNF385A	CCL2	Ctnnd1
Zfp142	LINS1	CDKN2C	Ube2l6
Zfp142	DLX4	PRKDC	Prg2
Zfp142	FOXS1	PMS2L5	Olfr1010
Bcs1l	RBBP5	CKS1B	Sfpi1
Bcs1l	FUS	IKZF1	Ambra1
Stk36	ACSS1	PKMYT1	Ambra1
Stk36	HIST2H3A#HIST2H3C	MAP3K11	Gm13802
mKIAA0236	HIST2H3A#HIST2H3C	PPP5C	Cd82
Zfp142	KBTBD4	MYBL2	Cd82
Zfp142	MYC	DHFR	Gm13818
Zfp142	RNF13	FCGR2C///FCGR2B	Gm13889
Bcs1l	MAGEA1	RPS6KA1	Lrrc4c
Bcs1l	NUP107	PCNA	Cd44
Stk36	ZMAT3	UCP2	Hipk3
Stk36	SF3B14	CD79A	Pax6
Prkag3	RABIF	PMP22///SP110	Lgr4
Prkag3	RIC8A	DEK	Gm13930
Prkag3	RNF5P1	GNAI2	Scg5
Wnt6	ALS2	TNFSF9	7SK
Wnt10a	C3orf25	GNB1	Fam82a2
Fev	MORN4	TAF6	Spint1
Fev	PBX2	SCARB1	Spint1
Fev	PRKRIR	GNAS	Ino80
Fev	SH2B3	GLYAT///CAT	Gm14383
Fev	TMEM205	POLR1A///RPA1	Itpka
Nhej1	C7orf59	PSMB8	Gm13998
Slc23a3	HIST1H2BK	TANK	Mga
1810031K17Rik	CYP1A1	CXXC1///SPP1	Snap23
1810031K17Rik	BBS9	HADH	Frmd5
1810031K17Rik	DEDD	EIF2S2	Frmd5
1810031K17Rik	GNAT1	AURKA	Frmd5
BC038286	GPR84	POLD1///CDC2	Frmd5
BC038286	RGS3	GNB2	Gatm
Glb1l	NUDT9	POLD2	Hdc
Glb1l	RGS3	CTSS	Gm14010
Tuba4a	ILF2	IRF3	Gm14006
AK080399	MGST2	NOL3///MYC	Spef1
Dnajb10	DHX35	CORO1A	Lamr1-ps1
Dnajb10	HSPA4L	VPREB3	Slc23a2
Dnajb10	DLL3	HIST2H2AA3	Fermt1
AK039780	RTDR1	PAX5	Tmx4
Speg	BUD31	SH3KBP1	Sec23b
Speg	C15orf57	LDHA	Dtd1
Speg	RAB3IL1	EXOSC5	Plk1s1
Speg	SPANXN5	RASSF7	Gm14129
Accn4	MFAP4	C6orf32	Fkbp1a
Pax3	ATN1	ALOX5	Tcf15
Pax3	TNPO3	POU2AF1	Rbck1
Pax3	ATP6V0D1	BTK	Trib3
Pax3	BLVRA	STAP1	Gm14472
Pax3	C10orf2	SHC1	BC029722
Pax3	SPANXE	CD52	Ndrg3
Ap1s3	CENPH	ZFP36L1	Blcap
Trip12	LYSMD1	TRAF1	Rprd1b
Trip12	PARP8	CD44	Tgm2
Trip12	C1QC	CD86	Tox2
Fbxo36	ALG9	PRIM1	Ttpal
A630001G21Rik	MYO1B	LGMN	Pkig
A630001G21Rik	THADA	IGLL1	Pkig
A630001G21Rik	ZSCAN5A	RPL21	Pltp
Cab39	S100A6	MCM4	Gm11462
Cab39	ZDHHC4	CKS2	Ncoa3
Cab39	ATF6	INPP5D	Sulf2
Itm2c	ELA3B	LGALS1	Kcnb1
B3gnt7	PHB	E2F1	Gm17544
Ptma	UTP18	DDR1	AL589870.1
Pde6d	XAB2	BLR1	A530013C23Rik
Cops7b	C6orf222	FCER2	Gm14260
6430706D22Rik	BMS1	RNASEH2A///JUNB	Bcas1
A730008H23Rik	ELOVL1	HLA-DPB1	Ppp4r1l-ps
A730008H23Rik	PKD1L1	NPM1	Gm14337
6430706D22Rik	UFM1	SPI1	Npepl1
A730008H23Rik	CDK2AP2	FCGRT	BC066135
A730008H23Rik	WDR54	ARHGDIB	Ogfr
Centg2	HNRNPA1L2	TRAF5	Mrps28
AK036422	CLIP4		Snx16
Gbx2	RARRES3		Car3
Sned1	TWF2		Cypt12
Hdlbp	C7orf55		Cyp7b1
Sep-02	ZNF597		Armc1
Pign	GOLGA9P		Cp
Pig-n	GRINL1A		Slc2a2
Pign	C16orf71		Sec62
2310035C23Rik	NUCB1		4930429B21Rik
2310035C23Rik	SHMT2		Gm12532
2310035C23Rik	PRUNE		Fgf2
Clasp1	WFDC5		U1
Clasp1	ADAMTS13		Gm16508
Clasp1	POFUT1		Phf17
Clasp1	KCNQ1		Maml3
Dbi	NEU1		Eif2a
Dbi	OR56B4		RP23-445D13.2
3110009E18Rik	EEF1G		Schip1
3110009E18Rik	CSF1		Ppm1l
En1	LYSMD4		Gm17213
Ddx18	EVI5L		Pdcd10
Ddx18	AP3S2		Rapgef2
Slc35f5	DPCR1		Fbxw7
Mgat5	SMG7		Prcc
Mgat5	IGFN1		Crabp2
Mgat5	ALDH1B1		AW047730
Mgat5	C17orf63		Slc25a44
AK013506	ZNF516		Sema4a
Ccnt2	LMCD1		Rusc1
Ccnt2	CCDC127		Adam15
Ccnt2			Adar
Lct			Ube2q1
Pfkfb2			Creb3l4
Pfkfb2			Dennd4b
Pfkfb2			Gm6097
Pfkfb2			BC021767
Yod1			Cdc42se1
AA986860			Mcl1
Dyrk3			Bcl9
Lgtn			Gm17608
Lgtn			Gm5544

Lgtn	Trim45
Lgtn	Cd101
Lgtn	Atp1a1
Srgap2	Ngf
Srgap2	U1
Srgap2	Gm5546
2700049P18Rik	U6
Slc45a3	Wnt2b
Slc45a3	Dennd2d
Slc45a3	4933421E11Rik
Slc45a3	Csf1
Elk4	Ampd2
Elk4	1700010K24Rik
Elk4	Vav3
Pctk3	Cdc14a
Lemd1	Cdc14a
Lemd1	Ccdc76
mmu-mir-135b	snoCD11
Nuak2	U6
AK155877	A530020G20Rik
Nfasc	Gm4609
Nfasc	Mir760
Nfasc	Fnbp1l
Snrpe	7SK
Zc3h11a	Larp7
Zc3h11a	Pla2g12a
Zc3h11a	D630013G24Rik
Zc3h11a	Gbp2
Zc3h11a	Sh3glb1
Atp2b4	Clca5
Chit1	Ddah1
Adora1	Fam73a
Adora1	Usp33
Adipor1	AI606473
Adipor1	Depdc1a
Rabif	Wls
Csrp1	Vmn1r-ps2
Csrp1	Lyn
Cacna1s	snoU54
5730559C18Rik	Gm11803
5730559C18Rik	Tox
5730559C18Rik	Chd7
5730559C18Rik	2310030N02Rik
Nek7	Esrp1
Nek7	Rad54b
Nek7	Calb1
Nek7	Nbn
Dennd1b	U6
Dennd1b	U6
AK019844	RNaseP_nuc
Cdc73	Ndufaf4
Trove2	Bach2
Uchl5	Casp8ap2
AK144829	Mob3b
Ptgs2	Gm12369
Ivns1abp	U7
Ivns1abp	Gm12397
1200016B10Rik	Gm12397
1190005F20Rik	Kif24
1190005F20Rik	Tpm2
1190005F20Rik	Creb3
1190005F20Rik	Glipr2
1190005F20Rik	Gne
1200016B10Rik	AL772376.2
1190005F20Rik	Mcart1
1190005F20Rik	5830415F09Rik
1190005F20Rik	5830415F09Rik
1190005F20Rik	Nr4a3
1190005F20Rik	Stx17
Rgl1	Invs
Rgl1	Amd-ps4
Smg7	Gm12466
Smg7	Txn1
Smg7	Gm12579
Smg7	AI314180
Nmnat2	E130308A19Rik
Lamc1	Tnfsf15
Npl	Gm11229
Rgs16	Gm11256
Rnasel	Gm12913
Rnasel	Gm11411
Rnasel	Nfib
Rnasel	Ttc39b
AK044033	U6
Glul	Dennd4c
AK029591	Gm12646
Ier5	Gm12597
Mr1	Ifna13
Mr1	4930553M12Rik
Stx6	Gm12639
Stx6	Gm17636
Lhx4	BC042782
Lhx4	Cyp2j13
Tor1aip1	Gm12676
Lap1B	Gm12676
Tor1aip2	Gm12852
Ifrg15	Gm12681
Ifrg15	Slc35d1
Ifrg15	Acot11
Tor1aip2	Ssbp3
Tor1aip2	Gm12786
Tor1aip1	Gm12743
Lap1B	Zfyve9
Tor1aip2	Tal1
Ifrg15	Lrrc41
Ifrg15	Mast2
Ifrg15	Ptch2
Tor1aip2	Rnf220
Tor1aip2	Ptprf
Soat1	Ybx1
Soat1	Edn2
Ralgps2	Sf3a3
Ralgps2	Gnl2
Ralgps2	Zc3h12a
Ralgps2	Rnf19b
Ralgps2	Lck
Ralgps2	Tinagl1
Ralgps2	Serinc2
Ralgps2	Ythdf2
Ralgps2	Gm13033
Ralgps2	Wdtc1
Rasal2	Gm12974
Rasal2	Slc30a2
Rasal2	Syf2
2810025M15Rik	Id3
Sec16b	Tcea3
Sec16b	Zfp46
Sec16b	6030445D17Rik
Rfwd2	Gm13003
Rabgap1l	Hspg2
Rabgap1l	Alpl
Dars2	Ece1
Dars2	Eif4g3
Cenpl	Kif17
Cenpl	Kif17
Dars2	D4Ertd22e
Dars2	Ddi2
Cenpl	4930455G09Rik
Cenpl	Gm10565
Klhl20	Gm13062
Klhl20	Prdm2
Klhl20	U1
Klhl20	Dhrs3
Ankrd45	Gm13229
AI848100	Gm13207
AK135878	Gm13201
Bat2d	Gm13203
Bat2d	Gm17029
Bat2d	Clstn1

Bat2d	Gpr157
Atp1b1	Rere
Iqwd1	Gm16079
AK008044	Espn
Brp44	Arhgef16
BC056189	Ski
C030014K22Rik	Ski
Uck2	5830444B04Rik
Fcgr3	Dvl1
Fcgr3	1700015F17Rik
Fcgr3	Gm15459
1700009P17Rik	Gm9758
Pcp4l1	Pmpcb
Pcp4l1	Dnajc2
Nr1i3	5031425E22Rik
Nr1i3	Srpk2
Fcer1g	Gm9836
Ndufs2	Accn3
Adamts4	Smarcd3
Adamts4	Nub1
B4galt3	1500035N22Rik
Ppox	Gm5551
Usp21	Rnf32
Usp21	Ppm1g
Dedd	AC114613.1
Dedd	Gm15613
Pfdn2	Whsc1
Dedd	2310079F23Rik
Dedd	Cno
Pfdn2	Cytl1
Dedd	4930421P07Rik
Dedd	Gm5292
Pfdn2	Prom1
Nit1	Med28
Nit1	Gm19031
Pfdn2	Ppargc1a
Arhgap30	Sel1l3
mFLJ00267	Gm15975
Usf1	Tbc1d1
Usf1	Tbc1d1
Usf1	Tbc1d1
Usf1	Klhl5
AK085275	1110003E01Rik
F11r	Slc30a9
AK085275	Atp8a1
F11r	Gm15477
Refbp2	Lrrc66
Vangl2	Lrrc66
Wdr42a	1700094C10Rik
Wdr42a	Usp46
Wdr42a	Gm2199
Wdr42a	Ugt2b5
Wdr42a	Ugt2b38
Wdr42a	Mob1b
Igsf8	Dck
Atp1a2	7SK
Atp1a2	Cxcl1
Igsf8	Sowahb
AK017125	2010109A12Rik
Vsig8	Kat2b-ps
Vsig8	A930011G23Rik
Cadm3	Tmem150c
Cadm3	Gbp11
Cadm3	Gm16834
Rgs7	Lrrc8b
Cep170	Tgfbr3
Sdccag8	Gm9727
Sdccag8	Pde6b
Sdccag8	Myo18b
Sdccag8	Tmem211
Zfp238	Oasl2
Zfp238	Pxn
Zfp238	Hrk
AK082514	AC111115.1
Zfp238	Sdsl
Zfp238	Ddx54
Zfp238	Oas3
AK082514	Oas1b
Adss	Pptc7
Adss	P2rx4
EG545391	Kdm2b
EG545391	U2
Efcab2	Vps37b
Ahctf1	Scarb1
Itpkb	Ubc
H3f3a	Tmem132c
H3f3b	Abhd11
Lefty2	Bcl7b
Pycr2	Rhbdd2
Pycr2	Tmem120a
Tmem63a	Srcrb4d
Tmem63a	Rasa4
Cnih3	Emid2
Lbr	Plod3
Enah	Ephb4
Enah	Zkscan1
Iars2	Heatr2
Iars2	Adap1
Iars2	Iqce
Bpnt1	Foxk1
Iars2	Gm15708
Iars2	Gm15708
Iars2	Bud31
Bpnt1	Usp12
Iars2	Pan3
Iars2	n-R5s179
Iars2	Uspl1
Bpnt1	Stard13
Eprs	Slc25a13
Eprs	Col28a1
Eprs	Nxph1
Eprs	Gm15529
Tgfb2	Capza2
Tgfb2	Gm16854
Rrp15	SNORA17
AK020767	Fscn3
Ptpn14	2210408F21Rik
Ptpn14	B230378P21Rik
Ptpn14	Gm13860
Ptpn14	Tmem140
Ptpn14	Slc13a4
Ptpn14	AC157101.1
Ptpn14	Tbxas1
Prox1	U6
Prox1	Slc37a3
AK142161	Rab19
Prox1	Mrps33
Prox1	Clcn1
AK142161	U6
Prox1	Cul1
Prox1	Ezh2
AK142161	2410003K15Rik
Prox1	AC158667.1
Prox1	Mpp6
AK142161	Gm15051
Tatdn3	Jazf1
Tatdn3	Gm15527
Nsl1	Vmn1r6
Nsl1	Lancl2
Atf3	Gng12
Atf3	Igkv1-135
AK021325	Igkv2-112
Ppp2r5a	Igkv10-95
Ppp2r5a	Gm11065
Lpgat1	Kdm3a
Lpgat1	Kdm3a
Lpgat1	Usp39
Lpgat1	Mat2a
Rcor3	Sema4f

Rcor3	Atp6v1b1
AK142593	Gm5138
Rcor3	Kbtbd12
Rcor3	Txnrd3
AK142593	Iqsec1
Hhat	Iqsec1
Hhat	4930471M09Rik
AK006895	Slc6a6
AK145301	Prickle2
Irf6	Frmd4b
Irf6	Frmd4b
Irf6	Foxp1
A130010J15Rik	Foxp1
A130010J15Rik	Foxp1
Crry	Gm6565
Crry	Ppp4r2
Crry	Crbn
Crry	Itpr1
Akap12	Gm16433
Pcmt1	n-R5s165
Pcmt1	Il17re
Pcmt1	Irak2
AK030763	AC147047.1
Lats1	Rassf4
Lats1	Cxcl12
BC013529	Zfp239
BC013529	Adipor2
BC013529	Kdm5a
AK165776	Cecr6
Ppil4	Gm15856
Ppil4	Usp18
Ust	Rimklb
Sash1	Lpcat3
SASH1	Atn1
Samd5	Chd4
AK139890	Tapbpl
Utrn	Cd9
Utrn	Gm16303
Utrn	U6
AK045519	Gm4968
4930519B02Rik	7SK
Ltv1	Gm10069
Pex3	Clec2h
Pex3	Klrb1f
Pex3	Gm15854
Deadc1	Magohb
Hivep2	Magohb
Hivep2	Styk1
Hivep2	Bcl2l14
Hivep2	Crebl2
Hivep2	Art4
Hivep2	Pyroxd1
Hivep2	Bcat1
Hivep2	Rps25-ps1
Hivep2	Tuba3b
3110003A17Rik	Stk38l
3110003A17Rik	Tmtc1
Reps1	4833442J19Rik
AK041760	U1
5730409E15Rik	Ptprh
Nhsl1	Hspbp1
5730409E15Rik	U2af2
Nhsl1	U6
Nhsl1	Zfp773
AK043723	Clcn4-2
Pde7b	Sult2a5
Pde7b	Obox3
Ahi1	Meis3
Ahi-1	Ccdc9
Ahi-1	Bbc3
AK041716	Qpctl
Sgk	Ppp1r37
Sgk	Pvrl2
Sgk	Ceacam16
Sgk	B9d2
Sgk	Map3k10
Sgk	Fbxo27
AK053193	Rinl
Raet1e	Lgals4
Raet1a	Actn4
Raet1e	Gm17440
Raet1c	Hspb6
Eya4	Hamp
Eya4	Gm5058
Moxd1	AC173478.1
Epb4.1l2	Gm12780
L3mbtl3	Slc7a9
L3mbtl3	Nudt19
L3mbtl3	U6
L3mbtl3	Gm9244
Ptprk	Gm10351
Ptprk	Shank1
Ptprk	Nr1h2
Ptprk	Pnkp
Ptprk	Prrg2
Ncoa7	Flt3l
Ncoa7	Bcat2
2700019D07Rik	Bcat2
2700019D07Rik	Gm16047
2700019D07Rik	Tsg101
Hs3st5	Csrp3
Marcks	E2f8
AK017779	Nav2
AK040320	Dbx1
Fyn	U6
Fyn	Igf1r
AK164126	Gm10294
Sez4	Klhl25
Rev3l	Mir7-2
AI317395	Plin1
Bxdc1	Pex11a
Bxdc1	Cib1
Gtf3c6	Gm15880
Gtf3c6	Zfp592
Amd2	Homer2
Gpr6	1700026D08Rik
Foxo3a	Dlg2
Foxo3a	Gm16053
Foxo3a	AC149605.1
Fkhr2	Aqp11
Foxo3a	Pak1
Foxo3a	B3gnt6
Foxo3a	Uvrag
Fkhr2	Uvrag
AK035573	Slco2b1
Sec63	Mrpl48
AK122525	Art2a-ps
Cd24a	Art2b
AK158018	Il18bp
AK158018	Trim21
Prdm1	Trim34a
Prdm1	Fxc1
Prdm1	Olfr497
Prep	Olfr512
Grik2	Gm9105
Grik2	Nrip3
Grik2	Gm10087
Grik2	Mrvi1
Grik2	Mical2
Grik2	Mical2
Slc35f1	Parva
BC062185	AC103388.1
Mcm9	Insc
Asf1a	SNORA17
Man1a	Sox6
Man1a	U6
Man1a	Acsm5
AK138755	Gm15774
D630037F22Rik	Gm14389

Sh3md4
Sh3md4
P4ha1
Cbara1
Cbara1
Cbara1
Cbara1
Ddit4
Ddit4
Ddit4
D10Ertd641e
Ascc1
Ascc1
Ascc1
Ascc1
Pcbd1
Sgpl1
Sgpl1
Sgpl1
Sgpl1
Nodal
AK085995
Lrrc20
AK006239
Sar1a
Tysnd1
Col13a1
Col13a1
Col13a1
Ddx21
Ccar1
Ccar1
Ccar1
Hnrph3
Hnrph3
AK156477
3110049J23Rik
AK018967
Herc4
Herc4
Herc4
Sirt1
Sirt1
Ctnna3
Ctnna3
Ctnna3
Lrrtm3
Lrrtm3
AK079018
Reep3
Reep3
mKIAA1380
Nrbf2
Arid5b
Arid5b
Arid5b
Arid5b
AK029407
Zwint
Zwint
Zwint
1110038D17Rik
1110038D17Rik
1110038D17Rik
1110038D17Rik
1110038D17Rik
Snrpd3
Cabin1
Prmt2
S100b
Prmt2
S100b
Prmt2
S100b
Pcnt
2610028H24Rik
A130042E20Rik
Mcm3ap
Mcm3ap
Lss
Col18a1
Pttg1ip
Sumo3
Sumo3
Ube2g2
Pfkl
Icosl
Ppap2c
Ppap2c
Ppap2c
Ppap2c
Shc2
Gm1157
Bsg
Hcn2
Rnf126
Fstl3
FLRG
Prssl1
Palm
Palm
9130017N09Rik
9130017N09Rik
9130017N09Rik
Med16
Med16
C030046I01Rik
Kiss1r
Arid3a
Arid3a
mFLJ00277
ORF61
Cnn2
Abca7
Abca7
mFLJ00277
ORF61
Cnn2
Abca7
Abca7
Abca7
Dos
Dos
Atp5d
Midn
Midn
Cirbp
1600002K03Rik
Efna2
Cirbp
1600002K03Rik
Efna2
Adamtsl5
Adamtsl5
6330514A18Rik
Mex3d
Mbd3
Mbd3
Mbd3
Btbd2
Btbd2
Mknk2
Mknk2
Mnk2
Ap3d1
Dot1l
Dot1
Ap3d1
Dot1l

Tnrc6a
Arhgap17
Aqp8
Spns1
Mvp
Qprt
Fbxl19
Fus
Bag3
Ctbp2
Mki67
Ppp2r2d
Bnip3
Inpp5a
1190003J15Rik
Slc25a22
Pnpla2
Krtap5-5
Mir3105
Fgf4
Fgf15
Ccnd1
Tpcn2
Ccl25
Efnb2
Tnfsf13b
3930402G23Rik
9530052E02Rik
Mcf2l
Rasa3
4932443I19Rik
Fbxo25
SNORA70
Defa-ps17
Tpte
Adam5
Plekha2
Whsc1l1
1700024N20Rik
Snord13
Fut10
Leprotl1
Tmem66
Tusc3
Cyp4v3
Tlr3
Tlr3
Cdkn2aip
Psd3
Gm15716
Lzts1
Lzts1
Hapln4
Slc25a42
B3gnt3
SNORA50
Nr3c2
4933431K23Rik
U6
Zfp827
Elmod2
Ptger1
Gpt2
SNORA17
N4bp1
Cyld
9330175E14Rik
Arl2bp
Ccl22
4930513N10Rik
U6
Car7
Pdp2
Psmb10
Gm10073
Has3
C630050I24Rik
Mir140
4922502B01Rik
Ap1g1
Calb2
Glg1
Mlkl
Wdr59
Osgin1
Taf1c
Kcng4
4632415K11Rik
Gse1
Irf8
Fbxo31
Jph3
Slc7a5
Car5a
Gm17709
Zfpm1
Rab4a
Gm16983
Cwf19l2
Arhgap42
Mre11a
Hephl1
BC017612
1810064F22Rik
Gm10180
Glb1l3
Jam3
Dcps
Stt3a
Vwa5a
Tmem225
Zfp202
1700110K17Rik
Gramd1b
Gramd1b
D230004N17Rik
Pvrl1
C030014I23Rik
C030014I23Rik
C030014I23Rik
Atp5l
Ppp2r1b
Pou2af1
Kdelc2
Tnfaip8l3
Gldn
Psma4
Chrnb4
Pstpip1
Scamp2
Nptn
Gm7589
AC113291.1
Pias1
Smad3
Megf11
Spg21
Spg21
Zfp609
Zfp609
Dapk2
Tpm1
Vps13c
Gcnt3
Myo1e
Myo1e
Myzap
U6
Mns1

Dot1		Rfx7
Dot1l		U6
Plekhj1		n-R5s86
Plekhj1		Tmod3
Sf3a2		Gsta1
Amh		Myo6
Lmnb2		U6
Lmnb2		Bckdhb
Gadd45b		Me1
Lmnb2		Snx14
Gadd45b		Plscr2
Gng7		Cep70
Gng7		1600029I14Rik
Sirt6		Msl2
Sirt6		7SK
Ankrd24		Cpne4
mKIAA1981		Rpl29
mKIAA1981		Parp3
Ankrd24		Gm9917
Mif1		Dag1
Glt8d2		Qars
Hcfc2		Nckipsd
Rfx4		Shisa5
Rfx4		Setd2
Rfx4		Nbeal2
Btbd11		Ccdc12
Bpil2		Ccrl2
Fbxo7		7SK
Fbxo7		Clasp2
Syn3		Cnot10
Syn3		Gm16142
Syn3		Eomes
Syn3		7SK
Hsp90b1		Higd1a
BC030307		Sacm1l
BC030307		Esr1
BC030307		Esr1
BC030307		U1
Nt5dc3		Sf3b5
Nt5dc3		Gm16234
Nt5dc3		Olig3
Nt5dc3		Gm6846
Ascl1		E030030I06Rik
Ascl1		H60b
Ccdc53		Epb4.1l2
Ccdc53		Zufsp
Arl1		Zufsp
Utp20		Cep57l1
Pctk2		Cep57l1
Pctk2		Scml4
mmu-mir-1931		Gm16546
Lta4h		Gm10812
Lta4h		Prdm1
Metap2		Hace1
Metap2		SNORA25
Metap2		Mcm9
AK029692		Man1a
Plxnc1		Ccdc109a
AK202794		Col13a1
Nudt4		Hk1
mmu-mir-3058		Tet1
Btg1		Mypn
Atp2b1		1700120B22Rik
Atp2b1		Dnajc12
Tmtc3		Ctnna3
Tmtc3		Ccdc6
Alx1		Gm4798
Tmtc2		1110038D17Rik
Tmtc2		Slc5a4b
8430416H19Rik		Adarb1
8430416H19Rik		Aire
Pawr		Icosl
Pawr		Pdxk
Tmem19		Ppap2c
Tmem19		9130017N09Rik
Tmem19		Sbno2
Tmem19		Gadd45b
Thap2		Gm3828
Ccdc131		Ncln
Lgr5		Gna11
Lgr5		Txnrd1
Lgr5		Gm6721
Ptprb		Syn3
Gm239		Syn3
Frs2		Nr1h4
Frs2		Tmpo
Cpsf6		Tmcc3
Cpsf6		Tmcc3
Cpsf6		5S_rRNA
Cpm		Eea1
Dyrk2		U6
Cand1		Osbpl8
Cand1		Cnot2
Grip1		Gm239
Grip1		Grip1
Grip1		9230105E05Rik
Grip1		Msrb3
Helb		Gns
Hmga2		Tbk1
Hmga2		Tbk1
Hmga2		Ppm1h
Hmga2		Ppm1h
Msrb3		Lrig3
Gns		Agap2
Gns		Lrp1
AK081796		Stat6
Ppm1h		Erbb3
Ppm1h		Rdh5
Ppm1h		Olfr782
Ppm1h		AC132266.1
Ppm1h		Gm12592
AI851790		Lif
Tsfm		Gm11958
Tsfm		Ascc2
Tsfm		Ap1b1
Mettl1		Kremen1
Mettl1		SNORA17
Cyp27b1		Gm12019
	Mar-09	Bcl11a
Tspan31		Bcl11a
Centg1		Bcl11a
C78409		Gm12107
D10Ertd610e		Cpeb4
D10Ertd610e		Stk10
Dtx3		Gm10462
Dtx3		Rars
Dtx3		Rnf145
Deltex3		Gnb2l1
AK077682		Mapk9
Pip4k2c		Mgat4b
Mbd6		Mir804
Mbd6		Agxt2l2
Ddit3		Cdkl3
Gli1		Ppp2ca
Gli1		Gm12225
Gli1		Fnip1
Gli1		Fnip1
Gli1		Tnip1
Gli1		Sparc
Lrp1		Gm12243
Lrp1		Irgm2
Lrp1		2210407C18Rik
Stat6		Gm12714
Stat6		Rasd1
Nab2		Pemt
Nab2		Rai1
BC030440		Tom1l2
BC030440		Top3a
Kiaa0286		Aldh3a1

TIP5	2810001G20Rik
Baz2a	Zkscan6
Baz2a	9130409J20Rik
Baz2a	Ccdc42
Baz2a	U6
Rbms2	2310047M10Rik
Rbms2	Chd3
Gls2	Chd3
Rbms2	Kdm6b
Rbms2	Plscr3
Gls2	Cldn7
Gls2	Xaf1
Rbms2	Trpv3
Rbms2	Aspa
Gls2	Olfr404-ps1
Gls2	Mnt
Myl6	Slc6a4
Myl6	Ssh2
Myl6b	Spag5
AK040027	Gm11201
Mbc2	Rab11fip4
Mbc2	1110002N22Rik
Zc3h10	4930507D10Rik
Rpl41	Spaca3
Rpl41	Gm11426
Zc3h10	Slfn2
Rpl41	RP23-381B19.7
Rpl41	Synrg
Zc3h10	Gm11437
Rpl41	Bcas3
Rpl41	Gm11477
Pa2g4	Med13
Zc3h10	5S_rRNA
Rpl41	Cltc
Rpl41	Cltc
Pa2g4	Trim37
Erbb3	Mtmr4
Rps26	U6atac
Rps26	7SK
Ikzf4	Mmd
Ikzf4	SNORA17
AK047988	Tob1
AK206637	Ankrd40
Rab5b	Phb
Rab5b	Mir10a
Rab5b	Gm11525
Si	Tbkbp1
Rab5b	Arhgap23
Rab5b	Erbb2
Rab5b	Nr1d1
Cdk2	Krt20
Cdk2	2300003K06Rik
Si	Gm11566
Rab5b	Krt15
Rab5b	Cnp
Rab5b	Rab5c
Cdk2	Stat3
Cdk2	Stat3
Si	Ifi35
Dgka	Tmem106a
Wibg	Atxn7l3
Wibg	Grn
Dgka	Gm11627
Wibg	Gjc1
Wibg	Fam187a
1110012D08Rik	4933400C05Rik
1110012D08Rik	Gm11648
Ormdl2	Nsf
1110005A23Rik	Nsf
1110005A23Rik	Gm11672
Patz1	Gm11707
mazr	Gm11649
Patz1	Abca8a
Patz1	Kcnj2
Patz1	Cdc42ep4
AK177174	Cdc42ep4
Pik3ip1	Gm11692
Pik3ip1	Nat9
Hgfl	Mir3968
Slc35e4	Slc16a5
Tcn2	Myo15b
Tcn2	H3f3b
Tcn2	Gm11744
Tcn2	Tmc6
Pes1	Socs3
Gal3st1	Gm11761
1700020C11Rik	Aatk
Sec14l2	Aatk
Lif	P4hb
Lif	Lrrc45
Lif	Gm11774
Lif	Sectm1b
Zmat5	Gm11792
Zmat5	Dnmt3a
Nipsnap1	Dnajc27
Nefh	Ncoa1
Ap1b1	5S_rRNA
Gas2l1	AC122860.1
Gas2l1	Wdr35
Gas2l1	Osr1
Ewsr1	1700034J04Rik
Ewsr1	2410018L13Rik
Ewsr1	Rsad2
Rhbdd3	Sntg2
Rhbdd3	Fam110c
Kremen1	2010109K11Rik
Znrf3	Gdap10
Znrf3	Snx13
Ankrd48	Agmo
Ankrd36	Scin
Ankrd36	Zfp277
Ankrd36	U6
Polm	Mbip
Ddx56	C87198
Ddx56	Gm17529
Tmed4	Ctage5
Ogdh	Psma3
Ogdh	RP23-113F14.3
Ogdh	Trmt5
AK009365	Tmem30b
Purb	Gm11042
AK009365	Spnb1
Purb	Fntb
Tbrg4	Max
Tbrg4	Atp6v1d
Upp1	Gm17194
Upp1	Zfp36l1
Upp1	C130039O16Rik
Upp1	C130039O16Rik
Upp1	Abcd4
Upp1	Ltbp2
Fignl1	Dlst
Fignl1	Fam164c
Fignl1	0610007P14Rik
Fignl1	U6
Fignl1	Pomt2
Fignl1	Gm2022
Ddc	Ston2
Ddc	3300002A11Rik
Ddc	Foxn3
Sec61g	Foxn3
Sec61g	Gm17043
Egfr	Clmn
Egfr	4831426I19Rik
Ppp3r1	Setd3
Ppp3r1	Ccdc85c
Spred-2	Evl
Spred2	Dio3
Actr2	Ppp2r5c

Actr2	Cdc42bpb
Slc1a4	1200009I06Rik
Aftph	Tnfaip2
Aftph	Akt1
Aftph	4930427A07Rik
Otx1	Ighv6-6
Otx1	Cdca7l
Ehbp1	Sp4
Ehbp1	Sp8
Ehbp1	Gm17177
Ehbp1	BC016423
Ehbp1	Asb13
Ehbp1	Gm9742
Ehbp1	Gm17294
Ehbp1	Ggps1
B3gnt2	U6
B3gnt2	Arid4b
B3gnt2	U1
4930430E16Rik	Olfr1361
4930430E16Rik	Olfr1359
4930430E16Rik	Hist1h2bl
AK149094	Gm11274
Usp34	Hist1h4n
Pex13	Zfp184
Ccdc139	Gm11291
Ccdc139	Gm11292
Ccdc139	Hist1h4d
Pex13	Hfe
Ccdc139	Lrrc16a
Ccdc139	Prl3d3
Ccdc139	2610307P16Rik
Pex13	Gm11370
Ccdc139	Hus1b
Ccdc139	Gm5447
Ccdc139	Foxq1
Papolg	Serpinb9
Papolg	Serpinb6a
Bcl11a	Psmg4
Bcl11a	Cdyl
Bcl11a	Nedd9
Bcl11a	Ccdc90a
Bcl11a	C030044B11Rik
Bcl11a	1110007C09Rik
Bcl11a	Susd3
Bcl11a	Nxnl2
Bcl11a	S1pr3
Bcl11a	Cks2
Bcl11a	Gadd45g
Bcl11a	Syk
Bcl11a	Sfxn1
Bcl11a	Gm6344
Bcl11a	Fgfr4
Bcl11a	Rgs14
Bcl11a	Golm1
Bcl11a	Fbp1
Bcl11a	Zfp748
Bcl11a	Irx4
Bcl11a	Slc12a7
Bcl11a	Gm10126
Bcl11a	Pdcd6
Bcl11a	Erap1
Smek2	Mctp1
Smek2	Rasa1
Smek2	U6
A430106J12Rik	Jmy
Ccdc88a	Lhfpl2
Rps27a	F2rl1
RP23-92B18.7	AC137692.1
Rtn4	Gcnt4
Rtn4	Tnpo1
C230094A16Rik	Gm807
Stc2	Ptcd2
Stc2	Naip3
Cpeb4	AC165159.1
Cpeb4	Mast4
Cpeb4	Adamts6
Cpeb4	Adamts6
Cpeb4	Gm2822
Cpeb4	Plk2
Ubtd2	Gm6320
Ubtd2	4833420G17Rik
1700007I06Rik	Gm3460
Stk10	Gm6356
mKIAA4026	Pxk
Fgf18	Oit1
Npm1	Olfr721-ps1
Npm1	Nr1d2
Npm1	Nkiras1
Fgf18	Gm2244
Npm1	SNORA70
Npm1	Comtd1
Npm1	Zmiz1
Ranbp17	SNORA71
Ranbp17	Arf4
Kcnip1	Sh3bp5
Kcnip1	3425401B19Rik
Kcnip1	Anxa8
Kcnip1	SNORA17
Odz2	9230112D13Rik
ten-m2	n-R5s46
Gabrb2	Mat1a
Gabrb2	Gm2959
mmu-mir-146a	Ear-ps12
Ccnjl	Gm17184
Ccnjl	Ptger2
Ttc1	Gm7206
Rnf145	Mapk1ip1l
Rnf145	Peli2
Rnf145	Olfr741
Rnf145	Olfr1507
Lsm11	Abhd4
Gnb2l1	Cebpe
Trim41	Psme1
Trim7	Psme2
Trim7	Ltb4r1
Trim41	Sdr39u1
Trim7	Gzmb
Trim7	Parp4
AK029860	Spata13
AK029860	Wdfy2
Btnl9	Fdft1
Zfp62	Xkr6
Zfp62	Msra
Zfp62	Hmbox1
Zfp62	Pbk
Zfp62	Gm10233
Btnl9	Rhobtb2
Zfp62	Bmp1
Zfp62	Dok2
Zfp62	Fndc3a
Zfp62	Lrch1
Zfp62	Slc25a30
Flt4	Nufip1
Cnot6	Nufip1
Cnot6	Tsc22d1
Gfpt2	AC124745.1
Mapk9	Elf1
Jnk2	SNORA17
Mapk9	Mzt1
Mapk9	Dis3
Sqstm1	Klf5
Mgat4b	U2
AK157916	Commd6
Sqstm1	Lmo7
Mgat4b	Spry2
AK157916	1110020G09Rik
Sqstm1	Mtmr12
Mgat4b	Pdzd2
AK157916	Cdh6

Mgat4b	Cdh18
AK157916	5S_rRNA
Canx	Gm10384
Canx	Gm10384
Hnrph1	Oxr1
Hnrph1	U2
Hnrph1	Mtbp
mmu-mir-804	Sqle
Rufy1	Myc
9630041N07Rik	Pvt1
BC021919	Fam49b
Grm6	Adcy8
Grm6	Eif2c2
Grm6	Slc45a4
Hnrpab	Slc45a4
Hnrpab	4930572J05Rik
Nola2	Ly6a
Rmnd5b	1110038F14Rik
D930048N14Rik	Apol11a
Rmnd5b	Apol11b
D930048N14Rik	Apol7e
0610009B22Rik	U6
Phf15	Kctd17
Phf15	Lgals2
Phf15	Cbx6
Cdkn2aipnl	SNORA17
Ube2b	Fam83f
Cdkl3	Gm17025
Cdkl3	Gm8444
Cdkl3	1500032L24Rik
Cdkl3	Cyp2d26
Cdkl3	Gm17401
Cdkl3	Cpne8
Skp1a	4933438A12Rik
AK202663	Lrrk2
Vdac1	Adamts20
Fstl4	Pus7l
Hspa4	Twf1
Hspa4	AC113178.1
AK038627	Slc38a2
Zcchc10	SNORA17
Zcchc10	4930578M01Rik
Irf1	Troap
Irf1	Lima1
Irf1	RP24-73K9.3
Tnip1	Cela1
Tnip1	Galnt6
Tnip1	Acvrl1
Tnip1	Krt8
Atox1	Krt8
G3BP	Krt18
G3bp1	Atf7
AK007998	Hoxc6
Sap30l	Cbx5
Cnot8	Pde1b
Cnot8	Nudt16l1
Cnot8	Abat
Cnot8	Ciita
Zfp692	Ciita
Zfp692	Socs1
Zfp672	Gm11172
Zfp672	Gm4262
Zfp672	Cpped1
Sh3bp5l	Bfar
Sh3bp5l	Mapk1
Sh3bp5l	Rimbp3
Sh3bp5l	Trmt2a
Sh3bp5l	Tbx1
Zfp672	Abcc5
Zfp672	Vps8
Zfp672	Gm17439
Sh3bp5l	Igf2bp2
Sh3bp5l	Tra2b
Sh3bp5l	Snord2
Sh3bp5l	Il1rap
Sh3bp5l	Lsg1
1810065E05Rik	Gm15742
Zfp39	Apod
Butr1	Osta
Butr1	Muc4
Rnf187	Kalrn
Hist3h2ba	Hspbap1
Hist3h2a	Parp14
Trim17	Gm10237
Trim17	Parp9
Rnf187	U6
Hist3h2bb	Cd86
Hist3h2a	Cd86
Trim17	Hgd
Trim17	Nr1i2
Wnt3a	Cox17
Wnt9a	Cox17
Wnt3a	Arhgap31
Wnt9a	Gm15803
Wnt3a	4932412D23Rik
Wnt9a	Gramd1c
Wnt3a	Slc35a5
Wnt9a	U6
1110031B06Rik	Cd47
Jmjd4	Cblb
Jmjd4	Senp7
1110031B06Rik	Tfg
Jmjd4	Gpr128
Jmjd4	2610528E23Rik
BC050078	Gpr15
Zkscan17	Pros1
Zfp496	Cggbp1
Zkscan17	AC163694.1
Zkscan17	Robo1
BC099591	Usp25
AK163429	Rwdd2b
AK030920	Bach1
Flcn	2310061N02Rik
Flcn	Krtap20-2
AK144909	Donson
Pemt	Cryzl1
Pemt	Mir802
Rai1	Cbr1
Rai1	2310043M15Rik
Rai1	Ttc3
Rai1	Dyrk1a
Rai1	Lca5l
Rai1	Pcp4
Rai1	Prdm15
Rai1	5730437N04Rik
Rai1	Ppp1r2-ps6
Rai1	Dynlt1a
Rai1	Tmem181b-ps
Rai1	Unc93a
Rai1	U6
Rai1	Tcte2
Rai1	Gm7358
Rai1	Prdm9
Llgl1	Prdm9
Llgl1	Zfp97
Top3a	Vmn2r90
Top3a	Vmn2r-ps118
Top3a	Fpr-rs3
Smcr8	Zfp160
Shmt1	Zfp943
Shmt1	Zfp758
Dhrs7b	Vmn2r-ps131
Dhrs7b	Zscan10
Tmem11	1520401A03Rik
AK140192	E4f1
Aldh3a2	Haghl
Mapk7	Axin1
Mapk7	Bak1
Mapk7	Hmga1

Mapk7	E230001N04Rik
BC058096	Brpf3
B9d1	Srsf3
Ncor1	Srsf3
Ncor1	Ftsjd2
Ncor1	Dnahc8
Ncor1	Slc37a1
Ncor1	Gm17276
Ncor1	Ephx3
Ncor1	Brd4
Ncor1	Cyp4f16
Ncor1	Cyp4f16
Ncor1	Cyp4f16
Ncor1	Cyp4f40
Pigl	Gm4461
Prr6	Rps28
Ubb	Tapbp
AK010427	Rxrb
AK157519	Psmb9
BC046404	Psmb8
BC046404	Tap2
BC046404	Btnl5
AK005754	Btnl6
BC046404	Btnl7
BC046404	Btnl7
BC046404	Notch4
BC025076	Gm11131
BC025076	Cchcr1
BC025076	Gm20443
BC046404	H2-T24
BC046404	H2-T23
BC025076	H2-Bl
BC025076	Gm6659
BC025076	H2-T3
Cdrt4	Gm8835
Cdrt4	Trim26
Pmp22	Trim31
Pmp22	Pgk2
Hs3st3b1	Cd2ap
Hs3st3b1	Tdrd6
Hs3st3b1	Enpp4
Elac2	Aars2
AU040829	Hsp90ab1
OTTMUSP00000006088	Mrpl14
AU040829	Gm16862
OTTMUSP00000006088	Ppp2r5d
Zkscan6	Guca1a
Gm879	1700001C19Rik
Ntn1	Ccnd3
Ntn1	Sult1c2
Arhgef15	Pot1b
Arhgef15	Hdgfrp2
Arhgef15	Vav1
1500010J02Rik	U1
1500010J02Rik	4930405O22Rik
Aurkb	Man2a1
1500010J02Rik	1110012J17Rik
1500010J02Rik	RP23-349B16.3
Aurkb	Ypel5
1500010J02Rik	Lclat1
1500010J02Rik	Birc6
Aurkb	Gm17538
Aurkb	Prkd3
2310047M10Rik	Atl2
Aurkb	U6
2310047M10Rik	Prkce
Aurkb	Mcfd2
2310047M10Rik	1700011E24Rik
Aurkb	Calm2
2310047M10Rik	Msh2
Aurkb	U6
2310047M10Rik	Gm6741
2310047M10Rik	U6
Tmem107	7SK
Tmem107	Usp14
Tmem107	Ss18
Tmem107	Dsg1a
Tmem107	Rit2
Tmem107	Sap130
Vamp2	Epb4.1l4a
Tmem107	Srp19
Tmem107	n-R5s24
Vamp2	Ctnna1
Tmem107	Ankhd1
Tmem107	Taf7
Vamp2	AC134576.1
Tmem107	Arhgap26
Tmem107	Gm15333
Vamp2	Prelid2
Vamp2	Sh3rf2
Per1	4933407I08Rik
Per1	Spinkl
Per1	Sema6a
Per1	Dmxl1
Per1	Tnfaip8
Per1	Hsd17b4
Per1	Zfp608
Per1	Gramd3
Hes7	BC023105
Aloxe3	AC116595.1
Hes7	Ppargc1b
Aloxe3	Ppargc1b
Hes7	Atp8b1
Aloxe3	U6
Hes7	Lman1
Aloxe3	Gm15958
Cyb5d1	7SK
Tmem88	Mro
Jmjd3	Myo5b
Jmjd3	Atp5a1
Cyb5d1	Cyb5
Tmem88	Cyb5
Jmjd3	Ndufs8
Jmjd3	Unc93b1
Jmjd3	BC021614
Cyb5d1	Pitpnm1
Tmem88	Ssh3
Jmjd3	Syt12
Jmjd3	Yif1a
Jmjd3	Sf3b2
Cyb5d1	Snx32
Tmem88	Map3k11
Jmjd3	Gpha2
Jmjd3	Ccdc88b
Jmjd3	Ms4a12
Cyb5d1	Olfr1420
Tmem88	Osbp
Jmjd3	Dtx4
Jmjd3	Fam111a
Jmjd3	Gm8250
Cyb5d1	Pip5k1b
Tmem88	Gm10053
Jmjd3	Cd274
Jmjd3	Il33
Jmjd3	U1
Tmem88	Sgms1
Jmjd3	Pten
Jmjd3	Gm7237
Jmjd3	Gm7237
Tmem88	AC157912.1
Jmjd3	Ifit1
Jmjd3	Hectd2
Jmjd3	Cpeb3
Jmjd3	Cpeb3
Jmjd3	Cyp2c67
Jmjd3	Pdlim1
Dnahc2	Aldh18a1
Jmjd3	Entpd1
Jmjd3	Lcor

Jmjd3
Dnahc2
Jmjd3
Dnahc2
Dnahc2
Dnahc2
Efnb3
Wdr79
Wdr79
p53
Trp53
Trp53
Wdr79
Atp1b2
Atp1b2
Sat2
Sat2
Sat2
Atp1b2
Sat2
Sat2
Sat2
Shbg
Fxr2
Mpdu1
Supl15h
Cd68
mmu-mir-1934
Polr2a
Amac1
Zbtb4
Zbtb4
Zbtb4
Polr2a
Amac1
Zbtb4
Zbtb4
Zbtb4
Polr2a
Amac1
Zbtb4
Zbtb4
Zbtb4
Polr2a
Amac1
Zbtb4
Zbtb4
Zbtb4
Polr2a
Amac1
Zbtb4
Zbtb4
Zbtb4
Chrnb1
Tmem102
Chrnb1
Fgf11
Tmem102
Tmem102
Tnk1
Tnk1
Kctd11
Centb1
Centb1
2810408A11Rik
0610025P10Rik
Gps2
Gps2
2810408A11Rik
Eif5a
Eif5a
Eif5a
Ybx2
2810408A11Rik
Eif5a
Eif5a
Eif5a
Ybx2
2810408A11Rik
Eif5a
Eif5a
Eif5a
Ybx2
Eif5a
Eif5a
Eif5a
Ybx2
Msy2
GLUT4
Slc2a4
Slc2a4
Cldn7
Rai12
Rai12
Rai12
Dullard
Gabarap
Phf23
Phf23
Phf23
Dvl2
Dvl2
Dvl2
Rai12
Rai12
Rai12
Gabarap
Phf23
Phf23
Phf23
Dvl2
Dvl2
Dvl2
mmu-mir-324
Rai12
Rai12
Rai12
Phf23
Phf23
Phf23
Dvl2
Dvl2
Dvl2
mmu-mir-324
Rai12
Rai12
Rai12
Phf23
Phf23
Phf23
Dvl2
Dvl2
Dvl2
mmu-mir-324
Acadvl
Dlg4
Slc16a13
Bcl6b
mmu-mir-497
mmu-mir-195
D11Bwg0434e
D11Bwg0434e
0610010K14Rik
D11Bwg0434e
Alox12
Alox12
D11Bwg0434e
D11Bwg0434e
0610010K14Rik

Pgam1
Avpi1
SNORA17
Abcc2
Dnmbp
Erlin1
Scd3
Sufu
Trim8
Cnnm2
Col17a1
Acsl5
Tcf7l2
Casp7
Nhlrc2
B230217O12Rik
Trub1
1700011F14Rik
Pnliprp1
Gm6020
Gripap1
Gata1
Gm14502
Gm14458
Gm5755
Gm6923
Gm4732
Dock11
AL451076.1
6030498E09Rik
Xiap
9530027J09Rik
7SK
Gm14612
Gpc4
Gm7851
U6
Gm14594
Gm14625
Nsdhl
Mageb18
Gm14780
Fam123b
1700010D01Rik
Gm14933
Gm14808
Gm14876
Gm14886
Gm6429
Gm14940
Vmn2r121
Gm14983
Prps1
Trpc5
Amot
AL808014.1
Gm15101
Itih5l-ps
Shroom2
Phex
Gm6568
Sh3kbp1
U6
U6
Tmsb4x
Mid1

D11Bwg0434e
Alox12
Alox12
D11Bwg0434e
D11Bwg0434e
0610010K14Rik
D11Bwg0434e
Alox12
Alox12
Alox12e
Tm4sf5
C730027E14Rik
Psmb6
MINK
Mink1
MINK
Mink1
Slc25a11
Pfn1
ENO3
Eno3
Eno3
Slc25a11
Pfn1
ENO3
Eno3
Eno3
Pfn1
Spag7
Camta2
mKIAA0909
Camta2
Spag7
Camta2
mKIAA0909
Camta2
AI842396
AI842396
Kif1c
Kif1c
Camta2
mKIAA0909
Camta2
AI842396
AI842396
Kif1c
Kif1c
AK039373
Zfp3
AK039373
Zfp3
Rabep1
Rabep1
4933427D14Rik
mKIAA0753
4933427D14Rik
4933427D14Rik
Med31
AK043242
Ube2g1
Ube2g1
Ankhzn
Ankfy1
1200014J11Rik
1200014J11Rik
1200014J11Rik
Tmem93
Tax1bp3
Tax1bp3
Trpv3
Garnl4
mKIAA1039
Garnl4
mKIAA1039
Garnl4
mKIAA1039
Pafah1b1
Pafah1b1
Pafah1b1
AK039862
Pafah1b1
Pafah1b1
Pafah1b1
AK039862
Pafah1b1
Pafah1b1
Pafah1b1
AK039862
Smg6
BC127131
BC127131
Hic1
Hic1
AK006051
mmu-mir-212
mmu-mir-132
Ovca2
Dph1
Rtn4rl1
Ovca2
Dph1
Rtn4rl1
Rtn4rl1
Myo1c
Myo1c
Myo1c
Myo1c
Glod4
2310047D13Rik
2310047D13Rik
Dbil5
Glod4
2310047D13Rik
2310047D13Rik
Dbil5
Glod4
AK198984
Gemin4
Gemin4
Dbil5
Nxn
Nxn
Nxn
Timm22
Nxn
Nxn
Timm22
Blmh
Efcab5
Ssh2
mSSH-2L
Ssh2
Ssh2
mSSH-2L
Ssh2
Ankrd13b
Ankrd13b
RP23-81E16.2-005
Ankrd13b
Git1
Ankrd13b
Ankrd13b
RP23-81E16.2-005
Ankrd13b
Git1
Ankrd13b
RP23-81E16.2-005
Ankrd13b
Git1

Trp53i13
1300007F04Rik
AK040172
Trp53i13
1300007F04Rik
AK040172
Taok1
Taok1
Nufip2
Myo18a
Myo18a
Myo18a
Myo18a
Pipox
Sez6
Sez6
BC128327
Phf12
Phf12
Phf12
BC128327
Phf12
Phf12
Phf12
Dhrs13
Dhrs13
Flot2
Flot2
Eral1
BC017647
BC017647
Eral1
BC017647
BC017647
Eral1
BC017647
BC017647
BC017647
BC017647
BC017647
Traf4
traf4
Nek8
Tlcd1
Tlcd1
Tlcd1
Rab34
4933404M19Rik
Nek8
Rpl23a
Rab34
4933404M19Rik
Nek8
Rpl23a
Rab34
4933404M19Rik
Nek8
Rpl23a
4933404M19Rik
Supt6h
Sdf2
2610507B11Rik
Supt6h
Sdf2
2610507B11Rik
Supt6h
2610507B11Rik
Supt6h
2610507B11Rik
AK161406
Spag5
Nlk
Nlk
Nlk
Nlk
Ksr1
Ksr1
Ksr1
NF1
Nf1
NF1
Nf1
mmu-mir-193
mmu-mir-193
mmu-mir-193
mmu-mir-193
mmu-mir-365-2
Suz12
Suz12
Crlf3
Cytor4
Atad5
Atad5
1110002N22Rik
1110002N22Rik
Centa2
Centa2
Rhot1
BC055823
Rhbdl3
VRHO
Rhbdl3
VRHO
Rhbdl3
VRHO
Psmd11
Psmd11
Psmd11
Tmem98
Accn1
Accn1
Cct6b
Ccdc16
Lig3
Lig3
Lig3
Lig3
Cct6b
Ccdc16
Lig3
Lig3
Lig3
Lig3
Nle1
Unc45b
Unc45b
Unc45b
Slfn2
AK135766
AK166453
Ap2b1
Ap2b1
Ap2b1
Pex12
Pex12
Pex12
Ap2b1
Ap2b1
Ap2b1
Mmp28
Mmp28
Taf15
Taf15
Taf15
1100001G20Rik
Heatr6
Heatr6
Heatr6
Hnf1b

Hnf1b
Hnf1b
Hnf1b
Ddx52
Acaca
OTTMUSG00000000997
Acaca
Acaca
Acaca
Aatf
Aatf
Lhx1
AK018772
Aatf
Aatf
Lhx1
AK018772
Aatf
Aatf
Lhx1
AK018772
Ggnbp2
Ggnbp2
Ggnbp2
DIF-3
Myohd1
Myohd1
Myohd1
Ggnbp2
Ggnbp2
Ggnbp2
DIF-3
Pigw
Pigw
Myohd1
Myohd1
Myohd1
RP23-391K14.2-001
Ggnbp2
Ggnbp2
Ggnbp2
DIF-3
Pigw
Pigw
Myohd1
Myohd1
Myohd1
RP23-391K14.2-001
Ppm1d
Ppm1d
Bcas3
Bcas3
Bcas3
Bcas3
Bcas3
Bcas3
Bcas3
Bcas3
AK085522
AK011568
Tbx2
AK085522
AK011568
Tbx2
AK085522
AK011568
Tbx2
AK085522
AK011568
Tbx2
Brip1
4632419I22Rik
4632419I22Rik
4632419I22Rik
Ints2
Ints2
Med13
Med13
0610013E23Rik
0610013E23Rik
RP23-467J12.2-001
Rps6kb1
Rps6kb1
AK165329
Tubd1
Tubd1
Tubd1
RP23-467J12.2-001
Rps6kb1
Rps6kb1
AK165329
Tubd1
Tubd1
Tubd1
Tmem49
Tmem49
ni-2
Ptrh2
Cltc
Cltc
Prr11
1110001A07Rik
1110001A07Rik
1110001A07Rik
Trim37
mmu-mir-301a
Prr11
1110001A07Rik
1110001A07Rik
1110001A07Rik
Trim37
mmu-mir-301a
Prr11
AK040752
Trim37
Ppm1e
Rad51c
Tex14
Sep-04
Sep-04
Mtmr4
Mtmr4
Hsf5
Rnf43
Rnf43
Rnf43
AK043747
mKIAA0612
Bzrap1
Bzrap1
mKIAA0612
Olfr462
Olfr463
Dynll2
Dynll2
Sfrs1
AK040276
Vezf1
AK040276
Vezf1
AK040276
Vezf1
Cuedc1
Cuedc1
Cuedc1
Cuedc1
Cuedc1
Cuedc1
Cuedc1

Cuedc1
Mrps23
Mrps23
Msi2
Msi2
Msi2
AK002838
Akap1
Akap1
Akap1
Akap1
Akap1
Akap1
Dgke
AK044435
AK039819
Gm525
Nog
Nog
Hlf
Hlf
Hlf
Hlf
Hlf
Hlf
Hlf
Hlf
Hlf
Stxbp4
Stxbp4
Stxbp4
Cox11
Stxbp4
Stxbp4
Stxbp4
Cox11
Nme2
Nme2
Nme2
Nme2
Nme1
Nme2
Nme2
Nme1
JSAP2
JSAP2
Spag9
Spag9
JSAP2
JSAP2
Spag9
Spag9
Spag9
Tob1
3300001P08Rik
3300001P08Rik
Ankrd40
Ankrd40
3300001P08Rik
3300001P08Rik
Ankrd40
Ankrd40
Cacna1g
Cacna1g
Spata20
Epn3
Mycbpap
Mycbpap
Mycbpap
Mycbpap
Lrrc59
Eme1
Mrpl27
Col1a1
Col1a1
Col1a1
Sgca
Sgca
Ppp1r9b
Ppp1r9b
Sgca
Sgca
Ppp1r9b
Ppp1r9b
Samd14
Itga3
Itga3
Itga3
Dlx3
AK139229
Tac4
5730593F17Rik
Slc35b1
Phb
Phb
AK027957
Zfp652
Zfp652
Phospho1
Gngt2
Gngt2
Zfp652
Phospho1
Gngt2
Gngt2
B4galnt2
B4galnt2
Igf2bp1
Gip
Igf2bp1
Gip
Igf2bp1
Gip
Igf2bp1
Gip
Igf2bp1
Gip
Igf2bp1
Gip
Snf8
Ube2z
Ube2z
Atp5g1
Atp5g1
Atp5g1
Ube2z
Atp5g1
Atp5g1
Atp5g1
AK078566
mmu-mir-196a-1
AK078566
mmu-mir-196a-1
AK078566
Hoxb9
Hoxb9
mmu-mir-196a-1
Hoxb8
Hoxb8
Hoxb7
Hoxb6
Hoxb8
Hoxb8
Hoxb7
Hoxb6
Hoxb5
Hoxb8
Hoxb8
Hoxb7

Hoxb6
Hoxb5
AK002860
Hoxb6
Hoxb5
Hoxb4
mmu-mir-10a
AK002860
Hoxb6
Hoxb5
Hoxb4
Hoxb3
mmu-mir-10a
AK002860
Hoxb4
Hoxb3
mmu-mir-10a
AK002860
Hoxb4
Hoxb3
mmu-mir-10a
AK002860
Hoxb4
Hoxb3
AK002860
Hoxb4
Hoxb3
Hoxb3
Hoxb3
Hoxb2
Hoxb2
Hoxb1
Hoxb2
Hoxb1
Hoxb2
Hoxb1
Hoxb1
Hoxb1
Skap1
Skap1
Skap1
Snx11
Cbx1
Cbx1
Snx11
Cbx1
Cbx1
Nfe2l1
Nfe2l1
Nfe2l1
Nfe2l1
Nfe2l1
AK132462
Nfe2l1
Nfe2l1
Nfe2l1
Nfe2l1
Nfe2l1
AK132462
Cdk5rap3
Cdk5rap3
AK188991
Atad4
Cdk5rap3
Cdk5rap3
AK188991
Atad4
AK086164
NR_003293
Cdk5rap3
Cdk5rap3
AK188991
Atad4
AK086164
NR_003293
Sp2
Sp6
Sp6
Sp6
AM295304
Scrn2
Sp6
Sp6
AM295304
Scrn2
Mrpl10
AM295304
Scrn2
Mrpl10
AM295304
Lrrc46
Mrpl10
Osbpl7
Osbpl7
Tbx21
Tbx21
Tbkbp1
Tbkbp1
Kpnb1
Kpnb1
Kpnb1
Npepps
Npepps
Npepps
Mrpl45
Mrpl45
DQ716032
4933428G20Rik
4933428G20Rik
P140
AK155239
Mllt6
AK155239
Mllt6
AK155239
Mllt6
Mel13
Mel13
Pcgf2
Pcgf2
Pcgf2
Psmb3
Psmb3
Pcgf2
Pcgf2
Pcgf2
Psmb3
Psmb3
Pcgf2
Pcgf2
Pcgf2
Psmb3
Psmb3
Pcgf2
Pcgf2
Pcgf2
Psmb3
Psmb3
Pcgf2
Pcgf2
Pcgf2
Psmb3
Psmb3
Pcgf2
Pcgf2
Pcgf2
Psmb3
Psmb3
Pcgf2

Pcgf2
Pcgf2
Psmb3
Psmb3
Rpl23
Rpl23
Lasp1
Rpl23
Rpl23
Lasp1
Rpl23
Rpl23
Lasp1
B230217C12Rik
B230217C12Rik
B230217C12Rik
Fbxo47
Plxdc1
Arl5c
Cacnb1
Cacnb1
Cacnb1
Rpl19
Rpl19
Cacnb1
Cacnb1
Cacnb1
Rpl19
Rpl19
Cacnb1
Cacnb1
Cacnb1
Rpl19
Rpl19
Stac2
Stac2
Fbxl20
Fbxl20
Fbxl20
Med1
Med1
Med1
Crkrs
Crkrs
mKIAA0904
mKIAA0904
mKIAA0904
AK047916
Neurod2
Ppp1r1b
Ppp1r1b
Ppp1r1b
Stard3
Perld1
Erbb2
Perld1
Erbb2
Perld1
Erbb2
1810046J19Rik
Ormdl3
Thrap4
Med24
Med24
Thrap4
Thra
Thra
Thra
Thra
Nr1d1
4121402D02Rik
4121402D02Rik
4121402D02Rik
4121402D02Rik
Nr1d1
4121402D02Rik
4121402D02Rik
4121402D02Rik
4121402D02Rik
Wipf2
Wipf2
Rara
Rara
Rara
Rara
Rara
Gjc1
Top2a
Top2a
Top2a
Top2a
Igfbp4
Top2a
Igfbp4
Top2a
Igfbp4
Smarce1
Smarce1
Krt28
Krt10
Eif1
Gast
Jup
Fkbp10
Jup
Fkbp10
Jup
Fkbp10
Jup
1110036O03Rik
Fkbp10
Cnp
Cnp
Cnp
AK053109
Dhx58
D11lgp2
Gcn5l2
Gcn5l2
Gcn5l2
Rab5c
Rab5c
Stat5b
Stat5a
Stat5a
Stat5A
Stat3
Stat3
Stat3
Ptrf
Ptrf
Ptrf
Hsd17b1
Coasy
Coasy
Mlx
Mlx
1300010M03Rik
Tubg1
tubg1
Plekhh3
Plekhh3
Plekhh3
Cntnap1
Plekhh3
Plekhh3
Plekhh3
Ccr10
Cntnap1

Plekhh3
Plekhh3
Plekhh3
Ccr10
Cntnap1
Plekhh3
Plekhh3
Plekhh3
Ccr10
Cntnap1
Ezh1
Ezh1
Ramp2
Vps25
Vps25
Vps25
Vps25
Wnk4
PRKWNK4
G6pc
1700113I22Rik
Rpl27
Ifi35
Vat1
Vat1
Rnd2
Rnd2
Vat1
Vat1
Brca1
Brca1
Nbr1
Nbr1
Nbr1
Brca1
Brca1
Nbr1
Nbr1
Nbr1
Nbr1
Nbr1
Nbr1
Tmem106a
Tmem106a
BC048585
Rdm1
Rdm1
Rdm1
Arl4d
Arl4d
Etv4
Etv4
Etv4
Etv4
Etv4
Etv4
Etv4
Etv4
Etv4
Etv4
Etv4
Etv4
Etv4
Etv4
Etv4
Etv4
Etv4
Etv4
Etv4
Etv4
Sost
Dusp3
1700006E09Rik
Mpp2
Mpp2
Hdac5
BC030867
Atxn7l3
Atxn7l3
Atxn7l3
Ubtf
Ubtf
Ubtf
Ubtf
Ubtf
Ubtf
Slc4a1
Rundc3a
Rundc3a
Rundc3a
Rundc3a
Rundc3a
Slc25a39
Slc25a39
Slc25a39
Slc25a39
Grn
AK144330
AK144330
Gja7
Gja7
Gja7
Higd1b
Eftud2
mKIAA0031
mKIAA0031
Ccdc103
4933439F11Rik
Dcakd
Dcakd
Dcakd
Nmt1
Plcd3
Acbd4
Acbd4
Acbd4
Acbd4
Acbd4
Acbd4
Hexim1
Plcd3
Hexim1
Hexim2
Hexim2
AK194920
Hexim2
Plcd3
Hexim1
Hexim2
Hexim2
AK194920
Hexim2
Hexim2
Hexim2
AK194920
Hexim2
Fmnl1
1700023F06Rik
4933400C05Rik
Map3k14
Map3k14
Map3k14
Map3k14
Arhgap27
5730442P18Rik
Plekhm1
Wnt9b
Wnt3
Wnt3

Wnt3
Wnt3
Nsf
AK160502
Nsf
Crhr1
Mapt
Mapt
1700081L11Rik
1700081L11Rik
1700081L11Rik
1700081L11Rik
1700081L11Rik
1700081L11Rik
Cdc27
Cdc27
Myl4
Tlk2
1700052K11Rik
Tlk2
Tlk2
Mrc2
Mrc2
Tanc2
Tanc2
Tanc2
Wdr68
Wdr68
AK019365
Map3k3
2610019A05Rik
asp4
Ftsj3
Psmc5
Ftsj3
Smarcd2
Smarcd2
Tcam1
Ftsj3
Smarcd2
Smarcd2
Tcam1
Icam2
Ern1
ire1 alpha
Ern1
Tex2
Tex2
Tex2
Tex2
Tex2
Polg2
MtPolB
MtPolB
Ddx5
Ddx5
Ddx5
Ddx5
BC058545
Ccdc45
mmu-mir-3064
Polg2
MtPolB
MtPolB
Ddx5
Ddx5
Ddx5
Ddx5
BC058545
Ccdc45
mmu-mir-3064
Smurf2
Smurf2
1810010H24Rik
1810010H24Rik
Bptf
Bptf
Pitpnc1
Pitpnc1
Pitpnc1
Pitpnc1
Pitpnc1
Pitpnc1
Pitpnc1
M-rdgB beta
Psmd12
Pitpnc1
Pitpnc1
Pitpnc1
M-rdgB beta
Psmd12
AK049282
AK043804
Helz
Helz
Cacng4
AK048292
Prkca
Prkca
Prkca
BC027814
Gna13
Gna13
Slc16a6
Slc16a6
Arsg
Arsg
Slc16a6
Slc16a6
Arsg
Arsg
Abca5
Abca5
Abca5
abca5
abca5
Kcnj2
BC006965
Sox9
AK138212
AK015526
AK011682
AK031204
Slc39a11
Slc39a11
Slc39a11
Slc39a11
Slc39a11
Slc39a11
D11Wsu99e
D11Wsu99e
D11Wsu47e
D11Wsu47e
D11Wsu47e
D11Wsu99e
D11Wsu99e
D11Wsu47e
D11Wsu47e
D11Wsu47e
AK053334
Ttyh2
Dnaic2
Dnaic2
Dnaic2
1500005I02Rik
1500005I02Rik
1500005I02Rik
Gpr142
Gprc5c

Gprc5c	
Gprc5c	
Gprc5c	
Gprc5c	
Gprc5c	
Gprc5c	
Gprc5c	
Rab37	
C630004H02Rik	
Cdr2l	
Atp5h	
Kctd2	
Atp5h	
Kctd2	
Atp5h	
Kctd2	
4933422H20Rik	
Slc16a5	
Slc16a5	
Slc16a5	
Armc7	
Grb2	
Grb2	
Grb2	
2310067B10Rik	
2310067B10Rik	
2310067B10Rik	
mKIAA0195	
Caskin2	
Tsen54	
Tsen54	
Tsen54	
Llgl2	
Caskin2	
Tsen54	
Tsen54	
Tsen54	
Llgl2	
Caskin2	
Llgl2	
Myo15b	
Myo15b	
Myo15b	
Galk1	
Unk	
Galk1	
Unk	
Galk1	
Unk	
muspaox	
Acox1	
Acox1	
2310004N24Rik	
Cdk3	
Srp68	
Galr2	
Exoc7	
Exoc7	
Rnf157	
AK011693	
Rnf157	
AK011693	
Prpsap1	
Prpsap1	
AK009121	
Sphk1	
Sphk1	
Sphk1	
Sphk1	
SPHK1a	
Sphk1	
Cygb	
Prcd	
Prcd	
1810032O08Rik	
1810032O08Rik	
Cygb	
Prcd	
Prcd	
1810032O08Rik	
1810032O08Rik	
Cygb	
1810032O08Rik	
1810032O08Rik	
Cygb	
1810032O08Rik	
1810032O08Rik	
Cygb	
1810032O08Rik	
1810032O08Rik	
mKIAA0585	
Jmjd6	
Ptdsr	
Sfrs2	
Sfrs2	
Mfsd11	
AK007898	
ET	
Mfsd11	
ET	
Mgat5b	
	Sep-09
	Sep-09
	Sep-09
	Sep-09
	Sep-09
	Sep-09
	Sep-09
	Sep-09
	Sep-09
	Sep-09
	Sep-09
	Sep-09
	Sep-09
	Sep-09
	Sep-09
	Sep-09
	Sep-09
	Sep-09
	Sep-09
	Sep-09
	Sep-09
Tmc6	
Tmc6	
Tmc6	
Tmc6	
Tmc6	
Tmc6	
Tmc8	
Tmc8	
AK020081	
Tmc6	
Tmc6	
Tmc6	
AK020081	
Clast2	
Syngr2	
Clast2	
Clast2	
Syngr2	
Clast2	
Afmid	
Afmid	
Clast2	
Tk1	
Afmid	
Afmid	
Afmid	
Clast2	

Tk1
Afmid
Afmid
Afmid
Clast2
Tk1
Afmid
Afmid
Afmid
Birc5
Socs3
Pgs1
mKIAA3028
mKIAA3028
mKIAA3028
mKIAA3028
Timp2
Ddc8
Ddc8
Timp2
D11Bwg0517e
D11Bwg0517e
Cbx8
Cbx8
Cbx8
Cbx8
Cbx8
AK149260
Tbc1d16
Card14
Nptx1
Raptor
4932417H02Rik
4932417H02Rik
4932417H02Rik
4932417H02Rik
AK077811
Baiap2
Baiap2
Baiap2
AK077811
Baiap2
Baiap2
Baiap2
Aatk
mKIAA0641
Aatk
Azi1
1810073N04Rik
1810073N04Rik
1810073N04Rik
1810073N04Rik
AK084828
Bahcc1
Bahcc1
AK084828
Bahcc1
Bahcc1
AK084828
Bahcc1
Bahcc1
Bahcc1
Bahcc1
Bahcc1
Bahcc1
Bahcc1
Bahcc1
Actg1
AK005967
Fscn2
AK217863
Actg1
AK005967
Fscn2
AK217863
2310003H01Rik
2310003H01Rik
2310003H01Rik
Nploc4
P4hb
Stra13
Lrrc45
Rac3
Stra13
Dcxr
Dcxr
Cbr2
AK195902
Gps1
Gps1
Gps1
Gps1
Gps1
Gps1
Rfng
radical-fringe
Dus1l
Dus1l
Fasn
Fasn
Ccdc57
Slc16a3
Slc16a3
Cd7
Tex19
Uts2r
1110031I02Rik
Tbcd
AK031200
AK035515
Asxl2
Ncoa1
Fkbp1b
FKBP1b-B
BC068281
BC068281
BC068281
Ubxd4
Ubxd4
Atad2b
Atad2b
mKIAA1921
BC065777
Pum2
Pum2
Pum2
Laptm4a
AK006240
Osr1
Kcns3
5830483C08Rik
Smc6
Smc6
Mycn
Mycn
Ddx1
Ddx1
4933425L03Rik
Greb1
E2f6
E2f6
Atp6v1c2
Atp6v1c2
Atp6c2
Odc1
Adam17
Rrm2
Id2
Colec11

Rps7
Rnaseh1
Rnh1
mKIAA0230
mKIAA0230
Pxdn
Cbll1
Cbll1
Prkar2b
Prkar2b
4930504H06Rik
Tspan13
Etv1
Etv1
Nrcam
Nrcam
Nrcam
Nova1
Nova1
Foxg1
AK042354
AK019435
AK043991
6030408C04Rik
Strn3
Ap4s1
Ap4s1
Heatr5a
D930036F22Rik
Arhgap5
Arhgap5
Arhgap5
Arhgap5
Arhgap5
Arhgap5
Arhgap5
Arhgap5
Arhgap5
Arhgap5
Arhgap5
Arhgap5
AK006587
Npas3
cbp146
Ppp2r3c
G5pr
1110008L16Rik
1110008L16Rik
BC064451
Titf1
Titf1
Sdccag1
Sdccag1
Sdccag1
Arf6
Psma3
AK015004
AK035085
Six1
Six4
Six4
Mnat1
Mnat1
Six4
Six4
Mnat1
Mnat1
Six4
Six4
Mnat1
Mnat1
Tmem30b
2600001A11Rik
Sgpp1
Sgpp1
Nua
Syne2
Nua
Syne2
Nua
Syne2
Nua
Syne2
Esr2
Esr2
Tex21
Tex21
Zbtb25
Zbtb25
Zbtb1
Zbtb1
Hspa2
AK133227
Plekhg3
Plekhg3
Plekhg3
Rab15
Fntb
Max
Max
Gphn
Pals1
Mpp5
Mpp5
Atp6v1d
Eif2s1
Plek2
6330442E10Rik
6330442E10Rik
Zfyve26
Zfyve26
Rad51l1
Rad51l1
Rad51l1
Synj2bp
1810020G14Rik
Synj2bp
Arip2
Synj2bp
Pcnx
Sipa1l1
Sipa1l1
Sipa1l1
Sipa1l1
Dpf3
Dpf3
Numb
Acot2
Dnalc1
C130039O16Rik
C130039O16Rik
C130039O16Rik
C130039O16Rik
C130039O16Rik
Zadh1
Zadh1
Zfp410
Zfp410
Zfp410
Zfp410
Zfp410
Apa1
9830169C18Rik
9830169C18Rik
Coq6
Coq6
Entpd5
Entpd5
2900006K08Rik

Rnf113a2
Entpd5
Entpd5
2900006K08Rik
Rnf113a2
Aldh6a1
Lin52
Abcd4
Abcd4
Abcd4
Abcd4
Abcd4
Abcd4
7420416P09Rik
Npc2
Ltbp2
Rps6kl1
Rps6kl1
Nek9
Fos
Jundm2
Jundm2
Tgfb3
1700019E19Rik
6430527G18Rik
6430527G18Rik
mKIAA1737
2310044G17Rik
2310044G17Rik
Tmed8
AI413782
AI413782
AI413782
Ahsa1
Nrxn3
Nrxn3
Nrxn3
Nrxn3
Nrxn3
Nrxn3
Nrxn3
Nrxn3
Nrxn3
Nrxn3
Nrxn3
Nrxn3
Nrxn3
Nrxn3
Nrxn3
Gtf2a1
Gtf2a1
AK053772
AK131831
Calm1
Calm1
Rps6ka5
Rps6ka5
Rps6ka5
Rps6ka5
Tc2n
Tc2n
Tc2n
Itpk1
Moap1
Moap1
5730410I19Rik
5730410I19Rik
Moap1
Moap1
5730410I19Rik
5730410I19Rik
Btbd7
Btbd7
Btbd7
Asb2
Otub2
8430415E04Rik
Clmn
mKIAA1188
Clmn
mKIAA1188
Bdkrb2
Bdkrb2
Bdkrb1
Papola
Papola
Papola
Papola
Papola
Papola
Papola
Papola
Papola
Papola
Bcl11b
Setd3
Setd3
Ccnk
Ppp2r5c
mKIAA0044
Rage
MOK
AK015341
AK013356
Rage
MOK
AK013356
Rcor1
Amn
Amn
AK039023
1200009I06Rik
1200009I06Rik
1200009I06Rik
Klc1
Klc1
Klc1
Klc1
Kif26a
BC115444
AW555464
AW555464
AW555464
Crip1
4930427A07Rik
Wdr60
Wdr60
Wdr60
D430033N04Rik
D12Ertd551e
D12Ertd551e
D12Ertd551e
Wdr60
Wdr60
Wdr60
D430033N04Rik
D12Ertd551e
D12Ertd551e
D12Ertd551e
Rapgef5
Dnahc11
Sp8
Sp8
Sp8
Sp8
Sp8
Sp8
Itgb8
Itgb8

Itgb8
Gdi2
AK164218
Gdi2
AK164218
BC016423
mKIAA2006
Wdr37
Wdr37
Wdr37
Wdr37
AK016837
Idi1
Zmynd11
Zmynd11
Ryr2
Gli3
Gli3
Gli3
Gli3
Gli3
Gli3
AK045681
AK078363
Inhba
Cdc2l5
Cdc2l5
Cdc2l5
Zscan12
Zscan12
Zscan12
DQ686689
DQ702757
AK132930
Hist1h2ai
Hist1h3h
Hist1h2bm
Hist1h4k
Hist1h2bn
mmu-mir-1983
Zfp184
Prss16
Prss16
Prss16
Hist1h2bk
Hist1h2bj
Hist1h2ah
Hist1h4i
Hist1h2ag
Hist1h2bj
Hist1h2ah
Hist1h4i
Hist1h2ag
Zfp322a
Zfp322a
Zfp322a
Abt1
Abt1
Abt1
AK040430
AK040430
AK040430
AK040430
Btn1a1
Btn1a1
Btn1a1
Btn1a1
Hist1h2bh
Hist1h4f
Hist1h1d
Hist1h2bg
Hist1h2ae
Hist1h2bf
Hist1h2be
Hist1h1c
Hist1h2bb
Hist1h2ab
Hist1h3b
Hist1h4b
Hist1h3c
AK016178
Hist1h4a
Hist1h3a
Hist1h1a
Scgn
Scgn
Them2
AK140178
Ttrap
Ttrap
D130043K22Rik
Sox4
Sox4
Sox4
Cdkal1
Cdkal1
Cdkal1
Exoc2
Exoc2
EG432743
BC070417
AK009887
Prpf4b
Prpf4b
Prpf4b
Prpf4b
Prpf4b
Prpf4b
Prpf4b
Prpf4b
Nrn1
Nrn1
Tcfap2a
Tcfap2a
Tcfap2a
Tcfap2a
Tcfap2a
Tcfap2a
Tcfap2a
AK017409
Tcfap2a
Tcfap2a
Tcfap2a
Tcfap2a
Tcfap2a
Tcfap2a
Tcfap2a
AK017409
Gcnt2
Gcnt2
Gcnt2
Gcnt2
Nedd9
Nedd9
AK132865
Nedd9
Nedd9
AK132865
Sirt5
Sirt5
Ccdc90a
Ccdc90a
Atxn1
Gm1574
Nup153
Kif13a
Dek
Rnf144b
AK034282

AK034282
AK039372
Id4
AK034282
AK039372
Id4
Barx1
Cenpp
Cenpp
Nol8
Nol8
Shc3
Gadd45g
Gadd45g
Auh
Sptlc1
AK006343
AK030238
BC100495
DQ711560
DQ689104
DQ558020
DQ712497
DQ549198
DQ703302
DQ699684
DQ553546
Uimc1
Rxrip110
Zfp346
Zfp346
Zfp346
Lman2
Lman2
Rgs14
Rgs14
Pdlim7
Pdlim7
Pdlim7
Pdlim7
Neurog1
Smad5
Smad5
Smad5
Smad5
Trpc7
5133401N09Rik
Gkap1
Gkap1
AK015814
Zcchc6
Zcchc6
Zcchc6
Gas1
2010111I01Rik
2010111I01Rik
apo
apo
apo
2010111I01Rik
2010111I01Rik
apo
apo
apo
Ptch1
PTCH
PTCH
Ptch1
PTCH
PTCH
2410141K09Rik
Zfp817
Zfp87
Zfp817
Zfp87
Zfp817
Zfp87
Adcy2
Arts1
Arts1
AK018847
Ell2
mKIAA1376
Arrdc3
mKIAA1376
Gpr98
Gpr98
Gpr98
Gpr98
AK144545
Cetn3
AK053270
Tmem161b
Vcan
Cspg2
Vcan
Vcan
Cspg2
AK040967
AK087351
Vcan
Cspg2
Vcan
Cspg2
AK040967
AK087351
AK043659
AK047727
6430502M16Rik
6430502M16Rik
Zfyve16
Papd4
Papd4
Lhfpl2
F2rl1
Iqgap2
Sv2c
AK157788
Rgnef
mKIAA1998
Utp15
Ankra2
Ankra2
Ankra2
Ankra2
Ankra2
Foxd1
Ptcd2
Mrps27
Mtap1b
Mtap1b
AK135852
Gtf2h2
Gtf2h2
Gtf2h2
Gtf2h2
Gtf2h2
Erbb2ip
Erbb2ip
2410002O22Rik
2410002O22Rik
Trim23
Trim23
2410002O22Rik
2410002O22Rik
Trim23
Trim23
Sdccag10
3110031B13Rik

Kif2a
Kif2a
AK043130
AK136919
Zswim6
2810008M24Rik
2810008M24Rik
Depdc1b
Depdc1b
Pde4d
Pde4d
Pde4d
Pde4d
GPBP
Gpbp1
Mier3
Mier3
Mier3
Mier3
Map3k1
Fst
Fst
Itga2
Isl1
isl1-beta
Parp8
Parp8
Parp8
Parp8
Dnase1l3
Abhd6
Abhd6
4930452B06Rik
4930452B06Rik
4930452B06Rik
4930452B06Rik
AK015458
AK132658
AK006409
Slc4a7
Slc4a7
Slc4a7
Slc4a7
Lrrc3b
Lrrc3b
AK080648
Top2b
Top2b
Rpl15
Nkiras1
Ube2e1
Ube2e2
2700060E02Rik
Ecd
AA536717
AA536717
AA536717
Anxa7
Anxa7
Anxa7
Anxa7
AK018074
2310021P13Rik
2310021P13Rik
2310021P13Rik
Vdac2
AK079754
Zfp503
Kcnma1
Kcnma1
Slo
Kcnma1
Dlg5
AK086741
AK047978
Zmiz1
Zmiz1
AK086741
AK047978
Zmiz1
Zmiz1
AK086741
AK047978
Zmiz1
Zmiz1
Zmiz1
Zmiz1
Zmiz1
AK076950
Arf4
E430028B21Rik
AK044563
Wnt5a
Wnt5a
Prkcd
Prkcd
Prkcd
Prkcd
Prkcd
Sh3bp5
Sh3bp5
Sh3bp5
Sh3bp5
Mettl6
Mettl6
Eaf1
Timm23
Parg
Parg
Parg
Parg
Ogdhl
Prrxl1
AK041587
Anxa8
Wapal
Gnpnat1
Bmp4
Cnih
Samd4
Samd4
C130032J12Rik
C130032J12Rik
Peli2
Otx2
Otx2
Otx2
AK042665
Otx2
Otx2
Otx2
AK042665
AK042665
Ttc5
Osgep
Apex1
Mett11d1
Mett11d1
Slc39a2
Slc39a2
Zfp219
Zfp219
Zfp219
Zfp219
G630016D24Rik
Zfp219
Zfp219
Zfp219
Zfp219

G630016D24Rik
Supt16h
AK146690
Chd8
Supt16h
AK146690
Chd8
Rab2b
Rab2b
Rab2B
mKIAA0737
Tox4
Tox4
Rab2b
Rab2b
Rab2B
mKIAA0737
Tox4
Tox4
Rab2b
Rab2b
Rab2B
mKIAA0737
Tox4
Tox4
Rab2b
Rab2b
Rab2B
mKIAA0737
Tox4
Tox4
Mettl3
Mettl3
Sall2
Sall2
Sall2
Sall2
Slc7a7
Slc7a7
Mrpl52
Mrpl52
Mrpl52
Mrpl52
Lrp10
Rem2
D14Ertd500e
Jub
Jub
Jub
Jub
Cdh24
Acin1
Acin1
Acin1
Cdh24
Acin1
Acin1
Acin1
Acin1
Slc7a8
Lat2
Homez
Homez
Bcl2l2
Homez
Bcl2l2
Bcl2l2
Pabpn1
Pabpn1
Pabpn1
Slc22a17
mboct
Slc22a17
Il25
Slc22a17
mboct
Slc22a17
Efs
Il25
Cmtm5
Cmtm5
Ngdn
Thtpa
Zfhx2
Zfhx2
Thtpa
Thtpa
Zfhx2
Thtpa
Ap1g2
G2ad
Ap1g2
G2ad
Ap1g2
G2ad
Ap1g2
G2ad
Jph4
Jph4
Jph4
Jph4
Ap1g2
G2ad
Ap1g2
G2ad
Jph4
Jph4
Jph4
Jph4
Ap1g2
G2ad
Ap1g2
G2ad
Jph4
Jph4
Jph4
Jph4
1500005A01Rik
1500005A01Rik
Psme2
Psme2
Rnf31
Rnf31
Isgf3g
Isgf3g
Isgf3g
1810034K20Rik
Nedd8
Gmpr2
Gmpr2
Nedd8
Tinf2
Pspc1
Pspc1
Gjb2
Xpo4
mKIAA1721
Xpo4
mKIAA1721
Lats2
F630043A04Rik
Mrp63
Fgf9
Fgf9
Setdb2
Setdb2
Cab39l
Cab39l
Cab39l

Setdb2
Setdb2
Cab39l
Cab39l
Cab39l
Setdb2
Setdb2
Cab39l
Cab39l
Cab39l
Cdadc1
Cdadc1
Cdadc1
Tmem46
Tmem46
mFLJ00298
Spata13
Spata13
mFLJ00298
Spata13
Spata13
Tnfrsf19
Tnfrsf19
Tnfrsf19
Tnfrsf19
Tnfrsf19
AF380425
AK134888
AK080165
AK143097
AK033134
AK029956
mmu-mir-16-1
mmu-mir-15a
AK134888
AK080165
AK139063
AK134888
AK080165
AK139063
AK134888
AK080165
AK139063
Ints6
Ints6
Ints6
Ints6
Ints6
AK016511
Ints6
Ints6
Ints6
Ints6
Ints6
AK016511
Ctsb
Fdft1
Fdft1
Neil2
Neil2
AK044422
mmu-mir-124-1
mmu-mir-3078
AK044422
mmu-mir-124-1
mmu-mir-3078
Extl3
Zfp395
Zfp395
Zfp395
Zfp395
Pbk
Ebf2
Ebf2
Nefl
Nefm
AK162870
Rhobtb2
Rhobtb2
Rhobtb2
Rhobtb2
Rhobtb2
Rhobtb2
1700081D17Rik
1700081D17Rik
Egr3
Egr3
Egr3
Egr3
Egr3
Egr3
Egr3
Egr3
Bin3
Rai16
Gfra2
Klf5
Klf5
Gpc6
Gpc6
EG328479
Zic5
Zic2
Fbxo4
AW549877
AW549877
AK041537
Card6
Rpl37
Dab2
Dab2
Dab2
Nipbl
Nipbl
Nipbl
1110020G09Rik
1110020G09Rik
1110020G09Rik
1110020G09Rik
1110020G09Rik
1110020G09Rik
Npr3
Npr3
Npr3
BC028532
Npr3
Npr3
Npr3
BC028532
Zfr
Golph3
Golph3
Pdzd2
Pdzd2
Pdzd2
Pdzd2
Pdzd2
6030458C11Rik
6030458C11Rik
Rnasen
Rnasen
Rnasen
AK048368
Cdh10
Cdh10
Cdh10
Myo10
Myo10
myo 10

Myo10
Trio
Trio
Mar-06
Sema5a
Sema5a
Sema5a
Sema5a
Sema5a
Sema5a
Mtdh
Mtdh
Mtdh
Hrsp12
Pop1
Osr2
Osr2
Osr2
Osr2
Ywhaz
Ywhaz
Ywhaz
Grhl2
Ubr5
Azin1
Azin1
Fzd6
Fzd6
Rims2
Oxr1
Oxr1
Rspo2
Rspo2
Rspo2
Nudcd1
Nudcd1
Nudcd1
Eny2
Eny2
Csmd3
Csmd3
Eif3h
D530033C11Rik
Ext1
Sntb1
Has2
NR_002874
Zhx2
Derl1
Wdr67
Wdr67
Wdr67
Mtss1
mKIAA0429
Trib1
Trib1
Myc
Myc
Myc
Myc
Myc
Myc
Ddef1
Adcy8
Adcy8
Lrrc6
St3gal1
AK169958
1810044A24Rik
1810044A24Rik
1810044A24Rik
1810044A24Rik
Arc
Exosc4
Gpaa1
Gpaa1
Cyc1
Cyc1
Cyhr1
Chrp
Chrp
Cyhr1
Kifc2
Kifc2
Ppp1r16a
Ppp1r16a
Commd5
Rpl8
Rbm9
Myh9
Myh9
Myh9
Ean57
1700061J05Rik
Tst
Mpst
Mpst
Ean57
1700061J05Rik
Tst
Mpst
Mpst
Kctd17
C1qtnf6
C1qtnf6
Sstr3
Mfng
Card10
Card10
Gga1
AK131739
Lgals1
Nol12
Triobp
Triobp
Micall1
Micall1
Micall1
Slc16a8
Baiap2l2
Baiap2l2
Pla2g6
Pla2g6
Pla2g6
Maff
Kcnj4
Kdelr3
4933432B09Rik
4933432B09Rik
Tomm22
Cbx7
Cbx7
Tnrc6b
Tnrc6b
Tnrc6b
Tnrc6b
Tnrc6b
Tnrc6b
Mkl1
Mkl1
Mkl1
St13
St13
Xpnpep3
Xpnpep3
Ep300
Ep300
Ep300
Ep300

Rangap1
Rangap1
mKIAA1835
Zc3h7b
Zc3h7b
Rangap1
Rangap1
mKIAA1835
Zc3h7b
Zc3h7b
Tob2
AK044623
Aco2
Tob2
AK044623
Aco2
Tob2
AK044623
Aco2
D15Wsu75e
Xrcc6
Xrcc6
Tcf20
Poldip3
Poldip3
Phf21b
Phf21b
Celsr1
Celsr1
Cerk
Tubgcp6
Hdac10
Mapk12
Mapk12
Mapk12
Mapk12
Plxnb2
1700027J05Rik
1700027J05Rik
Saps2
Saps2
Saps2
1700027J05Rik
Saps2
Saps2
Saps2
1700027J05Rik
Saps2
Saps2
Saps2
Mapk8ip2
Slc2a13
Lrrk2
Prickle1
Prickle1
Prickle1
Prickle1
Nell2
Nell2
Tmem16f
AK163711
1700124K17Rik
Slc38a1
Slc38a1
Slc38a1
Slc38a1
Slc38a1
Slc38a1
Slc38a2
mKIAA1382
BC038822
BC038822
Rapgef3
4930570C03Rik
4930570C03Rik
Col2a1
Senp1
Senp1
Senp1
Pfkm
Pfkm
Ccnt1
Ccnt1
AK140384
4930415O20Rik
Cacnb3
Cacnb3
Cacnb3
Cacnb3
Cacnb3
Cacnb3
Ddx23
Ddx23
Ddx23
Rnd1
Arf3
5830427D03Rik
Ddn
AK157795
Ddn
AK157795
AK140390
BC012717
Rhebl1
Dhh
Lmbr1l
Tuba1b
Tuba1b
Tuba1a
Tuba1b
Tuba1a
Tuba1b
Tuba1a
Tuba1a
AK016307
Tuba1c
Prph
Prph
Troap
Troap
Troap
Troap
Troap
Troap
C1ql4
2810451A06Rik
C1ql4
2810451A06Rik
Spats2
Spats2
Spats2
Kcnh3
Kcnh3
Mcrs1
Mcrs1
MSP58
Lima1
Gm920
Larp4
D330037H05Rik
Larp4
Larp4
Gm920
Larp4
D330037H05Rik
Larp4
Larp4
AI317237
AI317237

AI317237
Nramp2
Tcfcp2
Tcfcp2
Tcfcp2
Tcfcp2
Tcfcp2
Tcfcp2
BC004728
BC004728
Scn8a
Scn8a
Scn8a
Scn8a
Scn8a
Scn8a
Scn8a
Scn8a
Scn8a
Scn8a
Scn8a
Scn8a
Scn8a
Scn8a
Ankrd33
Krt18
Itgb7
Rarg
Rarg
Rarg
Rarg
Espl1
Aaas
Aaas
Aaas
Aaas
Sp7
Sp7
Sp7
Sp7
Sp1
Sp1
hnRNP X
Pcbp2
Pcbp2
Pcbp2
hnRNP X
Pcbp2
Pcbp2
hnRNP X
Atf7
Atf7
Atf7
Atf7
Atf7
Atf7
Atf7
Atf7
Atf7
Atf7
Atf7
Atf7
Atp5g2
Atp5g2
AK035706
Hoxc11
Hoxc10
mmu-mir-196a-2
AK035706
Hoxc10
Hoxc9
mmu-mir-196a-2
Hoxc9
Hoxc8
Hoxc6
Hoxc5
Hoxc4
Hoxc4
mmu-mir-615
Cbx5
Cbx5
Cbx5
AK084270
Hnrnpa1
Nfe2
AK207593
Gpr84
Zfp385
Zfp385
Crebbp
NR_003628
Crebbp
NR_003628
Glis2
Magmas
Coro7
Vasn
5730403B10Rik
5730403B10Rik
AK153988
Mgrn1
mKIAA0544
Mgrn1
3930401K13Rik
3930401K13Rik
3930401K13Rik
Ubn1
Ubn1
Ubn1
3930401K13Rik
3930401K13Rik
3930401K13Rik
Ubn1
Ubn1
Ubn1
Grin2a
AK086290
2700045P11Rik
AK049860
BC030884
Ntan1
2410018G20Rik
2410018G20Rik
Igll1
AK137552
Vpreb1
mTOP3beta
Top3b
Top3b
Top3b
Top3b
mTOP3beta
mTOP3beta
Igll1
AK137552
Vpreb1
mTOP3beta
Top3b
Top3b
Top3b
Top3b
mTOP3beta
mTOP3beta
Ppm1f
Ppm1f
Ube2l3
ubcM4
Gm603
Pi4ka

Pik4ca
Snap29
4930451C15Rik
4930451C15Rik
P2rxl1
P2rxl1
Gnb1l
Wdvcf
Wdvcf
Gnb1l
Tbx1
Tbx1
Mrpl40
Hira
Hira
Parl
Parl
Ap2m1
Abcf3
Abcf3
Ap2m1
Abcf3
Abcf3
Abcf3
Abcf3
Alg3
Alg3
Ece2
Camk2n2
Ece2
Ece2
Ece2
Ece2
Clcn2
Polr2h
Chrd
Chrd
Chrd
Clcn2
Polr2h
Chrd
Chrd
Chrd
Clcn2
Polr2h
Chrd
Chrd
Chrd
Clcn2
Polr2h
Chrd
Chrd
Chrd
AK043964
AK043964
2510009E07Rik
2510009E07Rik
Sfrs10
Etv5
Eif4a2
Rfc4
Rfc4
Rfc4
Adipoq
B630019A10Rik
St6gal1
Bcl6
Il1rap
Il1rap
Il1rap
Il1rap
Fgf12
Fgf12
Hes1
Centb2
Mfi2
Fbxo45
Wdr53
Rnf168
mmu-mir-1946a
Pcyt1a
Pcyt1a
Tfrc
Tfrc
Tfrc
Tnk2
Tnk2
Tnk2
Tnk2
Iqcg
Rpl35a
Rpl35a
Rpl35a
Lmln
Osbpl11
Osbpl11
AK160141
Zfp148
Zfp148
AK160141
Zfp148
Zfp148
Itgb5
Kalrn
Sema5b
Dirc2
Hspbap1
Hspbap1
Dirc2
Hspbap1
Hspbap1
Dirc2
Hspbap1
Hspbap1
Eaf2
Eaf2
Traits
Iqcb1
Iqcb1
4932425I24Rik
4932425I24Rik
Cox17
Popdc2
Pla1a
Adprh
AK086460
Cd80
Cd80
4930455C21Rik
Lsamp
Lsamp
Lsamp
Atp6v1a
Atp6v1a
Nat13
Gm608
Sidt1
AK047199
Ccdc52
Ccdc52
Slc35a5
Atg3
Atg3
Phldb2
Phldb2
Phldb2
Cd47
Cd47
Bbx

Bbx
Bbx
Bbx
Bbx
Hbp2
Bbx
Bbx
Bbx
Bbx
Bbx
Hbp2
AK144852
Alcam
Pcnp
Pcnp
Rg9mtd1
Senp7
Senp7
mKIAA1707
Senp7
Senp7
Senp7
Senp7
mKIAA1707
Senp7
Senp7
Senp7
Gpr15
Cldnd1
Cldnd1
Epha3
Epha3
Cggbp1
Cggbp1
Robo1
Robo1
Robo1
Nrip1
Nrip1
AK144299
AK012955
mmu-mir-125b-2
App
Tiam1
Tiam1
Tiam1
Tiam1
Tiam1
Tiam1
Sod1
Sod1
Sfrs15
Sfrs15
Hunk
1110004E09Rik
AK005956
Synj1
4932438H23Rik
Gart
Gart
Son
Son
Son
Donson
AK038992
Cryzl1
Cryzl1
AK045951
Rcan1
Rcan1
Runx1
Runx1
Runx1
Runx1
Runx1
Runx1
Runx1
Runx1
Dopey2
Dopey2
mKIAA0933
AK009785
Ripply3
Ripply3
Brwd1
Brwd1
Brwd1
4921526F01Rik
4921526F01Rik
Arid1b
Arid1b
Arid1b
Arid1b
Arid1b
5730437N04Rik
5730437N04Rik
5730437N04Rik
AK163710
2210038L17Rik
2210038L17Rik
Tulp4
AK163710
2210038L17Rik
2210038L17Rik
Tulp4
AK139523
AK139523
Mrpl18
AK163149
Tcp1
AK139523
AK139523
Mrpl18
AK163149
Tcp1
Wdr27
AK004434
Wdr27
1600012H06Rik
Phf10
Phf10
Wdr27
AK004434
Wdr27
Phf10
Phf10
9030025P20Rik
9030025P20Rik
9030025P20Rik
9030025P20Rik
AK145347
Dll1
Dll1
4932442K08Rik
Psmb1
Tbp
Tbp
Pdcd2
Chd1
Chd1
Rgmb
Rgmb
Lnpep
Lnpep
Lnpep
AK137492
Lnpep
Lnpep
Lnpep

AK137492
4930546H06Rik
4930546H06Rik
mmu-mir-99b
mmu-let-7e
mmu-mir-125a
V1rf5
Zfp160
Zfp160
Zfp160
Zfp51
Zfp51
Zfp51
AK008833
Zfp13
Zfp13
Zscan10
Zscan10
Zfp206
Zscan10
Zfp206
Zfp13
Zfp13
Zscan10
Zscan10
Zfp206
Zscan10
Zfp206
1520401A03Rik
Pkmyt1
Myt1
Myt1
AK037113
Abca17
Abca3
Abca3
Abca3
Abca3
AK038021
AK084778
Rnps1
Dci
AK038021
AK084778
Rnps1
Dci
AK014798
Gbl
Gbl
Caskin1
Caskin1
Slc9a3r2
Slc9a3r2
Zfp598
Syngr3
Gfer
Tbl3
Rps2
Rps2
NR_002897
Fahd1
Hagh
Cacna1h
Axin1
Axin1
Dusp1
Nkx2-5
Nkx2-5
Cuta
Cuta
DQ716012
Zbtb9
Bak1
Ggnbp1
Bak1
Itpr3
Itpr3
Itpr3
2900010M23Rik
Lemd2
Hmga1
Hmga1
Hmga1
Hmga1
D17Wsu92e
D17Wsu92e
D17Wsu92e
D17Wsu92e
F830021D11Rik
Tcp11
Tcp11
Scube3
Scube3
Scube3
Tead3
Tead3
Tead3
Fkbp5
AK146407
AK162363
4930511I11Rik
Slc26a8
Mapk14
Mapk14
Pxt1
Kctd20
Kctd20
Kctd20
Stk38
Stk38
Sfrs3
AK144811
Cdkn1a
Cdkn1a
AK156427
1300018I05Rik
AK156427
1300018I05Rik
AK156427
1300018I05Rik
Glo1
Dnahc8
Dnahc8
Dnahc8
Glp1r
Rsph1
Slc37a1
Slc37a1
Pde9a
Pde9a
Hsf2bp
Rrp1b
Rrp1b
Cyp4f16
Cyp4f16
Cyp4f16
Cyp4f16
Cyp4f13
6030422M01Rik
Zfp472
C920016K16Rik
BC057593
Zfp422-rs1
Adamts10
Adamts10
Adamts10
Myo1f
Hnrpm
Hnrpm

mKIAA4193	
	Mar-02
	Mar-02
	Mar-02
	Mar-02
	Mar-02
Rab11b	
Angptl4	
Kifc1	
Kifc1	
BC033916	
Daxx	
Daxx	
Kifc1	
Kifc1	
AK136742	
Daxx	
Daxx	
Zbtb22	
Tapbp	
Kifc1	
Kifc1	
AK136742	
Daxx	
Daxx	
Zbtb22	
Tapbp	
Rgl2	
AK136742	
Zbtb22	
AK140218	
Tapbp	
Rgl2	
AK140218	
Tapbp	
Rgl2	
Wdr46	
AK140218	
Tapbp	
Rgl2	
Wdr46	
Ring1	
H2-Ke6	
H2-Ke6	
Slc39a7	
Rxrb	
Rxrb	
Col11a2	
Col11a2	
Col11a2	
mmu-mir-219-1	
AK136114	
Brd2	
Brd2	
Brd2	
Brd2	
H2-DMa	
Brd2	
Brd2	
Brd2	
Brd2	
H2-DMa	
H2-DMa	
Psmb9	
Tap1	
Tap1	
Psmb8	
Tap2	
Psmb9	
Tap2	
Hspa1a	
Lsm2	
Lsm2	
SmX5	
SmX5	
Vars	
Vars	
Hspa1a	
Lsm2	
Lsm2	
SmX5	
SmX5	
Vars	
Vars	
D17H6S56E-5	
D17H6S56E-5	
AK088743	
Vars	
Vars	
D17H6S56E-3	
D17H6S56E-3	
Msh5	
Msh5	
Msh5	
Msh5	
Clic1	
Ddah2	
NR_001462	
Ly6g6c	
Ly6g6c	
Msh5	
Msh5	
Msh5	
Msh5	
Clic1	
Ddah2	
NR_001462	
Ly6g6c	
Ly6g6c	
Msh5	
Msh5	
Msh5	
Msh5	
Clic1	
Ddah2	
NR_001462	
Ly6g6c	
Ly6g6c	
Bat2	
Bat2	
Nfkbil1	
BC025190	
Atp6v1g2	
Bat1a	
Bat1a	
Bat1a	
Bat1a	
Bat1a	
Bat1a	
Nfkbil1	
BC025190	
Atp6v1g2	
Bat1a	
Bat1a	
Bat1a	
Bat1a	
Bat1a	
Bat1a	
Nfkbil1	
Bat1a	
Bat1a	
Bat1a	
Bat1a	
Bat1a	
Bat1a	
Nfkbil1	
Bat1a	
Bat1a	
Bat1a	

Bat1a
Bat1a
Bat1a
Tcf19
Cchcr1
Psors1c2
Dpcr1
AK087178
Vars2
Vars2
Gtf2h4
Ddr1
Nep
Ddr1
Ddr1
Ddr1
Ddr1
Abcf1
Gna-rs1
mmu-mir-877
Abcf1
Prr3
Prr3
CAT56
Gna-rs1
BC055918
mmu-mir-877
Prr3
Prr3
CAT56
BC055918
Rpp21
CAT60
Trim39
Trim39
Trim39
Trim39
Trim39
Trim26
Trim26
Trim26
Trim26
Ppp1r11
1700022C21Rik
1700022C21Rik
Mog
Gabbr1
Gabbr1
GABA-B-R
Gabbr1
Gabbr1
Gabbr1
GABA-B-R
Gabbr1
Gabbr1
Cd2ap
Aars2
Aarsl
Tcte1
Hsp90ab1
Hsp90ab1
Tmem63b
Mrpl14
Vegfa
Vegfa
Vegf
Vegfa
Vegfa
Vegf
Polh
Xpo5
Xpo5
Tjap1
Tjap1
Dlk2
Dlk2
Abcc10
Abcc10
Abcc10
Zfp318
Zfp318
AK144536
BC048355
Parc
Parc
Parc
Srf
Klhdc3
Ppp2r5d
Ppp2r5d
Pex6
Gnmt
Tnrc5
Ptcra
Rpl7l1
Rpl7l1
Ubr2
Ubr2
Ubr2
Ubr2
Trerf1
Trerf1
Trerf1
1700001C19Rik
AI661453
Ccnd3
Ccnd3
Ccnd3
Med20
Med20
Med20
Usp49
Ccnd3
Ccnd3
Ccnd3
Med20
Med20
Med20
Usp49
Ccnd3
Ccnd3
Ccnd3
Med20
Med20
Med20
Usp49
Bysl
Med20
Usp49
Usp49
AK043085
Trem3
Treml4
Trem3
Treml4
Plcl2
Kcnh8
Pcaf
Pcaf
Sgol1
Sgo1
Pot1b
Pot1b
Pot1b
BC031441
BC031441
Ebi3
Ccdc94
Shd

BC031441
BC031441
Ccdc94
Shd
Ticam1
Ticam1
Ticam1
Ticam1
M6prbp1
Uhrf1
Uhrf1
M6prbp1
Arrdc5
Uhrf1
Uhrf1
M6prbp1
Arrdc5
Uhrf1
Uhrf1
2410015M20Rik
Qil1
Rpl36
AK014981
Ranbp3
Ranbp3
Ranbp3
Khsrp
Slc25a41
Dennd1c
Dennd1c
Tubb4
AK154177
AK047366
Tnfsf9
C3
Gpr108
Trip10
Gpr108
Trip10
Vav1
Vav1
Vav1
Efna5
Vapa
Vapa
Ralbp1
Ralbp1
Ralbp1
AK079730
Ralbp1
Ralbp1
Ralbp1
AK079730
Ddx11
1110012J17Rik
Arhgap28
Tgif1
Tgif1
Tgif1
Mylc2b
Mylc2b
2900073G15Rik
Myom1
Myom1
mKIAA0650
Alk
2810410M20Rik
2810410M20Rik
2810410M20Rik
Birc6
Birc6
Strn
Prkcn
Prkcn
Prkcn
Prkcn
Prkcn
Prkcn
Prkcn
Prkcn
Qpct
Arl6ip2
Arl6ip2
Arl6ip2
Sos1
Sos1
Sos1
Sos1
Map4k3
Map4k3
Cox7a2l
Mta3
Mta3
Mta3
Mta3
Haao
Haao
AK036982
Haao
Haao
AK036982
Thada
Thada
Plekhh2
Plekhh2
AK003841
AK011222
Ppm1b
Ppm1b
Ppm1b
Ppm1b
Ppm1b
AK003841
AK011222
Ppm1b
Ppm1b
Ppm1b
Ppm1b
Ppm1b
1700106N22Rik
1700106N22Rik
AY589789
AY589791
AK053722
AY590888
Six3
BC065087
Six3
Srbd1
1700011E24Rik
Calm2
1700011E24Rik
Calm2
Fbxo11
Fbxo11
Nrxn1
Nrxn1
mKIAA0578
mKIAA0578
AK139280
2610044O15Rik
2610044O15Rik
AK143926
Cul2
Cul2
mKIAA4106
Cul2
Cul2
mKIAA4106

Bambi
Bambi
Fzd8
Rock1
Rock1
AK220484
AK220484
Abhd3
Mib1
Mib1
Rbbp8
Rbbp8
Rbbp8
Rbbp8
Lama3B
lama3
2810439F02Rik
Osbpl1a
Osbpl1a
Osbpl1a
Osbpl1a
Osbpl1a
Osbpl1a
Orp1
Zfp521
Zfp521
AK012135
Gm944
Gm944
Nol4
Nol4
Mapre2
Mapre2
Mapre2
AK088734
AK088914
Zfp191
Zfp191
2700062C07Rik
BC021395
Brunol4
Brunol4
Brunol4
Brunol4
Brunol4
Brunol4
Slc25a46
Slc25a46
Sap130
Sap130
Sap130
Sap130
Sap130
Sap130
Sap130
Sap130
Wdr33
Wdr33
wdc146
Wdr33
Wdr33
Wdr33
AK043919
Bin1
Wdr36
Wdr36
Wdr36
Wdr36
D0H4S114
D0H4S114
Epb4.1l4a
Epb4.1l4a
Epb4.1l4a
Epb4.1l4a
Brd8
Brd8
Brd8
Kif20a
BC094408
AK216043
Jmjd1b
Jmjd1b
Jmjd1b
Jmjd1b
Reep2
Reep2
Etf1
Etf1
Hspa9
AK088959
AK080350
Matr3
Matr3
mmu-mir-1949
AK088959
AK080350
Matr3
Matr3
mmu-mir-1949
2010001M09Rik
5133400G04Rik
5133400G04Rik
Pura
Pura
0610010O12Rik
0610010O12Rik
0610010O12Rik
0610010O12Rik
Hbegf
Hbegf
Slc4a9
Slc4a9
Cd14
Tmco6
Tmco6
Tmco6
Ik
Cd14
Ndufa2
Ik
Wdr55
Pcdha4
Slc25a2
Taf7
AK149307
Pcdhga1
Diap1
Diap1
Pcdh1
Pcdh1
AK050707
AK039796
0610009O20Rik
0610009O20Rik
0610009O20Rik
Lars
Lars
Rbm27
Pou4f3
Kcnn2
Kcnn2
Kcnn2
Kcnn2
Trim36
Fem1c
Fem1c
Fem1c
Fem1c
Sema6a

Sema6a
Sema6a
Sema6a
Sema6a
Sema6a
Tnfaip8
AK021048
AK043286
AK046742
Tnfaip8
Sncaip
Sncaip
Sncaip
Sncaip
Snx24
Snx24
Prdm6
Csnk1g3
Zfp608
mKIAA1281
Zfp608
Zfp608
Zfp608
mKIAA1281
Zfp608
AK155149
AK163160
Zfp608
mKIAA1281
Zfp608
AK155149
AK163160
Megf10
Megf10
Slc12a2
Rbm22
Rbm22
Camk2a
Camk2a
Csnk1a1
Csnk1a1
Csnk1a1
Csnk1a1
Csnk1a1
Csnk1a1
Afap1l1
Txnl1
Txnl1
Wdr7
Wdr7
Wdr7
Wdr7
Wdr7
BC048666
St8sia3
St8sia3
Onecut2
Onecut2
Onecut2
Nedd4l
Sec11c
Impa2
AK078979
Seh1l
Seh1l
AK016077
AK078979
Seh1l
Seh1l
AK016077
AK016077
AK158414
AK016077
AK158414
Tcf4
Tcf4
Tcf4
Tcf4
Tcf4
Tcf4
Tcf4
Tcf4
Tcf4
Tcf4
Tcf4
Tcf4
Tcf4
Tcf4
Tcf4
Tcf4
Mapk4
Mapk4
AK036329
Myo5b
Myo5b
Lipg
Lipg
Rpl17
BC031181
BC031181
Smad7
Smad7
Smad7
Ccdc5
Ccdc5
Atp5a1
Pstpip2
Pstpip2
Pstpip2
Setbp1
Nfatc1
Nfatc1
Nfatc1
Sall3
Sall3
Sall3
AK015395
Zfp516
Zfp516
Tshz1
Tshz1
Zadh2
Zfp407
B230399E16Rik
Cpt1a
Suv420h1
Suv420h1
Suv420h1
Suv420h1
Suv420h1
Chka
Chka
Chka
Chka
Chka
Chka
Chka
Chka
Chka
Aldh3b1
Unc93b1
Unc93b1
Unc93b1
Aip
Aip
Cabp4
Gpr152
Coro1b
AK184225

Ptprcap
Cabp4
Coro1b
Ptprcap
Rad9
Rad9
Clcf1
Clcf1
Pold4
Pold4
Pold4
Pold4
Ssh3
mSSH-3L
Ankrd13d
Fbxl11
Fbxl11
Rhod
Rhod
Syt12
Gm960
Spnb3
Spnb3
Rbm14
Rbm4
Rbm4b
Rbm4
Rbm4b
Rbm14
Rbm4
Rbm4b
Rbm4
Rbm4b
Rbm14
Rbm14
Npas4
Pacs1
Pacs1
Pacs1
Sf3b2
Sf3b2
Sf3b2
Gal3st3
Mus81
Mus81
Cfl1
Ehbp1l1
Ehbp1l1
Mtvr2
C184
Sssca1
Sssca1
Ltbp3
Ltbp3
Ltbp-3
Ehbp1l1
Ehbp1l1
Mtvr2
C184
Sssca1
Sssca1
Ltbp3
Ltbp3
Ltbp-3
AK019083
AK019083
AK019083
AK019083
Tigd3
Tigd3
Gja10
Dpf2
Dpf2
Dpf2
Tigd3
Tigd3
Gja10
Dpf2
Dpf2
Dpf2
Tm7sf2
Tm7sf2
Tm7sf2
1110014N23Rik
1110014N23Rik
1110014N23Rik
Zfpl1
Cdca5
Cdca5
BC049760
Ppp2r5b
Atg2a
Atg2a
mKIAA0404
AK149321
Ehd1
AK149321
Ehd1
Nrxn2
Nrxn2
Nrxn2
Nrxn2
Nrxn2
Nrxn2
Nrxn2
Nrxn2
Nrxn2
Nrxn2
Nrxn2
Nrxn2
Nrxn2
Nrxn2
Rps6ka4
Ccdc88b
Stip1
Stip1
Macrod1
Cox8a
Nat11
Nat11
Nat11
Mark2
Mark2
AI846148
AI846148
AK018996
Rtn3
AK043844
5730596K20Rik
Rtn3
AK043844
5730596K20Rik
Rtn3
5730596K20Rik
Mta2
Tut1
Tut1
Eef1g
Tut1
Eef1g
Ahnak
Ahnak
Rab3il1
Rab3il1
Rab3il1
Fads2
Fads2
Dagla
Nsddr

Syt7
0610038F07Rik
0610038F07Rik
5730453I16Rik
5730453I16Rik
5730453I16Rik
5730453I16Rik
Tmem138
Tmem138
Cybasc3
Cybasc3
Cybasc3
Cybasc3
Tmem132a
Tmem132a
Tmem109
Tmem132a
Tmem132a
Tmem109
Prpf19
Stx3
Stx3
AK044380
Zfp91-cntf
Zfp91
Lpxn
Psat1
Psat1
Psat1
Vps13a
Vps13a
Foxb2
Foxb2
Rorb
Rorb
Gda
Gda
AK019124
Klf9
mmu-mir-1192
AK019124
Klf9
mmu-mir-1192
AK019124
Klf9
mmu-mir-1192
Tjp2
Pip5k1b
2900009I07Rik
Pip5k1b
2900009I07Rik
Pip5k1b
Dmrt3
Smarca2
Smarca2
Smarca2
Rfx3
Rfx3
Rfx3
Jak2
Ermp1
Ermp1
Kiaa1815
ENSMUSG00000048936
Prkg1
Prkg1
Asah2
Asah2
Asah2
Asah2
Minpp1
Minpp1
Pten
Pten
Pten
Pten
Pcgf5
Pcgf5
Pcgf5
Hectd2
Hectd2
Hectd2
Hectd2
Hectd2
Hectd2
Tnks2
Tnks2
Tnks2
Kif11
Exoc6
Fer1l3
Fer1l3
AK153534
Cep55
Cep55
Cep55
Cyp2c69
Pdlim1
Clim1
Sorbs1
Sorbs1
Sorbs1
Tm9sf3
Tm9sf3
Arhgap19
Mms19
Mms19
Mms19
Mms19
Ubtd1
BC023055
BC023055
Pi4k2a
Pi4k2a
Avpi1
Marveld1
LOC545291
Nkx2-3
Nkx2-3
Dnmbp
Dnmbp
AK076939
Cwf19l1
Cwf19l1
Scd2
Scd2
Scd1
Scd1
Wnt8b
Sec31b
Ndufb8
Ndufb8
Hif1an
Hif1an
Pax2
Pax2
Pax2
Pax2
mKIAA4123
Btrc
Btrc
mKIAA4123
Btrc
Btrc
mKIAA4123
Btrc
Btrc
Poll
5330431N19Rik

Fbxw4
Fgf8
Fgf8
Npm3
AK040197
Ldb1
Ldb1
Ldb1
Pprc1
Pprc1
Pitx3
Gbf1
Gbf1
Gbf1
Gbf1
Pitx3
Gbf1
Gbf1
Gbf1
Gbf1
AK029443
Nfkb2
Nfkb2
Nfkb2
Nfkb2
Nfkb2
Nfkb2
Nfkb2
Fbxl15
Fbxl15
Psd
Psd
Cuedc2
AK178562
Tmem180
mmu-mir-146b
Cuedc2
AK178562
Tmem180
Cuedc2
AK178562
Tmem180
AK204799
Trim8
Arl3
Arl3
Sfxn2
Sfxn2
Pcgf6
Pcgf6
Taf5
Usmg5
Pdcd11
Sh3pxd2a
Sh3pxd2a
Slk
6330577E15Rik
D19Ertd652e
BC063749
BC063749
Mxi1
Mxi1
Mxi1
Mxi1
Mxi1
Mxi1
Mxi1
Mxi1
Smndc1
Smndc1
Dusp5
AK016185
BC040803
Shoc2
Tcf4
Tcf7l2
Tcf7l2
Tcf7l2
Tcf4
Tcf7l2
Tcf7l2
Tcf7l2
Tcf4
Tcf7l2
Tcf7l2
Tcf7l2
A630007B06Rik
A630007B06Rik
Tdrd1
Tdrd1
Tdrd1
AK045643
AI450540
AK045643
AI450540
AK045643
AI450540
Vax1
Pdzd8
2700078E11Rik
Prdx3
Grk5
Frmd4a
Frmd4a
Frmd4a
mKIAA1294
Frmd4a
Camk1d
Camk1d
Camk1d
Cdc123
Cdc123
Nudt5
Cdc123
Cdc123
Nudt5
Dhtkd1
Upf2
Dhtkd1
Upf2
Gata3
Gata3
Gata3
AK029605
AK020309
Gata3
Gata3
Gata3
AK029605
AK020309
Taf3
AK076687
Sfmbt2
Sfmbt2
Sfmbt2
Sfmbt2
Sfmbt2
AK076687
Sfmbt2
Sfmbt2
Sfmbt2
Sfmbt2
Sfmbt2
Sfmbt2
Il15ra
Il15ra
Il15ra
Il15ra
Cacnb2

Cacnb2
Plxdc2
Nebl
AK044329
AK045818
AK044329
AK045818
AK044329
Mllt10
Mllt10
AK044329
2810030E01Rik
Mllt10
Mllt10
mAF10
Mllt10
Mllt10
AK044329
2810030E01Rik
Mllt10
Mllt10
mAF10
Mllt10
Mllt10
Bmi1
Bmi1
BC061194
Arhgap21
Arhgap21
Arhgap21
4933434I06Rik
Thnsl1
Thnsl1
Gad2
Gad2
Gad2
Gad2
Gad2
Gad2
Acbd5
Acbd5
Mastl
Mastl
Yme1l1
Yme1l1
Mastl
Mastl
Yme1l1
Yme1l1
Ehmt1
Ehmt1
glp
Ehmt1
Ehmt1
glp
Wdr85
Wdr85
Pnpla7
Pnpla7
Pnpla7
Nrarp
Traf2
Traf2
Edf1
B230208H17Rik
B230208H17Rik
B230208H17Rik
4921530D09Rik
AK039020
Tmem141
Tmem141
Bmyc
Sec16a
Sec16a
Sec16a
AK075572
Notch1
Notch1
Agpat2
Agpat2
Agpat2
AK011283
AK030770
Wdr5
Vav2
BC094334
Wdr5
Vav2
BC094334
Wdr5
Vav2
BC094334
BC050122
Barhl1
Barhl1
1700101E01Rik
Ttf1
Urm1
Spna2
Wdr34
AK031561
Set
Set
Pkn3
Pkn3
Pkn3
Dolpp1
Ier5l
Ier5l
mmu-mir-3089
mmu-mir-3089
mmu-mir-3089
Tor1a
BC005624
Usp20
Usp20
Gpr107
Gpr107
Fubp3
Fubp3
Fibcd1
A130092J06Rik
A130092J06Rik
Ppapdc3
5830434P21Rik
mKIAA0515
5830434P21Rik
mKIAA0515
5830434P21Rik
mKIAA0515
mKIAA0515
C230093N12Rik
C230093N12Rik
Stxbp1
Stxbp1
Stxbp1
9130404D14Rik
Lrsam1
Lrsam1
Rpl12
Pbx3
Mapkap1
Mapkap1
Mapkap1
Gapvd1
Gapvd1
Hspa5
Hspa5
Rabepk

Rabepk	
AK019757	
Pdcl	
Pdcl	
Rc3h2	
Zbtb6	
Zbtb6	
Zbtb26	
Zbtb26	
Rabgap1	
Rabgap1	
Zbtb6	
Zbtb26	
Zbtb26	
Rabgap1	
Rabgap1	
Rabgap1	
Rabgap1	
Rabgap1	
Strbp	
Gpr21	
Strbp	
Strbp	
Strbp	
Strbp	
Crb2	
Crb2	
Lhx2	
Lhx2	
Lhx2	
Lhx2	
Psmb7	
Psmb7	
Nr5a1	
Nr5a1	
ELP	
Nr6a1	
Olfml2a	
Nr6a1	
Olfml2a	
Nr6a1	
Olfml2a	
Lrp1b	
Lrp1b	
Lrp1b	
Lrp1b	
Gtdc1	
Orc4l	
MuORC4	
Orc4l	
Mbd5	
Kif5c	
Rnd3	
Rnd3	
Rnd3	
Rnd3	
Stam2	
Stam2	
Stam2	
Prpf40a	
Prpf40a	
FBP11	
Arl6ip6	
Arl6ip6	
Nr4a2	
Nr4a2	
BC062650	
Pkp4	
Pkp4	
Tanc1	
Tanc1	
Tanc1	
Tanc1	
Baz2b	
Baz2b	
Baz2b	
Baz2b	
AK166079	
	Mar-07
	Mar-07
Tank	
Tank	
Tank	
Ifih1	
Ifih1	
Ifih1	
Gca	
Fign	
Stk39	
Stk39	
4930578N16Rik	
Phospho2	
Phospho2	
Phospho2	
Klhl23	
Mettl5	
Mettl5	
Zfp650	
Zfp650	
Zfp650	
Dync1i2	
Dncic2	
Dync1i2	
Itga6	
Itga6	
Cdca7	
Ola1	
Chrna1	
Atf2	
Atf2	
Atf2	
Atf2	
Hoxd10	
BC060302	
Hoxd9	
Hoxd8	
Hoxd3	
Hoxd3	
AK144266	
Hoxd1	
Hnrpa3	
Hnrpa3	
Agps	
Agps	
Zfp533	
Ssfa2	
Ssfa2	
Ssfa2	
Itgav	
Itgav	
Itgav	
Itgav	
Ctnnd1	
Ctnnd1	
Ctnnd1	
Ctnnd1	
Zdhhc5	
Clp1	
Ypel4	
Clp1	
mmu-mir-130a	
P2rx3	
AK019679	
Ssrp1	
Ssrp1	
Tnks1bp1	
mKIAA1741	
BC027685	
Agtrl1	

Ptprj
Nup160
Gtl-13
Nup160
Nup160
Gtl-13
Nup160
Gtl-13
Nup160
Nup160
Gtl-13
Nup160
Gtl-13
Nup160
Nup160
Gtl-13
Mtch2
Mtch2
Mtch2
Mtch2
Mtch2
Mtch2
Mtch2
Mtch2
AK020549
Slc39a13
Madd
mKIAA0358
Madd
Madd
Nr1h3
Nr1h3
Nr1h3
Acp2
1110051M20Rik
1110051M20Rik
Lrp4
Lrp4
Lrp4
Lrp4
mKIAA0097
Ckap5
mKIAA0097
Ckap5
F2
Zfp408
Arhgap1
Arhgap1
F2
Zfp408
Arhgap1
Arhgap1
D2Ertd391e
D030051N19Rik
D030051N19Rik
D030051N19Rik
D030051N19Rik
D030051N19Rik
D030051N19Rik
AK039132
Phf21a
Phf21a
mmu-mir-1955
Phf21a
Phf21a
mmu-mir-1955
Phf21a
Phf21a
mmu-mir-1955
Phf21a
Phf21a
mmu-mir-1955
Phf21a
Phf21a
Phf21a
Gyltl1b
Gyltl1b
Gyltl1b
Gyltl1b
Pex16
Pex16
1700029I15Rik
Trp53i11
C230071H18Rik
Ext2
Ext2
Ext2
AK140370
AK089118
mmu-mir-670
Lrrc4c
Lrrc4c
B230118H07Rik
B230118H07Rik
B230118H07Rik
Rag2
Traf6
Traf6
2600010E01Rik
Trim44
Pdhx
Pdhx
Apip
Caprin1
Caprin1
Caprin1
Hipk3
Prrg4
Prrg4
AK165089
Wt1
Wt1
NR_002867
Pax6
AK044354
AK080416
Pax6
AK047243
Pax6
Pax6
Dph4
AK163667
BC048594
Mpped2
BC020182
Mpped2
Bdnf
Bdnf
Bdnf
Bdnf
Bdnf
Bdnf
Bdnf
Bdnf
Agpat7
Agpat7
Aven
Scg5
Arhgap11a
Arhgap11a
AK135429
BC052040
Meis2
Mrg1
Mrg1
Meis2
meis2a
AK144367
AK144485

Meis2
Mrg1
Mrg1
Meis2
meis2a
AK012325
AK144367
AK144485
Meis2
Mrg1
Mrg1
Meis2
meis2a
AK012325
AK144367
AK144485
Bmf
Bmf
AK145129
Plcb2
Plcb2
BC026434
D2Ertd750e
Bahd1
Rad51
Rad51
Rad51
Rad51
1200015F23Rik
Gchfr
OTTMUSG00000015762
Ppp1r14d
Spint1
Spint1
Rhov
Vps18
Vps18
Vps18
Rhov
Vps18
Vps18
Vps18
AK039958
Dll4
Inoc1
Inoc1
Exdl1
Sid470
1500003O03Rik
Exdl1
Sid470
1500003O03Rik
Ndufaf1
Ndufaf1
cbp82
Itpka
Itpka
Mga
Ehd4
Tmem87a
Tmem87a
Tmem87a
mFLJ00088
Ganc
Ganc
Capn3
Ganc
Ttbk2
Ttbk2
AK015969
AK006022
Trp53bp1
Trp53bp1
Trp53bp1
53BP1
Mtap1a
Catsper2
Catsper2
Catsper2
Pdia3
Catsper2
Catsper2
Catsper2
Pdia3
Ell3
Serf2
2310003F16Rik
Ell3
Serf2
LOC574418
2310003F16Rik
Mfap1a
Wdr76
Wdr76
Mageb3
Ctdspl2
Ctdspl2
Ctdspl2
Mageb3
Ctdspl2
Ctdspl2
Ctdspl2
Eif3j
Eif3j
Shf
AK016335
Pldn
pa
Shc4
Shc4
Trpm7
ChaK
2010106G01Rik
Ap4e1
Blvra
Blvra
Blvra
Adra2b
Kcnip3
DREAM/calsenilin/KChIP3
Kcnip3
DREAM/calsenilin/KChIP3
Kcnip3
DREAM/calsenilin/KChIP3
Prom2
Nphp1
BC048556
Acoxl
Snrpb
Tmc2
Idh3b
Ebf4
O/E-4S
Ebf4
O/E-4S
Adra1d
AK019738
Prnp
Prnd
Slc23a2
Slc23a2
Slc23a2
Slc23a2
Chgb
Trmt6
Trm6
Mcm8
Pak7
Pak7

Jag1
Snx5
Snx5
8430406I07Rik
8430406I07Rik
Rbbp9
Sec23b
Sec23b
Gm561
Dtd1
Dtd1
Dtd1
Insm1
Gm114
Xrn2
Xrn2
Xrn2
Xrn2
Xrn2
Xrn2
Cst3
Snph
Snph
Psmf1
Psmf1
Psmf1
Psmf1
AK006186
Tcf15
Rbck1
Rbck1
AY512942
Rbck1
Rbck1
Trib3
AY512942
Sox12
Zcchc3
6820408C15Rik
6820408C15Rik
AK006531
Hm13
H13
H13
Mcts2
Id1
Cox4i2
Cox4i2
Cox4i2
Cox4i2
Cox4i2
Cox4i2
Bcl2l1
Bcl2l1
Bcl2l1
Bcl2l1
Bcl2l1
Tpx2
Tpx2
Bcl2l1
Bcl2l1
Bcl2l1
Bcl2l1
Bcl2l1
Tpx2
Tpx2
Fkhl18
Pdrg1
Xkr7
Tspyl3
Plagl2
Plagl2
Pofut1
Pofut1
Pofut1
AK010870
Asxl1
8430427H17Rik
8430427H17Rik
8430427H17Rik
8430427H17Rik
8430427H17Rik
Commd7
Commd7
Commd7
Dnmt3b
Commd7
Commd7
Commd7
Dnmt3b
Cdk5rap1
Cdk5rap1
Apba2bp
1700003F12Rik
Pxmp4
AK145231
Zfp341
Raly
Raly
Raly
Raly
Ggtl3
Ggtl3
Acss2
Acss2
Acss2
Acss2
AK158030
Eif6
AK158030
Eif6
Uqcc
Uqcc
Uqcc
Uqcc
mKIAA0338
mKIAA0338
Epb4.1l1
Epb4.1l1
Dlgap4
AK015092
Dlgap4
Dlgap4
Rbl1
Rbl1
Rbl1
AK012418
4922505G16Rik
Rpn2
Ghrh
Ghrh
Manbal
Blcap
2610036D13Rik
2610036D13Rik
Zhx3
Zhx3
Lpin3
Lpin3
Rp23-297j14.5
3230401D17Rik
Gdap1l1
Gdap1l1
Gdap1l1
Serinc3
Pkig
Pkig
AK043662
Wfdc2

AK015392
F730014I05Rik
BC060988
Zfp335
AK141411
Slc12a5
Cdh22
Slc13a3
Slc13a3
Slc2a10
Prkcbp1
Prkcbp1
Prkcbp1
Prkcbp1
mKIAA1125
mKIAA1125
Sulf2
Sulf2
Sulf2
Sulf2
Arfgef2
Cse1l
Stau1
AK041781
Ddx27
Ddx27
Kcnb1
Kcnb1
AY672066
AK003793
NHE8
Slc9a8
Slc9a8
Ube2v1
Ptpn1
2310033K02Rik
AK009597
AK027991
Adnp
Adnp
Adnp
Adnp
Tshz2
Tshz2
Cyp24a1
Pfdn4
Pfdn4
Dok5
Tcfap2c
Tcfap2c
Tcfap2c
Tcfap2c
AK006546
AK006104
AK029171
AK164611
AK080176
AK161459
AK170078
AK046224
Stx16
Stx16
Taf4a
AK014955
Psma7
Ss18l1
Gtpbp5
BC066135
BC066135
Ythdf1
Birc7
Arfrp1
Arfrp1
Zgpat
Zgpat
Lime1
Lime1
AK082896
Polr3k
Polr3k
AK012506
Zfhx4
Tpd52
Aadacl1
Evi1
evi-1
Evi1
Evi1
Evi1
Evi1
AK145113
Evi1
evi-1
Evi1
Evi1
Evi1
Evi1
AK145113
Evi1
evi-1
Evi1
Evi1
Evi1
Evi1
AK145113
Evi1
Mds1
Arpm1
Mynn
Phc3
Phc3
Phc3
Prkci
Skil
Skil
Usp13
Pex2
Pex2
Pex2
AK029638
AK153849
Nudt6
Nudt6
Nudt6
Spata5
Fat4
Fat4
AK078307
3110057O12Rik
3110057O12Rik
3110057O12Rik
AK012035
Pcdh10
Pcdh10
Pcdh10
Elf2
Elf2
Elf2
Elf2
ELF2
AK017883
Elf2
Elf2
Elf2
ELF2
AK042136
Setd7
AK017144
Setd7

AK017144
Setd7
AK017144
AK145396
AK145396
Foxo1
Lhfp
Dclk1
Dclk1
Dclk1
Dclk1
Nbea
Lyst2
D3Ucla1
Eif2a
Eif2a
Eif2a
Siah2
AK016388
Med12l
Med12l
Med12l
4631416L12Rik
Mme
Mme
Mme
Mme
AK144610
Schip1
Schip1
Schip1
Schip1
Schip1
Kpna4
BC062821
Ppm1l
Etfdh
Etfdh
Etfdh
Etfdh
4930579G24Rik
Tmem144
Tmem144
Pdgfc
Pdgfc
Mtap9
Mtap9
Mtap9
Mtap9
Mtap9
Mtap9
Mtap9
Mtap9
AK137033
Trim2
Trim2
Trim2
Trim2
Trim2
Trim2
Arfip1
Arfip1
Tigd4
Arfip1
Fbxw6
SH3d19
Lrba
Lba
Lrba
Lrba
Lrba
Lrba
Lrba
Lrba
Mab21l2
Lrba
Lrba
Lrba
Mab21l2
Lrba
Lrba
Lrba
Mab21l2
Kirrel
Kirrel
Kirrel
Kirrel
Kirrel
Kirrel
Kirrel
PE1
Etv3
PE1
Nes
Nes
Nes
Nes
Nes
Nes
Nes
Nes
Nes
Bcan
Bcan
Mef2d
Mef2d
AK007191
AK008852
AK141565
AK140632
mmu-mir-3093
AK141565
AK140632
mmu-mir-3093
mmu-mir-9-1
1700021C14Rik
1700021C14Rik
Cct3
Cct3
Cct3
Tmem79
Tmem79
Smg5
Smg5
Lmna
Lmna
Lmna
Arhgef2
Arhgef2
Arhgef2
Arhgef2
Arhgef2
Arhgef2
mKIAA0651
Arhgef2
Arhgef2
Arhgef2
Arhgef2
Arhgef2
Arhgef2
mKIAA0651
Arhgef2
Arhgef2
Arhgef2
Arhgef2
Arhgef2
Arhgef2
mKIAA0651
AK078352

2810403A07Rik
2810403A07Rik
2810403A07Rik
AK078352
2810403A07Rik
2810403A07Rik
2810403A07Rik
AK078352
2810403A07Rik
2810403A07Rik
2810403A07Rik
Dap3
Ash1l
Ash1l
Hcn3
AK177718
Clk2
Clk2
Scamp3
Gba
Thbs3
Mtx1
Thbs3
Muc1
Krtcap2
mmu-mir-92b
Efna3
AK140090
AK132440
Efna4
AK132440
Adam15
Adam15
Adam15
Dcst1
Zbtb7b
Zbtb7b
Zbtb7b
AK161599
AK136654
AK053703
Flad1
Flad1
Flad1
Lenep
Cks1b
Shc1
Shc1
Pygo2
Pygo2
Pygo2
Pbxip1
AK053703
Flad1
Flad1
Flad1
Lenep
Cks1b
Shc1
Shc1
Pygo2
Pygo2
Pygo2
Pbxip1
AK053703
Flad1
Flad1
Flad1
Lenep
Cks1b
Shc1
Shc1
Pygo2
Pygo2
Pygo2
Pbxip1
AK053703
Flad1
Flad1
Flad1
Lenep
Cks1b
Shc1
Shc1
Pygo2
Pygo2
Pygo2
Pbxip1
AK053703
Flad1
Flad1
Flad1
Lenep
Cks1b
Shc1
Shc1
Pygo2
Pygo2
Pygo2
Pbxip1
AK053703
Flad1
Flad1
Flad1
Lenep
Cks1b
Shc1
Shc1
Pygo2
Pygo2
Pygo2
Pbxip1
AK053703
Flad1
Flad1
Flad1
Lenep
Cks1b
Shc1
Shc1
Pygo2
Pygo2
Pygo2
Pbxip1
Flad1
Flad1
Flad1
Cks1b
Shc1
Shc1
Pygo2
Pygo2
Pygo2
Pbxip1
Pmvk
Pmvk
Il6ra
Atp8b2
Atp8b2
Hax1
Ubap2l
Ubap2l
Ubap2l
Ubap2l
Tpm3
Tpm3
Tpm3
Tpm3
Tpm3

Tpm3
Tpm3
Tpm3
Tpm3
Tpm3
Tpm3
Tpm3
Rps27
Rab13
Rab13
Jtb
Rps27
Jtb
Slc39a1
Creb3l4
Creb3l4
Creb3l4
Slc39a1
Crtc2
Crtc2
Crtc2
Dennd4b
Dennd4b
Dennd4b
Creb3l4
Creb3l4
Creb3l4
Slc39a1
Crtc2
Crtc2
Crtc2
Dennd4b
Dennd4b
Dennd4b
Creb3l4
Creb3l4
Creb3l4
Slc39a1
Crtc2
Crtc2
Crtc2
Dennd4b
Dennd4b
Dennd4b
Crtc2
Crtc2
Crtc2
Dennd4b
Dennd4b
Dennd4b
Gatad2b
Npr1
Ilf2
Ilf2
Ilf2
Pogz
Pogz
Rfx5
Rfx5
Rfx5
Rfx5
Pi4kb
AK042619
AK042619
Zfp687
mKIAA1441
Pip5k1a
Pip5k1a
Vps72
Tmod4
Tmod4
Gabpb2
Gabpb2
AK020406
Mllt11
Cdc42se1
Cdc42se1
Gabpb2
Gabpb2
AK020406
Mllt11
Cdc42se1
Cdc42se1
Gabpb2
Gabpb2
AK020406
Mllt11
Cdc42se1
Cdc42se1
Gabpb2
Gabpb2
Mllt11
Cdc42se1
Cdc42se1
Gabpb2
Gabpb2
Mllt11
Cdc42se1
Cdc42se1
Prune
4930504E06Rik
4930504E06Rik
4930504E06Rik
4930504E06Rik
4930504E06Rik
Anxa9
Anxa9
AK144859
Lass2
Lass2
Arnt
Arnt
Arnt
Ctss
Hormad1
Hormad1
Golph3l
Golph3l
Adamtsl4
4930535B03Rik
4930535B03Rik
4930535B03Rik
mKIAA0460
Prpf3
Prpf3
Mrps21
Mrps21
AK044447
BC028528
BC028528
Car14
Car14
BC028528
BC028528
Car14
Car14
BC028528
BC028528
Car14
Car14
Anp32e
AK028005
AK028005
Otud7b
Otud7b
Otud7b
Otud7b
Hist2h4
Hist2h3b

Hist2h2bb
Hist2h4
Hist2h3b
Hist2h2bb
Fcgr1
Fcgr1
AK135774
AK135774
AK145360
AK135774
AK145360
AK135774
AK145360
AK135774
AK145360
BC107364
AK143784
Txnip
AK052161
AK143784
Txnip
AK052161
AK143784
AK052161
Polr3gl
Ankrd34
AK032024
NR_003619
Rbm8a
Pex11b
Pex11b
Pex11b
Pex11b
Itga10
Itga10
Ankrd35
Ankrd35
Ankrd35
Ankrd35
Pias3
Pias3
Pias3
Pias3
Pias3
Pias3
Zfp364
Gpr89
Gpr89
Acp6
Acp6
Bcl9
Bcl9
AK145276
Chd1l
Chd1l
Fmo5
Fmo5
Fmo5
Pde4dip
Pde4dip
Pde4dip
Tbx15
Ttf2
Ttf2
Slc22a15
Slc22a15
Csde1
Csde1
mKIAA0885
Syt6
Syt6
Syt6
Syt6
Syt6
Olfml3
Olfml3
AK031871
Rsbn1
Rsbn1
Magi3
Magi3
Lrig2
Lrig2
7530404M11Rik
Ppm1j
Rhoc
Mov10
Mov10
Mov10
AK048523
Wnt2b
Rbm15
Csf1
Csf1
Csf1
Csf1
Gstm1
Gnai3
Cyb561d1
Cyb561d1
Atxn7l2
Sypl2
Cyb561d1
Cyb561d1
Atxn7l2
Sypl2
Cyb561d1
Cyb561d1
Atxn7l2
Sypl2
AK005839
Psma5
AK005839
Psma5
mKIAA0893
Wdr47
Gpsm2
AK164420
Stxbp3a
Stxbp3a
Prpf38b
Prpf38b
Vav3
Vav3
Ntng1
Ntng1
netrin-G1
Ntng1
Ntng1
Ntng1
AK053689
Prmt6
Prmt6
Prmt6
Prmt6
Olfm3
Slc30a7
Extl2
Extl2
Slc30a7
Extl2
Extl2
Cdc14a
Cdc14a
Rtcd1
Rtcd1
Dbt
Snx7
Arhgap29

Arhgap29
Arhgap29
Arhgap29
Arhgap29
Bcar3
AK152002
Pde5a
Pde5a
BC096391
BC025067
Camk2d
Camk2d
Camk2d
mKIAA4163
Camk2d
Camk2d
Ank2
Ank2
Ank2
Ank2
Neurog2
Neurog2
Pitx2
Pitx2
Pitx2
Pitx2
Pitx2
Pitx2
Pitx2
Pitx2
Pitx2
Elovl6
FAE
Elovl6
Pla2g12a
Pla2g12a
Lef1
BC051212
Lef1
Lef1
Lef1
Scye1
A630047E20Rik
A630047E20Rik
Ppa2
Ppa2
Cisd2
Cisd2
AK015999
Ube2d3
Ube2d3
Ube2d3
Ube2d3
Nfkb1
Nfkb1
H2afz
Dnajb14
Dnajb14
Mttp
Mttp
Rg9mtd2
Rnmtd2
Rg9mtd2
Unc5c
Unc5c
Unc5c
Gbp1
Gbp3
Gbp3
Gbp3
Pkn2
Pkn2
Pkn2
AK035466
Lmo4
Lmo4
Lmo4
Lmo4
Lmo4
Lmo4
Lmo4
Lmo4
Lmo4
Lmo4
Lmo4
Lmo4
Hs2st1
Sep-15
Clca5
Clca5
Odf2l
Odf2l
Cyr61
Wdr63
Mcoln3
Mcoln3
Mcoln2
Mcoln2
Edg7
Bxdc5
Bxdc5
Lphn2
Lphn2
Lphn2
Lphn2
Semcap2
Gipc2
Dnajb4
Dnajb4
AK077587
Fubp1
Fubp1
Fubp1
Fubp1
Fubp1
Fubp1
Lhx8
AK142074
Gpr177
Gpr177
Gpr177
Gpr177
Gpr177
Gpr177
Plekhf2
AK080292
Ccne2
Ccne2
Rbm35a
Rbm35a
Rbm35a
Ppm2c
Ppm2c
AK007255
AK005538
Runx1t1
Cbfa2t1h
Runx1t1
Cbfa2t1h
Tmem55a
Tmem55a
mt3-mmp
Mmp16
mt3-mmp
Mmp16
Fam77D
E130310K16Rik
E130310K16Rik
Fam77D

E130310K16Rik
E130310K16Rik
Prdm13
DQ722851
AK081407
AK083021
AK031964
BC060111
AK031964
BC060111
AK077040
Coq3
6230409E13Rik
6230409E13Rik
AK039117
Pou3f2
AK039117
Pou3f2
AK039117
Pou3f2
AK039117
Pou3f2
Fut9
Epha7
Epha7
Epha7
Map3k7
Gja10
Casp8ap2
Casp8ap2
Gja10
Casp8ap2
Casp8ap2
Lyrm2
Rragd
Rragd
Mobkl2b
Mobkl2b
Aptx
AK139295
Dnaja1
B4galt1
AK142614
Bag1
Chmp5
Chmp5
Nol6
Ube2r2
Ubap2
Ubap2
Wdr40a
Cntfr
Cntfr
Cntfr
2810432D09Rik
Dctn3
Arid3c
Vcp
Pigo
Pigo
Pigo
Stoml2
B230312A22Rik
B230312A22Rik
B230312A22Rik
B230312A22Rik
AK043958
Stoml2
B230312A22Rik
B230312A22Rik
B230312A22Rik
B230312A22Rik
AK043958
Unc13b
Unc13b
Rusc2
Rusc2
mKIAA0375
4833436C18Rik
Tesk1
Sit1
NR_001460
LOC622404
Car9
Tln1
mKIAA1027
Creb3
1110029E03Rik
1110029E03Rik
Gne
Gne
Gne
Gne
Gne
Gne
Zcchc7
D4Wsu132e
Zbtb5
Zbtb5
Polr1e
Fbxo10
Fbxo10
Tdrd7
Tdrd7
Tdrd7
BC057893
BC057893
AU014645
AU014645
AU014645
BC057893
BC057893
AU014645
AU014645
AU014645
Foxe1
5830415F09Rik
5830415F09Rik
Trim14
Trim14
Trim14
BC038520
Nr4a3
Nr4a3
Nr4a3
BC038520
Nr4a3
Nr4a3
Nr4a3
BC038520
Nr4a3
Nr4a3
Nr4a3
BC038520
Nr4a3
Nr4a3
Nr4a3
mKIAA4116
mKIAA4116
Rnf20
Rnf20
mKIAA1973
mKIAA1973
Abca1
Slc44a1
Slc44a1
Zfp462
Zfp462
Akap2

Rod1
Hsdl2
Hsdl2
Hsdl2
Hsdl2
1110054O05Rik
1110054O05Rik
Cdc26
Cdc26
Cdc26
Cdc26
Fkbp15
Slc31a1
Cdc26
Cdc26
Cdc26
Cdc26
Fkbp15
Slc31a1
Cdc26
Cdc26
Prpf4
Hdhd3
Alad
Alad
4933430I17Rik
Hdhd3
Alad
Alad
4933430I17Rik
Hdhd3
Alad
Alad
4933430I17Rik
Hdhd3
Alad
Alad
4933430I17Rik
Hdhd3
Alad
Alad
4933430I17Rik
Akna
Akna
Akna
Whrn
Whrn
Jmjd2c
Jmjd2c
D4Bwg0951e
Nfib
Nfib
Nfib
Nfib
Nfib
Nfib
Nfib
Nfib
Nfib
Nfib
Nfib
Nfib
Zdhhc21
Gramp3
Psip1
Psip1
Psip1
4930473A06Rik
4930473A06Rik
AK029637
4930473A06Rik
4930473A06Rik
AK029637
Bnc2
Bnc2
Bnc2
6230416J20Rik
Dennd4c
C87499
Cdkn2a
Cdkn2a
AK148321
Cdkn2a
Cdkn2a
AK148321
Elavl2
Elavl2
Elavl2
Elavl2
Elavl2
Elavl2
Elavl2
Elavl2
Plaa
Plaa
Ift74
E130114P18Rik
Nfia
E130114P18Rik
Nfia
AK047309
Nfia
Nfia
Nfia
Dock7
Dock7
Foxd3
Cachd1
Cachd1
Cachd1
Cachd1
AK021368
AK002929
mmu-mir-101a
SGIP1alpha
Sgip1
Dab1
Dab1
Dab1
Dab1
Dab1
Dab1
Dab1
Dab1
Dab1
Ppap2b
Pcsk9
Ttc4
Ttc4
Ttc4
AK134749
Ssbp3
Ssbp3
Ssbp3
Ssbp3
Ssbp3
Ssbp3
AK164829
Glis1
AK164829
Glis1
Lrp8
Lrp8
Lrp8
Lrp8
Lrp8
Lrp8
Magoh
0610037L13Rik

0610037L13Rik
0610037L13Rik
Scp2
Zyg11a
Zyg11b
Zyg11b
Zcchc11
Zcchc11
Zcchc11
Prpf38a
2410002M20Rik
Orc1l
Orc1l
Prpf38a
2410002M20Rik
Orc1l
Orc1l
Prpf38a
2410002M20Rik
Orc1l
Orc1l
Nrd1
AK138781
Nrd1
mmu-mir-761
Osbpl9
AK019375
Dmrta2
4931433A01Rik
4931433A01Rik
2310026E23Rik
OTTMUSG00000008561
AK048089
AK078937
Foxd2
AK048089
AK078937
Tal1
Tal1
Tal1
Nsun4
Uqcrh
Lrrc41
Lrrc41
Rad54l
Rad54l
Rad54l
Rad54l
AK010894
Pik3r3
Pik3r3
Pik3r3
Ipp
Akr1a4
Akr1a4
AK131819
Prdx1
AK131819
Prdx1
Mmachc
0610037D15Rik
0610037D15Rik
Tesk2
Mmachc
Tesk2
Mmachc
Tesk2
Ptch2
9530048O09Rik
9530048O09Rik
4833401D15Rik
4931406I20Rik
4931406I20Rik
4931406I20Rik
4931406I20Rik
4931406I20Rik
4931406I20Rik
4931406I20Rik
4931406I20Rik
4931406I20Rik
4931406I20Rik
4931406I20Rik
4931406I20Rik
Dmap1
Slc6a9
St3gal3
St3gal3
Ptprf
Ptprf
Med8
Elovl1
Elovl1
Elovl1
Elovl1
Elovl1
AA415398
Foxj3
Foxj3
Foxj3
Kcnq4
Ppie
Nt5c1a
Macf
Macf
Macf1
Macf
Macf
Macf
Macf
Rragc
AK160260
Pou3f1
Pou3f1
Pou3f1
Utp11l
Fhl3
Sf3a3
Sf3a3
AK134673
Dnali1
Snip1
2310005N01Rik
2310005N01Rik
Stk40
Stk40
Thrap3
Thrap3
Thrap3
Mtap7d1
Mtap7d1
Mtap7d1
Trappc3
Mtap7d1
Mtap7d1
Mtap7d1
Trappc3
Mtap7d1
Mtap7d1
Mtap7d1
Trappc3
Eif2c3
Eif2c3
Eif2c3
Eif2c3
Eif2c1
Clspn
Clspn
Clspn
Clspn

Tcfap2e
Ncdn
Ncdn
AU040320
AU040320
AU040320
AU040320
AU040320
AU040320
AK019743
Ibrdc3
Rnf19b
Hpca
Hpca
Hpca
Fndc5
Hpca
Hpca
Hpca
Fndc5
2410081M15Rik
AK049295
Tssk3
Marcksl1
Marcksl1
Hdac1
Hdac1
Hdac1
Lck
Lck
Lck
Lck
Hdac1
Lck
Lck
Lck
Lck
Eif3i
Eif3s2
Iqcc
Ccdc28b
Ccdc28b
AK031278
Ccdc28b
Khdrbs1
Khdrbs1
Khdrbs1
Ptp4a2
Ptp4a2
Ptp4a2
Ptp4a2
Ptp4a2
Ptp4a2
AK005651
AK005651
AK005651
Col16a1
Col16a1
Fabp3
Pum1
Pum1
Pum1
Laptm5
Laptm5
Laptm5
Sfrs4
Sfrs4
Sfrs4
Sfrs4
Sfrs4
Sfrs4
Epb4.1
Epb4.1
Epb4.1
Epb4.1
Gmeb1
Gmeb1
Taf12
Gmeb1
Gmeb1
NR_002865
Taf12
Phactr4
Med18
Sesn2
AK155592
Sesn2
AK155592
Atpif1
Dnajc8
Eya3
Ppp1r8
Stx12
Stx12
Fgr
Ahdc1
Ahdc1
Ahdc1
Ahdc1
Ahdc1
Ahdc1
Ahdc1
Ahdc1
D4Ertd196e
Wdtc1
Wdtc1
Wdtc1
Slc9a1
2300002D11Rik
Arid1a
Arid1a
Arid1a
Arid1a
Arid1a
Arid1a
Arid1a
Arid1a
Arid1a
Arid1a
Dhdds
Dhdds
Lin28
Extl1
Extl1
Pafah2
Pafah2
Pafah2
Pafah2
Pafah2
Pafah2
Ldlrap1
Ldlrap1
Rhd
Rhd
Rhd
Rhd
Tmem50a
Tmem50a
D4Wsu53e
D4Wsu53e
Syf2
Tmem50a
Tmem50a
D4Wsu53e
D4Wsu53e
Syf2
Syf2
Clic4
Clic4

Clic4
Srrm1
Srrm1
Srrm1
Npal3
Npal3
4930555I21Rik
4930555I21Rik
Grhl3
nssr 1
Fusip1
Fusip1
Fusip1
nssr 1
Pnrc2
Hmgcl
Gale
Gale
Lypla2
Lypla2
Lypla2
Lypla2
1110049F12Rik
Id3
Id3
Id3
Id3
E2f2
Tcea3
Zfp46
Ephb2
Ephb2
Ephb2
Ephb2
Ephb2
Ephb2
C1qb
C1qc
C1qa
Cdc42
Cdc42
Cdc42
Hspg2
Ldlrad2
Usp48
Usp48
Usp48
Usp48
Sh2d5
MmKIF17
Kif17
Kif17
MmKIF17
Sh2d5
MmKIF17
Kif17
Kif17
MmKIF17
Camk2n1
Camk2n1
Tmco4
Htr6
Htr6
Nbl1
Pax7
Pax7
Rcc2
Rcc2
BC005803
Padi1
D4Ertd22e
Fbxo42
D4Ertd22e
Fbxo42
Arhgef19
Clcnka
Clcnka
BC047062
Clcnkb
Hspb7
Gm694
Zbtb17
Spen
Spen
Spen
AK148126
Spen
Spen
Spen
AK148126
Spen
Spen
Spen
AK148126
Spen
Spen
Spen
AK148126
Fblim1
Fblim1
AK131719
Fblim1
Fblim1
AK131719
Fblim1
Fblim1
Tmem82
AK131719
Tmem82
Slc25a34
Fblim1
Fblim1
Tmem82
Tmem82
Slc25a34
Ddi2
Tmem51
AK154034
AK158244
9030409G11Rik
9030409G11Rik
mKIAA1026
9030409G11Rik
AK018156
Pramef12
2610109H07Rik
Mad2l2
Mad2l2
Fbxo6
Fbxo6
Fbxo6
Fbxo44
Fbxo44
Fbxo44
Fbxo44
Fbxo2
Tardbp
Tardbp
Gm572
Casz1
Casz1
Casz1
Casz1
Casz1
Casz1
Pex14
Pex14
Kif1b
Kif1b

Ube4b
Ube4b
Clstn1
D4Ertd429e
D4Ertd429e
D4Ertd429e
D4Ertd429e
D4Ertd429e
D4Ertd429e
D4Ertd429e
D4Ertd429e
D4Ertd429e
D4Ertd429e
Spsb1
H6pd
H6pd
Rere
Thap3
Phf13
Phf13
Klhl21
Tnfrsf25
Espn
Espn
Espn
Espn
Espn
Prdm16
5930403L14Rik
AK083203
Vwa1
A230069A22Rik
AK054201
Mrpl20
Dvl1
Dvl1
mKIAA4029
Dvl1
Cpsf3l
Ttll10
Ttll10
Cdk6
Cdk6
mKIAA1386
Ankib1
Ankib1
mKIAA1386
Akap9
Akap9
Akap9
Akap9
Fzd1
Pftk1
Pftk1
Pftk1
Pftk1
Steap2
Steap2
Steap2
Rundc3b
Dmtf1
Dmtf1
Dmtf1
Dmtf1
AK037159
Sema3a
Sema3a
Sema3a
Gnai1
AK046351
Ptpn12
Fbxl13
Fbxl13
Armc10
Armc10
Dnajc2
Dnajc2
Dnajc2
Dnajc2
Psmc2
Reln
Reln
Reln
Srpk2
Srpk2
Srpk2
Srpk2
Srpk2
Srpk2
Kcnh2
Nos3
Gbx1
Abcf2
2010209O12Rik
AK012891
mmu-mir-671
Abcf2
2010209O12Rik
AK012891
mmu-mir-671
Abcf2
2010209O12Rik
AK012891
mmu-mir-671
Smarcd3
Smarcd3
Nub1
Rheb
Mll3
mKIAA1506
Mll3
AK149295
Gm443
Dpp6
Dpp6
Dpp6
Dpp6
Dpp6
Dpp6
Dpp6
Dpp6
9630008K15Rik
Cnpy1
AK039125
AK132123
Gm444
AK132123
Gm444
Hadha
Hadha
Hadhb
Hadhb
mmu-mir-1960
Hadha
Hadha
Hadhb
Hadhb
mmu-mir-1960
Hadha
Hadha
Hadhb
Hadhb
mmu-mir-1960
D5Wsu178e
mKIAA1724
D5Wsu178e
mKIAA1724
4930471M23Rik
Cenpa

Cenp-a
Dpysl5
Dpysl5
Crmp5
Agbl5
9430057O19Rik
Agbl5
Agbl5
Agbl5
Agbl5
Agbl5
2310016E02Rik
Emilin1
Emilin1
Khk
2310016E02Rik
Emilin1
Emilin1
Khk
Slc30a3
Slc30a3
Dnajc5g
Slc30a3
Slc30a3
Dnajc5g
Trim54
Ucn
Gtf3c2
Gtf3c2
Gtf3c2
Gtf3c2
Snx17
Gtf3c2
Gtf3c2
Gtf3c2
Gtf3c2
Eif2b4
Eif2b4
Snx17
Gtf3c2
Gtf3c2
Gtf3c2
Gtf3c2
Eif2b4
Eif2b4
Snx17
Fosl2
Fosl2
Yes1
Yes1
Pisd
Ywhah
Ctbp1
Ctbp1
CtBP1
Slbp
Slbp
Tmem129
Tmem129
Tacc3
Tacc3
Tacc3
Nol14
Grk4
Grk4
Nol14
Grk4
Grk4
Sorcs2
Sorcs2
AK144870
Grpel1
Msx1
Stx18
Stx18
Drd5
Wdr1
Wdr1
Hs3st1
Hs3st1
Rab28
Rab28
A230054D04Rik
A230054D04Rik
AK017756
AK136254
Gpr125
Dhx15
Dhx15
AK163067
AK050940
AK079034
Anapc4
Rbpj
Rbpj
Rbpj
Rbpj
Stim2
AK039574
Pcdh7
Pcdh7
Pgm1
Tbc1d1
mKIAA1108
Tbc1d1
Klf3
Klf3
Klf3
Klf3
Klf3
Klf3
Klf3
Klf3
Pds5a
AK006245
B3bp
B3bp
Limch1
Limch1
mKIAA1102
Limch1
Phox2b
Phox2b
Tmem33
Tmem33
Tmem33
Tmem33
Atp8a1
Gabrb1
Tec
Tec
Tec
Dcun1d4
Dcun1d4
Pdgfra
Pdgfra
Pdgfra
Pdgfra
Pdgfra
Pdgfra
Epha5
Epha5
Epha5
Epha5
Epha5
Epha5
Epha5
Cenpc1
Cenpc1

Cenpc1	
stap-1	
stap-1	
Stap1	
Rufy3	
Rufy3	
Rufy3	
Rufy3	
Cox18	
Ankrd17	
Ankrd17	
1110019K23Rik	
Cdkl2	
Cdkl2	
Cdkl2	
Nup54	
Nup54	
Scarb2	
Ankrd56	
	Sep-11
	Sep-11
	Sep-11
	Sep-11
	Sep-11
	Sep-11
	Sep-11
	Sep-11
Ccni	
Ccni	
Cnot6l	
Cnot6l	
Cnot6l	
Prdm8	
Bmp3	
Bmp3	
Hnrpd	
Hnrpdl	
Hnrpdl	
Hnrpdl	
Enoph1	
Enoph1	
Hnrpdl	
Hnrpdl	
Hnrpdl	
Enoph1	
Enoph1	
AI461788	
Lin54	
mKIAA2037	
AI461788	
Cops4	
Cops4	
Ccdc98	
Ccdc98	
AK133807	
A230097K15Rik	
A230097K15Rik	
Nkx6-1	
Nkx6-1	
Arhgap24	
Arhgap24	
Arhgap24	
Ptpn13	
Ptpn13	
Ptpn13	
Aff1	
Aff1	
Aff1	
Aff1	
Aff1	
Aff1	
Nudt9	
Dspp	
Dspp	
Barhl2	
Cdc7	
Cdc7	
Tgfbr3	
Tgfbr3	
Tgfbr3	
Tgfbr3	
Brdt	
Fsrg3	
Glmn	
Rpap2	
Rpap2	
Rpap2	
Mtf2	
Mtf2	
Mtf2	
Mtf2	
Tmed5	
Tmed5	
Ccdc18	
Ccdc18	
Tmed5	
Tmed5	
Ccdc18	
Ccdc18	
Tmed5	
Tmed5	
Ccdc18	
Ccdc18	
Tmed5	
Tmed5	
Ccdc18	
Ccdc18	
Atp5k	
AK142681	
Gtpbp6	
A830023I12Rik	
A830023I12Rik	
Tpst2	
Tpst2	
Myo18b	
AK085912	
AK085912	
Adrbk2	
Adrbk2	
Gm854	
Wscd2	
Wscd2	
Foxn4	
BC057022	
Sppl3	
Sppl3	
Pop5	
Pop5	
Rnf10	
Rnf10	
Coq5	
Coq5	
Dynll1	
Dynll1	
Dynll1	
Sfrs9	
Msi1	
Msi1	
Sirt4	
Sirt4	
Pxn	
Ccdc60	
Hspb8	
1500001A10Rik	
1500001A10Rik	
AK050700	
Wsb2	
Wsb2	
Wsb2	

Wsb2
Ksr2
Nos1
Nos1
Fbxw8
Fbxw8
Hrk
Fbxw8
Fbxw8
Hrk
Thrap2
Med13l
Med13l
Thrap2
Med13l
Med13l
Med13l
Tbx3
Tbx3
Tbx3
Tbx3
Rbm19
Lhx5
Lhx5
1110008J03Rik
Ddx54
Acad10
Acad10
Brap
Brap
Atxn2
Sh2b3
A230106M15Rik
Cutl2
Ppp1cc
Ppp1cc
Rad9b
Rad9b
Vps29
Atpbd1c
Anapc7
Anapc7
Cdv1R
Ift81
Cdv1R
Ift81
Fbxl10
Fbxl10
Fbxl10
mKIAA3014
AK079939
Tmem120b
Rhof
Rhof
Setd1b
Setd1b
Rhof
Rhof
Setd1b
Setd1b
Rhof
Rhof
Setd1b
Setd1b
Psmd9
AK030281
Bcl7a
Bcl7a
Bcl7a
Bcl7a
Vps33a
Zcchc8
Zcchc8
Rsrc2
Rsrc2
Rsrc2
Rsrc2
Rsrc2
Kntc1
Mphosph9
Mphosph9
Mphosph9
Mphosph9
Mphosph9
2810006K23Rik
2810006K23Rik
BC003324
6330548G22Rik
Ccdc92
Zfp664
Ubc
Ubc
Ubb
Ubc
Ubb
AK183279
Ubb
AK004727
Ubc
Ubc
Ubb
Ubc
Ubb
AK183279
Ubb
AK004727
Caln1
Caln1
Caln1
Wbscr17
Wbscr17
mpp-GalNAc-T17(O6)
Auts2
Auts2
Wbscr16
Gtf2ird2
Gtf2ird2
Gtf2ird2
Ncf1
Ncf1
Ncf1
Gtf2i
Gtf2i
Gtf2i
Gtf2i
Diws1t
Gtf2ird1
Gtf2ird1
Gtf2ird1
Gtf2ird1
Gtf2ird1
Eif4h
Eif4h
Limk1
Limk1
Limk1
Mlxipl
Mlxipl
BCL7B
Bcl7b
Baz1b
Baz1b
Baz1b
Baz1b
Hip1
Por
Srcrb4d
Srcrb4d
Srcrb4d

Zp3
1200011O22Rik
1200011O22Rik
Alkbh4
Alkbh4
Cutl1
Cutl1
Cutl1
Cutl1
Cux/CDP(1B1)
Cutl1
Cutl1
Cutl1
Cutl1
Cux/CDP(1B1)
Emid2
Emid2
Emid2
Fis1
Cldn15
Znhit1
Znhit1
Plod3
Plod3
mmu-mir-702
Trim56
Trim56
Ache
Ache
Ache
Ache
2700038N03Rik
Ars2
Ars2
Ars2
Trip6
Slc12a9
Ars2
Ars2
Trip6
Slc12a9
Slc12a9
Slc12a8
Ephb4
Ephb4
Slc12a9
Slc12a8
Ephb4
Ephb4
Epo
Gnb2
Pop7
mKIAA4110
Perq1
Hrbl
Hrbl
Hrbl
BC028660
6430598A04Rik
6430598A04Rik
6430598A04Rik
Tsc22d4
Tsc22d4
Tsc22d4
Mepce
Zcwpw1
Zfp113
COPS6
Cops6
COPS6
Ap4m1
Pdgfa
1110007L15Rik
1110007L15Rik
Centa1
Mad1l1
Mad1
Grifin
Lfng
Lfng
Zfp469
Ocm
Smurf1
Rpl21
Rpl21
Rasl11a
Rasl11a
Lnx2
Rpo1-3
Rpo1-3
Gsx1
AK008755
Pdx1
Cdx2
Cdx2
Prhoxnb
AK038337
Pan3
Pan3
AK082086
Uspl1
Uspl1
Uspl1
Fry
BC037393
Pds5b
AK021342
AK006454
Ppp1r9a
Ppp1r9a
Neurabin
Ppp1r9a
Ppp1r9a
AK139424
Nxph1
Nxph1
Nxph1
Nxph1
Nxph1
Nxph1
Ndufa4
Phf14
Phf14
AK173072
Tmem168
2610001J05Rik
AK003812
AK143417
Tes
Met
Met
St7
St7
St7
Fam4a2
Fam4a2
St7
Fam4a2
Kcnd2
Tspan12
AK040992
Cadps2
Cadps2
Grm8
Grm8
Grm8A
Zfp800
Zfp800
AK052319

Impdh1
Impdh1
Calu
Tnpo3
Tnpo3
Tnpo3
Tnpo3
Tspan33
4631427C17Rik
4631427C17Rik
4631427C17Rik
4631427C17Rik
4631427C17Rik
4631427C17Rik
mmu-mir-182
mmu-mir-96
mmu-mir-183
Zc3hc1
Zc3hc1
2410127E18Rik
Chchd3
Chchd3
4921528H16Rik
4921528H16Rik
4921528H16Rik
4921528H16Rik
Cnot4
Cnot4
Cnot4
Cnot4
Cnot4
Cnot4
Mtpn
Dgki
Dgki
Zc3hav1
Zc3hav1
AK167137
BC099561
D130059P03Rik
D130059P03Rik
BC099561
D130059P03Rik
D130059P03Rik
BC099561
D130059P03Rik
D130059P03Rik
Luc7l2
Luc7l2
Luc7l2
AK015545
Luc7l2
Luc7l2
Luc7l2
Luc7l2
Klrg2
Hipk2
Tbxas1
Tbxas1
Tbxas1
Tbxas1
Mrps33
Agk
Agk
Agk
E330009J07Rik
E330009J07Rik
Wee2
AK008907
Arhgef5
Arhgef5
AK008907
Arhgef5
Arhgef5
NR_002841
Atp6v0e2
Lrrc61
Lrrc61
Lrrc61
Lrrc61
Lrrc61
Lrrc61
Igf2bp3
mimp3
Igf2bp3
mimp3
Igf2bp3
mimp3
Igf2bp3
mimp3
Igf2bp3
mimp3
Tra2a
AK076606
Hnrnpa2b1
Hnrnpa2b1
Hnrnpa2b1
hnRNP A2/B1
Cbx3
Hoxa1
Hoxa1
Hoxa2
Hoxa3
Hoxa3
AK142386
AK039882
AK146012
Hoxa1
Hoxa1
Hoxa2
Hoxa3
Hoxa3
AK039882
AK146012
Hoxa3
Hoxa3
AK146012
Hoxa4
AK012572
Hoxa5
Hoxa9
Hoxa10
Hoxa11
U20367
U20366
U20369
AK133929
mmu-mir-196b
AK031498
Evx1
AK031498
Evx1
Jazf1
AK035031
9430076C15Rik
Creb5
Creb5
AK035031
9430076C15Rik
Creb5
Creb5
Cpvl
Chn2
Chn2
AK020588
Pigy
Pigy
Lancl2
Grid2

Grid2
Grid2
Atoh1
A930038C07Rik
Serbp1
Serbp1
Serbp1
abParts
abParts
Eif2ak3
Eif2ak3
Rmnd5a
Jmjd1a
Jmjd1a
Jmjd1a
Jmjd1a
St3gal5
Vamp5
Vamp8
Tcf3
Tcf3
Tcf3
Sucla1
Suclg1
Ctnna2
Ctnna2
Lrrtm1
Lrrtm1
Lrrtm1
Hk2
D6Mm5e
D6Mm5e
Dok1
Dok1
Loxl3
Loxl3
Htra2
HtrA2
HtrA2
HtrA2
Aup1
Aup1
Dqx1
Dqx1
Dqx1
Htra2
HtrA2
HtrA2
HtrA2
Tlx2
PMUR10F
Pcgf1
Pcgf1
Lbx2
Ccdc142
Ccdc142
Mrpl53
Gcs1
mmu-mir-3470a
Wbp1
Znhit4
Rtkn
Rtkn
Rtkn
1700003E16Rik
1700003E16Rik
NR_002871
NR_002873
AK044313
AY589788
Vax2
Paip2b
AK144571
Cyp26b1
Cyp26b1
mKIAA0919
Sec15l2
Sec15l2
Emx1
Pcyox1
Tia1
Tia1
Tia1
C87436
AK052590
AK079912
Pcbp1
BC029726
AK005503
AK079912
Pcbp1
BC029726
AK005503
BC029726
AK005503
Anxa4
Nfu1
Rpn1
AK157963
AK157963
Wnt7a
Wnt7a
Tmem43
Chchd4
Tmem43
Xpc
Lsm3
Xpc
Lsm3
Slc6a6
Slc6a6
Slc6a6
Slc6a6
C130022K22Rik
C130022K22Rik
C130022K22Rik
C130022K22Rik
Fgd5
Fgd5
Nr2c2
Nr2c2
Zfyve20
Trh
Prickle2
Prickle2
Prickle2
Prickle2
Prickle2
AK007436
AK044653
AK044242
AK007436
AK044653
AK044242
AK007436
AK044653
AK044242
AK007436
AK044653
AK044242
Magi1
Magi1
Magi1
Magi1
Magi1
Magi1
Magi1
Magi1
Magi1

Magi1
Magi1
Magi1
Magi1
Magi1
Lrig1
Lrig1
Suclg2
Suclg2
Suclg2
C130034I18Rik
Frmd4b
Frmd4b
Frmd4b
Frmd4b
Frmd4b
Frmd4b
Frmd4b
GOBLIN
Mitf
Mitf
Mitf
Mitf
Mitf
Mitf
Foxp1
Foxp1
Foxp1
Foxp1
Foxp1a
AK051887
AK051887
AK051887
Prok2
Prok2
Rybp
Shq1
Glt8d4
Ppp4r2
Ppp4r2
Ppp4r2
Ppp4r2
Pdzrn3
Semcap3
Pdzrn3
Semcap3
Pdzrn3
Semcap3
Chl1
Chl1
Chl1
Chl1
Cntn4
Cntn4
Cntn4
Cntn4
Lrrn1
Lrrn1
Itpr1
Itpr1
Itpr1
Itpr1
Itpr1
AK039720
AK042445
Bhlhb2
AK039720
AK042445
Bhlhb2
Arl8b
Grm7
Grm7
Srgap3
Srgap3
Srgap3
Srgap3
Thumpd3
Thumpd3
AK021028
U83175
U83174
mKIAA1757
Setd5
AK021028
U83175
U83174
mKIAA1757
Setd5
AK021028
U83175
U83174
mKIAA1757
Setd5
AK021028
U83175
U83174
mKIAA1757
Setd5
AK021028
U83175
U83174
mKIAA1757
Setd5
mKIAA1757
Setd5
Setd5
mKIAA1757
Cpne9
Brpf1
Brpf1
Brpf1
Brpf1
Brpf1
Brpf1
Brpf1
AK016007
Brpf1
Ogg1
Camk1
Camk1
Arpc4
Tada3l
Tada3l
Tada3l
Arpc4
Ttll3
Ttll3
Ttll3
Cidec
Cidec
Jagn1
Jagn1
Jagn1
Il17re
Prrt3
Prrt3
Prrt3
Tmem111
AK164779
Fancd2
Fancd2
4931417G12Rik
6720456B07Rik
Vhlh
Slc6a1
Slc6a1
Slc6a1
Slc6a1
Slc6a1

Slc6a1	
Hrh1	
Hrh1	
Vgll4	
Vgll4	
Vgll4	
Vgll4	
Vgll4	
AK076253	
AK078466	
Vgll4	
Vgll4	
AK078466	
Pparg	
Raf1	
Raf1	
Raf1	
Raf1	
Raf1	
Raf1	
Rpl32	
BC060267	
Mbd4	
Mbd4	
Ift122	
mFLJ00025	
H1foo	
Plxnd1	
Plxnd1	
Plxnd1	
Tmcc1	
Tmcc1	
Tmcc1	
D6Wsu116e	
D6Wsu116e	
D6Wsu116e	
	Mar-08
	Mar-08
Rassf4	
Rassf4	
Rassf4	
Cxcl12	
Cxcl12	
Cxcl12	
Cxcl12	
AK006540	
AK006540	
AK006540	
AK006540	
AK006540	
Hnrpf	
Hnrpf	
AK180911	
Rasgef1a	
Bms1	
Bms1	
Bms1	
Cacna1c	
Cacna1c	
Adipor2	
Adipor2	
Adipor2	
Adipor2	
Adipor2	
Adipor2	
Adipor2	
Wnt5b	
Wnt5b	
Wnt5b	
Wnt5b	
Wnt5b	
Fbxl14	
Wnt5b	
Wnt5b	
Fbxl14	
Wnt5b	
Wnt5b	
Fbxl14	
Wnt5b	
Wnt5b	
Fbxl14	
Wnt5b	
Wnt5b	
Fbxl14	
Wnt5b	
Erc1	
Erc1	
Erc1	
Wnk1	
Wnk1	
Wnk1	
Wnk1	
mmu-mir-706	
Ninj2	
B4galnt3	
Ccdc77	
Ccdc77	
Ccdc77	
Ccdc77	
Jarid1a	
Jarid1a	
Ccdc77	
Ccdc77	
Ccdc77	
Jarid1a	
Jarid1a	
Cecr6	
Cecr5	
AK006942	
AK137536	
AK137536	
AK137536	
Atp6v1e1	
Bcl2l13	
Bcl2l13	
Pex26	
Tuba8	
Usp18	
Usp18	
M6pr	
M6pr	
Phc1	
Phc1	
Phc1	
Phc1	
Phc1	
Phc1	
Phc1	
Phc1	
Phc1	
Phc1	
Phc1	
Phc1	
Phc1	
Phc1	
Phc1	
mKIAA1238	
4933426K21Rik	
4933426K21Rik	
4933426K21Rik	
Apobec1	
Apobec1	
Slc2a3	
Foxj2	
Clstn3	
AK213543	
Clstn3	
AK213543	
C1s	

C1s
Mboat5
Ptpn6
Ptpn6
Ptpn6
Grcc10
Atn1
Grcc10
Atn1
Atn1
Ptpn6
Ptpn6
Ptpn6
Grcc10
Atn1
Grcc10
Atn1
Atn1
Ptpn6
Ptpn6
Grcc10
Atn1
Grcc10
Atn1
Atn1
Ptpn6
Ptpn6
Grcc10
Atn1
Grcc10
Atn1
Atn1
Atn1
Atn1
Atn1
Eno2
Lrrc23
Spsb2
Spsb2
Spsb2
Spsb2
Spsb2
Spsb2
Tpi1
Tpi1
Cdca3
Tpi1
Usp5
Cdca3
Usp5
Gnb3
Leprel2
Gnb3
Leprel2
Gnb3
Leprel2
Gpr162
Gnb3
Leprel2
Gpr162
Leprel2
Gpr162
Cd4
Leprel2
Gpr162
Cd4
Cd4
Cd4
Cd4
Lag3
A230083G16Rik
Cd4
Cd4
Cd4
Lag3
A230083G16Rik
Lag3
Ptms
A230083G16Rik
Mlf2
Mlf2
Mlf2
Lag3
Ptms
A230083G16Rik
Mlf2
Mlf2
Mlf2
Lag3
Ptms
A230083G16Rik
Mlf2
Mlf2
Mlf2
Lag3
Ptms
A230083G16Rik
Mlf2
Mlf2
Mlf2
Lag3
Ptms
A230083G16Rik
Mlf2
Mlf2
Mlf2
Chd4
mKIAA4075
Chd4
Chd4
mKIAA4075
Nol1
A930037G23Rik
Gapdh
Gapdh
Ncapd2
Ncapd2
AK013380
bMRP64
Mrpl51
mmu-mir-3098
Vamp1
mVAMP-1
AK080173
mVAMP-1
Tapbpl
AK080173
Cd27
mVAMP-1
Tapbpl
AK080173
Cd27
Ltbr
Scnn1a
Tnfrsf1a
Tnfrsf1a
Tnfrsf1a
Plekhg6
Cd9
AK046349
Cd9
AK046349
Ntf3
Ntf3
Kcna1
Kcna1
Ndufa9
Akap3
Dyrk4

Dyrk4
Rad51ap1
D6Wsu163e
D6Wsu163e
D6Wsu163e
Fgf23
9630033F20Rik
Ccnd2
Ccnd2
AK043994
AK020388
AK187594
Ccnd2
Ccnd2
AK043994
AK020388
AK187594
Ccnd2
Ccnd2
AK043994
AK020388
AK187594
Ccnd2
Ccnd2
AK043994
AK020388
AK187594
Parp11
Parp11
Parp11
Parp11
Parp11
Parp11
Prmt8
Prmt8
Tspan9
Tspan9
Tead4
Tead4
Tulp3
Tulp3
5930416I19Rik
5930416I19Rik
Foxm1
Tulp3
Tulp3
5930416I19Rik
5930416I19Rik
Foxm1
4933413G19Rik
Tulp3
Tulp3
5930416I19Rik
5930416I19Rik
Foxm1
4933413G19Rik
Itfg2
Itfg2
Itfg2
Fkbp4
AK029950
BC091771
DQ689702
DQ697726
DQ568996
DQ704729
DQ687171
DQ701953
DQ711509
DQ704526
DQ726464
DQ726464
DQ726464
Klrb1c
Klrb1b
Klrb1d
Klrb1c
Klrb1b
Klrb1d
Klrb1b
Klrb1d
Klrb1d
Klrb1b
Klrb1d
Klrb1d
Klrb1b
Klrb1d
Klrb1d
Klrb1b
Klrb1d
Klrb1d
Klrb1b
Klrb1d
Klrb1d
Klrb1b
Klrb1d
Klrb1d
Klrb1b
Klrb1d
Klrb1d
Klrb1b
Klrb1d
Klrb1d
Klrb1f
Csda
Etv6
Etv6
Etv6
Loh12cr1
Dusp16
MKP-7
Dusp16
Gpr19
Gpr19
Gpr19
Gpr19
Gpr19
Gpr19
Gpr19
Gpr19
Gpr19
Gpr19
Gpr19
Gpr19
Gpr19
Gpr19
Gpr19
Gpr19
Gpr19
Gpr19
Cdkn1b
Cdkn1b
AK012387
E330021D16Rik
Hist4h4
H2afj
Hist4h4
H2afj
Rerg
Rerg
Rerg
Rerg
Rerg
Rerg
Plekha5
Plekha5
Plekha5
Plekha5
Plekha5

Plekha5
Aebp2
Aebp2
Aebp2
Aebp2
Pde3a
Pde3a
AK043509
Ldhb
Kcnj8
Bcat1
Bcat1
Las1
Casc1
Las1
Lyrm5
Kras
AK008222
Bhlhb3
Bhlhb3
Bhlhb3
Itpr2
TIPR
TIPR
TIPR
Itpr2
TIPR
TIPR
TIPR
4933424B01Rik
Fgfr1op2
Tm7sf3
Med21
Stk38l
Arntl2
Ppfibp1
EG545893
Klhdc5
Ccdc91
Ergic2
Ergic2
AK005757
Ipo8
Ipo8
Ipo8
Ipo8
Caprin2
Caprin2
Caprin2
Caprin2
Tera
Tera
AK087382
Tera
Tera
AK087382
Tera
Tera
AK087382
D030011O10Rik
D030011O10Rik
4833442J19Rik
4833442J19Rik
2810474O19Rik
2810474O19Rik
AK134400
mmu-mir-290
mmu-mir-291a
mmu-mir-292
mmu-mir-291b
mmu-mir-293
mmu-mir-294
mmu-mir-295
Prkcc
Rdh13
Rdh13
Eps8R1
Eps8l1
Eps8l1
Rdh13
Rdh13
Eps8R1
Eps8l1
Eps8l1
Eps8R1
Eps8l1
Eps8l1
1500019G21Rik
Brsk1
Brsk1
BC022651
BC022651
Suv420h2
Suv420h2
Suv420h2
Il11
BC126955
2210411K11Rik
Rpl28
Isoc2b
AK080857
Ccdc106
Ccdc106
Ccdc106
U2af2
U2af2
U2af2
Epn1
Zfp787
Zfp787
Zfp444
Zfp444
Zfp444
6430701C03Rik
6430701C03Rik
6430701C03Rik
Zfp329
Zfp329
2810007J24Rik
2810007J24Rik
Sepw1
Gltscr2
Gltscr2
Gltscr2
Ehd2
Ehd2
Gltscr1
Slc8a2
Mrg2
Bbc3
Bbc3
Sae1
Sae1
BC057627
Sae1
Sae1
BC057627
Grlf1
Strn4
Prkd2
Prkd2
Prkd2
Prkd2
Prkd2
Prkd2
Calm3
Pglyrp1
Dmpk
Dmpk

Six5
mmu-mir-3100
Vasp
Vasp
Nspl1
Rtn2
Rtn2
Fosb
Fosb
Mark4
Exoc3l2
BC024868
Gemin7
Zfp296
Zfp296
Relb
Clptm1
Clptm1
Apoc2
Apoc4
Apoc1
Apoc1
Apoe
Apoe
Tomm40
Tomm40
Tomm40
Apoe
Apoe
Tomm40
Tomm40
Tomm40
Tom40
Pvrl2
Pvrl2
Pvrl2
Bcam
Bcam
Cblc
Cblc
Cblc
Cblc
Cblc
Cblc
Bcl3
Bcl3
Zfp93
Zfp93
Cd79a
mFLJ00369
Arhgef1
Arhgef1
Arhgef1
Pou2f2
Pou2f2
Dedd2
Dedd2
Flame-3
Zfp526
9130221H12Rik
Cic
Cic
Cic
mKIAA0306
Cic
Cic
Cic
mKIAA0306
Pafah1b3
Pafah1b3
Tmem145
Megf8
Tmem91
Tmem91
Tmem91
Tmem91
B9d2
B9d2
Tgfb1
Ccdc97
Hnrpul1
Hnrpul1
Hnrpul1
Axl
Axl
Axl
Cyp2s1
Cyp2s1
Cyp2s1
Axl
Axl
Cyp2s1
Cyp2s1
Cyp2s1
Ltbp4
Ltbp4
Ltbp4
AK008665
AK191411
Sertad1
Prx
2310022A10Rik
2310022A10Rik
2310022A10Rik
Akt2
Zfp60
Zfp607
1700049G17Rik
1700049G17Rik
1700049G17Rik
AK076358
Supt5h
Supt5h
Supt5h
Supt5h
Rps16
Supt5h
Supt5h
Zfp36
Med29
Paf1
Samd4b
Samd4b
Gmfg
Gmfg
Gmfg
Lrfn1
Hnrnpl
Hnrnpl
Ech1
Actn4
Eif3k
Eif3s12
Eif3k
Map4k1
Map4k1
Map4k1
A230107C01Rik
A230107C01Rik
Neud4
Sipa1l3
Sipa1l3
Sipa1l3
Sipa1l3
Sipa1l3
mKIAA0545
Zfp82
Zfp566
Zfp260

0610010E21Rik
Nphs1
Nphs1
Prodh2
Gapdhs
BC053749
BC053749
Lin37
U2af1l4
Tmem149
U2af26
Tmem149
Hamp2
AK218399
AK154275
Usf2
Usf2
Lsr
Tmem162
Tmem162
2410004F06Rik
Nudt19
AK081136
Vstm2b
AK081136
Vstm2b
AK081136
Vstm2b
Lim2
Nkg7
Cldnd2
BC112410
BC112410
AK078290
AK082624
AK082624
AK082624
AK005784
AK005784
AK005784
Syt3
Lrrc4b
Mybpc2
Spib
Pold1
AK184603
Zfp473
Zfp473
Zfp473
Zfp473
Vrk3
Zfp473
Zfp473
Zfp473
Zfp473
Vrk3
Atf5
Atf5
Nup62
Il4i1
Il4i1
Il4i1
Prr12
Prrg2
Prrg2
Nosip
AK219239
Rps11
NR_000004
Rpl13a
Rpl13a
NR_000003
NR_001277
Flt3l
Flt3l
Flt3l
Pih1d1
Pih1d1
Cd37
Cd37
Cd37
Cd37
Snrp70
Snrp70
Kcna7
Ntf5
Snrp70
Snrp70
Kcna7
Ntf5
Lhb
Bax
Bax
Dhdh
Tulp2
Tulp2
Tulp2
Tulp2
Nucb1
Nucb1
Nucb1
Tulp2
Tulp2
Tulp2
Tulp2
Plekha4
Plekha4
Nucb1
Nucb1
Nucb1
Myd116
Myd116
Plekha4
Plekha4
Plekha4
Nucb1
Nucb1
Nucb1
Myd116
Myd116
Plekha4
Plekha4
Plekha4
Nucb1
Nucb1
Nucb1
Myd116
Myd116
Plekha4
Plekha4
Plekha4
Sec1
Car11
Dbp
Rpl18
EG545963
Lmtk3
Lmtk3
Grin2d
Kdelr1
Erd2.1
Sergef
Spty2d1
Spty2d1
Dbx1
Gas2
Gas2
Gas2
Gas2

Herc2
rjs
Herc2
rjs
Gabrg3
Gabrg3
Gabrb3
Gabrb3
Snrpn
6030441H18Rik
6030441H18Rik
Mcee
Adamts17
Adamts17
Lrrc28
Lrrc28
Lrrc28
Lrrc28
Ttc23
Ttc23
AK049871
Nr2f2
Nr2f2
AK045070
BC070425
Nr2f2
Nr2f2
AK045070
BC070425
DQ694834
DQ703064
DQ720410
DQ716740
DQ542042
DQ707380
DQ568730
DQ686988
DQ551845
DQ564514
DQ555568
DQ693555
AK043101
Rgma
Rgma
Rgma
Rgma
Chd2
Chd2
Chd2
Klhl25
Klhl25
Klhl25
Klhl25
Klhl25
Klhl25
Ntrk3
Ntrk3
Ntrk3
Ntrk3
Ntrk3
Ntrk3
Isg20l1
Isg20l1
mmu-mir-7a-2
Rlbp1
Rlbp1
BC025462
Fanci
Fanci
Polg
Pol gamma
AK032343
AK047441
mmu-mir-9-3
Plin
Plin
Plin
Plin
Pex11a
EG626359
EG626359
Zfp710
Znf710
Zfp710
Zfp710
Znf710
Zfp710
Sema4b
Sema4b
Sema4b
mKIAA1745
Cib1
D330012F22Rik
Ttll13
Ttll13
Ttll13
Ttll13
Iqgap1
AK006397
Iqgap1
Iqgap1
Iqgap1
Iqgap1
Iqgap1
Iqgap1
Iqgap1
Wdr73
Wdr73
Wdr73
Sec11a
sid2895
Fsd2
Whdc1
mKIAA1971
Fsd2
Whdc1
mKIAA1971
Hdgfrp3
Hdgfrp3
Hdgfrp3
Eftud1
Eftud1
Eftud1
Eftud1
AK076849
Rkhd3
AK076849
Rkhd3
Mesdc1
Mesdc2
Rab38
Dlg2
Dlg2
Pcf11
Pcf11
mKIAA0824
Pcf11
4632434I11Rik
Prcp
Prcp
Alg8
1810020D17Rik
Rsf1
Rsf1
Phca
2210018M11Rik
2210018M11Rik
2210018M11Rik
Wnt11

Wnt11
Wnt11
Uvrag
Uvrag
Uvrag
Dgat2
Gdpd5
Neu3
Pold3
Pgm2l1
Pgm2l1
Pgm2l1
Pgm2l1
Pgm2l1
Pgm2l1
Ppme1
AU020772
AU020772
AU020772
Ppme1
AU020772
AU020772
AU020772
Ppme1
AU020772
AU020772
AU020772
AK040808
Ucp2
AK171117
Chchd8
Mrpl48
Mrpl48
Mrpl48
Rab6
B930006L02Rik
B930006L02Rik
Arhgef17
mKIAA0769
Fchsd2
Atg16l2
Atg16l2
Stard10
Stard10
Stard10
Inppl1
Inppl1
Inppl1
Inppl1
AK148373
Il18bp
Il18bp
Rnf121
Rnf121
Rnf121
Rnf121
Trpc2
Trp2
Trpc2
Trpc2
Trpc2
Rnf121
Rnf121
Trpc2
Trp2
Trpc2
Trpc2
Trpc2
Rrm1
Rrm1
Trim21
Olfr550
AK136817
Mrpl17
Mrpl17
Nrip3
Nrip3
Rab6ip1
AK005418
Zfp143
mKIAA1766
Sbf2
Sbf2
Sbf2
Adm
Usp47
Usp47
Usp47
Tead1
4632411J06Rik
Mlstd2
Mlstd2
Rras2
Psma1
Psma1
AK016693
AK016630
Pde3b
Sox6
Sox6
Sox6
Sox6
Sox6
Sox6
Sox6
Sox6
BC048672
1110004F10Rik
1110004F10Rik
Exod1
2610020H08Rik
Dcun1d3
Dcun1d3
Lyrm1
Lyrm1
Lyrm1
Dcun1d3
Dcun1d3
Lyrm1
Lyrm1
Lyrm1
4930408O21Rik
AK034016
AK018490
BC030336
4930408O21Rik
AK034016
AK018490
BC030336
Cog7
Cog7
Cacng3
AK140221
Atp2a1
Psmbeta
Sh2b1
Sh2bpsm1
Sh2b1
Sh2bpsm1
Tufm
Tufm
Sult1a1
Sult1a1
Giyd2
Bola2
Mapk3
Mapk3
Gdpd3
Ypel3

Ppp4c
Fam57b
Taok2
mKIAA0881
1110032O16Rik
1110032O16Rik
Taok2
mKIAA0881
1110032O16Rik
1110032O16Rik
Mvp
2900092E17Rik
1500031I19Rik
1500031I19Rik
Cd2bp2
Cd2bp2
Cd2bp2
mKIAA1178
Tbc1d10b
Mylpf
Cd2bp2
Cd2bp2
Cd2bp2
mKIAA1178
Tbc1d10b
Mylpf
Sep-01
Zfp553
Zfp553
Zfp553
Zfp553
Zfp771
Zfp553
2410015N17Rik
Zfp553
Zfp768
Zfp553
Zfp689
AK041042
AK045290
Bcl7c
Bcl7c
Ctf1
mmu-mir-762
Bcl7c
Bcl7c
Ctf1
Fbxl19
Fbxl19
Fbxl19
Fbxl19
Fbxl19
Fbxl19
Zfp668
Zfp646
Zfp646
Prss8
Prss8
Prss36
Prss36
Prss36
Prss36
Fus
Fus
Fus
Fus
Fus
Fus
AK134717
AK079777
Fus
Fus
Fus
AK134717
AK079777
Fus
Fus
Fus
AK134717
AK079777
9130023H24Rik
Tgfb1i1
Tgfb1i1
Tgfb1i1
Slc5a2
Rgs10
Tial1
Fgfr2
Fgfr2
Fgfr2
Fgfr2
Tacc2
Tacc2
Tacc2
Tacc2
Plekha1
Plekha1
Plekha1
Plekha1
Cuzd1
1700007K09Rik
1700063I17Rik
1700063I17Rik
Hmx2
Bub3
Hmx2
Bub3
Hmx2
Bub3
4631426J05Rik
4631426J05Rik
4631426J05Rik
4631426J05Rik
Oat
2010208K18Rik
C430003P19Rik
C430003P19Rik
C430003P19Rik
mKIAA0157
AK158854
2010208K18Rik
C430003P19Rik
C430003P19Rik
C430003P19Rik
mKIAA0157
AK158854
AK005115
Zranb1
Zranb1
Ctbp2
Ebf3
Ebf3
Tcerg1l
Lrrc27
Pwwp2
Lrrc27
Pwwp2
Pwwp2
Msx3
Tmem16j
Tmem16j
Ptdss2
Ptdss2
Tmem16j
Tmem16j
Ptdss2
Ptdss2
Rnh1
Rnh1

Rnh1
Chid1
2700078K21Rik
Mob2
2700078K21Rik
Mob2
Igf2
Igf2
NR_002855
mmu-mir-483
Phlda2
Cars
Shank2
Ccnd1
AK007416
Tpcn2
Arhgef18
Arhgef18
AK019114
Trappc5
AK019114
Trappc5
B130050I23Rik
B130050I23Rik
B130050I23Rik
B130050I23Rik
B130050I23Rik
Lrrc8e
Map2k7
Map2k7
MKK7
Map2k7
Map2k7
Snapc2
BC003267
AK082314
Efnb2
AK007249
Efnb2
AK007249
Tmem28
Rab20
BC030398
0710008K08Rik
0710008K08Rik
Rab20
BC030398
0710008K08Rik
0710008K08Rik
Ankrd10
Ankrd10
Arhgef7
Arhgef7
Arhgef7
EG434280
Lamp1
Dlgap2
Dlgap2
6820431F20Rik
6820431F20Rik
6820431F20Rik
LOC436177
5330429B09Rik
Ap3m2
Ap3m2
Ap3m2
Ap3m2
Ap3m2
Ap3m2
Ap3m2
Ap3m2
Ap3m2
Ap3m2
Ap3m2
Ap3m2
Ank1
Ank1
Agpat6
Tsarg7
Agpat6
Tsarg7
Sfrp1
Tacc1
Tacc1
Tacc1
Fgfr1
Fgfr1
Rab11fip1
AK040272
Rab11fip1
Got1l1
Got1l1
Rbm13
Fut10
Fut10
Nrg1
Nrg1
Nrg1
Nrg1
Nrg1
Nrg1
Ppp2cb
Rbpms
Rbpms
Dusp4
Dusp4
Tnks
Cldn23
AK076961
Sgcz
Tusc3
Tusc3
Fat1
Fat1
Fat1
Helt
Helt
Helt
Ing2
AK083994
AK029624
AK032387
Vegfc
BC088983
Fbxo8
Fbxo8
Sap30
AK141659
AK019137
2700029M09Rik
Clcn3
Clcn3
CLCN3
CLCN3
CLCN3
Clcn3
CLCN3
CLCN3
Nek1
Nek1
Nek1
Nek1
Clcn3
CLCN3
CLCN3
Nek1
Nek1
Nek1
Nek1

1810029B16Rik
Psd3
Psd3
Psd3
Slc18a1
Slc18a1
Slc18a1
Atp6v1b2
Atp6v1b2
Atp6v1b2
AK203140
AK076396
Gatad2a
mKIAA0892
9130404D08Rik
9130404D08Rik
Sf4
Sf4
Sf4
Hapln4
Rfxank
Mef2b
Mef2b
Tmem161a
Armc6
Armc6
Sfrs14
Sfrs14
Sfrs14
Sfrs14
Sfrs14
Comp
Klhl26
Klhl26
Klhl26
Crlf1
Klhl26
Klhl26
Klhl26
Tmem59l
Crlf1
CRLM3
Klhl26
Klhl26
Klhl26
Tmem59l
Crlf1
CRLM3
Klhl26
Klhl26
Klhl26
Tmem59l
Crlf1
CRLM3
Klhl26
Klhl26
Klhl26
Tmem59l
Crlf1
CRLM3
Klhl26
Klhl26
Klhl26
Tmem59l
Crlf1
CRLM3
Ssbp4
Ssbp4
Lrrc25
Ssbp4
Ssbp4
Lrrc25
Ssbp4
Ssbp4
Lrrc25
Jund1
Jund1
AK010542
Kcnn1
Kcnn1
Kcnn1
2410004L22Rik
Myo9b
Myo9b
Myo9b
Myo9b
Mrpl34
1500034J01Rik
Unc13a
Unc13a
Unc13a
Jak3
Jak3
Jak3
Jak3
Jak3
Insl3
Zfp617
s11-6
Tpm4
Tpm4
Rab8A
Rab8a
Nwd1
Nwd1
Nwd1
Nwd1
Nwd1
Large
Hmox1
Mcm5
Mcm5
Mcm5
Mcm5
AK162930
Nr3c2
Rbmxrt
Rbmxrt
P7
Slc10a7
Slc10a7
D630040G17Rik
D630040G17Rik
D630040G17Rik
D630040G17Rik
D630040G17Rik
D630040G17Rik
D630040G17Rik
D630040G17Rik
D630040G17Rik
Smad1
Smad1
Otud4
Otud4
Hhip
Gab1
Pkn1
Pkn1
Ddx39
AK157878
Asf1b
Prkaca
AK008705
4432412L15Rik
mmu-mir-1199
Rfx1
Rfx1
Rfx1
mmu-mir-709

Cacna1a
BC048761
Cacna1a
AK037841
Ier2
AK037841
Ier2
AK037841
Ier2
AK037841
Nfix
Nfix
AK168184
Nfix
Nfix
Nfix
Nfix
Dand5
SP1
Gadd45gip1
Rad23a
Rad23a
Rad23a
AK007237
SP1
Rad23a
Rad23a
Rad23a
Calr
AK007237
Farsa
Farsla
Farsa
Mast1
Mast1
Mast1
Rtbdn
Rtbdn
Rtbnd
Rnaseh2a
Rnaseh2a
Prdx2
Prdx2
Rnaseh2a
Rnaseh2a
Prdx2
Prdx2
Rnaseh2a
Rnaseh2a
Prdx2
Prdx2
Rnaseh2a
Rnaseh2a
Prdx2
AK006748
Junb
Hook2
Hook2
Rnaseh2a
Rnaseh2a
Prdx2
AK006748
Junb
Hook2
Hook2
Rnaseh2a
Rnaseh2a
AK006748
Junb
Hook2
Hook2
Junb
Hook2
Hook2
Vps35
Orc6l
Orc6
Nkd1
Cyld
Cyld
Sall1
Sall1
Chd9
Nudt21
mKIAA1612
Ogfod1
Mt2
Mt1
Mt1
Mt1
Mt1
Cpne2
Cpne2
2310065K24Rik
2310065K24Rik
Rspry1
Rspry1
Rspry1
2310065K24Rik
2310065K24Rik
Rspry1
Rspry1
Rspry1
Gtl3
Gtl3
mKIAA1180
Ndrg4
Setd6
Setd6
Setd6
mmu-mir-1186b
Cdh8
Cdh8
Cdh8
A330008L17Rik
AK042807
Cdh5
Tk2
Cklf
Cklf
CKLF6
Cklf
Cmtm4
Cbfb
Lrrc29
Lrrc29
Hspc171
mmu-mir-328
Agrp
2310066E14Rik
mmu-mir-1966
Agrp
AK086852
AK080383
Ctcf
Ctrl
Psmb10
Lcat
Slc12a4
Slc12a4
AK139607
Has3
Has3
Has3
Has3
Cog8
AK209600
Cog8
Nip7

Nfat5
Nfat5
Nfat5
Nfat5
Nfat5
Nfat5
Nfat5
Nfat5
Psmd7
AK158379
Zfhx3
Zfhx3
mKIAA0174
2400003C14Rik
2400003C14Rik
4930566A11Rik
4930566A11Rik
4930566A11Rik
4930566A11Rik
Ap1g1
Ap1g1
Ddx19b
Ddx19b
Aars
Ddx19b
Ddx19b
Aars
Mrcl
AK020517
AK053982
AK020517
AK053982
Glg1
Rfwd3
Rfwd3
Fa2h
Fa2h
Wdr59
Znrf1
Znrf1
Wdr59
Znrf1
Znrf1
Bcar1
Bcar1
Bcar1
p130Cas
Bcar1
Bcar1
p130Cas
Tmem170
Tmem170
4932417I16Rik
Gabarapl2
Adat1
Adat1
Kars
Terf2ip
Terf2ip
Adat1
Adat1
Kars
Terf2ip
Terf2ip
Adat1
Adat1
Kars
Terf2ip
Terf2ip
4632415K11Rik
mKIAA1609
Cotl1
Gse1
Gse1
Gse1
Gse1
Gse1
Mthfsd
Mthfsd
Foxc2
Mthfsd
Mthfsd
Foxc2
Foxl1
Mthfsd
Mthfsd
Foxc2
Foxl1
Zfpm1
Trhr2
5830416A07Rik
5830416A07Rik
Cbfa2t3h
Cbfa2t3h
Ankrd11
Ankrd11
Fanca
Fanca
Fanca
Fanca
Fanca
Spire2
Fanca
Fanca
Fanca
Fanca
Spire2
Spire2
Tubb3
AK193394
Afg3l1
Afg3l1
Dbndd1
Dbndd1
Gas8
Gas8
Rhou
Taf5l
Taf5l
AK122209
mmu-mir-1967
Ttc13
Ttc13
Ttc13
Arv1
Arv1
Disc1
Disc1
Kcnk1
1810063B05Rik
Pard3
Par3
Pard3
Pard3
Pard3
Par3
Pard3
Pard3
Nrp1
Nrp1
Nrp1
Nrp1
Itgb1
Ccdc7
8430410K20Rik
8430410K20Rik
Tmem123
Tmem123
Birc2

Yap1
Yap1
Mtmr2
Mtmr2
Cep57
Cep57
Cep57
Cep57
2810485I05Rik
Fam76b
Cep57
Cep57
Cep57
Cep57
2810485I05Rik
Fam76b
Amotl1
Amotl1
Amotl1
Amotl1
Panx1
mKIAA4237
Zfp426
Zfp426
Zfp426
mKIAA4237
Zfp426
Zfp426
Zfp426
mKIAA4237
Zfp426
Zfp426
Zfp426
mKIAA4237
Zfp426
Zfp426
Zfp426
Fbxl12
Fbxl12
Fbxl12
Ubl5
Pin1
Olfm2
Olfm2
Dnmt1
Edg5
Edg5
MRPL4
Mrpl4
Edg5
MRPL4
Mrpl4
MRPL4
Mrpl4
H1f0
Icam1
H1f0
Icam1
Icam4
Icam4
Icam5
Icam1
Icam4
Icam4
Icam5
Cdc37
Pde4a
Pde4a
Pde4a
Pde4a
Keap1
Keap1
Keap1
Keap1
Keap1
Keap1
Keap1
Keap1
Edg8
Ilf3
Ilf3
Ilf3
Rab3d
BC010787
2510048L02Rik
2510048L02Rik
2510048L02Rik
BC018242
BC018242
Elavl3
Elavl3
Elavl3
Zfp653
AK154427
AK016276
Acp5
Acp5
Acp5
Pigyl
9530015I07Rik
Bmper
Tbx20
Tbx20
Tbx20
Sep-07
mKIAA4020
Glb1l3
Ncapd3
Ncapd3
mKIAA0056
Hnt
Hnt
Hnt
Hnt
Barx2
Kcnj1
Ets1
St3gal4
AK019824
Cdon
Cdon
Stt3a
AK018988
Pknox2
Pknox2
PREP2
Pknox2
Pknox2
PREP2
Slc37a2
AF060570
Vsig2
Nrgn
Nrgn
Zfp202
Znf202
Gramd1b
Gramd1b
AK089373
Hspa8
Arhgef12
Arhgef12
Arhgef12
Arhgef12
Pou2f3
Pvrl1
Pvrl1
AK087650
Thy1

Rnf26
Rnf26
Mcam
Tmem24
Tmem24
Dpagt1
Dpagt1
H2afx
Tmem24
Tmem24
Dpagt1
Dpagt1
H2afx
Hmbs
Hmbs
Vps11
Vps11
Vps11
Hyou1
Hyou1
Trappc4
Trappc4
Rps25
Rps25
AK078316
Bcl9l
Bcl9l
AK078316
Bcl9l
Bcl9l
Phldb1
Phldb1
Phldb1
Phldb1
Arcn1
Arcn1
1500035H01Rik
1500035H01Rik
Mll1
Mll1
Mpzl2
Mpzl3
Mpzl3
Cep164
Cep164
Bace
Bace1
Bace
Cep164
Cep164
Bace
Bace1
Bace
Pafah1b2
AK011326
BC033915
Zfp259
Bud13
Bud13
sgigsf
Cadm1
Cadm1
Cadm1
sgigsf
Cadm1
Cadm1
Cadm1
sgigsf
Cadm1
Cadm1
Cadm1
sgigsf
Cadm1
Cadm1
Cadm1
Rexo2
Rexo2
EG434402
Zbtb16
Zbtb16
Zbtb16
Tmprss5
Drd2
Dlat
Dixdc1
Dixdc1
Dixdc1
Dixdc1
Dixdc1
2310030G06Rik
Cryab
Cryab
Cryab
Ppp2r1b
Ppp2r1b
4833427G06Rik
Btg4
if5
Btg4
4833427G06Rik
Btg4
if5
Btg4
mKIAA1726
AI593442
AI593442
Wdr61
Wdr61
C630028N24Rik
C630028N24Rik
AY074887
Psma4
Etfa
Isl2
Sgk269
C230081A13Rik
Hmg20a
Hmg20a
Hmg20a
Hmg20a
Hmg20a
Sgk269
C230081A13Rik
Hmg20a
Hmg20a
Hmg20a
Hmg20a
Hmg20a
Sh3px3
Imp3
Sh3px3
Imp3
Snupn
Snupn
Ptpn9
AK143982
AK162906
AK012238
Sin3a
Sin3a
Sin3a
AK143982
AK162906
AK012238
Sin3a
Sin3a
Sin3a
AK077427
AK010793

Man2c1
Man2c1
Man2c1
Man2c1
AK077427
AK010793
Man2c1
Man2c1
Man2c1
Man2c1
2310046O06Rik
2310046O06Rik
Mpi
Scamp2
Arid3b
Arid3b
Arid3b
Arid3b
Ccdc33
4930535E21Rik
Stra6
4930535E21Rik
4930535E21Rik
Stra6
Stra6
Ccdc33
4930535E21Rik
Stra6
4930535E21Rik
4930535E21Rik
Stra6
Stra6
Pml
Pml
Pml
Stoml1
Stoml1
Nptn
Nptn
Nptn
Hcn4
Adpgk
Adpgk
Adpgk
Adpgk
Senp8
AK048681
AK168746
Nr2e3
Pnr
Pnr
Kif23
Kif23
Kif23
Kif23
Glce
AK043361
Anp32a
AK028224
Anp32a
AK028224
Anp32a
AK028224
Fem1b
Cln6
Pias1
Lbxcor1
Lbxcor1
Lbxcor1
Zwilch
Rpl4
Snapc5
Map2k1
Tipin
Dis3l
Dis3l
Dis3l
AV340375
Megf11
Megf11
Megf11
Ptplad1
Dpp8
Dpp8
Ptplad1
Dpp8
Dpp8
Nope
Punc
Punc
Punc
Rbpms2
Rbpms2
Rbpms2
Rbpms2
Zfp609
Trip4
Trip4
2810417H13Rik
Trip4
Trip4
2810417H13Rik
Herc1
Herc1
Herc1
Herc1
Fbxl22
Rab8b
Rab8b
Perq1
Tpm1
Tpm1
Tpm1
Rora
Rora
Foxb1
AK045913
Foxb1
AK045913
Myo1e
Myo1e
Myo1e
Met
Met
Met
Sltm
Sltm
B230380D07Rik
B230380D07Rik
B230380D07Rik
Adam10
Grinl1a
Grinl1a
Tcf12
Tcf12
Tcf12
Rfxdc2
Rfxdc2
Prtg
Dyx1c1
Onecut1
Onecut1
Onecut1
BC031353
BC031353
BC031353
Gnb5
AK134928
Gnb5

Gnb5
Leo1
Elovl5
Fbxo9
Fbxo9
Fbxo9
Fbxo9
Ick
Ick
Ick
Gsta4
Cd109
Cd109
Cox7a2
Phip
Phip
Phip
Phip
Phip
Phip
Phip
Phip
Phip
Phip
Lca5
Lca5
Lca5
AK076672
Ttk
Ttk
mKIAA1417
Ibtk
mKIAA1417
Ibtk
Ibtk
mKIAA1417
Ibtk
AK162386
Tpbg
Tpbg
Tpbg
Tbx18
Snx14
Snx14
Snx14
Rasgrf1
Rasgrf1
Rasgrf1
Adamts7
Adamts7
Adamts7
Adamts7
Zic4
Zic4
Zic4
Zic1
Zic1
Zic4
Zic4
Zic4
Zic4
Zic4
Zic1
Zic1
Zic4
Zic4
Zic4
Zic4
Zic4
Zic1
Zic1
Zic4
Zic4
Zic4
Zic4
Zic4
Zic1
Zic1
Zic4
Zic4
Zic4
Zic4
Zic4
Zic1
Zic1
Zic4
Zic4
Zic4
Zic4
Zic4
Zic1
Zic1
Zic4
Zic4
Zic4
Plscr1
Plscr1
2610101N10Rik
2610101N10Rik
2610101N10Rik
2610101N10Rik
AI427122
AK006889
1700065D16Rik
AK141900
Atr
AK142244
Tfdp2
Tfdp2
Tfdp2
Slc25a36
NR_003248
Foxl2
Faim
Faim
Cep70
Cep70
Cep70
Cep70
Stag1
Stag1
Stag1
Stag1
Stag1
Stag1
3222402P14Rik
3222402P14Rik
3222402P14Rik
Ephb1
Ephb1
Ephb1
Ephb1
Cep63
Cep63
D9Mgc48e
Anapc13
Anapc13
AK147911
Nek11
Nek11
Nek11
Aste1
Aste1
Aste1
Atp2c1
Atp2c1
Atp2c1
AK051019
CS444466

mmu-mir-135a-1
Alas1
Alas1
Dusp7
Rpl29
Rpl29
Rpl29
Rpl29
Abhd14b
Rbm15b
Armet
Armet
Mapkapk3
Mapkapk3
Mapkapk3
Mapkapk3
Cish
Tmem115
Tusc4
Zmynd10
Rassf1
Cyb561d2
Cyb561d2
Rassf1
Tusc2
Hyal2
Hyal2
AK044848
Hyal2
Hyal2
Hyal1
Hyal1
Hyal1
Nat6
Nat6
Hyal3
Ifrd2
Rbm6
D330022A01Rik
Ube1l
D330022A01Rik
6230427J02Rik
D330022A01Rik
1700021K14Rik
Ihpk1
AK197603
Ihpk1
Ihpk1
Ip6k1
Dag1
Dag1
Dag1
Klhdc8b
Klhdc8b
Lamb2
Usp19
Usp19
Usp19
Usp19
Usp19
Qars
Qars
Qars
Qrich1
Qrich1
Qrich1
Qars
Qars
Qars
Qrich1
Qrich1
Qrich1
Impdh2
4733401H18Rik
Dalrd3
mmu-mir-191
mmu-mir-425
Prkar2a
Scotin
Scotin
Scotin
Scotin
6620401K05Rik
6620401K05Rik
Ccdc72
Ccdc51
Ccdc51
Plxnb1
6620401K05Rik
6620401K05Rik
Ccdc72
Ccdc51
Ccdc51
Plxnb1
Ccdc72
Plxnb1
Ccdc72
Plxnb1
Cdc25a
Cdc25a
Cdc25a
Cdc25a
Mtap4
Mtap4
Mtap4
Mtap4
Mtap4
Mtap4
Klhl18
Kif9
Kif9
Kif9
Kif9
Ccdc12
Ccdc12
Ccdc12
Tessp2
Rtp3
Mlh1
Mlh1
Lba1
Ubp1
Trim71
Cnot10
Dync1li1
Dync1li1
Cmtm7
Cmtm7
Lnv
Cmtm8
AK161079
Stt3b
AK013480
Azi2
Azi2
AZ2
Eomes
Eomes
Eomes
Oxsr1
Acaa1a
Slc25a38
Slc25a38
Rpsa
Rpsa
NR_002902
Slc25a38
Slc25a38
Rpsa

Rpsa
NR_002902
Slc25a38
Slc25a38
Rpsa
Rpsa
NR_002902
Trak1
Trak1
Trak1
limd1
Limd1
limd1
Limd1
limd1
Sacm1l
Xtrp3s1
Ccr9
AK017500
Cul4b
Cul4b
Cul4b
Mcts1
Mcts1
Mcts1
C1galt1c1
Birc4
Birc4
Birc4
mKIAA1823
Phf6
Phf6
Spin4
Mllt7
Foxo4
Med12
Rps4x
Rps4x
AK085125
AK035525
BC027389
BC076616
Cnbp2
Abcb7
BC080769
AK043766
BC019699
AK017459
AK157237
BC055463
AK080296
AK164650
AK135254
2610029G23Rik
2610029G23Rik
Gm784
Tceal8
Tceal8
Col4a6
Col4a5
Col4a5
Jarid1c
Jarid1c
Ubqln2
Reps2
Mid1
Mid1
Mid1
Mid1
Mid1
Mid1
Mid1
Mid1
Mid1
Mid1
Mid1
Mid1
Mid1
Mid1
Mid1
Mid1
Mid1
Mid1
Mid1
Mid1
Mid1
AK149987
AK019053
Mid1
Mid1
AK149987
AK019053
Mid1
Mid1
AK149987
AK019053

pan Blimp1 target genes	pan DP lineage signature genes	intersection pan Blimp1 target genes with pan DP lineage signature genes
0610007P14Rik	0610005C13Rik	0610037L13Rik
0610009B22Rik	0610010B08Rik///Gm14295///Gm1430[illegible]	1100001G20Rik
0610009O20Rik	0610010B08Rik///Gm14308///Gm1432[illegible]	1110004E09Rik
0610010E21Rik	0610037L13Rik	1190003J15Rik
0610010K14Rik	0610040J01Rik	1700001C19Rik
0610010O12Rik	0910001A06Rik	1700021K14Rik
0610013E23Rik	1100001G20Rik	1700120B22Rik
0610025P10Rik	1100001H23Rik /// LOC100045163	1810032O08Rik
0610037D15Rik	1110003F05Rik	2210407C18Rik
0610037L13Rik	1110004E09Rik	2310067B10Rik
0610038F07Rik	1110006O24Rik	2410015M20Rik
0710008K08Rik	1110008P14Rik	2510009E07Rik
1100001G20Rik	1110020A10Rik	2610001J05Rik
1110001A07Rik	1110035H17Rik	2610507B11Rik
1110002N22Rik	1110037F02Rik	2810025M15Rik
1110003E01Rik	1110054M08Rik	2810417H13Rik
1110004E09Rik	1110057K04Rik	2810439F02Rik
1110004F10Rik	1110059E24Rik	2810474O19Rik
1110005A23Rik	1110064A23Rik	3110057O12Rik
1110007C09Rik	1190002N15Rik	4930427A07Rik
1110007L15Rik	1190003J15Rik	4930429B21Rik
1110008J03Rik	1190005I06Rik	4930430E16Rik
1110008L16Rik	1200016E24Rik	4930570C03Rik
1110012D08Rik	1300017J02Rik	4932438H23Rik
1110012J17Rik	1500002F19Rik	4933428G20Rik
1110014N23Rik	1500015O10Rik	6030458C11Rik
1110019K23Rik	1600014C10Rik	9030409G11Rik
1110020G09Rik	1600015I10Rik	A130010J15Rik
1110029E03Rik	1600029D21Rik	AI317395
1110031B06Rik	1700001C19Rik	AI661453
1110031I02Rik	1700001F09Rik///Gm10375///Gm1037[illegible]	AK009121
1110032O16Rik	1700001G17Rik	AU040320
1110034B05Rik	1700001O22Rik	Aars
1110036O03Rik	1700008F21Rik///Zfp821	Aatk
1110038D17Rik	1700010I14Rik	Abat
1110038F14Rik	1700011A15Rik	Abca1
1110049F12Rik	1700012L04Rik///Gm14474///Gm14475	Abca8a
1110051M20Rik	1700017B05Rik	Abcb7
1110054O05Rik	1700018B24Rik	Abcc10
1190003J15Rik	1700019D03Rik	Abcc2
1190005F20Rik	1700019G17Rik	Abcc5
1200009I06Rik	1700019N19Rik	Abcd4
1200011O22Rik	1700020D05Rik	Abcf1
1200014J11Rik	1700021K14Rik	Abcf2
1200015F23Rik	1700028P14Rik	Abcf3
1200016B10Rik	1700029F12Rik	Abhd14b
1300007F04Rik	1700029H14Rik	Abhd3
1300010M03Rik	1700029P11Rik	Acbd3
1300018I05Rik	1700037H04Rik	Ache
1500001A10Rik	1700065O13Rik	Acin1
1500003O03Rik	1700067P10Rik	Aco2
1500005A01Rik	1700071K01Rik///Phb	Acot11
1500005I02Rik	1700073E17Rik	Acp2
1500010J02Rik	1700074P13Rik	Acss2
1500019G21Rik	1700080E11Rik	Acvrl1
1500031I19Rik	1700082M22Rik	Adam10
1500032L24Rik	1700088E04Rik	Adam15
1500034J01Rik	1700091H14Rik///Gm8068	Adam5
1500035H01Rik	1700094J05Rik	Adamts10
1500035N22Rik	1700097N02Rik	Adamts20
1520401A03Rik	1700102P08Rik	Adamts4
1600002K03Rik	1700104A03Rik	Adamts7
1600012H06Rik	1700120B22Rik	Adamtsl4
1600029I14Rik	1700123K08Rik	Adamtsl5
1700001C19Rik	1810009A15Rik///LOC102308570	Adat1
1700003E16Rik	1810009J06Rik///Gm2663	Adck3
1700003F12Rik	1810009N02Rik	Adcy2
1700006E09Rik	1810011O10Rik	Adcy8
1700007I06Rik	1810019J16Rik	Adipoq
1700007K09Rik	1810032O08Rik	Adm
1700009P17Rik	1810041L15Rik	Adora1
1700010D01Rik	1810043G02Rik	Adpgk
1700010K24Rik	1810044D09Rik	Adprh
1700011E24Rik	1810055G02Rik	Adrbk2
1700011F14Rik	2010001M06Rik	Aftph
1700015F17Rik	2010012O05Rik	Agap2
1700019E08Rik	2010106E10Rik	Agbl5
1700019E19Rik	2010204K13Rik	Agpat6
1700020C11Rik	2010205A11Rik /// EG628498 /// ENSM[illegible]	Agrp
1700021C14Rik	2200001I15Rik	Ahctf1
1700021K14Rik	2210010C04Rik	Ahdc1
1700022C21Rik	2210016F16Rik	Ahi1
1700023F06Rik	2210016L21Rik	Ahnak
1700024G10Rik	2210023G05Rik	Aip
1700024N20Rik	2210407C18Rik	Aire
1700026D08Rik	2210409E12Rik	Akap12
1700027J05Rik	2210418O10Rik	Akap2
1700029F09Rik	2310005E10Rik	Akap9
1700029I15Rik	2310010J17Rik	Akna
1700034J04Rik	2310010M24Rik	Akt1
1700049G17Rik	2310014G06Rik	Alas1
1700052K11Rik	2310031A07Rik	Alcam
1700061J05Rik	2310034C09Rik	Aldh18a1
1700063I17Rik	2310038H17Rik	Alg3
1700065D16Rik	2310039H08Rik	Alk
1700081D17Rik	2310057J18Rik///2310065H11Rik	Alox12
1700081L11Rik	2310061J03Rik	Alpl
1700094C10Rik	2310067B10Rik	Alppl2
1700101E01Rik	2310067E19Rik	Ambra1
1700106N22Rik	2310075K07Rik	Amh
1700110K17Rik	2410015M20Rik	Amn
1700113I22Rik	2410025L10Rik	Amot
1700120B22Rik	2410133F24Rik///Man2c1os	Ampd2
1700124K17Rik	2510009E07Rik	Anapc7
1810010H24Rik	2610001J05Rik	Angptl4
1810020D17Rik	2610002J02Rik	Ank1
1810020G14Rik	2610002M06Rik///Chmp1b	Ankhd1
1810029B16Rik	2610005L07Rik	Ankrd17
1810031K17Rik	2610018G03Rik	Ankrd24
1810032O08Rik	2610019E17Rik///Rab26os	Ankrd33
1810034K20Rik	2610034M16Rik	Ankrd36
1810044A24Rik	2610042L04Rik///4930555G01Rik///D8[illegible]	Anp32a
1810046J19Rik	2610201A13Rik	Anp32e
1810063B05Rik	2610301B20Rik	Anxa8
1810064F22Rik	2610301F02Rik	Anxa9
1810065E05Rik	2610507B11Rik	Ap1g1
1810073N04Rik	2610524H06Rik	Ap1s3
2010001M09Rik	2610528A11Rik	Ap2b1
2010106G01Rik	2700046A07Rik	Ap2m1
2010109A12Rik	2700079J08Rik	Ap3m2
2010109K11Rik	2700089E24Rik	Ap4m1
2010111I01Rik	2810002D19Rik	Ap4s1
2010208K18Rik	2810025M15Rik	Apip
2010209O12Rik	2810040C05Rik	Apobec1
2210018M11Rik	2810408B13Rik	Apoc1
2210038L17Rik	2810408M09Rik	Apoc2
2210407C18Rik	2810408M09Rik///Trp53rk	Apoc4
2210408F21Rik	2810417H13Rik	Apod
2210411K11Rik	2810428I15Rik	App
2300002D11Rik	2810439F02Rik	Aptx
2300003K06Rik	2810449G22Rik	Aqp8
2310003F16Rik	2810459M11Rik	Arf3
2310003H01Rik	2810474O19Rik	Arf4
2310004N24Rik	2900054C01Rik	Arf6
2310005N01Rik	2900076A13Rik	Arhgap24
2310016E02Rik	2900078E11Rik /// Cadm2	Arhgap29
2310021P13Rik	2900097C17Rik	Arhgap30
2310022A10Rik	3021401C12Rik	Arhgap31
2310026E23Rik	3110007F17Rik	Arhgef16
2310030G06Rik	3110057O12Rik	Arhgef2
2310030N02Rik	3110057O12Rik///Gm2011	Arhgef7
2310033K02Rik	3110070M22Rik	Arid1a
2310035C23Rik	3110079O15Rik	Arid3b
2310043M15Rik	3632451O06Rik	Arid4b
2310044G17Rik	4632413I24Rik	Arid5b
2310046O06Rik	4632415L05Rik	Arl1
2310047D13Rik	4632428N05Rik	Arl2bp
2310047M10Rik	4732474O15Rik	Arl4d

2310061N02Rik	4832441B07Rik	Arl6ip6
2310065K24Rik	4833422C13Rik	Arl8b
2310066E14Rik	4833423E24Rik	Armc1
2310067B10Rik	4833439L19Rik	Armc6
2310079F23Rik	4921505C17Rik	Arnt
2400003C14Rik	4921511H03Rik	Arntl2
2410002M20Rik	4921521F21Rik	Arpc4
2410002O22Rik	4921524J17Rik	Arrdc3
2410003K15Rik	4921530L21Rik	Arsg
2410004F06Rik	4921536K21Rik	Art2a-ps
2410004L22Rik	4922502D21Rik	Art2b
2410015M20Rik	4930413G21Rik	Asah2
2410015N17Rik	4930414L22Rik	Asb13
2410018G20Rik	4930420K17Rik	Ascc2
2410018L13Rik	4930427A07Rik	Ascl1
2410081M15Rik	4930429B21Rik	Asf1a
2410127E18Rik	4930430E16Rik	Aspa
2410141K09Rik	4930432F04Rik	Atf2
2510009E07Rik	4930433N12Rik	Atf5
2510048L02Rik	4930447C04Rik	Atf7
2600001A11Rik	4930449I24Rik///Gm3402///Gm6309///	Atg2a
2600010E01Rik	4930453N24Rik	Atg3
2610001J05Rik	4930470P17Rik	Atn1
2610019A05Rik	4930483J18Rik	Atox1
2610020H08Rik	4930500M09Rik///4930500M09Rik///UI	Atp1a1
2610028H24Rik	4930507D05Rik	Atp1a2
2610029G23Rik	4930524B15Rik	Atp1b1
2610036D13Rik	4930535I16Rik	Atp1b2
2610044O15Rik	4930544G11Rik	Atp2a1
2610101N10Rik	4930549C01Rik	Atp6v1b1
2610109H07Rik	4930550L24Rik	Atp6v1e1
2610307P16Rik	4930562D21Rik	Atp6v1g2
2610507B11Rik	4930570C03Rik	Atp8a1
2610528E23Rik	4930589M24Rik	Atp8b2
2700019D07Rik	4930591A17Rik	Atpif1
2700029M09Rik	4931406G06Rik///4931406G06Rik	Atr
2700038N03Rik	4931406H21Rik	Auh
2700045P11Rik	4931417E11Rik	Aup1
2700049P18Rik	4931432E15Rik	Auts2
2700060E02Rik	4932438H23Rik	Axin1
2700062C07Rik	4932441J04Rik	Axl
2700078E11Rik	4933400A11Rik	Azin1
2700078K21Rik	4933407K13RIK	B230118H07Rik
2810001G20Rik	4933411G06Rik	B230217C12Rik
2810006K23Rik	4933411K20Rik	B3gnt2
2810007J24Rik	4933415F23Rik	B3gnt3
2810008M24Rik	4933417A18Rik	B4galnt2
2810025M15Rik	4933417O08Rik	B4galt3
2810030E01Rik	4933421I07Rik	B9d1
2810403A07Rik	4933424G05Rik	B9d2
2810408A11Rik	4933425L06Rik	BC005624
2810410M20Rik	4933428G20Rik	BC006965
2810417H13Rik	4933429H19Rik	BC018242
2810432D09Rik	4933434I20Rik	BC021614
2810439F02Rik	4933436I01Rik	BC028528
2810451A06Rik	4933439C10Rik	BC031181
2810474O19Rik	5031439G07Rik	BC031353
2810485I05Rik	5033414K04Rik	BC051212
2900006K08Rik	5330433J24Rik	Bace1
2900009I07Rik	5430417L22Rik	Bach2
2900010M23Rik	5430421F17Rik	Bai3
2900073G15Rik	5430421N21Rik	Baiap2
2900092E17Rik	5730408K05Rik///Mir5136	Bak1
3110003A17Rik	5730493B19Rik	Bambi
3110009E18Rik	5830405N20Rik	Barx1
3110031B13Rik	5830411J07Rik	Baz1b
3110049J23Rik	5830428H23Rik	Bbc3
3110057O12Rik	5830468K08Rik	Bbx
3222402P14Rik	6030419C18Rik	Bcan
3230401D17Rik	6030442H21Rik	Bcar1
3300001P08Rik	6030458C11Rik	Bcas3
3300002A11Rik	6330403K07Rik	Bcat1
3425401B19Rik	6330406I15Rik	Bcat2
3930401K13Rik	6330416G13Rik	Bckdhb
3930402G23Rik	6330509M05Rik///Slc9a7	Bcl11a
4121402D02Rik	6430548M08Rik	Bcl2l1
4432412L15Rik	6430550H21Rik	Bcl2l14
4631416L12Rik	6430573F11Rik	Bcl2l2
4631426J05Rik	6430704M03Rik	Bcl3
4631427C17Rik	6720401G13Rik	Bcl6
4632411B12Rik	6720418B01Rik	Bcl7b
4632411J06Rik	6720458F09Rik	Bcl9
4632415K11Rik	6720475J19Rik	Bdkrb1
4632419I22Rik	8430419K02Rik	Bdnf
4632434I11Rik	8430419L09Rik	Bfar
4733401H18Rik	8430420F16Rik	Bin1
4831426I19Rik	9030409G11Rik	Birc6
4833401D15Rik	9030624J02Rik	Bmf
4833420G17Rik	9130004C02Rik	Bmi1
4833427G06Rik	9230009I02Rik	Bmp1
4833436C18Rik	9230115E21Rik	Bmp3
4833442J19Rik	9330159F19Rik	Bmp4
4921526F01Rik	9330182L06Rik	Bnc2
4921528H16Rik	9430015G10Rik	Bnip3
4921530D09Rik	9530002B09Rik	Bola2
4921533L14Rik	9530053H05Rik	Bpil2
4922502B01Rik	9530068E07Rik	Bpnt1
4922505G16Rik	9630023C09Rik	Bptf
4930405O22Rik	9630027E11 /// Grik3	Brap
4930408O21Rik	9630041G16Rik	Brca1
4930415O20Rik	9830134C10Rik	Brd2
4930421P07Rik	A	Brd4
4930427A07Rik	A030004J04Rik	Brd8
4930429B21Rik	A030005K14Rik	Brdt
4930430E16Rik	A130010J15Rik	Brsk1
4930451C15Rik	A230006K03Rik///LOC102633679	Brwd1
4930452B06Rik	A230046K03Rik	Bsg
4930455C21Rik	A230050P20Rik	Btbd2
4930455G09Rik	A2m	Btg4
4930471M09Rik	A330021E22Rik	Btn1a1
4930471M23Rik	A330068G13Rik	Btrc
4930473A06Rik	A330076H08Rik	Bub3
4930504E06Rik	A330076H08Rik,Mir344g	Bud31
4930504H06Rik	A430001F24Rik	Bysl
4930507D10Rik	A430104N18Rik///Mir142	C1galt1c1
4930511I11Rik	A4galt	C1qb
4930513N10Rik	A530016L24Rik	C1qc
4930519B02Rik	A630005I04Rik	C1qtnf6
4930527J03Rik	A630033E08Rik	C3
4930535B03Rik	A630033H20Rik	C87436
4930535E21Rik	A630038E17Rik	Cab39l
4930546H06Rik	A630072M18Rik	Cabp4
4930553M12Rik	A730028G07Rik	Cacna1a
4930555I21Rik	A730034C02///Map2	Cacna1c
4930566A11Rik	A730054J21Rik	Cacna1g
4930570C03Rik	A730082K24Rik	Cacna1h
4930572J05Rik	A830059I20Rik	Cacnb2
4930578M01Rik	A930012O16Rik	Cacnb3
4930578N16Rik	AA407107	Cacng3
4930579G24Rik	AA407331	Cacng4
4931406I20Rik	AA407782	Cadm3
4931417G12Rik	AA408251	Cadps2
4931433A01Rik	AA408954	Calb1
4932412D23Rik	AA409316	Calb2
4932417H02Rik	AA409749	Caln1
4932417I16Rik	AA414903	Calr
4932425I24Rik	AA414993	Calu
4932438H23Rik	AA416453	Camk1
4932442K08Rik	AA511254	Camk2d
4932443I19Rik	AA511261	Camk2n1
4933400C05Rik	AA516738	Camk2n2
4933404M19Rik	AA517023	Cand1
4933407I08Rik	AA517562	Canx
4933413G19Rik	AA517650	Capn3
4933421E11Rik	AA517841	Caprin1
4933422H20Rik	AA517858	Car11
4933424B01Rik	AA517864	Car3
4933425L03Rik	AA522020	Car9
4933426K21Rik	AA536748	Cars

4933427D14Rik	AA545190	Cblb
4933428G20Rik	AA589418	Cblc
4933430I17Rik	AA672641	Cbll1
4933431K23Rik	AA675344	Cbx1
4933432B09Rik	AB041806	Cbx3
4933434I06Rik	AF366264	Cbx5
4933438A12Rik	AI118078	Cbx6
4933439F11Rik	AI317395	Ccdc106
5031425E22Rik	AI413582	Ccdc12
5133400G04Rik	AI450241	Ccdc53
5133401N09Rik	AI503316///Hnrnpu	Ccdc88a
5330429B09Rik	AI504432	Ccdc91
5330431N19Rik	AI506816	Ccdc97
53BP1	AI596198	Ccl22
5730403B10Rik	AI646383	Ccl25
5730409E15Rik	AI661453	Ccnd2
5730410I19Rik	AI662270	Ccnd3
5730437N04Rik	AI844685	Ccnt1
5730442P18Rik	AK009121	Ccr10
5730453I16Rik	AK010638///Gm14820///Gm14820	Ccr9
5730559C18Rik	AK077428	Ccrl2
5730593F17Rik	AK138291	Cct3
5730596K20Rik	AK201917///Vdac1	Cct6b
5830415F09Rik	AK213652	Cd14
5830416A07Rik	ASAP3	Cd24a
5830427D03Rik	AU016916	Cd274
5830434P21Rik	AU018091	Cd2bp2
5830444B04Rik	AU018829///Gm16367///Gm3139///Gm	Cd37
5830483C08Rik	AU020206	Cd47
5930403L14Rik	AU021092	Cd80
5930416I19Rik	AU022245	Cdadc1
5S_rRNA	AU040096	Cdc27
6030408C04Rik	AU040320	Cdc37
6030422M01Rik	AW049604	Cdc42
6030441H18Rik	AW112010	Cdc42ep4
6030445D17Rik	AW121567	Cdc7
6030458C11Rik	AW209491	Cdc73
6030498E09Rik	AW551984	Cdca3
6230409E13Rik	Aacs	Cdca5
6230416J20Rik	Aaed1	Cdh10
6230427J02Rik	Aak1	Cdh18
6330442E10Rik	Aamdc	Cdh22
6330514A18Rik	Aamp	Cdh5
6330548G22Rik	Aanat	Cdh8
6330577E15Rik	Aar2	Cdk5rap3
6430502M16Rik	Aard	Cdkl2
6430527G18Rik	Aars	Cdkn1a
6430598A04Rik	Aass	Cdkn2a
6430701C03Rik	Aatk	Cdr2l
6430706D22Rik	Abat	Cdx2
6620401K05Rik	Abca1	Cecr5
6720456B07Rik	Abca8a	Cela1
6820408C15Rik	Abca9	Cep164
6820431F20Rik	Abcb10	Cep170
7420416P09Rik	Abcb11	Cep55
7530404M11Rik	Abcb1a	Cep57l1
7SK	Abcb1b	Cep70
8430406I07Rik	Abcb4	Cerk
8430410K20Rik	Abcb6	Cfl1
8430415E04Rik	Abcb7	Chchd3
8430416H19Rik	Abcb8	Chd4
8430427H17Rik	Abcb9	Chgb
8430437L04Rik	Abcc10	Chid1
9030025P20Rik	Abcc2	Chka
9030409G11Rik	Abcc3	Chn2
9130017N09Rik	Abcc5	Chrd
9130023H24Rik	Abcc6	Chrna1
9130221H12Rik	Abcc8	Chrnb4
9130404D08Rik	Abcc9	Cic
9130404D14Rik	Abcd1	Cidec
9130409J20Rik	Abcd2	Cirbp
9230105E05Rik	Abcd4	Cish
9230112D13Rik	Abce1	Cklf
9330175E14Rik	Abcf1	Clasp1
9430016H08Rik	Abcf2	Clcf1
9430024E24Rik	Abcf3	Clcn1
9430057O19Rik	Abcg1	Clcn2
9430076C15Rik	Abcg2	Clcn3
9530015I07Rik	Abcg3	Clcn4-2
9530027J09Rik	Abcg4	Clcnka
9530048O09Rik	Abcg5	Clcnkb
9530052E02Rik	Abhd10	Cldn15
9630008K15Rik	Abhd11os	Cldn23
9630033F20Rik	Abhd12	Clec2h
9630041N07Rik	Abhd14a	Clmn
9830169C18Rik	Abhd14b	Clstn1
A130010J15Rik	Abhd15	Clstn3
A130042E20Rik	Abhd16a	Cmtm7
A130092J06Rik	Abhd2	Cmtm8
A230054D04Rik	Abhd3	Cnih3
A230069A22Rik	Abhd8	Cnn2
A230083G16Rik	Abi3bp	Cnnm2
A230097K15Rik	Abl1	Cnot2
A230106M15Rik	Acaa1a///Acaa1b	Cnot4
A230107C01Rik	Acaa1b	Cnot6l
A330008L17Rik	Acacb	Cnp
A430093A21Rik	Acad8	Cntfr
A430106J12Rik	Acadl	Cntn4
A530013C23Rik	Acadsb	Cntnap1
A530020G20Rik	Acan	Col11a2
A630001G21Rik	Acbd3	Col13a1
A630007B06Rik	Acd	Col16a1
A630047E20Rik	Ace	Col17a1
A730008H23Rik	Ace2	Col19a1
A830018L16Rik	Acer2	Col1a1
A830023I12Rik	Ache	Col2a1
A930011G23Rik	Acin1	Col5a2
A930037G23Rik	Ackr1	Commd5
A930038C07Rik	Ackr4	Commd7
AA415398	Acly	Comp
AA536717	Acnat2	Comtd1
AA645442	Aco1	Cops5
AA986860	Aco2	Cops6
AC103388.1	Acot11	Coq3
AC109145.1	Acot3	Coq5
AC111115.1	Acot4	Cotl1
AC113178.1	Acot5	Cox17
AC113291.1	Acot6	Cox8a
AC114613.1	Acox2	Cp
AC116595.1	Acox3	Cpeb3
AC122860.1	Acp1	Cpeb4
AC124613.1	Acp2	Cpne2
AC124745.1	Acpp	Cpne4
AC125186.1	Acr	Crabp2
AC132266.1	Acrbp	Creb1
AC134576.1	Acrv1	Creb3
AC137692.1	Acsf2	Creb5
AC147047.1	Acsl4	Crhr1
AC149605.1	Acsl6	Crtc2
AC157101.1	Acsm2	Cryab
AC157912.1	Acss1	Cryzl1
AC158667.1	Acss2	Csf1
AC163694.1	Acss3	Csnk1a1
AC165159.1	Acta1	Cspp1
AC167197.1	Acta2	Csrp3
AC173478.1	Actb	Ctbp2
ACSS1	Actc1	Ctcf
ACSS2	Actc1 /// LOC100048431	Ctnna1
ACTB	Actg2	Ctnna2
ACTG2	Actn1	Ctrl
ACTL6A	Actn2	Ctsb
ADAMTS13	Actn3	Cuedc1
ADAMTS6	Actr10	Cuedc2
ADCK4	Actr1a	Cul2
ADSS	Actrt1	Cuta
AF060570	Actrt2	Cxcl1
AF380425	Acvr1	Cxcl12
AFF3	Acvr1b	Cygb
AGK	Acvr2b	Cyhr1

AI314180	Acvrl1	Cyp2j13
AI317237	Acy1	Cyp4f16
AI317395	Acy3	Cyp4v3
AI413782	Acyp1	Cyp7b1
AI427122	Acyp2	Cyr61
AI450540	Ada	D030011O10Rik
AI461788	Adad1	D17Wsu92e
AI593442	Adam10	D930048N14Rik
AI606473	Adam11	Dab1
AI661453	Adam12	Dab2
AI842396	Adam15	Dagla
AI846148	Adam18	Dand5
AI848100	Adam19	Dbndd1
AI851790	Adam1a	Dbp
AICDA	Adam1b	Dcakd
AIM2	Adam21	Dck
AK002838	Adam22	Dclk1
AK002860	Adam26a	Dcps
AK002929	Adam28	Dcun1d4
AK003793	Adam3	Ddah1
AK003812	Adam33	Ddah2
AK003841	Adam34	Ddc
AK004434	Adam4	Ddit3
AK004727	Adam5	Ddr1
AK005115	Adam7	Ddx18
AK005418	Adam8	Ddx19b
AK005503	Adamts1	Ddx39
AK005538	Adamts10	Dennd4b
AK005651	Adamts15	Derl1
AK005754	Adamts16	Dgka
AK005757	Adamts20	Dgke
AK005784	Adamts4	Dhdds
AK005839	Adamts5	Dhh
AK005956	Adamts7	Dhrs7b
AK005967	Adamts8	Dhx58
AK006022	Adamtsl4	Dixdc1
AK006051	Adamtsl5	Dlat
AK006104	Adarb2	Dlg2
AK006186	Adat1	Dlg4
AK006239	Adat3///Scamp4	Dlgap4
AK006240	Adck3	Dlst
AK006245	Adck4	Dlx3
AK006343	Adcy2	Dmpk
AK006397	Adcy3	Dmtf1
AK006409	Adcy4	Dnahc2
AK006454	Adcy6	Dnaja1
AK006531	Adcy7	Dnajc12
AK006540	Adcy8	Dnajc2
AK006546	Adcy9	Dnajc27
AK006587	Adcyap1	Dnali1
AK006607	Adcyap1r1	Dnase1l3
AK006748	Add1	Dnmt3a
AK006889	Add2	Dnmt3b
AK006895	Add3	Dock7
AK006942	Adh1	Dok1
AK007191	Adh7	Dok5
AK007237	Adi1	Donson
AK007249	Adig	Dpf3
AK007255	Adipoq	Dpp6
AK007416	Adk	Dqx1
AK007436	Adm	Drd2
AK007898	Adora1	Dsg1a
AK007998	Adora2a	Dst
AK008044	Adpgk	Dtx3
AK008222	Adprh	Dusp10
AK008665	Adprm	Dusp3
AK008705	Adra1a	Dvl2
AK008755	Adra2a	Dyrk3
AK008833	Adrb1	Dyx1c1
AK008852	Adrb2	E2f2
AK008907	Adrb3	E2f6
AK009121	Adrbk2	E2f8
AK009365	Adrm1	Eaf2
AK009597	Adssl1	Ebf2
AK009785	Aebp1	Ebf3
AK009887	Aen	Ebi3
AK010427	Aes	Ece1
AK010542	Afap1	Ece2
AK010793	Afap1l2	Efcab2
AK010870	Aff2	Efna2
AK010894	Aff4	Efna5
AK011222	Afg3l2	Efnb2
AK011283	Afm	Efnb3
AK011326	Afp	Eftud2
AK011568	Aftph	Egfr
AK011682	Agap2	Egr3
AK011693	Agbl3	Ehbp1l1
AK011913	Agbl5	Ehd1
AK012035	Ager	Ehd2
AK012135	Ago2	Eif2ak3
AK012238	Agpat6	Eif2c1
AK012325	Agphd1	Eif2c3
AK012387	Agrn	Eif3h
AK012418	Agrp	Eif4e2
AK012506	Agt	Eif4g3
AK012572	Agtpbp1	Elavl2
AK012891	Agtr1a	Elavl3
AK012955	Agtr1b	Elk4
AK013356	Agtr2	Elovl6
AK013380	Agtrap	Emilin1
AK013480	Agxt	En1
AK013506	Ahctf1	Enah
AK014798	Ahcy	Eno2
AK014955	Ahcyl1	Enoph1
AK014981	Ahdc1	Enpp4
AK015004	Ahi1	Entpd1
AK015092	Ahnak	Eny2
AK015341	Ahr	Eomes
AK015384	Ahrr	Epb4.1l1
AK015392	Ahsa2	Epb4.1l2
AK015395	Ahsg	Epb4.1l4a
AK015458	Aicda	Epha3
AK015526	Aida	Epha5
AK015545	Aif1	Epha7
AK015814	Aif1l	Ephb1
AK015969	Aim1	Ephb2
AK015999	Aim2	Ephb4
AK016007	Aip	Eral1
AK016077	Aipl1	Erbb3
AK016178	Aire	Erc1
AK016185	Airn	Ergic2
AK016276	Ajap1	Erlin1
AK016307	Ajuba	Ern1
AK016335	Ak1	Espl1
AK016388	Ak3	Espn
AK016511	Ak4	Esr1
AK016630	Akap10	Esr2
AK016693	Akap12	Esrp1
AK016837	Akap17b	Etf1
AK017125	Akap2	Ets1
AK017144	Akap4	Etv3
AK017409	Akap7	Etv4
AK017459	Akap8	Etv5
AK017500	Akap8l	Etv6
AK017756	Akap9	Evl
AK017779	Akirin2	Ewsr1
AK017883	Akna	Exosc4
AK017924	Akp3	Extl2
AK018074	Akr1b10	Extl3
AK018156	Akr1b3///Gm6644	Eya3
AK018490	Akr1b7	Eya4
AK018772	Akr1b8	Ezh1
AK018847	Akr1c12	F11r
AK018967	Akr1c12///Akr1c13	F2rl1
AK018988	Akr1c13	Fa2h
AK018996	Akr1c14	Fabp3
AK019053	Akr1c18	Fads2
AK019083	Akr1c20	Fahd1
AK019114	Akr1c21	Fam110c

AK019124	Akr1cl	Fam111a
AK019137	Akr1d1	Fam20b
AK019154	Akr7a5	Fam49b
AK019365	Akt1	Fam83f
AK019375	Akt3	Fanca
AK019435	Aktip	Fanci
AK019679	Alas1	Farsa
AK019738	Alas2	Fasn
AK019743	Alcam	Fbp1
AK019757	Aldh18a1	Fbxl15
AK019824	Aldh1a1	Fbxo2
AK019844	Aldh1a2	Fbxo27
AK020081	Aldh1a3	Fbxo31
AK020309	Aldh1b1	Fbxo36
AK020388	Aldh7a1	Fbxo4
AK020406	Aldh8a1	Fbxo6
AK020517	Aldh9a1	Fbxo9
AK020549	Alg1	Fbxw7
AK020588	Alg12	Fbxw8
AK020767	Alg13	Fcer1g
AK021028	Alg14	Fcgr1
AK021048	Alg2	Fcgr3
AK021325	Alg3	Fdft1
AK021342	Alk	Fgf11
AK021368	Alkbh5	Fgf12
AK027957	Alox12	Fgf18
AK027991	Alox15	Fgf2
AK028005	Alpk2	Fgf23
AK028224	Alpk3	Fgf4
AK029171	Alpl	Fgfr1
AK029407	Alppl2	Fgfr1op2
AK029443	Als2	Fgfr2
AK029591	Alx3	Fgfr4
AK029605	Alx4	Fgr
AK029624	Alyref	Fign
AK029637	Alyref///Alyref2	Fis1
AK029638	Ambp	Fkbp10
AK029692	Ambra1	Fkbp1a
AK029860	Amelx	Fkbp1b
AK029950	Amh	Fkbp5
AK029956	Amigo1	Flcn
AK030238	Ammecr1	Flot2
AK030281	Ammecr1l	Flt4
AK030763	Amn	Fndc3a
AK030770	Amot	Fntb
AK030920	Ampd2	Fosb
AK031200	Ampd3	Fosl2
AK031204	Amph	Foxb2
AK031278	Amy1	Foxc2
AK031498	Amy2a1///Amy2a2///Amy2a3///Amy2a	Foxd1
AK031561	Anapc1	Foxd3
AK031871	Anapc11	Foxg1
AK031964	Anapc16	Foxk1
AK032024	Anapc5	Foxm1
AK032343	Anapc7	Foxo1
AK032387	Ang3	Foxo4
AK033134	Angel2	Fpr-rs3
AK034016	Angpt1	Frmd4b
AK034282	Angpt2	Ftsj3
AK035031	Angpt4	Fubp1
AK035085	Angptl1	Fus
AK035466	Angptl2	Fut10
AK035515	Angptl3	Fut9
AK035525	Angptl4	Fxr2
AK035573	Ank1	Fyn
AK035706	Ankef1	Fzd6
AK036329	Ankhd1	Fzd7
AK036422	Ankmy2	Fzd8
AK036982	Ankrd1	G6pc
AK037113	Ankrd17	Gabarap
AK037159	Ankrd24	Gabarapl2
AK037841	Ankrd26	Gabbr1
AK038021	Ankrd28	Gabrb1
AK038337	Ankrd33	Gabrb3
AK038627	Ankrd33b	Gad2
AK038992	Ankrd36	Gadd45b
AK039020	Ankrd46	Gadd45g
AK039023	Ankrd54	Gadd45gip1
AK039030	Ankrd6	Gale
AK039117	Ankrd61	Galk1
AK039125	Anks3	Galnt6
AK039132	Anks6	Galr2
AK039372	Ankzf1	Gapdh
AK039373	Ankzf1///Glb1l	Gas1
AK039574	Ano1	Gas2
AK039720	Ano10	Gas5
AK039780	Ano5	Gas8
AK039796	Anp32a	Gast
AK039819	Anp32e	Gata1
AK039862	Anpep	Gata3
AK039882	Antxr1	Gatad2b
AK039958	Antxr2	Gatm
AK040027	Anxa10	Gbf1
AK040172	Anxa11///Gm2260///Gm2274	Gbp2
AK040197	Anxa2	Gbp3
AK040272	Anxa3	Gbx2
AK040276	Anxa5	Gca
AK040320	Anxa6	Gcnt2
AK040430	Anxa8	Gda
AK040752	Anxa9	Gdap10
AK040808	Aoc1	Gdap1l1
AK040967	Aoc2 /// Aoc3	Gdi2
AK040992	Aoc3	Gdpd3
AK041042	Aof1	Gdpd5
AK041537	Aox3	Gfi1b
AK041587	Aox4	Gfpt2
AK041716	Ap1g1	Gfra2
AK041760	Ap1m1	Gga1
AK041781	Ap1s2	Ggnbp2
AK042136	Ap1s3	Ggps1
AK042354	Ap2a1	Ghrh
AK042445	Ap2a2	Gipc2
AK042619	Ap2b1	Gja10
AK042665	Ap2m1	Gjb2
AK042807	Ap2s1	Gjc1
AK043085	Ap3b2	Gkap1
AK043101	Ap3m1	Glb1l
AK043130	Ap3m2	Gldn
AK043242	Ap3s1	Gli1
AK043286	Ap3s2	Gli3
AK043361	Ap4b1	Glipr2
AK043509	Ap4m1	Glis1
AK043659	Ap4s1	Glis2
AK043662	Ap5m1	Glo1
AK043723	Ap5s1	Glul
AK043747	Apaf1	Gm11437
AK043766	Apba3	Gm13889
AK043804	Apbb1	Gm561
AK043844	Apbb2	Gna11
AK043919	Apc	Gnb5
AK043958	Apc2	Gne
AK043964	Apcdd1	Gnl2
AK043991	Apex2	Gnpnat1
AK043994	Api5	Gns
AK044033	Apip	Golm1
AK044242	Aplf	Golph3
AK044313	Apln	Golph3l
AK044329	Aplnr	Gpa33
AK044354	Aplp1	Gpaa1
AK044380	Aplp2	Gpatch2
AK044422	Apoa4	Gpc4
AK044435	Apoa5	Gpc6
AK044447	Apobec1	Gpr108
AK044563	Apobec2	Gpr162
AK044623	Apobec3	Gpr177
AK044653	Apobr	Gprc5c
AK044848	Apoc1	Gps1
AK045070	Apoc2	Gpt2
AK045290	Apoc3	Grb14

AK045519	Apoc4	Grb2
AK045643	Apod	Grcc10
AK045681	Apof	Grhl2
AK045818	Apoh	Grhl3
AK045913	Apom	Grid2
AK045951	App	Grin2d
AK046224	Appl2	Grk4
AK046349	Aptx	Grk5
AK046351	Aqp1	Grm8
AK046742	Aqp2	Gsta4
AK047199	Aqp3	Gstm1
AK047243	Aqp4	Gtf2a1
AK047309	Aqp5	Gtf2h4
AK047366	Aqp8	Gtf2i
AK047441	Aqp9	Gtf2ird1
AK047727	Aqr	Gtf2ird2
AK047916	Ar	Gtl3
AK047978	Arap1	Gtpbp6
AK047988	Arap2	Guca1a
AK048089	Arap3	Gyltl1b
AK048292	Areg	Gzmb
AK048368	Arf1	H13
AK048523	Arf2	H1f0
AK048630	Arf3	H1foo
AK048681	Arf4	H2-Ke6
AK049282	Arf5	H2-T24
AK049295	Arf6	H2afj
AK049860	Arfgap2	H6pd
AK049871	Arg1	Hamp2
AK050700	Arg2	Hax1
AK050707	Arhgap10	Hcn2
AK050940	Arhgap20	Hcn3
AK051019	Arhgap24	Hdc
AK051887	Arhgap29	Hdgfrp2
AK052161	Arhgap30	Hdgfrp3
AK052319	Arhgap31	Hdlbp
AK052590	Arhgap39	Heatr2
AK053109	Arhgap6	Helb
AK053193	Arhgap9	Herc1
AK053270	Arhgdia	Herc2
AK053334	Arhgef10l	Herc4
AK053590	Arhgef16	Hes1
AK053689	Arhgef2	Hes7
AK053703	Arhgef25	Hfe
AK053722	Arhgef28	Hhip
AK053772	Arhgef40	Hic1
AK053982	Arhgef7	Higd1b
AK054201	Arhgef9	Hip1
AK075572	Arid1a	Hipk2
AK076253	Arid3b	Hipk3
AK076358	Arid4b	Hira
AK076396	Arid5b	Hist1h1c
AK076606	Arih2	Hk1
AK076672	Arl1	Hlf
AK076687	Arl10	Hmbs
AK076849	Arl14ep	Hmg20a
AK076939	Arl16	Hmga2
AK076950	Arl2	Hmgcl
AK076961	Arl2bp	Hmox1
AK077040	Arl4c	Hnrnpa1
AK077427	Arl4d	Hnrnpa2b1
AK077587	Arl5a	Hnrnpl
AK077682	Arl6	Hook2
AK077811	Arl6ip6	Hormad1
AK078290	Arl8b	Hoxa10
AK078307	Armc1	Hoxa2
AK078316	Armc12	Hoxa9
AK078352	Armc6	Hoxb9
AK078363	Armcx1	Hoxc4
AK078466	Armcx2	Hoxc5
AK078566	Armcx3	Hoxc6
AK078937	Armcx4	Hoxc8
AK078979	Armcx6	Hoxc9
AK079018	Arnt	Hoxd1
AK079034	Arntl	Hoxd10
AK079730	Arntl2	Hoxd3
AK079754	Arpc1b///Gm5637	Hoxd8
AK079777	Arpc4	Hoxd9
AK079912	Arpp19	Hrh1
AK079939	Arpp21	Hs2st1
AK080165	Arr3	Hs3st1
AK080173	Arrb1	Hs3st3b1
AK080176	Arrb2	Hsd17b1
AK080292	Arrdc3	Hsp90b1
AK080296	Arsg	Hspa4
AK080350	Arsi	Hspa5
AK080383	Arsj	Hspa8
AK080399	Art2a-ps	Hspa9
AK080416	Art2b	Hspb6
AK080648	Art5	Hspb7
AK080857	Artn	Hspg2
AK081136	Arvcf	Htr6
AK081407	Arx	Htra2
AK081796	Arxes1///Arxes2	Hyou1
AK082086	As3mt	Ibtk
AK082264	Asah2	Icam2
AK082314	Asap1	Icam4
AK082514	Asb10	Icam5
AK082624	Asb11	Ick
AK082896	Asb12	Icosl
AK083021	Asb13	Id2
AK083203	Asb15	Idi1
AK083994	Asb17	Ier5
AK084270	Asb18	Ier5l
AK084284	Asb4	Ifi35
AK084778	Asb5	Ifih1
AK084828	Asb6	Ifrd2
AK085125	Asb7	Ift122
AK085275	Asb8	Ift74
AK085522	Asb9	Ift81
AK085912	Ascc2	Igf1r
AK085995	Ascl1	Igf2
AK086164	Asf1a	Igf2bp1
AK086290	Asgr1	Igf2bp2
AK086460	Asic2	Igf2bp3
AK086741	Asl	Igfbp4
AK086852	Aspa	Igll1
AK087178	Asph	Igsf8
AK087351	Aspn	Ikzf2
AK087382	Asprv1	Ikzf4
AK087650	Aspscr1	Il11
AK088734	Asrgl1	Il15ra
AK088743	Ass1///Gm5424	Il17a
AK088914	Astn1	Il17re
AK088959	Astn2	Il18bp
AK089118	Asz1	Il1r1
AK089373	Atad3a	Il1rap
AK122209	Atcay	Il25
AK122525	Ate1	Il2ra
AK131719	Atf2	Il6ra
AK131739	Atf5	Ilf2
AK131819	Atf6b	Ilf3
AK131831	Atf7	Inhba
AK132123	Atf7ip2	Ino80
AK132440	Atg101	Inpp1
AK132462	Atg12	Inpp4a
AK132658	Atg2a	Inpp5a
AK132865	Atg3	Ints6
AK132930	Atg5	Invs
AK133153	Atg7	Ip6k1
AK133227	Atg9b	Ipo8
AK133807	Athl1	Iqcb1
AK133929	Atl3	Iqgap1
AK134400	Atn1	Iqgap2
AK134673	Atoh7	Iqsec1
AK134717	Atoh8	Irf1
AK134749	Atox1	Irf6
AK134888	Atp10a	Irf8
AK134928	Atp11a	Irx4

AK135254	Atp11c	Isl1	
AK135429	Atp1a1	Isoc2b	
AK135766	Atp1a2	Itga2	
AK135774	Atp1a3	Itgav	
AK135852	Atp1b1	Itgb1	
AK135878	Atp1b2	Itgb5	
AK136114	Atp2a1	Itm2c	
AK136254	Atp2a3	Itpr1	
AK136654	Atp2b2	Itpr2	
AK136742	Atp4a	Jag1	
AK136817	Atp6v0a1	Jak2	
AK136919	Atp6v0a2	Jam3	
AK137033	Atp6v0a4	Jmjd6	
AK137492	Atp6v1b1	Jmy	
AK137536	Atp6v1c1	Jph3	
AK137552	Atp6v1e1	Junb	
AK138212	Atp6v1g1	Kalrn	
AK138755	Atp6v1g2	Kcna7	
AK138781	Atp7a	Kcnb1	
AK139063	Atp7b	Kcnd2	
AK139229	Atp8a1	Kcnh2	
AK139280	Atp8b2	Kcnh3	
AK139295	Atpif1	Kcnip1	
AK139424	Atr	Kcnj1	
AK139523	Atraid	Kcnj2	
AK139607	Atrip///Trex1	Kcnj8	
AK139890	Atrx	Kcnk1	
AK140090	Atxn10	Kcnma1	
AK140178	Atxn7	Kcnn1	
AK140192	Atxn7l3b	Kcnn2	
AK140218	Auh	Kcnq4	
AK140221	Aup1	Kctd17	
AK140370	Aurka	Kdelc1	
AK140384	Aurkc	Kdelr3	
AK140390	Auts2	Keap1	
AK140632	Avp	Khdrbs1	
AK141411	Avpr1a	Kif11	
AK141565	Avpr1b	Kif13a	
AK141659	Axin1	Kif17	
AK141900	Axin2	Kif1b	
AK142074	Axl	Kif1c	
AK142161	Azgp1	Kif2a	
AK142244	Azin1	Kif5c	
AK142386	B020004J07Rik	Kifc2	
AK142593	B130024G19Rik	Kiss1r	
AK142614	B230118H07Rik	Klc1	
AK142681	B230120H23Rik	Klf7	
AK143097	B230215L15Rik	Klf9	
AK143417	B230217C12Rik	Klhl20	
AK143784	B230219D22Rik	Klhl25	
AK143926	B230354K17Rik	Klhl5	
AK143982	B3galnt1	Klrb1b	
AK144266	B3galt1	Kpna4	
AK144299	B3galt2	Kpnb1	
AK144330	B3galt5	Kras	
AK144367	B3galt6	Kremen1	
AK144485	B3gat1	Krt10	
AK144536	B3gnt1	Krt15	
AK144545	B3gnt2	Krt20	
AK144571	B3gnt3	Krt28	
AK144610	B3gnt4	Krt8	
AK144811	B3gnt5	Krtcap2	
AK144829	B3gnt9	Ksr1	
AK144852	B4galnt1	Lag3	
AK144859	B4galnt2	Lamb2	
AK144870	B4galt2	Lamc1	
AK144909	B4galt3	Lamc3	
AK145113	B4galt4	Lancl2	
AK145129	B4galt6	Laptm4a	
AK145231	B4galt6 /// LOC675709	Laptm5	
AK145276	B4galt7	Lasp1	
AK145301	B930003M22Rik	Lat2	
AK145347	B930041F14Rik	Lats2	
AK145360	B9d1	Lbx2	
AK145396	B9d2	Lcat	
AK146012	BB144871	Lct	
AK146407	BC003331	Ldb1	
AK146690	BC005512///F630007L15Rik///Gm6958	Ldhb	
AK147911	BC005537	Lef1	
AK148126	BC005561	Leprel2	
AK148321	BC005624	Leprotl1	
AK148373	BC005685	Lfng	
AK149094	BC006965	Lgals1	
AK149260	BC016548	Lgals2	
AK149295	BC016579	Lgr5	
AK149307	BC017158	Lhfpl2	
AK149321	BC018242	Lhx1	
AK149987	BC018473///BC018473	Lhx2	
AK152002	BC021614	Lhx5	
AK153534	BC022687	Lif	
AK153849	BC022960	Lig3	
AK153988	BC023829	Limd1	
AK154034	BC025446	Limk1	
AK154177	BC025933	Lipg	
AK154275	BC027072	Lman1	
AK154427	BC028528	Lmna	
AK155149	BC028777	Lmnb2	
AK155239	BC031181	Loxl3	
AK155592	BC031353	Lpgat1	
AK155877	BC038268	Lpin3	
AK156427	BC043118	Lpxn	
AK156477	BC051019	Lrba	
AK157237	BC051142	Lrig1	
AK157519	BC051212	Lrp1	
AK157788	BC053393	Lrp1b	
AK157795	BC065403	Lrp8	
AK157878	BC089491	Lrrc20	
AK157916	BC089597	Lrrc46	
AK157963	BC094435	Lrrc59	
AK158018	BC094435///Ccrn4l///Cog6///Sgip1	Lrrc6	
AK158030	BC100451	Lrrc8e	
AK158244	BC100530 /// Stfa1 /// Stfa3	Lrrk2	
AK158379	BICC1	Lrrn1	
AK158414	Baalc	Lrrtm1	
AK158854	Baat	Lrrtm3	
AK160141	Bace1	Lsamp	
AK160260	Bace2	Lsm2	
AK160502	Bach2	Lss	
AK161079	Bad	Lta4h	
AK161406	Bag5	Ltb4r1	
AK161459	Bai1	Ltbp2	
AK161599	Bai2	Ltbp3	
AK162363	Bai3	Ltbp4	
AK162386	Baiap2	Luc7l2	
AK162870	Baiap2l1	Ly6a	
AK162906	Baiap3	Lyrm2	
AK162930	Bak1	Lyrm5	
AK163067	Bambi	Mab21l2	
AK163149	Banf1	Mad1l1	
AK163160	Barx1	Mad2l2	
AK163429	Basp1	Madd	
AK163667	Batf	Maff	
AK163710	Batf3	Mageb3	
AK163711	Baz1b	Magi1	
AK164126	Bbc3	Magi3	
AK164218	Bbip1	Man1a	
AK164420	Bbx	Manbal	
AK164611	Bcan	Map2k7	
AK164650	Bcap31	Map3k14	
AK164779	Bcar1	Map3k3	
AK164829	Bcas3	Map3k7	
AK165089	Bcat1	Map4k1	
AK165329	Bcat2	Mapk14	
AK165776	Bche	Mapk8ip2	
AK166079	Bckdhb	Mapk9	
AK166453	Bcl10	Mapkap1	
AK167137	Bcl11a	Mapre2	
AK168184	Bcl2	Mapt	
AK168746	Bcl2a1a /// Bcl2a1b /// Bcl2a1d		Mar-09

AK169958	Bcl2a1a///Bcl2a1b///Bcl2a1c///Bcl2a1d	Marcks
AK170078	Bcl2a1a///Bcl2a1b///Bcl2a1d	Marcksl1
AK171117	Bcl2l1	Mark2
AK173072	Bcl2l10	Mark4
AK177174	Bcl2l11	Marveld1
AK177718	Bcl2l14	Mast1
AK178562	Bcl2l2	Mastl
AK180911	Bcl3	Mat1a
AK183279	Bcl6	Mat2a
AK184225	Bcl7b	Mbd3
AK184603	Bcl9	Mbd4
AK187594	Bco2	Mcam
AK188991	Bdh2	Mcfd2
AK191411	Bdkrb1	Mcm3
AK193394	Bdnf	Mcm5
AK194920	Bean1	Mcm9
AK195902	Becn1	Mcoln3
AK197603	Bex1	Mcts1
AK198984	Bex4	Me1
AK202663	Bex6	Med1
AK202794	Bfar	Med12l
AK203140	Bfsp1	Med20
AK204799	Bgn	Med21
AK206637	Bhlha15	Med24
AK207593	Bhlhe22	Med29
AK209600	Bhlhe40	Mef2b
AK213543	Bhlhe41	Mef2d
AK216043	Bhmt2	Meis2
AK217863	Bicc1	Meis3
AK218399	Bicd1	Mesdc1
AK219239	Bicd2	Mesdc2
AK220484	Bid	Met
AKT2	Bik	Mettl1
AL451076.1	Bin1	Mettl6
AL589870.1	Birc3	Mfi2
AL732528.1	Birc6	Mfng
AL772376.2	Bloc1s6	Mfsd11
AL772404.1	Bmf	Mga
AL808014.1	Bmi1	Mgat5
ALDH1B1	Bmp1	Mical2
ALG10	Bmp10	Mid1
ALG9	Bmp15	Midn
ALOX5	Bmp2	Minpp1
ALS2	Bmp2k	Mlf2
AM295304	Bmp3	Mllt10
AMY1A	Bmp4	Mllt11
ANGEL2	Bmp5	Mlx
ANKRD17	Bmp6	Mlxipl
ANP32A	Bmp7	Mme
AP3S2	Bmp8a	Mmp16
APOL3	Bmpr1a	Mns1
ARHGDIB	Bmpr1b	Mnt
ARPC2	Bmx	Mog
ASB8	Bnc1	Mov10
ATF3	Bnc2	Moxd1
ATF6	Bnip2	Mphosph9
ATN1	Bnip3	Mpi
ATP2A3	Bnipl	Mpp5
ATP6V0D1	Boc	Mpp6
ATXN7L2	Bola1	Mpped2
AU014645	Bola2	Mpzl2
AU020772	Boll	Mr1
AU040320	Bpgm	Mrc1
AU040829	Bpifa1	Mrc2
AURKA	Bpifa2	Mrpl10
AV340375	Bpifb1	Mrpl27
AW047730	Bpifb5	Mrpl34
AW549877	Bpil2	Mrpl45
AW555464	Bpnt1	Mrpl51
AY074887	Bptf	Mrps23
AY512942	Brap	Mrps33
AY589788	Brat1	Mrvi1
AY589789	Brca1	Msh2
AY589791	Brcc3	Msh5
AY590888	Brd2	Msi1
AY672066	Brd3	Msl2
AZ2	Brd4	Msx1
Aaas	Brd8	Msx3
Aadacl1	Brdt	Mt1
Aars	Bre	Mt2
Aars2	Brinp1	Mta2
Aarsl	Brms1	Mta3
Aatf	Brox	Mtch2
Aatk	Brsk1	Mtdh
Abat	Brwd1	Mtmr2
Abca1	Brwd3	Mtmr4
Abca17	Bscl2	Mtpn
Abca3	Bsg	Muc4
Abca5	Bst1	Mus81
Abca7	Bst2	Mxi1
Abca8a	Btbd1	Myc
Abcb7	Btbd17	Mycbpap
Abcc10	Btbd2	Mycn
Abcc2	Btd	Myl4
Abcc5	Btg4	Mylpf
Abcd4	Btk	Mynn
Abcf1	Btn1a1	Myo10
Abcf2	Btnl10	Myo15b
Abcf3	Btrc	Myo1c
Abhd11	Bub1	Myo1f
Abhd14b	Bub3	Myo6
Abhd3	Bud31	Myt1
Abhd4	Bves	Myzap
Abhd6	Bysl	Nab2
Abt1	C030003D03Rik	Nav2
Acaa1a	C030018P15Rik	Nbea
Acaca	C030019I05Rik	Nbl1
Acad10	C030026M15Rik	Ncdn
Acadvl	C030033M12Rik /// Rsf1	Ncf1
Acbd3	C030044C12Rik	Ncln
Acbd4	C130026I21Rik///LOC102634528	Ncoa3
Acbd5	C130026I21Rik///LOC102638047	Ncoa7
Accn1	C130090K23Rik	Ndrg3
Accn3	C1d	Ndrg4
Accn4	C1galt1c1	Ndufa2
Ache	C1qb	Ndufa9
Acin1	C1qbp	Ndufaf4
Aco2	C1qc	Ndufs8
Acot11	C1ql3	Nebl
Acot2	C1qtnf1	Nedd4l
Acox1	C1qtnf2	Nedd9
Acoxl	C1qtnf4	Nefh
Acp2	C1qtnf5///Mfrp	Nefl
Acp5	C1qtnf6	Nek8
Acp6	C1qtnf7	Nek9
Acsl5	C1ra	Nell2
Acsm5	C1ra///C1rb	Nes
Acss2	C1s /// LOC100044326	Neu3
Actg1	C1s1	Neurog2
Actn4	C2	Nf1
Actr2	C230052I12Rik	Nfat5
Acvr1c	C230078M08Rik	Nfe2
Acvrl1	C2cd2l	Nfia
Adam10	C3	Nfib
Adam15	C330006A16Rik	Nfix
Adam17	C330007P06Rik	Nfkb2
Adam5	C330027C09Rik	Nfu1
Adamts10	C3ar1	Ninj2
Adamts17	C430049B03Rik,Mir322,Mir351,Mir503	Nkd1
Adamts20	C4a///C4b	Nle1
Adamts4	C4bp	Nlk
Adamts6	C4bp-ps1	Nme1
Adamts7	C530030P08Rik	Nme2
Adamtsl4	C5ar1	Nmt1
Adamtsl5	C76132	Nodal
Adap1	C76336	Nog
Adar	C76472	Nol6
Adarb1	C76533	Nos1
Adat1	C76554	Nos3

Adck3	C76614	Nosip
Adcy2	C76628	Notch4
Adcy8	C77027	Nova1
Adhfe1	C77137	Npas2
Adipoq	C77144	Npas3
Adipor1	C77370	Npas4
Adipor2	C77405	Npepps
Adm	C77545	Nphp1
Adnp	C77609	Nphs1
Adora1	C77681	Npr3
Adpgk	C77691	Nr1d1
Adprh	C77815	Nr1d2
Adra1d	C78228	Nr1h3
Adra2b	C78441	Nr1h4
Adrbk2	C78444	Nr1i3
Adss	C78513	Nr2c2
Aebp2	C78532	Nr2e3
Afap1l1	C78651	Nr2f2
Aff1	C78859	Nr3c2
Afg3l1	C78878	Nr4a2
Afmid	C78891	Nr4a3
Aftph	C78948	Nr5a1
Agap2	C79122	Nrgn
Agbl5	C79130	Nrip1
Agk	C79242	Nrn1
Agmo	C79246	Nrp1
Agpat2	C79329	Nrp2
Agpat6	C79452	Nrxn1
Agpat7	C79468	Nsdhl
Agps	C79490	Nsun4
Agrp	C79562	Nt5dc3
Agtrl1	C79709	Ntn1
Agxt2l2	C80060	Ntng1
Ahctf1	C80068	Ntrk3
Ahdc1	C80171	Nub1
Ahi-1	C80256	Nudcd1
Ahi1	C80360	Nudt16l1
Ahnak	C80425	Nudt4
Ahsa1	C80678	Numb
Aip	C80993	Nup54
Aire	C81600	Nup62
Akap1	C86090	Nwd1
Akap12	C86187	Nxn
Akap2	C87436	Nxnl2
Akap3	C920021L13Rik///C920021L13Rik	Nxph1
Akap9	C920025E04Rik /// H2-T23	Oasl2
Akna	C920025E04Rik///H2-T23///LOC102641[illegible]	Oat
Akr1a4	CRTC1	Obox3
Akt1	CSN1S1	Odc1
Akt2	Caap1	Odz2
Alad	Cab39l	Oit1
Alas1	Cabp1	Ola1
Alcam	Cabp2	Olfm2
Aldh18a1	Cabp4	Olfm3
Aldh3a1	Cabyr	Olfml3
Aldh3a2	Cacfd1	Olig3
Aldh3b1	Cacna1a	Onecut1
Aldh6a1	Cacna1b	Osbpl1a
Alg3	Cacna1c	Osbpl9
Alg8	Cacna1d	Osr1
Alk	Cacna1e	Osr2
Alkbh4	Cacna1f	Otud4
Alox12	Cacna1g	Otud7b
Alox12e	Cacna1h	Otx2
Aloxe3	Cacna2d1	Ovca2
Alpl	Cacna2d2	P2rx4
Alppl2	Cacna2d3	P4ha1
Als2cr2	Cacnb2	P4hb
Alx1	Cacnb3	Pabpn1
Amac1	Cacnb4	Padi1
Ambra1	Cacng1	Pafah1b2
Amd-ps4	Cacng2	Pafah1b3
Amd2	Cacng3	Pak1
Amh	Cacng4	Palm
Amn	Cacng5	Pard3
Amot	Cacng7	Parp3
Amotl1	Cacul1	Parp9
Ampd2	Cad	Parva
Anapc13	Cadm2	Patz1
Anapc4	Cadm3	Pax3
Anapc7	Cadps	Pax6
Angptl4	Cadps2	Pax7
Ank1	Calb1	Pbx3
Ank2	Calb2	Pbxip1
Ankar	Calca	Pcbd1
Ankfy1	Calcb	Pcdh10
Ankhd1	Calcoco2	Pcgf1
Ankhzn	Calcr	Pcgf2
Ankib1	Calcrl	Pcgf6
Ankra2	Cald1	Pcmt1
Ankrd10	Calm1///Calm2///Calm3	Pcnp
Ankrd11	Calm4	Pcnx
Ankrd13b	Calml4	Pcp4
Ankrd13d	Caln1	Pcp4l1
Ankrd17	Calr	Pctk2
Ankrd24	Calu	Pcyt1a
Ankrd33	Camk1	Pde1b
Ankrd34	Camk1g	Pde3a
Ankrd35	Camk2b	Pde3b
Ankrd36	Camk2d	Pde4a
Ankrd40	Camk2n1	Pde4dip
Ankrd45	Camk2n2	Pde6b
Ankrd48	Camk4	Pde6d
Ankrd56	Camkk2	Pde7b
Anp32a	Camp	Pde9a
Anp32e	Camsap1	Pdgfc
Anxa4	Cand1	Pdgfra
Anxa7	Cant1	Pdia3
Anxa8	Canx	Pdlim1
Anxa9	Cap1	Pdlim7
Ap1b1	Cap2	Pdrg1
Ap1g1	Capn1	Pdx1
Ap1g2	Capn10	Pdxk
Ap1s3	Capn12	Pdzrn3
Ap2b1	Capn15	Pemt
Ap2m1	Capn3	Pex13
Ap3d1	Capn6	Pex16
Ap3m2	Capn8	Pex6
Ap4e1	Capn9	Pfkfb2
Ap4m1	Capns1	Pfkl
Ap4s1	Capns2	Pgk2
Apa1	Caprin1	Pglyrp1
Apba2bp	Capzb	Pgm1
Apcs	Car1	Pgm2l1
Apex1	Car10	Phc1
Apip	Car11	Phex
Apobec1	Car13	Phf19
Apoc1	Car3	Phf21a
Apoc2	Car4	Php
Apoc4	Car5b	Phldb1
Apod	Car6	Phldb2
Apoe	Car8	Phospho2
Apol11a	Car9	Pi4k2a
Apol11b	Carf	Pi4ka
Apol7e	Carhsp1	Pign
App	Cars	Pip4k2c
Aptx	Cartpt	Pip5k1a
Aqp11	Casd1	Pip5k1b
Aqp8	Casp12	Pipox
Arc	Casp12 /// LOC100044205	Pitpnc1
Arcn1	Casp14	Pitpnm1
Arf3	Casp3	Pitx2
Arf4	Casp4	Pitx3
Arf6	Casq2	Pkig
Arfgef2	Casr	Pkmyt1
Arfip1	Catsper1	Pknox2
Arfrp1	Catsperg1	Pla1a
Arhgap1	Cav2	Pla2g12a
Arhgap11a	Cav3	Pla2g6

Arhgap17	Cbfa2t3	Plaa
Arhgap19	Cbl	Plagl2
Arhgap21	Cblb	Plcb2
Arhgap23	Cblc	Plcl2
Arhgap24	Cbll1	Plek2
Arhgap26	Cbln1 /// LOC100046314	Plekha2
Arhgap27	Cbln3	Plekha4
Arhgap28	Cbs	Plekha5
Arhgap29	Cbx1	Plekhh2
Arhgap30	Cbx2	Plk2
Arhgap31	Cbx3	Plod3
Arhgap42	Cbx3///Gm5792///Gm6901///Gm6917	Plscr1
Arhgap5	Cbx5	Plscr2
Arhgef1	Cbx6	Plscr3
Arhgef12	Cby1	Plxnc1
Arhgef15	Ccar2	Plxnd1
Arhgef16	Ccbe1	Pml
Arhgef17	Ccdc101	Pmp22
Arhgef18	Ccdc102a	Pmpcb
Arhgef19	Ccdc104	Pnliprp1
Arhgef2	Ccdc106	Pnpla2
Arhgef5	Ccdc107	Pnpla7
Arhgef7	Ccdc109b	Pofut1
Arid1a	Ccdc114	Pold4
Arid1b	Ccdc117	Polg
Arid3a	Ccdc12	Polh
Arid3b	Ccdc120	Polr2a
Arid3c	Ccdc127	Polr3gl
Arid4b	Ccdc132	Polr3k
Arid5a	Ccdc136	Pop5
Arid5b	Ccdc138	Por
Arip2	Ccdc166	Pou2af1
Arl1	Ccdc167	Pou4f3
Arl2bp	Ccdc175	Ppap2b
Arl3	Ccdc25	Ppap2c
Arl4d	Ccdc28a	Ppapdc3
Arl5c	Ccdc34	Pparg
Arl6ip2	Ccdc34///LOC102632005	Ppargc1a
Arl6ip6	Ccdc39	Ppm1g
Arl8b	Ccdc50	Ppox
Armc1	Ccdc53	Ppp1cc
Armc10	Ccdc58	Ppp1r14d
Armc6	Ccdc71l	Ppp1r1b
Armc7	Ccdc80	Ppp1r8
Armet	Ccdc85a	Ppp2cb
Arnt	Ccdc86	Ppp2r3c
Arntl2	Ccdc88a	Ppp2r5b
Arpc4	Ccdc91	Ppp2r5c
Arpm1	Ccdc93	Ppp4r2
Arrdc3	Ccdc97	Pprc1
Arrdc5	Ccer1	Prcp
Ars2	Cckar	Prdm1
Arsg	Cckbr	Prdx3
Art2a-ps	Ccl11	Prelid2
Art2b	Ccl12	Prep
Art4	Ccl19///Gm12407///Gm13309///Gm202	Prg2
Arts1	Ccl21a///Ccl21b///Ccl21c///Gm10591//	Prickle1
Arv1	Ccl22	Prkca
Asah2	Ccl25	Prkce
Asb13	Ccl27a	Prkci
Asb2	Ccl27a///Ccl27b///Gm13306///Gm2506	Prkd3
Ascc1	Ccl28	Prkg1
Ascc2	Ccl5	Prmt2
Ascl1	Ccl6	Prmt6
Asf1a	Ccl9	Prok2
Asf1b	Ccm2	Prom1
Ash1l	Ccna1	Pros1
Aspa	Ccnb1///Gm5593	Prpf19
Aste1	Ccnb3	Prpf38b
Asxl1	Ccnc	Prpf4b
Asxl2	Ccnd2	Prph
Atad2b	Ccnd3	Prrg2
Atad4	Ccne1	Prtg
Atad5	Ccng1	Prune
Atf2	Ccng2	Prx
Atf3	Ccnh	Psd3
Atf5	Ccnl2	Psip1
Atf7	Ccnt1	Psma1
Atg16l2	Ccpg1	Psma5
Atg2a	Ccr1	Psmb10
Atg3	Ccr10	Psmb7
Atl2	Ccr2	Psmc5
Atn1	Ccr3	Psmd11
Atoh1	Ccr5	Psmd7
Atox1	Ccr9	Psmf1
Atp1a1	Ccrl1	Pspc1
Atp1a2	Ccrl2	Pstpip2
Atp1b1	Ccs	Ptch1
Atp1b2	Ccsap	Ptch2
Atp2a1	Ccser2	Ptdss2
Atp2b1	Cct3	Pten
Atp2b4	Cct4	Ptgs2
Atp2c1	Cct6b	Ptms
Atp5a1	Cct8	Ptp4a2
Atp5d	Cd14	Ptpn1
Atp5g1	Cd151	Ptpn12
Atp5g2	Cd160	Ptpn18
Atp5h	Cd163	Ptpn6
Atp5k	Cd164	Ptpn9
Atp5l	Cd177	Ptprb
Atp6c2	Cd180	Ptprcap
Atp6v0e2	Cd19	Ptprj
Atp6v1a	Cd1d1	Ptprk
Atp6v1b1	Cd1d2	Ptrf
Atp6v1b2	Cd2	Pttg1ip
Atp6v1c2	Cd200	Pura
Atp6v1d	Cd200r2 /// Cd200r4 /// LOC100044182	Pvt1
Atp6v1e1	Cd200r3	Pxk
Atp6v1g2	Cd207	Pycr2
Atp8a1	Cd209a	Qpct
Atp8b1	Cd209b	Qpctl
Atp8b2	Cd209c	Qprt
Atpbd1c	Cd209e	Rab11b
Atpif1	Cd209f	Rab13
Atr	Cd209g	Rab28
Atxn1	Cd244///LOC677008	Rab2b
Atxn2	Cd247	Rab34
Atxn7l2	Cd248	Rab3d
Atxn7l3	Cd24a	Rab3il1
Auh	Cd274	Rab4a
Aup1	Cd276	Rab8b
Aurkb	Cd28	Rabep1
Auts2	Cd2bp2	Rabgap1
Aven	Cd300a	Rabgap1l
Avpi1	Cd300ld	Rad23a
Axin1	Cd302	Rad51
Axl	Cd320	Rad51ap1
Azi1	Cd34	Rad54l
Azi2	Cd36	Raf1
Azin1	Cd37	Rai1
B130050I23Rik	Cd38	Ralgps2
B230118H07Rik	Cd3e	Raly
B230208H17Rik	Cd40	Ramp2
B230209C24Rik	Cd40lg	Ranbp17
B230217C12Rik	Cd46	Rapgef3
B230217O12Rik	Cd47	Rarg
B230312A22Rik	Cd48	Rasa1
B230378P21Rik	Cd52	Rasa3
B230380D07Rik	Cd53	Rasa4
B230399E16Rik	Cd55	Rasal2
B3bp	Cd55///Daf2	Rasd1
B3gnt2	Cd5l	Rasgrf1
B3gnt3	Cd6	Rassf1
B3gnt6	Cd70	Rassf5
B3gnt7	Cd72	Rbm14
B4galnt2	Cd74	Rbm35a
B4galnt3	Cd79b	Rbm4b
B4galt1	Cd80	Rbm6
B4galt3	Cd81	Rbms2

B630019A10Rik	Cd83	Rbpj	
B930006L02Rik	Cd84	Rbpms	
B9d1	Cd8a	Rc3h2	
B9d2	Cd8b1	Rcan1	
BBS9	Cd93	Rdh10	
BC003267	Cd97	Reep3	
BC003324	Cd99	Reln	
BC004728	Cd99l2	Rerg	
BC005624	Cdadc1	Rev3l	
BC005803	Cdc14b	Rfng	
BC006965	Cdc23	Rftn2	
BC010787	Cdc25c	Rfx3	
BC012717	Cdc27	Rfxank	
BC013529	Cdc2l6	Rgl1	
BC016423	Cdc37	Rgnef	
BC017612	Cdc37l1	Rgs10	
BC017647	Cdc42	Rgs16	
BC018242	Cdc42bpa	Rgs7	
BC019699	Cdc42ep4	Rhbdd2	
BC020182	Cdc42ep5	Rhbdd3	
BC021395	Cdc7	Rhbdl3	
BC021614	Cdc73	Rhd	
BC021767	Cdca3	Rhoc	
BC021919	Cdca5	Rhou	
BC022651	Cdca8	Rimklb	
BC023055	Cdcp1	Rims2	
BC023105	Cdh1	Ripply3	
BC024868	Cdh10	Rit2	
BC025067	Cdh11	Rlbp1	
BC025076	Cdh12	Rnasel	
BC025190	Cdh15	Rnd3	
BC025462	Cdh17	Rnf10	
BC026434	Cdh18	Rnf121	
BC027389	Cdh2	Rnf144b	
BC027685	Cdh2 /// LOC100044363	Rnf157	
BC027814	Cdh20	Rnf187	
BC028528	Cdh22	Rnf19b	
BC028532	Cdh23	Rnf20	
BC028660	Cdh3	Rnf43	
BC029722	Cdh4	Rnps1	
BC029726	Cdh5	Robo1	
BC030307	Cdh8	Rock1	
BC030336	Cdh9	Rora	
BC030398	Cdhr1	Rorb	
BC030440	Cdhr5	Rpl12	
BC030867	Cdipt	Rpl18	
BC030884	Cdk12	Rpl37a	
BC031181	Cdk14	Rpl41	
BC031353	Cdk16	Rpl7	
BC031441	Cdk18	Rpn1	
BC033915	Cdk20	Rpn2	
BC033916	Cdk2ap1	Rprd1b	
BC037393	Cdk2ap2	Rps27	
BC038286	Cdk5	Rps6ka5	
BC038520	Cdk5r1	Rps6kl1	
BC038822	Cdk5rap2	Rpsa	
BC040803	Cdk5rap3	Rragd	
BC042782	Cdk9	Rrp1b	
BC046404	Cdkl2	Rrs1	
BC047062	Cdkn1a	Rsad2	
BC048355	Cdkn1c	Rsph1	
BC048556	Cdkn2a	Rspo2	
BC048585	Cdo1	Rsrc2	
BC048594	Cdpf1	Rtbdn	
BC048666	Cdr2	Rtn2	
BC048672	Cdr2l	Rtn3	
BC048761	Cds2	Rtn4	
BC049760	Cdx2	Rtp3	
BC050078	Cdx4	Rufy1	
BC050122	Ceacam1	Rufy3	
BC051212	Ceacam1///Ceacam2	Rundc3a	
BC052040	Ceacam10	Runx1	
BC053749	Ceacam11	Runx1t1	
BC055463	Ceacam12	Rusc1	
BC055823	Ceacam2	Rusc2	
BC055918	Ceacam9	Ryr2	
BC056189	Cebpa	S100b	
BC057022	Cebpb	S1pr3	
BC057593	Cebpd	Sall1	
BC057627	Cebpg	Sall2	
BC057893	Cebpz	Samd14	
BC058096	Cebpzos	Samd4	
BC058417	Cecr5	Sar1a	
BC058545	Cel	Sash1	
BC059892	Cela1	Sat2	
BC060111	Cela2a	Sbf2	
BC060267	Cela3b	Scamp3	
BC060302	Cela3b///Gm13011///LOC102641402	Scarb1	
BC060988	Cela3b///LOC102641402	Sccpdh	
BC061194	Celf1	Scd1	
BC062185	Celf2	Scd2	
BC062650	Celsr3	Scd3	
BC062821	Cemip	Scml4	
BC063749	Cend1	Scn8a	
BC064451	Cenpb	Scrn2	
BC065087	Cenph	Scube3	
BC065777	Cenpn	Sdccag8	
BC066135	Cenpo	Sec1	
BC068281	Centb1 /// LOC100045877	Sec16b	
BC070417	Centb1 /// Tnk1	Sec62	
BC070425	Cep112	Sec63	
BC076616	Cep131	Seh1l	
BC080769	Cep164	Sel1l3	
BC088983	Cep170	Sema3a	
BC091771	Cep250	Sema4a	
BC094334	Cep350	Sema5a	
BC094408	Cep41	Sema5b	
BC096391	Cep55	Sema6a	
BC099561	Cep57l1		Sep-02
BC099591	Cep68		Sep-04
BC100495	Cep70		Sep-07
BC107364	Cep89		Sep-11
BC112410	Cer1		Sep-15
BC115444	Cercam	Sepw1	
BC126955	Cerk	Serinc3	
BC127131	Cers1///Gdf1	Serpinb9	
BC128327	Cers2	Set	
BCAN	Ces1c	Setd7	
BCAP31	Ces1d	Sez6	
BCAT1	Ces1e	Sf3a3	
BCKDHA	Ces1g	Sf3b2	
BCL11A	Ces2a	Sf3b5	
BCL2A1	Ces2c///Ces2d-ps	Sfmbt2	
BCL6	Ces2e	Sfrp1	
BCL7A	Ces3a///Ces3b	Sfxn1	
BCL7B	Ces4a	Sgca	
BIRC4	Cetn1	Sgip1	
BLNK	Cetn2	Sgk3	
BLR1	Cfb	Sgol1	
BLVRA	Cfd	Sgpl1	
BMS1	Cfdp1	Sgpp1	
BNIP3L	Cfh	Sh2b3	
BRIP1	Cfhr2	Sh3bp5	
BTK	Cfl1	Sh3kbp1	
BTN3A2	Cflar	Sh3pxd2a	
BTN3A3	Cfp	Shank2	
BUD31	Cftr	Shbg	
Bace	Cga	Shc1	
Bace1	Cgnl1	Shc2	
Bach1	Cgref1	Shc3	
Bach2	Chad	Shd	
Bag1	Chchd3	Shmt1	
Bag3	Chchd6	Shoc2	
Bahcc1	Chchd7	Si	
Bahd1	Chd3os	Siah2	
Bai3	Chd4	Sidt1	
Baiap2	Chek1	Sirt4	
Baiap2l2	Chgb	Sit1	
Bak1	Chic1	Six1	

Bambi	Chid1	Six3
Barhl1	Chil1	Six4
Barhl2	Chil3	Skil
Barx1	Chil3///Chil4	Slbp
Barx2	Chka	Slc12a2
Bat1a	Chkb	Slc12a5
Bat2	Chkb///ChkbCpt1b///Cpt1b	Slc12a7
Bat2d	Chmp4c	Slc12a8
Bax	Chmp4c /// LOC100044854	Slc13a3
Baz1b	Chn1	Slc16a3
Baz2a	Chn2	Slc16a8
Baz2b	Chodl	Slc18a1
Bbc3	Chpf	Slc1a4
Bbx	Chpf2	Slc22a17
Bcam	Chpt1	Slc23a2
Bcan	Chrd	Slc25a30
Bcar1	Chrdl1	Slc25a36
Bcar3	Chrdl2	Slc25a39
Bcas1	Chrm2	Slc26a8
Bcas3	Chrm3	Slc2a10
Bcat1	Chrm4	Slc2a2
Bcat2	Chrna1	Slc2a3
Bckdhb	Chrna2	Slc2a4
Bcl11a	Chrna3	Slc30a2
Bcl11b	Chrna4	Slc30a3
Bcl2l1	Chrna5	Slc30a9
Bcl2l13	Chrna6	Slc35a5
Bcl2l14	Chrna7	Slc35b1
Bcl2l2	Chrnb3	Slc35e4
Bcl3	Chrnb4	Slc37a1
Bcl6	Chrnd	Slc37a3
Bcl6b	Chrne	Slc39a7
Bcl7a	Chrng	Slc44a1
Bcl7b	Chst1	Slc4a1
Bcl7c	Chst10	Slc4a7
Bcl9	Chst11	Slc5a2
Bcl9l	Chst11 /// Phactr1	Slc5a4b
Bcs1l	Chst11///Phactr1	Slc6a1
Bdkrb1	Chst12	Slc6a6
Bdkrb2	Chst14	Slc6a9
Bdnf	Chst15	Slc7a5
Bfar	Chst2	Slc7a7
Bhlhb2	Chst3	Slc8a2
Bhlhb3	Chst4	Slc9a3r2
Bin1	Chst5	Slc9a8
Bin3	Chst7	Slco5a1
Birc2	Chst8	Slk
Birc4	Chsy3	Smad3
Birc5	Chtop	Smarcd3
Birc6	Chuk	Smg5
Birc7	Churc1	Snap23
Bivm	Cic	Snap29
Blcap	Cideb	Snapc2
Blmh	Cidec	Snapc5
Blvra	Cipc	Sncaip
Bmf	Cirbp	Sned1
Bmi1	Cisd1	Snrpb
Bmp1	Cish	Snrpd3
Bmp3	Cit	Sntb1
Bmp4	Cited2	Snx5
Bmper	Cited4	Soat1
Bmpr2	Ciz1	Socs1
Bms1	Ckap4	Socs3
Bmyc	Ckb	Sod1
Bnc2	Cklf	Son
Bnip3	Ckmt1	Sorbs1
Bola2	Ckmt2	Sorcs2
Bpag1	Clasp1	Sos1
Bpil2	Clca2	Sox13
Bpnt1	Clca3	Sox4
Bptf	Clcc1	Sp1
Brap	Clcf1	Sp110
Brca1	Clcn1	Sp4
Brd2	Clcn2	Sp6
Brd4	Clcn3	Sp7
Brd8	Clcn4-2	Spag5
Brdt	Clcn6	Spag7
Brip1	Clcnka	Sparc
Brp44	Clcnkb	Spata13
Brpf1	Cldn1	Spata5
Brpf3	Cldn10	Speg
Brsk1	Cldn11	Sphk1
Brunol4	Cldn14	Spin4
Brwd1	Cldn15	Spint1
Bsg	Cldn16	Spire2
Btbd11	Cldn18	Spred2
Btbd2	Cldn19	Spry2
Btbd7	Cldn2	Spsb1
Btg1	Cldn23	Sptlc1
Btg4	Cldn25	Sqle
Btn1a1	Cldn26	Sqstm1
Btnl5	Cldn3	Srgap2
Btnl6	Cldn4	Srgap3
Btnl7	Cldn5	Srp68
Btnl9	Cldn6	Srrm1
Btrc	Cldn9	Srsf3
Bub3	Clec10a	Ss18
Bud13	Clec12b	Ssbp3
Bud31	Clec14a	Ssbp4
Butr1	Clec1b	Ssrp1
Bxdc1	Clec2d	Sstr3
Bxdc5	Clec2h	St3gal1
Bysl	Clec3b	St3gal3
Bzrap1	Clec4a2	St3gal4
C030014I23Rik	Clec4b1	St3gal5
C030014K22Rik	Clec4d	St6gal1
C030044B11Rik	Clec4e	Stac2
C030046I01Rik	Clec4f	Stag1
C10orf2	Clec4g	Stam2
C10orf26	Clec5a	Stap1
C10orf4	Clec7a	Stard10
C11orf67	Clgn	Stat3
C130022K22Rik	Clic5	Stat5a
C130032J12Rik	Clip2	Stat5b
C130034I18Rik	Clip4	Stau1
C130039O16Rik	Clk4	Stc2
C14orf181	Clmn	Steap2
C14orf43	Clmp	Stip1
C15orf57	Clns1a	Stk11ip
C16orf71	Clock	Stk25
C17orf63	Clpb	Stk39
C184	Clps	Stk40
C1QC	Clstn1	Stra13
C1galt1c1	Clstn2	Stra6
C1qa	Clstn3	Strbp
C1qb	Clta	Strn
C1qc	Clu	Strn3
C1ql4	Clvs1	Stt3a
C1qtnf6	Cma1	Stx17
C1s	Cma2///Mcpt9	Stx18
C22orf32	Cmah	Stx3
C230071H18Rik	Cmbl	Stx6
C230081A13Rik	Cmip	Stxbp1
C230093N12Rik	Cmklr1	Stxbp3a
C230094A16Rik	Cml1	Stxbp4
C2CD2	Cml2	Sufu
C3	Cml5	Sulf1
C3orf25	Cmpk1	Sulf2
C430003P19Rik	Cmpk2	Sult1a1
C5	Cmtm1///Cmtm2a	Sult1c2
C630004H02Rik	Cmtm2a	Sumo3
C630028N24Rik	Cmtm3	Suv420h1
C630050I24Rik	Cmtm7	Suz12
C6orf108	Cmtm8	Syk
C6orf222	Cmtr1	Syngr3
C6orf32	Cnbd2	Synj2bp
C730027E14Rik	Cnep1r1	Sypl2
C78409	Cngb1	Syt12
C7orf55	Cngb3	Syt3

C7orf59	Cnih1	Syt6
C87198	Cnih3	Syt7
C87436	Cnn2	Tac4
C87499	Cnn3	Tacc3
C920016K16Rik	Cnnm1	Taf7
CALM2	Cnnm2	Tal1
CAMK1	Cnnm3	Tanc1
CAT56	Cnot2	Tap1
CAT60	Cnot4	Tapbp
CCDC127	Cnot6l	Tapbpl
CCDC19	Cnp	Tardbp
CCDC28B	Cnpy2	Tax1bp3
CCL2	Cnpy3	Tbc1d1
CCL3	Cnr2	Tbkbp1
CCL4	Cntd1	Tbl3
CCL4L1#CCL4L2	Cntf///Zfp91///Zfp91Cntf	Tbp
CCL5	Cntfr	Tbrg4
CCNB1IP1	Cntln	Tbx15
CD180	Cntn1	Tbx18
CD19	Cntn2	Tbx2
CD22	Cntn3	Tbx20
CD24	Cntn4	Tbx21
CD27	Cntn6	Tbx3
CD37	Cntnap1	Tbxas1
CD44	Cntnap2	Tc2n
CD52	Cntnap4	Tceal8
CD53	Cntrl	Tceb1
CD68	Coa3	Tcf15
CD69	Coa5	Tcf20
CD74	Coa7	Tcf3
CD79A	Cobl	Tcf4
CD83	Cobll1	Tcf7l2
CD86	Coch	Tcfap2c
CD97	Cog1	Tcfap2e
CDC123	Cog1///LOC102641618	Tcp11
CDC20	Cog4	Tcte2
CDC37	Coil	Tdrd7
CDK2	Col11a1	Tead1
CDK2AP2	Col11a2	Tead4
CDKN2C	Col12a1	Tesk1
CENPH	Col13a1	Tesk2
CHKA	Col14a1	Tet1
CHM	Col15a1	Tex14
CIITA	Col16a1	Tfrc
CKLF6	Col17a1	Tgfb1
CKS1B	Col19a1	Tgfb1i1
CKS2	Col1a1	Tgfb2
CLCN3	Col1a2	Tgfb3
CLEC4A	Col22a1	Tgfbr3
CLIP4	Col23a1	Tgm2
CLPP	Col25a1	Thap2
CMTM3	Col26a1	Thbs3
COPS6	Col2a1	Thra
CORO1A	Col3a1	Thtpa
CR2	Col4a1	Thumpd3
CRLM3	Col4a2	Thy1
CS444436	Col4a3	Tia1
CS444466	Col4a4	Tial1
CSF1	Col5a1	Tiam1
CSK	Col5a2	Timp2
CTSB	Col5a3	Tinagl1
CTSS	Col6a1	Tk2
CUEDC1	Col6a2	Tlcd1
CWF19L1	Col6a3	Tlk2
CXCL10	Col7a1	Tln1
CXXC1///SPP1	Col8a2	Tlr3
CXorf42	Col9a1	Tm4sf5
CYB5B	Col9a2	Tm7sf3
CYFIP2	Col9a3	Tm9sf3
CYP1A1	Colec12	Tmc2
Cab39	Colq	Tmcc3
Cab39l	Commd5	Tmed4
Cabin1	Commd7	Tmed5
Cabp4	Comp	Tmed8
Cachd1	Comt	Tmeff2
Cacna1a	Comtd1	Tmem106a
Cacna1c	Copb2	Tmem109
Cacna1g	Copg1	Tmem120b
Cacna1h	Copg2os2	Tmem129
Cacna1s	Coprs	Tmem132a
Cacnb1	Cops5	Tmem141
Cacnb2	Cops6	Tmem161a
Cacnb3	Copz1	Tmem16j
Cacng3	Copz2	Tmem30b
Cacng4	Coq10a	Tmem33
Cadm1	Coq3	Tmem55a
Cadm3	Coq5	Tmem82
Cadps2	Corin	Tmem98
Calb1	Coro1a	Tmod3
Calb2	Coro1c	Tmpo
Calm1	Coro2b	Tmprss5
Calm2	Coro6	Tmtc1
Calm3	Cotl1	Tmtc2
Caln1	Cox15	Tnfaip2
Calr	Cox16	Tnfaip8
Calu	Cox17	Tnfrsf19
Camk1	Cox20	Tnfsf13b
Camk1d	Cox5b	Tnfsf9
Camk2a	Cox6b2	Tnks2
Camk2d	Cox7c	Tnrc6a
Camk2n1	Cox8a	Tnrc6b
Camk2n2	Cox8b	Tom1l2
Camta2	Cp	Tomm40
Cand1	Cpa2	Top2b
Canx	Cpa3	Top3a
Capn3	Cpa5	Top3b
Caprin1	Cpb2	Tox
Caprin2	Cpd	Tpd52
Capza2	Cpe /// LOC100046434	Tpi1
Car11	Cpeb1	Tpm1
Car14	Cpeb3	Tpm2
Car3	Cpeb4	Tpm3
Car5a	Cphx1///Cphx2///Cphx3	Tpp2
Car7	Cphx3	Tpst2
Car9	Cplx4	Tpte
Card10	Cpn1	Traf4
Card14	Cpn2	Traf6
Card6	Cpne2	Trak1
Cars	Cpne4	Trappc3
Casc1	Cpq	Trappc4
Caskin1	Cps1	Trem3
Caskin2	Cpsf3	Trh
Casp7	Cpsf4l	Trhr2
Casp8ap2	Cpxm1	Trib3
Casz1	Cpz	Trim2
Catsper2	Cr1l	Trim26
Cbara1	Cr2	Trim31
Cbfa2t1h	Crabp1	Trim34a
Cbfa2t3h	Crabp2	Trim44
Cbfb	Crat	Trim7
Cblb	Crb1	Trim8
Cblc	Creb1	Trio
Cbll1	Creb3	Trip4
Cbr1	Creb3l1	Trp53
Cbr2	Creb3l2	Trp53bp1
Cbx1	Creb3l3	Trpc7
Cbx3	Creb5	Trpm7
Cbx5	Crebrf	Tsc22d1
Cbx6	Crebzf	Tshz2
Cbx7	Creld1	Tspan12
Cbx8	Creld2	Tspan13
Ccar1	Crem	Tspan31
Ccdc103	Crhr1	Tspan33
Ccdc106	Crhr2	Tssk3
Ccdc109a	Crim2	Tst
Ccdc12	Cript	Ttbk2
Ccdc131	Crisp1	Ttc1
Ccdc139	Crisp2	Ttc3
Ccdc142	Crisp3	Ttc39b

Ccdc16	Crispld1	Ttf1
Ccdc18	Crk	Ttk
Ccdc28b	Crkl	Ttll3
Ccdc33	Crlf2	Ttyh2
Ccdc42	Crmp1	Tuba4a
Ccdc45	Crp	Tuba8
Ccdc5	Crtac1	Tubb3
Ccdc51	Crtap	Tufm
Ccdc52	Crtc2	Tulp2
Ccdc53	Crtc3	Tulp3
Ccdc57	Crx	Tusc2
Ccdc6	Crxos	Tusc3
Ccdc60	Crxos1	Tut1
Ccdc7	Cry1	Txnip
Ccdc72	Cryab	Txnl1
Ccdc76	Cryba1	Txnrd1
Ccdc77	Cryba2	U2af1l4
Ccdc85c	Cryba4	Ubap2l
Ccdc88a	Crybb1	Ubc
Ccdc88b	Crybb2	Ube2d3
Ccdc9	Crybb3	Ube2e2
Ccdc90a	Crybg3	Ube2g2
Ccdc91	Cryga	Ube2l6
Ccdc92	Crygb	Ube2v1
Ccdc94	Crygb///Crygc	Ube4b
Ccdc97	Crygd	Ubn1
Ccdc98	Crygn	Ubqln2
Cchcr1	Crygs	Ubtd1
Ccl22	Cryl1	Ucp2
Ccl25	Crym	Ugt2b38
Ccnd1	Cryzl1	Unc13a
Ccnd2	Csad	Unc5c
Ccnd3	Csdc2	Unc93b1
Ccne2	Csf1	Unk
Ccni	Csf2	Upp1
Ccnjl	Csf2ra	Urm1
Ccnk	Csf2rb///Csf2rb2	Usf1
Ccnt1	Csf2rb2	Usp12
Ccnt2	Csf3	Usp13
Ccr10	Csf3r	Usp39
Ccr9	Csgalnact1	Usp48
Ccrl2	Csgalnact2	Utrn
Cct3	Csk	Vamp2
Cct6b	Csn1s1	Vamp5
Cd101	Csn1s2a	Vangl2
Cd109	Csn1s2b	Vapa
Cd14	Csn2	Vars
Cd24a	Csn3	Vasn
Cd27	Csnk1a1	Vav3
Cd274	Csnk1d	Vax2
Cd2ap	Csnk1e	Vcan
Cd2bp2	Csnk1g2	Vcp
Cd37	Csnk2a1	Vegfa
Cd4	Csnk2a2	Vegfc
Cd44	Csnk2b	Vgll4
Cd47	Cspg4	Vil1
Cd68	Cspp1	Vpreb1
Cd7	Csprs///Gm15433///Gm2666///Gm7609	Vps11
Cd79a	Csprs///Gm15433///Gm2666///Gm7609	Vps13c
Cd80	Csrnp3	Vps18
Cd82	Csrp3	Vps25
Cd86	Cst10	Vps35
Cd9	Cst12	Vps72
Cdadc1	Cst7	Vsig2
Cdc123	Cst8	Vstm2b
Cdc14a	Csta	Vwa1
Cdc25a	Cstf1	Vwa5a
Cdc26	Ctbp2	Wdr33
Cdc27	Ctbs	Wdr75
Cdc2l5	Ctc1	Wfdc2
Cdc37	Ctcf	Wnk1
Cdc42	Ctdspl	Wnk4
Cdc42bpb	Ctla4	Wnt10a
Cdc42ep4	Ctnna1	Wnt2b
Cdc42se1	Ctnna2	Wnt5a
Cdc7	Ctnnb1	Wnt5b
Cdc73	Ctnnd2 /// LOC100045979	Wnt6
Cdca3	Ctps2	Wnt7a
Cdca5	Ctr9	Wnt8b
Cdca7	Ctrc	Wnt9a
Cdca7l	Ctrl	Wnt9b
Cdh10	Cts3	Wt1
Cdh18	Cts6	Xiap
Cdh22	Cts8	Xkr6
Cdh24	Ctsb	Ybx1
Cdh5	Ctse	Ybx2
Cdh6	Ctsf	Yes1
Cdh8	Ctsg	Yme1l1
Cdk2	Ctsh	Ythdf1
Cdk3	Ctsj	Ywhaz
Cdk5rap1	Ctsk	Zap70
Cdk5rap3	Ctsl	Zbtb16
Cdk6	Ctsm	Zc3h12a
Cdkal1	Ctso	Zc3hc1
Cdkl2	Ctsq	Zcchc3
Cdkl3	Ctsr	Zfhx3
Cdkn1a	Ctsz	Zfhx4
Cdkn1b	Cttn	Zfp142
Cdkn2a	Cubn	Zfp143
Cdkn2aip	Cuedc1	Zfp148
Cdkn2aipnl	Cuedc2	Zfp160
Cdon	Cul2	Zfp191
Cdr2l	Cul7	Zfp202
Cdrt4	Cul9	Zfp239
Cdv1R	Cuta	Zfp260
Cdx2	Cux1	Zfp277
Cdyl	Cux2	Zfp318
Ceacam16	Cwc15	Zfp346
Cebpe	Cwh43	Zfp36
Cecr5	Cx3cl1	Zfp36l1
Cecr6	Cx3cr1	Zfp395
Cela1	Cxadr	Zfp444
Celsr1	Cxcl1	Zfp462
Cenp-a	Cxcl12	Zfp472
Cenpa	Cxcl13	Zfp503
Cenpc1	Cxcl14	Zfp521
Cenpl	Cxcl16	Zfp553
Cenpp	Cxcl17	Zfp60
Centa1	Cxcl2	Zfp689
Centa2	Cxcl5	Zfp768
Centb1	Cxcr2	Zfp771
Centb2	Cxcr4	Zfp787
Centg1	Cxcr5	Zfp82
Centg2	Cxx1a///Cxx1b	Zfp87
Cep164	Cxx1c	Zfp93
Cep170	Cxxc1	Zfp943
Cep55	Cxxc5	Zfpm1
Cep57	Cyb5b	Zfr
Cep57l1	Cyb5r1	Zfyve16
Cep63	Cyb5r3	Zic1
Cep70	Cyb5rl	Zic2
Cerk	Cyba	Zic4
Cetn3	Cybb	Zic5
Cfhr1	Cyct	Zmat5
Cfl1	Cygb	Zmiz1
Cggbp1	Cyhr1	Zmynd10
ChaK	Cyp11a1	Zmynd11
Chchd3	Cyp11b2	Znhit1
Chchd4	Cyp1a2	Znrf1
Chchd8	Cyp1b1	Zscan12
Chd1	Cyp21a1	Zwint
Chd1l	Cyp26a1	Zyg11a
Chd2	Cyp2a12	
Chd3	Cyp2a4///Cyp2a5	
Chd4	Cyp2c37	
Chd7	Cyp2c38	
Chd8	Cyp2c50	
Chd9	Cyp2c54	
Chgb	Cyp2c65	

Chid1	Cyp2c68
Chit1	Cyp2d10
Chka	Cyp2d13
Chl1	Cyp2d22
Chmp5	Cyp2d9
Chn2	Cyp2e1
Chrd	Cyp2f2
Chrna1	Cyp2g1
Chrnb1	Cyp2j13
Chrnb4	Cyp2j5
Chrp	Cyp3a11
Cib1	Cyp3a16
Cic	Cyp3a25
Cidec	Cyp3a44
Ciita	Cyp46a1
Cirbp	Cyp4f15
Cisd2	Cyp4f16
Cish	Cyp4f16///Cyp4f37
Ckap5	Cyp4v3
Cklf	Cyp51
Cks1b	Cyp7b1
Cks2	Cyp8b1
Clasp1	Cypt1///Cypt7///Cypt8
Clasp2	Cyr61
Clast2	Cys1
Clca5	Cysltr1
Clcf1	Cysltr2
Clcn1	Cyth1
Clcn2	Cyth3
Clcn3	Cyyr1
Clcn4-2	D030011O10Rik
Clcnka	D030029J20Rik
Clcnkb	D030056L22Rik
Cldn15	D11Ertd326e
Cldn23	D12Ertd123e
Cldn7	D12Ertd216e
Cldnd1	D13Ertd205e
Cldnd2	D13Ertd608e///LOC102631530
Clec2h	D15Ertd30e
Clic1	D15Ertd55e
Clic4	D17892
Clim1	D17Wsu104e
Clk2	D17Wsu92e
Clmn	D18Ertd232e
Cln6	D1Ertd259e
Clp1	D1Ertd622e
Clptm1	D1Ertd75e
Clspn	D1Pas1
Clstn1	D2Ertd127e
Clstn3	D2Ertd63e
Cltc	D2Ertd93e
Cmtm4	D2hgdh
Cmtm5	D3Bwg0562e
Cmtm7	D3Ertd246e
Cmtm8	D3Ertd258e
Cnbp2	D3Wsu167e
Cnih	D430019H16Rik
Cnih3	D4Ertd117e
Cnih4	D4Ertd58e
Cnn2	D530037H12Rik
Cnnm2	D5Ertd102e
Cno	D730048I06Rik
Cnot10	D7Bwg0826e
Cnot2	D7Ertd143e///Mir295
Cnot4	D7Ertd183e
Cnot6	D7Ertd187e
Cnot6l	D7Ertd715e
Cnot8	D830044I16Rik
Cnp	D8Ertd107e
Cnpy1	D8Ertd67e
Cntfr	D8Ertd738e
Cntn4	D8Wsu26e
Cntnap1	D930015E06Rik
Coasy	D930048N14Rik
Cog7	D9Ertd26e
Cog8	D9Ertd306e
Col11a2	D9Ertd356e
Col13a1	DDX26B
Col16a1	DIO2
Col17a1	DXBay18///Gm14685///Gm5639///Gm5640///Gm5936
Col18a1	DXErtd223e
Col19a1	DXErtd242e
Col1a1	Daam2
Col28a1	Dab1
Col2a1	Dab2
Col4a5	Dab2ip
Col4a6	Dach1
Col5a2	Dach2
Colec11	Dact1
Commd5	Dad1
Commd6	Daf2
Commd7	Dagla
Comp	Dak
Comtd1	Dand5
Cops4	Dao
Cops5	Dapk1
Cops6	Dapk3
Cops7b	Dapp1
Coq3	Dazap1
Coq5	Dazl
Coq6	Dbf4
Coro1b	Dbh
Coro7	Dbn1
Cotl1	Dbndd1
Cox11	Dbp
Cox17	Dcaf12l1
Cox18	Dcakd
Cox4i2	Dcbld2
Cox7a2	Dcc
Cox7a2l	Dchs1
Cox8a	Dck
Cp	Dclk1
Cpeb3	Dcn
Cpeb4	Dcp1a
Cpm	Dcpp1///Dcpp2///Dcpp3
Cpne2	Dcpp3
Cpne4	Dcps
Cpne8	Dct
Cpne9	Dctn4
Cpped1	Dctn6
Cpsf3l	Dcun1d1
Cpsf6	Dcun1d4
Cpt1a	Dcun1d5
Cpvl	Dcx
Crabp2	Dda1
Crb2	Ddah1
Crbn	Ddah2
Creb1	Ddc
Creb3	Ddhd1
Creb3l4	Ddit3
Creb5	Ddit4l
Crebbp	Ddo
Crebl2	Ddost
Crhr1	Ddr1
Crip1	Ddr2
Crkrs	Ddrgk1
Crlf1	Ddt
Crlf3	Ddx10
Crmp5	Ddx17
Crry	Ddx18
Crtc2	Ddx19b
Cryab	Ddx20
Cryzl1	Ddx25
Csda	Ddx26b
Csde1	Ddx39
Cse1l	Ddx39b
Csf1	Ddx3x
Csmd3	Ddx3y
Csnk1a1	Ddx4

Csnk1g3	Ddx41
Cspg2	Ddx46
Cspp1	Ddx47
Csrp1	Ddx49
Csrp3	Ddx6
Cst3	Ddx60
CtBP1	Decr1
Ctage5	Defa-rs1
Ctbp1	Defa-rs2
Ctbp2	Defa-rs7
Ctcf	Defa1///Defa24///Defa6///Gm15284
Ctdsp1	Defa15
Ctdspl2	Defa3
Ctf1	Defa4
Ctnna1	Defa5///Gm10104///Gm15315
Ctnna2	Defb10
Ctnna3	Defb11
Ctnnd1	Defb13
Ctrl	Defb15
Ctsb	Defb2
Ctss	Defb3
Cuedc1	Defb35
Cuedc2	Defb4
Cul1	Defb5
Cul2	Defb7
Cul4b	Defb8
Cuta	Defb9
Cutl1	Dennd4b
Cutl2	Dennd5a
Cux/CDP(1B1)	Depdc2
Cuzd1	Deptor
Cwf19l1	Derl1
Cwf19l2	Derl3
Cxcl1	Des
Cxcl12	Desi2
Cyb5	Dfna5
Cyb561d1	Dgat1
Cyb561d2	Dgcr14
Cyb5d1	Dgcr2
Cybasc3	Dgka
Cyc1	Dgke
Cygb	Dgkh
Cyhr1	Dguok
Cyld	Dhcr24
Cyp24a1	Dhdds
Cyp26b1	Dhh
Cyp27b1	Dhps
Cyp2c67	Dhrs11
Cyp2c69	Dhrs4
Cyp2d26	Dhrs7
Cyp2j13	Dhrs7b
Cyp2s1	Dhx30
Cyp4f13	Dhx32
Cyp4f16	Dhx36
Cyp4f40	Dhx38
Cyp4v3	Dhx58
Cyp7b1	Diablo
Cypt12	Diap2
Cyr61	Dimt1
Cytl1	Dio1
Cytor4	Dio2
D030011O10Rik	Diras1
D030051N19Rik	Dixdc1
D0H4S114	Dkk1
D10Ertd610e	Dkk2
D10Ertd641e	Dkk4
D11Bwg0434e	Dlat
D11Bwg0517e	Dlc1
D11Wsu47e	Dleu2
D11Wsu99e	Dlg2
D11lgp2	Dlg3
D12Ertd551e	Dlg4
D130043K22Rik	Dlgap4
D130058E05Rik	Dlk1
D130059P03Rik	Dll3
D14Ertd500e	Dlst
D15Wsu75e	Dlx1
D17H6S56E-3	Dlx1as
D17H6S56E-5	Dlx2
D17Wsu92e	Dlx3
D19Ertd652e	Dlx4
D230004N17Rik	Dlx6
D2Ertd391e	Dmc1
D2Ertd750e	Dmgdh
D330012F22Rik	Dmkn
D330022A01Rik	Dmp1
D330037H05Rik	Dmpk
D3Ucla1	Dmrt1
D430033N04Rik	Dmrt2
D4Bwg0951e	Dmrtb1
D4Ertd196e	Dmtf1
D4Ertd22e	Dmtn
D4Ertd429e	Dmxl2
D4Wsu132e	Dnaaf1
D4Wsu53e	Dnah1
D530033C11Rik	Dnah5
D5Wsu178e	Dnah8
D630013G24Rik	Dnahc2
D630037F22Rik	Dnaja1
D630040G17Rik	Dnaja2
D6Mm5e	Dnaja3
D6Wsu116e	Dnajb11
D6Wsu163e	Dnajb3
D930036F22Rik	Dnajb5
D930048N14Rik	Dnajb7
D9Mgc48e	Dnajb9
DAXX	Dnajc1
DCC	Dnajc10
DDIT3	Dnajc12
DDR1	Dnajc15
DEDD	Dnajc17
DEK	Dnajc18
DHFR	Dnajc19///Dnajc19-ps
DHX35	Dnajc2
DIF-3	Dnajc21
DLEU2	Dnajc24
DLL3	Dnajc27
DLX4	Dnajc3
DNMT1	Dnajc30
DPCR1	Dnajc7
DQ542042	Dnali1
DQ549198	Dnase1
DQ551845	Dnase1l2
DQ553546	Dnase1l3
DQ555568	Dnase2a
DQ558020	Dnase2b
DQ564514	Dnd1
DQ568730	Dner
DQ568996	Dnm1
DQ686689	Dnm1l
DQ686988	Dnm2
DQ687171	Dnm3
DQ689104	Dnm3os///Mir214
DQ689702	Dnmt3a
DQ693555	Dnmt3b
DQ694834	Dnmt3l
DQ697726	Dntt
DQ699684	Doc2a
DQ701953	Doc2b
DQ702757	Dock10
DQ703064	Dock2
DQ703302	Dock5
DQ704526	Dock6
DQ704729	Dock7
DQ707380	Dock8
DQ711509	Dok1
DQ711560	Dok5
DQ712497	Dok6
DQ716012	Donson

DQ716032	Dpep1
DQ716740	Dpf1
DQ720410	Dpf3
DQ722851	Dph7
DQ726464	Dpp3
DREAM/calsenilin/KChIP3	Dpp4
DUSP10	Dpp6
DUSP12///DUSP1	Dppa2
DUT	Dppa4
DYNC1I2	Dpt
Dab1	Dpyd
Dab2	Dpys
Dag1	Dpysl2
Dagla	Dpysl3
Dalrd3	Dpysl4
Dand5	Dqx1
Dap3	Dram1
Dapk2	Dram2
Dars2	Drap1
Daxx	Draxin
Dbi	Drd2
Dbil5	Drd3
Dbndd1	Drd4
Dbp	Drg1
Dbt	Drg2
Dbx1	Dsc1
Dcaf8	Dsc2
Dcakd	Dsc3
Dci	Dscam
Dck	Dscaml1
Dclk1	Dsg1a
Dcps	Dsg1b
Dcst1	Dsg2
Dctn3	Dsn1
Dcun1d3	Dsp
Dcun1d4	Dst
Dcxr	Dstyk
Ddah1	Dtd2
Ddah2	Dtl
Ddc	Dtna
Ddc8	Dtnb
Ddef1	Dtnbp1
Ddi2	Dtx1
Ddit3	Dtx3
Ddit4	Dtymk
Ddn	Dusp10
Ddr1	Dusp12///Gm26620
Ddx1	Dusp15
Ddx11	Dusp18
Ddx18	Dusp19
Ddx19b	Dusp2
Ddx21	Dusp26
Ddx23	Dusp3
Ddx27	Dusp9
Ddx39	Dut
Ddx5	Dvl2
Ddx52	Dvl3
Ddx54	Dync1i1
Ddx56	Dynlrb1
Deadc1	Dynlt1a///Dynlt1b///Dynlt1c///Dynlt1f
Dedd	Dynlt1a///Dynlt1c///Dynlt1f
Dedd2	Dynlt1f
Defa-ps17	Dyrk3
Degs1	Dysf
Dek	Dyx1c1
Deltex3	E130203B14Rik
Dennd1b	E230006M18Rik
Dennd1c	E2f1
Dennd2d	E2f2
Dennd4b	E2f5
Dennd4c	E2f6
Depdc1a	E2f8
Depdc1b	E430018J23Rik
Derl1	EG328264
Dgat2	EG545758 /// Slc10a4
Dgka	EG628498 /// ENSMUSG00000076577 /// Igk-C /// Igk-V28 /// LOC100046552 /// LOC100047628
Dgke	ENPP2
Dgki	Eaf2
Dhdds	Ear1///Ear12///Ear2///Ear3
Dhdh	Ear2
Dhh	Ear3
Dhrs13	Ears2
Dhrs3	Ebf1
Dhrs7b	Ebf2
Dhtkd1	Ebf3
Dhx15	Ebi3
Dhx58	Ece1
Diap1	Ece2
Dio3	Ecel1
Dirc2	Echdc3
Dis3	Echs1
Dis3l	Eci1
Disc1	Eci2
Diws1t	Eda
Dixdc1	Edar
Dlat	Edf1///Gm11964
Dlg2	Edil3
Dlg4	Edn1
Dlg5	Edn3
Dlgap2	Ednra
Dlgap4	Ednrb
Dlk2	Eed
Dll1	Eef1a2
Dll4	Eef1d
Dlst	Eef1e1
Dlx3	Eefsec
Dmap1	Efcab14
Dmpk	Efcab2
Dmrt3	Efcc1
Dmrta2	Efemp1
Dmtf1	Efemp2
Dmxl1	Efhd1
Dnahc11	Efna2
Dnahc2	Efna5
Dnahc8	Efnb2
Dnaic2	Efnb3
Dnaja1	Efr3a
Dnajb10	Efr3b
Dnajb14	Eftud2
Dnajb4	Egfbp2
Dnajc12	Egfl7
Dnajc2	Egfl8
Dnajc27	Egfr
Dnajc5g	Egr3
Dnajc8	Ehbp1l1
Dnalc1	Ehd1
Dnali1	Ehd2
Dnase1l3	Ehd3
Dncic2	Ehf
Dnmbp	Ehmt2
Dnmt1	Eid1
Dnmt3a	Eif1a
Dnmt3b	Eif1b
Dock11	Eif2ak1
Dock7	Eif2ak2
Dok1	Eif2ak3
Dok2	Eif2ak4
Dok5	Eif2b2
Dolpp1	Eif2c1
Donson	Eif2c3
Dopey2	Eif2d
Dos	Eif2s2
Dot1	Eif2s3y
Dot1l	Eif3a
Dpagt1	Eif3e
Dpcr1	Eif3h
Dpf2	Eif3l
Dpf3	Eif3m
Dph1	Eif4e

Dph4	Eif4e2
Dpp6	Eif4e3
Dpp8	Eif4ebp1
Dpysl5	Eif4ebp2
Dqx1	Eif4enif1
Drd2	Eif4g1
Drd5	Eif4g2
Dsg1a	Eif4g3
Dspp	Eif5b
Dst	Elac1
Dtd1	Elavl1
Dtx3	Elavl2
Dtx4	Elavl3
Dullard	Elavl4
Dus1l	Elf5
Dusp1	Elk1
Dusp10	Elk3
Dusp16	Elk4
Dusp3	Elmo1
Dusp4	Elmo2
Dusp5	Elmo3
Dusp7	Elmod1
Dvl1	Eln
Dvl2	Elovl2
Dync1i2	Elovl4
Dync1li1	Elovl6
Dynll1	Elovl7
Dynll2	Elp2
Dynlt1a	Eltd1
Dyrk1a	Emc2
Dyrk2	Emc7
Dyrk3	Emc8
Dyrk4	Emcn
Dyx1c1	Emid1
E030030I06Rik	Emilin1
E130003G02Rik	Eml5
E130114P18Rik	Eml6
E130308A19Rik	Emp3
E130310K16Rik	Emr4
E230001N04Rik	En1
E2F1	En2
E2f2	Enah
E2f6	Enam
E2f8	Enc1
E330009J07Rik	Endod1
E330021D16Rik	Endog
E430028B21Rik	Eng
E4f1	Eno1
EBF1	Eno1///Eno1b
EBI3	Eno2
EEF1G	Enoph1
EG328479	Enpep
EG432743	Enpp2
EG434280	Enpp3
EG434402	Enpp4
EG545391	Enpp5
EG545893	Entpd1
EG545963	Entpd7
EG626359	Env
EHD1	Eny2
EIF2S2	Eomes
EIF4EBP1	Epas1
ELA3A	Epb4.1l1
ELA3B	Epb4.1l2
ELF2	Epb4.1l3
ELL3	Epb4.1l4a
ELMOD3	Epb4.2
ELOVL1	Epc1
ELP	Epdr1
EML2	Epgn
ENO3	Epha1
ENSMUSG00000048936	Epha3
EPHA4	Epha4
ET	Epha5
EVI5L	Epha6
EXOSC5	Epha7
Eaf1	Epha8
Eaf2	Ephb1
Ean57	Ephb2
Ear-ps12	Ephb4
Ebf2	Ephb6
Ebf3	Ephx2
Ebf4	Epm2a
Ebi3	Epm2aip1
Ecd	Epor
Ece1	Eppk1
Ece2	Eps15
Ech1	Eps8l3
Edf1	Epsti1
Edg5	Epx
Edg7	Epyc
Edg8	Eqtn
Edn2	Eral1
Eea1	Eras
Eef1g	Erbb3
Efcab2	Erc1
Efcab5	Erc2
Efhc1	Ercc1
Efna2	Ercc2
Efna3	Ercc6l
Efna4	Ercc6l2
Efna5	Ercc8
Efnb2	Erdr1
Efnb3	Ereg
Efs	Erf
Eftud1	Erg
Eftud2	Ergic1
Egfr	Ergic2
Egr3	Ergic3
Ehbp1	Erh///LOC101056061
Ehbp1l1	Eri2
Ehd1	Eri3
Ehd2	Erlin1
Ehd4	Ern1
Ehmt1	Ero1l
Eif1	Ero1lb
Eif2a	Errfi1
Eif2ak3	Esam
Eif2b4	Esco1
Eif2c1	Esm1
Eif2c2	Espl1
Eif2c3	Espn
Eif2s1	Esr1
Eif3h	Esr2
Eif3i	Esrp1
Eif3j	Esrrb
Eif3k	Esrrg
Eif3s12	Esx1
Eif3s2	Esyt1
Eif4a2	Etf1
Eif4e2	Etnk1
Eif4g3	Etos1
Eif4h	Ets1
Eif5a	Etv1///Gm5454
Eif6	Etv2
Elac2	Etv3
Elavl2	Etv4
Elavl3	Etv5
Elf1	Etv6
Elf2	Eva1a
Elk4	Evc
Ell2	Evi2a-evi2b///Evi2b
Ell3	Evl
Elmod2	Evx2
Elovl1	Ewsr1
Elovl5	Exo1
Elovl6	Exoc6///Sh3glb1
Eme1	Exosc1
Emid2	Exosc10

Emilin1	Exosc4
Emx1	Extl2
En1	Extl3
Enah	Eya1
Eno2	Eya2
Eno3	Eya3
Enoph1	Eya4
Enpp4	Ezh1
Entpd1	Ezr
Entpd5	F10
Eny2	F11
Eomes	F11r
Ep300	F13a1
Epb4.1	F13b
Epb4.1l1	F2r
Epb4.1l2	F2rl1
Epb4.1l4a	F2rl3
Epha3	F3
Epha5	F5
Epha7	F630111L10Rik
Ephb1	F7
Ephb2	F9
Ephb4	FAM198A
Ephx3	FGF7
Epn1	FIGNL2
Epn3	Fa2h
Epo	Fabp1
Eprs	Fabp2
Eps8R1	Fabp3
Eps8l1	Fabp4
Eral1	Fabp5
Erap1	Fabp7
Erbb2	Fabp9
Erbb2ip	Fads1
Erbb3	Fads2
Erc1	Fads3
Erd2.1	Faf1
Ergic2	Fahd1
Erlin1	Fam101b
Ermp1	Fam104a
Ern1	Fam105a
Espl1	Fam109b
Espn	Fam110a
Esr1	Fam110c
Esr2	Fam111a
Esrp1	Fam114a1
Etf1	Fam115c
Etfa	Fam117b
Etfdh	Fam118a
Etl4	Fam120a
Ets1	Fam120b
Etv1	Fam122a
Etv3	Fam126a
Etv4	Fam129a
Etv5	Fam131a
Etv6	Fam131b
Evi1	Fam133b
Evl	Fam13c
Evx1	Fam149a
Ewsr1	Fam160b1
Exdl1	Fam161a
Exoc2	Fam166a
Exoc3l2	Fam169a
Exoc6	Fam171b
Exoc7	Fam174a
Exod1	Fam174b
Exosc4	Fam181b
Ext1	Fam184b
Ext2	Fam189b
Extl1	Fam196b
Extl2	Fam198b
Extl3	Fam19a5
Eya3	Fam20b
Eya4	Fam20c
Ezh1	Fam21
Ezh2	Fam210b
F11r	Fam213a
F2	Fam216a
F2rl1	Fam222b
F630043A04Rik	Fam227b
F730014I05Rik	Fam26e
F830021D11Rik	Fam3c
FAE	Fam43a
FAM123B	Fam45a
FAM129C	Fam46c
FAM82A2	Fam49b
FANCF	Fam64a
FBP11	Fam65a
FBXO16	Fam65b
FCER1G	Fam65c
FCER2	Fam69b
FCGR2C///FCGR2B	Fam73b
FCGRT	Fam83f
FCRL4	Fam83g
FCRL5	Fam83h
FCRLA	Fam86
FKBP1b-B	Fam89b///Sssca1
FLII///FLI1	Fam92a
FLJ39739	Fam96a
FLRG	Fanca
FOXP1	Fancc
FOXS1	Fancf
FTH1	Fancg
FTL	Fanci
FUS	Fancm
FYN	Fank1
Fa2h	Fap
Fabp3	Far1
Fads2	Farp1
Fahd1	Farp2
Faim	Farsa
Fam102a	Farsb
Fam110c	Fas
Fam111a	Fasl
Fam123b	Fasn
Fam129b	Fastk
Fam164c	Fastkd2
Fam187a	Fbln1
Fam20b	Fbln2
Fam23a	Fbln5
Fam49b	Fbln7
Fam4a2	Fbn1
Fam57b	Fbn2
Fam5c	Fbp1
Fam73a	Fbp2
Fam76b	Fbrs
Fam77D	Fbxl15
Fam82a2	Fbxl3
Fam83f	Fbxo16
Fanca	Fbxo17
Fancd2	Fbxo2
Fanci	Fbxo21
Farsa	Fbxo22
Farsla	Fbxo27
Fasn	Fbxo3
Fat1	Fbxo31
Fat4	Fbxo32
Fblim1	Fbxo33
Fbp1	Fbxo36
Fbxl10	Fbxo4
Fbxl11	Fbxo6
Fbxl12	Fbxo9
Fbxl13	Fbxw11
Fbxl14	Fbxw14
Fbxl15	Fbxw2
Fbxl19	Fbxw7
Fbxl20	Fbxw8
Fbxl22	Fcer1a
Fbxo10	Fcer1g

Fbxo11	Fcer2a
Fbxo2	Fcgr1
Fbxo25	Fcgr2b
Fbxo27	Fcgr3
Fbxo31	Fcgr4
Fbxo36	Fcgrt
Fbxo4	Fcho1
Fbxo42	Fcrl1
Fbxo44	Fcrla
Fbxo45	Fdft1
Fbxo47	Fdps
Fbxo6	Fech
Fbxo7	Fem1a
Fbxo8	Ferd3l
Fbxo9	Fermt2
Fbxw4	Fermt3
Fbxw5	Fert2
Fbxw6	Fes
Fbxw7	Fetub
Fbxw8	Fez1
Fcer1g	Fez2
Fcgr1	Fezf2
Fcgr3	Ffar2
Fchsd2	Fgd1
Fdft1	Fgd6
Fem1b	Fgf10
Fem1c	Fgf11
Fer1l3	Fgf12
Fermt1	Fgf13
Fev	Fgf14
Fgd5	Fgf16
Fgf11	Fgf17
Fgf12	Fgf18
Fgf15	Fgf2
Fgf18	Fgf22
Fgf2	Fgf23
Fgf23	Fgf3
Fgf4	Fgf4
Fgf8	Fgf5
Fgf9	Fgf6
Fgfr1	Fgf7
Fgfr1op2	Fgfbp1
Fgfr2	Fgfr1
Fgfr4	Fgfr1op2
Fgr	Fgfr2
Fhl3	Fgfr4
Fibcd1	Fgfrl1
Fign	Fgl1
Fignl1	Fgl2
Fis1	Fgr
Fkbp10	Fhit
Fkbp15	Fhl2
Fkbp1a	Fhl4
Fkbp1b	Fhl5
Fkbp4	Fhod3
Fkbp5	Fibin
Fkhl18	Fibp
Fkhr2	Ficd
Flad1	Figf
Flame-3	Figla
Flcn	Fign
Flot2	Fip1l1
Flt3l	Fis1
Flt4	Fkbp10
Fmnl1	Fkbp11
Fmo5	Fkbp1a
Fnbp1l	Fkbp1b
Fndc3a	Fkbp4///LOC102642559
Fndc5	Fkbp5
Fnip1	Fkbp7
Fntb	Fktn
Fos	Flad1///Lenep
Fosb	Flcn
Fosl2	Fli1
Foxb1	Flii
Foxb2	Flna
Foxc2	Flot2
Foxd1	Flrt2
Foxd2	Flt1
Foxd3	Flt3
Foxe1	Flt4
Foxg1	Flywch1
Foxj2	Flywch2
Foxj3	Fmn2
Foxk1	Fmnl3
Foxl1	Fmo1
Foxl2	Fmo2
Foxm1	Fmo3
Foxn3	Fmo4
Foxn4	Fmod
Foxo1	Fmr1
Foxo3a	Fmr1nb
Foxo4	Fn1
Foxp1	Fn3k
Foxp1a	Fnbp1
Foxq1	Fnbp4
Fpr-rs3	Fndc1
Frmd4a	Fndc3a
Frmd4b	Fndc4
Frmd5	Fndc7
Frs2	Fntb
Fry	Folr4
Fscn2	Fosb
Fscn3	Fosl1
Fsd2	Fosl2
Fsrg3	Foxa1
Fst	Foxa3
Fstl3	Foxb2
Fstl4	Foxc2
Ftsj3	Foxd1
Ftsjd2	Foxd3
Fubp1	Foxf1
Fubp3	Foxf2
Fus	Foxg1
Fusip1	Foxh1
Fut10	Foxi1
Fut9	Foxj1
Fxc1	Foxk1
Fxr2	Foxk2
Fyn	Foxm1
Fzd1	Foxn1
Fzd6	Foxo1
Fzd7	Foxo3
Fzd8	Foxo4
G2ad	Foxp2
G3BP	Fpr-rs3
G3bp1	Fpr1
G5pr	Fpr2
G630016D24Rik	Fpr3
G6pc	Frat1
GABA-B-R	Frem1
GADD45B	Frk
GAL3ST4	Frmd3
GCET2	Frmd4b
GGTLC1	Frmd6
GJA8	Frmpd4
GLI3	Frs3
GLT8D1	Frzb
GLUT4	Fscn1
GLYAT///CAT	Fsip1
GMPS	Fstl1
GNAI2	Ftcd
GNAS	Fth1
GNAT1	Fthl17///Gm14458///Gm14499///Gm14511///Gm5634
GNB1	Ftl1///Gm20746///LOC100862446
GNB2	Ftl1///Gm20746///LOC100862446///Mir692-1///Mir692-2b///Mir692-3
GOBLIN	Ftsj3
GOLGA9P	Fubp1
GOT2	Fuca1

GPBP	Fundc1
GPR18	Fundc2
GPR84	Fus
GRINL1A	Fut10
GSK3A	Fut2
GSTP1	Fut7
Gab1	Fut8
Gabarap	Fut9
Gabarapl2	Fv1
Gabbr1	Fxr2
Gabpb2	Fxyd1
Gabrb1	Fxyd2
Gabrb2	Fxyd3
Gabrb3	Fxyd5
Gabrg3	Fxyd6
Gad2	Fxyd7
Gadd45b	Fyb
Gadd45g	Fyn
Gadd45gip1	Fzd2
Gal3st1	Fzd3
Gal3st3	Fzd4
Gale	Fzd5
Galk1	Fzd6
Galnt6	Fzd7
Galr2	Fzd8
Ganc	G0s2
Gapdh	G6pc
Gapdhs	G6pc2
Gapvd1	G6pc3
Garnl4	G6pd2///G6pdx
Gart	GLIS3
Gas1	GPR98
Gas2	Gaa
Gas2l1	Gab3
Gas5	Gabarap
Gas8	Gabarapl1
Gast	Gabarapl2
Gata1	Gabbr1
Gata3	Gabra1
Gatad2a	Gabra2
Gatad2b	Gabra6
Gatm	Gabrb1
Gba	Gabrb3
Gbf1	Gabrd
Gbl	Gabre
Gbp1	Gabrg1
Gbp11	Gabrq
Gbp2	Gabrr1
Gbp3	Gabrr2
Gbx1	Gad2
Gbx2	Gadd45a
Gca	Gadd45b
Gchfr	Gadd45g
Gcn5l2	Gadd45gip1
Gcnt2	Gal
Gcnt3	Galc
Gcnt4	Gale
Gcs1	Galk1
Gda	Galm
Gdap10	Galns
Gdap1l1	Galnt1
Gdi2	Galnt10
Gdpd3	Galnt11
Gdpd5	Galnt13
Gemin4	Galnt16
Gemin7	Galnt2
Gene ID	Galnt4
Gfer	Galnt6
Gfi1b	Galnt7
Gfpt2	Galntl1
Gfra2	Galntl5
Gga1	Galr2
Ggnbp1	Galr3///Gcat
Ggnbp2	Gamt
Ggps1	Ganab
Ggtl3	Gap43
Ghrh	Gapdh
Gigyf2	Gareml
Gip	Gas1
Gipc2	Gas2
Git1	Gas5
Giyd2	Gas5///Snord47
Gja10	Gas6
Gja7	Gas8
Gjb2	Gast
Gjc1	Gata1
Gkap1	Gata2
Glb1l	Gata3
Glb1l3	Gata5
Glce	Gatad1
Gldn	Gatad2b
Glg1	Gatm
Gli1	Gatsl2
Gli3	Gba2
Glipr2	Gbas
Glis1	Gbf1
Glis2	Gbgt1
Glmn	Gbp2
Glo1	Gbp3
Glod4	Gbp4///Gbp8
Glp1r	Gbp6
Gls2	Gbp7
Glt8d2	Gbp8
Glt8d4	Gbx2
Gltscr1	Gca
Gltscr2	Gcap8
Glul	Gcc2
Gm10053	Gcdh
Gm10069	Gcet2
Gm10073	Gcg
Gm10087	Gch1
Gm10126	Gck
Gm10180	Gckr
Gm10193	Gcm1
Gm10233	Gcm2
Gm10237	Gcnt1
Gm10294	Gcnt2
Gm10351	Gda
Gm10384	Gdap1
Gm10462	Gdap10
Gm10565	Gdap1l1
Gm10812	Gde1
Gm11042	Gdf10
Gm11065	Gdf11
Gm11131	Gdf15
Gm11172	Gdf2
Gm11201	Gdf5
Gm11229	Gdf9
Gm11256	Gdi1
Gm11274	Gdi2
Gm11291	Gdnf
Gm11292	Gdpd3
Gm11370	Gdpd5
Gm114	Gem
Gm11411	Gemin8
Gm11426	Gfap
Gm11437	Gfi1
Gm11462	Gfi1b
Gm11477	Gfpt1
Gm11525	Gfpt2
Gm11566	Gfra1
Gm1157	Gfra2
Gm11627	Gfra3
Gm11648	Gga1
Gm11649	Ggact
Gm11672	Ggct
Gm11692	Ggcx
Gm11707	Ggnbp2
Gm11744	Ggps1

Gm11761	Ggt1
Gm11774	Ggt5
Gm11792	Ggt7
Gm11803	Gh
Gm11958	Ghdc
Gm12019	Ghr
Gm12107	Ghrh
Gm12225	Ghrhr
Gm12243	Ghrl
Gm12369	Gif
Gm12397	Gimap1
Gm12466	Gimap4
Gm12532	Gimap6
Gm12579	Gimap7
Gm12592	Ginm1
Gm12597	Gipc1
Gm12639	Gipc2
Gm12646	Gipc3
Gm12676	Git2
Gm12681	Gja1
Gm12714	Gja10
Gm12743	Gja3
Gm12780	Gja8
Gm12786	Gjb1
Gm12852	Gjb2
Gm12913	Gjb3
Gm12974	Gjb5
Gm13003	Gjb6
Gm13033	Gjc1
Gm13062	Gjc2
Gm13201	Gjc3
Gm13203	Gjd2
Gm13207	Gk2
Gm13218	Gkap1
Gm13229	Gkn1
Gm13313	Gkn2
Gm13334	Gla
Gm13380	Glb1
Gm13394	Glb1l
Gm13402	Glcci1
Gm13446	Gldc
Gm13540	Gldn
Gm13553	Gli1
Gm13666	Gli2
Gm13667	Gli3
Gm13699	Glipr1l2
Gm13802	Glipr2
Gm13818	Glis1
Gm13860	Glis2
Gm13889	Glo1
Gm13930	Glra1
Gm13998	Glra4
Gm14006	Glrx
Gm14010	Glrx2
Gm14129	Glt25d1
Gm14260	Glul
Gm14337	Glyat
Gm14383	Glycam1
Gm14389	Glyctk
Gm14458	Gm10015///Gm10145///Gm10705///Gm5858///Ube2l3
Gm14471	Gm10058///Gm10096///Gm10147///Gm10230///Gm10486///Gm10487///Gm10488///Gm14525///Gm14632///Gm
Gm14472	Gm10094///Sap18
Gm14486	Gm10193///Zfp706
Gm14502	Gm10228
Gm14594	Gm10229
Gm14612	Gm10256///Gm10352///Gm21677///Gm21693///Gm21704///Gm21708///Gm4064///Rbmy
Gm14625	Gm10340///Gm10406///Gm2888///Gm2897///Gm3005///Gm3095///Gm3115///Gm3164///Gm3173///Gm3239///
Gm14780	Gm10340///Gm10409///Gm16525///Gm2897///Gm3002///Gm3005///Gm3095///Gm3115///Gm3164///Gm3173///
Gm14808	Gm10349///Gm9386///Nutf2///Nutf2-ps1
Gm14876	Gm10400
Gm14886	Gm10436///Gm2042///Oog1
Gm14933	Gm10439///Gm15080///Gm15085///Gm15093///Gm15107///Gm15114///Gm15128///Luzp4///Ott
Gm14940	Gm10664
Gm14983	Gm10817
Gm15051	Gm10997
Gm15101	Gm11149
Gm15333	Gm11397///Serpinb9e///Serpinb9f///Serpinb9g
Gm15459	Gm11437
Gm15477	Gm11567
Gm15480	Gm11767
Gm15527	Gm11787///Lyn
Gm15529	Gm11944
Gm15613	Gm12264
Gm15708	Gm12606
Gm15716	Gm12617///Gm4802
Gm1574	Gm12816///Gm5879///Rpl3
Gm15742	Gm12942///Zmym6
Gm15774	Gm13102///Pramel4
Gm15803	Gm13139
Gm15854	Gm13152
Gm15856	Gm13271///Gm13272///Gm13275///Gm13276///Gm13277///Gm13278///Gm13279///Gm13283///Gm13285///Gm
Gm15880	Gm13363///Ptp4a1
Gm15958	Gm13583
Gm15975	Gm13669///Plp2
Gm16047	Gm13770///Gm2573///Wdr83os
Gm16053	Gm13889
Gm16079	Gm13999
Gm16142	Gm14277///Snrpd1
Gm16234	Gm14680///Sms
Gm16303	Gm15270
Gm16433	Gm15393///Gm15393
Gm16508	Gm15455///Rbm10
Gm16523	Gm15483///LOC102642137///Rps13///Rps13-ps4
Gm16546	Gm1549
Gm16834	Gm15498
Gm16854	Gm15713
Gm16862	Gm15776///Gng5
Gm16983	Gm16340///Ifi203///LOC100862473
Gm17025	Gm16340///Ifi203///LOC100862473///LOC102641031
Gm17029	Gm16343///Gm16343
Gm17043	Gm16489
Gm17177	Gm1653
Gm17184	Gm17066///Rbm26
Gm17194	Gm17344///Gm17344
Gm17213	Gm17748///Ppih
Gm17276	Gm1976
Gm17294	Gm20172
Gm17401	Gm20274
Gm17407	Gm20362
Gm17439	Gm20559
Gm17440	Gm20737///Gm20772///Gm20773///Gm20777///Gm20793///Gm20795///Gm20807///Gm20827///Gm20831///Gm
Gm17529	Gm20738///Gm20747///Gm20791///Gm20799///Gm20801///Gm20803///Gm20804///Gm20805///Gm20806///Gm
Gm17538	Gm20767
Gm17544	Gm2083///Mup1///Mup10///Mup12///Mup13///Mup14///Mup15///Mup16///Mup19///Mup2///Mup7///Mup8///
Gm17608	Gm21098///Rhox4a///Rhox4b///Rhox4c///Rhox4d///Rhox4e///Rhox4f///Rhox4g
Gm17636	Gm21320///LOC100048884///Mup1///Mup10///Mup11///Mup14///Mup16///Mup19///Mup2///Mup3///Mup9
Gm17709	Gm21451///Loxl2
Gm19031	Gm21540///Tmed2
Gm2022	Gm21596///Gm6115///Hmgb1///LOC102635075
Gm20443	Gm26568
Gm20539	Gm266
Gm216	Gm2682
Gm2199	Gm2a
Gm2244	Gm3362///Gm6177///Gm8112///Gm8137///Gm8158///Gm8264///Gm8290///Rpl23a
Gm239	Gm3417///Gm3448///Tcte3
Gm2822	Gm3470///Rbpms2
Gm2959	Gm3579
Gm3460	Gm3716
Gm3608	Gm3837///Pdcd5
Gm3828	Gm4024///Tmed10
Gm3834	Gm4120///Gm4983///Gm9292///LOC101055823
Gm4262	Gm428
Gm443	Gm4354
Gm444	Gm4736///Gm8882///Prb1///Prpmp5
Gm4461	Gm4887///Twf1
Gm4609	Gm5415
Gm4732	Gm5451///Rpl9
Gm4798	Gm5481///Gm6109///Gm6570///Gm7429///Rpl30
Gm4968	Gm5526///Gm8894///LOC102643184///Myl6

Gm5058	Gm5595
Gm5138	Gm5607
Gm525	Gm561
Gm5292	Gm568
Gm5447	Gm5802
Gm5544	Gm6211
Gm5546	Gm6277
Gm5551	Gm6682///Tuba1a
Gm561	Gm6750///Gm7931///Gm9525///Hmgn2
Gm572	Gm6793///Hnrnpa3
Gm5755	Gm6910///Rybp
Gm6020	Gm7040///Prl2c2///Prl2c3///Prl2c4
Gm603	Gm7040///Prl2c2///Prl2c3///Prl2c4///Prl2c5
Gm608	Gm8479
Gm6097	Gm8539///Zbtb8os
Gm6320	Gm885
Gm6344	Gm9159
Gm6356	Gm9222
Gm6429	Gm9706
Gm6565	Gm9706///Isg15
Gm6568	Gm9757
Gm6659	Gm9780///Plac9a///Plac9b
Gm6721	Gm9798
Gm6741	Gm9817
Gm6846	Gm9839
Gm6923	Gm9840///Rbx1
Gm694	Gm9905
Gm7206	Gmcl1
Gm7237	Gmfb
Gm7358	Gmppa
Gm7589	Gmppb
Gm784	Gmpr
Gm7851	Gna11
Gm807	Gna12
Gm8250	Gna14
Gm8444	Gnai2
Gm854	Gnao1
Gm879	Gnaq
Gm8835	Gnas
Gm9105	Gnat1
Gm920	Gnat2
Gm9244	Gnaz
Gm944	Gnb1
Gm9530	Gnb4
Gm960	Gnb5
Gm9727	Gne
Gm9742	Gng10
Gm9758	Gng11
Gm9836	Gng13
Gm9917	Gng2
Gmeb1	Gng3
Gmfg	Gng4
Gmpr2	Gng8
Gna-rs1	Gngt1
Gna11	Gnl2
Gna13	Gnpda2
Gnai1	Gnpnat1
Gnai3	Gnrhr
Gnb1l	Gns
Gnb2	Golga3
Gnb2l1	Golm1
Gnb3	Golph3
Gnb5	Golph3l
Gne	Golt1a
Gng12	Golt1b
Gng7	Gopc
Gngt2	Gorasp2
Gnl2	Gosr2
Gnmt	Got2
Gnpnat1	Gp1ba
Gns	Gp1bb///Sept5
Golm1	Gp2
Golph3	Gp49a /// Lilrb4
Golph3l	Gp49a///Lilrb4
Got1l1	Gp9
Gpa33	Gpa33
Gpaa1	Gpaa1
Gpatch2	Gpam
Gpbp1	Gpank1
Gpc4	Gpatch2
Gpc6	Gpatch4
Gpha2	Gpc1
Gphn	Gpc2
Gpr107	Gpc3
Gpr108	Gpc4
Gpr125	Gpc6
Gpr128	Gpc6 /// LOC100045283
Gpr142	Gpcpd1
Gpr15	Gpd1
Gpr152	Gpd2
Gpr157	Gpm6a
Gpr162	Gpm6b
Gpr177	Gpr108
Gpr19	Gpr109a
Gpr21	Gpr110
Gpr39	Gpr12
Gpr6	Gpr124
Gpr84	Gpr132
Gpr89	Gpr133
Gpr98	Gpr137b
Gprc5c	Gpr137b///Gpr137b-ps
Gps1	Gpr143
Gps2	Gpr146
Gpsm2	Gpr153
Gpt2	Gpr162
Gramd1b	Gpr165
Gramd1c	Gpr177
Gramd3	Gpr180
Gramp3	Gpr182
Grb14	Gpr3
Grb2	Gpr31b
Grcc10	Gpr37
Greb1	Gpr37l1
Grhl2	Gpr63
Grhl3	Gpr65
Grid2	Gpr83
Grifin	Gpr85
Grik2	Gpr87
Grin2a	Gprasp1
Grin2d	Gprasp2
Grinl1a	Gprc5b
Grip1	Gprc5c
Gripap1	Gprc6a
Grk4	Gprin1
Grk5	Gps1
Grlf1	Gpsm1
Grm6	Gpsm3
Grm7	Gpt2
Grm8	Gpx1
Grm8A	Gpx2
Grn	Gpx3
Grpel1	Gpx4
Gse1	Gpx5
Gsta1	Gpx6
Gsta4	Gpx7
Gstm1	Gpx8
Gsx1	Gramd1a
Gtdc1	Grap2
Gtf2a1	Grasp
Gtf2h2	Grb10
Gtf2h4	Grb14
Gtf2i	Grb2
Gtf2ird1	Grb7
Gtf2ird2	Grcc10
Gtf3c2	Greb1l
Gtf3c6	Grem1
Gtl-13	Grem2
Gtl3	Grhl1
Gtpbp5	Grhl1 /// LOC100043996

Gtpbp6	Grhl2
Guca1a	Grhl3
Gyltl1b	Gria1
Gzmb	Gria2
H13	Gria3
H1f0	Gria4
H1foo	Grid1
H2-Bl	Grid2
H2-DMa	Grid2ip
H2-Ke6	Grik5
H2-T23	Grin1
H2-T24	Grin2b
H2-T3	Grin2d
H2afj	Grin3a
H2afx	Grin3b
H2afz	Grk1
H3f3a	Grk4
H3f3b	Grk5
H60b	Grk6
H6pd	Grm1
HADH	Grm8
HAS3	Grp
HDGF2	Grpr
HEATR1	Grsf1
HIGD1A	Grtp1
HIRIP3	Grwd1
HIST1H2BK	Gsdmc///Gsdmc2
HIST2H2AA3	Gsg2
HIST2H2BF	Gsn
HIST2H3A#HIST2H3C	Gspt2
HLA-DMA	Gsr
HLA-DMB	Gsta3
HLA-DOA	Gsta4
HLA-DPA1	Gstm1
HLA-DPB1	Gstm2
HLA-DQA1	Gstm5
HLA-DRA	Gstm6
HMBOX1	Gsto2
HMGN4	Gstp1
HNRNPA1L2	Gstt1
HNRNPK	Gstt2
HSPA1B///HSPA1A	Gstz1
HSPA4L	Gsx2
Haao	Gt(ROSA)26Sor
Hace1	Gtdc2
Hadha	Gtf2a1
Hadhb	Gtf2e2
Hagh	Gtf2f1
Haghl	Gtf2h4
Hamp	Gtf2i
Hamp2	Gtf2ird1
Hapln4	Gtf2ird2
Has2	Gtf3c1
Has3	Gtl3
Hax1	Gtpbp1
Hbegf	Gtpbp3
Hbp2	Gtpbp4
Hcfc2	Gtpbp6
Hcn2	Gtpbp8
Hcn3	Gtse1
Hcn4	Gtsf1l
Hdac1	Guca1a
Hdac10	Guca1b
Hdac5	Guca2b
Hdc	Gucy1a3
Hdgfrp2	Gucy1b3
Hdgfrp3	Gulo
Hdhd3	Gusb
Hdlbp	Gyg
Heatr2	Gyk
Heatr5a	Gykl1
Heatr6	Gyltl1b
Hectd2	Gypa
Helb	Gypc
Helt	Gys1
Helz	Gzma
Hephl1	Gzmb
Herc1	Gzmd
Herc2	Gzme
Herc4	Gzmf
Hes1	Gzmk
Hes7	Gzmm
Hexim1	Gzmn
Hexim2	H13
Hfe	H19
Hgd	H1f0
Hgfl	H1foo
Hhat	H1fx
Hhip	H2-Aa
Hic1	H2-Ab1
Hif1an	H2-Ab1 /// LOC641240 /// Rmcs2 /// Rmcs5
Higd1a	H2-Ab1 /// Rmcs2 /// Rmcs5
Higd1b	H2-D1
Hip1	H2-D4
Hipk2	H2-DMb1///H2-DMb2
Hipk3	H2-DMb2
Hira	H2-Ea
Hist1h1a	H2-Ea-ps
Hist1h1c	H2-Eb1
Hist1h1d	H2-Ke6
Hist1h2ab	H2-Ob///LOC102633400
Hist1h2ae	H2-Q10
Hist1h2ag	H2-Q2
Hist1h2ah	H2-T10///H2-T22///H2-T9
Hist1h2ai	H2-T24
Hist1h2bb	H2afj
Hist1h2be	H2afy2
Hist1h2bf	H2afy2///H2afy3
Hist1h2bg	H2afy3
Hist1h2bh	H6pd
Hist1h2bj	Habp2
Hist1h2bk	Habp4
Hist1h2bl	Hacl1
Hist1h2bm	Hal
Hist1h2bn	Hamp2
Hist1h3a	Hand1
Hist1h3b	Hand2
Hist1h3c	Hapln1
Hist1h3h	Hapln2
Hist1h4a	Hapln3
Hist1h4b	Has1
Hist1h4d	Haus6
Hist1h4f	Haus7
Hist1h4i	Hax1
Hist1h4k	Hba-a1
Hist1h4n	Hba-a1///Hba-a2
Hist2h2bb	Hba-x
Hist2h3b	Hbb-b1///Hbb-b2///Hbb-bs///Hbb-bt
Hist2h4	Hbb-b2
Hist3h2a	Hbb-bh1
Hist3h2ba	Hbb-y
Hist3h2bb	Hbs1l
Hist4h4	Hc
Hivep2	Hcar2
Hk1	Hcfc1
Hk2	Hck
Hlf	Hcn1
Hm13	Hcn2
Hmbox1	Hcn3
Hmbs	Hcst
Hmg20a	Hdac2
Hmga1	Hdac4
Hmga2	Hdac6
Hmgcl	Hdac7
Hmox1	Hdac9
Hmx2	Hdc
Hnf1b	Hddc2
Hnrnpa1	Hdgfl1
Hnrnpa2b1	Hdgfrp2

Hnrnpl	Hdgfrp3
Hnrpa3	Hdhd1a
Hnrpab	Hdlbp
Hnrpd	Hdx
Hnrpdl	Heatr1
Hnrpf	Heatr2
Hnrph1	Heatr5b
Hnrph3	Heatr8
Hnrpm	Hebp1
Hnrpul1	Helb
Hnt	Hells
Homer2	Hemk1
Homez	Heph
Hook2	Herc1
Hormad1	Herc2
Hoxa1	Herc4
Hoxa10	Herpud1
Hoxa11	Herpud2
Hoxa2	Hes1
Hoxa3	Hes2
Hoxa4	Hes3
Hoxa5	Hes5
Hoxa9	Hes7
Hoxb1	Hesx1
Hoxb2	Hexa
Hoxb3	Hexdc
Hoxb4	Hey1
Hoxb5	Hey2
Hoxb6	Heyl
Hoxb7	Hfe
Hoxb8	Hgf
Hoxb9	Hgsnat
Hoxc10	Hhex
Hoxc11	Hhip
Hoxc4	Hiat1
Hoxc5	Hic1
Hoxc6	Hic2
Hoxc8	Hid1
Hoxc9	Hif1a
Hoxd1	Hif3a
Hoxd10	Higd1b
Hoxd3	Higd1c///Mettl7a1///Mettl7a2///Mettl7a2Higd1c///Mettl7a3
Hoxd8	Hint1
Hoxd9	Hip1
Hpca	Hip1r
Hrbl	Hipk1
Hrh1	Hipk2
Hrk	Hipk3
Hrsp12	Hira
Hs2st1	Hist1h1c
Hs3st1	Hist1h1e
Hs3st3b1	Hist1h1t
Hs3st5	Hist1h2ab /// Hist1h2ac /// Hist1h2ad /// Hist1h2ae /// Hist1h2ag /// Hist1h2ai /// Hist1h2an /// Hist1h2ao /// RP23-
Hsd17b1	Hist1h2bb /// Hist1h2bc /// Hist1h2be /// Hist1h2bf /// Hist1h2bg /// Hist1h2bj /// Hist1h2bl /// Hist1h2bm /// Hist1
Hsd17b4	Hist1h2bc///Hist1h2be///Hist1h2bf///Hist1h2bg///Hist1h2bj///Hist1h2bl///Hist1h2bm///Hist1h2bn///Hist1h2bp///H
Hsdl2	Hist1h3a///Hist1h3b///Hist1h3c///Hist1h3d///Hist1h3e///Hist1h3f///Hist1h3g///Hist1h3h///Hist1h3i///Hist2h3b///H
Hsf2bp	Hist1h3b///Hist1h3c///Hist1h3d///Hist1h3e///Hist1h3f///Hist2h3b///Hist2h3c1///Hist2h3c2
Hsf5	Hist1h3f
Hsp90ab1	Hist1h4a /// Hist1h4b /// Hist1h4c /// Hist1h4d /// Hist1h4f /// Hist1h4h /// Hist1h4i /// Hist1h4j /// Hist1h4k /// Hist
Hsp90b1	Hist1h4a///Hist1h4b///Hist1h4c///Hist1h4d///Hist1h4f///Hist1h4h///Hist1h4i///Hist1h4j///Hist1h4k///Hist1h4m///H
Hspa1a	Hist2h2aa2
Hspa2	Hist3h2ba///Hist3h2bb-ps
Hspa4	Hk1
Hspa5	Hlf
Hspa8	Hlx
Hspa9	Hmbs
Hspb6	Hmcn1
Hspb7	Hmg20a
Hspb8	Hmga1 /// Hmga1-rs1
Hspbap1	Hmga2
Hspbp1	Hmga2-ps1
Hspc171	Hmgcl
Hspd1	Hmgcll1
Hspe1	Hmgcr
Hspg2	Hmgcs2
Htr6	Hmgn3
HtrA2	Hmmr
Htra2	Hmox1
Hunk	Hmox2
Hus1b	Hmx1
Hyal1	Hn1
Hyal2	Hnf1a
Hyal3	Hnf4a
Hyou1	Hnf4g
ICAM1	Hnmt
ID2	Hnrnpa1
ID3	Hnrnpa2b1
IFI16	Hnrnpab
IFIT2	Hnrnpc
IFITM1	Hnrnph1
IFITM2	Hnrnph3
IFNGR1	Hnrnpl
IGFN1	Hnrnpu
IGHG3	Hoga1
IGJ	Homer1
IGKC	Homer3
IGLC2	Hook2
IGLL1	Hopx
IKZF1	Hormad1
IL10	Hoxa10
IL13RA2	Hoxa11os
IL16	Hoxa2
IL28A	Hoxa9
IL8	Hoxb7///Hoxb8
ILF2	Hoxb9
INHBC	Hoxc13
INPP5D	Hoxc4
INPPL1	Hoxc5
INTS8	Hoxc6
IRF3	Hoxc8
IRF8	Hoxc9
ISG15	Hoxd1
ISG20	Hoxd10
ITGAL	Hoxd12
ITGB7	Hoxd13
IVNS1ABP	Hoxd3
Iars2	Hoxd3///Hoxd4
Ibrdc3	Hoxd8
Ibtk	Hoxd9
Icam1	Hp
Icam2	Hpd
Icam4	Hpgd
Icam5	Hpgds
Ick	Hpn
Icosl	Hps1
Id1	Hpvc-ps
Id2	Hpx
Id3	Hrasls
Id4	Hrg
Idh3b	Hrh1
Idi1	Hrh2
Ier2	Hrh4
Ier5	Hrnr
Ier5l	Hs1bp3
Ifi35	Hs2st1
Ifih1	Hs3st1
Ifit1	Hs3st3a1
Ifna13	Hs3st3b1
Ifrd2	Hs6st2
Ifrg15	Hs6st3
Ift122	Hsbp1
Ift74	Hsd11b1
Ift81	Hsd11b2
Igf1r	Hsd17b1
Igf2	Hsd17b10
Igf2bp1	Hsd17b11
Igf2bp2	Hsd17b3
Igf2bp3	Hsd17b6
Igfbp4	Hsd17b7

Ighv6-6	Hsd3b1
Igkv1-135	Hsd3b2
Igkv10-95	Hsd3b3
Igkv2-112	Hsf2
Igll1	Hsf4
Igsf8	Hsp90b1
Ihpk1	Hspa12b
Ik	Hspa1l
Ikzf2	Hspa4
Ikzf4	Hspa4l
Il11	Hspa5
Il15ra	Hspa8
Il17a	Hspa9
Il17re	Hspb1
Il18bp	Hspb11
Il1r1	Hspb6
Il1rap	Hspb7
Il25	Hspe1-ps2///Hspe1-ps2
Il2ra	Hspg2
Il33	Hsph1
Il4i1	Htatsf1
Il6ra	Htr1a
Ilf2	Htr1b
Ilf3	Htr1f
Imp3	Htr2b
Impa2	Htr3a
Impdh1	Htr3b
Impdh2	Htr4
Ing2	Htr5a
Inhba	Htr6
Ino80	Htr7
Inoc1	Htra1
Inpp1	Htra2
Inpp4a	Htra3
Inpp5a	Hus1
Inppl1	Huwe1
Insc	Hvcn1
Insl3	Hyou1
Insm1	Hypm
Ints2	I54
Ints6	Iapp
Invs	Ibsp
Ip6k1	Ibtk
Ipo8	Ica1
Ipp	Icam2
Iqcb1	Icam4
Iqcc	Icam5
Iqce	Ick
Iqcg	Icos
Iqgap1	Icosl
Iqgap2	Ict1
Iqsec1	Id2
Iqwd1	Ide
Irak2	Idh2
Irf1	Idh3g
Irf6	Idi1
Irf8	Ido1
Irgm2	Ido2
Irs1	Ids
Irx4	Idua
Isg20l1	Ier5
Isgf3g	Ier5l
Isl1	Iffo1
Isl2	Iffo2
Isoc2b	Ifi202b
Itfg2	Ifi204
Itga10	Ifi204///Ifi205///Mnda///Mndal
Itga2	Ifi205///Mnda
Itga3	Ifi27l2b
Itga6	Ifi35
Itgav	Ifi44
Itgb1	Ifi44l
Itgb5	Ifi47
Itgb7	Ifih1
Itgb8	Ifit2
Itih5l-ps	Ifitm1
Itm2c	Ifitm2
Itpk1	Ifitm3
Itpka	Ifna1
Itpkb	Ifna1///Ifna12///Ifna15///Ifna16///Ifna5///Ifna6///Ifna7///Ifna9///Ifnab
Itpr1	Ifna11
Itpr2	Ifna15///Ifna6///Ifnab
Itpr3	Ifna2
Ivns1abp	Ifna4
JSAP2	Ifna5
Jag1	Ifnab
Jagn1	Ifnar1
Jak2	Ifnar2
Jak3	Ifnb1
Jam3	Ifng
Jarid1a	Ifngr2
Jarid1c	Ifnz
Jazf1	Ifrd2
Jmjd1a	Ift122
Jmjd1b	Ift20
Jmjd2c	Ift22
Jmjd3	Ift27
Jmjd4	Ift57
Jmjd6	Ift74
Jmy	Ift80
Jnk2	Ift81
Jph1	Igbp1b
Jph3	Igdcc3
Jph4	Igdcc4
Jtb	Igf1
Jub	Igf1os
Junb	Igf1r
Jund1	Igf2
Jundm2	Igf2bp1
Jup	Igf2bp2
KBTBD4	Igf2bp3
KCNH8	Igf2os
KCNQ1	Igf2r
KIAA1680	Igfals
KIAA1712	Igfbp3
KIF18A	Igfbp4
KLF10	Igfbp5
KLHDC9	Igfbp6
KPNB1	Igfbp7
KRT10	Igfbpl1
KRT85	Igh-VJ558
Kalrn	Igh-VJ558///Igha
Kars	Igh-VJ558///Ighg///Ighm
Kat2b-ps	Ighg
Kbtbd12	Ighm
Kcna1	Ighmbp2
Kcna7	Ighv14-2
Kcnb1	Igj
Kcnd2	Igk
Kcng4	Igk-V1///Igkv9-120
Kcnh2	Igk-V28
Kcnh3	Igk-V28///Igkc///Igkj1///Igkv4-53///Igkv6-23///Igkv8-30
Kcnh8	Igk-V28///Igkv6-14///Igkv6-23///Igkv8-19
Kcnip1	Igkv1-117
Kcnip3	Igkv19-93
Kcnj1	Igkv4-72
Kcnj2	Igkv4-73///Igkv4-81
Kcnj4	Igkv5-39
Kcnj8	Igkv6-15
Kcnk1	Igkv8-30
Kcnma1	Iglc1///Iglv1
Kcnn1	Igll1
Kcnn2	Iglv1
Kcnq4	Igsf1
Kcns3	Igsf10
Kctd11	Igsf8
Kctd17	Igtp
Kctd2	Iigp1
Kctd20	Iigp1b

Kdelc1	Ikbip
Kdelc2	Ikbkap
Kdelr1	Ikbkb
Kdelr3	Ikbke
Kdm2b	Ikbkg
Kdm3a	Ikzf1
Kdm5a	Ikzf2
Kdm6b	Ikzf3
Keap1	Ikzf4
Khdrbs1	Il10
Khk	Il10ra
Khsrp	Il11
Kiaa0286	Il12a
Kiaa1815	Il12b
Kif11	Il13
Kif13a	Il13ra2
Kif17	Il15ra
Kif1b	Il16
Kif1c	Il17a
Kif20a	Il17b
Kif23	Il17d
Kif24	Il17ra
Kif26a	Il17rd
Kif2a	Il17re
Kif5c	Il18
Kif9	Il18bp
Kifc1	Il18r1
Kifc2	Il1b
Kirrel	Il1f10
Kiss1r	Il1f5
Klc1	Il1f9
Klf3	Il1r1
Klf5	Il1r2
Klf7	Il1rap
Klf9	Il1rapl1
Klhdc3	Il1rapl2
Klhdc5	Il1rl1
Klhdc8b	Il1rl2
Klhl18	Il1rn
Klhl20	Il21
Klhl21	Il21r
Klhl23	Il22///Iltifb
Klhl25	Il23a
Klhl26	Il24
Klhl5	Il25
Klrb1b	Il27ra
Klrb1c	Il2ra
Klrb1d	Il2rb
Klrb1f	Il2rg
Klrg2	Il3ra
Kntc1	Il4
Kpna4	Il4i1///Nup62-il4i1
Kpnb1	Il5ra
Kras	Il6
Kremen1	Il6ra
Krt10	Il6st
Krt15	Il7
Krt18	Il7r
Krt20	Ilf2
Krt28	Ilf3
Krt8	Ilk
Krtap20-2	Image:619641
Krtap5-5	Immp1l
Krtcap2	Imp4
Ksr1	Impa1
Ksr2	Impact
L3mbtl3	Impad1
LCK	Impg1
LDB1	Ina
LDHA	Inca1
LGALS1	Incenp
LGMN	Ing3
LINS1	Ing4
LMCD1	Inha
LMO2	Inhba
LOC436177	Inhbb
LOC545291	Inhbe
LOC574418	Inmt
LOC622404	Ino80
LOC644974	Ino80b
LRMP	Ino80e
LRRC41	Inpp1
LYN	Inpp4a
LYRM7	Inpp5a
LYSMD1	Inpp5b
LYSMD4	Inpp5e
Lag3	Inpp5k
Lama3B	Ins1
Lamb2	Insl6
Lamc1	Insr
Lamc3	Insrr
Lamp1	Ints5
Lamr1-ps1	Ints6
Lancl2	Ints8
Lap1B	Invs
Laptm4a	Ip6k1
Laptm5	Ipo4
Large	Ipo4///Tm9sf1
Larp4	Ipo5
Larp7	Ipo8
Lars	Ipo9
Las1	Iqcb1
Lasp1	Iqcf3
Lass2	Iqgap1
Lat2	Iqgap2
Lats1	Iqsec1
Lats2	Irak1
Lba	Irak1bp1
Lba1	Irak4
Lbr	Irf1
Lbx2	Irf3
Lbxcor1	Irf4
Lca5	Irf6
Lca5l	Irf8
Lcat	Irgm1
Lck	Irs2
Lclat1	Irs3
Lcn14	Irs4
Lcor	Irx1
Lct	Irx4
Ldb1	Irx6
Ldhb	Iscu
Ldlrad2	Isg20
Ldlrap1	Isl1
Lef1	Islr
Lefty2	Isoc2b
Lemd1	Isyna1
Lemd2	Itch
Lenep	Itga2
Leo1	Itga2b
Leprel2	Itga4
Leprotl1	Itga5
Lfng	Itga7
Lgals1	Itga8
Lgals2	Itga9
Lgals4	Itgal
Lgr4	Itgam
Lgr5	Itgav
Lgtn	Itgax
Lhb	Itgb1
Lhfp	Itgb1bp1
Lhfpl2	Itgb1bp2
Lhx1	Itgb2
Lhx2	Itgb3bp
Lhx4	Itgb4
Lhx5	Itgb5
Lhx8	Itgbl1
Lif	Itih2
Lig3	Itih3

Lim2	Itih4
Lima1	Itih5
Limch1	Itk
Limd1	Itm2a
Lime1	Itm2c
Limk1	Itpr1
Lin28	Itpr2
Lin37	Itsn1
Lin52	Ivd
Lin54	Iyd
Lipg	Izumo4
Lipt1	JAM2
Llgl1	Jade1
Llgl2	Jag1
Lman1	Jak1
Lman2	Jak2
Lmbr1l	Jam2
Lmln	Jam3
Lmna	Jarid2
Lmnb2	Jdp2
Lmo4	Jhdm1d
Lmo7	Jkamp
Lmtk3	Jmjd1c
Lnpep	Jmjd6
Lnv	Jmy
Lnx2	Josd2
Loh12cr1	Jph2
Loxl3	Jph3
Lpcat3	Junb
Lpgat1	Kalrn
Lphn2	Kank2
Lpin3	Kank3
Lpxn	Kansl1
Lrba	Kansl1l
Lrch1	Kansl2
Lrfn1	Kap
Lrig1	Kat2a
Lrig2	Kat2b
Lrig3	Kat6b
Lrp1	Kat7
Lrp10	Kazn
Lrp1b	Kbtbd10
Lrp4	Kbtbd11
Lrp8	Kcna3
Lrrc16a	Kcna4
Lrrc20	Kcna5
Lrrc23	Kcna7
Lrrc25	Kcnab1
Lrrc27	Kcnab2
Lrrc28	Kcnab3
Lrrc29	Kcnb1
Lrrc3b	Kcnb1///Pacsin2
Lrrc41	Kcnc1
Lrrc45	Kcnc3
Lrrc46	Kcnc4
Lrrc4b	Kcnd1
Lrrc4c	Kcnd2
Lrrc59	Kcnd3
Lrrc6	Kcne1l
Lrrc61	Kcne2
Lrrc66	Kcne3
Lrrc8b	Kcne4
Lrrc8e	Kcnh2
Lrrk2	Kcnh3
Lrrn1	Kcnh7
Lrrtm1	Kcnip1
Lrrtm3	Kcnip2
Lrsam1	Kcnip4
Lsamp	Kcnj1
Lsg1	Kcnj10
Lsm11	Kcnj11
Lsm2	Kcnj12
Lsm3	Kcnj14
Lsr	Kcnj2
Lss	Kcnj3
Lta4h	Kcnj5
Ltb4r1	Kcnj6
Ltbp-3	Kcnj8
Ltbp2	Kcnj9
Ltbp3	Kcnk1
Ltbp4	Kcnk10
Ltbr	Kcnk13
Ltv1	Kcnk3
Luc7l2	Kcnk5
Ly6a	Kcnma1
Ly6g6c	Kcnmb1
Lyn	Kcnmb2
Lypla2	Kcnmb4
Lyrm1	Kcnn1
Lyrm2	Kcnn2
Lyrm5	Kcnn3
Lyst2	Kcnn4
Lzts1	Kcnq1
M-rdgB beta	Kcnq2
M6pr	Kcnq3
M6prbp1	Kcnq4
MAGEA1	Kcns1
MAGI2	Kcns2
MAN1C1	Kctd1
MAP2K1	Kctd12///Mir5130
MAP3K11	Kctd12b
MARK2	Kctd14
MCFD2	Kctd17
MCM4	Kdelc1
MCM7	Kdelr2
MCM7///MCM2	Kdelr3
MEIS1	Kdf1
MFAP4	Kdm1b
MGAT2	Kdm5b
MGLL	Kdm5c
MGST2	Kdm5d
MINK	Kdm6a
MKK7	Kdm7a
MKP-7	Kdm8
MMP1	Kdr
MMP2	Keap1
MOK	Kel
MORN2	Kera
MORN4	Khdc1b
MRPL4	Khdrbs1
MRPS23	Khdrbs2
MS4A2///MS4A1	Khdrbs3
MSP58	Kif11
MTHFD2	Kif12
MTHFS	Kif13a
MTMR11	Kif13b
MVP	Kif17
MYBL1	Kif1a
MYBL2	Kif1b
MYC	Kif1c
MYL6	Kif22
MYO1B	Kif26b
MYO1E	Kif2a
Mab21l2	Kif2c
Macf	Kif3a
Macf1	Kif3b
Macrod1	Kif5a
Mad1	Kif5c
Mad1l1	Kifap3
Mad2l2	Kifc2
Madd	Kifc3
Maff	Kifc5b
Mageb18	Kiss1r
Mageb3	Kit
Magi1	Kitl
Magi3	Kl
Magmas	Klb
Magoh	Klc1
Magohb	Klc3

Maml3		Klf1
Man1a		Klf12
Man2a1		Klf13
Man2c1		Klf14
Manbal		Klf17
Map2k1		Klf7
Map2k7		Klf9
Map3k1		Klhdc2
Map3k10		Klhl10
Map3k11		Klhl11
Map3k14		Klhl13
Map3k3		Klhl14
Map3k7		Klhl20
Map4k1		Klhl22
Map4k3		Klhl24
Map4k4		Klhl25
Mapk1		Klhl36
Mapk12		Klhl42
Mapk14		Klhl5
Mapk1ip1l		Klhl7
Mapk3		Klk1
Mapk4		Klk11
Mapk7		Klk1b1
Mapk8ip2		Klk1b11
Mapk9		Klk1b16
Mapkap1		Klk1b21
Mapkapk3		Klk1b22///Klk1b9
Mapre2		Klk1b4
Mapt		Klk1b5
	Mar-02	Klk1b8
	Mar-04	Klk4
	Mar-06	Klk6
	Mar-07	Klk7
	Mar-08	Klkb1
	Mar-09	Klra1
Marcks		Klra1///Klra22
Marcksl1		Klra12
Mark2		Klra15
Mark4		Klra16
Marveld1		Klra18
Mast1		Klra2
Mast2		Klra21
Mast4		Klra21///Klra8
Mastl		Klra7
Mat1a		Klrb1b
Mat2a		Klrb1b///Klrb1c
Matr3		Klrc1
Max		Klrc2
Mbc2		Klrc2///Klrc3
Mbd3		Klrc3
Mbd4		Klrk1
Mbd5		Kmt2d
Mbd6		Kng1
Mbip		Knop1
Mboat5		Knstrn
Mcam		Kpna1
Mcart1		Kpna4
Mcee		Kpna6
Mcf2l		Kpnb1
Mcfd2		Kras
Mcl1		Kremen1
Mcm3		Krr1
Mcm3ap		Krt1
Mcm5		Krt10
Mcm8		Krt12
Mcm9		Krt13
Mcoln2		Krt14
Mcoln3		Krt15
Mcrs1		Krt16
Mctp1		Krt17
Mcts1		Krt19
Mcts2		Krt20
Mds1		Krt23
Me1		Krt25
Med1		Krt26
Med12		Krt27
Med12l		Krt28
Med13		Krt33b
Med13l		Krt35
Med16		Krt36
Med18		Krt5
Med20		Krt6a
Med21		Krt71
Med24		Krt72
Med28		Krt73
Med29		Krt75
Med31		Krt77
Med8		Krt79
Mef2b		Krt8
Mef2d		Krt84
Megf10		Krt85 /// LOC100044179
Megf11		Krtap1-5
Megf8		Krtap12-1
Meis2		Krtap13
Meis3		Krtap13-1
Mel13		Krtap15
Mepce		Krtap16-1
Mesdc1		Krtap16-3
Mesdc2		Krtap17-1
Met		Krtap19-1
Metap2		Krtap19-3
Mett11d1		Krtap19-4
Mettl1		Krtap19-5
Mettl3		Krtap19-9b
Mettl5		Krtap21-1
Mettl6		Krtap28-13
Mettl8		Krtap3-2 /// Krtap3-3
Mex3d		Krtap3-2///Krtap3-3
Mfap1a		Krtap4-2
Mfi2		Krtap5-1
Mfng		Krtap5-2
Mfsd11		Krtap5-4
Mga		Krtap6-2
Mgat4b		Krtap6-3
Mgat5		Krtap6-5
Mgat5b		Krtap8-1
Mgrn1		Krtap9-3
Mib1		Krtcap2
Mical2		Krtdap
Micall1		Ksr1
Mid1		Kti12
Midn		Ky
Mier3		Kynu
Mif1		L1cam
Mink1		L3hypdh
Minpp1		LEPR
Mir10a		LOC100038947 /// LOC668101 /// Sirpb1
Mir140		LOC100040358 /// Zfp521
Mir194-1		LOC100041195 /// LOC100041874 /// LOC666442
Mir3105		LOC100043315
Mir3968		LOC100044094 /// LOC100044314 /// Xlr3a /// Xlr3b /// Xlr3c
Mir7-2		LOC100044106 /// Plxnc1
Mir760		LOC100044139 /// Snrpn /// Snurf
Mir802		LOC100044234 /// Nptx2
Mir804		LOC100045677 /// Mcm3
Mitf		LOC100047619 /// Slc7a5
Mki67		LOC100047803 /// Ntsr1
Mkl1		LOC100049077
Mknk2		LOC100653389
Mlf2		LOC100862468///Nhp2l1
Mlh1		LOC101055731///LOC101055745///Sytl3
Mlkl		LOC101055763
Mll1		LOC101055909///Sec61b
Mll3		LOC102631817
Mllt10		LOC102632747
Mllt11		LOC102633540
Mllt6		LOC102633720
Mllt7		LOC102636151
Mlstd2		LOC102638039

Mlx	LOC102638292
Mlxipl	LOC102638854
MmKIF17	LOC102640192
Mmachc	LOC102640359
Mmd	LOC102640476///Rnf2
Mme	LOC102641289///Tbc1d14
Mmp16	LOC102641785///Tcf20
Mmp28	LOC102641923///Srsf1
Mms19	LOC102641972///Ptprj
Mnat1	LOC102642017///Uap1
Mnk2	LOC102642893///Scd3
Mns1	LOC102643023///Mfap1a///Mfap1b
Mnt	LOC102643033
Moap1	LOC102643054
Mob1b	LOC102643254///Rps6///Rps6-ps4
Mob2	LOC171588
Mob3b	LOC269472
Mobkl2b	LOC381765 /// LOC665506 /// Tcrb-J
Mog	LOC493582
Mosc2	LOC545261
Mov10	LOC552901
Moxd1	LOC631639 /// Lonrf1
Mpdu1	LOC633966
Mphosph9	LOC640441 /// Thbs1
Mpi	LOC665506
Mpp2	LOC666466 /// Obox2
Mpp5	LOC671878 /// Sms
Mpp6	LOC677224
Mpped2	LOC677224 /// Ubash3b
Mpst	Lactb2
Mpzl2	Lad1
Mpzl3	Lag3
Mr1	Lair1
Mrc1	Lalba
Mrc2	Lama1
Mrcl	Lama2
Mre11a	Lama3
Mrg1	Lama4
Mrg2	Lama5
Mro	Lamb1
Mrp63	Lamb2
Mrpl10	Lamb3
Mrpl14	Lamc1
Mrpl17	Lamc2
Mrpl18	Lamc3
Mrpl20	Lamtor1
Mrpl27	Lamtor5
Mrpl34	Lancl2
Mrpl4	Lancl3
Mrpl40	Lao1
Mrpl45	Laptm4a
Mrpl48	Laptm5
Mrpl51	Larp6
Mrpl52	Lasp1
Mrpl53	Lat2
Mrps21	Lats2
Mrps23	Lats2///Xpo4
Mrps27	Lbh
Mrps28	Lbh /// LOC100048380
Mrps33	Lbp
Mrvi1	Lbx1
Ms4a12	Lbx2
Msh2	Lcat
Msh5	Lce1a1
Msi1	Lce1a2
Msi2	Lce1d
Msl2	Lce1f
Msra	Lce1g
Msrb3	Lce1h
Msx1	Lce1i
Msx3	Lcn2
Msy2	Lcn3
Mt1	Lcn4
Mt2	Lcp1
MtPolB	Lcp2
Mta2	Lct
Mta3	Ldb1
Mtap1a	Ldb2
Mtap1b	Ldb3
Mtap4	Ldhb
Mtap7d1	Ldhc
Mtap9	Ldlr
Mtbp	Ldlrad4
Mtch2	Ldoc1l
Mtdh	Leap2
Mtf2	Lect1
Mthfsd	Lef1
Mtmr12	Lefty1
Mtmr2	Leng8
Mtmr4	Lep
Mtpn	Lepr
Mtss1	Lepre1
Mttp	Leprel2
Mtvr2	Leprel4
Mtx1	Leprotl1
MuORC4	Letm1
Muc1	Letm2
Muc4	Lfng
Mus81	Lgals1
Mvp	Lgals12
Mxi1	Lgals2
Mybpc2	Lgals3bp
Myc	Lgals7
Mycbpap	Lgalsl
Mycn	Lgi1
Myd116	Lgr5
Myh9	Lgr6
Myl4	Lhcgr
Myl6	Lhfpl2
Myl6b	Lhfpl3
Mylc2b	Lhpp
Mylpf	Lhx1
Mynn	Lhx2
Myo10	Lhx3
Myo15b	Lhx5
Myo18a	Lhx6
Myo18b	Lhx9
Myo1c	Lif
Myo1e	Lig3
Myo1f	Limd1
Myo5b	Limd2
Myo6	Limk1
Myo9b	Lims1
Myohd1	Lims2
Myom1	Lin28a
Mypn	Lin7b
Myt1	Lincpint
Myzap	Lingo1
Mzt1	Lingo2
N4bp1	Lipa
NAPSB	Lipc
NDUFA5	Lipe
NEGR1	Lipf
NEU1	Lipg
NF1	Lipt2
NFE2L2	Lman1
NFIX	Lman1l
NFKBIA	Lmbr1
NHE8	Lmbrd1
NME7	Lmcd1
NOL3///MYC	Lmf1
NOL5A	Lmna
NPM1	Lmnb2
NR3C1	Lmo2
NRAS	Lmod1
NR_000003	Lmod2
NR_000004	Lmx1a
NR_001277	Lmx1b
NR_001460	Lnx1

NR_001462	Loh11cr2a
NR_002841	Lonp2
NR_002855	Lor
NR_002865	Lox
NR_002867	Loxl1
NR_002871	Loxl3
NR_002873	Loxl4
NR_002874	Lpar1
NR_002897	Lpar2
NR_002902	Lpar4
NR_003248	Lpcat1
NR_003293	Lpgat1
NR_003619	Lphn1
NR_003628	Lpin1
NTHL1	Lpin2
NUB1	Lpin3
NUCB1	Lpl
NUDT9	Lpp
NUP107	Lpxn
Nab2	Lrat
Naip3	Lrba
Nat11	Lrfn5
Nat13	Lrg1
Nat6	Lrig1
Nat9	Lrmp
Nav2	Lrp1
Nbea	Lrp1b
Nbeal1	Lrp2
Nbeal2	Lrp3
Nbl1	Lrp5
Nbn	Lrp6
Nbr1	Lrp8
Ncapd2	Lrpap1
Ncapd3	Lrr1
Ncdn	Lrrc15
Ncf1	Lrrc2
Nckipsd	Lrrc20
Ncln	Lrrc4
Ncoa1	Lrrc46
Ncoa3	Lrrc49
Ncoa7	Lrrc51
Ncor1	Lrrc58
Ncstn	Lrrc59
Ndrg3	Lrrc6
Ndrg4	Lrrc8a
Ndufa2	Lrrc8a///Phyhd1
Ndufa4	Lrrc8c
Ndufa9	Lrrc8e
Ndufaf1	Lrrfip1
Ndufaf4	Lrriq4
Ndufb8	Lrrk2
Ndufs2	Lrrn1
Ndufs8	Lrrn2
Nebl	Lrrn3
Nedd4l	Lrrtm1
Nedd8	Lrrtm2
Nedd9	Lrrtm3
Nefh	Lrrtm4
Nefl	Lsamp
Nefm	Lsm2
Neil2	Lsm4
Nek1	Lsp1
Nek11	Lss
Nek7	Lst1
Nek8	Lta
Nek9	Lta4h
Nell2	Ltb
Nep	Ltb4r1
Nes	Ltbp1
Neu3	Ltbp2
Neud4	Ltbp3
Neurabin	Ltbp4
Neurod2	Ltc4s
Neurog1	Ltf
Neurog2	Ltn1
Nf1	Luc7l
Nfasc	Luc7l2
Nfat5	Luc7l3
Nfatc1	Lum
Nfe2	Luzp2
Nfe2l1	Lxn
Nfia	Ly6a
Nfib	Ly6c1///Ly6c2
Nfix	Ly6d
Nfkb1	Ly6h
Nfkb2	Ly6i
Nfkbil1	Ly75
Nfu1	Ly86
Ngdn	Ly96
Ngf	Lynx1
Nhej1	Lypd1
Nhlrc2	Lypd3
Nhsl1	Lyrm2
Nif3l1	Lyrm5
Ninj2	Lysmd2
Nip7	Lyst
Nipbl	Lyz1
Nipsnap1	Lztfl1
Nit1	Lztr1
Nkd1	Lzts2
Nkg7	M1ap
Nkiras1	MGST1
Nkx2-3	MRAS
Nkx2-5	MTHFR
Nkx6-1	Mab21l1
Nle1	Mab21l2
Nlk	Mad1l1
Nme1	Mad2l1
Nme2	Mad2l2
Nmi	Madd
Nmnat2	Maea
Nmt1	Maf
Nodal	Maf1
Nog	Mafb
Nol1	Maff
Nol12	Mafg
Nol14	Mag
Nol4	Magea1///Magea2///Magea3///Magea5///Magea6///Magea8
Nol5	Magea4
Nol6	Magea6
Nol8	Mageb1///Mageb2///Mageb3
Nola2	Mageb3
Nope	Maged1
Nos1	Maged2
Nos3	Magee1
Nosip	Mageh1
Notch1	Magel2
Notch4	Magi1
Nova1	Magi2
Npal3	Magi3
Npas2	Magt1
Npas3	Mak
Npas4	Mal
Npc2	Malat1
Npepl1	Mall
Npepps	Maml1
Nphp1	Maml2
Nphs1	Man1a
Npl	Man1a2
Nploc4	Man1b1
Npm1	Man2b1
Npm3	Man2b2
Npr1	Manba
Npr3	Manbal
Nptn	Maneal
Nptx1	Manf
Nr1d1	Maoa
Nr1d2	Maob
Nr1h2	Map1b

Nr1h3	Map1lc3a	
Nr1h4	Map1lc3b	
Nr1i2	Map1s	
Nr1i3	Map2k2	
Nr2c2	Map2k5	
Nr2e3	Map2k6	
Nr2f2	Map2k7	
Nr3c2	Map3k12	
Nr4a2	Map3k14	
Nr4a3	Map3k2	
Nr5a1	Map3k3	
Nr6a1	Map3k5	
Nramp2	Map3k6	
Nrarp	Map3k7	
Nrbf2	Map4	
Nrcam	Map4k1	
Nrd1	Map4k2	
Nrg1	Map4k5	
Nrgn	Map6	
Nrip1	Map7	
Nrip3	Map7d1	
Nrn1	Mapk10	
Nrp1	Mapk11	
Nrp2	Mapk14	
Nrxn1	Mapk8	
Nrxn2	Mapk8ip1	
Nrxn3	Mapk8ip2	
Nsddr	Mapk8ip3	
Nsdhl	Mapk9	
Nsf	Mapkap1	
Nsl1	Mapre1	
Nspl1	Mapre2	
Nsun4	Mapre3	
Nt5c1a	Mapt	
Nt5dc3		Mar-01
Ntan1		Mar-09
Ntf3	Marcks	
Ntf5	Marcksl1	
Ntn1	Mark1	
Ntng1	Mark2	
Ntrk3	Mark4	
Nua	Marveld1	
Nuak2	Marveld2	
Nub1	Marveld3	
Nucb1	Mas1	
Nudcd1	Masp1	
Nudt16l1	Masp2	
Nudt19	Mast1	
Nudt21	Mastl	
Nudt4	Mat1a	
Nudt5	Mat2a	
Nudt6	Matk	
Nudt9	Matn1	
Nufip1	Matn2	
Nufip2	Matn3	
Numb	Matn4	
Nup153	Mau2	
Nup160	Mavs	
Nup54	Maz	
Nup62	Mb	
Nwd1	Mbd1	
Nxn	Mbd2	
Nxnl2	Mbd3	
Nxph1	Mbd3l2	
O/E-4S	Mbd4	
OAS1	Mbl1	
OCIAD2	Mbl2	
OR56B4	Mboat2	
OR6K2	Mboat7	
ORC4L	Mbp	
ORF61	Mbtps1	
OSBPL3	Mc1r	
OTTMUSG00000000997	Mc2r	
OTTMUSG00000008561	Mc3r	
OTTMUSG00000015762	Mc5r	
OTTMUSP00000006088	Mcam	
Oas1b	Mcf2	
Oas3	Mcfd2	
Oasl2	Mcg1038069///Ube2n	
Oat	Mchr1	
Obox3	Mcm10	
Ocm	Mcm2	
Odc1	Mcm3	
Odf2l	Mcm5	
Odz2	Mcm7	
Ogdh	Mcm9	
Ogdhl	Mcoln3	
Ogfod1	Mcpt-ps1	
Ogfr	Mcpt4	
Ogg1	Mcpt8	
Oit1	Mcts1	
Ola1	Mdfic	
Olfm2	Mdga2	
Olfm3	Mdk	
Olfml2a	Mdn1	
Olfml3	Me1	
Olfr1010	Me2	
Olfr1359	Mea1	
Olfr1361	Mecom	
Olfr1420	Mecp2	
Olfr1507	Med1	
Olfr404-ps1	Med12l	
Olfr462	Med20	
Olfr463	Med21	
Olfr497	Med24	
Olfr512	Med29	
Olfr550	Med7	
Olfr721-ps1	Med9	
Olfr741	Medag	
Olfr782	Mef2a	
Olig3	Mef2b	
Onecut1	Mef2c	
Onecut2	Mef2d	
Optn	Meg3	
Orc1l	Meg3,Mir1906-2,Mir770	
Orc4l	Meg3///Meg3	
Orc6	Meig1	
Orc6l	Meis1	
Ormdl2	Meis2	
Ormdl3	Meis3	
Orp1	Mela	
Osbp	Men1	
Osbpl11	Meox1	
Osbpl1a	Meox2	
Osbpl7	Mesdc1	
Osbpl8	Mesdc2	
Osbpl9	Mest	
Osgep	Met	
Osgin1	Metap1d	
Osr1	Metrn	
Osr2	Metrnl	
Osta	Mettl1	
Otub2	Mettl13	
Otud4	Mettl20	
Otud7b	Mettl21a	
Otx1	Mettl6	
Otx2	Mettl7a1	
Ovca2	Mettl7a1///Mettl7a2	
Oxr1	Mettl7a2	
Oxsr1	Mfap2	
P140	Mfap3	
P2rx3	Mfap4	
P2rx4	Mfap5	
P2rxl1	Mfge8	
P4ha1	Mfi2	
P4hb	Mfng	
P7	Mfsd10	
PA2G4	Mfsd11	
PAG1///PRDX1	Mfsd4	

PAK3///CDKN1A	Mfsd6
PAPK-A	Mfsd6l
PARP1	Mfsd7b
PARP8	Mfsd7c
PAX5	Mfsd8
PBEF1	Mga
PBX2	Mgat3
PCDH18	Mgat4c
PCDHB16///ME1	Mgat5
PCDHGC3	Mgl1
PCNA	Mgll
PCSK4///SUB1///IFRD1	Mgme1
PE1	Mgmt
PEA15	Mgst1
PEX12	Mgst3
PHB	Mia
PHKG2	Mia2
PIGR	Mia3
PIK3CA	Mical1
PKD1L1	Mical2
PKMYT1	Mid1
PLEK	Mid1ip1
PLK1	Mid2
PMAIP1	Midn
PMP22///SP110	Mier2
PMS2L5	Mill2
PMUR10F	Mina
POFUT1	Minpp1
POLD1///CDC2	Miox
POLD2	Mip
POLR1A///RPA1	Mipol1
POM121L1P	Mir1188
POU2AF1	Mir143hg
POU2F2	Mir1897,Sox2ot
PPFIBP2	Mir369,Mir410,Mir412,Mirg
PPP5C	Mir425
PREP2	Mis12
PRIM1	Mitd1
PRKCB1	Mixl1
PRKDC	Mkks
PRKRIR	Mkln1
PRKWNK4	Mknk1
PRUNE	Mkrn1
PSMA4	Mkrn3
PSMB10	Mlana
PSMB8	Mlec
PSMB9	Mlf1
PTCH	Mlf2
PTDSS1	Mllt1
PTPN18	Mllt10
PTPN6	Mllt11
PTPRC	Mllt3
PVRIG	Mlph
PXMP4	Mlst8
Pa2g4	Mlx
Pabpn1	Mlxipl
Pacs1	Mmab
Padi1	Mmd2
Paf1	Mme
Pafah1b1	Mmel1
Pafah1b2	Mmgt2
Pafah1b3	Mmp11
Pafah2	Mmp12
Paip2b	Mmp13
Pak1	Mmp14
Pak7	Mmp15
Palm	Mmp16
Pals1	Mmp17
Pan3	Mmp19
Panx1	Mmp1a
Papd4	Mmp1b
Papola	Mmp2
Papolg	Mmp23
Par3	Mmp24
Parc	Mmp3
Pard3	Mmp7
Pard3b	Mmp8
Parg	Mmp9
Parl	Mndal
Parp11	Mns1
Parp14	Mnt
Parp3	Mnx1
Parp4	Mobp
Parp8	Mocs2
Parp9	Mog
Parva	Mok
Patz1	Morc3
Pawr	Morc4
Pax2	Morn3
Pax3	Morn4
Pax6	Mos
Pax7	Mospd2
Pbk	Mov10
Pbx3	Mov10l1
Pbxip1	Moxd1
Pcaf	Moxd2
Pcbd1	Mpdz
Pcbp1	Mpg
Pcbp2	Mphosph10
Pcdh1	Mphosph6
Pcdh10	Mphosph8
Pcdh7	Mphosph9
Pcdha4	Mpi
Pcdhga1	Mpl
Pcf11	Mpnd
Pcgf1	Mpp1
Pcgf2	Mpp3
Pcgf5	Mpp4
Pcgf6	Mpp5
Pcmt1	Mpp6
Pcnp	Mpped1
Pcnt	Mpped2
Pcnx	Mpv17l
Pcp4	Mpz
Pcp4l1	Mpzl2
Pcsk9	Mr1
Pctk2	Mrap
Pctk3	Mras
Pcyox1	Mrc1
Pcyt1a	Mrc2
Pdcd10	Mrgpra1
Pdcd11	Mrgpra4
Pdcd2	Mrgprb1
Pdcd6	Mrgprf
Pdcl	Mrgprh
Pde1b	Mri1
Pde3a	Mrpl10
Pde3b	Mrpl11
Pde4a	Mrpl15
Pde4d	Mrpl19
Pde4dip	Mrpl21
Pde5a	Mrpl23
Pde6b	Mrpl27
Pde6d	Mrpl3
Pde7b	Mrpl34
Pde9a	Mrpl35
Pdgfa	Mrpl38
Pdgfc	Mrpl41
Pdgfra	Mrpl42
Pdhx	Mrpl45
Pdia3	Mrpl49
Pdlim1	Mrpl51
Pdlim7	Mrps11
Pdp2	Mrps23
Pdrg1	Mrps24
Pds5a	Mrps25
Pds5b	Mrps33
Pdx1	Mrps34///Nme3
Pdxk	Mrps5

Pdzd2	Mrps6
Pdzd8	Mrs2
Pdzrn3	Mrvi1
Peli2	Ms4a1
Pemt	Ms4a10
Per1	Ms4a2
Perld1	Ms4a4b
Perq1	Ms4a4c
Pes1	Ms4a4d
Pex11a	Ms4a6c
Pex11b	Ms4a7
Pex12	Ms4a8a
Pex13	Msantd3
Pex14	Msc
Pex16	Msh2
Pex2	Msh3
Pex26	Msh4
Pex3	Msh5
Pex6	Msh6
Pfdn2	Msi1
Pfdn4	Msl1
Pfkfb2	Msl2
Pfkl	Msl3l2
Pfkm	Msln
Pfn1	Msmb
Pftk1	Msmo1
Pgam1	Msn
Pgk2	Msr1
Pglyrp1	Msrb2
Pgm1	Msx1
Pgm2l1	Msx2
Pgs1	Msx3
Phactr4	Mt1
Phb	Mt2
Phc1	Mt4
Phc3	Mta1
Phca	Mta2
Phex	Mta3
Phf10	Mtag2
Phf12	Mtap
Phf13	Mtch1
Phf14	Mtch2
Phf15	Mtcp1
Phf17	Mtdh
Phf19	Mterfd1
Phf21a	Mterfd2
Phf21b	Mterfd3
Phf23	Mtf1
Phf6	Mtfmt
Phip	Mtfp1
Phlda2	Mtfr1l
Phldb1	Mtg2
Phldb2	Mthfd2
Phospho1	Mthfr
Phospho2	Mtmr2
Phox2b	Mtmr3
Pi4k2a	Mtmr4
Pi4ka	Mtmr6
Pi4kb	Mtmr7
Pias1	Mtmr9
Pias3	Mtnr1a
Pid1	Mto1
Pig-n	Mtpn
Pigl	Mtrr
Pign	Muc13
Pigo	Muc15
Pigw	Muc3
Pigy	Muc4
Pigyl	Muc5b
Pih1d1	Mucl1
Pik3ip1	Mug-ps1///Mug1///Mug2
Pik3r3	Mug1
Pik4ca	Mul1
Pin1	Mum1l1
Pip4k2c	Mup4
Pip5k1a	Mup5
Pip5k1b	Mus81
Pipox	Musk
Pisd	Mustn1
Pitpnc1	Mut
Pitpnm1	Mutyh
Pitx2	Mvb12a
Pitx3	Mvd
Pkig	Mvk
Pkmyt1	Mx1
Pkn1	Mxd1
Pkn2	Mxd3
Pkn3	Mxd4
Pknox2	Mxi1
Pkp4	Mxra8
Pla1a	Myadm
Pla2g12a	Myb
Pla2g6	Myc
Plaa	Mycbpap
Plagl2	Mycl
Plcb2	Mycn
Plcd3	Mycs
Plcl1	Myct1
Plcl2	Myd88
Pldn	Myef2
Plek2	Myh1
Plekha1	Myh11
Plekha2	Myh2
Plekha4	Myh3
Plekha5	Myh4
Plekhf2	Myh6
Plekhg3	Myh7
Plekhg6	Myh8
Plekhh2	Myl1
Plekhh3	Myl10
Plekhj1	Myl2
Plekhm1	Myl4
Plin	Myl7
Plin1	Myl9
Plk1s1	Mylk
Plk2	Mylk2
Plod3	Mylpf
Plscr1	Mynn
Plscr2	Myo10
Plscr3	Myo15b
Pltp	Myo16
Plxdc1	Myo1b
Plxdc2	Myo1c
Plxnb1	Myo1f
Plxnb2	Myo1h
Plxnc1	Myo5c
Plxnd1	Myo6
Pml	Myo7a
Pmp22	Myoc
Pmpcb	Myocd
Pmvk	Myod1
Pnkp	Myof
Pnliprp1	Myog
Pnpla2	Myot
Pnpla7	Myoz1
Pnr	Myoz2
Pnrc2	Myrf
Pofut1	Myt1
Pogz	Myt1l
Pol gamma	Myzap
Pold1	Mzf1
Pold3	N6amt2
Pold4	NDNF
Poldip3	Naa10
Polg	Naa15
Polg2	Naa60
Polh	Naalad2
Poll	Nab2
Polm	Nabp1

Polr1e	Nabp2
Polr2a	Nacc1
Polr2h	Nacc2
Polr3gl	Naga
Polr3k	Naglu
Pomt2	Naip1
Pop1	Naip2
Pop5	Naip5
Pop7	Nampt
Popdc2	Nanog
Por	Nanos1
Pot1b	Nap1l1
Pou2af1	Nap1l2
Pou2f2	Nap1l3
Pou2f3	Nap1l5
Pou3f1	Napb
Pou3f2	Napsa
Pou3f3	Narf
Pou4f3	Nat3
Ppa2	Nat8
Ppap2b	Nat8l
Ppap2c	Nav2
Ppapdc3	Nav3
Pparg	Nbea
Ppargc1a	Nbl1
Ppargc1b	Ncald
Ppfibp1	Ncam1
Ppie	Ncam2
Ppil3	Ncan
Ppil4	Ncapg
Ppm1b	Ncbp2
Ppm1d	Ncdn
Ppm1e	Ncf1
Ppm1f	Ncf2
Ppm1g	Nck1
Ppm1h	Nck2
Ppm1j	Nckap1l
Ppm1l	Nckap5l
Ppm2c	Ncl
Ppme1	Ncln
Ppox	Ncmap
Ppp1cc	Ncoa2
Ppp1r11	Ncoa3
Ppp1r14d	Ncoa7
Ppp1r16a	Ncor2
Ppp1r1b	Ncs1
Ppp1r2-ps6	Ndn
Ppp1r37	Ndp
Ppp1r8	Ndph
Ppp1r9a	Ndrg3
Ppp1r9b	Ndrg4
Ppp2ca	Ndst1
Ppp2cb	Ndst3
Ppp2r1b	Ndst4
Ppp2r2d	Ndufa10
Ppp2r3c	Ndufa12
Ppp2r5a	Ndufa13///Yjefn3
Ppp2r5b	Ndufa2
Ppp2r5c	Ndufa7
Ppp2r5d	Ndufa9
Ppp3r1	Ndufab1
Ppp4c	Ndufaf3
Ppp4r1l-ps	Ndufaf4
Ppp4r2	Ndufb11
Pprc1	Ndufb4
Pptc7	Ndufb9
Pramef12	Ndufs5
Prcc	Ndufs8
Prcd	Nebl
Prcp	Necab3
Prdm1	Necap1
Prdm13	Nedd4
Prdm15	Nedd4l
Prdm16	Nedd9
Prdm2	Nefh
Prdm6	Nefl
Prdm8	Neil3
Prdm9	Nek3
Prdx1	Nek4
Prdx2	Nek6
Prdx3	Nek8
Prdx6	Nek9
Prelid2	Nelfcd
Prep	Nell1
Prg2	Nell2
Prhoxnb	Nemf
Prickle1	Nenf
Prickle2	Neo1
Primary Gene	Nepn
Prkaca	Nes
Prkag3	Nespas
Prkar2a	Neto1
Prkar2b	Neto2
Prkca	Neu1
Prkcbp1	Neu2
Prkcc	Neu3
Prkcd	Neurl4
Prkce	Neurod1
Prkci	Neurod4
Prkcn	Neurod6
Prkcq	Neurog2
Prkd2	Neurog3
Prkd3	Nf1
Prkg1	Nf2
Prl3d3	Nfam1
Prmt2	Nfat5
Prmt6	Nfatc2
Prmt8	Nfatc2ip
Prnd	Nfatc4
Prnp	Nfe2
Prodh2	Nfe2l2
Prok2	Nfia
Prom1	Nfib
Prom2	Nfic
Pros1	Nfil3
Prox1	Nfix
Prpf19	Nfkb2
Prpf3	Nfkbie
Prpf38a	Nfu1
Prpf38b	Nfx1
Prpf4	Nfxl1
Prpf40a	Nfya
Prpf4b	Ngb
Prph	Ngfr
Prps1	Ngp
Prpsap1	Nhedc1
Prr11	Nhedc2
Prr12	Nhlh1
Prr3	Nhp2
Prr6	Nhsl2
Prrc2b	Nicn1
Prrc2c	Nid1
Prrg2	Nid2
Prrg4	Ninj2
Prrt3	Nipa2
Prrxl1	Nisch
Prss16	Nit2
Prss36	Nkain1
Prss8	Nkain4
Prssl1	Nkap
Prtg	Nkapl
Prune	Nkd1
Prx	Nkd2
Psat1	Nktr
Psd	Nkx1-2
Psd3	Nkx2-1
Psip1	Nkx2-2
Psma1	Nkx2-4
Psma3	Nkx2-9

Psma4	Nkx3-2
Psma5	Nkx6-2
Psma7	Nle1
Psmb1	Nlgn1
Psmb10	Nlgn2
Psmb3	Nlk
Psmb6	Nlrp3
Psmb7	Nlrp4c
Psmb8	Nlrp5
Psmb9	Nlrp6
Psmbeta	Nmb
Psmc2	Nmd3
Psmc5	Nme1
Psmd11	Nme2
Psmd12	Nme4
Psmd7	Nmnat1
Psmd9	Nmrk1
Psme1	Nmt1
Psme2	Nnat
Psmf1	Nnmt
Psmg4	Nnt
Psors1c2	Nobox
Pspc1	Noc4l
Pstpip1	Nodal
Pstpip2	Nog
Ptcd2	Nol11
Ptch1	Nol3
Ptch2	Nol6
Ptcra	Nolc1
Ptdsr	Nono
Ptdss2	Nop16
Pten	Nop56
Ptger1	Nop58
Ptger2	Nop9
Ptgs2	Nos1
Ptma	Nos3
Ptms	Nosip
Ptp4a2	Notch3
Ptplad1	Notch4
Ptpn1	Notum
Ptpn12	Nov
Ptpn13	Nova1
Ptpn14	Nox4
Ptpn18	Npas1
Ptpn6	Npas2
Ptpn9	Npas3
Ptprb	Npas4
Ptprcap	Npcd///Nptxr
Ptprf	Npepps
Ptprh	Npffr2
Ptprj	Nphp1
Ptprk	Nphp3
Ptrf	Nphs1
Ptrh2	Nphs2
Pttg1ip	Npm3///Npm3-ps1
Pum1	Npnt
Pum2	Nppa
Punc	Nppb
Pura	Nppc
Purb	Npr2
Pus7l	Npr3
Pvrl1	Nptx2
Pvrl2	Npy
Pvt1	Npy1r
Pwwp2	Npy5r
Pxdn	Npy6r
Pxk	Nqo2
Pxmp4	Nr0b1
Pxn	Nr0b2
Pxt1	Nr1d1
Pycr2	Nr1d2
Pygo2	Nr1h3
Pyroxd1	Nr1h4
QRSL1	Nr1i3
Qars	Nr2c1
Qil1	Nr2c2
Qpct	Nr2e3
Qpctl	Nr2f1
Qprt	Nr2f2
Qrich1	Nr3c1
Qsox2	Nr3c2
RAB3IL1	Nr4a1
RABIF	Nr4a2
RAD23B	Nr4a3
RALY	Nr5a1
RAP1B	Nr5a2
RARRES3	Nrep
RASSF7	Nrf1
RB1	Nrg2
RBBP5	Nrg3
RBCK1	Nrg4
RBM25	Nrgn
RFX1	Nrip1
RFX5	Nrk
RGS18///RGS13	Nrl
RGS3	Nrn1
RHOH	Nrp1
RIC8A	Nrp2
RIOK2	Nrros
RLIM	Nrsn1
RNASEH2A///JUNB	Nrxn1
RNASEL	Nsa2
RNF13	Nsd1
RNF219	Nsdhl
RNF5P1	Nsg1
RNaseP_nuc	Nsmaf
RP23-113F14.3	Nsmce4a
RP23-349B16.3	Nsmf
RP23-381B19.7	Nsun4
RP23-391K14.2-001	Nsun5
RP23-445D13.2	Nt5c1b
RP23-467J12.2-001	Nt5c2
RP23-81E16.2-005	Nt5c3b
RP23-92B18.7	Nt5dc3
RP24-73K9.3	Nt5e
RPL10A	Ntm
RPL21	Ntmt1
RPS6	Ntn1
RPS6KA1	Ntn3
RTDR1	Ntn4
Rab11b	Ntng1
Rab11fip1	Ntrk2
Rab11fip4	Ntrk3
Rab13	Nts
Rab15	Ntsr1
Rab19	Ntsr2
Rab20	Nub1
Rab28	Nucb2
Rab2B	Nucks1
Rab2b	Nudc
Rab34	Nudcd1
Rab37	Nudcd1///Nudcd1
Rab38	Nudcd2
Rab3d	Nudcd3
Rab3il1	Nudt1
Rab4a	Nudt10///Nudt11
Rab5b	Nudt16
Rab5c	Nudt16l1
Rab6	Nudt4
Rab6ip1	Numb
Rab8A	Numbl
Rab8a	Nup133
Rab8b	Nup210
Rabep1	Nup35
Rabepk	Nup54
Rabgap1	Nup62
Rabgap1l	Nup98
Rabif	Nupl1
Rac3	Nupr1

Rad23a	Nus1
Rad51	Nusap1
Rad51ap1	Nwd1
Rad51c	Nxf2
Rad51l1	Nxf7
Rad54b	Nxn
Rad54l	Nxnl1
Rad9	Nxnl2
Rad9b	Nxph1
Raet1a	Nynrin
Raet1c	ORF63
Raet1e	OSR1
Raf1	OTTMUSG00000006457
Rag2	Oaf
Rage	Oas1a
Rai1	Oas1d///Oas1e
Rai12	Oas1e
Rai16	Oasl1
Ralbp1	Oasl2
Ralgps2	Oat
Raly	Oaz2
Ramp2	Obox1///Obox2
Ranbp17	Obox2
Ranbp3	Obox3
Rangap1	Obp1a
Rapgef2	Obp2a
Rapgef3	Obsl1
Rapgef5	Oc90
Raptor	Odc1
Rara	Odf1
Rarg	Odf2
Rars	Odf4
Rasa1	Odz2
Rasa3	Ofa
Rasa4	Ogfrl1
Rasal2	Ogn
Rasd1	Ogt
Rasgef1a	Oit1
Rasgrf1	Oit3
Rasl11a	Ola1
Rassf1	Olah
Rassf4	Olfm1
Rassf5	Olfm2
Rbbp8	Olfm3
Rbbp9	Olfml2b
Rbck1	Olfml3
Rbl1	Olfr140
Rbm13	Olfr15
Rbm14	Olfr1508
Rbm15	Olfr1509
Rbm15b	Olfr151///Olfr160
Rbm19	Olfr155
Rbm22	Olfr159
Rbm27	Olfr17
Rbm35a	Olfr480
Rbm4	Olfr49
Rbm43	Olfr544
Rbm4b	Olfr64
Rbm6	Olfr66
Rbm8a	Olfr67
Rbm9	Olfr672
Rbms2	Olfr68///Olfr69
Rbmxrt	Olfr690
Rbpj	Olfr71
Rbpms	Olfr74
Rbpms2	Olfr749
Rc3h2	Olfr78
Rcan1	Olig1
Rcc2	Olig2
Rcor1	Olig3
Rcor3	Olr1
Rdh10	Omd
Rdh13	Omg
Rdh5	Omt2a
Rdm1	Onecut1
Reep2	Onecut3
Reep3	Opcml
Refbp2	Ophn1
Relb	Opn1mw
Reln	Opn1sw
Rem2	Opn3
Reps1	Oprk1
Reps2	Oprl1
Rere	Oprm1
Rerg	Orc2
Rev3l	Orc4
Rexo2	Orm1
Rfc4	Orm2
Rfng	Orm3
Rftn2	Os9
Rfwd2	Osbpl1a
Rfwd3	Osbpl6
Rfx1	Osbpl9
Rfx3	Oscar
Rfx4	Osm
Rfx5	Osr1
Rfx7	Osr2
Rfxank	Ost4
Rfxdc2	Ostc
Rg9mtd1	Otc
Rg9mtd2	Otoa
Rgl1	Otof
Rgl2	Otog
Rgma	Otor
Rgmb	Otos
Rgnef	Otp
Rgs10	Otub1
Rgs14	Otud4
Rgs16	Otud5
Rgs20	Otud7a
Rgs7	Otud7b
Rhbdd2	Otx2
Rhbdd3	Ovca2
Rhbdl3	Ovol1
Rhd	Oxnad1
Rheb	Oxsm
Rhebl1	Oxtr
Rhobtb2	P2rx1
Rhoc	P2rx4
Rhod	P2ry12
Rhof	P2ry13
Rhot1	P2ry14
Rhou	P2ry4
Rhov	P2ry6
Rimbp3	P4ha1
Rimklb	P4ha2
Rims2	P4ha3
Ring1	P4hb
Rinl	PAGR1A
Ripply3	PAPPA
Rit2	PCBP3
Rkhd3	PIEZO2
Rlbp1	Pabpc1
Rmnd5a	Pabpc2
Rmnd5b	Pabpc5
Rnaseh1	Pabpn1
Rnaseh2a	Pacrgl
Rnasel	Pacs2
Rnasen	Pacsin1
Rnd1	Pacsin3
Rnd2	Padi1
Rnd3	Padi2
Rnf10	Padi3
Rnf113a2	Padi4
Rnf121	Pafah1b2
Rnf126	Pafah1b3
Rnf144b	Pag1
Rnf145	Pagr1a
Rnf157	Paip1

Rnf168	Pak1
Rnf187	Pak3
Rnf19b	Pak6
Rnf20	Pald1
Rnf220	Palm
Rnf26	Palmd
Rnf31	Pam
Rnf32	Pank3
Rnf43	Papolb
Rnh1	Pappa
Rnmtd2	Pappa2
Rnps1	Papss2
Robo1	Paqr4
Rock1	Paqr8
Rod1	Pard3
Rora	Pard6a
Rorb	Park2
Rp23-297j14.5	Parm1
Rpap2	Parp16
Rpl12	Parp3
Rpl13a	Parp6
Rpl15	Parp9
Rpl17	Parva
Rpl17-ps1	Parvb
Rpl18	Pate4
Rpl19	Patz1
Rpl21	Pax1
Rpl23	Pax3
Rpl23a	Pax4
Rpl27	Pax5
Rpl28	Pax6
Rpl29	Pax7
Rpl30-ps6	Pax9
Rpl32	Paxbp1
Rpl35a	Paxip1
Rpl36	Pbld1
Rpl37	Pbp2
Rpl37a	Pbsn
Rpl4	Pbx1
Rpl41	Pbx2
Rpl7	Pbx3
Rpl7l1	Pbx4
Rpl8	Pbxip1
Rpn1	Pcbd1
Rpn2	Pcbd2
Rpo1-3	Pcbp3
Rpp21	Pcbp4
Rprd1b	Pccb
Rps11	Pcdh10
Rps16	Pcdh11x
Rps2	Pcdh15
Rps25	Pcdh17
Rps25-ps1	Pcdh18
Rps26	Pcdh20
Rps27	Pcdh8
Rps27a	Pcdha1,Pcdha10,Pcdha11,Pcdha12,Pcdha2,Pcdha3,Pcdha4,Pcdha4-g,Pcdha5,Pcdha6,Pcdha7,Pcdha8,Pcdha9,Pcdhac1
Rps28	Pcdha1///Pcdha10///Pcdha11///Pcdha12///Pcdha2///Pcdha3///Pcdha4///Pcdha5///Pcdha6///Pcdha7///Pcdha8///P
Rps4x	Pcdha1///Pcdha10///Pcdha11///Pcdha12///Pcdha2///Pcdha4///Pcdha9///Pcdhac1///Pcdhac2
Rps6ka4	Pcdhb1
Rps6ka5	Pcdhb10
Rps6kb1	Pcdhb11
Rps6kl1	Pcdhb12
Rps7	Pcdhb13
Rpsa	Pcdhb14
Rragc	Pcdhb15
Rragd	Pcdhb16
Rras2	Pcdhb17
Rrm1	Pcdhb18
Rrm2	Pcdhb19
Rrp15	Pcdhb2
Rrp1b	Pcdhb20
Rrs1	Pcdhb21
Rsad2	Pcdhb22
Rsbn1	Pcdhb3
Rsf1	Pcdhb4
Rsph1	Pcdhb5
Rspo2	Pcdhb6
Rspry1	Pcdhb7
Rsrc2	Pcdhb8
Rtbdn	Pcdhb9
Rtbnd	Pcdhga1///Pcdhga10///Pcdhga11///Pcdhga12///Pcdhga2///Pcdhga3///Pcdhga4///Pcdhga5///Pcdhga6///Pcdhga7///
Rtcd1	Pcgf1
Rtkn	Pcgf2
Rtn2	Pcgf6
Rtn3	Pcid2
Rtn4	Pck1
Rtn4rl1	Pclo
Rtp3	Pcm1
Rufy1	Pcmt1
Rufy3	Pcmtd2
Rufy4	Pcnp
Rundc3a	Pcnx
Rundc3b	Pcnxl4
Runx1	Pcolce
Runx1t1	Pcp2
Rusc1	Pcp4
Rusc2	Pcp4l1
Rwdd2b	Pcsk1n
Rxrb	Pcsk2
Rxrip110	Pcsk5
Rybp	Pcsk6
Ryr2	Pctk2
S100A4	Pctp
S100A6	Pcx
S100b	Pcyt1a
S1pr3	Pcyt1b
SART3	Pcyt2
SASH1	Pdap1
SCARB1	Pdc
SDR39U1	Pdcd1
Sep-07	Pdcd6ip
SF3B14	Pdcl2
SGIP1alpha	Pde10a
SH2B3	Pde1a
SH3BP5L	Pde1b
SH3KBP1	Pde1c
SH3d19	Pde2a
SHC1	Pde3a
SHMT2	Pde3b
SIAH2	Pde4a
SLC25A21///ODC1	Pde4dip
SLC25A22	Pde6a
SLC25A37///MSC	Pde6b
SLC2A5	Pde6d
SLC7A11	Pde6g
SMG7	Pde7a
SMPD3	Pde7b
SMS	Pde8a
SNORA17	Pde8b
SNORA25	Pde9a
SNORA32	Pdgfb
SNORA50	Pdgfc
SNORA70	Pdgfd
SNORA71	Pdgfra
SNRPF	Pdgfrb
SOX4	Pdhb
SP1	Pdia3
SP100	Pdia4
SP110	Pdia5
SP140	Pdia6
SPANXE	Pdik1l
SPANXN5	Pdk2
SPATA4	Pdk3
SPC25	Pdk4
SPHK1a	Pdlim1
SPI1	Pdlim2
SPIB	Pdlim3
SREBF2	Pdlim4
SSR2	Pdlim5

ST3GAL1		Pdlim7
ST6GAL1		Pdpn
STAP1		Pdrg1
STAT6		Pdss1
SYK		Pdx1
Sacm1l		Pdxdc1
Sae1		Pdxk
Sall1		Pdxp
Sall2		Pdyn
Sall3		Pdzd11
Samd14		Pdzd3
Samd4		Pdzd9
Samd4b		Pdzk1
Samd5		Pdzrn3
Sap130		Pea15a
Sap30		Peak1
Sap30l		Pear1
Saps2		Pebp1
Sar1a		Pecam1
Sash1		Peg12
Sat2		Peg3
Sbf2		Pemt
Sbno2		Penk
Scamp2		Penk1
Scamp3		Per2
Scarb1		Per3
Scarb2		Perp
Sccpdh		Pet100
Scd1		Pet2
Scd2		Pex13
Scd3		Pex16
Scg5		Pex19
Scgn		Pex5
Schip1		Pex5l
Scin		Pex6
Scly		Pfdn1
Scml4		Pfdn5
Scn8a		Pfdn6
Scnn1a		Pfkfb1
Scotin		Pfkfb2
Scp2		Pfkfb3
Scrn2		Pfkl
Scube3		Pfkp
Scye1		Pfn2
Sdccag1		Pfn3
Sdccag10		Pfpl
Sdccag8		Pgam2
Sdf2		Pgc
Sdr39u1		Pgd
Sdsl		Pgf
Sec1		Pgk1
Sec11a		Pgk2
Sec11c		Pglyrp1
Sec14l2		Pgm1
Sec15l2		Pgm2l1
Sec16a		Pgp
Sec16b		Pgpep1
Sec23b		Pgr
Sec31b		Phactr1
Sec61g		Phactr2
Sec62		Phactr3
Sec63		Phc1
Sectm1b		Phex
Seh1l		Phf19
Sel1l3		Phf2
Sema3a		Phf20l1
Sema4a		Phf21a
Sema4b		Phf3
Sema4f		Phgdh
Sema5a		Phip
Sema5b		Phka1
Sema6a		Phka2
Semcap2		Phkg1
Semcap3		Phldb1
Senp1		Phldb2
Senp7		Phospho2
Senp8		Phox2a
	Sep-01	Phpt1
	Sep-02	Phxr1
	Sep-04	Phxr2
	Sep-07	Phxr4
	Sep-09	Phyhipl
	Sep-11	Phykpl
	Sep-15	Pi15
Sepw1		Pi16
Serbp1		Pi4k2a
Serf2		Pi4ka
Sergef		Pias2
Serinc2		Pibf1
Serinc3		Pigb
Serpinb6a		Pigc
Serpinb9		Pigk
Sertad1		Pign
Sesn2		Pigq
Set		Pigr
Setbp1		Pigu
Setd1b		Pigx
Setd2		Pik3ap1
Setd3		Pik3ca
Setd5		Pik3cd
Setd6		Pik3r1
Setd7		Pikfyve
Setdb2		Pilra
Sez4		Pilrb1
Sez6		Pim1
Sf3a2		Pim3
Sf3a3		Pink1
Sf3b2		Pinlyp
Sf3b5		Pinx1
Sf4		Pip
Sfmbt2		Pip4k2a
Sfpi1		Pip4k2b
Sfrp1		Pip4k2c
Sfrs1		Pip5k1a
Sfrs10		Pip5k1b
Sfrs14		Pip5k1c
Sfrs15		Pip5kl1
Sfrs2		Pipox
Sfrs3		Pira1///Pira11///Pira2///Pira5///Pirb
Sfrs4		Pithd1
Sfrs9		Pitpnb
Sfxn1		Pitpnc1
Sfxn2		Pitpnm1
Sgca		Pitpnm3
Sgcz		Pitx1
Sgip1		Pitx2
Sgk		Pitx3
Sgk269		Piwil2
Sgk3		Pja1
Sgms1		Pja2
Sgo1		Pkd1
Sgol1		Pkd2
Sgpl1		Pkd2l2
Sgpp1		Pkdcc
Sh2b1		Pkdrej
Sh2b3		Pkhd1
Sh2bpsm1		Pkhd1l1
Sh2d5		Pkia
Sh3bp5		Pkig
Sh3bp5l		Pklr
Sh3glb1		Pkmyt1
Sh3kbp1		Pkn1///Ptger1
Sh3md4		Pknox1
Sh3px3		Pknox2
Sh3pxd2a		Pkp1
Sh3rf2		Pkp3
Shank1		Pla1a
Shank2		Pla2g12a
Shbg		Pla2g15

Shc1	Pla2g1b
Shc2	Pla2g2d
Shc3	Pla2g2e /// Pla2g2f
Shc4	Pla2g2f
Shd	Pla2g6
Shf	Pla2g7
Shisa5	Pla2r1
Shmt1	Plaa
Shoc2	Plac1
Shq1	Plac8
Shroom2	Plag1
Si	Plagl1
Siah2	Plagl2
Sid470	Plau
Sidt1	Plbd2
Sin3a	Plcb1
Sipa1l1	Plcb2
Sipa1l3	Plcb4
Sirt1	Plce1
Sirt4	Plcg2
Sirt5	Plch2
Sirt6	Plcl2
Sit1	Plcz1
Six1	Pld1
Six3	Pld4
Six4	Plek
Six5	Plek2
Skap1	Plekha2
Ski	Plekha3
Skil	Plekha4
Skp1a	Plekha5
Slbp	Plekha6
Slc10a7	Plekha7
Slc11a1	Plekhb1
Slc12a2	Plekhf1
Slc12a4	Plekhg2
Slc12a5	Plekhh2
Slc12a7	Plekho1
Slc12a8	Plekhs1
Slc12a9	Plet1
Slc13a3	Plin2
Slc13a4	Plin5
Slc16a13	Plk1
Slc16a3	Plk2
Slc16a5	Plk3
Slc16a6	Plk4
Slc16a8	Pllp
Slc18a1	Pln
Slc1a4	Plod1
Slc22a15	Plod2
Slc22a17	Plod3
Slc23a2	Plp1
Slc23a3	Plrg1
Slc25a11	Pls1
Slc25a13	Plscr1
Slc25a2	Plscr2
Slc25a22	Plscr3
Slc25a30	Plscr4
Slc25a34	Plvap
Slc25a36	Plxna1
Slc25a38	Plxna3
Slc25a39	Plxna4
Slc25a41	Plxnc1
Slc25a42	Plxnd1
Slc25a44	Pm20d1
Slc25a46	Pmepa1
Slc26a8	Pmf1
Slc2a10	Pmfbp1
Slc2a13	Pml
Slc2a2	Pmm1
Slc2a3	Pmp22
Slc2a4	Pmpcb
Slc30a2	Pnck
Slc30a3	Pnlip
Slc30a7	Pnliprp1
Slc30a9	Pnliprp2
Slc31a1	Pnmt
Slc35a5	Pnn
Slc35b1	Pno1
Slc35d1	Pnoc
Slc35e4	Pnpla2
Slc35f1	Pnpla7
Slc35f5	Pnpt1
Slc37a1	Pnrc1
Slc37a2	Poc5
Slc37a3	Podxl
Slc38a1	Pof1b
Slc38a2	Pofut1
Slc39a1	Pofut2
Slc39a11	Pola2
Slc39a13	Pold2
Slc39a2	Pold4
Slc39a7	Pole
Slc44a1	Pole3
Slc45a3	Polg
Slc45a4	Polh
Slc4a1	Poli
Slc4a7	Polr1a
Slc4a9	Polr1d
Slc5a2	Polr2a
Slc5a4b	Polr2f
Slc6a1	Polr2l///Usp50
Slc6a4	Polr2m
Slc6a6	Polr3d
Slc6a9	Polr3e
Slc7a5	Polr3f
Slc7a7	Polr3gl
Slc7a8	Polr3k
Slc7a9	Pomc
Slc8a2	Pomk
Slc9a1	Pomt1
Slc9a3r2	Pon1
Slc9a8	Pop5
Slco2b1	Por
Slco5a1	Pou1f1
Slfn2	Pou2af1
Slk	Pou2f1
Slo	Pou3f4
Sltm	Pou4f3
SmX5	Pou6f1
Smad1	Ppa1
Smad3	Ppap2a
Smad5	Ppap2b
Smad7	Ppap2c
Smarca2	Ppapdc3
Smarcd2	Ppara
Smarcd3	Pparg
Smarce1	Ppargc1a
Smc6	Ppat
Smcr8	Ppbp
Smek2	Ppdpf
Smg5	Ppfia4
Smg6	Ppib
Smg7	Ppl
Smndc1	Ppm1a
Smurf1	Ppm1g
Smurf2	Ppm1m
Snap23	Ppox
Snap29	Ppp1cc
Snapc2	Ppp1r14a
Snapc5	Ppp1r14d
Sncaip	Ppp1r15b
Sned1	Ppp1r17
Snf8	Ppp1r18
Snip1	Ppp1r1a
Snord13	Ppp1r1b
Snord2	Ppp1r2
Snph	Ppp1r2-ps9
Snrp70	Ppp1r32

Snrpb	Ppp1r3a
Snrpd3	Ppp1r3c
Snrpe	Ppp1r42
Snrpn	Ppp1r8
Sntb1	Ppp2cb
Sntg2	Ppp2r1a
Snupn	Ppp2r2a
Snx11	Ppp2r2b
Snx13	Ppp2r2c
Snx14	Ppp2r3c
Snx16	Ppp2r3d
Snx17	Ppp2r4
Snx24	Ppp2r5b
Snx32	Ppp2r5c
Snx5	Ppp3cb
Snx7	Ppp3cc
Soat1	Ppp3r2
Socs1	Ppp4r2
Socs3	Ppp5c
Sod1	Ppp6r2
Son	Ppp6r3
Sorbs1	Pprc1
Sorcs2	Ppt1
Sos1	Ppt2
Sost	Ppy
Sowahb	Ppyr1
Sox12	Pqbp1
Sox13	Praf2
Sox4	Pramel6
Sox6	Pramel7
Sox9	Prap1
Sp1	Prcp
Sp110	Prdm1
Sp2	Prdm4
Sp4	Prdm5
Sp6	Prdx3
Sp7	Prdx4
Sp8	Preb
Spaca3	Prelid1
Spag5	Prelid2
Spag7	Prelp
Spag9	Prep
Sparc	Prepl
Spata13	Prg2
Spata20	Prg3
Spata5	Prg4
Spats2	Prickle1
Spef1	Prickle4///Tomm6
Speg	Prim1
Spen	Prima1
Spg21	Prkaa2
Sphk1	Prkab1
Spib	Prkacb
Spin4	Prkar1a
Spinkl	Prkar1b
Spint1	Prkca
Spire2	Prkcb
Spna2	Prkcb1
Spnb1	Prkcdbp
Spnb3	Prkce
Spns1	Prkcg
Sppl3	Prkci
Spred-2	Prkd1
Spred2	Prkd3
Spry2	Prkg1
Spsb1	Prkg2
Spsb2	Prkrir
Sptlc1	Prl2a1
Spty2d1	Prl3a1
Sqle	Prl3c1
Sqstm1	Prl3d1///Prl3d2
Srbd1	Prl4a1
Srcrb4d	Prl6a1
Srf	Prl7a2
Srgap2	Prl7d1
Srgap3	Prl8a2
Srp19	Prl8a6
Srp68	Prl8a9
Srp9	Prlr
Srpk2	Prm1
Srrm1	Prm2
Srsf3	Prm3
Ss18	Prmt1
Ss18l1	Prmt2
Ssbp3	Prmt3
Ssbp4	Prmt5
Ssfa2	Prmt6
Ssh2	Prn///Prnd
Ssh3	Procr
Ssrp1	Prok2
Sssca1	Prokr1
Sstr3	Prol1
St13	Prom1
St3gal1	Prop1
St3gal3	Pros1
St3gal4	Prosc
St3gal5	Proser2
St6gal1	Proz
St7	Prpf19
St8sia3	Prpf31
Stac2	Prpf38b
Stag1	Prpf39
Stam2	Prpf4b
Stap1	Prpf6
Stard10	Prph
Stard13	Prpmp5
Stard3	Prr15
Stat1	Prr16
Stat3	Prr5
Stat5A	Prrc1
Stat5a	Prrc2a
Stat5b	Prrg2
Stat6	Prrt1
Stau1	Prrx1
Stc2	Prrx2
Steap2	Prss12
Stim2	Prss2
Stip1	Prss21
Stk10	Prss22
Stk11ip	Prss23os
Stk25	Prss28
Stk36	Prss29
Stk38	Prss3
Stk38l	Prss30
Stk39	Prss32
Stk40	Prss35
Stoml1	Prss37
Stoml2	Prss39
Ston2	Prss44
Stra13	Prss58
Stra6	Prtg
Strbp	Prtn3
Strn	Prune
Strn3	Prx
Strn4	Psap
Stt3a	Pscdbp
Stt3b	Psd2
Stx12	Psd3
Stx16	Psen2
Stx17	Psg-ps1
Stx18	Psg16
Stx3	Psg17
Stx6	Psg18
Stxbp1	Psg19
Stxbp3a	Psg23
Stxbp4	Psip1
Styk1	Psma1
Sucla1	Psma5
Suclg1	Psma6

Suclg2	Psma8
Sufu	Psmb10
Sulf1	Psmb7
Sulf2	Psmc1
Sult1a1	Psmc3
Sult1c2	Psmc3ip
Sult2a5	Psmc4
Sumo1	Psmc5
Sumo3	Psmd11
Supl15h	Psmd4
Supt16h	Psmd6
Supt5h	Psmd7
Supt6h	Psme3
Susd3	Psme4
Suv420h1	Psmf1
Suv420h2	Pspc1
Suz12	Psph
Sv2c	Pspn
Syf2	Psrc1
Syk	Pstpip2
Syn3	Ptafr
Syne2	Ptbp1
Syngr2	Ptbp2
Syngr3	Ptcd1
Synj1	Ptch1
Synj2bp	Ptch2
Synrg	Ptchd4
Sypl2	Ptdss2
Syt12	Pten
Syt3	Pter
Syt6	Ptf1a
Syt7	Ptgdr2
TACC3	Ptgds
TAF6	Ptgds2
TAGLN3	Ptger3
TANK	Ptger4
TAPBP	Ptges
TBC1D22B	Ptges2
TCF7L1///TCF3	Ptgfr
TECPR1	Ptgfrn
THADA	Ptgir
TIP5	Ptgis
TIPR	Ptgr2
TIPRL	Ptgs1
TLN1	Ptgs2
TLR1	Pth
TLR10	Pth1r
TMEFF2	Pth2r
TMEM179B	Ptk2
TMEM205	Ptk6
TMEM39A	Ptk7
TMEM69	Ptms
TMEM79	Ptov1
TMF1	Ptp4a2
TMSB4X	Ptpla
TNFAIP3	Ptplad2
TNFRSF10B	Ptpn1
TNFSF9	Ptpn11
TNPO3	Ptpn12
TNRC6B	Ptpn18
TOX4///LCP1	Ptpn2
TPD52L1	Ptpn20
TRAF1	Ptpn4
TRAF5	Ptpn5
TRIM34	Ptpn6
TRIM44	Ptpn9
TRIP10	Ptprb
TSLP	Ptprc
TUBB	Ptprcap
TUBG1	Ptprd
TWF2	Ptpre
TXNIP	Ptprg
Tac4	Ptprj
Tacc1	Ptprk
Tacc2	Ptprm
Tacc3	Ptprn
Tada3l	Ptpro
Taf12	Ptprr
Taf15	Ptprs
Taf1c	Ptprt
Taf3	Ptpru
Taf4a	Ptprz1
Taf5	Ptrf
Taf5l	Ptrh1
Taf7	Pttg1
Tal1	Pttg1ip
Tanc1	Ptx3
Tanc2	Pura
Tank	Purg
Taok1	Pus3
Taok2	Pus7
Tap1	Pvalb
Tap2	Pvr
Tapbp	Pvrl3
Tapbpl	Pvrl4
Tardbp	Pvt1
Tatdn3	Pxk
Tax1bp3	Pycr1
Tbc1d1	Pycr2
Tbc1d10b	Pycrl
Tbc1d16	Pygl
Tbcd	Pygm
Tbk1	Pyhin1
Tbkbp1	Pyroxd2
Tbl3	Pzp
Tbp	Qk
Tbrg4	Qpct
Tbx1	Qpctl
Tbx15	Qprt
Tbx18	Qrsl1
Tbx2	Qsox1
Tbx20	Qtrt1
Tbx21	Qtrtd1
Tbx3	R74862
Tbxas1	RAB39B
Tc2n	RSPO4
Tcam1	RTL1
Tcea1	Rab11b
Tcea3	Rab12
Tceal8	Rab13
Tceb1	Rab14
Tcerg1l	Rab17
Tcf12	Rab18
Tcf15	Rab1b
Tcf19	Rab23
Tcf20	Rab24
Tcf3	Rab25
Tcf4	Rab27b
Tcf7l2	Rab28
Tcfap2a	Rab2a
Tcfap2c	Rab2b
Tcfap2d	Rab31
Tcfap2e	Rab32
Tcfcp2	Rab33a
Tcn2	Rab34
Tcp1	Rab3a
Tcp11	Rab3b
Tcte1	Rab3d
Tcte2	Rab3il1
Tdrd1	Rab40b
Tdrd6	Rab43
Tdrd7	Rab4a
Tead1	Rab4b
Tead3	Rab6a
Tead4	Rab6b
Tec	Rab7
Tera	Rab8b
Terf2ip	Rabac1
Tes	Rabep1

Tesk1	Rabgap1
Tesk2	Rabgap1l
Tessp2	Rabl3
Tet1	Rabl6
Tex14	Rac1
Tex19	Rac2
Tex2	Rad18
Tex21	Rad23a
Tfdp2	Rad50
Tfg	Rad51
Tfrc	Rad51ap1
Tgfb1	Rad51b
Tgfb1i1	Rad51d
Tgfb2	Rad52
Tgfb3	Rad54l
Tgfbr3	Rad9a
Tgif1	Rae1
Tgm2	Raet1a///Raet1b///Raet1c///Raet1d///Raet1e
Thada	Raf1
Thap2	Rag1
Thap3	Rai1
Thbs3	Rai2
Them2	Rala
Thnsl1	Ralb
Thra	Ralgapa1
Thrap2	Ralgps2
Thrap3	Raly
Thrap4	Ramp2
Thtpa	Ramp3
Thumpd3	Ran
Thy1	Ranbp1
Tia1	Ranbp10
Tial1	Ranbp17
Tiam1	Rangrf
Ticam1	Rap1gds1
Tigd3	Rap2a
Tigd4	Rap2b
Timm22	Rapgef3
Timm23	Rapgef4
Timp2	Rarb
Tinagl1	Rarg
Tinf2	Rarres2
Tipin	Rasa1
Titf1	Rasa3
Tjap1	Rasa4
Tjp2	Rasal2
Tk1	Rasal3
Tk2	Rasd1
Tlcd1	Rasd2
Tlk2	Rasgrf1
Tln1	Rasgrp2
Tlr3	Rasip1
Tlr5	Rasl11b
Tlx2	Rasl2-9
Tm4sf5	Rassf1
Tm7sf2	Rassf2
Tm7sf3	Rassf3
Tm9sf3	Rassf5
Tmc2	Rassf7
Tmc6	Rassf8
Tmc8	Rassf9
Tmcc1	Rbbp4
Tmcc3	Rbbp6
Tmco4	Rbfox1
Tmco6	Rbfox2
Tmed4	Rbks
Tmed5	Rbm12
Tmed8	Rbm14
Tmeff2	Rbm14-rbm4///Rbm4
Tmem102	Rbm18
Tmem106a	Rbm25
Tmem107	Rbm26
Tmem109	Rbm35a
Tmem11	Rbm35b
Tmem111	Rbm39
Tmem115	Rbm42
Tmem120a	Rbm47
Tmem120b	Rbm4b
Tmem123	Rbm5
Tmem129	Rbm6
Tmem132a	Rbms1
Tmem132c	Rbms2
Tmem138	Rbms3
Tmem140	Rbmx
Tmem141	Rbp1
Tmem144	Rbp2
Tmem145	Rbp3
Tmem149	Rbp4
Tmem150c	Rbp7
Tmem161a	Rbpj
Tmem161b	Rbpjl
Tmem162	Rbpms
Tmem168	Rc3h2
Tmem16f	Rcan1
Tmem16j	Rcan2
Tmem170	Rcan3
Tmem180	Rcbtb1
Tmem181b-ps	Rcbtb2
Tmem19	Rcc1
Tmem211	Rccd1
Tmem225	Rcl1
Tmem24	Rcn1
Tmem28	Rcn2
Tmem30b	Rcn3
Tmem33	Rcvrn
Tmem43	Rd3
Tmem46	Rdh10
Tmem49	Rdh14
Tmem50a	Rdh16
Tmem51	Reck
Tmem55a	Recql
Tmem59l	Reep1
Tmem63a	Reep3
Tmem63b	Reep5
Tmem66	Reep6
Tmem70	Reg2
Tmem79	Reg3a
Tmem82	Reg3b
Tmem87a	Reg3d
Tmem88	Rell1
Tmem91	Reln
Tmem93	Rem1
Tmem98	Ren1///Ren2
Tmod3	Renbp
Tmod4	Rep15
Tmpo	Repin1
Tmprss5	Rerg
Tmsb4x	Rest
Tmtc1	Ret
Tmtc2	Retnla
Tmtc3	Retnlb
Tmx4	Rev1
Tnfaip2	Rev3l
Tnfaip8	Rex2
Tnfaip8l3	Rexo1
Tnfrsf19	Rffl
Tnfrsf1a	Rfk
Tnfrsf25	Rfng
Tnfsf13b	Rfpl4
Tnfsf15	Rftn2
Tnfsf9	Rfx3
Tnip1	Rfxank
Tnk1	Rgl1
Tnk2	Rgl3
Tnks	Rgn
Tnks1bp1	Rgnef
Tnks2	Rgs1
Tnpo1	Rgs10
Tnpo3	Rgs16

Tnrc5	Rgs17
Tnrc6a	Rgs18
Tnrc6b	Rgs19
Tob1	Rgs2
Tob2	Rgs3
Tom1l2	Rgs4
Tom40	Rgs5
Tomm22	Rgs6
Tomm40	Rgs7
Top2a	Rgs7bp
Top2b	Rgs8
Top3a	Rhag
Top3b	Rhbdd2
Tor1a	Rhbdd3
Tor1aip1	Rhbdf1
Tor1aip2	Rhbdl3
Tox	Rhbg
Tox2	Rhcg
Tox4	Rhd
Tpbg	Rho
Tpcn2	Rhoa
Tpd52	Rhob
Tpi1	Rhoc
Tpm1	Rhoh
Tpm2	Rhoj
Tpm3	Rhoq
Tpm4	Rhot2
Tpp2	Rhou
Tpst2	Rhox2a///Rhox2e
Tpte	Rhox4b
Tpx2	Rhox5
Tra2a	Rhox6
Tra2b	Rhpn1
Traf2	Rian
Traf4	Rian///Rian///Rian
Traf5	Rictor
Traf6	Rilpl1
Traits	Rilpl2
Trak1	Rimklb
Trak2	Rims2
Trappc3	Rin2
Trappc4	Riok1
Trappc5	Riok2
Trem3	Ripk1
Treml4	Ripk3
Trerf1	Ripk4
Trh	Ripply3
Trhr2	Rit1
Trib1	Rit2
Trib3	Rlbp1
Trim14	Rlim
Trim17	Rln1
Trim2	Rmdn1
Trim21	Rmst
Trim23	Rnase2b
Trim26	Rnaseh2c
Trim31	Rnasel
Trim34a	Rnd3
Trim36	Rnf10
Trim37	Rnf11
Trim39	Rnf112
Trim41	Rnf114
Trim44	Rnf121
Trim45	Rnf128
Trim54	Rnf138
Trim56	Rnf138rt1
Trim7	Rnf141
Trim71	Rnf144a
Trim8	Rnf144b
Trio	Rnf146
Triobp	Rnf151
Trip10	Rnf157
Trip12	Rnf17
Trip4	Rnf183
Trip6	Rnf186
Trm6	Rnf187
Trmt2a	Rnf19b
Trmt5	Rnf2
Trmt6	Rnf20
Troap	Rnf207
Trove2	Rnf214
Trp2	Rnf219
Trp53	Rnf25
Trp53bp1	Rnf41
Trp53i11	Rnf43
Trp53i13	Rnf5
Trpc2	Rnf8
Trpc5	Rnft1
Trpc7	Rngtt
Trpm7	Rnpc3
Trpv3	Rnpep
Trub1	Rnps1
Tsarg7	Robo1
Tsc22d1	Robo3
Tsc22d4	Rock1
Tsen54	Rock2
Tsfm	Rom1
Tsg101	Ropn1
Tsga10	Ropn1l
Tshz1	Ror1
Tshz2	Ror2
Tspan12	Rora
Tspan13	Rorb
Tspan31	Rorc
Tspan33	Ros1
Tspan9	Rp1
Tspyl3	Rp2h
Tssk3	Rpe65
Tst	Rpgrip1
Ttbk2	Rpl12
Ttc1	Rpl18
Ttc13	Rpl27a
Ttc23	Rpl35
Ttc3	Rpl37a
Ttc39b	Rpl39l
Ttc4	Rpl3l
Ttc5	Rpl41
Ttf1	Rpl7
Ttf2	Rpn1
Ttk	Rpn2
Ttll10	Rpp25
Ttll13	Rpp30
Ttll3	Rprd1a
Ttll4	Rprd1b
Ttpal	Rprd2
Ttrap	Rprm
Ttyh2	Rps10
Tuba1a	Rps15
Tuba1b	Rps15a-ps6
Tuba1c	Rps18
Tuba3b	Rps24
Tuba4a	Rps27
Tuba8	Rps4l
Tubb3	Rps6
Tubb4	Rps6ka2
Tubd1	Rps6ka5
Tubg1	Rps6kc1
Tubgcp6	Rps6kl1
Tufm	Rps9
Tulp2	Rpsa
Tulp3	Rptn
Tulp4	Rpusd1
Tusc2	Rrad
Tusc3	Rragb
Tusc4	Rragd
Tut1	Rras
Twf1	Rrbp1
Txn1	Rrh
Txnip	Rrnad1

Txnl1	Rrp12
Txnrd1	Rrp1b
Txnrd3	Rrp36
Tysnd1	Rrp8
U1	Rrp9
U2	Rrs1
U20366	Rsad2
U20367	Rsph1
U20369	Rsph6a
U2af1l4	Rsph9
U2af2	Rspo1
U2af26	Rspo2
U5	Rspo3
U6	Rsrc1
U6atac	Rsrc2
U7	Rsrp1
U83174	Rsu1
U83175	Rtbdn
UBR2	Rtcb
UCP2	Rtfdc1
UFM1	Rtkn2
UQCRC2	Rtn1
UTP18	Rtn2
Uap1	Rtn3
Ubap2	Rtn4
Ubap2l	Rtn4ip1
Ubb	Rtp3
Ubc	Rtp4
Ube1l	Rufy1
Ube2b	Rufy2
Ube2d3	Rufy3
Ube2e1	Rundc3a
Ube2e2	Runx1
Ube2g1	Runx1t1
Ube2g2	Runx2
Ube2l3	Runx3
Ube2l6	Rusc1
Ube2q1	Rusc2
Ube2r2	Rwdd3
Ube2v1	Rwdd4a
Ube2z	Rxfp2
Ube4b	Rxrg
Ubl5	Ryk
Ubn1	Ryr2
Ubp1	S100a1
Ubqln2	S100a13
Ubr2	S100a14
Ubr5	S100a16
Ubtd1	S100a3
Ubtd2	S100a4
Ubtf	S100a6
Ubxd4	S100a8
Uchl5	S100a9
Uck2	S100b
Ucn	S100g
Ucp2	S100pbp
Ugt2b38	S1pr1
Ugt2b5	S1pr3
Uhrf1	SCARA5
Uimc1	SCUBE2
Unc13a	SLC26A6
Unc13b	SLC5A7
Unc45b	STAT2
Unc5c	Saa1
Unc93a	Saa2
Unc93b1	Saa3
Unk	Saa4
Upf2	Saal1
Upp1	Sac3d1
Uqcc	Sacs
Uqcrh	Sall1
Urm1	Sall2
Usf1	Sall4
Usf2	Samd14
Usmg5	Samd4
Usp12	Samhd1
Usp13	Samsn1
Usp14	Samt3
Usp18	Samt4
Usp19	Sap30bp
Usp20	Sapcd1
Usp21	Sar1a
Usp25	Sardh
Usp33	Sarnp
Usp34	Sars2
Usp39	Sart3
Usp46	Sash1
Usp47	Sat2
Usp48	Satb1
Usp49	Satb2
Usp5	Sav1
Uspl1	Sbf2
Ust	Sbp///Sbpl
Utp11l	Sbsn
Utp15	Sc5d
Utp20	Scaf11
Utrn	Scaf4
Uts2r	Scamp3
Uvrag	Scamp5
V1rf5	Scara3
VEGFA	Scara5
VNN2	Scarb1
VPREB3	Sccpdh
VRHO	Scd1
Vamp1	Scd2
Vamp2	Scd3
Vamp5	Scel
Vamp8	Scfd1
Vangl2	Scg2
Vapa	Scg3
Vars	Scgb1b27
Vars2	Scgb2b20///Scgb2b7
Vasn	Scgb3a2
Vasp	Scml4
Vat1	Scn10a
Vav1	Scn1a
Vav2	Scn1b
Vav3	Scn2a1
Vax1	Scn3a
Vax2	Scn3b
Vcan	Scn4b
Vcp	Scn7a
Vcpip1	Scn8a
Vdac1	Scn9a
Vdac2	Scoc
Vegf	Scpep1
Vegfa	Scrn2
Vegfc	Scrn3
Vezf1	Scrt1
Vgll4	Sct
Vhlh	Scube1
Vil1	Scube3
Vmn1r-ps2	Scyl1
Vmn1r6	Sdad1
Vmn2r-ps118	Sdc1
Vmn2r-ps131	Sdc2
Vmn2r121	Sdc3
Vmn2r90	Sdcbp
Vpreb1	Sdccag3
Vps11	Sdccag8
Vps13a	Sdf2l1
Vps13c	Sdf4
Vps18	Sdhaf1
Vps25	Sdk2
Vps29	Sdpr
Vps33a	Sds
Vps35	Sec1
Vps37b	Sec13
Vps72	Sec14l1

Vps8	Sec14l4	
Vrk3	Sec16b	
Vsig2	Sec22b	
Vsig8	Sec23a	
Vstm2b	Sec24d	
Vwa1	Sec31a	
Vwa3b	Sec61a1	
Vwa5a	Sec61a2	
WDR5	Sec61b	
WDR54	Sec62	
WFDC5	Sec63	
WIBG	Secisbp2l	
WIPF1	Sectm1a	
Wapal	Seh1l	
Wbp1	Sel1l	
Wbscr16	Sel1l2	
Wbscr17	Sel1l3	
Wdfy2	Sele	
Wdr1	Selenbp1	
Wdr27	Sell	
Wdr33	Selm	
Wdr34	Selp	
Wdr35	Selplg	
Wdr36	Sema3a	
Wdr37	Sema3b	
Wdr38	Sema3e	
Wdr40a	Sema3f	
Wdr42a	Sema3g	
Wdr46	Sema4a	
Wdr47	Sema4d	
Wdr5	Sema5a	
Wdr53	Sema5b	
Wdr55	Sema6a	
Wdr59	Sema6b	
Wdr60	Sema6c	
Wdr61	Sema6d	
Wdr63	Sema7a	
Wdr67	Senp2	
Wdr68	Senp6	
Wdr7		Sep-02
Wdr73		Sep-04
Wdr75		Sep-06
Wdr76		Sep-07
Wdr79		Sep-08
Wdr85		Sep-11
Wdtc1		Sep-15
Wdvcf	Sephs2	
Wee2	Sepn1	
Wfdc2		Sep-03
Whdc1	Sepw1	
Whrn	Serf1	
Whsc1	Serhl	
Whsc1l1	Serinc1	
Wibg	Serinc3	
Wipf2	Serinc4	
Wls	Serp1	
Wnk1	Serpina10	
Wnk4	Serpina12	
Wnt10a	Serpina1b	
Wnt11	Serpina1b///Serpina1c///Serpina1e	
Wnt2b	Serpina3a	
Wnt3	Serpina3c	
Wnt3a	Serpina3k	
Wnt5a	Serpina3m	
Wnt5b	Serpina5	
Wnt6	Serpina6	
Wnt7a	Serpinb1a	
Wnt8b	Serpinb2	
Wnt9a	Serpinb5	
Wnt9b	Serpinb8	
Wsb2	Serpinb9	
Wscd2	Serpinb9b	
Wt1	Serpinb9c	
XAB2	Serpinc1	
XRCC5	Serpind1	
XRCC6	Serpine1	
Xaf1	Serpine2	
Xiap	Serpinf1	
Xkr6	Serpinf2	
Xkr7	Serping1	
Xpc	Serpinh1	
Xpnpep3	Serpini1	
Xpo4	Serpini2	
Xpo5	Sertad2	
Xrcc6	Sertad3	
Xrn2	Sesn3	
Xtrp3s1	Set	
YWHAH	Setd1a	
YWHAQ	Setd4	
Yap1	Setd7	
Ybx1	Setdb1	
Ybx2	Sez6	
Yes1	Sez6l	
Yif1a	Sf1	
Yme1l1	Sf3a1	
Yod1	Sf3a3	
Ypel3	Sf3b2	
Ypel4	Sf3b5	
Ypel5	Sfmbt1	
Ythdf1	Sfmbt2	
Ythdf2	Sfpq	
Ywhah	Sfrp1	
Ywhaz	Sfrp2	
ZDHHC4	Sfrp5	
ZFP36L1	Sfswap	
ZFP36L2	Sftpa1	
ZHX1	Sfxn1	
ZMAT3	Sfxn3	
ZNF385A	Sfxn4	
ZNF516	Sgca	
ZNF597	Sgcb	
ZSCAN2	Sgce	
ZSCAN5A	Sgcg	
Zadh1	Sgip1	
Zadh2	Sgk1	
Zap70	Sgk3	
Zbtb1	Sgol1	
Zbtb16	Sgpl1	
Zbtb17	Sgpp1	
Zbtb22	Sgsh	
Zbtb25	Sgta	
Zbtb26	Sgtb	
Zbtb4	Sh2b2	
Zbtb5	Sh2b3	
Zbtb6	Sh2d1b1///Sh2d1b2	
Zbtb7b	Sh2d2a	
Zbtb9	Sh2d3c	
Zc3h10	Sh3bgr	
Zc3h11a	Sh3bgrl	
Zc3h12a	Sh3bgrl2	
Zc3h7b	Sh3bp5	
Zc3hav1	Sh3d19	
Zc3hc1	Sh3gl2	
Zcchc10	Sh3gl3	
Zcchc11	Sh3kbp1	
Zcchc2	Sh3pxd2a	
Zcchc3	Sh3tc1	
Zcchc6	Sh3yl1	
Zcchc7	Shank2	
Zcchc8	Shank3	
Zcwpw1	Shbg	
Zdhhc21	Shc1	
Zdhhc5	Shc2	
Zfhx2	Shc3	
Zfhx3	Shd	
Zfhx4	She	
Zfp113	Shh	
Zfp13	Shisa9	
Zfp142	Shmt1	

Zfp143	Shmt2
Zfp148	Shoc2
Zfp160	Shox2
Zfp184	Shroom1
Zfp191	Shroom3
Zfp202	Si
Zfp206	Siae
Zfp219	Siah2
Zfp238	Sidt1
Zfp239	Sigirr
Zfp259	Siglec1
Zfp260	Siglec5
Zfp277	Sike1
Zfp296	Sim2
Zfp3	Simc1
Zfp318	Sipa1
Zfp322a	Sirpa
Zfp329	Sirt2
Zfp335	Sirt4
Zfp341	Sit1
Zfp346	Siva1
Zfp36	Six1
Zfp364	Six2
Zfp36l1	Six3
Zfp385	Six4
Zfp39	Six6
Zfp395	Ska1
Zfp407	Skap2
Zfp408	Skil
Zfp410	Skiv2l
Zfp422-rs1	Skp2
Zfp426	Sla
Zfp444	Slain1
Zfp46	Slamf1
Zfp462	Slamf6
Zfp469	Slamf7
Zfp472	Slbp
Zfp473	Slc10a6
Zfp496	Slc11a2
Zfp503	Slc12a1
Zfp51	Slc12a2
Zfp516	Slc12a3
Zfp521	Slc12a5
Zfp526	Slc12a7
Zfp533	Slc12a8
Zfp553	Slc13a1
Zfp566	Slc13a3
Zfp592	Slc14a1
Zfp598	Slc14a2
Zfp60	Slc15a2
Zfp607	Slc16a1
Zfp608	Slc16a12
Zfp609	Slc16a2
Zfp617	Slc16a3
Zfp62	Slc16a7
Zfp646	Slc16a8
Zfp650	Slc17a1
Zfp652	Slc17a2
Zfp653	Slc17a3
Zfp664	Slc17a9
Zfp668	Slc18a1
Zfp672	Slc19a1
Zfp687	Slc19a2
Zfp689	Slc1a1
Zfp692	Slc1a2
Zfp710	Slc1a3
Zfp748	Slc1a4
Zfp758	Slc1a5
Zfp768	Slc20a1
Zfp771	Slc20a2
Zfp773	Slc22a12
Zfp787	Slc22a13
Zfp800	Slc22a17
Zfp817	Slc22a18
Zfp82	Slc22a19
Zfp827	Slc22a21
Zfp87	Slc22a26
Zfp91	Slc22a27
Zfp91-cntf	Slc22a29
Zfp93	Slc22a29///Slc22a30
Zfp943	Slc22a30
Zfp97	Slc22a4
Zfpl1	Slc22a5
Zfpm1	Slc22a6
Zfr	Slc22a8
Zfyve16	Slc23a1
Zfyve20	Slc23a2
Zfyve26	Slc24a1
Zfyve9	Slc24a3
Zgpat	Slc25a10
Zhx2	Slc25a14
Zhx3	Slc25a15
Zic1	Slc25a19
Zic2	Slc25a23
Zic4	Slc25a25
Zic5	Slc25a26
Zkscan1	Slc25a30
Zkscan17	Slc25a31
Zkscan6	Slc25a33
Zmat5	Slc25a35
Zmiz1	Slc25a36
Zmynd10	Slc25a39
Zmynd11	Slc25a4
Znf202	Slc25a45
Znf710	Slc25a51
Znhit1	Slc25a53
Znhit4	Slc26a2
Znrf1	Slc26a3
Znrf3	Slc26a4
Zp3	Slc26a6
Zranb1	Slc26a7
Zscan10	Slc26a8
Zscan12	Slc27a2
Zswim6	Slc27a3
Zufsp	Slc28a3
Zwilch	Slc29a2
Zwint	Slc29a3
Zyg11a	Slc29a4
Zyg11b	Slc2a10
abParts	Slc2a2
abca5	Slc2a3
apo	Slc2a4
asp4	Slc2a5
bMRP64	Slc2a8
cbp146	Slc2a9
cbp82	Slc30a1
evi-1	Slc30a2
glp	Slc30a3
hCG_1998957///HLA-DRB5	Slc30a4
hnRNP A2/B1	Slc30a9
hnRNP X	Slc32a1
if5	Slc34a1
ire1 alpha	Slc34a2
isl1-beta	Slc35a1
lama3	Slc35a2
limd1	Slc35a3
mAF10	Slc35a5
mFLJ00025	Slc35b1
mFLJ00088	Slc35b2
mFLJ00267	Slc35b4
mFLJ00277	Slc35c1
mFLJ00298	Slc35c2
mFLJ00369	Slc35e4
mKIAA0031	Slc35f4
mKIAA0044	Slc35g3
mKIAA0056	Slc37a1
mKIAA0097	Slc37a3
mKIAA0157	Slc37a4
mKIAA0174	Slc38a10

mKIAA0195	Slc38a3
mKIAA0230	Slc39a14
mKIAA0236	Slc39a7
mKIAA0306	Slc39a8
mKIAA0338	Slc39a9
mKIAA0358	Slc41a2
mKIAA0375	Slc41a3
mKIAA0404	Slc43a3
mKIAA0429	Slc44a1
mKIAA0460	Slc44a2
mKIAA0515	Slc44a3
mKIAA0544	Slc44a4
mKIAA0545	Slc46a1
mKIAA0578	Slc46a2
mKIAA0585	Slc48a1
mKIAA0612	Slc4a1
mKIAA0641	Slc4a10
mKIAA0650	Slc4a3
mKIAA0651	Slc4a4
mKIAA0737	Slc4a5
mKIAA0753	Slc4a7
mKIAA0769	Slc4a8
mKIAA0824	Slc50a1
mKIAA0881	Slc51a
mKIAA0885	Slc52a2
mKIAA0892	Slc5a1
mKIAA0893	Slc5a2
mKIAA0904	Slc5a3
mKIAA0909	Slc5a4a
mKIAA0919	Slc5a4b
mKIAA0933	Slc5a5
mKIAA1026	Slc5a7
mKIAA1027	Slc5a8
mKIAA1039	Slc6a1
mKIAA1102	Slc6a12
mKIAA1108	Slc6a14
mKIAA1125	Slc6a15
mKIAA1178	Slc6a17
mKIAA1180	Slc6a18
mKIAA1188	Slc6a2
mKIAA1238	Slc6a3
mKIAA1281	Slc6a5
mKIAA1294	Slc6a6
mKIAA1376	Slc6a9
mKIAA1380	Slc7a10
mKIAA1382	Slc7a11
mKIAA1386	Slc7a12
mKIAA1417	Slc7a13
mKIAA1441	Slc7a2
mKIAA1506	Slc7a3
mKIAA1609	Slc7a4
mKIAA1612	Slc7a5
mKIAA1707	Slc7a7
mKIAA1721	Slc8a1
mKIAA1724	Slc8a2
mKIAA1726	Slc8a3
mKIAA1737	Slc9a3r2
mKIAA1741	Slc9a8
mKIAA1745	Slc9b1
mKIAA1757	Slc9b2
mKIAA1766	Slco1a1
mKIAA1823	Slco1a4
mKIAA1835	Slco1a5
mKIAA1921	Slco1a6
mKIAA1971	Slco1b2
mKIAA1973	Slco1c1
mKIAA1981	Slco2a1
mKIAA1998	Slco4a1
mKIAA2006	Slco4c1
mKIAA2037	Slco5a1
mKIAA3014	Slfn1
mKIAA3028	Slfn4
mKIAA4020	Slit1
mKIAA4026	Slit2
mKIAA4029	Slit3
mKIAA4075	Slitrk1
mKIAA4106	Slitrk5
mKIAA4110	Slk
mKIAA4116	Slmap
mKIAA4123	Slmo1
mKIAA4163	Sln
mKIAA4193	Slpi
mKIAA4237	Slu7
mSSH-2L	Slurp1
mSSH-3L	Slx1b
mTOP3beta	Smad3
mVAMP-1	Smad6
mazr	Smad9
mboct	Smap1
meis2a	Smarca1
mimp3	Smarca4
mmu-let-7e	Smarca5
mmu-mir-101a	Smarcb1
mmu-mir-10a	Smarcc1
mmu-mir-1186b	Smarcd1
mmu-mir-1192	Smarcd3
mmu-mir-1199	Smc1a
mmu-mir-124-1	Smc1b
mmu-mir-125a	Smc3
mmu-mir-125b-2	Smc5
mmu-mir-130a	Smg5
mmu-mir-132	Smim11
mmu-mir-135a-1	Smo
mmu-mir-135b	Smoc1
mmu-mir-146a	Smoc2
mmu-mir-146b	Smok3a///Smok3b///Smok3c
mmu-mir-15a	Smox
mmu-mir-16-1	Smpd3
mmu-mir-182	Smpd4
mmu-mir-183	Smpd5
mmu-mir-191	Smpx
mmu-mir-193	Smr2
mmu-mir-1931	Smtnl1
mmu-mir-1934	Smu1
mmu-mir-1946a	Smyd1
mmu-mir-1949	Smyd5
mmu-mir-195	Snai1
mmu-mir-1955	Snai2
mmu-mir-1960	Snap23
mmu-mir-1966	Snap29
mmu-mir-1967	Snap47
mmu-mir-196a-1	Snap91
mmu-mir-196a-2	Snapc2
mmu-mir-196b	Snapc3
mmu-mir-1983	Snapc5
mmu-mir-212	Snapin
mmu-mir-219-1	Snca
mmu-mir-26b	Sncaip
mmu-mir-290	Sncb
mmu-mir-291a	Sncg
mmu-mir-291b	Snd1
mmu-mir-292	Sned1
mmu-mir-293	Snhg1
mmu-mir-294	Snhg11
mmu-mir-295	Snhg5
mmu-mir-301a	Snhg7
mmu-mir-3058	Snn
mmu-mir-3064	Snrk
mmu-mir-3078	Snrnp27
mmu-mir-3089	Snrnp40
mmu-mir-3093	Snrnp70
mmu-mir-3098	Snrpb
mmu-mir-3100	Snrpb2
mmu-mir-324	Snrpd3
mmu-mir-328	Snrpn /// Snurf
mmu-mir-3470a	Snrpn///Snurf
mmu-mir-365-2	Snta1
mmu-mir-425	Sntb1
mmu-mir-483	Sntb2

mmu-mir-497
mmu-mir-615
mmu-mir-670
mmu-mir-671
mmu-mir-702
mmu-mir-706
mmu-mir-709
mmu-mir-761
mmu-mir-762
mmu-mir-7a-2
mmu-mir-804
mmu-mir-877
mmu-mir-9-1
mmu-mir-9-3
mmu-mir-92b
mmu-mir-96
mmu-mir-99b
mpp-GalNAc-T17(O6)
mt3-mmp
muspaox
myo 10
n-R5s165
n-R5s179
n-R5s24
n-R5s46
n-R5s86
netrin-G1
ni-2
nssr 1
p130Cas
p53
pa
radical-fringe
rjs
s11-6
sgigsf
sid2895
snoCD11
snoU54
stap-1
ten-m2
traf4
tubg1
ubcM4
wdc146

Sntg1
Snx15
Snx19
Snx2
Snx20
Snx21
Snx30
Snx4
Snx5
Snx6
Snx9
Soat1
Soat2
Sobp
Socs1
Socs2
Socs3
Socs4
Socs5
Socs7
Sod1
Sod2
Sod3
Soga3
Son
Sorbs1
Sorbs3
Sorcs1
Sorcs2
Sorcs3
Sorl1
Sort1
Sos1
Sos2
Sostdc1
Sox1
Sox10
Sox11
Sox13
Sox14
Sox17
Sox18
Sox2
Sox21
Sox3
Sox4
Sox5
Sp1
Sp100
Sp110
Sp4
Sp6
Sp7
Spa17
Spag1
Spag4
Spag5
Spag6
Spag7
Spam1
Sparc
Sparcl1
Spata13
Spata19
Spata5
Spata6
Spats2l
Spcs2
Spcs3
Spdef
Spdl1
Speer4a
Speer4f
Spef2
Speg
Spesp1
Sphk1
Spic
Spin1
Spin4
Spink3
Spint1
Spint2
Spire2
Spn
Spn-ps
Spo11
Spock1
Spock2
Spock3
Spon1
Spon2
Spop
Spp1
Spp2
Sppl2a
Spr
Spred1
Spred2
Sprr1a
Sprr2d
Sprr2f
Sprr2h
Sprr2i
Sprr3
Spry1
Spry2
Spry4
Spryd3
Spsb1
Spsb4
Spt1
Spta1
Sptbn1
Sptbn2
Sptbn4
Sptlc1
Sptlc2
Sptlc3
Sptssb
Spz1
Sqle
Sqrdl
Sqstm1
Src
Srcin1
Srd5a2
Srd5a3
Srebf1
Srebf2
Srek1
Srgap1
Srgap2
Srgap3
Srgn
Sri
Srm
Srp54a///Srp54b
Srp68
Srpk3
Srpr
Srprb
Srpx
Srpx2
Srr
Srrm1
Srsf12
Srsf2
Srsf3

Srsf7
Srsf9
Srst
Srxn1
Ss18
Ssb
Ssbp1
Ssbp2
Ssbp3
Ssbp4
Ssc5d
Sspn
Ssr1
Ssr4
Ssrp1
Sst
Sstr2
Sstr3
Sstr4
Ssx2ip
St14
St3gal1
St3gal2
St3gal3
St3gal4
St3gal5
St3gal6
St6gal1
St6gal2
St6galnac1
St6galnac2
St6galnac3
St6galnac4
St6galnac5
St6galnac6
St8sia1
St8sia2
St8sia4
St8sia5
Stab2
Stac
Stac2
Stag1
Stag3
Stam2
Stambpl1
Stap1
Stap2
Stard10
Stard4
Stard6
Stard8
Stat3
Stat5a
Stat5b
Stau1
Stbd1
Stc1
Stc2
Steap1
Steap2
Steap3
Stfa1 /// Stfa3
Stfa2l1
Stil
Stip1
Stk11
Stk11ip
Stk16
Stk25
Stk3
Stk31
Stk32c
Stk35
Stk39
Stk4
Stk40
Stmn2
Stmn3
Stom
Ston1
Stra13
Stra6
Stra8
Strada
Strap
Strbp
Strc
Strm
Strn
Strn3
Stt3a
Stx17
Stx18
Stx19
Stx1a
Stx2
Stx3
Stx4a
Stx6
Stx8
Stxbp1
Stxbp2
Stxbp3a
Stxbp4
Stxbp6
Styx
Suco
Sufu
Sugct
Sugp2
Sulf1
Sulf2
Sult1a1
Sult1c1
Sult1c2
Sult1d1
Sult1e1
Sult2a2
Sumf1
Sumo3
Sun2
Supt20
Surf2
Surf4
Susd4
Suv420h1
Suz12
Sva
Sval1
Svep1
Svop
Svopl
Svs2
Svs3a
Svs3a///Svs3b
Svs5
Svs6
Swap70
Swi5
Swsap1
Syap1
Sycp1
Sycp3
Syde2
Syk
Syn1
Syn2
Sync

Syncrip
Syne1
Syne4
Syngr1
Syngr3
Synj2
Synj2bp
Synpo
Syp
Sypl
Sypl2
Sys1
Syt1
Syt10
Syt11
Syt12
Syt13
Syt17
Syt2
Syt3
Syt4
Syt5
Syt6
Syt7
Syt9
Sytl1
Sytl2
Sytl4
T
T2
TCRB///Tcrb-J///Trbv14
Taar1
Tac1
Tac2
Tac4
Tacc3
Tacr3
Tacstd1
Tacstd2
Tada1
Tada2b
Taf10
Taf11
Taf1d
Taf4b
Taf6l
Taf7
Tagln
Tagln3
Tal1
Tal2
Tanc1
Taok3
Tap1
Tapbp
Tapbpl
Tarbp2
Tardbp
Tas1r1
Tas1r2
Tas1r3
Tas2r105
Tasp1
Tax1bp1
Tax1bp3
Tbata
Tbc1d1
Tbc1d13
Tbc1d14
Tbc1d15
Tbc1d22a
Tbc1d24
Tbc1d4
Tbc1d8b
Tbca
Tbcel
Tbkbp1
Tbl1x
Tbl1xr1
Tbl2
Tbl3
Tbp
Tbrg4
Tbx15
Tbx18
Tbx2
Tbx20
Tbx21
Tbx3
Tbx4
Tbx5
Tbxa2r
Tbxas1
Tc2n
Tcea2
Tceal1
Tceal3
Tceal3///Tceal5///Tceal6
Tceal8
Tceb1
Tceb2
Tceb3
Tcerg1
Tcf15
Tcf20
Tcf21
Tcf23
Tcf25
Tcf3
Tcf4
Tcf7
Tcf7l1
Tcf7l2
Tcfap2c
Tcfap2e
Tcfl5
Tcl1
Tcl1b4
Tcof1
Tcp10a///Tcp10b///Tcp10c
Tcp11
Tcrb-J
Tcrg-V3
Tcstv1
Tcstv3
Tcta
Tcte2
Tctex1d1
Tctn2
Tctn3
Tdgf1
Tdh
Tdo2
Tdp1
Tdp2
Tdpoz1
Tdrd7
Tead1
Tead2
Tead4
Tecpr1
Tecta
Tectb
Tef
Tek
Tekt2
Telo2
Tenc1
Tenm1

Tenm3
Terf1
Terf2
Tesc
Tescl
Tesk1
Tesk2
Tet1
Tex12
Tex13
Tex14
Tex15
Tex16
Tex19.1
Tex40
Tex9
Tfap2a
Tfap2c
Tfap2d
Tfap4
Tfb1m
Tfcp2
Tfcp2l1
Tfdp1
Tff2
Tfpi
Tfpi2
Tfpt
Tfr2
Tfrc
Tg
Tgds
Tgfb1
Tgfb1i1
Tgfb2
Tgfb3
Tgfbi
Tgfbr1
Tgfbr2
Tgfbr3
Tgif2
Tgif2lx1///Tgif2lx2
Tgm2
Tgm3
Tgs1
Tha1
Thap11
Thap2
Thap7
Thbd
Thbs1
Thbs2
Thbs3
Theg
Thnsl2
Thoc1
Thoc2
Thoc7
Thop1
Thpo
Thra
Thrb
Thsd1
Thsd7a
Thtpa
Thumpd3
Thy1
Thyn1
Tia1
Tial1
Tiam1
Tiam2
Tie1
Tifa
Timd2
Timeless
Timm10b
Timm17a
Timm9
Timp1
Timp2
Timp3
Timp4
Tinag
Tinagl1
Tirap
Tk2
Tktl1
Tlcd1
Tlcd2
Tle1
Tle2
Tle3
Tle6
Tlk2
Tlm
Tln1
Tlr1
Tlr2
Tlr3
Tlr4
Tlr7
Tlr8
Tlx1
Tlx3
Tm2d1
Tm4sf1
Tm4sf5
Tm6sf1
Tm6sf2
Tm7sf3
Tm9sf2
Tm9sf3
Tma16
Tmc1
Tmc2
Tmcc2
Tmcc3
Tmco3
Tmco5
Tmed1
Tmed11
Tmed3
Tmed4
Tmed5
Tmed6
Tmed7
Tmed8
Tmed9
Tmeff1
Tmeff2
Tmem100
Tmem106a
Tmem106b
Tmem106c
Tmem109
Tmem116
Tmem119
Tmem120b
Tmem129
Tmem132a
Tmem132e
Tmem141
Tmem14c
Tmem150a
Tmem151b
Tmem158
Tmem160
Tmem161a

Tmem163
Tmem166
Tmem167
Tmem16j
Tmem173
Tmem175
Tmem176a
Tmem176b
Tmem179b
Tmem184c
Tmem189
Tmem191c
Tmem192
Tmem2
Tmem203
Tmem206
Tmem214
Tmem216
Tmem218
Tmem223
Tmem229b
Tmem242
Tmem246
Tmem255a
Tmem258
Tmem259
Tmem263
Tmem27
Tmem30a
Tmem30b
Tmem33
Tmem35
Tmem37
Tmem38a
Tmem38b
Tmem39a
Tmem40
Tmem41a
Tmem45a
Tmem47
Tmem5
Tmem53
Tmem54
Tmem55a
Tmem56
Tmem59
Tmem68
Tmem71
Tmem80
Tmem82
Tmem97
Tmem98
Tmigd1
Tmlhe
Tmod1
Tmod2
Tmod3
Tmpo
Tmppe
Tmprss11d
Tmprss13
Tmprss2
Tmprss5
Tmsb10
Tmtc1
Tmtc2
Tmx1
Tnf
Tnfaip2
Tnfaip6
Tnfaip8
Tnfrsf10b
Tnfrsf11a
Tnfrsf11b
Tnfrsf13b
Tnfrsf13c
Tnfrsf14
Tnfrsf17
Tnfrsf18
Tnfrsf19
Tnfrsf1b
Tnfrsf21
Tnfrsf22
Tnfsf10
Tnfsf11
Tnfsf12///Tnfsf12Tnfsf13
Tnfsf12///Tnfsf12Tnfsf13///Tnfsf13
Tnfsf13b
Tnfsf4
Tnfsf8
Tnfsf9
Tnks2
Tnmd
Tnn
Tnnc1
Tnnc2
Tnni2
Tnni3
Tnnt1
Tnnt2
Tnnt3
Tnp1
Tnpo2
Tnrc6a
Tnrc6b
Tns1
Tns3
Tns4
Tnxb
Toe1
Tom1
Tom1l2
Tomm40
Tomm70a
Top2b
Top3a
Top3b
Topors
Toporsl
Toporsos
Tor1b
Tor2a
Tox
Tox3
Tpbpa
Tpcn1
Tpd52
Tpd52l1
Tpd52l2
Tph1
Tpi1
Tpm1
Tpm2
Tpm3
Tpmt
Tpo
Tpp2
Tppp3
Tpr
Tprkb
Tprn
Tpsab1
Tpsb2
Tpsg1
Tpst1
Tpst2
Tpte
Trabd
Traf1

Traf3
Traf4
Traf6
Trak1
Tram1
Tram1l1
Trap1a
Trappc13
Trappc3
Trappc4
Trav12n-1
Trav9d-3
Trbv13-2
Trdmt1
Trdn
Treh
Trem1
Trem3
Trex2
Trh
Trhr
Trhr2
Trib2
Trib3
Tril
Trim12a
Trim15
Trim16
Trim2
Trim24
Trim25
Trim26
Trim27
Trim29
Trim3
Trim30a
Trim30a///Trim30d
Trim30d
Trim31
Trim34a
Trim34a///Trim34b
Trim35
Trim43a
Trim44
Trim46
Trim47
Trim6
Trim60
Trim60,Trim61
Trim62
Trim69
Trim7
Trim8
Trim9
Trio
Trip4
Trmt1
Trmt10a
Trnt1
Tro
Trp53
Trp53bp1
Trp53inp2
Trp63
Trpc1
Trpc2///Xntrpc
Trpc4
Trpc6
Trpc7
Trpd52l3
Trpm1
Trpm3
Trpm5
Trpm7
Trps1
Trpv2
Trub2
Tsc1
Tsc22d1
Tsc22d3
Tsen15
Tsga13
Tshb
Tshr
Tshz2
Tsix
Tsks
Tslp
Tsn
Tsnax
Tsnaxip1
Tspan1
Tspan10
Tspan12
Tspan13
Tspan15
Tspan17
Tspan2
Tspan3
Tspan31
Tspan32
Tspan33
Tspan4
Tspan5
Tspan6
Tspan7
Tspo2
Tspyl1
Tspyl4
Tsr2
Tsr3
Tssc4
Tssk1
Tssk2
Tssk3
Tssk6
Tst
Tsx
Ttbk2
Ttc1
Ttc14
Ttc19
Ttc27
Ttc28
Ttc3
Ttc30b
Ttc36
Ttc39b
Ttc39c
Ttc39d
Ttc7b
Ttf1
Tti2
Ttk
Ttl
Ttll1
Ttll3
Ttn
Ttr
Ttyh1
Ttyh2
Tub
Tuba-rs1
Tuba3a///Tuba3b
Tuba4a
Tuba8
Tubb2a-ps2
Tubb3
Tubb4a

Tubb5
Tubb6
Tubg2
Tufm
Tulp1
Tulp2
Tulp3
Tusc2
Tusc3
Tusc5
Tut1
Tvp23a
Twf2
Twist1
Twist2
Twsg1
Txlnb
Txn2
Txndc11
Txndc12
Txndc5
Txndc8
Txnip
Txnl1
Txnrd1
Txnrd2
Tyms
Tyr
Tyrobp
Tyrp1
Tyw1
U2af1l4
U2surp
U90926
Uba1
Uba1y
Uba5
Uba7
Ubap2l
Ubash3b
Ubc
Ube2c
Ube2d2b
Ube2d3
Ube2e2
Ube2g2
Ube2h
Ube2j1
Ube2k
Ube2l6
Ube2m
Ube2o
Ube2s
Ube2v1
Ube3a
Ube4a
Ube4b
Ubfd1
Ubl4b
Ublcp1
Ubn1
Ubqln1
Ubqln2
Ubr1
Ubr7
Ubtd1
Ubxn10
Ubxn11
Ubxn2a
Ubxn4
Ubxn8
Uchl1
Uchl4
Ucp2
Ucp3
Uevld
Ufd1l
Ufm1
Ugcg
Uggt2
Ugt2a1///Ugt2a2
Ugt2b34
Ugt2b38
Ugt3a1
Ugt8a
Uhmk1
Uhrf1bp1
Ulk1
Ulk2
Umod
Umps
Unc13a
Unc5b
Unc5c
Unc93b1
Ung
Unk
Upk1a
Upk2
Upk3a
Upk3b
Upp1
Upp2
Uqcr10
Urm1
Uroc1
Usf1
Ush1c
Ush2a
Ushbp1
Usmg2
Usp1
Usp11
Usp12
Usp13
Usp16
Usp17la
Usp17lc///Usp17ld
Usp22
Usp36
Usp39
Usp42
Usp43
Usp45
Usp48
Usp51
Usp7
Usp9x
Usp9y
Utf1
Utp6
Utrn
Uts2
Utx
Uty
Uxs1
Vamp2
Vamp3
Vamp4
Vamp5
Vamp7
Vangl2
Vapa
Vapb
Vars
Vash1
Vash2
Vasn
Vav3

Vax2
Vcam1
Vcan
Vcl
Vcp
Vdac3
Vegfa
Vegfb
Vegfc
Vgll1
Vgll3
Vgll4
Vhl
Vil1
Vim
Vimp
Vip
Vipr1
Vipr2
Vit
Vkorc1
Vldlr
Vma21
Vmn1r-ps8///Vmn1r-ps8
Vmn1r10///Vmn1r9
Vmn1r13
Vmn1r14
Vmn1r15
Vmn1r171
Vmn1r172
Vmn1r187///Vmn1r63
Vmn1r25
Vmn1r29
Vmn1r40
Vmn1r41
Vmn1r44
Vmn1r45
Vmn1r46
Vmn1r47
Vmn1r47///Vmn1r48
Vmn1r49
Vmn1r50
Vmn1r51
Vmn1r53
Vmn1r56
Vmn1r58
Vmn2r-ps54
Vmn2r1
Vmn2r10
Vmn2r103///Vmn2r96///Vmn2r97///Vmn2r98
Vmn2r26
Vmn2r88
Vnn1
Vopp1
Vprbp
Vpreb1
Vps11
Vps13c
Vps18
Vps25
Vps28
Vps35
Vps39
Vps53
Vps72
Vps9d1
Vrk2
Vsig2
Vsnl1
Vstm2a
Vstm2b
Vsx1
Vtn
Vwa1
Vwa2
Vwa5a
Vwc2
Vwf
WIF1
Wap
Wars
Wasf1
Wasf3
Wasl
Wbp1l
Wbscr22
Wbscr25
Wbscr27
Wdfy1
Wdfy3
Wdpcp
Wdr12
Wdr13
Wdr20
Wdr26
Wdr33
Wdr45
Wdr54
Wdr6
Wdr75
Wdr77
Wdr81
Wdr86
Wdsub1
Wee1
Wfdc1
Wfdc12
Wfdc15b
Wfdc18
Wfdc2
Wfdc21
Wfdc5
Wfikkn1
Wfs1
Wif1
Wipf1
Wipi1
Wipi2
Wisp1
Wisp2
Wnk1
Wnk2
Wnk4
Wnt1
Wnt10a
Wnt10b
Wnt16
Wnt2b
Wnt5a
Wnt5b
Wnt6
Wnt7a
Wnt7b
Wnt8b
Wnt9a
Wnt9b
Wrap53
Wrn
Wrnip1
Wsb1
Wt1
Wtap
Wwc1
Wwc2
Wwox
Wwp2
Wwtr1
Xbp1
Xcr1

Xdh
Xiap
Xirp1
Xist
Xk
Xkr6
Xlr4a///Xlr4b///Xlr4c
Xpa
Xpnpep1
Xpnpep2
Xpr1
Xrcc2
Xrcc4
Xrn1
Xylb
Xylt2
Yae1d1
Yaf2
Yars
Ybx1
Ybx2
Yes1
Yipf4
Ykt6
Yme1l1
Ypel1
Ypel2
Ythdf1
Ywhab
Ywhag
Ywhaq
Ywhaz
Zak
Zap70
Zbed3
Zbed6///Zc3h11a
Zbp1
Zbtb11
Zbtb12
Zbtb14
Zbtb16
Zbtb20
Zbtb3
Zbtb33
Zbtb37
Zbtb45
Zbtb46
Zbtb7a
Zbtb7c
Zbtb8a
Zc2hc1c
Zc3h12a
Zc3h12b
Zc3h3
Zc3h7a
Zc3hc1
Zcchc14
Zcchc18
Zcchc3
Zcchc5
Zdhhc1
Zdhhc12
Zdhhc14
Zdhhc2
Zdhhc3
Zdhhc6
Zeb1
Zeb2
Zfand2a
Zfand3
Zfand5
Zfand6
Zfhx3
Zfhx4
Zfml
Zfp105
Zfp106
Zfp108
Zfp110
Zfp111
Zfp125
Zfp14
Zfp142
Zfp143
Zfp148
Zfp160
Zfp179
Zfp189
Zfp191
Zfp202
Zfp207
Zfp213
Zfp235
Zfp239
Zfp260
Zfp275
Zfp276
Zfp277
Zfp28
Zfp280d
Zfp281
Zfp286
Zfp287
Zfp30
Zfp318
Zfp319
Zfp334
Zfp346
Zfp352
Zfp353-ps
Zfp354a
Zfp354b
Zfp354c
Zfp36
Zfp362
Zfp369
Zfp36l1
Zfp36l2
Zfp37
Zfp386
Zfp395
Zfp398
Zfp40
Zfp418
Zfp42
Zfp422
Zfp423
Zfp428
Zfp444
Zfp451
Zfp462
Zfp467
Zfp472
Zfp474
Zfp493
Zfp503
Zfp518b
Zfp52
Zfp521
Zfp53
Zfp553
Zfp57
Zfp574
Zfp575
Zfp59
Zfp60
Zfp605
Zfp61
Zfp612

Zfp618
Zfp64
Zfp641
Zfp644
Zfp651
Zfp68
Zfp688
Zfp689
Zfp697
Zfp704
Zfp750
Zfp768
Zfp771
Zfp787
Zfp790
Zfp804b
Zfp81
Zfp810
Zfp82
Zfp825
Zfp865
Zfp868
Zfp87
Zfp90
Zfp92
Zfp93
Zfp931
Zfp937
Zfp94
Zfp940
Zfp943
Zfp944
Zfp945
Zfpm1
Zfpm2
Zfr
Zfx
Zfy1///Zfy2
Zfyve16
Zfyve19
Zfyve21
Zfyve27
Zg16
Zhx1
Zic1
Zic2
Zic3
Zic4
Zic5
Zik1
Zim1
Zim3
Zkscan14
Zkscan16
Zkscan2
Zkscan3
Zkscan5
Zmat5
Zmiz1
Zmym1
Zmym5
Zmynd10
Zmynd11
Zmynd19
Znhit1
Znhit3
Znrd1
Znrf1
Znrf4
Zp1
Zp2
Zp3r
Zpbp
Zpr1
Zranb2
Zrsr1
Zrsr2
Zscan12
Zscan18
Zscan2
Zscan21
Zscan26
Zscan5b
Zswim1
Zswim7
Zwint
Zxdc
Zyg11a
a
abParts///Igh-VJ558///Ighm///Ighv1-53///Ighv1-62-3///Ighv1-72
abParts///Igh///Ighg1///Ighm
abParts///Ighm
abParts///Igkv14-111
abParts///Igkv4-55///Igkv4-62///Igkv4-68///Igkv4-69///Igkv4-71///Igkv4-72///Igkv4-78
abParts///Igkv4-68///Igkv4-73///Igkv4-81
abParts///Igkv6-13///Igkv6-14

Table S5: Bioinformatic analysis of the intersection between the pan-DP signature and the pan-Blimp1 target genes

This table consists of 3 tabs

Analysis Settings

Cutoff before duplicate resolution	TRUE
Reference set	Ingenuity Knowledge Base (Genes + Endogenous Chemicals)
Relationship to include	Direct and Indirect
Includes Endogenous Chemicals	Yes
Optional Analyses	My Pathways My List
Filter Summary	
Consider only relationships where	
confidence = Experimentally Observed	

Diseases and Bio Functions

Categories	Functions	Diseases or Functions Annotation	p-Value
Cellular Growth and Proliferation	proliferation	proliferation of cells	2.63E-77
Organismal Survival	organismal death	organismal death	6.43E-72
Organismal Survival	morbidity or mortality	morbidity or mortality	1.15E-70
Cancer, Organismal Injury and Abnormalities	malignant solid tumor	malignant solid tumor	1.64E-63
Cancer, Organismal Injury and Abnormalities	cancer	cancer	4.23E-60
Cancer, Organismal Injury and Abnormalities	non-melanoma solid tumor	non-melanoma solid tumor	5.60E-59
Cancer, Organismal Injury and Abnormalities	neoplasia	neoplasia of epithelial tissue	2.49E-58
Cellular Growth and Proliferation, Tissue Development	generation	generation of cells	4.51E-58
Cancer, Organismal Injury and Abnormalities	tumorigenesis	tumorigenesis of tissue	5.21E-58
Cellular Development	differentiation	differentiation of cells	5.79E-58
Cancer, Organismal Injury and Abnormalities	epithelial cancer	epithelial cancer	1.06E-56
Cancer, Gastrointestinal Disease, Organismal Injury and Abnormalities	digestive organ tumor	digestive organ tumor	2.03E-55
Cancer, Gastrointestinal Disease, Organismal Injury and Abnormalities	digestive system cancer	digestive system cancer	6.13E-55
Gene Expression	transcription	transcription of RNA	8.72E-55
Cancer, Organismal Injury and Abnormalities	abdominal neoplasm	abdominal neoplasm	1.52E-54
Gene Expression	transcription	transcription	4.76E-54
Cancer, Organismal Injury and Abnormalities	abdominal cancer	abdominal cancer	7.45E-54
Cell Morphology	morphology	morphology of cells	1.75E-52
Gene Expression	expression	expression of RNA	5.83E-52
Cancer, Organismal Injury and Abnormalities	adenocarcinoma	adenocarcinoma	4.60E-51
Gene Expression	transcription	transcription of DNA	9.68E-51
Nervous System Development and Function	morphology	morphology of nervous system	1.20E-49
Cell Death and Survival	cell death	cell death	3.33E-49
Cancer, Gastrointestinal Disease, Organismal Injury and Abnormalities	gastrointestinal carcinoma	gastrointestinal carcinoma	1.82E-47
Cancer, Gastrointestinal Disease, Organismal Injury and Abnormalities	gastrointestinal tract cancer	gastrointestinal tract cancer	3.33E-47
Cancer, Gastrointestinal Disease, Organismal Injury and Abnormalities	neoplasia	Gastrointestinal Tract Cancer and Tumors	4.27E-47
Embryonic Development, Organismal Development	development	development of body trunk	4.72E-47
Cancer, Gastrointestinal Disease, Organismal Injury and Abnormalities	large intestine carcinoma	large intestine carcinoma	1.92E-46
Cancer, Gastrointestinal Disease, Organismal Injury and Abnormalities	intestinal carcinoma	intestinal carcinoma	2.01E-46
Cancer, Gastrointestinal Disease, Organismal Injury and Abnormalities	large intestine neoplasm	large intestine neoplasm	2.19E-46
Cancer, Gastrointestinal Disease, Organismal Injury and Abnormalities	malignant neoplasm of large intestine	malignant neoplasm of large intestine	4.40E-46
Embryonic Development, Organismal Development	development	development of body axis	4.55E-46
Nervous System Development and Function, Neurological Disease	abnormal morphology	abnormal morphology of nervous system	5.00E-46

Cancer, Gastrointestinal Disease, Organismal Injury and Abnormalities	intestinal cancer	intestinal cancer	1.14E-45
Cancer, Gastrointestinal Disease, Organismal Injury and Abnormalities	intestinal tumor	intestinal tumor	1.18E-45
Cellular Development, Cellular Growth and Proliferation, Nervous System Development and	development	development of neurons	1.66E-45
Gene Expression	activation	activation of DNA endogenous promoter	3.71E-45
Cell Death and Survival	necrosis	necrosis	1.38E-44
Cell Death and Survival	apoptosis	apoptosis	2.47E-44
Cell Morphology	abnormal morphology	abnormal morphology of cells	6.31E-44
Cancer, Gastrointestinal Disease, Organismal Injury and Abnormalities	intestinal adenocarcinoma	intestinal adenocarcinoma	2.07E-43
Cancer, Gastrointestinal Disease, Organismal Injury and Abnormalities	large intestine adenocarcinoma	large intestine adenocarcinoma	3.46E-43
Embryonic Development, Organismal Development	development	development of head	4.51E-43
Cellular Movement	cell movement	cell movement	9.43E-41
Organismal Development, Organismal Injury and Abnormalities	abnormal morphology	abnormal morphology of head	3.74E-40
Organismal Development	morphology	morphology of head	6.76E-40
Organismal Development	morphology	morphology of body cavity	4.12E-39
Tissue Morphology	quantity	quantity of cells	1.31E-37
Cellular Assembly and Organization, Cellular Function and Maintenance	organization	organization of cytoplasm	5.91E-37
Cell Death and Survival	survival	cell survival	9.35E-37
Cellular Movement	migration	migration of cells	1.61E-36
Cellular Assembly and Organization, Cellular Function and Maintenance	organization	organization of cytoskeleton	1.93E-36
Cellular Development	differentiation	differentiation of nervous system	2.62E-36
Organismal Development	abnormal morphology	abnormal morphology of body cavity	8.09E-36
Cellular Assembly and Organization, Cellular Function and Maintenance	organization	microtubule dynamics	4.96E-35
Cell Death and Survival	cell viability	cell viability	1.36E-34
Cancer, Organismal Injury and Abnormalities	breast or colorectal cancer	breast or colorectal cancer	1.51E-34
Cellular Development, Cellular Growth and Proliferation	cell proliferation	cell proliferation of tumor cell lines	9.23E-34
Cardiovascular System Development and Function	development	development of vasculature	7.84E-33
Cellular Movement	invasion	invasion of cells	2.08E-32
Cancer, Organismal Injury and Abnormalities	abdominal adenocarcinoma	abdominal adenocarcinoma	2.94E-32
Cell Morphology, Cellular Assembly and Organization, Cellular Development, Cellular Functi	neuritogenesis	neuritogenesis	7.15E-32
Cell Death and Survival	cell death	cell death of tumor cell lines	7.25E-32
Cell Morphology, Cellular Assembly and Organization, Cellular Function and Maintenance	formation	formation of cellular protrusions	2.70E-31
Cancer, Organismal Injury and Abnormalities	abdominal carcinoma	abdominal carcinoma	6.04E-31
Cell Morphology, Nervous System Development and Function, Tissue Morphology	morphology	morphology of neurons	6.50E-31
Tissue Development	growth	growth of epithelial tissue	6.87E-31
Developmental Disorder, Embryonic Development, Organismal Development, Tissue Morpl	abnormal morphology	abnormal morphology of embryonic tissue	1.01E-30
Nervous System Development and Function, Tissue Morphology	morphology	morphology of nervous tissue	1.33E-30
Nervous System Development and Function	development	development of central nervous system	1.87E-30
Cell Death and Survival	apoptosis	apoptosis of tumor cell lines	6.19E-30
Cardiovascular System Development and Function, Organismal Development	angiogenesis	angiogenesis	3.33E-29
Cellular Development, Nervous System Development and Function, Tissue Development	differentiation	differentiation of neurons	6.52E-29
Developmental Disorder	dysgenesis	dysgenesis	7.88E-29
Gastrointestinal Disease, Hepatic System Disease, Organismal Injury and Abnormalities	liver lesion	liver lesion	1.08E-28
Cancer, Organismal Injury and Abnormalities	benign neoplasia	benign neoplasia	1.78E-28
Cell Morphology, Cellular Assembly and Organization, Cellular Development, Cellular Functi	axonogenesis	axonogenesis	2.62E-28
Molecular Transport	transport	transport of molecule	4.43E-28
Cellular Function and Maintenance	homeostasis	cellular homeostasis	6.68E-28

Developmental Disorder	hypoplasia	hypoplasia of organ	8.35E-28
Organismal Development, Organismal Injury and Abnormalities	abnormal morphology	abnormal morphology of abdomen	1.45E-27
Developmental Disorder	hypoplasia	Hypoplasia	3.08E-27
Developmental Disorder	growth failure	Growth Failure	3.10E-27
Cell Cycle	cell cycle progression	cell cycle progression	8.90E-27
Embryonic Development, Nervous System Development and Function, Organ Development	formation	formation of brain	9.52E-27
Organismal Development	growth	growth of organism	1.25E-26
Cellular Development, Cellular Growth and Proliferation, Nervous System Development and	proliferation	proliferation of neuronal cells	1.89E-26
Cellular Movement	cell movement	cell movement of tumor cell lines	2.72E-26
Cancer, Gastrointestinal Disease, Organismal Injury and Abnormalities	colorectal neoplasia	colorectal neoplasia	2.89E-26
Cancer, Gastrointestinal Disease, Hepatic System Disease, Organismal Injury and Abnormali	liver tumor	liver tumor	3.07E-26
Embryonic Development, Organ Development, Organismal Development, Tissue Developm	development	development of sensory organ	3.94E-26
Cell Death and Survival	cell death	neuronal cell death	4.31E-26
Nervous System Development and Function	morphology	morphology of central nervous system	4.82E-26
Gene Expression	transactivation	transactivation	5.45E-26
Organismal Development, Organismal Injury and Abnormalities	abnormal morphology	abnormal morphology of thoracic cavity	1.06E-25
Nervous System Development and Function, Neurological Disease	abnormal morphology	abnormal morphology of central nervous system	1.10E-25
Organismal Development	development	development of genitourinary system	1.30E-25
Cancer, Gastrointestinal Disease, Organismal Injury and Abnormalities	colorectal cancer	colorectal cancer	1.34E-25
Embryonic Development, Organismal Development	growth	growth of embryo	1.89E-25
Cancer, Organismal Injury and Abnormalities, Reproductive System Disease	breast or ovarian cancer	breast or ovarian cancer	1.99E-25
Cancer, Gastrointestinal Disease, Hepatic System Disease, Organismal Injury and Abnormali	hepatobiliary system cancer	hepatobiliary system cancer	2.83E-25
Organismal Development	size	size of body	4.39E-25
Neurological Disease	movement disorder	Movement Disorders	4.64E-25
Cellular Movement	invasion	invasion of tumor cell lines	5.29E-25
Cancer, Gastrointestinal Disease, Organismal Injury and Abnormalities	colorectal carcinoma	colorectal carcinoma	5.49E-25
Cancer, Gastrointestinal Disease, Hepatic System Disease, Organismal Injury and Abnormali	cancer	liver cancer	7.47E-25
Digestive System Development and Function	development	development of digestive system	7.75E-25
Nervous System Development and Function, Organ Morphology, Organismal Development	morphology	morphology of brain	8.23E-25
Cardiovascular System Development and Function, Organismal Development	vasculogenesis	vasculogenesis	8.40E-25
Cellular Movement	migration	migration of tumor cell lines	1.01E-24
Gene Expression	transactivation	transactivation of RNA	1.26E-24
Cell Morphology, Nervous System Development and Function, Neurological Disease, Tissue	abnormal morphology	abnormal morphology of neurons	1.58E-24
Cancer, Gastrointestinal Disease, Hepatic System Disease, Organismal Injury and Abnormali	liver carcinoma	liver carcinoma	1.68E-24
Nervous System Development and Function, Neurological Disease, Organ Morphology, Org	abnormal morphology	abnormal morphology of brain	2.23E-24
Cardiovascular System Development and Function	morphology	morphology of cardiovascular system	2.58E-24
Cancer, Gastrointestinal Disease, Organismal Injury and Abnormalities	colon tumor	colon tumor	6.34E-24
Cancer, Organismal Injury and Abnormalities, Reproductive System Disease	mammary tumor	mammary tumor	6.89E-24
Organismal Survival	perinatal death	perinatal death	1.13E-23
Cellular Development, Connective Tissue Development and Function, Tissue Development	differentiation	differentiation of connective tissue	1.68E-23
Cancer, Gastrointestinal Disease, Organismal Injury and Abnormalities	colon cancer	colon cancer	1.81E-23
Nervous System Development and Function, Tissue Development	morphogenesis	morphogenesis of nervous tissue	2.00E-23
Tissue Development	development	development of epithelial tissue	2.00E-23
Cancer, Organismal Injury and Abnormalities, Reproductive System Disease	breast cancer	breast cancer	2.81E-23
Cancer, Gastrointestinal Disease, Organismal Injury and Abnormalities	gastrointestinal adenocarcinoma	gastrointestinal adenocarcinoma	4.69E-23
Cell Morphology, Cellular Development, Cellular Growth and Proliferation, Nervous System	morphogenesis	morphogenesis of neurons	5.65E-23

Cellular Development, Cellular Growth and Proliferation, Nervous System Development and	growth	growth of neurites	6.16E-23
Cancer, Hematological Disease, Organismal Injury and Abnormalities	hematological neoplasia	hematological neoplasia	1.12E-22
Cancer, Hematological Disease, Organismal Injury and Abnormalities	lymphohematopoietic neoplasia	lymphohematopoietic neoplasia	1.30E-22
Cardiovascular System Development and Function, Embryonic Development, Organ Develo	cardiogenesis	cardiogenesis	1.68E-22
Cellular Growth and Proliferation, Lymphoid Tissue Structure and Development	proliferation	proliferation of lymphatic system cells	1.85E-22
Cellular Development, Cellular Growth and Proliferation	proliferation	proliferation of blood cells	1.96E-22
Embryonic Development, Organismal Development	development	development of abdomen	2.30E-22
Nervous System Development and Function	development	sensory system development	2.66E-22
Embryonic Development, Organ Development, Organismal Development	development	development of lymphatic system	4.09E-22
Cancer, Hematological Disease, Organismal Injury and Abnormalities	lymphocytic neoplasm	lymphocytic neoplasm	5.70E-22
Organismal Survival	survival	survival of organism	7.73E-22
Cell Morphology	sprouting	sprouting	8.38E-22
Cancer, Gastrointestinal Disease, Organismal Injury and Abnormalities	colon carcinoma	colon carcinoma	9.75E-22
Cardiovascular System Development and Function, Organ Morphology, Organismal Develop	morphology	morphology of heart	1.06E-21
Digestive System Development and Function	morphology	morphology of digestive system	1.15E-21
Cancer, Gastrointestinal Disease, Organismal Injury and Abnormalities	colorectal adenocarcinoma	colorectal adenocarcinoma	1.18E-21
Cell Morphology, Cellular Assembly and Organization, Cellular Development, Cellular Functi	morphogenesis	morphogenesis of neurites	1.28E-21
Infectious Diseases	infection	Viral Infection	1.68E-21
Cancer, Organismal Injury and Abnormalities	growth	growth of tumor	2.13E-21
Cellular Development, Cellular Growth and Proliferation, Nervous System Development and	outgrowth	outgrowth of neurites	3.76E-21
Cell Death and Survival, Organismal Injury and Abnormalities	necrosis	necrosis of epithelial tissue	5.02E-21
Behavior	behavior	behavior	8.53E-21
Cellular Growth and Proliferation	outgrowth	outgrowth of cells	1.44E-20
Cellular Growth and Proliferation, Tissue Development	proliferation	proliferation of epithelial cells	1.71E-20
Nervous System Development and Function, Tissue Morphology	quantity	quantity of neurons	1.77E-20
Cellular Development, Hematological System Development and Function	differentiation	differentiation of blood cells	2.27E-20
Cancer, Hematological Disease, Organismal Injury and Abnormalities	lymphohematopoietic cancer	lymphohematopoietic cancer	2.91E-20
Cancer, Hematological Disease, Organismal Injury and Abnormalities	hematologic cancer	hematologic cancer	3.74E-20
Connective Tissue Development and Function, Skeletal and Muscular System Development	morphology	morphology of bone	6.79E-20
Developmental Disorder	midline defect	midline defect	7.06E-20
Cancer, Gastrointestinal Disease, Organismal Injury and Abnormalities	colon adenocarcinoma	colon adenocarcinoma	7.93E-20
Cellular Development, Connective Tissue Development and Function, Tissue Development	differentiation	differentiation of connective tissue cells	8.25E-20
Cancer, Organismal Injury and Abnormalities	melanoma	melanoma	8.57E-20
Cell Death and Survival	apoptosis	apoptosis of neurons	1.04E-19
Hematological System Development and Function, Tissue Morphology	quantity	quantity of blood cells	1.42E-19
Cancer, Organismal Injury and Abnormalities	neoplasia	Lymphoid Cancer and Tumors	1.67E-19
Cardiovascular System Development and Function, Cellular Development, Cellular Function	development	endothelial cell development	1.83E-19
Cellular Development	branching	branching of cells	1.83E-19
Cellular Development, Cellular Growth and Proliferation, Hematological System Developme	proliferation	proliferation of immune cells	1.89E-19
Lymphoid Tissue Structure and Development, Tissue Morphology	quantity	quantity of lymphatic system cells	2.27E-19
Organismal Survival	neonatal death	neonatal death	2.94E-19
Embryonic Development, Nervous System Development and Function, Organ Development	formation	formation of eye	3.15E-19
Connective Tissue Development and Function, Tissue Development	growth	growth of connective tissue	3.42E-19
Cardiovascular System Development and Function	morphology	morphology of vasculature	3.86E-19
Cardiovascular Disease, Cardiovascular System Development and Function, Organ Morphol	abnormal morphology	abnormal morphology of heart	3.96E-19
Organismal Development, Reproductive System Development and Function	development	development of reproductive system	4.11E-19

Cardiovascular Disease, Cardiovascular System Development and Function	abnormal morphology	abnormal morphology of cardiovascular system	4.52E-19
Cardiovascular System Development and Function, Tissue Development	development	development of cardiovascular tissue	7.25E-19
Cellular Development, Cellular Growth and Proliferation, Embryonic Development, Lympho	development	development of lymphatic system cells	8.56E-19
Cancer, Organismal Injury and Abnormalities	metastasis	metastasis	9.23E-19
Cancer, Organismal Injury and Abnormalities	advanced malignant tumor	advanced malignant tumor	9.33E-19
Cellular Development, Cellular Growth and Proliferation, Hematological System Developme	proliferation	proliferation of lymphocytes	9.66E-19
Cellular Growth and Proliferation, Connective Tissue Development and Function, Tissue Dev	proliferation	proliferation of connective tissue cells	9.76E-19
Skeletal and Muscular System Development and Function	morphology	morphology of skeleton	1.34E-18
Cellular Development, Tissue Development	differentiation	differentiation of epithelial tissue	1.36E-18
Cell Cycle	interphase	interphase	1.38E-18
Cellular Development, Cellular Growth and Proliferation, Hematological System Developme	proliferation	proliferation of mononuclear leukocytes	1.70E-18
Hematological Disease, Immunological Disease	lymphoproliferative disorder	lymphoproliferative disorder	1.75E-18
Connective Tissue Development and Function, Connective Tissue Disorders, Organismal Inju	abnormal morphology	abnormal morphology of bone	1.81E-18
Cellular Growth and Proliferation	colony formation	colony formation of cells	1.88E-18
Embryonic Development, Organismal Development, Skeletal and Muscular System Develop	development	limb development	2.14E-18
Embryonic Development, Organismal Development	morphogenesis	morphogenesis of head	2.15E-18
Cancer, Hematological Disease, Immunological Disease, Organismal Injury and Abnormalitie	lymphoproliferative malignancy	lymphoproliferative malignancy	2.20E-18
Cell Death and Survival	cell death	cell death of connective tissue cells	2.25E-18
Hematological System Development and Function, Tissue Morphology	quantity	quantity of leukocytes	2.48E-18
Reproductive System Development and Function	morphology	morphology of reproductive system	2.62E-18
Cell Morphology, Nervous System Development and Function, Tissue Morphology	morphology	morphology of neurites	2.72E-18
Respiratory System Development and Function	development	respiratory system development	3.08E-18
Embryonic Development, Hair and Skin Development and Function, Organ Development, O	formation	formation of skin	3.31E-18
Digestive System Development and Function	development	development of gastrointestinal tract	3.41E-18
Cancer, Organismal Injury and Abnormalities	connective or soft tissue tumor	connective or soft tissue tumor	3.81E-18
Cell Morphology	shape change	shape change of neurites	4.17E-18
Cancer	transformation	cell transformation	4.73E-18
Cancer, Hematological Disease, Organismal Injury and Abnormalities	lymphoid cancer	lymphoid cancer	4.78E-18
Cellular Movement, Nervous System Development and Function	migration	migration of neurons	5.22E-18
Tissue Morphology	morphology	morphology of connective tissue	5.69E-18
Cell Morphology	shape change	shape change of neurons	5.70E-18
Cellular Development	differentiation	differentiation of tumor cell lines	7.04E-18
Cellular Development	differentiation	differentiation of central nervous system cells	7.07E-18
Cardiovascular System Development and Function, Cellular Development, Cellular Function	proliferation	proliferation of endothelial cells	7.68E-18
Cellular Development, Embryonic Development, Organismal Development, Tissue Developm	differentiation	differentiation of embryonic tissue	8.40E-18
Cellular Development, Cellular Growth and Proliferation, Embryonic Development, Organis	proliferation	proliferation of embryonic cells	8.41E-18
Cardiovascular System Development and Function	morphology	morphology of vessel	8.92E-18
Cell Death and Survival, Organismal Injury and Abnormalities	cell death	cell death of epithelial cells	9.03E-18
Behavior	locomotion	locomotion	9.22E-18
Cancer, Organismal Injury and Abnormalities	adenoma	adenoma	1.11E-17
Cardiovascular System Development and Function, Cellular Movement	cell movement	cell movement of endothelial cells	1.17E-17
Cellular Growth and Proliferation	colony formation	colony formation	1.18E-17
Developmental Disorder, Gastrointestinal Disease	congenital anomaly of digestive system	congenital anomaly of digestive system	1.21E-17
Digestive System Development and Function, Gastrointestinal Disease	abnormal morphology	abnormal morphology of digestive system	1.29E-17
Cell Morphology, Cellular Assembly and Organization, Cellular Development, Cellular Functi	branching	branching of neurons	1.93E-17
Gene Expression	binding	binding of DNA	2.06E-17

Connective Tissue Disorders, Organismal Injury and Abnormalities, Skeletal and Muscular D	abnormal morphology	abnormal morphology of skeleton	2.25E-17
Cell Death and Survival	cell viability	cell viability of tumor cell lines	2.40E-17
Cellular Development, Cellular Growth and Proliferation, Connective Tissue Development a	proliferation	proliferation of fibroblast cell lines	2.44E-17
Neurological Disease, Organismal Injury and Abnormalities	seizure disorder	seizure disorder	2.73E-17
Lymphoid Tissue Structure and Development, Tissue Morphology	quantity	quantity of lymphoid cells	2.75E-17
Hematological System Development and Function, Tissue Morphology	quantity	quantity of mononuclear leukocytes	2.79E-17
Cell Morphology, Cellular Assembly and Organization, Cellular Development, Cellular Functi	branching	branching of neurites	3.04E-17
Developmental Disorder	multiple congenital anomalies	multiple congenital anomalies	3.05E-17
Cellular Development, Cellular Growth and Proliferation, Hematological System Developme	development	development of blood cells	3.87E-17
Post-Translational Modification	phosphorylation	phosphorylation of protein	3.87E-17
Dermatological Diseases and Conditions, Organismal Injury and Abnormalities	skin lesion	skin lesion	4.04E-17
Cellular Development, Cellular Growth and Proliferation, Hematological System Developme	development	development of mononuclear leukocytes	4.42E-17
Hematological System Development and Function, Lymphoid Tissue Structure and Develop	quantity	quantity of lymphocytes	4.97E-17
Organismal Injury and Abnormalities, Reproductive System Development and Function, Rep	abnormal morphology	abnormal morphology of reproductive system	5.89E-17
Cardiovascular System Development and Function, Cellular Movement	migration	migration of endothelial cells	6.33E-17
Tissue Development	formation	formation of gland	8.58E-17
Cellular Development, Cellular Growth and Proliferation, Embryonic Development, Hemato	development	development of lymphocytes	1.04E-16
Cancer, Dermatological Diseases and Conditions, Organismal Injury and Abnormalities	skin tumor	skin tumor	1.09E-16
Cellular Development	differentiation	differentiation of lymphatic system cells	1.44E-16
Hematological System Development and Function, Lymphoid Tissue Structure and Develop	morphology	morphology of lymphoid organ	1.60E-16
Cellular Development, Tissue Development	differentiation	differentiation of epithelial cells	1.60E-16
Embryonic Development, Organ Development, Organismal Development, Skeletal and Mus	formation	formation of muscle	1.79E-16
Cell Death and Survival	cell death	cell death of blood cells	1.84E-16
Cancer, Organismal Injury and Abnormalities	cancer	cancer of secretory structure	2.09E-16
Hereditary Disorder, Organismal Injury and Abnormalities	autosomal dominant disease	autosomal dominant disease	2.45E-16
Cell Morphology	morphology	morphology of cellular protrusions	2.95E-16
Cellular Function and Maintenance	homeostasis	homeostasis of blood cells	3.38E-16
Cellular Development, Nervous System Development and Function	differentiation	differentiation of neuroglia	3.89E-16
Cellular Development, Cellular Growth and Proliferation, Hematological System Developme	development	development of leukocytes	4.18E-16
Hematological System Development and Function, Lymphoid Tissue Structure and Develop	morphology	morphology of lymphoid tissue	4.80E-16
Cell-To-Cell Signaling and Interaction, Cellular Assembly and Organization, Cellular Function	assembly	assembly of intercellular junctions	5.12E-16
Cancer, Organismal Injury and Abnormalities	ductal carcinoma	ductal carcinoma	5.41E-16
Cardiovascular System Development and Function, Tissue Morphology	morphology	morphology of blood vessel	6.19E-16
Cancer, Organismal Injury and Abnormalities	urogenital cancer	urogenital cancer	7.26E-16
Cellular Function and Maintenance	homeostasis	Lymphocyte homeostasis	8.21E-16
Cellular Assembly and Organization	formation	formation of plasma membrane	8.44E-16
Cellular Assembly and Organization	formation	formation of cellular membrane	8.55E-16
Hematological System Development and Function, Lymphoid Tissue Structure and Develop	quantity	quantity of T lymphocytes	1.08E-15
Cancer, Dermatological Diseases and Conditions, Organismal Injury and Abnormalities	skin cancer	skin cancer	1.09E-15
Cancer, Neurological Disease, Organismal Injury and Abnormalities	central nervous system cancer	central nervous system cancer	1.15E-15
Cardiovascular Disease, Cardiovascular System Development and Function	abnormal morphology	abnormal morphology of vasculature	1.16E-15
Skeletal and Muscular System Development and Function	morphology	morphology of muscle	1.55E-15
Embryonic Development, Organismal Development, Tissue Development	growth	growth of embryonic tissue	1.55E-15
Cellular Development, Hematological System Development and Function, Hematopoiesis, L	differentiation	differentiation of leukocytes	1.73E-15
Cellular Movement	invasion	invasion of carcinoma cell lines	1.79E-15
Cancer, Organismal Injury and Abnormalities, Reproductive System Disease	tumorigenesis	tumorigenesis of genital organ	2.23E-15

Cellular Development, Cellular Growth and Proliferation, Hematological System Developme	cell proliferation	cell proliferation of T lymphocytes	2.37E-15
Cancer, Organismal Injury and Abnormalities, Reproductive System Disease	genital tumor	genital tumor	2.63E-15
Neurological Disease, Organismal Injury and Abnormalities	seizures	seizures	2.74E-15
Cell Morphology, Nervous System Development and Function, Neurological Disease, Tissue	abnormal morphology	abnormal morphology of neurites	2.77E-15
Connective Tissue Disorders, Organismal Injury and Abnormalities, Skeletal and Muscular D	abnormal morphology	abnormal morphology of axial skeleton	2.96E-15
Organismal Injury and Abnormalities	cytosis	Cytosis	3.09E-15
Skeletal and Muscular System Development and Function	morphology	morphology of axial skeleton	3.63E-15
Cellular Development	maturation	maturation of cells	4.26E-15
Cell-To-Cell Signaling and Interaction, Cellular Assembly and Organization, Cellular Function	development	development of gap junctions	4.27E-15
Connective Tissue Development and Function, Tissue Development	development	development of connective tissue	4.65E-15
Hematological System Development and Function, Hematopoiesis, Tissue Morphology	quantity	quantity of hematopoietic cells	4.78E-15
Cellular Development, Nervous System Development and Function	differentiation	differentiation of astrocytes	4.86E-15
Cell Death and Survival, Organismal Injury and Abnormalities, Renal and Urological Disease	necrosis	necrosis of kidney	5.09E-15
Neurological Disease, Skeletal and Muscular Disorders	neuromuscular disease	neuromuscular disease	5.45E-15
Cancer, Endocrine System Disorders, Gastrointestinal Disease, Organismal Injury and Abnor	pancreatic tumor	pancreatic tumor	5.80E-15
Embryonic Development, Organismal Development, Tissue Morphology	size	size of embryo	6.74E-15
Cell Morphology	shape change	shape change of axons	7.82E-15
Cell-To-Cell Signaling and Interaction, Cellular Assembly and Organization, Cellular Develop	developmental process	developmental process of synapse	8.19E-15
Cellular Movement, Nervous System Development and Function	guidance	guidance of axons	8.28E-15
Cell Cycle	S phase	S phase	8.34E-15
Cell-mediated Immune Response, Cellular Function and Maintenance, Hematological Syster	homeostasis	T cell homeostasis	8.89E-15
Cancer, Organismal Injury and Abnormalities	breast or ovarian carcinoma	breast or ovarian carcinoma	9.89E-15
Cancer, Organismal Injury and Abnormalities	neoplasia	neoplasia of cells	9.96E-15
Hematological System Development and Function, Hematopoiesis, Tissue Morphology	quantity	quantity of hematopoietic progenitor cells	1.03E-14
Organismal Development, Tissue Development	development	development of exocrine gland	1.05E-14
Cell Death and Survival, Organismal Injury and Abnormalities	cell death	cell death of epithelial cell lines	1.20E-14
Cell Death and Survival	cell death	cell death of lymphatic system cells	1.59E-14
Embryonic Development, Nervous System Development and Function, Organ Development	formation	formation of forebrain	1.63E-14
Cancer, Gastrointestinal Disease, Organismal Injury and Abnormalities	stomach tumor	stomach tumor	2.04E-14
Cancer, Organismal Injury and Abnormalities	benign connective or soft tissue neoplasm	benign connective or soft tissue neoplasm	2.18E-14
Cell Morphology	size	size of cells	2.42E-14
Cell Signaling	protein kinase cascade	protein kinase cascade	2.64E-14
Cellular Development, Cellular Growth and Proliferation, Hematological System Developme	proliferation	proliferation of hematopoietic cells	3.25E-14
Cellular Development, Cellular Growth and Proliferation, Hair and Skin Development and Fu	proliferation	proliferation of epithelial cell lines	3.72E-14
Connective Tissue Disorders, Developmental Disorder, Gastrointestinal Disease, Organisma	orofacial dysmorphism	orofacial dysmorphism	3.98E-14
Cancer, Endocrine System Disorders, Gastrointestinal Disease, Organismal Injury and Abnor	pancreatic cancer	pancreatic cancer	4.45E-14
Cell Death and Survival	cell death	cell death of immune cells	4.46E-14
Organismal Development	morphology	morphology of face	5.12E-14
Cell Cycle	interphase	arrest in interphase	5.17E-14
Behavior	learning	learning	5.67E-14
Cancer, Endocrine System Disorders, Gastrointestinal Disease, Organismal Injury and Abnor	carcinoma	carcinoma in pancreas	5.75E-14
Cancer, Organismal Injury and Abnormalities	tumor	tumor in nervous system	5.78E-14
Organ Morphology	abnormal morphology	abnormal morphology of gland	5.95E-14
Hematological System Development and Function, Hematopoiesis	development	development of hematopoietic system	5.95E-14
Cell Death and Survival, Organismal Injury and Abnormalities, Renal and Urological Disease	cell death	cell death of kidney cells	5.96E-14
Cell-mediated Immune Response, Cellular Development, Cellular Function and Maintenanc	development	T cell development	6.25E-14

Organismal Development	size	size of animal	7.20E-14
Behavior	cognition	cognition	7.38E-14
Embryonic Development, Nervous System Development and Function, Organ Development	formation	formation of rhombencephalon	7.47E-14
Organ Morphology	morphology	morphology of gland	7.54E-14
Cellular Development, Hematological System Development and Function, Hematopoiesis	differentiation	differentiation of hematopoietic cells	8.02E-14
Cancer, Gastrointestinal Disease, Organismal Injury and Abnormalities	gastric cancer	gastric cancer	8.48E-14
Cancer, Organismal Injury and Abnormalities	pelvic cancer	pelvic cancer	9.60E-14
Cancer, Organismal Injury and Abnormalities, Reproductive System Disease	tumorigenesis	tumorigenesis of reproductive tract	1.14E-13
Cancer, Organismal Injury and Abnormalities, Reproductive System Disease	benign neoplasm of female genital organ	benign neoplasm of female genital organ	1.32E-13
Connective Tissue Disorders, Developmental Disorder, Gastrointestinal Disease, Organisma	congenital malformation	congenital malformation of face	1.39E-13
Cancer, Endocrine System Disorders, Organismal Injury and Abnormalities	endocrine gland tumor	endocrine gland tumor	1.40E-13
Cancer, Organismal Injury and Abnormalities, Reproductive System Disease	female genital neoplasm	female genital neoplasm	1.43E-13
Cancer, Organismal Injury and Abnormalities	pelvic tumor	pelvic tumor	1.47E-13
Cellular Development, Hematological System Development and Function, Hematopoiesis	differentiation	differentiation of hematopoietic progenitor cells	1.53E-13
Connective Tissue Disorders, Developmental Disorder, Gastrointestinal Disease, Organisma	orofacial cleft	orofacial cleft	1.54E-13
Embryonic Development, Organ Development, Organismal Development, Reproductive Sys	formation	formation of mammary gland	1.54E-13
Cancer, Organismal Injury and Abnormalities	connective tissue tumor	connective tissue tumor	1.56E-13
Endocrine System Disorders, Gastrointestinal Disease, Organismal Injury and Abnormalities	pancreatic duct disorder	pancreatic duct disorder	1.70E-13
Cell Cycle	interphase	interphase of tumor cell lines	1.73E-13
Organismal Development	development	development of endocrine gland	1.93E-13
Cancer, Organismal Injury and Abnormalities, Reproductive System Disease	carcinoma	carcinoma in breast	1.96E-13
Cancer, Cell Death and Survival, Organismal Injury and Abnormalities, Tumor Morphology	cell death	cell death of cancer cells	2.17E-13
Hematological System Development and Function, Lymphoid Tissue Structure and Develop	morphology	morphology of secondary lymphoid organ	2.34E-13
Cancer, Endocrine System Disorders, Gastrointestinal Disease, Organismal Injury and Abnor	pancreatic ductal adenocarcinoma	pancreatic ductal adenocarcinoma	2.38E-13
Cancer, Endocrine System Disorders, Gastrointestinal Disease, Organismal Injury and Abnor	pancreatic adenocarcinoma	pancreatic adenocarcinoma	2.39E-13
Cardiovascular System Development and Function, Cellular Movement	movement	movement of vascular endothelial cells	2.58E-13
Cell-To-Cell Signaling and Interaction, Nervous System Development and Function	neurotransmission	neurotransmission	2.61E-13
Cancer, Neurological Disease, Organismal Injury and Abnormalities	central nervous system tumor	central nervous system tumor	2.64E-13
Embryonic Development	quantity	quantity of embryo	2.67E-13
Cellular Development, Cellular Growth and Proliferation	proliferation	proliferation of stem cells	2.74E-13
Cell Morphology, Cellular Assembly and Organization, Cellular Development, Cellular Functi	branching	branching of axons	2.85E-13
Cellular Development, Hematological System Development and Function, Hematopoiesis, L	differentiation	differentiation of lymphocytes	2.86E-13
Cellular Growth and Proliferation	proliferation	arrest in proliferation of cells	2.87E-13
Cancer	hyperplasia	hyperplasia of tissue	2.87E-13
Cellular Development, Cellular Growth and Proliferation	cell proliferation	cell proliferation of carcinoma cell lines	3.16E-13
Cancer, Gastrointestinal Disease, Organismal Injury and Abnormalities	gastroesophageal cancer	gastroesophageal cancer	3.61E-13
Cancer, Organismal Injury and Abnormalities, Skeletal and Muscular Disorders	muscle tumor	muscle tumor	3.74E-13
Cardiovascular System Development and Function, Cellular Movement	migration	migration of vascular endothelial cells	3.76E-13
Connective Tissue Disorders, Developmental Disorder, Gastrointestinal Disease, Organisma	cleft palate syndrome	cleft palate syndrome	4.03E-13
Cancer, Dermatological Diseases and Conditions, Organismal Injury and Abnormalities	malignant cutaneous melanoma cancer	malignant cutaneous melanoma cancer	4.04E-13
Auditory and Vestibular System Development and Function, Embryonic Development, Orga	development	development of ear	4.32E-13
Molecular Transport, Protein Trafficking	internalization	internalization of protein	4.42E-13
Nervous System Development and Function, Organ Morphology, Organismal Development	morphology	morphology of rhombencephalon	4.77E-13
Cellular Development, Cellular Growth and Proliferation, Hematological System Developme	proliferation	proliferation of hematopoietic progenitor cells	4.89E-13
Neurological Disease, Organismal Injury and Abnormalities, Psychological Disorders	disorder of basal ganglia	disorder of basal ganglia	4.91E-13
Cancer, Organismal Injury and Abnormalities	sarcoma	sarcoma	4.99E-13

Cellular Development, Cellular Growth and Proliferation	proliferation	proliferation of leukemia cell lines	5.47E-13
Cellular Development, Embryonic Development, Organismal Development	differentiation	differentiation of embryonic cells	5.69E-13
Hematological System Development and Function, Lymphoid Tissue Structure and Developı	morphology	morphology of spleen	5.70E-13
Cancer, Cellular Development, Cellular Growth and Proliferation, Organismal Injury and Abı	proliferation	proliferation of tumor cells	5.95E-13
Embryonic Development, Organismal Development	morphogenesis	morphogenesis of embryo	6.63E-13
Hair and Skin Development and Function, Organ Development	growth	growth of skin	6.63E-13
Neurological Disease, Organismal Injury and Abnormalities, Psychological Disorders	dementia	Dementia	6.98E-13
Cell Cycle	cell cycle progression	arrest in cell cycle progression	7.32E-13
Amino Acid Metabolism, Post-Translational Modification, Small Molecule Biochemistry	phosphorylation	phosphorylation of L-amino acid	7.70E-13
Developmental Disorder	hypertrophy	Hypertrophy	7.87E-13
Embryonic Development, Nervous System Development and Function, Organ Development	formation	formation of cerebellum	7.96E-13
Nervous System Development and Function, Organ Morphology, Tissue Morphology	quantity	quantity of brain cells	7.96E-13
Cellular Movement, Hematological System Development and Function, Immune Cell Traffic	cell movement	cell movement of leukocytes	8.48E-13
Cellular Development, Hematological System Development and Function, Hematopoiesis, L	differentiation	differentiation of mononuclear leukocytes	8.70E-13
Cancer, Organismal Injury and Abnormalities, Tumor Morphology	progression	progression of tumor	9.76E-13
Skeletal and Muscular Disorders, Skeletal and Muscular System Development and Function	abnormal morphology	abnormal morphology of muscle	9.99E-13
Cancer, Organismal Injury and Abnormalities	malignant solid tumor	advanced malignant solid tumor	1.02E-12
Cancer, Organismal Injury and Abnormalities, Reproductive System Disease	genital tract cancer	genital tract cancer	1.05E-12
Embryonic Development, Organ Development, Organismal Development, Respiratory Syste	formation	formation of lung	1.07E-12
Cellular Development, Embryonic Development, Hair and Skin Development and Function,	differentiation	differentiation of skin	1.09E-12
Digestive System Development and Function, Hepatic System Development and Function, (	morphology	morphology of liver	1.10E-12
Cell Death and Survival	apoptosis	apoptosis of lymphatic system cells	1.11E-12
Cancer, Organismal Injury and Abnormalities	tumorigenesis	tumorigenesis of malignant tumor	1.11E-12
Cancer, Hematological Disease, Immunological Disease, Organismal Injury and Abnormalitie	lymphocytic cancer	lymphocytic cancer	1.11E-12
Hematological System Development and Function, Immunological Disease, Lymphoid Tissu	abnormal morphology	abnormal morphology of lymphoid organ	1.22E-12
Cancer, Organismal Injury and Abnormalities	mesenchymal tumor	mesenchymal tumor	1.25E-12
Organismal Development	size	size of head	1.38E-12
Cancer, Organismal Injury and Abnormalities	cancer	cancer of cells	1.62E-12
Cancer, Hematological Disease, Organismal Injury and Abnormalities	B-cell neoplasm	B-cell neoplasm	1.77E-12
Nervous System Development and Function, Organ Morphology, Organismal Development	morphology	morphology of cerebellum	1.77E-12
Cellular Movement	invasion	invasion of breast cancer cell lines	1.99E-12
Cancer, Cell Death and Survival, Organismal Injury and Abnormalities, Tumor Morphology	cell death	cell death of tumor cells	2.05E-12
Skeletal and Muscular System Development and Function	development	development of axial skeleton	2.12E-12
Molecular Transport, Protein Trafficking	transport	transport of protein	2.15E-12
Cell Death and Survival	cell death	cell death of fibroblast cell lines	2.15E-12
Cell Morphology, Nervous System Development and Function, Tissue Morphology	morphology	morphology of axons	2.17E-12
Cancer, Organismal Injury and Abnormalities	malignant connective or soft tissue neoplasm	malignant connective or soft tissue neoplasm	2.23E-12
Cell Death and Survival, Organismal Injury and Abnormalities, Renal and Urological Disease	cell death	cell death of kidney cell lines	2.30E-12
Developmental Disorder, Neurological Disease, Organismal Injury and Abnormalities	congenital encephalopathy	congenital encephalopathy	2.49E-12
Neurological Disease, Organismal Injury and Abnormalities, Psychological Disorders	tauopathy	tauopathy	2.59E-12
Nervous System Development and Function, Neurological Disease, Organ Morphology, Org	abnormal morphology	abnormal morphology of rhombencephalon	2.66E-12
Cancer, Organismal Injury and Abnormalities, Skeletal and Muscular Disorders	smooth muscle tumor	smooth muscle tumor	2.74E-12
Cell Death and Survival	apoptosis	apoptosis of blood cells	2.85E-12
Cellular Movement	cell movement	cell movement of blood cells	3.00E-12
Metabolic Disease	glucose metabolism disorder	glucose metabolism disorder	3.10E-12
Cancer, Cell Death and Survival, Organismal Injury and Abnormalities, Tumor Morphology	necrosis	necrosis of tumor	3.12E-12

Cancer, Organismal Injury and Abnormalities	growth	growth of malignant tumor	3.23E-12
Neurological Disease, Organismal Injury and Abnormalities	epilepsy	epilepsy	3.38E-12
Cancer, Organismal Injury and Abnormalities	serous neoplasm	serous neoplasm	3.56E-12
Respiratory System Development and Function	morphology	morphology of respiratory system	3.57E-12
Cancer, Endocrine System Disorders, Organismal Injury and Abnormalities, Reproductive Sy	gonadal tumor	gonadal tumor	3.64E-12
Lipid Metabolism, Molecular Transport, Small Molecule Biochemistry	concentration	concentration of lipid	3.90E-12
Cardiovascular System Development and Function, Organ Morphology, Organismal Develop	morphology	morphology of heart ventricle	4.00E-12
Nervous System Development and Function, Neurological Disease, Organ Morphology, Org	abnormal morphology	abnormal morphology of cerebellum	4.10E-12
Skeletal and Muscular System Development and Function	morphogenesis	morphogenesis of skeletal system	4.13E-12
Cell-To-Cell Signaling and Interaction, Nervous System Development and Function	potentiation	potentiation of synapse	4.34E-12
Developmental Disorder, Neurological Disease, Organismal Injury and Abnormalities	congenital malformation	congenital malformation of brain	4.38E-12
Cellular Movement, Immune Cell Trafficking	migration	leukocyte migration	4.71E-12
Cellular Movement	homing	homing of cells	4.83E-12
Lymphoid Tissue Structure and Development, Organ Morphology, Tissue Morphology	quantity	quantity of lymphoid organ	4.94E-12
Cell Morphology, Nervous System Development and Function	innervation	innervation	5.00E-12
Cellular Movement	cell movement	cell movement of melanoma cell lines	5.08E-12
Cell Death and Survival	cell death	cell death of lymphoid cells	5.19E-12
Cellular Development, Cellular Growth and Proliferation	cell proliferation	cell proliferation of breast cancer cell lines	5.33E-12
Organismal Injury and Abnormalities, Tissue Morphology	abnormal morphology	abnormal morphology of epithelial tissue	5.61E-12
Infectious Diseases	infection	infection by RNA virus	5.62E-12
Embryonic Development, Organismal Development, Tissue Development	closure	closure of embryonic tissue	6.25E-12
Reproductive System Development and Function	fertility	fertility	6.26E-12
Cell Cycle	cleavage	cleavage of cells	6.80E-12
Metabolic Disease	amyloidosis	amyloidosis	6.83E-12
Cancer, Gastrointestinal Disease, Organismal Injury and Abnormalities	gastric epithelial cancer	gastric epithelial cancer	6.89E-12
Cancer, Gastrointestinal Disease, Organismal Injury and Abnormalities	gastro-esophageal carcinoma	gastro-esophageal carcinoma	6.97E-12
Cancer, Organismal Injury and Abnormalities	serous adenocarcinoma	serous adenocarcinoma	7.13E-12
Cancer, Organismal Injury and Abnormalities	transformation	transformation of fibroblasts	7.16E-12
Cell-To-Cell Signaling and Interaction, Cellular Assembly and Organization, Cellular Function	formation	formation of focal adhesions	7.17E-12
Connective Tissue Development and Function, Organismal Development, Skeletal and Musc	morphology	morphology of skull	7.36E-12
Gastrointestinal Disease, Organismal Injury and Abnormalities	benign oral disorder	benign oral disorder	7.54E-12
Cellular Development, Cellular Growth and Proliferation	proliferation	proliferation of prostate cancer cell lines	7.65E-12
Cell-To-Cell Signaling and Interaction, Nervous System Development and Function	long-term potentiation	long-term potentiation	7.72E-12
Cell Death and Survival, Organismal Injury and Abnormalities	apoptosis	apoptosis of epithelial cell lines	8.39E-12
Embryonic Development, Hematological System Development and Function, Lymphoid Tiss	formation	formation of lymphoid tissue	8.55E-12
Skeletal and Muscular System Development and Function	fusion	fusion of bone	8.56E-12
Cancer, Neurological Disease, Organismal Injury and Abnormalities	primitive neuroectodermal tumor	primitive neuroectodermal tumor	8.60E-12
Cancer, Hematological Disease, Immunological Disease, Organismal Injury and Abnormalitie	lymphocytic leukemia	lymphocytic leukemia	8.69E-12
Cell Death and Survival	cell death	cell death of lymphocytes	8.77E-12
Cell Death and Survival	apoptosis	apoptosis of connective tissue cells	8.90E-12
Cellular Development, Connective Tissue Development and Function, Hematological System	differentiation	differentiation of red blood cells	9.10E-12
Neurological Disease	neurological signs	neurological signs	9.43E-12
Metabolic Disease, Neurological Disease, Organismal Injury and Abnormalities, Psychologic	Alzheimer disease	Alzheimer disease	9.58E-12
Cancer, Organismal Injury and Abnormalities	fibrous tissue tumor	fibrous tissue tumor	9.91E-12
DNA Replication, Recombination, and Repair	synthesis	synthesis of DNA	1.02E-11
Endocrine System Disorders, Organismal Injury and Abnormalities, Reproductive System Dis	ovarian lesion	ovarian lesion	1.10E-11

Cell Morphology, Tissue Morphology	morphology	morphology of connective tissue cells	1.10E-11
Cellular Development	differentiation	differentiation of stem cells	1.13E-11
Connective Tissue Development and Function, Tissue Morphology	quantity	quantity of connective tissue	1.13E-11
Embryonic Development, Organismal Development, Skeletal and Muscular System Develop	development	development of forelimb	1.26E-11
Organismal Development, Renal and Urological System Development and Function	development	development of urinary tract	1.28E-11
Organ Morphology, Organismal Injury and Abnormalities, Reproductive System Developme	abnormal morphology	abnormal morphology of genital organ	1.30E-11
Organ Morphology, Reproductive System Development and Function	morphology	morphology of genital organ	1.35E-11
Embryonic Development, Organ Development, Organismal Development, Renal and Urolog	formation	formation of kidney	1.37E-11
Cancer, Organismal Injury and Abnormalities	genitourinary carcinoma	genitourinary carcinoma	1.38E-11
Lymphoid Tissue Structure and Development, Tissue Morphology	quantity	quantity of lymphoid tissue	1.46E-11
Cancer, Hematological Disease, Immunological Disease, Organismal Injury and Abnormalitie	leukemia	leukemia	1.47E-11
Cellular Development, Skeletal and Muscular System Development and Function, Tissue De	differentiation	differentiation of muscle	1.51E-11
Cancer, Endocrine System Disorders, Organismal Injury and Abnormalities, Reproductive Sy	neoplasia	Ovarian Cancer and Tumors	1.54E-11
Cancer, Organismal Injury and Abnormalities	thoracic cancer	thoracic cancer	1.65E-11
Cancer, Organismal Injury and Abnormalities	metastatic solid tumor	metastatic solid tumor	1.66E-11
Cancer, Organismal Injury and Abnormalities	thoracic neoplasm	thoracic neoplasm	1.67E-11
Connective Tissue Development and Function, Nervous System Development and Function,	morphology	morphology of vertebrae	1.71E-11
Cellular Development, Cellular Growth and Proliferation, Nervous System Development and	growth	growth of axons	1.72E-11
Cell Cycle	G1 phase	G1 phase	1.75E-11
Lipid Metabolism, Small Molecule Biochemistry	synthesis	synthesis of lipid	1.85E-11
Cancer, Gastrointestinal Disease, Organismal Injury and Abnormalities	upper gastrointestinal tract cancer	upper gastrointestinal tract cancer	1.90E-11
Molecular Transport	secretion	secretion of molecule	1.95E-11
Cellular Function and Maintenance	endocytosis	endocytosis	2.03E-11
Neurological Disease	neurodegeneration	Neurodegeneration	2.05E-11
Cell Morphology, Tissue Development	tubulation	tubulation of cells	2.06E-11
Nervous System Development and Function, Tissue Morphology	quantity	quantity of central nervous system cells	2.08E-11
Organismal Injury and Abnormalities	bleeding	Bleeding	2.33E-11
Cell-To-Cell Signaling and Interaction	adhesion	adhesion of connective tissue cells	2.47E-11
Cell Death and Survival	degradation	cellular degradation	2.55E-11
Cancer, Endocrine System Disorders, Organismal Injury and Abnormalities, Reproductive Sy	ovarian cancer	ovarian cancer	2.61E-11
Cellular Growth and Proliferation	colony formation	colony formation of tumor cell lines	2.63E-11
Cancer, Neurological Disease, Organismal Injury and Abnormalities	glioma cancer	glioma cancer	2.66E-11
Embryonic Development, Endocrine System Development and Function, Organ Developmer	development	development of pituitary gland	2.67E-11
Cell Death and Survival, Cellular Function and Maintenance	self-renewal	self-renewal of cells	2.72E-11
Cell Death and Survival	cell death	cell death of mononuclear leukocytes	2.74E-11
Cell Signaling	cell surface receptor linked signal transduction	cell surface receptor linked signal transduction	2.94E-11
Connective Tissue Disorders, Organismal Injury and Abnormalities, Skeletal and Muscular D	abnormality	abnormality of rib cage	2.96E-11
Cellular Development, Connective Tissue Development and Function, Tissue Development	differentiation	differentiation of bone cells	3.01E-11
Cancer, Organismal Injury and Abnormalities, Reproductive System Disease	female genital tract cancer	female genital tract cancer	3.15E-11
Cellular Movement	migration	migration of melanoma cell lines	3.17E-11
Cancer, Organismal Injury and Abnormalities	primary neoplasm	primary neoplasm	3.18E-11
Connective Tissue Disorders, Organismal Injury and Abnormalities, Skeletal and Muscular D	abnormality	abnormality of vertebrae	3.31E-11
Cancer, Organismal Injury and Abnormalities, Respiratory Disease	lung tumor	lung tumor	3.39E-11
Organ Morphology	morphology	morphology of skin	3.39E-11
Cardiovascular Disease, Organismal Injury and Abnormalities	abnormality	abnormality of heart ventricle	3.48E-11
Cellular Development, Embryonic Development, Hair and Skin Development and Function, (	differentiation	differentiation of dermal cells	3.52E-11

Cellular Development, Cellular Growth and Proliferation, Connective Tissue Development a	proliferation	proliferation of keratinocytes	3.52E-11
Cardiovascular Disease, Cardiovascular System Development and Function, Tissue Morphol	abnormal morphology	abnormal morphology of blood vessel	3.52E-11
Cancer, Organismal Injury and Abnormalities, Renal and Urological Disease	urinary tract cancer	urinary tract cancer	3.71E-11
DNA Replication, Recombination, and Repair	metabolism	metabolism of DNA	3.99E-11
Cancer, Hematological Disease, Immunological Disease, Organismal Injury and Abnormalitie	acute lymphocytic leukemia	acute lymphocytic leukemia	4.16E-11
Cancer, Organismal Injury and Abnormalities	head and neck neoplasia	head and neck neoplasia	4.19E-11
Lipid Metabolism, Molecular Transport, Small Molecule Biochemistry	quantity	quantity of steroid	4.54E-11

Upstream Regulator	Molecule Type	p-value of overlap
TGFB1	growth factor	9.42E-28
APP	other	1.42E-24
TP53	transcription regulator	6.01E-23
ESR1	ligand-dependent nuclear recept	2.91E-21
CTNNB1	transcription regulator	1.68E-19
TNF	cytokine	1.99E-18
PDGF BB	complex	4.25E-17
MYC	transcription regulator	1.19E-16
FGF2	growth factor	4.52E-15
HRAS	enzyme	4.78E-15
EGF	growth factor	5.34E-15
IGF1	growth factor	5.42E-15
AGT	growth factor	1.04E-14
SOX2	transcription regulator	2.72E-14
ERBB2	kinase	5.46E-14
KRAS	enzyme	6.73E-14
WNT3A	cytokine	3.56E-13
HTT	transcription regulator	1.4E-12
HIF1A	transcription regulator	6.43E-12
SHH	peptidase	6.79E-12
WT1	transcription regulator	1.17E-11
NRG1	growth factor	2.52E-11
CREB1	transcription regulator	3.6E-11
Histone h3	group	5.47E-11
EP300	transcription regulator	1.21E-10
MBD3	enzyme	1.8E-10
NFkB (complex)	complex	2.24E-10
IL3	cytokine	2.84E-10
STAT3	transcription regulator	3.64E-10
EPO	cytokine	4.79E-10
YY1	transcription regulator	7.37E-10
PSEN1	peptidase	8.93E-10
BDNF	growth factor	9.28E-10
IL1B	cytokine	1.4E-09
VEGFA	growth factor	1.53E-09
NOTCH1	transcription regulator	1.54E-09
FOXO3	transcription regulator	1.72E-09
IFNG	cytokine	2.24E-09
NGF	growth factor	2.26E-09
CNR1	g-protein coupled receptor	2.48E-09
LDL	complex	4.21E-09
MAPT	other	4.38E-09
Pkc(s)	group	4.73E-09
Ins1	other	5.22E-09
CD3	complex	7.05E-09
MTOR	kinase	8.37E-09
PITX2	transcription regulator	9.49E-09
EGFR	kinase	9.69E-09
DKK1	growth factor	9.91E-09
MYOD1	transcription regulator	1.04E-08
ID3	transcription regulator	1.12E-08
Mek	group	1.13E-08
NFKBIA	transcription regulator	1.32E-08
EGR2	transcription regulator	1.35E-08
IL2	cytokine	2.13E-08
CYR61	other	0.000000026
FSH	complex	2.78E-08
COMMD3-BMI1	transcription regulator	2.99E-08
NR3C1	ligand-dependent nuclear recept	3.09E-08
EZH2	transcription regulator	3.29E-08
NFE2L2	transcription regulator	3.45E-08
estrogen receptor	group	3.53E-08
HSPA5	enzyme	3.87E-08
HDAC1	transcription regulator	0.00000004
SMARCA4	transcription regulator	4.36E-08
RUNX1	transcription regulator	6.29E-08
CDK2AP1	other	6.61E-08
SMAD4	transcription regulator	7.64E-08

STAT5A	transcription regulator	7.66E-08
EPHB4	kinase	8.56E-08
MGEA5	enzyme	9.92E-08
mir-9	microrna	0.0000001
IL6	cytokine	0.000000101
Vegf	group	0.000000109
GLI1	transcription regulator	0.000000127
PTEN	phosphatase	0.000000138
KLF4	transcription regulator	0.000000157
HNF4A	transcription regulator	0.000000186
SMO	g-protein coupled receptor	0.000000234
MAPK9	kinase	0.000000258
RBPJ	transcription regulator	0.000000296
ERK1/2	group	0.000000322
VHL	transcription regulator	0.000000386
TNFSF11	cytokine	0.000000412
TWIST1	transcription regulator	0.000000446
Growth hormone	group	0.000000459
BMP4	growth factor	0.000000514
ERBB4	kinase	0.000000529
ETS1	transcription regulator	0.000000534
TCR	complex	0.000000599
CREBBP	transcription regulator	0.000000628
Nr1h	group	0.000000659
POU5F1	transcription regulator	0.000000702
Hedgehog	group	0.000000724
ADORA2A	g-protein coupled receptor	0.000000759
HDAC2	transcription regulator	0.000000759
CEBPA	transcription regulator	0.000000858
IKZF1	transcription regulator	0.000000894
Akt	group	0.000000988
BMP2	growth factor	0.00000104
TGFB2	growth factor	0.00000104
PPARG	ligand-dependent nuclear recept	0.00000109
AR	ligand-dependent nuclear recept	0.00000117
EBF1	transcription regulator	0.00000123
IL15	cytokine	0.00000128
Laminin	complex	0.00000148
MED1	transcription regulator	0.00000163
KDM5B	transcription regulator	0.00000169
PRKAG3	other	0.00000173
IL4	cytokine	0.00000201
PI3K (complex)	complex	0.00000201
PTH	other	0.00000217
ATF4	transcription regulator	0.0000025
INSR	kinase	0.00000257
PI3K (family)	group	0.00000264
FGF1	growth factor	0.00000289
TGFBR2	kinase	0.0000029
TGFB3	growth factor	0.00000316
HOXA10	transcription regulator	0.00000333
ID2	transcription regulator	0.00000335
ESR2	ligand-dependent nuclear recept	0.00000339
POU4F2	transcription regulator	0.00000342
OSM	cytokine	0.00000348
Lh	complex	0.0000038
EPAS1	transcription regulator	0.00000407
PAX3	transcription regulator	0.00000407
ANXA7	ion channel	0.00000415
RNA polymerase II	complex	0.00000437
TAF4	transcription regulator	0.00000472
DICER1	enzyme	0.00000472
INS	other	0.00000476
Histone h4	group	0.00000489
P38 MAPK	group	0.00000511
FOXO4	transcription regulator	0.00000518
BRD2	kinase	0.00000524
LTBP4	growth factor	0.00000564
MAPK8	kinase	0.00000565

Insulin	group	0.00000619
PRKCD	kinase	0.00000631
TCF4	transcription regulator	0.00000644
PIK3R1	kinase	0.00000678
Creb	group	0.00000687
NRG2	growth factor	0.00000736
TEAD4	transcription regulator	0.00000792
TP63	transcription regulator	0.00000815
AKT1	kinase	0.00000828
PRL	cytokine	0.00000882
EGR1	transcription regulator	0.00000907
MKNK1	kinase	0.00000932
Smad	complex	0.00000939
SPRY2	other	0.00000939
APOE	transporter	0.00000947
26s Proteasome	complex	0.0000095
CDKN2A	transcription regulator	0.00000977
PRKAA2	kinase	0.0000103
miR-17-5p (and other miRNAs w/seed AAAGUGC)	mature microrna	0.0000104
RNF2	transcription regulator	0.0000104
E2F1	transcription regulator	0.0000128
Tgf beta	group	0.0000128
WNT1	cytokine	0.0000135
HSPA9	other	0.0000136
PHF1	transcription regulator	0.0000142
MAPK14	kinase	0.0000169
CD28	transmembrane receptor	0.0000171
FGF7	growth factor	0.0000174
F2	peptidase	0.000018
MYCN	transcription regulator	0.0000182
CDK7	kinase	0.0000203
STAT5a/b	group	0.0000204
AHR	ligand-dependent nuclear recept	0.0000205
PRKCA	kinase	0.0000209
TCF3	transcription regulator	0.000022
CSF1	cytokine	0.0000221
EIF2AK3	kinase	0.0000226
TCF7L2	transcription regulator	0.0000227
SIN3A	transcription regulator	0.0000232
SMAD3	transcription regulator	0.0000244
IRS1	enzyme	0.0000266
BMP7	growth factor	0.0000266
SIRT1	transcription regulator	0.000028
ITGA6	transmembrane receptor	0.0000302
GFI1B	transcription regulator	0.0000302
SOX2-OCT4-NANOG	complex	0.0000327
Jnk	group	0.000033
SREBF1	transcription regulator	0.000033
SOD1	enzyme	0.000033
PAX6	transcription regulator	0.0000342
GATA1	transcription regulator	0.000036
USF2	transcription regulator	0.0000361
CBX2	transcription regulator	0.0000379
SREBF2	transcription regulator	0.0000382
POU4F1	transcription regulator	0.0000382
CDKN1A	kinase	0.0000389
CXCL12	cytokine	0.0000389
KITLG	growth factor	0.0000396
ATN1	transcription regulator	0.0000402
MITF	transcription regulator	0.0000441
PGR	ligand-dependent nuclear recept	0.0000479
WWOX	enzyme	0.0000486
BMP	group	0.0000487
Hdac	group	0.0000495
miR-16-5p (and other miRNAs w/seed AGCAGCA)	mature microrna	0.0000496
FGF8	growth factor	0.0000498
STAT4	transcription regulator	0.0000519
GLI2	transcription regulator	0.0000539
XBP1	transcription regulator	0.0000548

SPDEF	transcription regulator	0.000056
TLR4	transmembrane receptor	0.0000583
EIF4E	translation regulator	0.0000588
GATA4	transcription regulator	0.00006
SNCA	enzyme	0.00006
GNA15	enzyme	0.0000628
SP1	transcription regulator	0.0000629
CSF3	cytokine	0.0000638
mir-24	microrna	0.000064
PCGF2	transcription regulator	0.000064
STUB1	enzyme	0.000064
BNIP3L	other	0.0000643
NR3C2	ligand-dependent nuclear recept	0.0000657
THRB	ligand-dependent nuclear recept	0.0000667
EDN1	cytokine	0.0000718
NFATC2	transcription regulator	0.0000729
HGF	growth factor	0.0000748
Ras homolog	group	0.0000754
CYB5R4	enzyme	0.0000754
ELOVL5	enzyme	0.0000754
TBX1	transcription regulator	0.0000756
USF1	transcription regulator	0.0000784
HES1	transcription regulator	0.0000796
FOXO1	transcription regulator	0.0000807
CITED2	transcription regulator	0.0000823
INHBA	growth factor	0.0000825
CUL4B	other	0.0000847
REST	transcription regulator	0.0000882
REL	transcription regulator	0.0000905
SOX3	transcription regulator	0.0000931
FOXA2	transcription regulator	0.0000942
Immunoglobulin	complex	0.0000988
ADAM10	peptidase	0.000102
Sos	group	0.000106
PRDM5	transcription regulator	0.00011
FOS	transcription regulator	0.000111
CTGF	growth factor	0.000113
MECP2	transcription regulator	0.000115
ZNF217	transcription regulator	0.000116
ISL1	transcription regulator	0.00012
MAPK1	kinase	0.00012
ERG	transcription regulator	0.000126
BCR (complex)	complex	0.000129
SP3	transcription regulator	0.000129
mir-10	microrna	0.00013
MED12	transcription regulator	0.000135
SOX1	transcription regulator	0.000138
MYOC	other	0.000139
ERBB3	kinase	0.000139
miR-9-5p (and other miRNAs w/seed CUUUGGU)	mature microrna	0.000141
NKX3-1	transcription regulator	0.000142
TNC	other	0.000149
HMGA1	transcription regulator	0.000152
CNTF	cytokine	0.000152
GMNN	transcription regulator	0.000152
LYN	kinase	0.000152
miR-124-3p (and other miRNAs w/seed AAGGCAC)	mature microrna	0.000153
COL18A1	other	0.000162
MET	kinase	0.000162
BMI1	transcription regulator	0.000164
FGF4	growth factor	0.000166
mir-103	microrna	0.000166
ERK	group	0.000167
NUMB	other	0.000174
miR-199a-3p (and other miRNAs w/seed CAGUAGU	mature microrna	0.000174
BRCA1	transcription regulator	0.000174
RUNX3	transcription regulator	0.000175
ADCYAP1	other	0.000182
Wnt	group	0.000184

SMAD1	transcription regulator	0.000184
TP73	transcription regulator	0.000184
IGF2	growth factor	0.000187
BCL2	transporter	0.000195
KAT6A	enzyme	0.000196
Il3	cytokine	0.000205
NR4A1	ligand-dependent nuclear recept	0.000213
PIM1	kinase	0.000214
FAS	transmembrane receptor	0.000214
SNAI1	transcription regulator	0.000222
Cg	complex	0.000236
EED	transcription regulator	0.000237
NCOR1	transcription regulator	0.000242
FBXO32	enzyme	0.000242
PRNP	other	0.000244
SLC4A1	transporter	0.000245
BMP6	growth factor	0.000251
LEP	growth factor	0.000254
JUN	transcription regulator	0.000254
CCND1	transcription regulator	0.000254
CYP1A1	enzyme	0.000254
LRP1	transmembrane receptor	0.000255
miR-141-3p (and other miRNAs w/seed AACACUG)	mature microrna	0.00026
SEL1L	other	0.000265
KIF3A	enzyme	0.000285
Mup1 (includes others)	other	0.000285
TAF6	transcription regulator	0.000285
miR-199a-5p (and other miRNAs w/seed CCAGUGU	mature microrna	0.000288
mir-1	microrna	0.000288
Mapk	group	0.000291
GLI3	transcription regulator	0.000294
RETN	other	0.000294
NFYB	transcription regulator	0.000296
IFNB1	cytokine	0.000296
STAT6	transcription regulator	0.000297
COL4A3	other	0.000331
NTRK1	kinase	0.000331
E2F4	transcription regulator	0.000335
IL5	cytokine	0.000357
NANOG	transcription regulator	0.000358
LMNA	other	0.000364
SYVN1	transporter	0.000365
mir-133	microrna	0.000368
MAX	transcription regulator	0.000374
ITGB1	transmembrane receptor	0.000374
Pdgf (complex)	complex	0.000379
IKBKB	kinase	0.000383
mir-27	microrna	0.000383
NOG	growth factor	0.000383
CCNH	transcription regulator	0.000392
MNT	transcription regulator	0.000392
SPHK2	kinase	0.0004
TERT	enzyme	0.000406
ANGPT2	growth factor	0.000414
ABL1	kinase	0.000419
KLF6	transcription regulator	0.000429
RARB	ligand-dependent nuclear recept	0.000431
NFKB1	transcription regulator	0.000439
THRA	ligand-dependent nuclear recept	0.000451
CD40LG	cytokine	0.000452
GRP	growth factor	0.000454
PCYT1A	enzyme	0.000454
KDM2B	enzyme	0.000454
NFATC4	transcription regulator	0.000454
ALB	transporter	0.000456
TNFSF13B	cytokine	0.000465
LMNB1	other	0.000465
CD40	transmembrane receptor	0.000482
CFC1/CFC1B	other	0.000482

STIL	other	0.000482
YEATS4	transcription regulator	0.000482
UCHL1	peptidase	0.000482
miR-2682-5p (and other miRNAs w/seed AGGCAGI	mature microrna	0.000482
PYCR2	enzyme	0.000482
TOP2B	enzyme	0.000482
CTH	enzyme	0.000482
BUB3	other	0.000482
MXD3	transcription regulator	0.000482
N-cor	group	0.000494
PLSCR1	enzyme	0.000498
OBP2B	transporter	0.000498
Collagen type I	complex	0.000506
CLOCK	transcription regulator	0.000519
MAP4K4	kinase	0.000529
CDKN1B	kinase	0.000529
miR-27a-3p (and other miRNAs w/seed UCACAGU)	mature microrna	0.000529
AGTR1	g-protein coupled receptor	0.000529
Shc	group	0.000532
PPM1D	phosphatase	0.000532
RYBP	transcription regulator	0.000532
Igkv1-117	other	0.000532
VCAN	other	0.000535
ADIPOQ	other	0.000557
CDC73	other	0.000562
ERVW-1	other	0.000562
ZNF202	transcription regulator	0.000562
mir-19	microrna	0.000562
AMPK	complex	0.000594
HMOX1	enzyme	0.000618
Fgfr	group	0.000622
48s	complex	0.000622
mir-214	microrna	0.000622
NF2	other	0.000622
BDKRB2	g-protein coupled receptor	0.000622
PLAGL1	transcription regulator	0.000622
TBX2	transcription regulator	0.000628
let-7a-5p (and other miRNAs w/seed GAGGUAG)	mature microrna	0.000633
CSF2	cytokine	0.000656
ANGPT1	growth factor	0.000662
CDK9	kinase	0.000675
mir-15	microrna	0.000675
Rock	group	0.000686
PAX1	transcription regulator	0.000686
SRF	transcription regulator	0.00071
PRMT5	enzyme	0.000753
mir-30	microrna	0.000753
SLC16A2	transporter	0.000753
miR-30c-5p (and other miRNAs w/seed GUAAACA)	mature microrna	0.000782
FGFR2	kinase	0.000783
IKBKG	kinase	0.000783
MECOM	transcription regulator	0.000786
PCDH11Y	other	0.000786
mir-22	microrna	0.000809
miR-26a-5p (and other miRNAs w/seed UCAAGUA	mature microrna	0.000809
CTSB	peptidase	0.000809
RB1	transcription regulator	0.000811
TREM1	transmembrane receptor	0.000819
CD44	enzyme	0.000819
HNRNPA2B1	other	0.000833
KMT2A	transcription regulator	0.000844
SOX9	transcription regulator	0.000891
DNA-methyltransferase	group	0.000913
SGPL1	enzyme	0.000913
Collagen Alpha1	group	0.000913
XRCC5	enzyme	0.000913
LNX2	other	0.000913
PARP1	enzyme	0.000915
INSIG1	other	0.000915

IRF4	transcription regulator	0.000918
Alpha catenin	group	0.000928
CHD7	enzyme	0.00093
MXI1	transcription regulator	0.00093
PDGFRA	kinase	0.00093
ZBED6	other	0.000954
EGLN1	enzyme	0.00096
SERPINF1	other	0.00096
TCF12	transcription regulator	0.00096
TRAF3	enzyme	0.000963
GAST	other	0.000963
T3-TR-RXR	complex	0.00101
KDR	kinase	0.00101
KAT2B	transcription regulator	0.00101
STK11	kinase	0.00101
IGF1R	transmembrane receptor	0.00103
mir-29	microrna	0.00104
Ubiquitin	group	0.00106
mir-25	microrna	0.00106
JAG1	growth factor	0.00106
TFAP2A	transcription regulator	0.00107
GFI1	transcription regulator	0.00107
NOV	growth factor	0.00108
PKD2	ion channel	0.00109
DVL1	other	0.00109
LRP2	transporter	0.00109
USP8	peptidase	0.00109
CUL1	enzyme	0.00109
PPM1A	phosphatase	0.00109
DMTF1	transcription regulator	0.00109
GATA3	transcription regulator	0.0011
CD38	enzyme	0.00112
TFDP1	transcription regulator	0.0012
FLT3	kinase	0.0012
S100A8	other	0.0012
ELAVL1	other	0.00121
THPO	cytokine	0.00121
FMR1	translation regulator	0.00121
BCL6	transcription regulator	0.00122
miR-1-3p (and other miRNAs w/seed GGAAUGU)	mature microrna	0.00125
PRKCE	kinase	0.00126
RAF1	kinase	0.00128
KLF2	transcription regulator	0.00134
MAP2K4	kinase	0.00134
AGPAT2	enzyme	0.00134
FKBP4	enzyme	0.00134
caspase	group	0.00136
HOXB13	transcription regulator	0.00136
PBX1	transcription regulator	0.00136
NUPR1	transcription regulator	0.00137
RARA	ligand-dependent nuclear recept	0.00137
BRD4	kinase	0.00141
Ras	group	0.00141
CDX2	transcription regulator	0.00141
OTX2	transcription regulator	0.00141
ITK	kinase	0.00144
LTBP1	other	0.00144
GNL3	other	0.00144
IL10	cytokine	0.00145
AURKA	kinase	0.00146
CHRM1	g-protein coupled receptor	0.00146
PTBP1	enzyme	0.00146
JUP	other	0.00146
PDE3B	enzyme	0.00146
DBI	other	0.00146
MMP2	peptidase	0.00148
mir-145	microrna	0.0015
MTA1	transcription regulator	0.0015
CHUK	kinase	0.00153

WNT5A	cytokine	0.00161
SOCS3	phosphatase	0.00164
GDNF	growth factor	0.00164
miR-133a-3p (and other miRNAs w/seed UUGGUC	mature microrna	0.00171
MIR124	group	0.00173
FGFR3	kinase	0.00173
CCNK	kinase	0.00173
SKIL	transcription regulator	0.0018
Meg3	other	0.00182
SUFU	transcription regulator	0.00182
GRPR	g-protein coupled receptor	0.00182
miR-125b-2-3p (miRNAs w/seed CAAGUCA)	mature microrna	0.00182
GGA3	transporter	0.00182
RAPSN	other	0.00182
ATP2A1	transporter	0.00182
CHD4	enzyme	0.00183
CBX5	transcription regulator	0.00186
LYL1	transcription regulator	0.00188
S100B	other	0.00188
mir-144	microrna	0.00188
JARID2	transcription regulator	0.00188
SLC6A3	transporter	0.00188
NONO	other	0.00188
BAG1	other	0.0019
RRP1B	other	0.00191
CST5	other	0.00193
miR-34a-5p (and other miRNAs w/seed GGCAGUG	mature microrna	0.00201
PNPLA8	enzyme	0.00204
ACACA	enzyme	0.00204
SH3KBP1	other	0.00204
ACLY	enzyme	0.00204
SMARCA1	transcription regulator	0.00204
HBP1	transcription regulator	0.00204
EMX2	transcription regulator	0.00204
SERPINE2	other	0.00204
FBXO2	enzyme	0.00204
DHH	peptidase	0.00204
RORA	ligand-dependent nuclear recept	0.00206
TSH	complex	0.00213
MMP9	peptidase	0.00213
TFAP2C	transcription regulator	0.00213
DGCR8	enzyme	0.00216
APOA1	transporter	0.00216
MYOG	transcription regulator	0.00217
KNG1	other	0.00217
Gli	group	0.00223
KLF10	transcription regulator	0.00223
ID4	transcription regulator	0.00223
FOXA1	transcription regulator	0.0023
UCP1	transporter	0.00231
FGF10	growth factor	0.00236
RXRA	ligand-dependent nuclear recept	0.00244
NCOR-LXR-Oxysterol-RXR-9 cis RA	complex	0.00246
miR-24-3p (and other miRNAs w/seed GGCUCAG)	mature microrna	0.00246
E2F6	transcription regulator	0.00246
KIT	transmembrane receptor	0.00253
A2M	transporter	0.00257
MAPK8IP1	other	0.00257
DAG1	transmembrane receptor	0.00257
RAE1	other	0.00257
NAB2	transcription regulator	0.00257
S100A9	other	0.00262
SMARCB1	transcription regulator	0.00262
GDF2	growth factor	0.00266
Hsp27	group	0.0027
ATP7B	transporter	0.0027
ACVR1	kinase	0.0027
HDAC5	transcription regulator	0.00276
POLR2A	enzyme	0.00276

FANCC	other	0.00276
FOXP3	transcription regulator	0.00282
MEF2C	transcription regulator	0.00282
miR-29b-3p (and other miRNAs w/seed AGCACCA)	mature microrna	0.00284
IgG	complex	0.00284
LEPR	transmembrane receptor	0.00284
ADRB	group	0.00302
HOXA3	transcription regulator	0.00304
IRF8	transcription regulator	0.00304
mir-21	microrna	0.0031
LIF	cytokine	0.0032
mir-17	microrna	0.00321
YBX1	transcription regulator	0.00321
AGER	transmembrane receptor	0.00323
TGFBR1	kinase	0.00323
STOX1	other	0.00324
APBB1	transcription regulator	0.00324
LAMC1	other	0.00324
EP400	other	0.00325
SPHK1	kinase	0.00325
RUVBL1	transcription regulator	0.00325
SPI1	transcription regulator	0.00329
MTTP	transporter	0.00332
LIPE	enzyme	0.00333
LGALS3	other	0.00335
EHF	transcription regulator	0.00335
SP100	transcription regulator	0.00343
HFE	transmembrane receptor	0.00343
RACK1	enzyme	0.00343
AXIN1	other	0.00343
EPOR	transmembrane receptor	0.00343
voltage-gated calcium channel	complex	0.00345
HUWE1	transcription regulator	0.00345
IFT88	other	0.00345
TIP60	complex	0.00345
MIRLET7	group	0.00345
mir-154	microrna	0.00345
CCND2	other	0.00345
CBX8	other	0.00345
DDB1	other	0.00345
RECK	other	0.00345
FOXC2	transcription regulator	0.00357
SUZ12	enzyme	0.00363
E2f	group	0.00365
NEUROG3	transcription regulator	0.00366
MAP2K7	kinase	0.00372
CEBPB	transcription regulator	0.00374
SOX7	transcription regulator	0.00387
RCE1	peptidase	0.00387
GHR	transmembrane receptor	0.00387
TGFA	growth factor	0.00396
USP7	peptidase	0.00396
CSF1R	kinase	0.00396
ECSIT	transcription regulator	0.00396
DRD2	g-protein coupled receptor	0.00396
THBS4	other	0.00396
ARNT	transcription regulator	0.00401
HBEGF	growth factor	0.00405
DIO2	enzyme	0.00405
MEIS1	transcription regulator	0.00405
PPARGC1A	transcription regulator	0.0041
LDLR	transporter	0.00417
MAPK7	kinase	0.00418
BRAF	kinase	0.00418
EOMES	transcription regulator	0.00426
TPPP2	other	0.00427
Cpla2	group	0.00427
Stat1 dimer	complex	0.00427
FA2H	enzyme	0.00427

RASSF6	other	0.00427
CDK12	kinase	0.00427
BCAS2	other	0.00427
MTF2	transcription regulator	0.00427
ZBTB46	other	0.00427
TYMS	enzyme	0.00427
RNF34	enzyme	0.00427
miR-378a-3p (and other miRNAs w/seed CUGGACU	mature microrna	0.00427
PPP1R13B	phosphatase	0.00427
RIPK4	kinase	0.00427
WSB1	enzyme	0.00427
ELAVL4	other	0.00427
KCTD11	other	0.00427
BRCA2	transcription regulator	0.00427
NAA10	enzyme	0.00427
HIVEP2	transcription regulator	0.00427
GNL2	enzyme	0.00427
Collagen type V	complex	0.00427
HDAC4	transcription regulator	0.0044
JINK1/2	group	0.00448
CaMKII	complex	0.00448
JUN/JUNB/JUND	group	0.00448
ATG5	other	0.00448
IGF2BP1	translation regulator	0.00448
TRPC1	ion channel	0.00448
SIN3B	transcription regulator	0.00448
PKD1	ion channel	0.0045
SELENOS	other	0.00455
DPH5	enzyme	0.00455
ZBTB33	other	0.00455
ZNF148	transcription regulator	0.00455
SPTLC2	enzyme	0.00455
PNN	other	0.00455
MIR101	group	0.00455
HOXC9	transcription regulator	0.00455
DDR1	kinase	0.00455
RND3	enzyme	0.00455
DDX5	enzyme	0.00455
NDN	transcription regulator	0.00455
HOXC6	transcription regulator	0.00459
MEF2D	transcription regulator	0.00459
STAT1	transcription regulator	0.00461
ASCL1	transcription regulator	0.00464
TCF	group	0.00464
Notch	group	0.00467
CYP19A1	enzyme	0.00467
PLG	peptidase	0.00467
MSX2	transcription regulator	0.00489
SCD	enzyme	0.00492
DDIT3	transcription regulator	0.00492
mir-193	microrna	0.00493
MEP1B	peptidase	0.00493
PTCH1	transmembrane receptor	0.00493
MKL2	transcription regulator	0.00496
SMAD2	transcription regulator	0.00496
STAT5B	transcription regulator	0.00503
FN1	enzyme	0.00518
let-7	microrna	0.00539
TXK	kinase	0.00539
IGF2R	transmembrane receptor	0.00539
ALK	kinase	0.00539
RELN	peptidase	0.00539
JMJD1C	enzyme	0.00539
ARNTL	transcription regulator	0.00541
YAP1	transcription regulator	0.00553
RHOA	enzyme	0.00553
H2AFB3 (includes others)	other	0.00563
ELK1	transcription regulator	0.00563
miR-21-5p (and other miRNAs w/seed AGCUUAU)	mature microrna	0.00563

SMAD7	transcription regulator	0.00567
miR-125b-5p (and other miRNAs w/seed CCCUGA(	mature microrna	0.00571
RNF31	enzyme	0.00576
SKI	transcription regulator	0.00576
FASN	enzyme	0.00576
NR1I3	ligand-dependent nuclear recept	0.00579
NCSTN	peptidase	0.00586
IL6R	transmembrane receptor	0.00589
SPP1	cytokine	0.006
CHADL	other	0.00607
Rbp	group	0.00615
NSA2	other	0.00615
ZSCAN1	other	0.00615
RTN3	other	0.00615
ISL2	transcription regulator	0.00615
SEMA4A	other	0.00615
EPN3	other	0.00615
GSTK1	enzyme	0.00615
NAT8	transcription regulator	0.00615
NCEH1	enzyme	0.00615
VAX2	transcription regulator	0.00615
Gm-Csf Receptor	complex	0.00615
Importin alpha	group	0.00615
BACE1-AS	other	0.00615
AP3D1	transporter	0.00615
APBA1	transporter	0.00615
PTPA	phosphatase	0.00615
miR-188-3p (miRNAs w/seed UCCCACA)	mature microrna	0.00615
mir-134	microrna	0.00615
CENPX	other	0.00615
RPS14	translation regulator	0.00615
PSG1	other	0.00615
TOP3A	enzyme	0.00615
MAGEA4	other	0.00615
SYNJ2BP	other	0.00615
TSPYL2	other	0.00615
ITM2B	other	0.00615
EHD3	other	0.00615
CCT5	other	0.00615
DST	other	0.00615
WASHC1	other	0.00615
ATP9A	transporter	0.00615
MXD4	transcription regulator	0.00615
PTPRT	phosphatase	0.00615
GAS1	other	0.00615
NPAS3	other	0.00615
MTHFR	enzyme	0.00615
PCBP4	other	0.00615
PDIA6	enzyme	0.00615
DDX20	transcription regulator	0.00615
EVC	other	0.00615
Usp17la (includes others)	peptidase	0.00615
NAT8B	enzyme	0.00615
S100A11	other	0.00615
DUOX2	enzyme	0.00615
mir-203	microrna	0.0062
ACACB	enzyme	0.0062
OSBPL8	transporter	0.0062
ITPR1	ion channel	0.0062
POMC	other	0.00631
ITGB2	transmembrane receptor	0.00634
ITGA5	transmembrane receptor	0.00634
mir-34	microrna	0.00634
mir-181	microrna	0.00634
BACH2	transcription regulator	0.00634
TSC2	other	0.00635
Gm-csf	group	0.00649
FSHR	g-protein coupled receptor	0.00649
INHA	growth factor	0.00699

CTR9	other	0.00729
PTF1A	transcription regulator	0.00729
TCF7L1	transcription regulator	0.00739
UPF2	other	0.00739
SOX10	transcription regulator	0.00739
PRKACA	kinase	0.00739
ERN1	kinase	0.00744
JAK2	kinase	0.00744
PTGES	enzyme	0.00745
EIF2AK2	kinase	0.00758
Hsp90	group	0.00784
NCOA3	transcription regulator	0.00784
PTHLH	other	0.00784
MKL1	transcription regulator	0.00786
Adaptor protein 2	complex	0.00795
mir-148	microrna	0.00795
miR-217-5p (and other miRNAs w/seed ACUGCAU	mature microrna	0.00795
mir-137	microrna	0.00795
FOXD1	transcription regulator	0.00795
VEGFC	growth factor	0.00795
ITGA3	other	0.00795
MZF1	transcription regulator	0.00795
GPR37	g-protein coupled receptor	0.00795
BHLHE41	transcription regulator	0.00795
ACO1	enzyme	0.00795
PADI4	enzyme	0.00805
HOXD8	transcription regulator	0.00805
RASSF3	other	0.00805
AQP11	transporter	0.00805
TCF21	transcription regulator	0.00805
BANP	other	0.00805
RBBP4	enzyme	0.00805
LRP8	transmembrane receptor	0.00805
KRT19	other	0.00805
BCL2L12	other	0.00805
miR-139-5p (miRNAs w/seed CUACAGU)	mature microrna	0.00805
ATP2B2	transporter	0.00805
SRSF3	other	0.00805
SSPN	other	0.00805
KSR1	kinase	0.00805
PEBP4	other	0.00805
SLIT2	other	0.00805
USP11	peptidase	0.00805
RPL11	other	0.00805
DNAJA3	other	0.00805
SIX3	transcription regulator	0.00805
PAX4	transcription regulator	0.00805
HOXD9	transcription regulator	0.00805
G6PC	phosphatase	0.00805
CDH13	other	0.00805
SULT1E1	enzyme	0.00805
YY1AP1	other	0.00805
RBFOX2	transcription regulator	0.00805
MYOCD	transcription regulator	0.00819
LGR4	transmembrane receptor	0.00822
Endothelin	group	0.00822
p85 (pik3r)	group	0.00822
BMPER	other	0.00822
ATP2A2	transporter	0.00822
ARG1	enzyme	0.00822
NCAM1	other	0.00822
FABP5	transporter	0.00822
CDK8	kinase	0.00822
MYCBP2	enzyme	0.00822
CARM1	transcription regulator	0.00824
DACH1	transcription regulator	0.00824
KIAA1524	other	0.00825
GHRL	growth factor	0.00825
FGFR1	kinase	0.00838

SOD2	enzyme	0.00843
mir-155	microrna	0.00843
IL1A	cytokine	0.0086
PML	transcription regulator	0.00877
TCOF1	transporter	0.00878
ACOX1	enzyme	0.00885
MAP2K1	kinase	0.00888
APOB	transporter	0.00891
WNT4	cytokine	0.00891
VTN	other	0.00891
Stat3-Stat3	complex	0.00908
IHH	enzyme	0.00908
CX3CL1	cytokine	0.00908
SIX5	transcription regulator	0.00908
NRAS	enzyme	0.00924
PPARA	ligand-dependent nuclear recept	0.0095
TFAP4	transcription regulator	0.00967
FLI1	transcription regulator	0.0097
CYP1B1	enzyme	0.0097
mir-182	microrna	0.00987
AVP	other	0.00987
EBI3	cytokine	0.00987
APC	enzyme	0.01
PDX1	transcription regulator	0.0101
CAV1	transmembrane receptor	0.0103
ETS2	transcription regulator	0.0105
MYD88	other	0.0105
SFRP1	transmembrane receptor	0.0106
PRKCZ	kinase	0.0106
F7	peptidase	0.0106
ZBTB7B	transcription regulator	0.0106
TIMP1	cytokine	0.0106
NRXN1	transporter	0.0106
ZBTB17	transcription regulator	0.0106
TMSB4	group	0.0107
RNF20	enzyme	0.0107
hemoglobin	complex	0.0107
COPS5	transcription regulator	0.0107
FMO3	enzyme	0.0107
ADRB1	g-protein coupled receptor	0.0107
Rxr	group	0.0111
IL27	cytokine	0.0111
NFYA	transcription regulator	0.0111
MTPN	transcription regulator	0.0111
HEXIM1	transcription regulator	0.0112
SAMSN1	other	0.0112
VGLL3	other	0.0112
PLD1	enzyme	0.0112
miR-375-3p (and other miRNAs w/seed UUGUUCG	mature microrna	0.0112
MAPK10	kinase	0.0112
EDNRB	g-protein coupled receptor	0.0112
BET	group	0.0112
TNFSF13	cytokine	0.0113
SERPINA1	other	0.0113
IRS2	enzyme	0.0113
NRF1	transcription regulator	0.0113
PIK3CA	kinase	0.0113
FOXM1	transcription regulator	0.0115
FOXL2	transcription regulator	0.0117
SFTPA1	transporter	0.0121
SLC13A1	transporter	0.0123
CFTR	ion channel	0.0123
PDGFB	growth factor	0.0124
CXCR4	g-protein coupled receptor	0.0124
RET	kinase	0.0129
KDM5A	transcription regulator	0.0129
IL17A	cytokine	0.013
Raf	group	0.0131
LONP1	peptidase	0.0131

ZNF100	other	0.0133
ZNF85	transcription regulator	0.0133
ZNF652	other	0.0133
ZNF254	other	0.0133
RASSF8	other	0.0133
ABCA7	transporter	0.0133
ZNF431	other	0.0133
ARID5A	transcription regulator	0.0133
DLG1	kinase	0.0133
Collagen type III	complex	0.0133
BAG6	enzyme	0.0133
HTR2A	g-protein coupled receptor	0.0133
AGR2	other	0.0133
CTNNBIP1	other	0.0133
RAX	transcription regulator	0.0133
GNE	kinase	0.0133
ENO1	enzyme	0.0133
ZNF665	other	0.0133
ZNF528	other	0.0133
MCM7	enzyme	0.0133
PROK1	growth factor	0.0133
NACC1	transcription regulator	0.0133
ZNF43	other	0.0133
ZNF429	other	0.0133
AFF1	transcription regulator	0.0133
SH2B1	other	0.0133
ZNF708	other	0.0133
GNA12	enzyme	0.0134
MPZ	other	0.0136
PPP1R13L	transcription regulator	0.0136
ING1	transcription regulator	0.0136
LHX2	transcription regulator	0.0136
CD9	other	0.0136
ARRB1	other	0.0136
TAF4B	transcription regulator	0.0136
SMAD5	transcription regulator	0.0136
Tcf7	transcription regulator	0.0136
NR1H3	ligand-dependent nuclear recept	0.0144
Ap1	complex	0.0145
KDM1A	enzyme	0.0146
TBP	transcription regulator	0.0146
PELP1	other	0.0147
miR-146a-5p (and other miRNAs w/seed GAGAACU	mature microrna	0.0149
DMP1	other	0.0151
GSX2	transcription regulator	0.0152
mir-26	microrna	0.0152
mir-218	microrna	0.0152
ELK4	transcription regulator	0.0152
FABP2	transporter	0.0152
TIMP3	other	0.0152
LEF1	transcription regulator	0.0157
ATF6	transcription regulator	0.0157
CTCF	transcription regulator	0.0162
ITGB3	transmembrane receptor	0.0162
GH1	growth factor	0.0163
ACTL6A	other	0.0163
NOX4	enzyme	0.0163
DMRT1	transcription regulator	0.0164
PTGER4	g-protein coupled receptor	0.0164
RORC	ligand-dependent nuclear recept	0.0166
ZBTB20	transcription regulator	0.0166
HOXA9	transcription regulator	0.0168
JUNB	transcription regulator	0.0169
ZMPSTE24	peptidase	0.0169
miR-31-5p (and other miRNAs w/seed GGCAAGA)	mature microrna	0.0169
miR-23a-3p (and other miRNAs w/seed UCACAUU)	mature microrna	0.0169
MLX	transcription regulator	0.0169
SMARCD3	transcription regulator	0.0169
MAP2K2	kinase	0.0169

IDO1	enzyme	0.0169
HOXA5	transcription regulator	0.0169
DUSP5	phosphatase	0.0169
CYP2J2	enzyme	0.0169
NR6A1	ligand-dependent nuclear recept	0.0169
GNAS	enzyme	0.0173
NME1	kinase	0.0173
IDH1	enzyme	0.0173
PTCHD4	other	0.0175
Smad1/5/8	group	0.0175
Beta Secretase	group	0.0175
TFIIH	complex	0.0175
ZNF219	transcription regulator	0.0175
CADPS	other	0.0175
PRMT7	enzyme	0.0175
RFFL	enzyme	0.0175
TTC5	other	0.0175
ELOVL2	enzyme	0.0175
BIRC6	enzyme	0.0175
EDEM1	enzyme	0.0175
PYHIN1	other	0.0175
MTCH2	other	0.0175
HDGF	growth factor	0.0175
CTBP1-AS2	other	0.0175
OSR2	transcription regulator	0.0175
EXOC3L1	other	0.0175
Eif2	complex	0.0175
CACNA1D	ion channel	0.0175
GTF2F2	transcription regulator	0.0175
PSMC3	transcription regulator	0.0175
ELL	transcription regulator	0.0175
PAG1	other	0.0175
RGS6	enzyme	0.0175
ANXA5	transporter	0.0175
SPTBN1	other	0.0175
MCRS1	other	0.0175
U2AF2	other	0.0175
BPTF	transcription regulator	0.0175
HAO1	enzyme	0.0175
miR-330-5p (and other miRNAs w/seed CUCUGGG	mature microrna	0.0175
DMBT1	transmembrane receptor	0.0175
SDHA	enzyme	0.0175
STIP1	other	0.0175
HSPA4	other	0.0175
AHNAK	other	0.0175
TCF20	transcription regulator	0.0175
PPP1R1A	phosphatase	0.0175
H3F3A/H3F3B	other	0.0175
CACNA2D1	ion channel	0.0175
DROSHA	enzyme	0.0175
FKBP1B	enzyme	0.0175
TNKS	enzyme	0.0175
SPAG9	other	0.0175
ANK1	other	0.0175
BLM	enzyme	0.0175
RPS7	other	0.0175
MAGI2	kinase	0.0175
CIAPIN1	other	0.0175
INHBC	growth factor	0.0175
ITGB8	other	0.0175
PLK3	kinase	0.0175
HSPH1	other	0.0175
TCP1	other	0.0175
CDC6	other	0.0175
Cacnb1	ion channel	0.0175
SRSF5	other	0.0175
RPL5	other	0.0175
BCKDHA	enzyme	0.0175
UIMC1	other	0.0175

Gsk3	group	0.018
ZBTB16	transcription regulator	0.018
PRKAA1	kinase	0.019
SRC	kinase	0.019
CA4	enzyme	0.0194
ASXL2	other	0.0194
PRKAR1A	kinase	0.0194
SRSF2	transcription regulator	0.0194
mir-122	microrna	0.0195
Calcineurin protein(s)	complex	0.0196
ABCA1	transporter	0.0197
IL7	cytokine	0.0199
Srebp	group	0.02
Ptprd	phosphatase	0.02
ALDH1A1	enzyme	0.02
mir-451	microrna	0.02
PRAME	other	0.02
PTPRE	phosphatase	0.02
SAT1	enzyme	0.02
CSF3R	transmembrane receptor	0.02
SPRY1	other	0.02
GTF2I	transcription regulator	0.02
SDCBP	enzyme	0.02
PDE5A	enzyme	0.02
SOCS2	other	0.02
Cyclin A	group	0.02
TCF/LEF	group	0.02
ZNF382	transcription regulator	0.02
Presenilin	group	0.02
VCL	enzyme	0.02
MDM4	enzyme	0.02
MTBP	other	0.02
PLA2G6	enzyme	0.02
TIA1	other	0.02
SDC2	other	0.02
WNT7B	other	0.02
PSIP1	transcription regulator	0.02
SOAT1	enzyme	0.02
ATXN3	peptidase	0.02
PAX9	transcription regulator	0.02
PIWIL2	other	0.02
PLA2G5	enzyme	0.02
TNRC6A	other	0.02
ZNF91	transcription regulator	0.02
NDP	growth factor	0.02
CAMKK2	kinase	0.02
ASXL1	transcription regulator	0.0201
PARK2	enzyme	0.0201
IL1	group	0.0203
DCAF1	kinase	0.0208
FUT8	enzyme	0.0208
PRKDC	kinase	0.0208
SRA1	transcription regulator	0.0208
DLL1	enzyme	0.0208
MAT1A	enzyme	0.0208
NCL	other	0.0208
mir-124	microrna	0.0208
FOXC1	transcription regulator	0.0208
MSX1	transcription regulator	0.0208
IKZF3	transcription regulator	0.0208
PDGFC	growth factor	0.0208
SMARCC1	transcription regulator	0.0208
SCAP	other	0.0211
Nfat (family)	group	0.0212
NKX2-1	transcription regulator	0.0213
Igm	complex	0.0217
RELA	transcription regulator	0.0219
SASH1	other	0.0219
RBL2	other	0.0223

E2F3	transcription regulator	0.0228
LRP6	transmembrane receptor	0.0229
FBXW7	transcription regulator	0.0229
SND1	enzyme	0.0229
NPPA	other	0.0229
NR1H2	ligand-dependent nuclear recept	0.0231
EGR3	transcription regulator	0.0232
GNB2	enzyme	0.0232
KLF5	transcription regulator	0.0236
GCG	other	0.0241
Pkg	group	0.0252
NADPH oxidase	complex	0.0252
IPMK	kinase	0.0252
PRKCI	kinase	0.0252
miR-203a-3p (and other miRNAs w/seed UGAAAU	mature microrna	0.0252
EIF4G1	translation regulator	0.0252
UBE3A	enzyme	0.0252
FABP4	transporter	0.0252
BTG2	transcription regulator	0.0252
PBX3	transcription regulator	0.0252
HSF1	transcription regulator	0.0253
IGFBP2	other	0.0253
CSF2RB	transmembrane receptor	0.0256
IGFBP7	transporter	0.0256
BCOR	transcription regulator	0.0256
ROCK1	kinase	0.0256
SMC3	other	0.0256
INSIG2	other	0.0256
GADD45B	other	0.0256
MAZ	transcription regulator	0.0256
S1PR2	g-protein coupled receptor	0.0256
HOXB4	transcription regulator	0.0256
EDNRA	transmembrane receptor	0.0256
CBX3	transcription regulator	0.0256
SPINT1	other	0.0256
IRF1	transcription regulator	0.0257
NFATC1	transcription regulator	0.0258
CDK4	kinase	0.0258
F2RL1	g-protein coupled receptor	0.0263
CASZ1	enzyme	0.0265
IL24	cytokine	0.0265
SUMO3	other	0.0265
NEDD9	other	0.0265
PRKCQ	kinase	0.0268
CLU	other	0.0268
MLXIPL	transcription regulator	0.0268
Interferon alpha	group	0.0273
TAC1	other	0.0281
MSGN1	transcription regulator	0.0283
Pde	group	0.0283
Ck2	complex	0.0283
APH1A	peptidase	0.0283
TENM1	peptidase	0.0283
AGRN	other	0.0283
DUSP16	phosphatase	0.0283
PDGF (family)	group	0.0283
AGRP	other	0.0283
mir-378	microrna	0.0283
PLCB1	enzyme	0.0283
RB1CC1	other	0.0283
AIMP2	other	0.0283
CDK6	kinase	0.0283
EYA1	phosphatase	0.0283
UTS2R	g-protein coupled receptor	0.0283
CRTC1	transcription regulator	0.0283
PNPT1	enzyme	0.0283
IREB2	translation regulator	0.0283
DBH	enzyme	0.0283
SLC30A3	transporter	0.0283

GRB2	kinase	0.0284
ADRA1B	g-protein coupled receptor	0.0284
PTGS2	enzyme	0.0289
SIRT6	enzyme	0.0293
PRKCB	kinase	0.0293
PLN	transporter	0.0293
NEUROG1	transcription regulator	0.0293
E2F2	transcription regulator	0.0293
HDL	complex	0.0302
MEN1	transcription regulator	0.0302
GNB1	enzyme	0.0302
PDGF-DD	complex	0.0302
NR2E1	ligand-dependent nuclear recept	0.0302
FAM3B	cytokine	0.0302
FGF9	growth factor	0.0302
HDAC3	transcription regulator	0.0306
MIF	cytokine	0.0308
CDK2	kinase	0.0311
FLT1	kinase	0.0315
ATG7	enzyme	0.0319
GATA6	transcription regulator	0.0319
LIN28B	other	0.032
CGB3 (includes others)	other	0.032
CTBP2	transcription regulator	0.032
miR-205-5p (and other miRNAs w/seed CCUUCAU)	mature microrna	0.032
MAFG	transcription regulator	0.032
CHEK2	kinase	0.032
DIRAS3	enzyme	0.032
Rhox5	transcription regulator	0.032
SMURF2	enzyme	0.032
DSP	other	0.032
SMAD6	transcription regulator	0.032
JUND	transcription regulator	0.0322
GNRH1	other	0.0324
Pln	other	0.0324
CD24	other	0.0325
GSK3B	kinase	0.0325
TR-RXR	complex	0.0332
PROM1	other	0.0332
ATF6B	transcription regulator	0.0332
USE1	other	0.0332
NOX5	ion channel	0.0332
HCAR1	g-protein coupled receptor	0.0332
LEO1	other	0.0332
SP6	transcription regulator	0.0332
SLCO1C1	transporter	0.0332
NLN	peptidase	0.0332
ARID2	transcription regulator	0.0332
ASPN	other	0.0332
USP22	peptidase	0.0332
MUCL1	other	0.0332
RAB23	enzyme	0.0332
DACT3	other	0.0332
K Channel	complex	0.0332
CDCA2	other	0.0332
P-TEFb	complex	0.0332
NEAT1	other	0.0332
UNG	enzyme	0.0332
TBR1	transcription regulator	0.0332
HDAC10	transcription regulator	0.0332
MBNL1	other	0.0332
ZNF281	transcription regulator	0.0332
RPS11	other	0.0332
CDC25B	phosphatase	0.0332
CELA2A	peptidase	0.0332
GGA1	transporter	0.0332
CSH1/CSH2	other	0.0332
Nppb	other	0.0332
CACNB4	ion channel	0.0332

STRA6	other	0.0332
miR-515-5p (and other miRNAs w/seed UCUCCAA)	mature microrna	0.0332
miR-516a-3p (and other miRNAs w/seed GCUUCCU	mature microrna	0.0332
miR-127-3p (miRNAs w/seed CGGAUCC)	mature microrna	0.0332
miR-324-5p (miRNAs w/seed GCAUCCC)	mature microrna	0.0332
BOK	other	0.0332
MPP5	kinase	0.0332
NUCB2	other	0.0332
CALCR	g-protein coupled receptor	0.0332
ANKH	transporter	0.0332
LIMK1	kinase	0.0332
PER3	other	0.0332
DLL3	other	0.0332
PIP5K1C	kinase	0.0332
CYP11B2	enzyme	0.0332
SIGMAR1	transmembrane receptor	0.0332
CHRM4	g-protein coupled receptor	0.0332
VLDLR	transporter	0.0332
KLK6	peptidase	0.0332
UBE2D1	enzyme	0.0332
RGD1560225	other	0.0332
APLP2	other	0.0332
PTCH2	transmembrane receptor	0.0332
PTP4A2	phosphatase	0.0332
RPS5	other	0.0332
DYNC2LI1	other	0.0332
SLC37A4	transporter	0.0332
LETMD1	other	0.0332
VSNL1	other	0.0332
RPS18	other	0.0332
PLAC8	other	0.0332
BARD1	transcription regulator	0.0332
CDON	other	0.0332
MYH9	enzyme	0.0332
TWSG1	other	0.0332
ZKSCAN3	transcription regulator	0.0332
MAD1L1	other	0.0332
ARNT2	transcription regulator	0.0333
KLF3	transcription regulator	0.0338
JAK3	kinase	0.0342
VIP	other	0.0349
MAP2K1/2	group	0.0353
HIPK2	kinase	0.0353
calpain	complex	0.0358
NAMPT	cytokine	0.0358
SIX1	transcription regulator	0.0358
DRAP1	transcription regulator	0.0358
NFIL3	transcription regulator	0.0358
NTRK2	kinase	0.0358
NPM1	transcription regulator	0.0358
GJA1	transporter	0.0358
BSG	transporter	0.0358
ACVRL1	kinase	0.0358
HIST1H1T	other	0.0359
Hist1h1a	other	0.0359
DPY30	enzyme	0.0381
CMKLR1	g-protein coupled receptor	0.0381
EIF4G2	translation regulator	0.0381
MXD1	transcription regulator	0.0381
MYF5	transcription regulator	0.0381
TIMP2	other	0.0381
RLIM	enzyme	0.0381
miR-223-3p (miRNAs w/seed GUCAGUU)	mature microrna	0.0381
RAMP1	transporter	0.0381
IL9R	transmembrane receptor	0.0381
ADIPOR2	transmembrane receptor	0.0381
MEOX1	transcription regulator	0.0381
PIM3	kinase	0.0381
GBX2	transcription regulator	0.0381

COPS8	other	0.0381
MAPKAPK3	kinase	0.0381
AEBP1	peptidase	0.0381
CAMK2A	kinase	0.0381
STK40	kinase	0.0386
PPP2CA	phosphatase	0.0386
HDAC6	transcription regulator	0.0386
ATF1	transcription regulator	0.0386
TXN	enzyme	0.0386
NR4A2	ligand-dependent nuclear recept	0.0386
Smad2/3-Smad4	complex	0.0394
Hmgb1	transcription regulator	0.0394
TF	transporter	0.0394
PHLPP1	enzyme	0.0394
SOX6	transcription regulator	0.0394
THY1	other	0.0394
TMBIM6	other	0.0394
PLD2	enzyme	0.0394
C5	other	0.0394
TRIM24	transcription regulator	0.0396
TICAM1	other	0.0399
MYB	transcription regulator	0.0403
Rar	group	0.041
RAB1B	other	0.041
HLX	transcription regulator	0.041
CAMK4	kinase	0.041
PTTG1	transcription regulator	0.041
PPARD	ligand-dependent nuclear recept	0.0415
NFIC	transcription regulator	0.0419
DOCK8	other	0.0421
C5	cytokine	0.0429
TAZ	enzyme	0.0433
TFRC	transporter	0.0433
EHMT2	transcription regulator	0.0433
POR	enzyme	0.0437
TSLP	cytokine	0.0466
IL27RA	transmembrane receptor	0.0466
Pro-inflammatory Cytokine	group	0.0474
SIRT2	transcription regulator	0.0476
SLC2A4	transporter	0.0476
miR-218-5p (and other miRNAs w/seed UGUGCUU	mature microrna	0.0476
CD22	transmembrane receptor	0.0476
MGAT5	enzyme	0.0476
NFASC	other	0.0476
ACVR1C	kinase	0.0476
RPS6KA5	kinase	0.0476
FDFT1	enzyme	0.0476
NPC2	other	0.0476
CREB3L1	transcription regulator	0.0476
HES3	other	0.0483
CEBPE	transcription regulator	0.0484
RTN4	other	0.0484
CDX1	transcription regulator	0.0484
DCN	other	0.0484
AHI1	other	0.0487
DLL4	other	0.0487
GAS6	growth factor	0.0487
TRIB3	kinase	0.0487
UBE2I	enzyme	0.0487
mGluR	group	0.0494
Gcn5l	group	0.0494
PIAS2	transcription regulator	0.0494
CHRM3	g-protein coupled receptor	0.0494
miR-101-3p (and other miRNAs w/seed ACAGUAC)	mature microrna	0.0494
miR-135a-5p (and other miRNAs w/seed AUGGCUU	mature microrna	0.0494
DRD1	g-protein coupled receptor	0.0494
RUVBL2	transcription regulator	0.0494
IGFBP1	other	0.0494
LIMA1	other	0.0494

CDKN2C	transcription regulator	0.0494
TRRAP	transcription regulator	0.0494
DDX17	enzyme	0.0494
BRD7	transcription regulator	0.0494
PARP2	enzyme	0.0494
CHRNA3	transmembrane receptor	0.0494
RFX2	transcription regulator	0.0494
HIST2H3C	other	0.0494
HSPA1A/HSPA1B	enzyme	0.0494
S100A7	other	0.0494

Overlap Results

Collection(s):	**CP**	**(Cannonical Pathways)**
# overlaps shown:	100	
# genesets in collections:	1329	
# genes in comparison (n):	1701	
# genes in universe (N):	45956	

**Gene Set Name**	**# Genes in Gene Set (K)**	**Description**	**# Genes in Overlap (k)**	**k/K**	**p-value**	**FDR q-value**
KEGG_PATHWAYS_IN_CANCER	328	Pathways in cancer	79	0.2409	4.29E-41	5.70E-38
REACTOME_DEVELOPMENTAL_BIOLOGY	396	Genes involved in Developmental Biology	79	0.1995	7.33E-35	4.87E-32
REACTOME_IMMUNE_SYSTEM	933	Genes involved in Immune System	120	0.1286	1.27E-32	5.62E-30
REACTOME_HEMOSTASIS	466	Genes involved in Hemostasis	76	0.1631	1.41E-27	4.68E-25
REACTOME_AXON_GUIDANCE	251	Genes involved in Axon guidance	55	0.2191	8.63E-27	2.29E-24
REACTOME_ADAPTIVE_IMMUNE_SYSTEM	539	Genes involved in Adaptive Immune System	79	0.1466	1.73E-25	3.83E-23
KEGG_MAPK_SIGNALING_PATHWAY	267	MAPK signaling pathway	54	0.2022	1.52E-24	2.89E-22
KEGG_WNT_SIGNALING_PATHWAY	151	Wnt signaling pathway	38	0.2517	3.39E-21	5.63E-19
KEGG_BASAL_CELL_CARCINOMA	55	Basal cell carcinoma	23	0.4182	6.11E-19	8.93E-17
REACTOME_NEURONAL_SYSTEM	279	Genes involved in Neuronal System	48	0.172	6.72E-19	8.93E-17
WNT_SIGNALING	89	Genes related to Wnt-mediated signal transduction	28	0.3146	7.96E-19	9.62E-17
KEGG_FOCAL_ADHESION	201	Focal adhesion	40	0.199	2.74E-18	3.03E-16
KEGG_AXON_GUIDANCE	129	Axon guidance	32	0.2481	7.05E-18	7.20E-16
NABA_MATRISOME	1028	Ensemble of genes encoding extracellular matrix and extracellular matrix-associated proteins	98	0.0953	1.98E-17	1.88E-15
PID_PDGFRB_PATHWAY	129	PDGFR-beta signaling pathway	30	0.2326	5.09E-16	4.51E-14
REACTOME_METABOLISM_OF_LIPIDS_AND_LIPOPROTEINS	478	Genes involved in Metabolism of lipids and lipoproteins	59	0.1234	7.94E-16	6.60E-14
KEGG_HEDGEHOG_SIGNALING_PATHWAY	56	Hedgehog signaling pathway	20	0.3571	4.59E-15	3.59E-13
KEGG_REGULATION_OF_ACTIN_CYTOSKELETON	216	Regulation of actin cytoskeleton	36	0.1667	4.24E-14	3.13E-12
KEGG_CHRONIC_MYELOID_LEUKEMIA	73	Chronic myeloid leukemia	21	0.2877	1.31E-13	9.17E-12
REACTOME_CLASS_I_MHC_MEDIATED_ANTIGEN_PROCESSING_PRESENTATION	251	Genes involved in Class I MHC mediated antigen processing & presentation	38	0.1514	1.95E-13	1.29E-11
REACTOME_SIGNALLING_BY_NGF	217	Genes involved in Signalling by NGF	35	0.1613	2.58E-13	1.64E-11
KEGG_MELANOGENESIS	102	Melanogenesis	24	0.2353	3.18E-13	1.92E-11
REACTOME_PLATELET_ACTIVATION_SIGNALING_AND_AGGREGATION	208	Genes involved in Platelet activation, signaling and aggregation	34	0.1635	3.80E-13	2.20E-11
REACTOME_SIGNALING_BY_EGFR_IN_CANCER	109	Genes involved in Signaling by EGFR in Cancer	24	0.2202	1.50E-12	8.28E-11
REACTOME_CYTOKINE_SIGNALING_IN_IMMUNE_SYSTEM	270	Genes involved in Cytokine Signaling in Immune system	38	0.1407	1.96E-12	1.04E-10
REACTOME_TRANSMISSION_ACROSS_CHEMICAL_SYNAPSES	186	Genes involved in Transmission across Chemical Synapses	31	0.1667	2.42E-12	1.21E-10
REACTOME_CELL_CYCLE_MITOTIC	325	Genes involved in Cell Cycle, Mitotic	42	0.1292	2.46E-12	1.21E-10
NABA_MATRISOME_ASSOCIATED	753	Ensemble of genes encoding ECM-associated proteins including ECM-affilaited proteins, ECM regulators and secreted factors	70	0.093	2.61E-12	1.24E-10
REACTOME_SIGNALING_BY_PDGF	122	Genes involved in Signaling by PDGF	25	0.2049	2.84E-12	1.30E-10
PID_ERBB1_DOWNSTREAM_PATHWAY	105	ErbB1 downstream signaling	23	0.219	4.87E-12	2.16E-10
REACTOME_SIGNALING_BY_SCF_KIT	78	Genes involved in Signaling by SCF-KIT	20	0.2564	5.31E-12	2.28E-10
REACTOME_SIGNALING_BY_FGFR_IN_DISEASE	127	Genes involved in Signaling by FGFR in disease	25	0.1969	7.26E-12	2.97E-10
REACTOME_SIGNALING_BY_ILS	107	Genes involved in Signaling by Interleukins	23	0.215	7.38E-12	2.97E-10
KEGG_ENDOCYTOSIS	183	Endocytosis	30	0.1639	8.37E-12	3.27E-10
KEGG_CELL_CYCLE	128	Cell cycle	25	0.1953	8.71E-12	3.31E-10
KEGG_ADHERENS_JUNCTION	75	Adherens junction	19	0.2533	2.27E-11	8.36E-10
PID_PTP1B_PATHWAY	52	Signaling events mediated by PTP1B	16	0.3077	3.40E-11	1.22E-09
REACTOME_NGF_SIGNALLING_VIA_TRKA_FROM_THE_PLASMA_MEMBRANE	137	Genes involved in NGF signalling via TRKA from the plasma membrane	25	0.1825	4.12E-11	1.44E-09
KEGG_UBIQUITIN_MEDIATED_PROTEOLYSIS	138	Ubiquitin mediated proteolysis	25	0.1812	4.86E-11	1.66E-09
PID_CDC42_PATHWAY	70	CDC42 signaling events	18	0.2571	5.78E-11	1.91E-09
NABA_SECRETED_FACTORS	344	Genes encoding secreted soluble factors	41	0.1192	5.89E-11	1.91E-09
REACTOME_INTEGRIN_CELL_SURFACE_INTERACTIONS	79	Genes involved in Integrin cell surface interactions	19	0.2405	6.06E-11	1.92E-09
KEGG_PROSTATE_CANCER	89	Prostate cancer	20	0.2247	7.21E-11	2.23E-09
REACTOME_CELL_CYCLE	421	Genes involved in Cell Cycle	46	0.1093	8.04E-11	2.39E-09
BIOCARTA_P38MAPK_PATHWAY	40	p38 MAPK Signaling Pathway	14	0.35	8.08E-11	2.39E-09
PID_TGFBR_PATHWAY	55	TGF-beta receptor signaling	16	0.2909	8.79E-11	2.53E-09
REACTOME_SIGNALING_BY_ERBB4	90	Genes involved in Signaling by ERBB4	20	0.2222	8.95E-11	2.53E-09
PID_AJDISS_2PATHWAY	48	Posttranslational regulation of adherens junction stability and dissassembly	15	0.3125	1.09E-10	3.02E-09
REACTOME_SIGNALING_BY_ERBB2	101	Genes involved in Signaling by ERBB2	21	0.2079	1.16E-10	3.14E-09
PID_CXCR4_PATHWAY	102	CXCR4-mediated signaling events	21	0.2059	1.41E-10	3.74E-09
REACTOME_NUCLEAR_RECEPTOR_TRANSCRIPTION_PATHWAY	49	Genes involved in Nuclear Receptor transcription pathway	15	0.3061	1.52E-10	3.95E-09
PID_CMYB_PATHWAY	84	C-MYB transcription factor network	19	0.2262	1.90E-10	4.86E-09
REACTOME_SIGNALING_BY_THE_B_CELL_RECEPTOR_BCR	126	Genes involved in Signaling by the B Cell Receptor (BCR)	23	0.1825	2.44E-10	6.05E-09
PID_TCPTP_PATHWAY	43	Signaling events mediated by TCPTP	14	0.3256	2.46E-10	6.05E-09
REACTOME_DOWNSTREAM_SIGNAL_TRANSDUCTION	95	Genes involved in Downstream signal transduction	20	0.2105	2.52E-10	6.09E-09
REACTOME_PHOSPHOLIPID_METABOLISM	198	Genes involved in Phospholipid metabolism	29	0.1465	3.01E-10	7.14E-09
BIOCARTA_MAPK_PATHWAY	87	MAPKinase Signaling Pathway	19	0.2184	3.62E-10	8.43E-09
REACTOME_PLATELET_HOMEOSTASIS	78	Genes involved in Platelet homeostasis	18	0.2308	4.00E-10	9.17E-09
PID_AP1_PATHWAY	70	AP-1 transcription factor network	17	0.2429	5.20E-10	1.17E-08
REACTOME_INTEGRATION_OF_ENERGY_METABOLISM	120	Genes involved in Integration of energy metabolism	22	0.1833	5.45E-10	1.21E-08
REACTOME_DOWNSTREAM_SIGNALING_OF_ACTIVATED_FGFR	100	Genes involved in Downstream signaling of activated FGFR	20	0.2	6.61E-10	1.44E-08
REACTOME_DNA_REPLICATION	192	Genes involved in DNA Replication	28	0.1458	6.70E-10	1.44E-08
PID_RAC1_PATHWAY	54	RAC1 signaling pathway	15	0.2778	7.00E-10	1.48E-08
PID_WNT_NONCANONICAL_PATHWAY	32	Noncanonical Wnt signaling pathway	12	0.375	7.22E-10	1.50E-08
KEGG_GNRH_SIGNALING_PATHWAY	101	GnRH signaling pathway	20	0.198	7.96E-10	1.63E-08
REACTOME_CELL_SURFACE_INTERACTIONS_AT_THE_VASCULAR_WALL	91	Genes involved in Cell surface interactions at the vascular wall	19	0.2088	8.16E-10	1.64E-08
REACTOME_SIGNALING_BY_FGFR	112	Genes involved in Signaling by FGFR	21	0.1875	8.70E-10	1.73E-08
PID_IL2_1PATHWAY	55	IL2-mediated signaling events	15	0.2727	9.30E-10	1.82E-08
ST_INTEGRIN_SIGNALING_PATHWAY	82	Integrin Signaling Pathway	18	0.2195	9.57E-10	1.84E-08
KEGG_DILATED_CARDIOMYOPATHY	92	Dilated cardiomyopathy	19	0.2065	9.93E-10	1.88E-08
REACTOME_NCAM_SIGNALING_FOR_NEURITE_OUT_GROWTH	64	Genes involved in NCAM signaling for neurite out-growth	16	0.25	1.05E-09	1.97E-08
REACTOME_MITOTIC_M_M_G1_PHASES	172	Genes involved in Mitotic M-M/G1 phases	26	0.1512	1.23E-09	2.27E-08
KEGG_GLIOMA	65	Glioma	16	0.2462	1.35E-09	2.42E-08
PID_RB_1PATHWAY	65	Regulation of retinoblastoma protein	16	0.2462	1.35E-09	2.42E-08
REACTOME_ANTIGEN_PROCESSING_UBIQUITINATION_PROTEASOME_DEGRADATION	212	Genes involved in Antigen processing: Ubiquitination & Proteasome degradation	29	0.1368	1.54E-09	2.73E-08
KEGG_TGF_BETA_SIGNALING_PATHWAY	86	TGF-beta signaling pathway	18	0.2093	2.17E-09	3.72E-08
REACTOME_L1CAM_INTERACTIONS	86	Genes involved in L1CAM interactions	18	0.2093	2.17E-09	3.72E-08
SIG_PIP3_SIGNALING_IN_CARDIAC_MYOCTES	67	Genes related to PIP3 signaling in cardiac myocytes	16	0.2388	2.18E-09	3.72E-08
KEGG_CHEMOKINE_SIGNALING_PATHWAY	190	Chemokine signaling pathway	27	0.1421	2.40E-09	4.00E-08
ST_G_ALPHA_I_PATHWAY	35	G alpha i Pathway	12	0.3429	2.41E-09	4.00E-08
KEGG_FC_GAMMA_R_MEDIATED_PHAGOCYTOSIS	97	Fc gamma R-mediated phagocytosis	19	0.1959	2.54E-09	4.17E-08
KEGG_ERBB_SIGNALING_PATHWAY	87	ErbB signaling pathway	18	0.2069	2.64E-09	4.28E-08
PID_ATF2_PATHWAY	59	ATF-2 transcription factor network	15	0.2542	2.71E-09	4.34E-08
SIG_INSULIN_RECEPTOR_PATHWAY_IN_CARDIAC_MYOCYTES	51	Genes related to the insulin receptor pathway	14	0.2745	3.07E-09	4.86E-08
REACTOME_CLASS_B_2_SECRETIN_FAMILY_RECEPTORS	88	Genes involved in Class B/2 (Secretin family receptors)	18	0.2045	3.20E-09	5.01E-08
PID_P73PATHWAY	79	p73 transcription factor network	17	0.2152	3.85E-09	5.95E-08
PID_KIT_PATHWAY	52	Signaling events mediated by Stem cell factor receptor (c-Kit)	14	0.2692	4.06E-09	6.20E-08
KEGG_PANCREATIC_CANCER	70	Pancreatic cancer	16	0.2286	4.34E-09	6.48E-08
KEGG_RENAL_CELL_CARCINOMA	70	Renal cell carcinoma	16	0.2286	4.34E-09	6.48E-08
KEGG_GAP_JUNCTION	90	Gap junction	18	0.2	4.68E-09	6.91E-08
KEGG_MELANOMA	71	Melanoma	16	0.2254	5.41E-09	7.90E-08
BIOCARTA_BIOPEPTIDES_PATHWAY	45	Bioactive Peptide Induced Signaling Pathway	13	0.2889	5.65E-09	8.14E-08
PID_TRKR_PATHWAY	62	Neurotrophic factor-mediated Trk receptor signaling	15	0.2419	5.70E-09	8.14E-08
PID_LYMPH_ANGIOGENESIS_PATHWAY	25	VEGFR3 signaling in lymphatic endothelium	10	0.4	9.24E-09	1.31E-07
PID_IL6_7_PATHWAY	47	IL6-mediated signaling events	13	0.2766	1.02E-08	1.42E-07
KEGG_HYPERTROPHIC_CARDIOMYOPATHY_HCM	85	Hypertrophic cardiomyopathy (HCM)	17	0.2	1.25E-08	1.73E-07
PID_EPHB_FWD_PATHWAY	40	EPHB forward signaling	12	0.3	1.36E-08	1.86E-07
PID_HIF1_TFPATHWAY	66	HIF-1-alpha transcription factor network	15	0.2273	1.43E-08	1.94E-07
REACTOME_DOWNSTREAM_SIGNALING_EVENTS_OF_B_CELL_RECEPTOR_BCR	97	Genes involved in Downstream Signaling Events Of B Cell Receptor (BCR)	18	0.1856	1.62E-08	2.18E-07
BIOCARTA_IL3_PATHWAY	15	IL 3 signaling pathway	8	0.5333	1.77E-08	2.35E-07

Gene/Gene Set Overlap Matrix

Entrez Gene Id	Gene Symbol	Gene Description	KEGG_PATHWAYS_IN_CANCER	REACTOME_DEVELOPMENTAL_BIOLOGY	REACTOME_IMMUNE_SYSTEM	REACTOME_HEMOSTASIS
5894	RAF1	v-raf-1 murine leukemia viral oncogene homolog 1	KEGG_PATHWAYS_IN_CANCER	REACTOME_DEVELOPMENTAL_BIOLOGY	REACTOME_IMMUNE_SYSTEM	REACTOME_HEMOSTASIS
998	CDC42	cell division cycle 42 (GTP binding protein, 25kDa)	KEGG_PATHWAYS_IN_CANCER	REACTOME_DEVELOPMENTAL_BIOLOGY	REACTOME_IMMUNE_SYSTEM	REACTOME_HEMOSTASIS
6654	SOS1	son of sevenless homolog 1 (Drosophila)	KEGG_PATHWAYS_IN_CANCER	REACTOME_DEVELOPMENTAL_BIOLOGY	REACTOME_IMMUNE_SYSTEM	REACTOME_HEMOSTASIS
2885	GRB2	growth factor receptor-bound protein 2	KEGG_PATHWAYS_IN_CANCER	REACTOME_DEVELOPMENTAL_BIOLOGY	REACTOME_IMMUNE_SYSTEM	REACTOME_HEMOSTASIS
3845	KRAS	v-Ki-ras2 Kirsten rat sarcoma viral oncogene homolog	KEGG_PATHWAYS_IN_CANCER	REACTOME_DEVELOPMENTAL_BIOLOGY	REACTOME_IMMUNE_SYSTEM	REACTOME_HEMOSTASIS
3688	ITGB1	integrin, beta 1 (fibronectin receptor, beta polypeptide, antigen CD29 includes MDF2, MSK12)	KEGG_PATHWAYS_IN_CANCER	REACTOME_DEVELOPMENTAL_BIOLOGY	REACTOME_IMMUNE_SYSTEM	REACTOME_HEMOSTASIS
3685	ITGAV	integrin, alpha V (vitronectin receptor, alpha polypeptide, antigen CD51)	KEGG_PATHWAYS_IN_CANCER	REACTOME_DEVELOPMENTAL_BIOLOGY	REACTOME_IMMUNE_SYSTEM	REACTOME_HEMOSTASIS
2308	FOXO1	forkhead box O1	KEGG_PATHWAYS_IN_CANCER	REACTOME_DEVELOPMENTAL_BIOLOGY	REACTOME_IMMUNE_SYSTEM	
3673	ITGA2	integrin, alpha 2 (CD49B, alpha 2 subunit of VLA-2 receptor)	KEGG_PATHWAYS_IN_CANCER	REACTOME_DEVELOPMENTAL_BIOLOGY		REACTOME_HEMOSTASIS
7040	TGFB1	transforming growth factor, beta 1	KEGG_PATHWAYS_IN_CANCER	REACTOME_DEVELOPMENTAL_BIOLOGY		REACTOME_HEMOSTASIS
1956	EGFR	epidermal growth factor receptor	KEGG_PATHWAYS_IN_CANCER	REACTOME_DEVELOPMENTAL_BIOLOGY		
2260	FGFR1	fibroblast growth factor receptor 1	KEGG_PATHWAYS_IN_CANCER	REACTOME_DEVELOPMENTAL_BIOLOGY		
4233	MET	met proto-oncogene (hepatocyte growth factor receptor)	KEGG_PATHWAYS_IN_CANCER	REACTOME_DEVELOPMENTAL_BIOLOGY		
3915	LAMC1	laminin, gamma 1 (formerly LAMB2)	KEGG_PATHWAYS_IN_CANCER	REACTOME_DEVELOPMENTAL_BIOLOGY		
5468	PPARG	peroxisome proliferator-activated receptor gamma	KEGG_PATHWAYS_IN_CANCER	REACTOME_DEVELOPMENTAL_BIOLOGY		
7189	TRAF6	TNF receptor-associated factor 6	KEGG_PATHWAYS_IN_CANCER		REACTOME_IMMUNE_SYSTEM	
5728	PTEN	phosphatase and tensin homolog	KEGG_PATHWAYS_IN_CANCER		REACTOME_IMMUNE_SYSTEM	
1026	CDKN1A	cyclin-dependent kinase inhibitor 1A (p21, Cip1)	KEGG_PATHWAYS_IN_CANCER		REACTOME_IMMUNE_SYSTEM	
6921	TCEB1	transcription elongation factor B (SIII), polypeptide 1 (15kDa, elongin C)	KEGG_PATHWAYS_IN_CANCER		REACTOME_IMMUNE_SYSTEM	
8453	CUL2	cullin 2	KEGG_PATHWAYS_IN_CANCER		REACTOME_IMMUNE_SYSTEM	
7704	ZBTB16	zinc finger and BTB domain containing 16	KEGG_PATHWAYS_IN_CANCER		REACTOME_IMMUNE_SYSTEM	
5601	MAPK9	mitogen-activated protein kinase 9	KEGG_PATHWAYS_IN_CANCER		REACTOME_IMMUNE_SYSTEM	
4791	NFKB2	nuclear factor of kappa light polypeptide gene enhancer in B-cells 2 (p49/p100)	KEGG_PATHWAYS_IN_CANCER		REACTOME_IMMUNE_SYSTEM	
6776	STAT5A	signal transducer and activator of transcription 5A	KEGG_PATHWAYS_IN_CANCER		REACTOME_IMMUNE_SYSTEM	
6777	STAT5B	signal transducer and activator of transcription 5B	KEGG_PATHWAYS_IN_CANCER		REACTOME_IMMUNE_SYSTEM	
6774	STAT3	signal transducer and activator of transcription 3 (acute-phase response factor)	KEGG_PATHWAYS_IN_CANCER		REACTOME_IMMUNE_SYSTEM	
598	BCL2L1	BCL2-like 1	KEGG_PATHWAYS_IN_CANCER		REACTOME_IMMUNE_SYSTEM	
5371	PML	promyelocytic leukemia	KEGG_PATHWAYS_IN_CANCER		REACTOME_IMMUNE_SYSTEM	
7184	HSP90B1	heat shock protein 90kDa beta (Grp94), member 1	KEGG_PATHWAYS_IN_CANCER		REACTOME_IMMUNE_SYSTEM	
7157	TP53	tumor protein p53	KEGG_PATHWAYS_IN_CANCER			REACTOME_HEMOSTASIS
5578	PRKCA	protein kinase C, alpha	KEGG_PATHWAYS_IN_CANCER			REACTOME_HEMOSTASIS
7042	TGFB2	transforming growth factor, beta 2	KEGG_PATHWAYS_IN_CANCER			REACTOME_HEMOSTASIS
7043	TGFB3	transforming growth factor, beta 3	KEGG_PATHWAYS_IN_CANCER			REACTOME_HEMOSTASIS
7422	VEGFA	vascular endothelial growth factor A	KEGG_PATHWAYS_IN_CANCER			REACTOME_HEMOSTASIS
7424	VEGFC	vascular endothelial growth factor C	KEGG_PATHWAYS_IN_CANCER			REACTOME_HEMOSTASIS
4609	MYC	v-myc myelocytomatosis viral oncogene homolog (avian)	KEGG_PATHWAYS_IN_CANCER			
5156	PDGFRA	platelet-derived growth factor receptor, alpha polypeptide	KEGG_PATHWAYS_IN_CANCER			
8074	FGF23	fibroblast growth factor 23	KEGG_PATHWAYS_IN_CANCER			
8817	FGF18	fibroblast growth factor 18	KEGG_PATHWAYS_IN_CANCER			
2256	FGF11	fibroblast growth factor 11	KEGG_PATHWAYS_IN_CANCER			
2257	FGF12	fibroblast growth factor 12	KEGG_PATHWAYS_IN_CANCER			
2263	FGFR2	fibroblast growth factor receptor 2	KEGG_PATHWAYS_IN_CANCER			
7474	WNT5A	wingless-type MMTV integration site family, member 5A	KEGG_PATHWAYS_IN_CANCER			
7476	WNT7A	wingless-type MMTV integration site family, member 7A	KEGG_PATHWAYS_IN_CANCER			
7475	WNT6	wingless-type MMTV integration site family, member 6	KEGG_PATHWAYS_IN_CANCER			
7482	WNT2B	wingless-type MMTV integration site family, member 2B	KEGG_PATHWAYS_IN_CANCER			
7483	WNT9A	wingless-type MMTV integration site family, member 9A	KEGG_PATHWAYS_IN_CANCER			
80326	WNT10A	wingless-type MMTV integration site family, member 10A	KEGG_PATHWAYS_IN_CANCER			
81029	WNT5B	wingless-type MMTV integration site family, member 5B	KEGG_PATHWAYS_IN_CANCER			
51176	LEF1	lymphoid enhancer-binding factor 1	KEGG_PATHWAYS_IN_CANCER			
8324	FZD7	frizzled family receptor 7	KEGG_PATHWAYS_IN_CANCER			
8323	FZD6	frizzled family receptor 6	KEGG_PATHWAYS_IN_CANCER			
1856	DVL2	dishevelled, dsh homolog 2 (Drosophila)	KEGG_PATHWAYS_IN_CANCER			
8325	FZD8	frizzled family receptor 8	KEGG_PATHWAYS_IN_CANCER			
8312	AXIN1	axin 1	KEGG_PATHWAYS_IN_CANCER			
7479	WNT8B	wingless-type MMTV integration site family, member 8B	KEGG_PATHWAYS_IN_CANCER			
7484	WNT9B	wingless-type MMTV integration site family, member 9B	KEGG_PATHWAYS_IN_CANCER			
6934	TCF7L2	transcription factor 7-like 2 (T-cell specific, HMG-box)	KEGG_PATHWAYS_IN_CANCER			
1488	CTBP2	C-terminal binding protein 2	KEGG_PATHWAYS_IN_CANCER			
652	BMP4	bone morphogenetic protein 4	KEGG_PATHWAYS_IN_CANCER			
64399	HHIP	hedgehog interacting protein	KEGG_PATHWAYS_IN_CANCER			
5727	PTCH1	patched 1	KEGG_PATHWAYS_IN_CANCER			
8643	PTCH2	patched 2	KEGG_PATHWAYS_IN_CANCER			

2735	GLI1	GLI family zinc finger 1	KEGG_PATHWAYS_IN_CANCER			
2737	GLI3	GLI family zinc finger 3	KEGG_PATHWAYS_IN_CANCER			
3913	LAMB2	laminin, beta 2 (laminin S)	KEGG_PATHWAYS_IN_CANCER			
1870	E2F2	E2F transcription factor 2	KEGG_PATHWAYS_IN_CANCER			
1029	CDKN2A	cyclin-dependent kinase inhibitor 2A (melanoma, p16, inhibits CDK4)	KEGG_PATHWAYS_IN_CANCER			
23624	CBLC	Cas-Br-M (murine) ecotropic retroviral transforming sequence c	KEGG_PATHWAYS_IN_CANCER			
861	RUNX1	runt-related transcription factor 1	KEGG_PATHWAYS_IN_CANCER			
1496	CTNNA2	catenin (cadherin-associated protein), alpha 2	KEGG_PATHWAYS_IN_CANCER			
2113	ETS1	v-ets erythroblastosis virus E26 oncogene homolog 1 (avian)	KEGG_PATHWAYS_IN_CANCER			
5743	PTGS2	prostaglandin-endoperoxide synthase 2 (prostaglandin G/H synthase and cyclooxygenase)	KEGG_PATHWAYS_IN_CANCER			
7170	TPM3	tropomyosin 3	KEGG_PATHWAYS_IN_CANCER			
5888	RAD51	RAD51 homolog (S. cerevisiae)	KEGG_PATHWAYS_IN_CANCER			
405	ARNT	aryl hydrocarbon receptor nuclear translocator	KEGG_PATHWAYS_IN_CANCER			
11186	RASSF1	Ras association (RalGDS/AF-6) domain family member 1	KEGG_PATHWAYS_IN_CANCER			
862	RUNX1T1	runt-related transcription factor 1; translocated to, 1 (cyclin D-related)	KEGG_PATHWAYS_IN_CANCER			
9618	TRAF4	TNF receptor-associated factor 4	KEGG_PATHWAYS_IN_CANCER			
2534	FYN	FYN oncogene related to SRC, FGR, YES		REACTOME_DEVELOPMENTAL_BIOLOGY	REACTOME_IMMUNE_SYSTEM	REACTOME_HEMOSTASIS
1432	MAPK14	mitogen-activated protein kinase 14		REACTOME_DEVELOPMENTAL_BIOLOGY	REACTOME_IMMUNE_SYSTEM	REACTOME_HEMOSTASIS
1385	CREB1	cAMP responsive element binding protein 1		REACTOME_DEVELOPMENTAL_BIOLOGY	REACTOME_IMMUNE_SYSTEM	
1173	AP2M1	adaptor-related protein complex 2, mu 1 subunit		REACTOME_DEVELOPMENTAL_BIOLOGY	REACTOME_IMMUNE_SYSTEM	
163	AP2B1	adaptor-related protein complex 2, beta 1 subunit		REACTOME_DEVELOPMENTAL_BIOLOGY	REACTOME_IMMUNE_SYSTEM	
55740	ENAH	enabled homolog (Drosophila)		REACTOME_DEVELOPMENTAL_BIOLOGY	REACTOME_IMMUNE_SYSTEM	
9252	RPS6KA5	ribosomal protein S6 kinase, 90kDa, polypeptide 5		REACTOME_DEVELOPMENTAL_BIOLOGY	REACTOME_IMMUNE_SYSTEM	
1277	COL1A1	collagen, type I, alpha 1		REACTOME_DEVELOPMENTAL_BIOLOGY		REACTOME_HEMOSTASIS
7094	TLN1	talin 1		REACTOME_DEVELOPMENTAL_BIOLOGY		REACTOME_HEMOSTASIS
1072	CFL1	cofilin 1 (non-muscle)		REACTOME_DEVELOPMENTAL_BIOLOGY		REACTOME_HEMOSTASIS
57113	TRPC7	transient receptor potential cation channel, subfamily C, member 7		REACTOME_DEVELOPMENTAL_BIOLOGY		REACTOME_HEMOSTASIS
783	CACNB2	calcium channel, voltage-dependent, beta 2 subunit		REACTOME_DEVELOPMENTAL_BIOLOGY		
784	CACNB3	calcium channel, voltage-dependent, beta 3 subunit		REACTOME_DEVELOPMENTAL_BIOLOGY		
8913	CACNA1G	calcium channel, voltage-dependent, T type, alpha 1G subunit		REACTOME_DEVELOPMENTAL_BIOLOGY		
8912	CACNA1H	calcium channel, voltage-dependent, T type, alpha 1H subunit		REACTOME_DEVELOPMENTAL_BIOLOGY		
6093	ROCK1	Rho-associated, coiled-coil containing protein kinase 1		REACTOME_DEVELOPMENTAL_BIOLOGY		
1280	COL2A1	collagen, type II, alpha 1		REACTOME_DEVELOPMENTAL_BIOLOGY		
1290	COL5A2	collagen, type V, alpha 2		REACTOME_DEVELOPMENTAL_BIOLOGY		
10154	PLXNC1	plexin C1		REACTOME_DEVELOPMENTAL_BIOLOGY		
57556	SEMA6A	sema domain, transmembrane domain (TM), and cytoplasmic domain, (semaphorin) 6A		REACTOME_DEVELOPMENTAL_BIOLOGY		
9037	SEMA5A	sema domain, seven thrombospondin repeats (type 1 and type 1-like), transmembrane domain (TM) and short cytoplasmic domain, (semaphorin) 5A		REACTOME_DEVELOPMENTAL_BIOLOGY		
10371	SEMA3A	sema domain, immunoglobulin domain (Ig), short basic domain, secreted, (semaphorin) 3A		REACTOME_DEVELOPMENTAL_BIOLOGY		
9423	NTN1	netrin 1		REACTOME_DEVELOPMENTAL_BIOLOGY		
3984	LIMK1	LIM domain kinase 1		REACTOME_DEVELOPMENTAL_BIOLOGY		
6091	ROBO1	roundabout, axon guidance receptor, homolog 1 (Drosophila)		REACTOME_DEVELOPMENTAL_BIOLOGY		
8829	NRP1	neuropilin 1		REACTOME_DEVELOPMENTAL_BIOLOGY		
23380	SRGAP2	SLIT-ROBO Rho GTPase activating protein 2		REACTOME_DEVELOPMENTAL_BIOLOGY		
9901	SRGAP3	SLIT-ROBO Rho GTPase activating protein 3		REACTOME_DEVELOPMENTAL_BIOLOGY		
8633	UNC5C	unc-5 homolog C (C. elegans)		REACTOME_DEVELOPMENTAL_BIOLOGY		
23129	PLXND1	plexin D1		REACTOME_DEVELOPMENTAL_BIOLOGY		
7204	TRIO	triple functional domain (PTPRF interacting)		REACTOME_DEVELOPMENTAL_BIOLOGY		
23332	CLASP1	cytoplasmic linker associated protein 1		REACTOME_DEVELOPMENTAL_BIOLOGY		
389	RHOC	ras homolog gene family, member C		REACTOME_DEVELOPMENTAL_BIOLOGY		
2675	GFRA2	GDNF family receptor alpha 2		REACTOME_DEVELOPMENTAL_BIOLOGY		
8650	NUMB	numb homolog (Drosophila)		REACTOME_DEVELOPMENTAL_BIOLOGY		
8828	NRP2	neuropilin 2		REACTOME_DEVELOPMENTAL_BIOLOGY		
10963	STIP1	stress-induced-phosphoprotein 1		REACTOME_DEVELOPMENTAL_BIOLOGY		
214	ALCAM	activated leukocyte cell adhesion molecule		REACTOME_DEVELOPMENTAL_BIOLOGY		
8506	CNTNAP1	contactin associated protein 1		REACTOME_DEVELOPMENTAL_BIOLOGY		
286	ANK1	ankyrin 1, erythrocytic		REACTOME_DEVELOPMENTAL_BIOLOGY		
6334	SCN8A	sodium channel, voltage gated, type VIII, alpha subunit		REACTOME_DEVELOPMENTAL_BIOLOGY		
100133941	CD24	CD24 molecule		REACTOME_DEVELOPMENTAL_BIOLOGY		
4651	MYO10	myosin X		REACTOME_DEVELOPMENTAL_BIOLOGY		
6478	SIAH2	seven in absentia homolog 2 (Drosophila)		REACTOME_DEVELOPMENTAL_BIOLOGY		
896	CCND3	cyclin D3		REACTOME_DEVELOPMENTAL_BIOLOGY		
51129	ANGPTL4	angiopoietin-like 4		REACTOME_DEVELOPMENTAL_BIOLOGY		
4838	NODAL	nodal homolog (mouse)		REACTOME_DEVELOPMENTAL_BIOLOGY		
9370	ADIPOQ	adiponectin, C1Q and collagen domain containing		REACTOME_DEVELOPMENTAL_BIOLOGY		
5469	MED1	mediator complex subunit 1		REACTOME_DEVELOPMENTAL_BIOLOGY		
6604	SMARCD3	SWI/SNF related, matrix associated, actin dependent regulator of chromatin, subfamily d, member 3		REACTOME_DEVELOPMENTAL_BIOLOGY		
9477	MED20	mediator complex subunit 20		REACTOME_DEVELOPMENTAL_BIOLOGY		
9862	MED24	mediator complex subunit 24		REACTOME_DEVELOPMENTAL_BIOLOGY		
55588	MED29	mediator complex subunit 29		REACTOME_DEVELOPMENTAL_BIOLOGY		
9412	MED21	mediator complex subunit 21		REACTOME_DEVELOPMENTAL_BIOLOGY		
5080	PAX6	paired box 6		REACTOME_DEVELOPMENTAL_BIOLOGY		
4209	MEF2D	myocyte enhancer factor 2D		REACTOME_DEVELOPMENTAL_BIOLOGY		
3280	HES1	hairy and enhancer of split 1, (Drosophila)		REACTOME_DEVELOPMENTAL_BIOLOGY		
6517	SLC2A4	solute carrier family 2 (facilitated glucose transporter), member 4		REACTOME_DEVELOPMENTAL_BIOLOGY		
6925	TCF4	transcription factor 4		REACTOME_DEVELOPMENTAL_BIOLOGY		
3516	RBPJ	recombination signal binding protein for immunoglobulin kappa J region		REACTOME_DEVELOPMENTAL_BIOLOGY		
6929	TCF3	transcription factor 3 (E2A immunoglobulin enhancer binding factors E12/E47)		REACTOME_DEVELOPMENTAL_BIOLOGY		
7026	NR2F2	nuclear receptor subfamily 2, group F, member 2		REACTOME_DEVELOPMENTAL_BIOLOGY		
3651	PDX1	pancreatic and duodenal homeobox 1		REACTOME_DEVELOPMENTAL_BIOLOGY		
3175	ONECUT1	one cut homeobox 1		REACTOME_DEVELOPMENTAL_BIOLOGY		
5516	PPP2CB	protein phosphatase 2, catalytic subunit, beta isozyme			REACTOME_IMMUNE_SYSTEM	REACTOME_HEMOSTASIS
5526	PPP2R5B	protein phosphatase 2, regulatory subunit B', beta			REACTOME_IMMUNE_SYSTEM	REACTOME_HEMOSTASIS
5527	PPP2R5C	protein phosphatase 2, regulatory subunit B', gamma			REACTOME_IMMUNE_SYSTEM	REACTOME_HEMOSTASIS
6464	SHC1	SHC (Src homology 2 domain containing) transforming protein 1			REACTOME_IMMUNE_SYSTEM	REACTOME_HEMOSTASIS
7534	YWHAZ	tyrosine 3-monooxygenase/tryptophan 5-monooxygenase activation protein, zeta polypeptide			REACTOME_IMMUNE_SYSTEM	REACTOME_HEMOSTASIS
7525	YES1	v-yes-1 Yamaguchi sarcoma viral oncogene homolog 1			REACTOME_IMMUNE_SYSTEM	REACTOME_HEMOSTASIS
3309	HSPA5	heat shock 70kDa protein 5 (glucose-regulated protein, 78kDa)			REACTOME_IMMUNE_SYSTEM	REACTOME_HEMOSTASIS
3709	ITPR2	inositol 1,4,5-trisphosphate receptor, type 2			REACTOME_IMMUNE_SYSTEM	REACTOME_HEMOSTASIS
10411	RAPGEF3	Rap guanine nucleotide exchange factor (GEF) 3			REACTOME_IMMUNE_SYSTEM	REACTOME_HEMOSTASIS
5777	PTPN6	protein tyrosine phosphatase, non-receptor type 6			REACTOME_IMMUNE_SYSTEM	REACTOME_HEMOSTASIS
3796	KIF2A	kinesin heavy chain member 2A			REACTOME_IMMUNE_SYSTEM	REACTOME_HEMOSTASIS
5592	PRKG1	protein kinase, cGMP-dependent, type I			REACTOME_IMMUNE_SYSTEM	REACTOME_HEMOSTASIS
3831	KLC1	kinesin light chain 1			REACTOME_IMMUNE_SYSTEM	REACTOME_HEMOSTASIS
3832	KIF11	kinesin family member 11			REACTOME_IMMUNE_SYSTEM	REACTOME_HEMOSTASIS
5770	PTPN1	protein tyrosine phosphatase, non-receptor type 1			REACTOME_IMMUNE_SYSTEM	REACTOME_HEMOSTASIS
3717	JAK2	Janus kinase 2			REACTOME_IMMUNE_SYSTEM	REACTOME_HEMOSTASIS
351	APP	amyloid beta (A4) precursor protein			REACTOME_IMMUNE_SYSTEM	REACTOME_HEMOSTASIS
3659	IRF1	interferon regulatory factor 1			REACTOME_IMMUNE_SYSTEM	REACTOME_HEMOSTASIS
6885	MAP3K7	mitogen-activated protein kinase kinase kinase 7			REACTOME_IMMUNE_SYSTEM	
9020	MAP3K14	mitogen-activated protein kinase kinase kinase 14			REACTOME_IMMUNE_SYSTEM	
8945	BTRC	beta-transducin repeat containing			REACTOME_IMMUNE_SYSTEM	
3693	ITGB5	integrin, beta 5			REACTOME_IMMUNE_SYSTEM	
1508	CTSB	cathepsin B			REACTOME_IMMUNE_SYSTEM	
57761	TRIB3	tribbles homolog 3 (Drosophila)			REACTOME_IMMUNE_SYSTEM	
9021	SOCS3	suppressor of cytokine signaling 3			REACTOME_IMMUNE_SYSTEM	
2209	FCGR1A	Fc fragment of IgG, high affinity Ia, receptor (CD64)			REACTOME_IMMUNE_SYSTEM	
51434	ANAPC7	anaphase promoting complex subunit 7			REACTOME_IMMUNE_SYSTEM	
996	CDC27	cell division cycle 27 homolog (S. cerevisiae)			REACTOME_IMMUNE_SYSTEM	
5682	PSMA1	proteasome (prosome, macropain) subunit, alpha type, 1			REACTOME_IMMUNE_SYSTEM	
5686	PSMA5	proteasome (prosome, macropain) subunit, alpha type, 5			REACTOME_IMMUNE_SYSTEM	
5695	PSMB7	proteasome (prosome, macropain) subunit, beta type, 7			REACTOME_IMMUNE_SYSTEM	
5717	PSMD11	proteasome (prosome, macropain) 26S subunit, non-ATPase, 11			REACTOME_IMMUNE_SYSTEM	
5699	PSMB10	proteasome (prosome, macropain) subunit, beta type, 10			REACTOME_IMMUNE_SYSTEM	
5705	PSMC5	proteasome (prosome, macropain) 26S subunit, ATPase, 5			REACTOME_IMMUNE_SYSTEM	
5713	PSMD7	proteasome (prosome, macropain) 26S subunit, non-ATPase, 7			REACTOME_IMMUNE_SYSTEM	
9491	PSMF1	proteasome (prosome, macropain) inhibitor subunit 1 (PI31)			REACTOME_IMMUNE_SYSTEM	
26272	FBXO4	F-box protein 4			REACTOME_IMMUNE_SYSTEM	
7323	UBE2D3	ubiquitin-conjugating enzyme E2D 3			REACTOME_IMMUNE_SYSTEM	
9817	KEAP1	kelch-like ECH-associated protein 1			REACTOME_IMMUNE_SYSTEM	
7327	UBE2G2	ubiquitin-conjugating enzyme E2G 2			REACTOME_IMMUNE_SYSTEM	
7325	UBE2E2	ubiquitin-conjugating enzyme E2E 2			REACTOME_IMMUNE_SYSTEM	
26232	FBXO2	F-box protein 2			REACTOME_IMMUNE_SYSTEM	
26259	FBXW8	F-box and WD repeat domain containing 8			REACTOME_IMMUNE_SYSTEM	
8924	HERC2	hect domain and RLD 2			REACTOME_IMMUNE_SYSTEM	
255488	RNF144B	ring finger protein 144B			REACTOME_IMMUNE_SYSTEM	
26270	FBXO6	F-box protein 6			REACTOME_IMMUNE_SYSTEM	
27252	KLHL20	kelch-like 20 (Drosophila)			REACTOME_IMMUNE_SYSTEM	
7174	TPP2	tripeptidyl peptidase II			REACTOME_IMMUNE_SYSTEM	
80176	SPSB1	splA/ryanodine receptor domain and SOCS box containing 1			REACTOME_IMMUNE_SYSTEM	
9520	NPEPPS	aminopeptidase puromycin sensitive			REACTOME_IMMUNE_SYSTEM	
811	CALR	calreticulin			REACTOME_IMMUNE_SYSTEM	
2923	PDIA3	protein disulfide isomerase family A, member 3			REACTOME_IMMUNE_SYSTEM	
6890	TAP1	transporter 1, ATP-binding cassette, sub-family B (MDR/TAP)			REACTOME_IMMUNE_SYSTEM	
9902	MRC2	mannose receptor, C type 2			REACTOME_IMMUNE_SYSTEM	
821	CANX	calnexin			REACTOME_IMMUNE_SYSTEM	
79109	MAPKAP1	mitogen-activated protein kinase associated protein 1			REACTOME_IMMUNE_SYSTEM	
4303	FOXO4	forkhead box O4			REACTOME_IMMUNE_SYSTEM	
3384	ICAM2	intercellular adhesion molecule 2			REACTOME_IMMUNE_SYSTEM	
3386	ICAM4	intercellular adhesion molecule 4 (Landsteiner-Wiener blood group)			REACTOME_IMMUNE_SYSTEM	
164	AP1G1	adaptor-related protein complex 1, gamma 1 subunit			REACTOME_IMMUNE_SYSTEM	
941	CD80	CD80 molecule			REACTOME_IMMUNE_SYSTEM	
718	C3	complement component 3			REACTOME_IMMUNE_SYSTEM	
23308	ICOSLG	inducible T-cell co-stimulator ligand			REACTOME_IMMUNE_SYSTEM	
114876	OSBPL1A	oxysterol binding protein-like 1A			REACTOME_IMMUNE_SYSTEM	
5609	MAP2K7	mitogen-activated protein kinase kinase 7			REACTOME_IMMUNE_SYSTEM	
929	CD14	CD14 molecule			REACTOME_IMMUNE_SYSTEM	
1845	DUSP3	dual specificity phosphatase 3			REACTOME_IMMUNE_SYSTEM	
4215	MAP3K3	mitogen-activated protein kinase kinase kinase 3			REACTOME_IMMUNE_SYSTEM	
3554	IL1R1	interleukin 1 receptor, type I			REACTOME_IMMUNE_SYSTEM	
1386	ATF2	activating transcription factor 2			REACTOME_IMMUNE_SYSTEM	
817	CAMK2D	calcium/calmodulin-dependent protein kinase II delta			REACTOME_IMMUNE_SYSTEM	
6285	S100B	S100 calcium binding protein B			REACTOME_IMMUNE_SYSTEM	
713	C1QB	complement component 1, q subcomponent, B chain			REACTOME_IMMUNE_SYSTEM	
714	C1QC	complement component 1, q subcomponent, C chain			REACTOME_IMMUNE_SYSTEM	
8878	SQSTM1	sequestosome 1			REACTOME_IMMUNE_SYSTEM	
81929	SEH1L	SEH1-like (S. cerevisiae)			REACTOME_IMMUNE_SYSTEM	
3556	IL1RAP	interleukin 1 receptor accessory protein			REACTOME_IMMUNE_SYSTEM	
3570	IL6R	interleukin 6 receptor			REACTOME_IMMUNE_SYSTEM	
3837	KPNB1	karyopherin (importin) beta 1			REACTOME_IMMUNE_SYSTEM	
4502	MT2A	metallothionein 2A			REACTOME_IMMUNE_SYSTEM	
1154	CISH	cytokine inducible SH2-containing protein			REACTOME_IMMUNE_SYSTEM	
23636	NUP62	nucleoporin 62kDa			REACTOME_IMMUNE_SYSTEM	
53371	NUP54	nucleoporin 54kDa			REACTOME_IMMUNE_SYSTEM	
3664	IRF6	interferon regulatory factor 6			REACTOME_IMMUNE_SYSTEM	
3430	IFI35	interferon-induced protein 35			REACTOME_IMMUNE_SYSTEM	
6041	RNASEL	ribonuclease L (2',5'-oligoisoadenylate synthetase-dependent)			REACTOME_IMMUNE_SYSTEM	
3840	KPNA4	karyopherin alpha 4 (importin alpha 3)			REACTOME_IMMUNE_SYSTEM	
81622	UNC93B1	unc-93 homolog B1 (C. elegans)			REACTOME_IMMUNE_SYSTEM	
64135	IFIH1	interferon induced with helicase C domain 1			REACTOME_IMMUNE_SYSTEM	
79132	DHX58	DEXH (Asp-Glu-X-His) box polypeptide 58			REACTOME_IMMUNE_SYSTEM	
10628	TXNIP	thioredoxin interacting protein			REACTOME_IMMUNE_SYSTEM	
10681	GNB5	guanine nucleotide binding protein (G protein), beta 5				REACTOME_HEMOSTASIS
9564	BCAR1	breast cancer anti-estrogen resistance 1				REACTOME_HEMOSTASIS
10451	VAV3	vav 3 guanine nucleotide exchange factor				REACTOME_HEMOSTASIS
2268	FGR	Gardner-Rasheed feline sarcoma viral (v-fgr) oncogene homolog				REACTOME_HEMOSTASIS
7277	TUBA4A	tubulin, alpha 4a				REACTOME_HEMOSTASIS
2207	FCER1G	Fc fragment of IgE, high affinity I, receptor for; gamma polypeptide				REACTOME_HEMOSTASIS

1606	DGKA	diacylglycerol kinase, alpha 80kDa	REACTOME_HEMOSTASIS
8526	DGKE	diacylglycerol kinase, epsilon 64kDa	REACTOME_HEMOSTASIS
747	DAGLA	diacylglycerol lipase, alpha	REACTOME_HEMOSTASIS
6647	SOD1	superoxide dismutase 1, soluble	REACTOME_HEMOSTASIS
6814	STXBP3	syntaxin binding protein 3	REACTOME_HEMOSTASIS
813	CALU	calumenin	REACTOME_HEMOSTASIS
10019	SH2B3	SH2B adaptor protein 3	REACTOME_HEMOSTASIS
10938	EHD1	EH-domain containing 1	REACTOME_HEMOSTASIS
30846	EHD2	EH-domain containing 2	REACTOME_HEMOSTASIS
50848	F11R	F11 receptor	REACTOME_HEMOSTASIS
682	BSG	basigin (Ok blood group)	REACTOME_HEMOSTASIS
2625	GATA3	GATA binding protein 3	REACTOME_HEMOSTASIS
161882	ZFPM1	zinc finger protein, multitype 1	REACTOME_HEMOSTASIS
5140	PDE3B	phosphodiesterase 3B, cGMP-inhibited	REACTOME_HEMOSTASIS
5139	PDE3A	phosphodiesterase 3A, cGMP-inhibited	REACTOME_HEMOSTASIS
487	ATP2A1	ATPase, Ca++ transporting, cardiac muscle, fast twitch 1	REACTOME_HEMOSTASIS
6543	SLC8A2	solute carrier family 8 (sodium/calcium exchanger), member 2	REACTOME_HEMOSTASIS
4846	NOS3	nitric oxide synthase 3 (endothelial cell)	REACTOME_HEMOSTASIS
4842	NOS1	nitric oxide synthase 1 (neuronal)	REACTOME_HEMOSTASIS
7804	LRP8	low density lipoprotein receptor-related protein 8, apolipoprotein e receptor	REACTOME_HEMOSTASIS
5152	PDE9A	phosphodiesterase 9A	REACTOME_HEMOSTASIS
23539	SLC16A8	solute carrier family 16, member 8 (monocarboxylic acid transporter 3)	REACTOME_HEMOSTASIS
9123	SLC16A3	solute carrier family 16, member 3 (monocarboxylic acid transporter 4)	REACTOME_HEMOSTASIS
9056	SLC7A7	solute carrier family 7 (amino acid transporter light chain, y+L system), member 7	REACTOME_HEMOSTASIS
481	ATP1B1	ATPase, Na+/K+ transporting, beta 1 polypeptide	REACTOME_HEMOSTASIS
482	ATP1B2	ATPase, Na+/K+ transporting, beta 2 polypeptide	REACTOME_HEMOSTASIS
961	CD47	CD47 molecule	REACTOME_HEMOSTASIS
23764	MAFF	v-maf musculoaponeurotic fibrosarcoma oncogene homolog F (avian)	REACTOME_HEMOSTASIS
23468	CBX5	chromobox homolog 5	REACTOME_HEMOSTASIS
4778	NFE2	nuclear factor (erythroid-derived 2), 45kDa	REACTOME_HEMOSTASIS
5547	PRCP	prolylcarboxypeptidase (angiotensinase C)	REACTOME_HEMOSTASIS
51701	NLK	nemo-like kinase	
5923	RASGRF1	Ras protein-specific guanine nucleotide-releasing factor 1	
3312	HSPA8	heat shock 70kDa protein 8	
773	CACNA1A	calcium channel, voltage-dependent, P/Q type, alpha 1A subunit	
10368	CACNG3	calcium channel, voltage-dependent, gamma subunit 3	
27092	CACNG4	calcium channel, voltage-dependent, gamma subunit 4	
627	BDNF	brain-derived neurotrophic factor	
8398	PLA2G6	phospholipase A2, group VI (cytosolic, calcium-independent)	
81579	PLA2G12A	phospholipase A2, group XIIA	
10912	GADD45G	growth arrest and DNA-damage-inducible, gamma	
4616	GADD45B	growth arrest and DNA-damage-inducible, beta	
1649	DDIT3	DNA-damage-inducible transcript 3	
11184	MAP4K1	mitogen-activated protein kinase kinase kinase kinase 1	
775	CACNA1C	calcium channel, voltage-dependent, L type, alpha 1C subunit	
23542	MAPK8IP2	mitogen-activated protein kinase 8 interacting protein 2	
4763	NF1	neurofibromin 1	
4137	MAPT	microtubule-associated protein tau	
2005	ELK4	ELK4, ETS-domain protein (SRF accessory protein 1)	
5330	PLCB2	phospholipase C, beta 2	
894	CCND2	cyclin D2	
6422	SFRP1	secreted frizzled-related protein 1	
1452	CSNK1A1	casein kinase 1, alpha 1	
85407	NKD1	naked cuticle homolog 1 (Drosophila)	
10725	NFAT5	nuclear factor of activated T-cells 5, tonicity-responsive	
144165	PRICKLE1	prickle homolog 1 (Drosophila)	
57216	VANGL2	vang-like 2 (van gogh, Drosophila)	
43	ACHE	acetylcholinesterase	
114	ADCY8	adenylate cyclase 8 (brain)	
108	ADCY2	adenylate cyclase 2 (brain)	
10142	AKAP9	A kinase (PRKA) anchor protein (yotiao) 9	
6844	VAMP2	vesicle-associated membrane protein 2 (synaptobrevin 2)	
6812	STXBP1	syntaxin binding protein 1	
2560	GABRB1	gamma-aminobutyric acid (GABA) A receptor, beta 1	
2562	GABRB3	gamma-aminobutyric acid (GABA) A receptor, beta 3	
1134	CHRNA1	cholinergic receptor, nicotinic, alpha 1 (muscle)	
2906	GRIN2D	glutamate receptor, ionotropic, N-methyl D-aspartate 2D	
2550	GABBR1	gamma-aminobutyric acid (GABA) B receptor, 1	
4747	NEFL	neurofilament, light polypeptide	
3759	KCNJ2	potassium inwardly-rectifying channel, subfamily J, member 2	
6529	SLC6A1	solute carrier family 6 (neurotransmitter transporter, GABA), member 1	
2752	GLUL	glutamate-ammonia ligase	
2572	GAD2	glutamate decarboxylase 2 (pancreatic islets and brain, 65kDa)	
3745	KCNB1	potassium voltage-gated channel, Shab-related subfamily, member 1	
3758	KCNJ1	potassium inwardly-rectifying channel, subfamily J, member 1	
23416	KCNH3	potassium voltage-gated channel, subfamily H (eag-related), member 3	
3743	KCNA7	potassium voltage-gated channel, shaker-related subfamily, member 7	
3751	KCND2	potassium voltage-gated channel, Shal-related subfamily, member 2	
3757	KCNH2	potassium voltage-gated channel, subfamily H (eag-related), member 2	
9132	KCNQ4	potassium voltage-gated channel, KQT-like subfamily, member 4	
3764	KCNJ8	potassium inwardly-rectifying channel, subfamily J, member 8	
3775	KCNK1	potassium channel, subfamily K, member 1	
3780	KCNN1	potassium intermediate/small conductance calcium-activated channel, subfamily N, member 1	
3781	KCNN2	potassium intermediate/small conductance calcium-activated channel, subfamily N, member 2	
57657	HCN3	hyperpolarization activated cyclic nucleotide-gated potassium channel 3	
610	HCN2	hyperpolarization activated cyclic nucleotide-gated potassium channel 2	
10052	GJC1	gap junction protein, gamma 1, 45kDa	
84694	GJA10	gap junction protein, alpha 10, 62kDa	
10228	STX6	syntaxin 6	
6809	STX3	syntaxin 3	
5308	PITX2	paired-like homeodomain 2	
2597	GAPDH	glyceraldehyde-3-phosphate dehydrogenase	
607	BCL9	B-cell CLL/lymphoma 9	
83999	KREMEN1	kringle containing transmembrane protein 1	
58480	RHOU	ras homolog gene family, member U	
85458	DIXDC1	DIX domain containing 1	
56034	PDGFC	platelet derived growth factor C	
7059	THBS3	thrombospondin 3	
1311	COMP	cartilage oligomeric matrix protein	
1302	COL11A2	collagen, type XI, alpha 2	
5649	RELN	reelin	
5501	PPP1CC	protein phosphatase 1, catalytic subunit, gamma isozyme	
29895	MYLPF	myosin light chain, phosphorylatable, fast skeletal muscle	
25759	SHC2	SHC (Src homology 2 domain containing) transforming protein 2	
53358	SHC3	SHC (Src homology 2 domain containing) transforming protein 3	
2324	FLT4	fms-related tyrosine kinase 4	
6387	CXCL12	chemokine (C-X-C motif) ligand 12	
54437	SEMA5B	sema domain, seven thrombospondin repeats (type 1 and type 1-like), transmembrane domain (TM) and short cytoplasmic domain, (semaphorin) 5B	
22854	NTNG1	netrin G1	
2048	EPHB2	EPH receptor B2	
1946	EFNA5	ephrin-A5	
2047	EPHB1	EPH receptor B1	
1948	EFNB2	ephrin-B2	
2050	EPHB4	EPH receptor B4	
1949	EFNB3	ephrin-B3	
1943	EFNA2	ephrin-A2	
2042	EPHA3	EPH receptor A3	
2044	EPHA5	EPH receptor A5	
2045	EPHA7	EPH receptor A7	
3339	HSPG2	heparan sulfate proteoglycan 2	
50846	DHH	desert hedgehog	
102	ADAM10	ADAM metallopeptidase domain 10	
1435	CSF1	colony stimulating factor 1 (macrophage)	
3481	IGF2	insulin-like growth factor 2 (somatomedin A)	
8646	CHRD	chordin	
3624	INHBA	inhibin, beta A	
268	AMH	anti-Mullerian hormone	
3976	LIF	leukemia inhibitory factor (cholinergic differentiation factor)	
3589	IL11	interleukin 11	
23529	CLCF1	cardiotrophin-like cytokine factor 1	
64806	IL25	interleukin 25	
8744	TNFSF9	tumor necrosis factor (ligand) superfamily, member 9	
10148	EBI3	Epstein-Barr virus induced 3	
222663	SCUBE3	signal peptide, CUB domain, EGF-like 3	
651	BMP3	bone morphogenetic protein 3	
10082	GPC6	glypican 6	
3956	LGALS1	lectin, galactoside-binding, soluble, 1	
8985	PLOD3	procollagen-lysine, 2-oxoglutarate 5-dioxygenase 3	
10536	LEPREL2	leprecan-like 2	
4325	MMP16	matrix metallopeptidase 16 (membrane-inserted)	
7077	TIMP2	TIMP metallopeptidase inhibitor 2	
5033	P4HA1	prolyl 4-hydroxylase, alpha polypeptide I	
8751	ADAM15	ADAM metallopeptidase domain 15	
114904	C1QTNF6	C1q and tumor necrosis factor related protein 6	
653145	ANXA8	annexin A8	
8416	ANXA9	annexin A9	
11173	ADAMTS7	ADAM metallopeptidase with thrombospondin type 1 motif, 7	
23213	SULF1	sulfatase 1	
339366	ADAMTSL5	ADAMTS-like 5	
54507	ADAMTSL4	ADAMTS-like 4	
55959	SULF2	sulfatase 2	
81794	ADAMTS10	ADAM metallopeptidase with thrombospondin type 1 motif, 10	
84695	LOXL3	lysyl oxidase-like 3	
9507	ADAMTS4	ADAM metallopeptidase with thrombospondin type 1 motif, 4	
3491	CYR61	cysteine-rich, angiogenic inducer, 61	
1462	VCAN	versican	
63827	BCAN	brevican	
1305	COL13A1	collagen, type XIII, alpha 1	
1307	COL16A1	collagen, type XVI, alpha 1	
3487	IGFBP4	insulin-like growth factor binding protein 4	
1310	COL19A1	collagen, type XIX, alpha 1	
11117	EMILIN1	elastin microfibril interfacer 1	
25992	SNED1	sushi, nidogen and EGF-like domains 1	
340419	RSPO2	R-spondin 2	
4053	LTBP2	latent transforming growth factor beta binding protein 2	
4054	LTBP3	latent transforming growth factor beta binding protein 3	
4753	NELL2	NEL-like 2 (chicken)	
64856	VWA1	von Willebrand factor A domain containing 1	
8425	LTBP4	latent transforming growth factor beta binding protein 4	
8877	SPHK1	sphingosine kinase 1	
8826	IQGAP1	IQ motif containing GTPase activating protein 1	
10458	BAIAP2	BAI1-associated protein 2	
10093	ARPC4	actin related protein 2/3 complex, subunit 4, 20kDa	
8844	KSR1	kinase suppressor of ras 1	
5795	PTPRJ	protein tyrosine phosphatase, receptor type, J	
1796	DOK1	docking protein 1, 62kDa (downstream of tyrosine kinase 1)	
4035	LRP1	low density lipoprotein receptor-related protein 1	

9351	SLC9A3R2	solute carrier family 9 (sodium/hydrogen exchanger), member 3 regulator 2
8395	PIP5K1B	phosphatidylinositol-4-phosphate 5-kinase, type I, beta
6095	RORA	RAR-related orphan receptor A
9572	NR1D1	nuclear receptor subfamily 1, group D, member 1
8612	PPAP2C	phosphatidic acid phosphatase type 2C
8613	PPAP2B	phosphatidic acid phosphatase type 2B
23446	SLC44A1	solute carrier family 44, member 1
5297	PI4KA	phosphatidylinositol 4-kinase, catalytic, alpha
137964	AGPAT6	1-acylglycerol-3-phosphate O-acyltransferase 6 (lysophosphatidic acid acyltransferase, zeta)
64900	LPIN3	lipin 3
9218	VAPA	VAMP (vesicle-associated membrane protein)-associated protein A, 33kDa
1119	CHKA	choline kinase alpha
10400	PEMT	phosphatidylethanolamine N-methyltransferase
5130	PCYT1A	phosphate cytidylyltransferase 1, choline, alpha
9926	LPGAT1	lysophosphatidylglycerol acyltransferase 1
81490	PTDSS2	phosphatidylserine synthase 2
22901	ARSG	arylsulfatase G
64781	CERK	ceramide kinase
377	ARF3	ADP-ribosylation factor 3
81537	SGPP1	sphingosine-1-phosphate phosphatase 1
8879	SGPL1	sphingosine-1-phosphate lyase 1
10558	SPTLC1	serine palmitoyltransferase, long chain base subunit 1
10825	NEU3	sialidase 3 (membrane sialidase)
56624	ASAH2	N-acylsphingosine amidohydrolase (non-lysosomal ceramidase) 2
9110	MTMR4	myotubularin related protein 4
55361	PI4K2A	phosphatidylinositol 4-kinase type 2 alpha
8898	MTMR2	myotubularin related protein 2
2194	FASN	fatty acid synthase
5034	P4HB	prolyl 4-hydroxylase, beta polypeptide
4862	NPAS2	neuronal PAS domain protein 2
19	ABCA1	ATP-binding cassette, sub-family A (ABC1), member 1
7003	TEAD1	TEA domain family member 1 (SV40 transcriptional enhancer factor)
7004	TEAD4	TEA domain family member 4
211	ALAS1	aminolevulinate, delta-, synthase 1
2222	FDFT1	farnesyl-diphosphate farnesyltransferase 1
3155	HMGCL	3-hydroxymethyl-3-methylglutaryl-CoA lyase
79071	ELOVL6	ELOVL fatty acid elongase 6
3931	LCAT	lecithin-cholesterol acyltransferase
344	APOC2	apolipoprotein C-II
81693	AMN	amnionless homolog (mouse)
9415	FADS2	fatty acid desaturase 2
4047	LSS	lanosterol synthase (2,3-oxidosqualene-lanosterol cyclase)
3422	IDI1	isopentenyl-diphosphate delta isomerase 1
3292	HSD17B1	hydroxysteroid (17-beta) dehydrogenase 1
2619	GAS1	growth arrest-specific 1
7546	ZIC2	Zic family member 2
7074	TIAM1	T-cell lymphoma invasion and metastasis 1
8394	PIP5K1A	phosphatidylinositol-4-phosphate 5-kinase, type I, alpha
8874	ARHGEF7	Rho guanine nucleotide exchange factor (GEF) 7
623	BDKRB1	bradykinin receptor B1
79837	PIP4K2C	phosphatidylinositol-5-phosphate 4-kinase, type II, gamma
10788	IQGAP2	IQ motif containing GTPase activating protein 2
5584	PRKCI	protein kinase C, iota
8997	KALRN	kalirin, RhoGEF kinase
9181	ARHGEF2	Rho/Rac guanine nucleotide exchange factor (GEF) 2
57142	RTN4	reticulon 4
10488	CREB3	cAMP responsive element binding protein 3
10254	STAM2	signal transducing adaptor molecule (SH3 domain and ITAM motif) 2
11140	CDC37	cell division cycle 37 homolog (S. cerevisiae)
26018	LRIG1	leucine-rich repeats and immunoglobulin-like domains 1
8379	MAD1L1	MAD1 mitotic arrest deficient-like 1 (yeast)
9184	BUB3	budding uninhibited by benzimidazoles 3 homolog (yeast)
10274	STAG1	stromal antigen 1
4174	MCM5	minichromosome maintenance complex component 5
8317	CDC7	cell division cycle 7 homolog (S. cerevisiae)
9088	PKMYT1	protein kinase, membrane associated tyrosine/threonine 1
151648	SGOL1	shugoshin-like 1 (S. pombe)
6232	RPS27	ribosomal protein S27
11130	ZWINT	ZW10 interactor
25909	AHCTF1	AT hook containing transcription factor 1
80776	B9D2	B9 protein domain 2
57804	POLD4	polymerase (DNA-directed), delta 4
7084	TK2	thymidine kinase 2, mitochondrial
22897	CEP164	centrosomal protein 164kDa
10806	SDCCAG8	serologically defined colon cancer antigen 8
80321	CEP70	centrosomal protein 70kDa
7538	ZFP36	zinc finger protein 36, C3H type, homolog (mouse)
1124	CHN2	chimerin (chimaerin) 2
378	ARF4	ADP-ribosylation factor 4
26127	FGFR1OP2	FGFR1 oncogene partner 2
56288	PARD3	par-3 partitioning defective 3 homolog (C. elegans)
23327	NEDD4L	neural precursor cell expressed, developmentally down-regulated 4-like
1601	DAB2	disabled homolog 2, mitogen-responsive phosphoprotein (Drosophila)
2065	ERBB3	v-erb-b2 erythroblastic leukemia viral oncogene homolog 3 (avian)
382	ARF6	ADP-ribosylation factor 6
2869	GRK5	G protein-coupled receptor kinase 5
157	ADRBK2	adrenergic, beta, receptor kinase 2
2868	GRK4	G protein-coupled receptor kinase 4
7037	TFRC	transferrin receptor (p90, CD71)
84313	VPS25	vacuolar protein sorting 25 homolog (S. cerevisiae)
9230	RAB11B	RAB11B, member RAS oncogene family
23362	PSD3	pleckstrin and Sec7 domain containing 3
80230	RUFY1	RUN and FYVE domain containing 1
9135	RABEP1	rabaptin, RAB GTPase binding effector protein 1
545	ATR	ataxia telangiectasia and Rad3 related
10459	MAD2L2	MAD2 mitotic arrest deficient-like 2 (yeast)
9700	ESPL1	extra spindle pole bodies homolog 1 (S. cerevisiae)
7272	TTK	TTK protein kinase
10580	SORBS1	sorbin and SH3 domain containing 1
5787	PTPRB	protein tyrosine phosphatase, receptor type, B
2212	FCGR2A	Fc fragment of IgG, low affinity IIa, receptor (CD32)
672	BRCA1	breast cancer 1, early onset
10277	UBE4B	ubiquitination factor E4B
27339	PRPF19	PRP19/PSO4 pre-mRNA processing factor 19 homolog (S. cerevisiae)
26091	HERC4	hect domain and RLD 4
4281	MID1	midline 1 (Opitz/BBB syndrome)
57448	BIRC6	baculoviral IAP repeat containing 6
8925	HERC1	hect (homologous to the E6-AP (UBE3A) carboxyl terminus) domain and RCC1 (CHC1)-like domain (RLD) 1
9586	CREB5	cAMP responsive element binding protein 5
4000	LMNA	lamin A/C
9765	ZFYVE16	zinc finger, FYVE domain containing 16
6498	SKIL	SKI-like oncogene
7049	TGFBR3	transforming growth factor, beta receptor III
25805	BAMBI	BMP and activin membrane-bound inhibitor homolog (Xenopus laevis)
4830	NME1	non-metastatic cells 1, protein (NM23A) expressed in
79872	CBLL1	Cas-Br-M (murine) ecotropic retroviral transforming sequence-like 1
9970	NR1I3	nuclear receptor subfamily 1, group I, member 3
2100	ESR2	estrogen receptor 2 (ER beta)
4306	NR3C2	nuclear receptor subfamily 3, group C, member 2
10062	NR1H3	nuclear receptor subfamily 1, group H, member 3
7067	THRA	thyroid hormone receptor, alpha
5916	RARG	retinoic acid receptor, gamma
8013	NR4A3	nuclear receptor subfamily 4, group A, member 3
10002	NR2E3	nuclear receptor subfamily 2, group E, member 3
2516	NR5A1	nuclear receptor subfamily 5, group A, member 1
4929	NR4A2	nuclear receptor subfamily 4, group A, member 2
6096	RORB	RAR-related orphan receptor B
6667	SP1	Sp1 transcription factor
28996	HIPK2	homeodomain interacting protein kinase 2
2944	GSTM1	glutathione S-transferase mu 1
463	ZFHX3	zinc finger homeobox 3
3726	JUNB	jun B proto-oncogene
10987	COPS5	COP9 constitutive photomorphogenic homolog subunit 5 (Arabidopsis)
2355	FOSL2	FOS-like antigen 2
9988	DMTF1	cyclin D binding myb-like transcription factor 1
2354	FOSB	FBJ murine osteosarcoma viral oncogene homolog B
4082	MARCKS	myristoylated alanine-rich protein kinase C substrate
3670	ISL1	ISL LIM homeobox 1
1045	CDX2	caudal type homeobox 2
51085	MLXIPL	MLX interacting protein-like
6945	MLX	MAX-like protein X
3708	ITPR1	inositol 1,4,5-trisphosphate receptor, type 1
6262	RYR2	ryanodine receptor 2 (cardiac)
6442	SGCA	sarcoglycan, alpha (50kDa dystrophin-associated glycoprotein)
7168	TPM1	tropomyosin 1 (alpha)
6908	TBP	TATA box binding protein
5077	PAX3	paired box 3
11016	ATF7	activating transcription factor 7
6046	BRD2	bromodomain containing 2
3398	ID2	inhibitor of DNA binding 2, dominant negative helix-loop-helix protein
9241	NOG	noggin
10803	CCR9	chemokine (C-C motif) receptor 9
2826	CCR10	chemokine (C-C motif) receptor 10
1813	DRD2	dopamine receptor D2
65108	MARCKSL1	MARCKS-like 1
2319	FLOT2	flotillin 2
133	ADM	adrenomedullin
10266	RAMP2	receptor (G protein-coupled) activity modifying protein 2
2691	GHRH	growth hormone releasing hormone
27113	BBC3	BCL2 binding component 3
578	BAK1	BCL2-antagonist/killer 1
274	BIN1	bridging integrator 1
7490	WT1	Wilms tumor 1
54894	RNF43	ring finger protein 43
200734	SPRED2	sprouty-related, EVH1 domain containing 2
26228	STAP1	signal transducing adaptor family member 1
201163	FLCN	folliculin
10381	TUBB3	tubulin, beta 3 class III
51807	TUBA8	tubulin, alpha 8
4916	NTRK3	neurotrophic tyrosine kinase, receptor, type 3
3162	HMOX1	heme oxygenase (decycling) 1
5236	PGM1	phosphoglucomutase 1
5211	PFKL	phosphofructokinase, liver
768	CA9	carbonic anhydrase IX
64283	RGNEF	190 kDa guanine nucleotide exchange factor
4953	ODC1	ornithine decarboxylase 1
6470	SHMT1	serine hydroxymethyltransferase 1 (soluble)

586	BCAT1	branched chain amino-acid transaminase 1, cytosolic
4831	NME2	non-metastatic cells 2, protein (NM23B) expressed in
3308	HSPA4	heat shock 70kDa protein 4
10935	PRDX3	peroxiredoxin 3
648	BMI1	BMI1 polycomb ring finger oncogene
8886	DDX18	DEAD (Asp-Glu-Ala-Asp) box polypeptide 18
92140	MTDH	metadherin
4836	NMT1	N-myristoyltransferase 1
90427	BMF	Bcl2 modifying factor
22985	ACIN1	apoptotic chromatin condensation inducer 1
2620	GAS2	growth arrest-specific 2
3005	H1F0	H1 histone family, member 0
3006	HIST1H1C	histone cluster 1, H1c
2538	G6PC	glucose-6-phosphatase, catalytic subunit
3178	HNRNPA1	heterogeneous nuclear ribonucleoprotein A1
3092	HIP1	huntingtin interacting protein 1
8204	NRIP1	nuclear receptor interacting protein 1
26524	LATS2	LATS, large tumor suppressor, homolog 2 (Drosophila)
23598	PATZ1	POZ (BTB) and AT hook containing zinc finger 1
24149	ZNF318	zinc finger protein 318
57178	ZMIZ1	zinc finger, MIZ-type containing 1
7041	TGFB1I1	transforming growth factor beta 1 induced transcript 1
6515	SLC2A3	solute carrier family 2 (facilitated glucose transporter), member 3
529	ATP6V1E1	ATPase, H+ transporting, lysosomal 31kDa, V1 subunit E1
534	ATP6V1G2	ATPase, H+ transporting, lysosomal 13kDa, V1 subunit G2
6533	SLC6A6	solute carrier family 6 (neurotransmitter transporter, taurine), member 6
6570	SLC18A1	solute carrier family 18 (vesicular monoamine), member 1
6536	SLC6A9	solute carrier family 6 (neurotransmitter transporter, glycine), member 9
64849	SLC13A3	solute carrier family 13 (sodium-dependent dicarboxylate transporter), member 3
81031	SLC2A10	solute carrier family 2 (facilitated glucose transporter), member 10
7781	SLC30A3	solute carrier family 30 (zinc transporter), member 3
7922	SLC39A7	solute carrier family 39 (zinc transporter), member 7
6524	SLC5A2	solute carrier family 5 (sodium/glucose cotransporter), member 2
6558	SLC12A2	solute carrier family 12 (sodium/potassium/chloride transporters), member 2
6509	SLC1A4	solute carrier family 1 (glutamate/neutral amino acid transporter), member 4
23315	SLC9A8	solute carrier family 9 (sodium/hydrogen exchanger), member 8
57468	SLC12A5	solute carrier family 12 (potassium/chloride transporter), member 5
6521	SLC4A1	solute carrier family 4, anion exchanger, member 1 (erythrocyte membrane protein band 3, Diego blood group)
9497	SLC4A7	solute carrier family 4, sodium bicarbonate cotransporter, member 7
477	ATP1A2	ATPase, Na+/K+ transporting, alpha 2 polypeptide
10396	ATP8A1	ATPase, aminophospholipid transporter (APLT), class I, type 8A, member 1
85440	DOCK7	dedicator of cytokinesis 7
6487	ST3GAL3	ST3 beta-galactoside alpha-2,3-sialyltransferase 3
3955	LFNG	LFNG O-fucosylpeptide 3-beta-N-acetylglucosaminyltransferase
4242	MFNG	MFNG O-fucosylpeptide 3-beta-N-acetylglucosaminyltransferase
5986	RFNG	RFNG O-fucosylpeptide 3-beta-N-acetylglucosaminyltransferase
192669	EIF2C3	eukaryotic translation initiation factor 2C, 3
26523	EIF2C1	eukaryotic translation initiation factor 2C, 1
23112	TNRC6B	trinucleotide repeat containing 6B
4343	MOV10	Mov10, Moloney leukemia virus 10, homolog (mouse)
23509	POFUT1	protein O-fucosyltransferase 1
8703	B4GALT3	UDP-Gal:betaGlcNAc beta 1,4- galactosyltransferase, polypeptide 3
10678	B3GNT2	UDP-GlcNAc:betaGal beta-1,3-N-acetylglucosaminyltransferase 2
6482	ST3GAL1	ST3 beta-galactoside alpha-2,3-sialyltransferase 1
2799	GNS	glucosamine (N-acetyl)-6-sulfatase
3938	LCT	lactase
7167	TPI1	triosephosphate isomerase 1
2582	GALE	UDP-galactose-4-epimerase
2584	GALK1	galactokinase 1
2026	ENO2	enolase 2 (gamma, neuronal)
5208	PFKFB2	6-phosphofructo-2-kinase/fructose-2,6-biphosphatase 2
9953	HS3ST3B1	heparan sulfate (glucosamine) 3-O-sulfotransferase 3B1
9653	HS2ST1	heparan sulfate 2-O-sulfotransferase 1
9957	HS3ST1	heparan sulfate (glucosamine) 3-O-sulfotransferase 1
6476	SI	sucrase-isomaltase (alpha-glucosidase)
3269	HRH1	histamine receptor H1
3362	HTR6	5-hydroxytryptamine (serotonin) receptor 6
904	CCNT1	cyclin T1
11168	PSIP1	PC4 and SFRS1 interacting protein 1
6749	SSRP1	structure specific recognition protein 1
5430	POLR2A	polymerase (RNA) II (DNA directed) polypeptide A, 220kDa
2968	GTF2H4	general transcription factor IIH, polypeptide 4, 52kDa
2957	GTF2A1	general transcription factor IIA, 1, 19/37kDa
3601	IL15RA	interleukin 15 receptor, alpha
1271	CNTFR	ciliary neurotrophic factor receptor
25998	IBTK	inhibitor of Bruton agammaglobulinemia tyrosine kinase
9467	SH3BP5	SH3-domain binding protein 5 (BTK-associated)
604	BCL6	B-cell CLL/lymphoma 6
7070	THY1	Thy-1 cell surface antigen
2011	MARK2	MAP/microtubule affinity-regulating kinase 2
200186	CRTC2	CREB regulated transcription coactivator 2
2118	ETV4	ets variant 4
57787	MARK4	MAP/microtubule affinity-regulating kinase 4
84446	BRSK1	BR serine/threonine kinase 1
27347	STK39	serine threonine kinase 39
7182	NR2C2	nuclear receptor subfamily 2, group C, member 2
2520	GAST	gastrin
2150	F2RL1	coagulation factor II (thrombin) receptor-like 1
84634	KISS1R	KISS1 receptor
7200	TRH	thyrotropin-releasing hormone
6753	SSTR3	somatostatin receptor 3
8811	GALR2	galanin receptor 2
134	ADORA1	adenosine A1 receptor
2918	GRM8	glutamate receptor, metabotropic 8
27115	PDE7B	phosphodiesterase 7B
84152	PPP1R1B	protein phosphatase 1, regulatory (inhibitor) subunit 1B
6000	RGS7	regulator of G-protein signaling 7
1003	CDH5	cadherin 5, type 2 (vascular endothelium)
9748	SLK	STE20-like kinase
1394	CRHR1	corticotropin releasing hormone receptor 1
2895	GRID2	glutamate receptor, ionotropic, delta 2
182	JAG1	jagged 1
6184	RPN1	ribophorin I
6185	RPN2	ribophorin II
6480	ST6GAL1	ST6 beta-galactosamide alpha-2,6-sialyltranferase 1
4249	MGAT5	mannosyl (alpha-1,6-)-glycoprotein beta-1,6-N-acetyl-glucosaminyltransferase
10195	ALG3	asparagine-linked glycosylation 3, alpha-1,3- mannosyltransferase homolog (S. cerevisiae)
4121	MAN1A1	mannosidase, alpha, class 1A, member 1
3703	STT3A	STT3, subunit of the oligosaccharyltransferase complex, homolog A (S. cerevisiae)
7991	TUSC3	tumor suppressor candidate 3
4351	MPI	mannose phosphate isomerase
9945	GFPT2	glutamine-fructose-6-phosphate transaminase 2
64841	GNPNAT1	glucosamine-phosphate N-acetyltransferase 1
2290	FOXG1	forkhead box G1
54512	EXOSC4	exosome component 4
8125	ANP32A	acidic (leucine-rich) nuclear phosphoprotein 32 family, member A
23671	TMEFF2	transmembrane protein with EGF-like and two follistatin-like domains 2
24146	CLDN15	claudin 15
64398	MPP5	membrane protein, palmitoylated 5 (MAGUK p55 subfamily member 5)
1006	CDH8	cadherin 8, type 2
1008	CDH10	cadherin 10, type 2 (T2-cadherin)
7016	TESK1	testis-specific kinase 1
4868	NPHS1	nephrosis 1, congenital, Finnish type (nephrin)
81617	CAB39L	calcium binding protein 39-like
10642	IGF2BP1	insulin-like growth factor 2 mRNA binding protein 1
9451	EIF2AK3	eukaryotic translation initiation factor 2-alpha kinase 3
2081	ERN1	endoplasmic reticulum to nucleus signaling 1
2137	EXTL3	exostoses (multiple)-like 3
11015	KDELR3	KDEL (Lys-Asp-Glu-Leu) endoplasmic reticulum protein retention receptor 3
10525	HYOU1	hypoxia up-regulated 1
10643	IGF2BP3	insulin-like growth factor 2 mRNA binding protein 3
667	DST	dystonin
7022	TFAP2C	transcription factor AP-2 gamma (activating enhancer binding protein 2 gamma)
137075	CLDN23	claudin 23
260425	MAGI3	membrane associated guanylate kinase, WW and PDZ domain containing 3
2037	EPB41L2	erythrocyte membrane protein band 4.1-like 2
5872	RAB13	RAB13, member RAS oncogene family
9223	MAGI1	membrane associated guanylate kinase, WW and PDZ domain containing 1
10921	RNPS1	RNA binding protein S1, serine-rich domain
57819	LSM2	LSM2 homolog, U6 small nuclear RNA associated (S. cerevisiae)
6628	SNRPB	small nuclear ribonucleoprotein polypeptides B and B1
6634	SNRPD3	small nuclear ribonucleoprotein D3 polypeptide 18kDa
8106	PABPN1	poly(A) binding protein, nuclear 1
10250	SRRM1	serine/arginine repetitive matrix 1
10992	SF3B2	splicing factor 3b, subunit 2, 145kDa
9343	EFTUD2	elongation factor Tu GTP binding domain containing 2
10946	SF3A3	splicing factor 3a, subunit 3, 60kDa
10421	CD2BP2	CD2 (cytoplasmic tail) binding protein 2
2521	FUS	fused in sarcoma
3181	HNRNPA2B1	heterogeneous nuclear ribonucleoprotein A2/B1
3191	HNRNPL	heterogeneous nuclear ribonucleoprotein L
7884	SLBP	stem-loop binding protein
339	APOBEC1	apolipoprotein B mRNA editing enzyme, catalytic polypeptide 1
7284	TUFM	Tu translation elongation factor, mitochondrial
5376	PMP22	peripheral myelin protein 22
3090	HIC1	hypermethylated in cancer 1
599	BCL2L2	BCL2-like 2
639	PRDM1	PR domain containing 1, with ZNF domain
3921	RPSA	ribosomal protein SA
6129	RPL7	ribosomal protein L7
6136	RPL12	ribosomal protein L12
6141	RPL18	ribosomal protein L18
6168	RPL37A	ribosomal protein L37a
6171	RPL41	ribosomal protein L41
2107	ETF1	eukaryotic translation termination factor 1
23381	SMG5	smg-5 homolog, nonsense mediated mRNA decay factor (C. elegans)
4850	CNOT4	CCR4-NOT transcription complex, subunit 4
221178	SPATA13	spermatogenesis associated 13
29071	C1GALT1C1	C1GALT1-specific chaperone 1
23556	PIGN	phosphatidylinositol glycan anchor biosynthesis, class N
8733	GPAA1	glycosylphosphatidylinositol anchor attachment protein 1 homolog (yeast)
10452	TOMM40	translocase of outer mitochondrial membrane 40 homolog (yeast)
7203	CCT3	chaperonin containing TCP1, subunit 3 (gamma)
63971	KIF13A	kinesin family member 13A
50	ACO2	aconitase 2, mitochondrial
3313	HSPA9	heat shock 70kDa protein 9 (mortalin)
6730	SRP68	signal recognition particle 68kDa

8667	EIF3H	eukaryotic translation initiation factor 3, subunit H
10063	COX17	COX17 cytochrome c oxidase assembly homolog (S. cerevisiae)
1351	COX8A	cytochrome c oxidase subunit VIIIA (ubiquitous)
4695	NDUFA2	NADH dehydrogenase (ubiquinone) 1 alpha subcomplex, 2, 8kDa
4704	NDUFA9	NADH dehydrogenase (ubiquinone) 1 alpha subcomplex, 9, 39kDa
1742	DLG4	discs, large homolog 4 (Drosophila)
146754	DNAH2	dynein, axonemal, heavy chain 2
7802	DNALI1	dynein, axonemal, light intermediate chain 1
30009	TBX21	T-box 21
51111	SUV420H1	suppressor of variegation 4-20 homolog 1 (Drosophila)
2289	FKBP5	FK506 binding protein 5
55504	TNFRSF19	tumor necrosis factor receptor superfamily, member 19
23085	ERC1	ELKS/RAB6-interacting/CAST family member 1
7335	UBE2V1	ubiquitin-conjugating enzyme E2 variant 1
4744	NEFH	neurofilament, heavy polypeptide
5630	PRPH	peripherin
79139	DERL1	Der1-like domain family, member 1
1628	DBP	D site of albumin promoter (albumin D-box) binding protein
56938	ARNTL2	aryl hydrocarbon receptor nuclear translocator-like 2
2665	GDI2	GDP dissociation inhibitor 2
257106	ARHGAP30	Rho GTPase activating protein 30
83478	ARHGAP24	Rho GTPase activating protein 24
9411	ARHGAP29	Rho GTPase activating protein 29
93185	IGSF8	immunoglobulin superfamily, member 8
196403	DTX3	deltex homolog 3 (Drosophila)
1960	EGR3	early growth response 3
2635	GBP3	guanylate binding protein 3
5796	PTPRK	protein tyrosine phosphatase, receptor type, K
1600	DAB1	disabled homolog 1 (Drosophila)
7391	USF1	upstream transcription factor 1
55816	DOK5	docking protein 5
9260	PDLIM7	PDZ and LIM domain 7 (enigma)
51582	AZIN1	antizyme inhibitor 1
22809	ATF5	activating transcription factor 5
8073	PTP4A2	protein tyrosine phosphatase type IVA, member 2
6801	STRN	striatin, calmodulin binding protein
4311	MME	membrane metallo-endopeptidase
23621	BACE1	beta-site APP-cleaving enzyme 1
8320	EOMES	eomesodermin
4942	OAT	ornithine aminotransferase
51074	APIP	APAF1 interacting protein
58478	ENOPH1	enolase-phosphatase 1
7263	TST	thiosulfate sulfurtransferase (rhodanese)
549	AUH	AU RNA binding protein/enoyl-CoA hydratase
1644	DDC	dopa decarboxylase (aromatic L-amino acid decarboxylase)
5092	PCBD1	pterin-4 alpha-carbinolamine dehydratase/dimerization cofactor of hepatocyte nuclear factor 1 alpha
3778	KCNMA1	potassium large conductance calcium-activated channel, subfamily M, alpha member 1
6418	SET	SET nuclear oncogene
135112	NCOA7	nuclear receptor coactivator 7
2120	ETV6	ets variant 6
84501	SPIRE2	spire homolog 2 (Drosophila)
55902	ACSS2	acyl-CoA synthetase short-chain family member 2
3206	HOXA10	homeobox A10
5049	PAFAH1B2	platelet-activating factor acetylhydrolase 1b, catalytic subunit 2 (30kDa)
5050	PAFAH1B3	platelet-activating factor acetylhydrolase 1b, catalytic subunit 3 (29kDa)
1827	RCAN1	regulator of calcineurin 1
429	ASCL1	achaete-scute complex homolog 1 (Drosophila)
2175	FANCA	Fanconi anemia, complementation group A
2130	EWSR1	Ewing sarcoma breakpoint region 1
7462	LAT2	linker for activation of T cells family, member 2
27131	SNX5	sorting nexin 5
7163	TPD52	tumor protein D52
8729	GBF1	golgi brefeldin A resistant guanine nucleotide exchange factor 1
2706	GJB2	gap junction protein, beta 2, 26kDa
51728	POLR3K	polymerase (RNA) III (DNA directed) polypeptide K, 12.3 kDa
84265	POLR3GL	polymerase (RNA) III (DNA directed) polypeptide G (32kD)-like
4781	NFIB	nuclear factor I/B
10302	SNAPC5	small nuclear RNA activating complex, polypeptide 5, 19kDa
6618	SNAPC2	small nuclear RNA activating complex, polypeptide 2, 45kDa
284119	PTRF	polymerase I and transcript release factor
11335	CBX3	chromobox homolog 3
9310	ZNF235	zinc finger protein 235
8861	LDB1	LIM domain binding 1
2119	ETV5	ets variant 5
7351	UCP2	uncoupling protein 2 (mitochondrial, proton carrier)
5147	PDE6D	phosphodiesterase 6D, cGMP-specific, rod, delta
9615	GDA	guanine deaminase
5141	PDE4A	phosphodiesterase 4A, cAMP-specific
58497	PRUNE	prune homolog (Drosophila)
10380	BPNT1	3'(2'), 5'-bisphosphate nucleotidase 1
80854	SETD7	SET domain containing (lysine methyltransferase) 7
10690	FUT9	fucosyltransferase 9 (alpha (1,3) fucosyltransferase)
2651	GCNT2	glucosaminyl (N-acetyl) transferase 2, I-branching enzyme (I blood group)
6484	ST3GAL4	ST3 beta-galactoside alpha-2,3-sialyltransferase 4
252983	STXBP4	syntaxin binding protein 4
1876	E2F6	E2F transcription factor 6
3945	LDHB	lactate dehydrogenase B
1737	DLAT	dihydrolipoamide S-acetyltransferase
8625	RFXANK	regulatory factor X-associated ankyrin-containing protein
6892	TAPBP	TAP binding protein (tapasin)
57863	CADM3	cell adhesion molecule 3
9378	NRXN1	neurexin 1
7156	TOP3A	topoisomerase (DNA) III alpha
80198	MUS81	MUS81 endonuclease homolog (S. cerevisiae)
8438	RAD54L	RAD54-like (S. cerevisiae)
8940	TOP3B	topoisomerase (DNA) III beta
2305	FOXM1	forkhead box M1
53615	MBD3	methyl-CpG binding domain protein 3
1108	CHD4	chromodomain helicase DNA binding protein 4
23409	SIRT4	sirtuin 4
57459	GATAD2B	GATA zinc finger domain containing 2B
9219	MTA2	metastasis associated 1 family, member 2
112483	SAT2	spermidine/spermine N1-acetyltransferase family member 2
29920	PYCR2	pyrroline-5-carboxylate reductase family, member 2
9562	MINPP1	multiple inositol-polyphosphate phosphatase 1
8930	MBD4	methyl-CpG binding domain protein 4
3980	LIG3	ligase III, DNA, ATP-dependent
5429	POLH	polymerase (DNA directed), eta
5980	REV3L	REV3-like, catalytic subunit of DNA polymerase zeta (yeast)
6319	SCD	stearoyl-CoA desaturase (delta-9-desaturase)
2170	FABP3	fatty acid binding protein 3, muscle and heart (mammary-derived growth inhibitor)
7316	UBC	ubiquitin C
10020	GNE	glucosamine (UDP-N-acetyl)-2-epimerase/N-acetylmannosamine kinase
4774	NFIA	nuclear factor I/A
9627	SNCAIP	synuclein, alpha interacting protein
51399	TRAPPC4	trafficking protein particle complex 4
3145	HMBS	hydroxymethylbilane synthase
5498	PPOX	protoporphyrinogen oxidase
951	CD37	CD37 molecule
558	AXL	AXL receptor tyrosine kinase
4661	MYT1	myelin transcription factor 1
53	ACP2	acid phosphatase 2, lysosomal
10947	AP3M2	adaptor-related protein complex 3, mu 2 subunit
11154	AP4S1	adaptor-related protein complex 4, sigma 1 subunit
130340	AP1S3	adaptor-related protein complex 1, sigma 3 subunit
26088	GGA1	golgi-associated, gamma adaptin ear containing, ARF binding protein 1
7805	LAPTM5	lysosomal protein transmembrane 5
9179	AP4M1	adaptor-related protein complex 4, mu 1 subunit
9741	LAPTM4A	lysosomal protein transmembrane 4 alpha
7402	UTRN	utrophin
2969	GTF2I	general transcription factor IIi
6879	TAF7	TAF7 RNA polymerase II, TATA box binding protein (TBP)-associated factor, 55kDa
9569	GTF2IRD1	GTF2I repeat domain containing 1
8993	PGLYRP1	peptidoglycan recognition protein 1
2135	EXTL2	exostoses (multiple)-like 2
10791	VAMP5	vesicle-associated membrane protein 5 (myobrevin)
53407	STX18	syntaxin 18
9342	SNAP29	synaptosomal-associated protein, 29kDa
151903	CCDC12	coiled-coil domain containing 12
55119	PRPF38B	PRP38 pre-mRNA processing factor 38 (yeast) domain containing B
8567	MADD	MAP-kinase activating death domain
84823	LMNB2	lamin B2
6909	TBX2	T-box 2
181	AGRP	agouti related protein homolog (mouse)
6646	SOAT1	sterol O-acyltransferase 1
57504	MTA3	metastasis associated 1 family, member 3
30851	TAX1BP3	Tax1 (human T-cell leukemia virus type I) binding protein 3
6462	SHBG	sex hormone-binding globulin
10460	TACC3	transforming, acidic coiled-coil containing protein 3
23424	TDRD7	tudor domain containing 7
9962	SLC23A2	solute carrier family 23 (nucleobase transporters), member 2
55215	FANCI	Fanconi anemia, complementation group I
6916	TBXAS1	thromboxane A synthase 1 (platelet)
4635	MYL4	myosin, light chain 4, alkali; atrial, embryonic
9388	LIPG	lipase, endothelial
7407	VARS	valyl-tRNA synthetase
16	AARS	alanyl-tRNA synthetase
2193	FARSA	phenylalanyl-tRNA synthetase, alpha subunit
833	CARS	cysteinyl-tRNA synthetase
58528	RRAGD	Ras-related GTP binding D
8536	CAMK1	calcium/calmodulin-dependent protein kinase I
9755	TBKBP1	TBK1 binding protein 1
239	ALOX12	arachidonate 12-lipoxygenase
4048	LTA4H	leukotriene A4 hydrolase
246175	CNOT6L	CCR4-NOT transcription complex, subunit 6-like
5826	ABCD4	ATP-binding cassette, sub-family D (ALD), member 4
22	ABCB7	ATP-binding cassette, sub-family B (MDR/TAP), member 7
89845	ABCC10	ATP-binding cassette, sub-family C (CFTR/MRP), member 10
51268	PIPOX	pipecolic acid oxidase
5190	PEX6	peroxisomal biogenesis factor 6
5194	PEX13	peroxisomal biogenesis factor 13
9409	PEX16	peroxisomal biogenesis factor 16
2117	ETV3	ets variant 3
7378	UPP1	uridine phosphorylase 1
1789	DNMT3B	DNA (cytosine-5-)-methyltransferase 3 beta
79178	THTPA	thiamine triphosphatase
3301	DNAJA1	DnaJ (Hsp40) homolog, subfamily A, member 1

654817	NCF1C	neutrophil cytosolic factor 1C pseudogene
11231	SEC63	SEC63 homolog (S. cerevisiae)
2739	GLO1	glyoxalase I
55766	H2AFJ	H2A histone family, member J
9563	H6PD	hexose-6-phosphate dehydrogenase (glucose 1-dehydrogenase)
6817	SULT1A1	sulfotransferase family, cytosolic, 1A, phenol-preferring, member 1
8869	ST3GAL5	ST3 beta-galactoside alpha-2,3-sialyltransferase 5
79947	DHDDS	dehydrodolichyl diphosphate synthase
120892	LRRK2	leucine-rich repeat kinase 2
27429	HTRA2	HtrA serine peptidase 2
283209	PGM2L1	phosphoglucomutase 2-like 1
602	BCL3	B-cell CLL/lymphoma 3
3543	IGLL1	immunoglobulin lambda-like polypeptide 1
11337	GABARAP	GABA(A) receptor-associated protein
11345	GABARAPL2	GABA(A) receptor-associated protein-like 2
64422	ATG3	ATG3 autophagy related 3 homolog (S. cerevisiae)
4439	MSH5	mutS homolog 5 (E. coli)
51070	NOSIP	nitric oxide synthase interacting protein
2941	GSTA4	glutathione S-transferase alpha 4
5886	RAD23A	RAD23 homolog A (S. cerevisiae)
131965	METTL6	methyltransferase like 6
157506	RDH10	retinol dehydrogenase 10 (all-trans)
54840	APTX	aprataxin
7155	TOP2B	topoisomerase (DNA) II beta 180kDa
100271849	MEF2B	myocyte enhancer factor 2B
100505576	LOC100505576	uncharacterized LOC100505576
10061	ABCF2	ATP-binding cassette, sub-family F (GCN20), member 2
10067	SCAMP3	secretory carrier membrane protein 3
10068	IL18BP	interleukin 18 binding protein
10114	HIPK3	homeodomain interacting protein kinase 3
10180	RBM6	RNA binding motif protein 6
10184	LHFPL2	lipoma HMGIC fusion partner-like 2
10198	MPHOSPH9	M-phase phosphoprotein 9
10205	MPZL2	myelin protein zero-like 2
10212	DDX39A	DEAD (Asp-Glu-Ala-Asp) box polypeptide 39A
10237	SLC35B1	solute carrier family 35, member B1
10290	SPEG	SPEG complex locus
10313	RTN3	reticulon 3
10363	HMG20A	high mobility group 20A
10406	WFDC2	WAP four-disulfide core domain 2
10420	TESK2	testis-specific kinase 2
10432	RBM14	RNA binding motif protein 14
10456	HAX1	HCLS1 associated protein X-1
10467	ZNHIT1	zinc finger, HIT-type containing 1
10526	IPO8	importin 8
10529	NEBL	nebulette
10586	MAB21L2	mab-21-like 2 (C. elegans)
10607	TBL3	transducin (beta)-like 3
10615	SPAG5	sperm associated antigen 5
10620	ARID3B	AT rich interactive domain 3B (BRIGHT-like)
10635	RAD51AP1	RAD51 associated protein 1
10657	KHDRBS1	KH domain containing, RNA binding, signal transduction associated 1
10664	CTCF	CCCTC-binding factor (zinc finger protein)
10693	CCT6B	chaperonin containing TCP1, subunit 6B (zeta 2)
10730	YME1L1	YME1-like 1 (S. cerevisiae)
10743	RAI1	retinoic acid induced 1
10749	KIF1C	kinesin family member 1C
10763	NES	nestin
10771	ZMYND11	zinc finger, MYND-type containing 11
10809	STARD10	StAR-related lipid transfer (START) domain containing 10
10900	RUNDC3A	RUN domain containing 3A
10902	BRD8	bromodomain containing 8
10951	CBX1	chromobox homolog 1
10955	SERINC3	serine incorporator 3
10962	MLLT11	myeloid/lymphoid or mixed-lineage leukemia (trithorax homolog, Drosophila); translocated to, 11
10980	COPS6	COP9 constitutive photomorphogenic homolog subunit 6 (Arabidopsis)
10982	MAPRE2	microtubule-associated protein, RP/EB family, member 2
11011	TLK2	tousled-like kinase 2
11030	RBPMS	RNA binding protein with multiple splicing
1114	CHGB	chromogranin B (secretogranin 1)
11142	PKIG	protein kinase (cAMP-dependent, catalytic) inhibitor gamma
11163	NUDT4	nudix (nucleoside diphosphate linked moiety X)-type motif 4
11217	AKAP2	A kinase (PRKA) anchor protein 2
112616	CMTM7	CKLF-like MARVEL transmembrane domain containing 7
11269	DDX19B	DEAD (Asp-Glu-Ala-As) box polypeptide 19B
113130	CDCA5	cell division cycle associated 5
113246	C12orf57	chromosome 12 open reading frame 57
113277	TMEM106A	transmembrane protein 106A
11334	TUSC2	tumor suppressor candidate 2
114757	CYGB	cytoglobin
114804	RNF157	ring finger protein 157
114883	OSBPL9	oxysterol binding protein-like 9
114990	VASN	vasorin
1153	CIRBP	cold inducible RNA binding protein
116039	OSR2	odd-skipped related 2 (Drosophila)
116238	TLCD1	TLC domain containing 1
116369	SLC26A8	solute carrier family 26, member 8
116931	MED12L	mediator complex subunit 12-like
117246	FTSJ3	FtsJ homolog 3 (E. coli)
117532	TMC2	transmembrane channel-like 2
1181	CLCN2	chloride channel 2
1182	CLCN3	chloride channel 3
118427	OLFM3	olfactomedin 3
1187	CLCNKA	chloride channel Ka
1188	CLCNKB	chloride channel Kb
119710	C11orf74	chromosome 11 open reading frame 74
120071	GYLTL1B	glycosyltransferase-like 1B
121340	SP7	Sp7 transcription factor
123036	TC2N	tandem C2 domains, nuclear
124641	OVCA2	ovarian tumor suppressor candidate 2
124872	B4GALNT2	beta-1,4-N-acetyl-galactosaminyl transferase 2
124925	SEZ6	seizure related 6 homolog (mouse)
124995	MRPL10	mitochondrial ribosomal protein L10
125488	TTC39C	tetratricopeptide repeat domain 39C
1265	CNN2	calponin 2
1267	CNP	2',3'-cyclic nucleotide 3' phosphodiesterase
127544	RNF19B	ring finger protein 19B
128553	TSHZ2	teashirt zinc finger homeobox 2
130271	PLEKHH2	pleckstrin homology domain containing, family H (with MyTH4 domain) member 2
130497	OSR1	odd-skipped related 1 (Drosophila)
130888	FBXO36	F-box protein 36
132014	IL17RE	interleukin 17 receptor E
132243	H1FOO	H1 histone family, member O, oocyte-specific
136319	MTPN	myotrophin
139886	SPIN4	spindlin family, member 4
141	ADPRH	ADP-ribosylarginine hydrolase
1410	CRYAB	crystallin, alpha B
144363	LYRM5	LYR motif containing 5
144404	TMEM120B	transmembrane protein 120B
146057	TTBK2	tau tubulin kinase 2
148281	SYT6	synaptotagmin VI
148979	GLIS1	GLIS family zinc finger 1
149111	CNIH3	cornichon homolog 3 (Drosophila)
149603	RNF187	ring finger protein 187
149951	COMMD7	COMM domain containing 7
1506	CTRL	chymotrypsin-like
151188	ARL6IP6	ADP-ribosylation-like factor 6 interacting protein 6
151987	PPP4R2	protein phosphatase 4, regulatory subunit 2
152189	CMTM8	CKLF-like MARVEL transmembrane domain containing 8
152330	CNTN4	contactin 4
160335	TMTC2	transmembrane and tetratricopeptide repeat containing 2
161291	TMEM30B	transmembrane protein 30B
161582	DYX1C1	dyslexia susceptibility 1 candidate 1
162494	RHBDL3	rhomboid, veinlet-like 3 (Drosophila)
162605	KRT28	keratin 28
165545	DQX1	DEAQ box RNA-dependent ATPase 1
166378	SPATA5	spermatogenesis associated 5
170463	SSBP4	single stranded DNA binding protein 4
170961	ANKRD24	ankyrin repeat domain 24
171586	ABHD3	abhydrolase domain containing 3
1740	DLG2	discs, large homolog 2 (Drosophila)
1747	DLX3	distal-less homeobox 3
1760	DMPK	dystrophia myotonica-protein kinase
1776	DNASE1L3	deoxyribonuclease I-like 3
1804	DPP6	dipeptidyl-peptidase 6
1822	ATN1	atrophin 1
1911	PHC1	polyhomeotic homolog 1 (Drosophila)
1993	ELAVL2	ELAV (embryonic lethal, abnormal vision, Drosophila)-like 2 (Hu antigen B)
1995	ELAVL3	ELAV (embryonic lethal, abnormal vision, Drosophila)-like 3 (Hu antigen C)
199699	DAND5	DAN domain family, member 5
199746	U2AF1L4	U2 small nuclear RNA auxiliary factor 1-like 4
200185	KRTCAP2	keratinocyte associated protein 2
201191	SAMD14	sterile alpha motif domain containing 14
201254	STRA13	stimulated by retinoic acid 13 homolog (mouse)
2019	EN1	engrailed homeobox 1
2070	EYA4	eyes absent homolog 4 (Drosophila)
2140	EYA3	eyes absent homolog 3 (Drosophila)
2145	EZH1	enhancer of zeste homolog 1 (Drosophila)
2186	BPTF	bromodomain PHD finger transcription factor
221035	REEP3	receptor accessory protein 3
221184	CPNE2	copine II
222068	TMED4	transmembrane emp24 protein transport domain containing 4
22807	IKZF2	IKAROS family zinc finger 2 (Helios)
2281	FKBP1B	FK506 binding protein 1B, 12.6 kDa
22839	DLGAP4	discs, large (Drosophila) homolog-associated protein 4
22858	ICK	intestinal cell (MAK-like) kinase
22883	CLSTN1	calsyntenin 1
22902	RUFY3	RUN and FYVE domain containing 3
22906	TRAK1	trafficking protein, kinesin binding 1
22913	RALY	RNA binding protein, autoantigenic (hnRNP-associated with lethal yellow homolog (mouse))
22941	SHANK2	SH3 and multiple ankyrin repeat domains 2
2297	FOXD1	forkhead box D1
22983	MAST1	microtubule associated serine/threonine kinase 1
22990	PCNX	pecanex homolog (Drosophila)
23	ABCF1	ATP-binding cassette, sub-family F (GCN20), member 1
23024	PDZRN3	PDZ domain containing ring finger 3
23025	UNC13A	unc-13 homolog A (C. elegans)

2303	FOXC2	forkhead box C2 (MFH-1, mesenchyme forkhead 1)
23034	SAMD4A	sterile alpha motif domain containing 4A
23076	RRP1B	ribosomal RNA processing 1 homolog B (S. cerevisiae)
23082	PPRC1	peroxisome proliferator-activated receptor gamma, coactivator-related 1
23095	KIF1B	kinesin family member 1B
23130	ATG2A	ATG2 autophagy related 2 homolog A (S. cerevisiae)
23142	DCUN1D4	DCN1, defective in cullin neddylation 1, domain containing 4 (S. cerevisiae)
23150	FRMD4B	FERM domain containing 4B
23152	CIC	capicua homolog (Drosophila)
23154	NCDN	neurochondrin
23179	RGL1	ral guanine nucleotide dissociation stimulator-like 1
23184	MESDC2	mesoderm development candidate 2
23187	PHLDB1	pleckstrin homology-like domain, family B, member 1
23210	JMJD6	jumonji domain containing 6
23212	RRS1	RRS1 ribosome biogenesis regulator homolog (S. cerevisiae)
23216	TBC1D1	TBC1 (tre-2/USP6, BUB2, cdc16) domain family, member 1
23228	PLCL2	phospholipase C-like 2
23269	MGA	MAX gene associated
23321	TRIM2	tripartite motif containing 2
23328	SASH1	SAM and SH3 domain containing 1
23406	COTL1	coactosin-like 1 (Dictyostelium)
2342	FNTB	farnesyltransferase, CAAX box, beta
23435	TARDBP	TAR DNA binding protein
23512	SUZ12	suppressor of zeste 12 homolog (Drosophila)
23536	ADAT1	adenosine deaminase, tRNA-specific 1
23554	TSPAN12	tetraspanin 12
23564	DDAH2	dimethylarginine dimethylaminohydrolase 2
23567	ZNF346	zinc finger protein 346
23584	VSIG2	V-set and immunoglobulin domain containing 2
23637	RABGAP1	RAB GTPase activating protein 1
23639	LRRC6	leucine rich repeat containing 6
23648	SSBP3	single stranded DNA binding protein 3
23678	SGK3	serum/glucocorticoid regulated kinase family, member 3
23788	MTCH2	mitochondrial carrier 2
238	ALK	anaplastic lymphoma receptor tyrosine kinase
253738	EBF3	early B-cell factor 3
254102	EHBP1L1	EH domain binding protein 1-like 1
254240	BPIFC	BPI fold containing family C
254394	MCM9	minichromosome maintenance complex component 9
255061	TAC4	tachykinin 4 (hemokinin)
25797	QPCT	glutaminyl-peptide cyclotransferase
25801	GCA	grancalcin, EF-hand calcium binding protein
25806	VAX2	ventral anterior homeobox 2
25807	RHBDD3	rhomboid domain containing 3
25816	TNFAIP8	tumor necrosis factor, alpha-induced protein 8
25842	ASF1A	ASF1 anti-silencing function 1 homolog A (S. cerevisiae)
25917	THUMPD3	THUMP domain containing 3
25925	ZNF521	zinc finger protein 521
25979	DHRS7B	dehydrogenase/reductase (SDR family) member 7B
26002	MOXD1	monooxygenase, DBH-like 1
26022	TMEM98	transmembrane protein 98
26053	AUTS2	autism susceptibility candidate 2
26057	ANKRD17	ankyrin repeat domain 17
26140	TTLL3	tubulin tyrosine ligase-like family, member 3
261729	STEAP2	STEAP family member 2, metalloreductase
26207	PITPNC1	phosphatidylinositol transfer protein, cytoplasmic 1
2622	GAS8	growth arrest-specific 8
26268	FBXO9	F-box protein 9
26284	ERAL1	Era G-protein-like 1 (E. coli)
2637	GBX2	gastrulation brain homeobox 2
26469	PTPN18	protein tyrosine phosphatase, non-receptor type 18 (brain-derived)
26499	PLEK2	pleckstrin 2
26512	INTS6	integrator complex subunit 6
266743	NPAS4	neuronal PAS domain protein 4
26960	NBEA	neurobeachin
27000	DNAJC2	DnaJ (Hsp40) homolog, subfamily C, member 2
27022	FOXD3	forkhead box D3
27075	TSPAN13	tetraspanin 13
27077	B9D1	B9 protein domain 1
27095	TRAPPC3	trafficking protein particle complex 3
27129	HSPB7	heat shock 27kDa protein family, member 7 (cardiovascular)
27239	GPR162	G protein-coupled receptor 162
27240	SIT1	signaling threshold regulating transmembrane adaptor 1
27245	AHDC1	AT hook, DNA binding motif, containing 1
27247	NFU1	NFU1 iron-sulfur cluster scaffold homolog (S. cerevisiae)
27440	CECR5	cat eye syndrome chromosome region, candidate 5
283578	TMED8	transmembrane emp24 protein transport domain containing 8
283659	PRTG	protogenin
284086	NEK8	NIMA (never in mitosis gene a)- related kinase 8
284406	ZFP82	zinc finger protein 82 homolog (mouse)
284434	NWD1	NACHT and WD repeat domain containing 1
284612	SYPL2	synaptophysin-like 2
285382	C3orf70	chromosome 3 open reading frame 70
2873	GPS1	G protein pathway suppressor 1
28981	IFT81	intraflagellar transport 81 homolog (Chlamydomonas)
28985	MCTS1	malignant T cell amplified sequence 1
28991	COMMD5	COMM domain containing 5
29105	C16orf80	chromosome 16 open reading frame 80
29789	OLA1	Obg-like ATPase 1
29855	UBN1	ubinuclein 1
29903	CCDC106	coiled-coil domain containing 106
29911	HOOK2	hook homolog 2 (Drosophila)
29943	PADI1	peptidyl arginine deiminase, type I
29966	STRN3	striatin, calmodulin binding protein 3
29978	UBQLN2	ubiquilin 2
29980	DONSON	downstream neighbor of SON
30010	NXPH1	neurexophilin 1
3069	HDLBP	high density lipoprotein binding protein
30820	KCNIP1	Kv channel interacting protein 1
30850	CDR2L	cerebellar degeneration-related protein 2-like
3131	HLF	hepatic leukemia factor
3140	MR1	major histocompatibility complex, class I-related
3199	HOXA2	homeobox A2
3205	HOXA9	homeobox A9
3219	HOXB9	homeobox B9
3221	HOXC4	homeobox C4
3222	HOXC5	homeobox C5
3223	HOXC6	homeobox C6
3224	HOXC8	homeobox C8
3225	HOXC9	homeobox C9
3231	HOXD1	homeobox D1
3232	HOXD3	homeobox D3
3234	HOXD8	homeobox D8
3235	HOXD9	homeobox D9
3236	HOXD10	homeobox D10
3275	PRMT2	protein arginine methyltransferase 2
338440	ANO9	anoctamin 9
339324	ZNF260	zinc finger protein 260
339488	TFAP2E	transcription factor AP-2 epsilon (activating enhancer binding protein 2 epsilon)
339665	SLC35E4	solute carrier family 35, member E4
340348	TSPAN33	tetraspanin 33
341	APOC1	apolipoprotein C-I
341405	ANKRD33	ankyrin repeat domain 33
342667	STAC2	SH3 and cysteine rich domain 2
342865	VSTM2B	V-set and transmembrane domain containing 2B
346	APOC4	apolipoprotein C-IV
347730	LRRTM1	leucine rich repeat transmembrane neuronal 1
347731	LRRTM3	leucine rich repeat transmembrane neuronal 3
3608	ILF2	interleukin enhancer binding factor 2, 45kDa
3609	ILF3	interleukin enhancer binding factor 3, 90kDa
375248	ANKRD36	ankyrin repeat domain 36
375775	PNPLA7	patatin-like phospholipase domain containing 7
379	ARL4D	ADP-ribosylation factor-like 4D
3800	KIF5C	kinesin family member 5C
3858	KRT10	keratin 10
387338	NSUN4	NOP2/Sun domain family, member 4
388595	TMEM82	transmembrane protein 82
388789	LINC00493	long intergenic non-protein coding RNA 493
389118	CDHR4	cadherin-related family member 4
389792	IER5L	immediate early response 5-like
390	RND3	Rho family GTPase 3
3902	LAG3	lymphocyte-activation gene 3
3927	LASP1	LIM and SH3 protein 1
3975	LHX1	LIM homeobox 1
400	ARL1	ADP-ribosylation factor-like 1
401397	LOC401397	uncharacterized LOC401397
404093	CUEDC1	CUE domain containing 1
4045	LSAMP	limbic system-associated membrane protein
4076	CAPRIN1	cell cycle associated protein 1
4114	MAGEB3	melanoma antigen family B, 3
4162	MCAM	melanoma cell adhesion molecule
4212	MEIS2	Meis homeobox 2
4234	METTL1	methyltransferase like 1
4241	MFI2	antigen p97 (melanoma associated) identified by monoclonal antibodies 133.2 and 96.5
4340	MOG	myelin oligodendrocyte glycoprotein
440590	ZYG11A	zyg-11 homolog A (C. elegans)
442425	FOXB2	forkhead box B2
4440	MSI1	musashi homolog 1 (Drosophila)
4487	MSX1	msh homeobox 1
4489	MT1A	metallothionein 1A
4542	MYO1F	myosin IF
4601	MXI1	MAX interactor 1
4613	MYCN	v-myc myelocytomatosis viral related oncogene, neuroblastoma derived (avian)
4641	MYO1C	myosin IC
4665	NAB2	NGFI-A binding protein 2 (EGR1 binding protein 2)
4681	NBL1	neuroblastoma, suppression of tumorigenicity 1
4739	NEDD9	neural precursor cell expressed, developmentally down-regulated 9
475	ATOX1	ATX1 antioxidant protein 1 homolog (yeast)
4784	NFIX	nuclear factor I/X (CCAAT-binding transcription factor)
4815	NINJ2	ninjurin 2
4857	NOVA1	neuro-oncological ventral antigen 1
4867	NPHP1	nephronophthisis 1 (juvenile)
4883	NPR3	natriuretic peptide receptor C/guanylate cyclase C (atrionatriuretic peptide receptor C)
4900	NRGN	neurogranin (protein kinase C substrate, RC3)
493911	PHOSPHO2	phosphatase, orphan 2
5015	OTX2	orthodenticle homeobox 2
50626	CYHR1	cysteine/histidine-rich 1

5064	PALM	paralemmin
5081	PAX7	paired box 7
50810	HDGFRP3	hepatoma-derived growth factor, related protein 3
5090	PBX3	pre-B-cell leukemia homeobox 3
50999	TMED5	transmembrane emp24 protein transport domain containing 5
51019	CCDC53	coiled-coil domain containing 53
51024	FIS1	fission 1 (mitochondrial outer membrane) homolog (S. cerevisiae)
51087	YBX2	Y box binding protein 2
5110	PCMT1	protein-L-isoaspartate (D-aspartate) O-methyltransferase
51192	CKLF	chemokine-like factor
51258	MRPL51	mitochondrial ribosomal protein L51
51264	MRPL27	mitochondrial ribosomal protein L27
51278	IER5	immediate early response 5
5128	CDK17	cyclin-dependent kinase 17
51290	ERGIC2	ERGIC and golgi 2
51299	NRN1	neuritin 1
51310	SLC22A17	solute carrier family 22, member 17
51317	PHF21A	PHD finger protein 21A
51364	ZMYND10	zinc finger, MYND-type containing 10
51365	PLA1A	phospholipase A1 member A
51367	POP5	processing of precursor 5, ribonuclease P/MRP subunit (S. cerevisiae)
51530	ZC3HC1	zinc finger, C3HC-type containing 1
51559	NT5DC3	5'-nucleotidase domain containing 3
51596	CUTA	cutA divalent cation tolerance homolog (E. coli)
51629	SLC25A39	solute carrier family 25, member 39
51631	LUC7L2	LUC7-like 2 (S. cerevisiae)
51649	MRPS23	mitochondrial ribosomal protein S23
51650	MRPS33	mitochondrial ribosomal protein S33
51663	ZFR	zinc finger RNA binding protein
51667	NUB1	negative regulator of ubiquitin-like proteins 1
51751	HIGD1B	HIG1 hypoxia inducible domain family, member 1B
51759	C9orf78	chromosome 9 open reading frame 78
51762	RAB8B	RAB8B, member RAS oncogene family
51768	TM7SF3	transmembrane 7 superfamily member 3
51804	SIX4	SIX homeobox 4
51805	COQ3	coenzyme Q3 homolog, methyltransferase (S. cerevisiae)
5309	PITX3	paired-like homeodomain 3
5326	PLAGL2	pleiomorphic adenoma gene-like 2
53335	BCL11A	B-cell CLL/lymphoma 11A (zinc finger protein)
53353	LRP1B	low density lipoprotein receptor-related protein 1B
5359	PLSCR1	phospholipid scramblase 1
54014	BRWD1	bromodomain and WD repeat domain containing 1
54020	SLC37A1	solute carrier family 37 (glycerol-3-phosphate transporter), member 1
5428	POLG	polymerase (DNA directed), gamma
5447	POR	P450 (cytochrome) oxidoreductase
54475	NLE1	notchless homolog 1 (Drosophila)
54477	PLEKHA5	pleckstrin homology domain containing, family A member 5
54542	RC3H2	ring finger and CCCH-type domains 2
5459	POU4F3	POU class 4 homeobox 3
54726	OTUD4	OTU domain containing 4
54765	TRIM44	tripartite motif containing 44
54766	BTG4	B-cell translocation gene 4
54796	BNC2	basonuclin 2
54806	AHI1	Abelson helper integration site 1
54810	GIPC2	GIPC PDZ domain containing family, member 2
54812	AFTPH	aftiphilin
54822	TRPM7	transient receptor potential cation channel, subfamily M, member 7
54828	BCAS3	breast carcinoma amplified sequence 3
54845	ESRP1	epithelial splicing regulatory protein 1
54847	SIDT1	SID1 transmembrane family, member 1
54866	PPP1R14D	protein phosphatase 1, regulatory (inhibitor) subunit 14D
54915	YTHDF1	YTH domain family, member 1
54927	CHCHD3	coiled-coil-helix-coiled-coil-helix domain containing 3
54929	TMEM161A	transmembrane protein 161A
54972	TMEM132A	transmembrane protein 132A
54987	C1orf123	chromosome 1 open reading frame 123
550	AUP1	ancient ubiquitous protein 1
55012	PPP2R3C	protein phosphatase 2, regulatory subunit B'', gamma
55023	PHIP	pleckstrin homology domain interacting protein
55032	SLC35A5	solute carrier family 35, member A5
55080	TAPBPL	TAP binding protein-like
55103	RALGPS2	Ral GEF with PH domain and SH3 binding motif 2
5511	PPP1R8	protein phosphatase 1, regulatory subunit 8
55137	FIGN	fidgetin
55161	TMEM33	transmembrane protein 33
55165	CEP55	centrosomal protein 55kDa
55170	PRMT6	protein arginine methyltransferase 6
55186	SLC25A36	solute carrier family 25, member 36
55196	C12orf35	chromosome 12 open reading frame 35
55204	GOLPH3L	golgi phosphoprotein 3-like
55207	ARL8B	ADP-ribosylation factor-like 8B
55222	LRRC20	leucine rich repeat containing 20
55269	PSPC1	paraspeckle component 1
55283	MCOLN3	mucolipin 3
552900	BOLA2	bolA homolog 2 (E. coli)
55297	CCDC91	coiled-coil domain containing 91
55298	RNF121	ring finger protein 121
55324	ABCF3	ATP-binding cassette, sub-family F (GCN20), member 3
55333	SYNJ2BP	synaptojanin 2 binding protein
55339	WDR33	WD repeat domain 33
55342	STRBP	spermatid perinuclear RNA binding protein
55379	LRRC59	leucine rich repeat containing 59
55450	CAMK2N1	calcium/calmodulin-dependent protein kinase II inhibitor 1
5550	PREP	prolyl endopeptidase
55529	TMEM55A	transmembrane protein 55A
55643	BTBD2	BTB (POZ) domain containing 2
55652	SLC48A1	solute carrier family 48 (heme transporter), member 1
55704	CCDC88A	coiled-coil domain containing 88A
55737	VPS35	vacuolar protein sorting 35 homolog (S. cerevisiae)
55764	IFT122	intraflagellar transport 122 homolog (Chlamydomonas)
55823	VPS11	vacuolar protein sorting 11 homolog (S. cerevisiae)
55832	CAND1	cullin-associated and neddylation-dissociated 1
55840	EAF2	ELL associated factor 2
55890	GPRC5C	G protein-coupled receptor, family C, group 5, member C
55892	MYNN	myoneurin
55893	ZNF395	zinc finger protein 395
55915	LANCL2	LanC lantibiotic synthetase component C-like 2 (bacterial)
55954	ZMAT5	zinc finger, matrin-type 5
56033	BARX1	BARX homeobox 1
56155	TEX14	testis expressed 14
56204	KIAA1370	KIAA1370
56245	C21orf62	chromosome 21 open reading frame 62
56254	RNF20	ring finger protein 20
5639	PRRG2	proline rich Gla (G-carboxyglutamic acid) 2
56681	SAR1A	SAR1 homolog A (S. cerevisiae)
56683	C21orf59	chromosome 21 open reading frame 59
56889	TM9SF3	transmembrane 9 superfamily member 3
56927	GPR108	G protein-coupled receptor 108
56943	ENY2	enhancer of yellow 2 homolog (Drosophila)
56944	OLFML3	olfactomedin-like 3
56957	OTUD7B	OTU domain containing 7B
56961	SHD	Src homology 2 domain containing transforming protein D
56987	BBX	bobby sox homolog (Drosophila)
57010	CABP4	calcium binding protein 4
57057	TBX20	T-box 20
57092	PCNP	PEST proteolytic signal containing nuclear protein
57198	ATP8B2	ATPase, class I, type 8B, member 2
57226	LYRM2	LYR motif containing 2
57326	PBXIP1	pre-B-cell leukemia homeobox interacting protein 1
57451	ODZ2	odz, odd Oz/ten-m homolog 2 (Drosophila)
57537	SORCS2	sortilin-related VPS10 domain containing receptor 2
57561	ARRDC3	arrestin domain containing 3
57575	PCDH10	protocadherin 10
57576	KIF17	kinesin family member 17
57617	VPS18	vacuolar protein sorting 18 homolog (S. cerevisiae)
5763	PTMS	parathymosin
57633	LRRN1	leucine rich repeat neuronal 1
57664	PLEKHA4	pleckstrin homology domain containing, family A (phosphoinositide binding specific) member 4
577	BAI3	brain-specific angiogenesis inhibitor 3
57713	SFMBT2	Scm-like with four mbt domains 2
57716	PRX	periaxin
5780	PTPN9	protein tyrosine phosphatase, non-receptor type 9
5782	PTPN12	protein tyrosine phosphatase, non-receptor type 12
57822	GRHL3	grainyhead-like 3 (Drosophila)
5790	PTPRCAP	protein tyrosine phosphatase, receptor type, C-associated protein
5813	PURA	purine-rich element binding protein A
58499	ZNF462	zinc finger protein 462
5866	RAB3IL1	RAB3A interacting protein (rabin3)-like 1
59274	MESDC1	mesoderm development candidate 1
5939	RBMS2	RNA binding motif, single stranded interacting protein 2
5991	RFX3	regulatory factor X, 3 (influences HLA class II expression)
6001	RGS10	regulator of G-protein signaling 10
6004	RGS16	regulator of G-protein signaling 16
6007	RHD	Rh blood group, D antigen
6017	RLBP1	retinaldehyde binding protein 1
60509	AGBL5	ATP/GTP binding protein-like 5
60675	PROK2	prokineticin 2
60681	FKBP10	FK506 binding protein 10, 65 kDa
6253	RTN2	reticulon 2
6297	SALL2	sal-like 2 (Drosophila)
6299	SALL1	sal-like 1 (Drosophila)
6302	TSPAN31	tetraspanin 31
63876	PKNOX2	PBX/knotted 1 homeobox 2
63905	MANBAL	mannosidase, beta A, lysosomal-like
63924	CIDEC	cell death-inducing DFFA-like effector c
63973	NEUROG2	neurogenin 2
64067	NPAS3	neuronal PAS domain protein 3
64083	GOLPH3	golgi phosphoprotein 3 (coat-protein)
64097	EPB41L4A	erythrocyte membrane protein band 4.1 like 4A
6415	SEPW1	selenoprotein W, 1
64211	LHX5	LIM homeobox 5
64220	STRA6	stimulated by retinoic acid gene 6 homolog (mouse)
64359	NXN	nucleoredoxin
64375	IKZF4	IKAROS family zinc finger 4 (Eos)
64405	CDH22	cadherin 22, type 2
64410	KLHL25	kelch-like 25 (Drosophila)
645638	LOC645638	WDNM1-like pseudogene

64641	EBF2	early B-cell factor 2
647024	C6orf132	chromosome 6 open reading frame 132
64748	LPPR2	lipid phosphate phosphatase-related protein type 2
64852	TUT1	terminal uridylyl transferase 1, U6 snRNA-specific
64901	RANBP17	RAN binding protein 17
6495	SIX1	SIX homeobox 1
6496	SIX3	SIX homeobox 3
64981	MRPL34	mitochondrial ribosomal protein L34
65009	NDRG4	NDRG family member 4
65083	NOL6	nucleolar protein family 6 (RNA-associated)
65117	RSRC2	arginine/serine-rich coiled-coil 2
65125	WNK1	WNK lysine deficient protein kinase 1
65266	WNK4	WNK lysine deficient protein kinase 4
653677	SEC1	secretory blood group 1
654790	PCP4L1	Purkinje cell protein 4 like 1
66005	CHID1	chitinase domain containing 1
6612	SUMO3	SMT3 suppressor of mif two 3 homolog 3 (S. cerevisiae)
6641	SNTB1	syntrophin, beta 1 (dystrophin-associated protein A1, 59kDa, basic component 1)
6651	SON	SON DNA binding protein
6659	SOX4	SRY (sex determining region Y)-box 4
6692	SPINT1	serine peptidase inhibitor, Kunitz type 1
676	BRDT	bromodomain, testis-specific
6780	STAU1	staufen, RNA binding protein, homolog 1 (Drosophila)
687	KLF9	Kruppel-like factor 9
6886	TAL1	T-cell acute lymphocytic leukemia 1
6913	TBX15	T-box 15
6926	TBX3	T-box 3
6939	TCF15	transcription factor 15 (basic helix-loop-helix)
6942	TCF20	transcription factor 20 (AR1)
6944	VPS72	vacuolar protein sorting 72 homolog (S. cerevisiae)
6954	TCP11	t-complex 11 homolog (mouse)
696	BTN1A1	butyrophilin, subfamily 1, member A1
705	BYSL	bystin-like
7072	TIA1	TIA1 cytotoxic granule-associated RNA binding protein
7073	TIAL1	TIA1 cytotoxic granule-associated RNA binding protein-like 1
7087	ICAM5	intercellular adhesion molecule 5, telencephalin
7265	TTC1	tetratricopeptide repeat domain 1
7270	TTF1	transcription termination factor, RNA polymerase I
7288	TULP2	tubby like protein 2
7289	TULP3	tubby like protein 3
728927	ZNF736	zinc finger protein 736
7290	HIRA	HIR histone cell cycle regulation defective homolog A (S. cerevisiae)
7415	VCP	valosin containing protein
744	MPPED2	metallophosphoesterase domain containing 2
7441	VPREB1	pre-B lymphocyte 1
754	PTTG1IP	pituitary tumor-transforming 1 interacting protein
7545	ZIC1	Zic family member 1
7701	ZNF142	zinc finger protein 142
7703	PCGF2	polycomb group ring finger 2
7707	ZNF148	zinc finger protein 148
7726	TRIM26	tripartite motif containing 26
780	DDR1	discoidin domain receptor tyrosine kinase 1
7866	IFRD2	interferon-related developmental regulator 2
78997	GDAP1L1	ganglioside-induced differentiation-associated protein 1-like 1
79004	CUEDC2	CUE domain containing 2
79007	DBNDD1	dysbindin (dystrobrevin binding protein 1) domain containing 1
79026	AHNAK	AHNAK nucleoprotein
79070	KDELC1	KDEL (Lys-Asp-Glu-Leu) containing 1
79073	TMEM109	transmembrane protein 109
79152	FA2H	fatty acid 2-hydroxylase
79153	GDPD3	glycerophosphodiester phosphodiesterase domain containing 3
79157	MFSD11	major facilitator superfamily domain containing 11
79176	FBXL15	F-box and leucine-rich repeat protein 15
79411	GLB1L	galactosidase, beta 1-like
79577	CDC73	cell division cycle 73, Paf1/RNA polymerase II complex component, homolog (S. cerevisiae)
79630	C1orf54	chromosome 1 open reading frame 54
79734	KCTD17	potassium channel tetramerisation domain containing 17
79776	ZFHX4	zinc finger homeobox 4
79789	CLMN	calmin (calponin-like, transmembrane)
79848	CSPP1	centrosome and spindle pole associated protein 1
79877	DCAKD	dephospho-CoA kinase domain containing
79893	GGNBP2	gametogenetin binding protein 2
79932	KIAA0319L	KIAA0319-like
79971	WLS	wntless homolog (Drosophila)
79977	GRHL2	grainyhead-like 2 (Drosophila)
80019	UBTD1	ubiquitin domain containing 1
80022	MYO15B	myosin XVB pseudogene
80131	LRRC8E	leucine rich repeat containing 8 family, member E
80167	C4orf29	chromosome 4 open reading frame 29
80173	IFT74	intraflagellar transport 74 homolog (Chlamydomonas)
80279	CDK5RAP3	CDK5 regulatory subunit associated protein 3
8028	MLLT10	myeloid/lymphoid or mixed-lineage leukemia (trithorax homolog, Drosophila); translocated to, 10
80315	CPEB4	cytoplasmic polyadenylation element binding protein 4
80318	GKAP1	G kinase anchoring protein 1
80320	SP6	Sp6 transcription factor
80351	TNKS2	tankyrase, TRF1-interacting ankyrin-related ADP-ribose polymerase 2
8036	SHOC2	soc-2 suppressor of clear homolog (C. elegans)
80709	AKNA	AT-hook transcription factor
8079	MLF2	myeloid leukemia factor 2
8091	HMGA2	high mobility group AT-hook 2
80975	TMPRSS5	transmembrane protease, serine 5
8110	DPF3	D4, zinc and double PHD fingers, family 3
81544	GDPD5	glycerophosphodiester phosphodiesterase domain containing 5
81572	PDRG1	p53 and DNA-damage regulated 1
81602	CDADC1	cytidine and dCMP deaminase domain containing 1
81603	TRIM8	tripartite motif containing 8
81605	URM1	ubiquitin related modifier 1
81611	ANP32E	acidic (leucine-rich) nuclear phosphoprotein 32 family, member E
81618	ITM2C	integral membrane protein 2C
81629	TSSK3	testis-specific serine kinase 3
81786	TRIM7	tripartite motif containing 7
81796	SLCO5A1	solute carrier organic anion transporter family, member 5A1
81846	SBF2	SET binding factor 2
81889	FAHD1	fumarylacetoacetate hydrolase domain containing 1
8225	GTPBP6	GTP binding protein 6 (putative)
825	CAPN3	calpain 3, (p94)
8289	ARID1A	AT rich interactive domain 1A (SWI-like)
8315	BRAP	BRCA1 associated protein
83440	ADPGK	ADP-dependent glucokinase
83461	CDCA3	cell division cycle associated 3
83546	RTBDN	retbindin
83591	THAP2	THAP domain containing, apoptosis associated protein 2
83597	RTP3	receptor (chemosensory) transporter protein 3
83694	RPS6KL1	ribosomal protein S6 kinase-like 1
83698	CALN1	calneuron 1
83715	ESPN	espin
83742	MARVELD1	MARVEL domain containing 1
83759	RBM4B	RNA binding motif protein 4B
83871	RAB34	RAB34, member RAS oncogene family
83931	STK40	serine/threonine kinase 40
84072	HORMAD1	HORMA domain containing 1
84073	MYCBPAP	MYCBP associated protein
84107	ZIC4	Zic family member 4
84108	PCGF6	polycomb group ring finger 6
84159	ARID5B	AT rich interactive domain 5B (MRF1-like)
84163	GTF2IRD2	GTF2I repeat domain containing 2
84196	USP48	ubiquitin specific peptidase 48
84251	SGIP1	SH3-domain GRB2-like (endophilin) interacting protein 1
84258	SYT3	synaptotagmin III
84274	COQ5	coenzyme Q5 homolog, methyltransferase (S. cerevisiae)
84288	EFCAB2	EF-hand calcium binding domain 2
84311	MRPL45	mitochondrial ribosomal protein L45
8444	DYRK3	dual-specificity tyrosine-(Y)-phosphorylation regulated kinase 3
84561	SLC12A8	solute carrier family 12 (potassium/chloride transporters), member 8
8459	TPST2	tyrosylprotein sulfotransferase 2
84662	GLIS2	GLIS family zinc finger 2
84667	HES7	hairy and enhancer of split 7 (Drosophila)
84750	FUT10	fucosyltransferase 10 (alpha (1,3) fucosyltransferase)
84759	PCGF1	polycomb group ring finger 1
84814	PPAPDC3	phosphatidic acid phosphatase type 2 domain containing 3
84836	ABHD14B	abhydrolase domain containing 14B
84858	ZNF503	zinc finger protein 503
84930	MASTL	microtubule associated serine/threonine kinase-like
84932	RAB2B	RAB2B, member RAS oncogene family
84937	ZNRF1	zinc and ring finger 1
84955	NUDCD1	NudC domain containing 1
85004	RERG	RAS-like, estrogen-regulated, growth inhibitor
85014	TMEM141	transmembrane protein 141
85364	ZCCHC3	zinc finger, CCHC domain containing 3
85416	ZIC5	Zic family member 5
85451	UNK	unkempt homolog (Drosophila)
85461	TANC1	tetratricopeptide repeat, ankyrin repeat and coiled-coil containing 1
85474	LBX2	ladybird homeobox 2
8549	LGR5	leucine-rich repeat containing G protein-coupled receptor 5
8609	KLF7	Kruppel-like factor 7 (ubiquitous)
8614	STC2	stanniocalcin 2
8880	FUBP1	far upstream element (FUSE) binding protein 1
8899	PRPF4B	PRP4 pre-mRNA processing factor 4 homolog B (yeast)
8975	USP13	ubiquitin specific peptidase 13 (isopeptidase T-3)
89765	RSPH1	radial spoke head 1 homolog (Chlamydomonas)
89866	SEC16B	SEC16 homolog B (S. cerevisiae)
8994	LIMD1	LIM domains containing 1
8999	CDKL2	cyclin-dependent kinase-like 2 (CDC2-related kinase)
90007	MIDN	midnolin
90102	PHLDB2	pleckstrin homology-like domain, family B, member 2
9031	BAZ1B	bromodomain adjacent to zinc finger domain, 1B
9032	TM4SF5	transmembrane 4 L six family member 5
90324	CCDC97	coiled-coil domain containing 97
90480	GADD45GIP1	growth arrest and DNA-damage-inducible, gamma interacting protein 1
9049	AIP	aryl hydrocarbon receptor interacting protein
9050	PSTPIP2	proline-serine-threonine phosphatase interacting protein 2
90506	LRRC46	leucine rich repeat containing 46
90507	SCRN2	secernin 2
9066	SYT7	synaptotagmin VII
90843	TCEAL8	transcription elongation factor A (SII)-like 8
9096	TBX18	T-box 18
90990	KIFC2	kinesin family member C2

9124	PDLIM1	PDZ and LIM domain 1
9143	SYNGR3	synaptogyrin 3
91683	SYT12	synaptotagmin XII
91754	NEK9	NIMA (never in mitosis gene a)- related kinase 9
9201	DCLK1	doublecortin-like kinase 1
92305	TMEM129	transmembrane protein 129
9238	TBRG4	transforming growth factor beta regulator 4
9275	BCL7B	B-cell CLL/lymphoma 7B
92797	HELB	helicase (DNA) B
93145	OLFM2	olfactomedin 2
9325	TRIP4	thyroid hormone receptor interactor 4
93436	ARMC6	armadillo repeat containing 6
9352	TXNL1	thioredoxin-like 1
9355	LHX2	LIM homeobox 2
9364	RAB28	RAB28, member RAS oncogene family
93664	CADPS2	Ca++-dependent secretion activator 2
9373	PLAA	phospholipase A2-activating protein
93974	ATPIF1	ATPase inhibitory factor 1
94015	TTYH2	tweety homolog 2 (Drosophila)
94032	CAMK2N2	calcium/calmodulin-dependent protein kinase II inhibitor 2
9404	LPXN	leupaxin
9462	RASAL2	RAS protein activator like 2
9513	FXR2	fragile X mental retardation, autosomal homolog 2
9545	RAB3D	RAB3D, member RAS oncogene family
9552	SPAG7	sperm associated antigen 7
9590	AKAP12	A kinase (PRKA) anchor protein 12
9625	AATK	apoptosis-associated tyrosine kinase
9644	SH3PXD2A	SH3 and PX domains 2A
9657	IQCB1	IQ motif containing B1
9659	PDE4DIP	phosphodiesterase 4D interacting protein
9686	VGLL4	vestigial like 4 (Drosophila)
9699	RIMS2	regulating synaptic membrane exocytosis 2
9718	ECE2	endothelin converting enzyme 2
9746	CLSTN3	calsyntenin 3
9753	ZSCAN12	zinc finger and SCAN domain containing 12
9768	KIAA0101	KIAA0101
9807	IP6K1	inositol hexakisphosphate kinase 1
9853	RUSC2	RUN and SH3 domain containing 2
9859	CEP170	centrosomal protein 170kDa
987	LRBA	LPS-responsive vesicle trafficking, beach and anchor containing
9898	UBAP2L	ubiquitin associated protein 2-like
9909	DENND4B	DENN/MADD domain containing 4B
9910	RABGAP1L	RAB GTPase activating protein 1-like
9921	RNF10	ring finger protein 10
9946	CRYZL1	crystallin, zeta (quinone reductase)-like 1
